# Penumbral Rescue by normobaric O = O administration in patients with ischemic stroke and target mismatch proFile (PROOF): Study protocol of a phase IIb trial

**DOI:** 10.1177/17474930231185275

**Published:** 2023-08-18

**Authors:** Sven Poli, Joshua Mbroh, Jean-Claude Baron, Aneesh B Singhal, Daniel Strbian, Carlos Molina, Robin Lemmens, Guillaume Turc, Robert Mikulik, Patrik Michel, Turgut Tatlisumak, Heinrich J Audebert, Martin Dichgans, Roland Veltkamp, Johannes Hüsing, Holm Graessner, Jens Fiehler, Joan Montaner, Adedolapo Kamaldeen Adeyemi, Katharina Althaus, Juan F Arenillas, Benjamin Bender, Frank Benedikt, Gabriel Broocks, Ina Burghaus, Pere Cardona, Milani Deb-Chatterji, Martina Cviková, Luc Defreyne, Veerle De Herdt, Olivier Detante, Ulrike Ernemann, Fabian Flottmann, Lídia García Guillamón, Monika Glauch, Alexandra Gomez-Exposito, Benjamin Gory, Sylvie Sylvie Grand, Michal Haršány, Till Karsten Hauser, Olivier Heck, Dimitri Hemelsoet, Florian Hennersdorf, Julia Hoppe, Pia Kalmbach, Lars Kellert, Martin Köhrmann, Markus Kowarik, Blanca Lara-Rodríguez, Loic Legris, Tobias Lindig, Steffen Luntz, Jay Lusk, Brian Mac Grory, Andreas Manger, Nicolas Martinez-Majander, Annerose Mengel, Johannes Meyne, Susanne Müller, Sibu Mundiyanapurath, Olivier Naggara, Krassen Nedeltchev, Thanh N Nguyen, Maike A Nilsson, Michael Obadia, Khouloud Poli, Jan C Purrucker, Silja Räty, Sebastien Richard, Hardy Richter, Clotilde Schilte, Eckhard Schlemm, Linda Stöhr, Benjamin Stolte, Marek Sykora, Götz Thomalla, Liisa Tomppo, Noel van Horn, Julia Zeller, Ulf Ziemann, Christine S Zuern, Florian Härtig, Johannes Tuennerhoff

**Affiliations:** 1 Department of Neurology & Stroke, Eberhard-Karls University, University Hospital, Tubingen, Germany; 2 Hertie Institute for Clinical Brain Research, Eberhard-Karls University, Tubingen, Germany; 3 Department of Neurology, Hopital Sainte-Anne, Universite de Paris, Paris, France; 4 Department of Neurology, Massachusetts General Hospital, Harvard Medical School, Boston, MA, USA; 5 Department of Neurology, Helsinki University Hospital and University of Helsinki, Helsinki, Finland; 6 Department of Neurology, Vall d’Hebron University Hospital, Barcelona, Spain; 7 Department of Neurosciences, Experimental Neurology, KU Leuven, University of Leuven, Leuven, Belgium; 8 Department of Neurology, University Hospitals Leuven, Leuven, Belgium; 9 Department of Neurology, GHU Paris Psychiatrie et Neurosciences INSERM U1266 Universite Paris Cite FHU NeuroVasc, Paris, France; 10 Department of Neurology, St. Anne’s University Hospital Brno and Masaryk University, Brno, Czech Republic; 11 Neurosciences Cliniques, Centre Hospitalier Universitaire Vaudois, Lausanne, Switzerland; 12 Department of Clinical Neuroscience, Institute of Neuroscience and Physiology, Sahlgrenska Academy at University of Gothenburg, Gothenburg, Sweden; 13 Department of Neurology, Sahlgrenska University Hospital, Gothenburg, Sweden; 14 Department of Neurology and Center for Stroke Research Berlin, Charite Universitatsmedizin Berlin, Berlin, Germany; 15 Institute for Stroke and Dementia Research (ISD), University Hospital, LMU Munich, Munich, Germany; 16 Munich Cluster for Systems Neurology (SyNergy), Munich, Germany; 17 German Center for Neurodegenerative Diseases (DZNE, Munich), Munich, Germany; 18 German Centre for Cardiovascular Research (DZHK, Munich), Munich, Germany; 19 Department of Neurology, Alfried Krupp Hospital, Essen, Germany; 20 Department of Brain Sciences, Imperial College London, London, UK; 21 Coordinating Centre for Clinical Trials, University of Heidelberg, Heidelberg, Germany; 22 Landeskrebsregister Nordrhein-Westfalen, Bochum, Germany; 23 Center for Rare Diseases, Eberhard-Karls University, Tubingen, Germany; 24 Neuroradiology, University Hospital Hamburg-Eppendorf, Hamburg, Germany; 25 Eppdata GmbH, Hamburg, Germany; 26 Vall d’Hebron Institut de Recerca, Neurovascular Research Lab, Barcelona, Spain; 27 Department of Anesthesiology and Intensive Care Medicine, Eberhard-Karls University, Tubingen, Germany; 28 Department of Neurology, University Hospital of Ulm, Ulm, Germany; 29 Hospital Clinico Universitario de Valladolid, Valladolid, Spain; 30 Department of Diagnostic and Interventional Neuroradiology, Eberhard-Karls University, Tubingen, Germany; 31 Department of Neurology, University Hospital Essen, Essen, Germany; 32 Department of Neuroradiology, University Hospital Hamburg-Eppendorf, Hamburg, Germany; 33 Department of Neurology, Hospital University de Bellvitge, Barcelona, Spain; 34 Department of Neurology, University Hospital Hamburg-Eppendorf, Hamburg, Germany; 35 Department of Neurology, St. Anne’s University Hospital in Brno, Faculty of Medicine Masaryk University, Brno, Czech Republic; 36 Department of Vascular and Interventional Radiology, Ghent University Hospital, Ghent, Belgium; 37 Department of Neurology, Ghent University Hospital, Ghent, Belgium; 38 Neurology, CHU Grenoble Alpes, Grenoble, France; 39 Inserm, U1216, Grenoble Institut Neurosciences, Université Grenoble Alpes, Grenoble, France; 40 Research Management, University of Tuebingen, Tuebingen, Germany; 41 Department of Diagnostic and Therapeutic Neuroradiology, Centre Hospital Regional Universitaire de Nancy, Universite de Lorraine, INSERM U1254, Nancy, France; 42 Neuroradiology / MRI Department, CHU Grenoble Alpes, Grenoble, France; 43 International Clinical Research Centre, St. Anne’s University Hospital in Brno, Brno, Czech Republic; 44 Department of Neurology, Ludwig Maximilian University (LMU), Munich, Germany; 45 Duke University School of Medicine, Durham, NC, USA; 46 Duke Clinical Research Institute, Durham, NC, USA; 47 Department of Neurology, Duke University School of Medicine, Durham, NC, USA; 48 Department of Neurology, University Hospital Schleswig-Holstein, Kiel, Germany; 49 Department of Neurology, Heidelberg University Hospital, Heidelberg, Germany; 50 Department of Neuroradiology, GHU Paris Psychiatrie et Neurosciences INSERM U1266 Universite Paris Cite FHU NeuroVasc, Paris, France; 51 Department of Neurology, KSA Kantonsspital Aarau and University of Bern, Bern, Switzerland; 52 Department of Radiology, Boston Medical Center, Boston, MA, USA; 53 Department of Neurology, Boston Medical Center, Boston, MA, USA; 54 Department of Neurology and Stroke Center, Hopital fondation Adolphe de Rothschild, Paris, France; 55 Centre Hospital Regional Universitaire de Nancy, Nancy, France; 56 Department of Infectiology, Eberhard-Karls-University, Tuebingen, Germany; 57 Department of Anaesthesia and Critical Care, CHU Grenoble Alpes, Grenoble, France; 58 Department of Neurology, University Medical Center Hamburg-Eppendorf, Hamburg, Germany; 59 European Clinical Research Infrastructure Network (ECRIN), Paris, France; 60 Department of Neurology, St. John’s Hospital, Vienna, Austria; 61 Department of Cardiology, Universitatsspital Basel, Basel, Switzerland

**Keywords:** Normobaric oxygen therapy, NBO, hyperoxygenation, neuroprotection, penumbra, ischemic stroke, thrombectomy

## Abstract

**Rationale:**

Oxygen is essential for cellular energy metabolism. Neurons are particularly vulnerable to hypoxia. Increasing oxygen supply shortly after stroke onset could preserve the ischemic penumbra until revascularization occurs.

**Aims:**

PROOF investigates the use of normobaric oxygen (NBO) therapy within 6 h of symptom onset/notice for brain-protective bridging until endovascular revascularization of acute intracranial anterior-circulation occlusion.

**Methods and design:**

Randomized (1:1), standard treatment-controlled, open-label, blinded endpoint, multicenter adaptive phase IIb trial.

**Study outcomes:**

Primary outcome is ischemic core growth (mL) from baseline to 24 h (intention-to-treat analysis). Secondary efficacy outcomes include change in NIHSS from baseline to 24 h, mRS at 90 days, cognitive and emotional function, and quality of life. Safety outcomes include mortality, intracranial hemorrhage, and respiratory failure. Exploratory analyses of imaging and blood biomarkers will be conducted.

**Sample size:**

Using an adaptive design with interim analysis at 80 patients per arm, up to 456 participants (228 per arm) would be needed for 80% power (one-sided alpha 0.05) to detect a mean reduction of ischemic core growth by 6.68 mL, assuming 21.4 mL standard deviation.

**Discussion:**

By enrolling endovascular thrombectomy candidates in an early time window, the trial replicates insights from preclinical studies in which NBO showed beneficial effects, namely early initiation of near 100% inspired oxygen during short temporary ischemia. Primary outcome assessment at 24 h on follow-up imaging reduces variability due to withdrawal of care and early clinical confounders such as delayed extubation and aspiration pneumonia.

**Trial registrations:**

ClinicalTrials.gov: NCT03500939; EudraCT: 2017-001355-31.

## Introduction and rationale

Ischemic stroke is caused by acute occlusion of cerebral arteries leading to interruption of blood flow and consequently of oxygen supply to brain tissue. Duration and severity of ischemia are primary determinants of brain tissue damage.^1^ Because of the high energy demand of neurons and their limited capacity for energy storage, cellular hypoxia quickly leads to the breakdown of oxidative mitochondrial metabolism and anoxic cell death in the ischemic core. The less-ischemic peripheral zone, the penumbra, is initially viable but will proceed to infarction unless there is timely reperfusion. It is particularly vulnerable to additional hemodynamic and metabolic challenges. Various cascades such as secondary hypoxia due to peri-infarct depolarization may aggravate tissue damage.^1^

So far, translational research has failed to establish brain-protective therapies in acute ischemic stroke, and revascularization of the occluded cerebral arteries by thrombolysis or mechanical thrombectomy (MT) is the only proven effective treatment.^2,3^ Rapid demise of the penumbra, however, explains unfavorable outcomes in a substantial proportion of patients despite successful reperfusion.^3^ To increase the number of patients eligible for revascularization and improve their outcomes, brain-protective “bridging” extending penumbral tissue survival (“freezing the penumbra”) would be desirable.^4,5^

Neuronal energy production depends almost exclusively on oxidative phosphorylation in the mitochondria.^6^ Although the brain represents only 2% of the body’s weight, it consumes roughly 20% of the oxygen available to the whole body.^6,7^ As the critical oxygen tension required for mitochondrial function is very low (1.5 mmHg),^7^ improving oxygen delivery to ischemic-hypoxic tissue appears to be a plausible therapeutic concept to mitigate cell death.^5^

Consequently, the PROOF trial investigates normobaric oxygen (NBO) therapy for brain-protective bridging until revascularization by MT in patients with acute ischemic stroke.

## Method

Study protocol and protocol changes are provided as Supplemental Material.

### Design

Randomized (1:1), standard treatment-controlled, open-label, blinded endpoint, multicenter adaptive phase IIb trial.

### Patient population

Previously functionally independent adults with moderate to severe stroke (NIHSS ⩾ 6) due to acute intracranial anterior-circulation large vessel occlusion (LVO), salvageable penumbra predicted by a high ASPECTS and likelihood for MT.

#### Inclusion criteria

- Age ⩾ 18 years
- Acute terminal internal carotid artery, M1 and/or M2/3 segment(s) occlusion on CT/MR angiography
- Likely MT
- NIHSS ⩾ 6
- ASPECTS 6–10 on non-contrast CT or 5–10 on diffusion-weighted MRI (DWI)
- CT/MR perfusion prior to NBO
- NBO can be initiated within 6 h of symptom onset or notice in case of unknown onset, and within 30 min after baseline brain imaging
- Pre-stroke mRS 0–2
- Breastfeeding women must stop breastfeeding
- Deferred consent or consent by patient/legally authorized representative

#### Key exclusion criteria

- Groin puncture prior to randomization
- Condition which precludes obtaining accurate baseline NIHSS or outcomes
- Intracranial hemorrhage/tumor/arteriovenous malformation
- Intracranial aneurysm or prior stent in the vascular territory of qualifying vessel occlusion
- Suspected complete common carotid artery occlusion/aortic dissection/vasculitis/septic embolism
- Acute/chronic respiratory disease that may interfere with NBO
- Prior to enrolment, ⩾2 L oxygen per minute required to maintain oxygen saturation >94%
- Clinical suspicion of acute myocardial infarction (e.g. chest pain)

### Randomization

Patients were randomized (1:1) using the minimization method with preferred treatment probability of 0.9 via the www.randomizer.at platform with following strata: (1) baseline brain imaging modality, (2) side of LVO, (3) LVO location, (4) baseline NIHSS, (5) time window, and (6) study site.

### Study intervention

**Figure 1.**
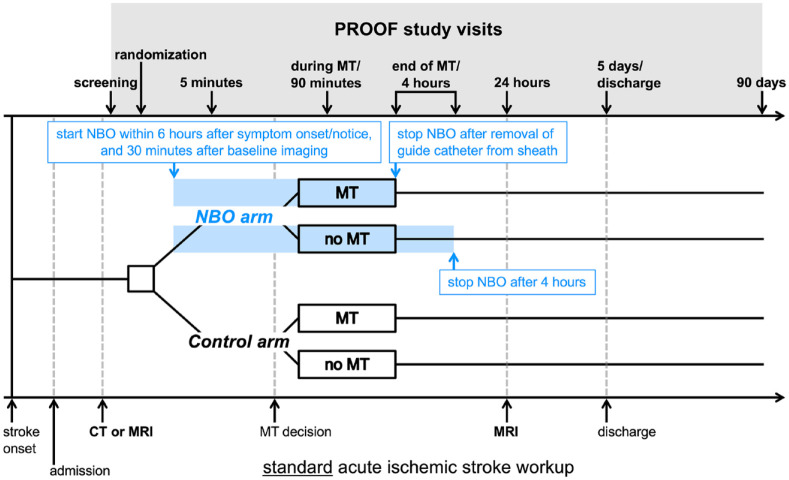
Study flow. MT: mechanical thrombectomy, NBO: normobaric oxygen.

NBO: ⩾40 L oxygen per minute via a non-rebreather face mask with reservoir or, if ventilated, 1.0 inspiratory oxygen fraction until removal of guide catheter from sheath at the end of MT, or for 4 h, if MT was not attempted or stopped prior to manipulation of LVO (Figure 1).

Control treatment: oxygen supplementation if oxygen saturation ⩽94% according to European Stroke Organization guidelines.^8^

### Primary outcome

Primary efficacy of NBO is determined by ischemic core growth in the NBO and Control arms. Ischemic core growth is defined as the change in core volume (mL) from baseline (determined on DWI, CT perfusion, or CT angiography source images) to 24 h (DWI).

### Secondary outcomes

Main secondary outcomes are change in NIHSS from baseline to 24 h and mRS at 90 days. Further secondary efficacy outcomes include arterial oxygen pressure during MT (or at 90 min), relative percent change in ischemic core volume from baseline to 24 h, and Barthel Index, Montreal Cognitive Assessment, Montgomery-Åsberg Depression Rating Scale, Stroke Impact Scale – 16, and EuroQoL Questionnaire 5D-5L at 90 days.

Safety outcomes include vasospasm during MT, intracranial hemorrhage at 24 h, symptomatic intracranial hemorrhage until day 5, and respiratory adverse events.

Exploratory outcomes are imaging and blood biomarkers.

### Blinding

Primary and all other imaging outcomes are assessed by two independent neuroradiologists at imaging core lab Eppdata (Hamburg, Germany) and main secondary outcomes by a local investigator, all blinded to treatment allocation. Conflicts in reading of study imaging are resolved by consensus.

### Data safety monitoring board

Four independent clinical stroke/MT trial experts and one biometrician ensure ethical conduct of the trial, and protect the rights and welfare of participants. Deaths and intracranial hemorrhages were reported without delay to Data Safety Monitoring Board (DSMB). In addition, DSMB received periodic reports of enrollment, drop-outs, adverse events, and—on request—any other trial data. DSMB meetings were planned in 6-month intervals.

### Sample size estimates

We planned adaptive sample size with interim analysis as the effect of NBO on infarct volume is unknown. Sample size calculation was based on absolute core growth of 62 anterior-circulation LVO cases from SWIFT-PRIME who underwent MT and achieved successful reperfusion.^9^ Mean and standard deviation were estimated as 17.8 ± 21.4 mL from the quartiles, assuming normal distribution.^9^ We expected a 50% reduction of core growth (i.e. 8.9 mL) in those 75% of NBO-treated participants who would undergo MT (93.75% of all cases) and achieve successful reperfusion (80% of MT cases^3,9^). Considering the other 25% of failed/not-attempted MT would reduce the mean effect to 6.68 mL. Consequently, 138 participants per arm would be needed for a one-sided test at alpha 0.05 to detect a treatment effect with 80% power. Adaptive design^10^ allowed the trial to be stopped for success (p < 0.0233) or futility (p ⩾ 0.5) after interim analysis (80 patients per arm), or to be continued with additional 11 to 148 patients per arm.

### Statistical analyses

Primary analysis will test the null hypothesis that the NBO-effect adjunct to standard treatment (compared with standard treatment alone) on ischemic core growth is at least 0 mL, at an overall alpha 0.05 using a re-randomization test (with all randomization strata) in all patients randomized in an intention-to-treat analysis. Missing values at 24 h will be imputed. Total infarction of individual penumbra (i.e. worst outcome) will be assumed in case of death. Sensitivity analyses using a linear model with additional explanatory variables such as baseline infarct core volume and strata, as well as subgroup analyses regarding age, time window, NIHSS, ASPECTS, LVO location, thrombolysis, anesthesia strategy, and reperfusion status are planned.

### Study organization

Recruitment was planned in trial sites in Belgium, Czech Republic, Finland, France, Germany, Spain, Sweden, and Switzerland. PROOF is carried out according to Good Clinical Practice E6(R2) guidelines and the current Declaration of Helsinki. The trial sponsor is Medical Faculty of Tübingen University, Germany. The Coordinating Center for Clinical Trials at Heidelberg University, Germany, coordinates and monitors the trial with support from European Clinical Infrastructure Network.

### Study conduct

Patients were recruited between August 17, 2019 and May 13, 2022. Trial results will be published separately.

### Protocol amendments

Until first active version 1.2, regulatory requests of competent authorities were implemented. In version 1.3, the upper age limit of 80 years was removed, M2/3 segment(s) occlusion was permitted, and the therapeutic window was extended from 3 to 6 h. In version 1.4, unknown onset stroke was allowed within 6 h of symptom notice, and ranges of ASPECTS and mRS were broadened by one point.

## Discussion

Four fundamental considerations based on previous experimental and clinical studies showing a benefit of NBO were implemented in the design of PROOF.

First, we aimed for near 100% oxygen delivery during respiratory inspiration. In animal studies, NBO was shown to nonlinearly increase penumbral partial pressure of oxygen, maintaining physiological levels during middle cerebral artery occlusion when inspiratory oxygen fraction was at least 0.95.^11^ Accordingly, in a randomized pilot trial, oxygen at 45 L per minute via a simple face mask led to significant arterial hyperoxygenation, temporary NIHSS improvement, and stabilization of DWI lesions during NBO.^12^ Compared to hyperbaric oxygen therapy, which may provide superior brain protection according to animal studies, NBO is inexpensive, widely available and easy to administer during acute stroke workup including MT.^5^

Second, we aimed to initiate NBO early. Brain tissue oxygen is depleted within seconds after blood flow interruption,^1^ and animal studies have shown no neuroprotection with delayed NBO.^13^ Considering results from extended time window MT trials, the high ASPECTS required for enrollment into PROOF combined with the time window of 6 h after symptom onset/notice would sufficiently well-indicate relevant volumes of potentially salvageable penumbra.^14^

Ethical concerns required brain imaging to exclude hemorrhagic stroke patients who are less likely to benefit from NBO and might even be harmed.^15^ To start NBO as soon as possible after exclusion of hemorrhage and confirmation of qualifying LVO, PROOF settled for a likely but not-yet-decided MT. In addition, evaluation of CT perfusion was not required before enrollment. Consequential inclusion of fast progressors with malignant perfusion profiles may help to differentiate their response to NBO from that of well-collateralized slow progressors.^16^ To accelerate enrollment procedure, we opted for deferred consent in countries where permitted.

Third, as a proof of concept, we focused on MT candidates as our study population. In animal models, NBO only consistently led to infarct volume reduction when ischemia was temporary and lasted up to 3 h.^4^ Given that “nothing can hold its breath forever,” beneficial effects of NBO vanished in case of no recanalization.^12^ MT provides high rates of successful reperfusion and clearly defined ischemia duration and revascularization status.^3,9^

Fourth, we aimed to stop NBO at the end of MT procedure. Few animal studies evaluated post-reperfusion NBO and reported mixed results.^11,17,18^ While Liu et al.^11^ warned that over-oxygenation during reperfusion could potentially lead to free radical toxicity, they also pointed out that hyperoxia seems more tolerable to the brain than hypoxia. This is why we did not stop NBO immediately after successful reperfusion but allowed NBO to also cover treatment of accidental new ischemia in case of thrombus dislocation during clot retrieval.

An early brain imaging primary outcome, heterogenous baseline imaging and intermodal comparison are potential limitations of the trial. Early imaging outcomes are predictive of clinical outcomes at 90 days^19^ and magnetic resonance imaging (MRI) can effectively quantify neuroprotection.^12^ To study the primary outcome, comparison of DWI at 24 h and at baseline would be optimal. However, to not distort the standard workflow, PROOF allowed for multimodal CT at baseline.^20^ Using baseline imaging modality as stratum for randomization mitigates the risk of confounding. To address interindividual ischemia size variability due to LVO location, infarct growth was chosen over infarct volume. Primary outcome assessment at 24 h on follow-up imaging avoids drop-outs due to withdrawal of care and reduces variability due to early clinical complications such as delayed extubation and aspiration pneumonia.

## Summary and conclusion

Available data indicate protective effects of NBO on ischemic but still viable brain tissue if (1) near 100% oxygen is (2) initiated early in (3) short temporary ischemia and (4) stopped soon after reperfusion. PROOF is a proof of concept for this assumption and focuses on a select population mimicking the animal models in which NBO was shown beneficial.

We believe that most clinical studies^15^ failed to show positive or long-lasting NBO-effects in ischemic stroke patients because evidence from successful preclinical application of NBO was not sufficiently considered in the design of these trials. Two recent Chinese single-center studies showed a strong benefit of NBO. One study was similar to PROOF,^21^ the other, however, started NBO only after successful MT.^22^

Considering its low cost, availability, and ease of use, the prospect of NBO to extend the time window for successful reperfusion by “freezing the penumbra” may impact acute stroke care worldwide.

## Supplemental Material

sj-pdf-1-wso-10.1177_17474930231185275 – Supplemental material for Penumbral Rescue by normobaric O = O administration in patients with ischemic stroke and target mismatch proFile (PROOF): Study protocol of a phase IIb trial Click here for additional data file. Supplemental material, sj-pdf-1-wso-10.1177_17474930231185275 for Penumbral Rescue by normobaric O = O administration in patients with ischemic stroke and target mismatch proFile (PROOF): Study protocol of a phase IIb trial by Sven Poli, Joshua Mbroh, Jean-Claude Baron, Aneesh B Singhal, Daniel Strbian, Carlos Molina, Robin Lemmens, Guillaume Turc, Robert Mikulik, Patrik Michel, Turgut Tatlisumak, Heinrich J Audebert, Martin Dichgans, Roland Veltkamp, Johannes Hüsing, Holm Graessner, Jens Fiehler, Joan Montaner, Adedolapo Kamaldeen Adeyemi, Katharina Althaus, Juan F Arenillas, Benjamin Bender, Frank Benedikt, Gabriel Broocks, Ina Burghaus, Pere Cardona, Milani Deb-Chatterji, Martina Cviková, Luc Defreyne, Veerle De Herdt, Olivier Detante, Ulrike Ernemann, Fabian Flottmann, Lídia García Guillamón, Monika Glauch, Alexandra Gomez-Exposito, Benjamin Gory, Sylvie Sylvie Grand, Michal Haršány, Till Karsten Hauser, Olivier Heck, Dimitri Hemelsoet, Florian Hennersdorf, Julia Hoppe, Pia Kalmbach, Lars Kellert, Martin Köhrmann, Markus Kowarik, Blanca Lara-Rodríguez, Loic Legris, Tobias Lindig, Steffen Luntz, Jay Lusk, Brian Mac Grory, Andreas Manger, Nicolas Martinez-Majander, Annerose Mengel, Johannes Meyne, Susanne Müller, Sibu Mundiyanapurath, Olivier Naggara, Krassen Nedeltchev, Thanh N Nguyen, Maike A Nilsson, Michael Obadia, Khouloud Poli, Jan C Purrucker, Silja Räty, Sebastien Richard, Hardy Richter, Clotilde Schilte, Eckhard Schlemm, Linda Stöhr, Benjamin Stolte, Marek Sykora, Götz Thomalla, Liisa Tomppo, Noel van Horn, Julia Zeller, Ulf Ziemann, Christine S Zuern, Florian Härtig and Johannes Tuennerhoff in International Journal of Stroke

sj-pdf-2-wso-10.1177_17474930231185275 – Supplemental material for Penumbral Rescue by normobaric O = O administration in patients with ischemic stroke and target mismatch proFile (PROOF): Study protocol of a phase IIb trial Click here for additional data file. Supplemental material, sj-pdf-2-wso-10.1177_17474930231185275 for Penumbral Rescue by normobaric O = O administration in patients with ischemic stroke and target mismatch proFile (PROOF): Study protocol of a phase IIb trial by Sven Poli, Joshua Mbroh, Jean-Claude Baron, Aneesh B Singhal, Daniel Strbian, Carlos Molina, Robin Lemmens, Guillaume Turc, Robert Mikulik, Patrik Michel, Turgut Tatlisumak, Heinrich J Audebert, Martin Dichgans, Roland Veltkamp, Johannes Hüsing, Holm Graessner, Jens Fiehler, Joan Montaner, Adedolapo Kamaldeen Adeyemi, Katharina Althaus, Juan F Arenillas, Benjamin Bender, Frank Benedikt, Gabriel Broocks, Ina Burghaus, Pere Cardona, Milani Deb-Chatterji, Martina Cviková, Luc Defreyne, Veerle De Herdt, Olivier Detante, Ulrike Ernemann, Fabian Flottmann, Lídia García Guillamón, Monika Glauch, Alexandra Gomez-Exposito, Benjamin Gory, Sylvie Sylvie Grand, Michal Haršány, Till Karsten Hauser, Olivier Heck, Dimitri Hemelsoet, Florian Hennersdorf, Julia Hoppe, Pia Kalmbach, Lars Kellert, Martin Köhrmann, Markus Kowarik, Blanca Lara-Rodríguez, Loic Legris, Tobias Lindig, Steffen Luntz, Jay Lusk, Brian Mac Grory, Andreas Manger, Nicolas Martinez-Majander, Annerose Mengel, Johannes Meyne, Susanne Müller, Sibu Mundiyanapurath, Olivier Naggara, Krassen Nedeltchev, Thanh N Nguyen, Maike A Nilsson, Michael Obadia, Khouloud Poli, Jan C Purrucker, Silja Räty, Sebastien Richard, Hardy Richter, Clotilde Schilte, Eckhard Schlemm, Linda Stöhr, Benjamin Stolte, Marek Sykora, Götz Thomalla, Liisa Tomppo, Noel van Horn, Julia Zeller, Ulf Ziemann, Christine S Zuern, Florian Härtig and Johannes Tuennerhoff in International Journal of Stroke

sj-pdf-3-wso-10.1177_17474930231185275 – Supplemental material for Penumbral Rescue by normobaric O = O administration in patients with ischemic stroke and target mismatch proFile (PROOF): Study protocol of a phase IIb trial Click here for additional data file. Supplemental material, sj-pdf-3-wso-10.1177_17474930231185275 for Penumbral Rescue by normobaric O = O administration in patients with ischemic stroke and target mismatch proFile (PROOF): Study protocol of a phase IIb trial by Sven Poli, Joshua Mbroh, Jean-Claude Baron, Aneesh B Singhal, Daniel Strbian, Carlos Molina, Robin Lemmens, Guillaume Turc, Robert Mikulik, Patrik Michel, Turgut Tatlisumak, Heinrich J Audebert, Martin Dichgans, Roland Veltkamp, Johannes Hüsing, Holm Graessner, Jens Fiehler, Joan Montaner, Adedolapo Kamaldeen Adeyemi, Katharina Althaus, Juan F Arenillas, Benjamin Bender, Frank Benedikt, Gabriel Broocks, Ina Burghaus, Pere Cardona, Milani Deb-Chatterji, Martina Cviková, Luc Defreyne, Veerle De Herdt, Olivier Detante, Ulrike Ernemann, Fabian Flottmann, Lídia García Guillamón, Monika Glauch, Alexandra Gomez-Exposito, Benjamin Gory, Sylvie Sylvie Grand, Michal Haršány, Till Karsten Hauser, Olivier Heck, Dimitri Hemelsoet, Florian Hennersdorf, Julia Hoppe, Pia Kalmbach, Lars Kellert, Martin Köhrmann, Markus Kowarik, Blanca Lara-Rodríguez, Loic Legris, Tobias Lindig, Steffen Luntz, Jay Lusk, Brian Mac Grory, Andreas Manger, Nicolas Martinez-Majander, Annerose Mengel, Johannes Meyne, Susanne Müller, Sibu Mundiyanapurath, Olivier Naggara, Krassen Nedeltchev, Thanh N Nguyen, Maike A Nilsson, Michael Obadia, Khouloud Poli, Jan C Purrucker, Silja Räty, Sebastien Richard, Hardy Richter, Clotilde Schilte, Eckhard Schlemm, Linda Stöhr, Benjamin Stolte, Marek Sykora, Götz Thomalla, Liisa Tomppo, Noel van Horn, Julia Zeller, Ulf Ziemann, Christine S Zuern, Florian Härtig and Johannes Tuennerhoff in International Journal of Stroke

sj-pdf-4-wso-10.1177_17474930231185275 – Supplemental material for Penumbral Rescue by normobaric O = O administration in patients with ischemic stroke and target mismatch proFile (PROOF): Study protocol of a phase IIb trial Click here for additional data file. Supplemental material, sj-pdf-4-wso-10.1177_17474930231185275 for Penumbral Rescue by normobaric O = O administration in patients with ischemic stroke and target mismatch proFile (PROOF): Study protocol of a phase IIb trial by Sven Poli, Joshua Mbroh, Jean-Claude Baron, Aneesh B Singhal, Daniel Strbian, Carlos Molina, Robin Lemmens, Guillaume Turc, Robert Mikulik, Patrik Michel, Turgut Tatlisumak, Heinrich J Audebert, Martin Dichgans, Roland Veltkamp, Johannes Hüsing, Holm Graessner, Jens Fiehler, Joan Montaner, Adedolapo Kamaldeen Adeyemi, Katharina Althaus, Juan F Arenillas, Benjamin Bender, Frank Benedikt, Gabriel Broocks, Ina Burghaus, Pere Cardona, Milani Deb-Chatterji, Martina Cviková, Luc Defreyne, Veerle De Herdt, Olivier Detante, Ulrike Ernemann, Fabian Flottmann, Lídia García Guillamón, Monika Glauch, Alexandra Gomez-Exposito, Benjamin Gory, Sylvie Sylvie Grand, Michal Haršány, Till Karsten Hauser, Olivier Heck, Dimitri Hemelsoet, Florian Hennersdorf, Julia Hoppe, Pia Kalmbach, Lars Kellert, Martin Köhrmann, Markus Kowarik, Blanca Lara-Rodríguez, Loic Legris, Tobias Lindig, Steffen Luntz, Jay Lusk, Brian Mac Grory, Andreas Manger, Nicolas Martinez-Majander, Annerose Mengel, Johannes Meyne, Susanne Müller, Sibu Mundiyanapurath, Olivier Naggara, Krassen Nedeltchev, Thanh N Nguyen, Maike A Nilsson, Michael Obadia, Khouloud Poli, Jan C Purrucker, Silja Räty, Sebastien Richard, Hardy Richter, Clotilde Schilte, Eckhard Schlemm, Linda Stöhr, Benjamin Stolte, Marek Sykora, Götz Thomalla, Liisa Tomppo, Noel van Horn, Julia Zeller, Ulf Ziemann, Christine S Zuern, Florian Härtig and Johannes Tuennerhoff in International Journal of Stroke

sj-pdf-5-wso-10.1177_17474930231185275 – Supplemental material for Penumbral Rescue by normobaric O = O administration in patients with ischemic stroke and target mismatch proFile (PROOF): Study protocol of a phase IIb trial Click here for additional data file. Supplemental material, sj-pdf-5-wso-10.1177_17474930231185275 for Penumbral Rescue by normobaric O = O administration in patients with ischemic stroke and target mismatch proFile (PROOF): Study protocol of a phase IIb trial by Sven Poli, Joshua Mbroh, Jean-Claude Baron, Aneesh B Singhal, Daniel Strbian, Carlos Molina, Robin Lemmens, Guillaume Turc, Robert Mikulik, Patrik Michel, Turgut Tatlisumak, Heinrich J Audebert, Martin Dichgans, Roland Veltkamp, Johannes Hüsing, Holm Graessner, Jens Fiehler, Joan Montaner, Adedolapo Kamaldeen Adeyemi, Katharina Althaus, Juan F Arenillas, Benjamin Bender, Frank Benedikt, Gabriel Broocks, Ina Burghaus, Pere Cardona, Milani Deb-Chatterji, Martina Cviková, Luc Defreyne, Veerle De Herdt, Olivier Detante, Ulrike Ernemann, Fabian Flottmann, Lídia García Guillamón, Monika Glauch, Alexandra Gomez-Exposito, Benjamin Gory, Sylvie Sylvie Grand, Michal Haršány, Till Karsten Hauser, Olivier Heck, Dimitri Hemelsoet, Florian Hennersdorf, Julia Hoppe, Pia Kalmbach, Lars Kellert, Martin Köhrmann, Markus Kowarik, Blanca Lara-Rodríguez, Loic Legris, Tobias Lindig, Steffen Luntz, Jay Lusk, Brian Mac Grory, Andreas Manger, Nicolas Martinez-Majander, Annerose Mengel, Johannes Meyne, Susanne Müller, Sibu Mundiyanapurath, Olivier Naggara, Krassen Nedeltchev, Thanh N Nguyen, Maike A Nilsson, Michael Obadia, Khouloud Poli, Jan C Purrucker, Silja Räty, Sebastien Richard, Hardy Richter, Clotilde Schilte, Eckhard Schlemm, Linda Stöhr, Benjamin Stolte, Marek Sykora, Götz Thomalla, Liisa Tomppo, Noel van Horn, Julia Zeller, Ulf Ziemann, Christine S Zuern, Florian Härtig and Johannes Tuennerhoff in International Journal of Stroke

sj-pdf-6-wso-10.1177_17474930231185275 – Supplemental material for Penumbral Rescue by normobaric O = O administration in patients with ischemic stroke and target mismatch proFile (PROOF): Study protocol of a phase IIb trial Click here for additional data file. Supplemental material, sj-pdf-6-wso-10.1177_17474930231185275 for Penumbral Rescue by normobaric O = O administration in patients with ischemic stroke and target mismatch proFile (PROOF): Study protocol of a phase IIb trial by Sven Poli, Joshua Mbroh, Jean-Claude Baron, Aneesh B Singhal, Daniel Strbian, Carlos Molina, Robin Lemmens, Guillaume Turc, Robert Mikulik, Patrik Michel, Turgut Tatlisumak, Heinrich J Audebert, Martin Dichgans, Roland Veltkamp, Johannes Hüsing, Holm Graessner, Jens Fiehler, Joan Montaner, Adedolapo Kamaldeen Adeyemi, Katharina Althaus, Juan F Arenillas, Benjamin Bender, Frank Benedikt, Gabriel Broocks, Ina Burghaus, Pere Cardona, Milani Deb-Chatterji, Martina Cviková, Luc Defreyne, Veerle De Herdt, Olivier Detante, Ulrike Ernemann, Fabian Flottmann, Lídia García Guillamón, Monika Glauch, Alexandra Gomez-Exposito, Benjamin Gory, Sylvie Sylvie Grand, Michal Haršány, Till Karsten Hauser, Olivier Heck, Dimitri Hemelsoet, Florian Hennersdorf, Julia Hoppe, Pia Kalmbach, Lars Kellert, Martin Köhrmann, Markus Kowarik, Blanca Lara-Rodríguez, Loic Legris, Tobias Lindig, Steffen Luntz, Jay Lusk, Brian Mac Grory, Andreas Manger, Nicolas Martinez-Majander, Annerose Mengel, Johannes Meyne, Susanne Müller, Sibu Mundiyanapurath, Olivier Naggara, Krassen Nedeltchev, Thanh N Nguyen, Maike A Nilsson, Michael Obadia, Khouloud Poli, Jan C Purrucker, Silja Räty, Sebastien Richard, Hardy Richter, Clotilde Schilte, Eckhard Schlemm, Linda Stöhr, Benjamin Stolte, Marek Sykora, Götz Thomalla, Liisa Tomppo, Noel van Horn, Julia Zeller, Ulf Ziemann, Christine S Zuern, Florian Härtig and Johannes Tuennerhoff in International Journal of Stroke

sj-pdf-7-wso-10.1177_17474930231185275 – Supplemental material for Penumbral Rescue by normobaric O = O administration in patients with ischemic stroke and target mismatch proFile (PROOF): Study protocol of a phase IIb trial Click here for additional data file. Supplemental material, sj-pdf-7-wso-10.1177_17474930231185275 for Penumbral Rescue by normobaric O = O administration in patients with ischemic stroke and target mismatch proFile (PROOF): Study protocol of a phase IIb trial by Sven Poli, Joshua Mbroh, Jean-Claude Baron, Aneesh B Singhal, Daniel Strbian, Carlos Molina, Robin Lemmens, Guillaume Turc, Robert Mikulik, Patrik Michel, Turgut Tatlisumak, Heinrich J Audebert, Martin Dichgans, Roland Veltkamp, Johannes Hüsing, Holm Graessner, Jens Fiehler, Joan Montaner, Adedolapo Kamaldeen Adeyemi, Katharina Althaus, Juan F Arenillas, Benjamin Bender, Frank Benedikt, Gabriel Broocks, Ina Burghaus, Pere Cardona, Milani Deb-Chatterji, Martina Cviková, Luc Defreyne, Veerle De Herdt, Olivier Detante, Ulrike Ernemann, Fabian Flottmann, Lídia García Guillamón, Monika Glauch, Alexandra Gomez-Exposito, Benjamin Gory, Sylvie Sylvie Grand, Michal Haršány, Till Karsten Hauser, Olivier Heck, Dimitri Hemelsoet, Florian Hennersdorf, Julia Hoppe, Pia Kalmbach, Lars Kellert, Martin Köhrmann, Markus Kowarik, Blanca Lara-Rodríguez, Loic Legris, Tobias Lindig, Steffen Luntz, Jay Lusk, Brian Mac Grory, Andreas Manger, Nicolas Martinez-Majander, Annerose Mengel, Johannes Meyne, Susanne Müller, Sibu Mundiyanapurath, Olivier Naggara, Krassen Nedeltchev, Thanh N Nguyen, Maike A Nilsson, Michael Obadia, Khouloud Poli, Jan C Purrucker, Silja Räty, Sebastien Richard, Hardy Richter, Clotilde Schilte, Eckhard Schlemm, Linda Stöhr, Benjamin Stolte, Marek Sykora, Götz Thomalla, Liisa Tomppo, Noel van Horn, Julia Zeller, Ulf Ziemann, Christine S Zuern, Florian Härtig and Johannes Tuennerhoff in International Journal of Stroke
