# Supplementary material for: Penumbral Rescue by normobaric O = O administration in patients with ischemic stroke and target mismatch proFile (PROOF): Study protocol of a phase IIb trial
Source: Int J Stroke. 2023 Aug 18;19(1):120–6. doi: 10.1177/17474930231185275 (PMC10759237; doi:10.1177/17474930231185275)
Supplement: sj-pdf-1-wso-10.1177_17474930231185275 – Supplemental material for Penumbral Rescue by normobaric O = O administration in patients with ischemic stroke and target mismatch proFile (PROOF): Study protocol of a phase IIb trial [file sj-pdf-1-wso-10.1177_17474930231185275.pdf]

Appendix 1 Description of the substantial amendment

Clinical Study Protocol "PROOF: Penumbral Rescue by Normobaric O=O Administration in Patients with Ischemic Stroke and Target Mismatch ProFile: A Phase II Proof-of-Concept Trial"
Phase of study: Phase II – proof-of-concept
EudraCT No.: 2017-001355-31
Study Registry Number: NCT03500939

The respective changes made to the Protocol Version 1.0/ 10.04.2018 to Version 1.1/ 30.05.2018 are tabulated below.

Previous and new wording in track change modus	New wording	Comments/ reasons for substantial amendment
**1 Protocol Synopsis Objectives (page 12)**		
Exploratory objectives ~~to be defined in imaging and biomarker protocol~~:<br>• Exploratory analyses of imaging (to be defined in imaging protocol and Interpretation Guidelines).<br>• Exploratory analyses of biochemical biomarkers to develop a blood-based test to monitor efficacy and safety of NBHO treatment~~.~~	Exploratory objectives:<br>• Exploratory analyses of imaging (to be defined in imaging protocol and Interpretation Guidelines).<br>• Exploratory analyses of biochemical biomarkers to develop a blood-based test to monitor efficacy and safety of NBHO treatment	
**1 Protocol Synopsis Study Population (page 12)**		
Inclusion Criteria<br>• Age: ~~male patients:~~ 18 to 80 years~~, female patients: 50 to 80 years~~	Inclusion Criteria<br>• Age: 18 to 80 years	
**1 Protocol Synopsis Study Population (page 13)**		
Respiratory:<br>• Known history of chronic pulmonary disease (e.g. COPD, pulmonary fibrosis, alveolitis or pneumonitis)<br>• ~~Or a~~Any condition leading to hypoxic respiratory drive (e.g. neuromuscular disease)<br>• Prior to enrolment, > 2 L/min oxygen required to maintain peripheral oxygen saturation ≥ 95%<br>• ~~Or a~~Acute respiratory distress that may, in the clinical judgment of the investigator, interfere with	Respiratory:<br>• Known history of chronic pulmonary disease (e.g. COPD, pulmonary fibrosis, alveolitis or pneumonitis)<br>• Any condition leading to hypoxic respiratory drive (e.g. neuromuscular disease)<br>• Prior to enrolment, > 2 L/min oxygen required to maintain peripheral oxygen saturation ≥ 95%<br>• Acute respiratory distress that may, in the clinical judgment of the investigator, interfere with the study intervention	

Previous and new wording in track change modus	New wording	Comments/ reasons for substantial amendment
the study intervention<br>• Acute viral, bacterial or fungal pneumonia	• Acute viral, bacterial or fungal pneumonia	
**1 Protocol Synopsis Study Population (page 14)**		
Other:<br>• Pregnancy at screening, to be excluded (β-HCG in serum or urine) in all women ≤ 55 years except if surgically sterile; in women >55 years pregnancy must be excluded only in case of increased probability e.g. due to in-vitro fertilization Women of childbearing age, i.e.<50 years as defined by World Health Organization<br>• Any pre-existing condition that may, in the clinical judgment of the investigator, not allow safe participation in the study (e.g. alcohol or substance abuse, co-existing disease) or would complicate assessment of outcomes (e.g. dementia, psychiatric disease) or would confound the neurological or functional evaluations (e.g. dementia)	Other:<br>• Pregnancy at screening, to be excluded (β-HCG in serum or urine) in all women ≤ 55 years except if surgically sterile; in women >55 years pregnancy must be excluded only in case of increased probability e.g. due to in-vitro fertilization<br>• Any pre-existing condition that may, in the clinical judgment of the investigator, not allow safe participation in the study (e.g. alcohol or substance abuse, co-existing disease) or would complicate assessment of outcomes (e.g. dementia, psychiatric disease) or would confound the neurological or functional evaluations (e.g. dementia)	
**1 Protocol Synopsis SUBTRIALS (page 15)**		
• Biomarkers (WB6); see Biomarker Study Protocol Section 10.19	• Biomarkers (WP6); see Section **Fehler! Verweisquelle konnte nicht gefunden werden.**	

Previous and new wording in track change modus	New wording	Comments/ reasons for substantial amendment
**2 Trial Schedule (page 17)**		
23. Compare Section **Fehler! Verweisquelle konnte nicht gefunden werden.** and Biomarker Study Protocol sample handling instructions<br>24. Blood for biomarkers should be drawn only from patients with study-independent venous or arterial access. It has to be assured, that the samples for biomarker assessment are not shipped to the central lab before the patient or his/her LAR gives consent. In case the patient / LAR does not consent, samples have to be discarded.<br>24.25. Urine or serum β-HCG, in women≤ 55 years except if surgically sterile; in women >55 years pregnancy must be excluded only in case of increased probability e.g. due to in-vitro fertilization	23. Compare Section **Fehler! Verweisquelle konnte nicht gefunden werden.** and Biomarker sample handling instructions<br>24. Blood for biomarkers should be drawn only from patients with study-independent venous or arterial access. It has to be assured, that the samples for biomarker assessment are not shipped to the central lab before the patient or his/her LAR gives consent. In case the patient / LAR does not consent, samples have to be discarded.<br>25. Urine or serum β-HCG, in women ≤ 55 years except if surgically sterile; in women >55 years pregnancy must be excluded only in case of increased probability e.g. due to in-vitro fertilization	
**4 Introduction (page 20)**		
4.1 Scientific Background	4.1 Scientific Backround	
**4 Introduction (page 23)**		
4.14.2 Trial Ratioale/ Justification	4.2 Trial Ratioale/ Justification	
**4 Introduction (page 24)**		
4.24.3 Risk-benefit Assessment<br><br>They are summarized in Table 1Table 1.<br><br>The total amount of blood drawn solely for study purposes amounts to 90.5 mL in case the patient consents to participation in the biomarker sub-study or to 54.5 mL if participation in the biomarker sub-study is refused prior to V5 or 72.5 mL if participation in the biomarker sub-study is refused prior to V6 (see Sections **Fehler! Verweisquelle konnte nicht gefunden werden.** Laboratory assessment, **Fehler! Verweisquelle konnte nicht gefunden werden.** Biomarkers (Pharmacodynamics), and **Fehler! Verweisquelle konnte nicht gefunden werden.** Arterial	4.3 Risk-benefit Assessment<br><br>They are summarized in **Fehler! Verweisquelle konnte nicht gefunden werden.**.<br><br>The total amount of blood drawn solely for study purposes amounts to 90.5 mL in case the patient consents to participation in the biomarker sub-study or to 54.5 mL if participation in the biomarker sub-study is refused prior to V5 or 72.5 mL if participation in the biomarker sub-study is refused prior to V6 (see Sections **Fehler! Verweisquelle konnte nicht gefunden werden.** Laboratory assessment, **Fehler! Verweisquelle konnte nicht gefunden werden.** Biomarkers (Pharmacodynamics), and **Fehler!**	

**Previous and new wording in track change modus**	**New wording**	**Comments/ reasons for substantial amendment**
blood gases (Pharmacokinetics) as well as Table 4 ~~**Fehler! Verweisquelle konnte nicht gefunden werden.**~~ for details).	**Verweisquelle konnte nicht gefunden werden.** Arterial blood gases (Pharmacokinetics) as well as **Fehler! Verweisquelle konnte nicht gefunden werden.** for details).	
**Prevention, early detection and management of adverse events in the PROOF-trial (page 28)**		
Prevention: In the PROOF-trial, NBHO treatment is limited to a maximum of four hours (which is the longest planned exposure to 100% oxygen inhalation). This time frame was chosen in order to maintain the highest possible safety with regard to potential oxygen toxicity. Thus, only mild and fully reversible adverse effects of oxygen administration are to be expected in the PROOF trial.<br>Patients with a history of COPD and other pulmonary disorders are not to be enrolled in this trial (see Section **Fehler! Verweisquelle konnte nicht gefunden werden.** Exclusion Criteria). Use of concomitant medications which may increase the risk of adverse events are prohibited (see Section **Fehler! Verweisquelle konnte nicht gefunden werden.** Prior and Concomitant Medication). To avoid harm for unborn children,pregnancy must be excluded in all women ≤55 years (except if surgically sterile) and in women >55 years in case of increased probability for pregnancy (e.g. due to in-vitro fertilization). ~~In childbearing age are excluded from trial participation (see Section 7.5 Exclusion Criteria); risk for bias is minor as women under the age of 50 years represent only~~	Prevention: In the PROOF-trial, NBHO treatment is limited to a maximum of four hours (which is the longest planned exposure to 100% oxygen inhalation). This time frame was chosen in order to maintain the highest possible safety with regard to potential oxygen toxicity. Thus, only mild and fully reversible adverse effects of oxygen administration are to be expected in the PROOF trial.<br>Patients with a history of COPD and other pulmonary disorders are not to be enrolled in this trial (see Section **Fehler! Verweisquelle konnte nicht gefunden werden.** Exclusion Criteria). Use of concomitant medications which may increase the risk of adverse events are prohibited (see Section **Fehler! Verweisquelle konnte nicht gefunden werden.** Prior and Concomitant Medication). To avoid harm for unborn children, pregnancy must be excluded in all women ≤55 years (except if surgically sterile) and in women >55 years in case of increased probability for pregnancy (e.g. due to in-vitro fertilization).	
**4 Introduction (page 29)**		
~~4.3~~4.4 Data and Safety Monitoring Board (DSMB)	4.4 Data and Safety Monitoring Board (DSMB)	
~~4.4~~4.5 Steering Committee (SC)	4.5 Steering Committee (SC)	
**6.2 Trial Duration and Schedule (page 32)**		

Previous and new wording in track change modus	New wording	Comments/ reasons for substantial amendment
The study end is defined as “last subject~~ patient~~ out” (~~LPO~~ LSO)	. The study end is defined as “last subject out” (LSO).	
**7.4 Inclusion Criteria (page 33)**		
Subjects meeting all of the following criteria will be considered for admission to the trial.<br>• Age: ~~male patients:~~ 18 to 80 years~~,female patients: 50 to 80 years~~	Subjects meeting all of the following criteria will be considered for admission to the trial.<br>• Age: 18 to 80 years	
**7.5 Exclusion Criteria (page 34)**		
Respiratory:<br>• Known history of chronic pulmonary disease (e.g. COPD, pulmonary fibrosis, alveolitis or pneumonitis)<br>• ~~Or a~~Any condition leading to hypoxic respiratory drive (e.g. neuromuscular disease)<br>• Prior to enrolment, > 2 L/min oxygen required to maintain peripheral oxygen saturation ≥ 95%<br>• ~~Or a~~Acute respiratory distress that may, in the clinical judgment of the investigator, interfere with the study intervention<br>• Acute viral, bacterial or fungal pneumonia	Respiratory:<br>• Known history of chronic pulmonary disease (e.g. COPD, pulmonary fibrosis, alveolitis or pneumonitis)<br>• Any condition leading to hypoxic respiratory drive (e.g. neuromuscular disease)<br>• Prior to enrolment, > 2 L/min oxygen required to maintain peripheral oxygen saturation ≥ 95%<br>• Acute respiratory distress that may, in the clinical judgment of the investigator, interfere with the study intervention<br>• Acute viral, bacterial or fungal pneumonia	
Other:<br>• Pregnancy at screening, to be excluded (β-HCG in serum or urine) in all women ≤ 55 years except if surgically sterile; in women >55 years pregnancy must be excluded only in case of increased probability e.g. due to in-vitro fertilization~~Women of childbearing age, i.e.<50 years as defined by World Health Organization (http://www.who.int/reproductivehealth/topics/infertility/defintions/en/)~~	Other:<br>• Pregnancy at screening, to be excluded (β-HCG in serum or urine) in all women ≤ 55 years except if surgically sterile; in women >55 years pregnancy must be excluded only in case of increased probability e.g. due to in-vitro fertilization	

Previous and new wording in track change modus	New wording	Comments/ reasons for substantial amendment
• Any pre-existing condition that may, in the clinical judgment of the investigator, not allow safe participation in the study (e.g. alcohol or substance abuse, co-existing disease) or would complicate assessment of outcomes (e.g. dementia, psychiatric disease) or would confound the neurological or functional evaluations (e.g. dementia)	• Any pre-existing condition that may, in the clinical judgment of the investigator, not allow safe participation in the study (e.g. alcohol or substance abuse, co-existing disease) or would complicate assessment of outcomes (e.g. dementia, psychiatric disease) or would confound the neurological or functional evaluations (e.g. dementia)	
**7.6 Pre-specified Measures in Case of Slow Recuitment (page 34)**		
To ensure enrolment in case of slow recruitment, the following inclusion criteria may be adapted: (1) upper age limit may be increased to 85 years or skipped, (2) pre-stroke mRS may include 0-2, and (3) ASPECTS may opened for 6-10 on non-contrast CT or 5-10 on DWI. The decision for adaption will be reviewed by the IEAB and the SAB, and suggested to the General Assembly by the Steering Committee. In case of approval by the General Assembly, the protocol will be amended accordingly and submitted to the Ethics Committees and Competent Authorities for approval (see Section **Fehler! Verweisquelle konnte nicht gefunden werden.**). In order to facilitate sensible protocol amendments, a pre-screening log will be maintained by the participating study centers (compare Section **Fehler! Verweisquelle konnte nicht gefunden werden.** Pre-Screening).	To ensure enrolment in case of slow recruitment, the following inclusion criteria may be adapted: (1) upper age limit may be increased to 85 years or skipped, (2) pre-stroke mRS may include 0-2, and (3) ASPECTS may opened for 6-10 on non-contrast CT or 5-10 on DWI. The decision for adaption will be reviewed by the IEAB and the SAB, and suggested to the General Assembly by the Steering Committee. In case of approval by the General Assembly, the protocol will be amended accordingly and submitted to the Ethics Committees and Competent Authorities for approval (see Section **Fehler! Verweisquelle konnte nicht gefunden werden.**). In order to facilitate sensible protocol amendments, a pre-screening log will be maintained by the participating study centers (compare Section **Fehler! Verweisquelle konnte nicht gefunden werden.** Pre-Screening).	
**9.2 Screening Visit (page 42)**		
For safety reasons, vital signs (incl. systolic and diastolic blood pressure, heart rate and respiratory rate, SpO_2, and tympanic temperature), blood samples incl. full blood count (white blood cells, platelet count, erythrocytes, hemoglobin, hematocrit), coagulation (international normalized ratio	For safety reasons, vital signs (incl. systolic and diastolic blood pressure, heart rate and respiratory rate, SpO_2, and tympanic temperature), blood samples incl. full blood count (white blood cells, platelet count, erythrocytes, hemoglobin, hematocrit), coagulation (international	

Previous and new wording in track change modus	New wording	Comments/ reasons for substantial amendment
(INR), activated partial thromboplastin time (aPTT), D-dimers), and blood chemistry (sodium, potassium, creatinine, urea, uric acid, total bilirubin, direct bilirubin, total protein, albumin, C-reactive protein, troponin I or T, brain natriuretic peptide (BNP) or N-terminal prohormone of brain natriuretic peptide (NT-proBNP), creatine kinase (CK), aspartate transaminase (AST), alanine transaminase (ALT), alkaline phosphatase, lactate dehydrogenase (LDH), gamma-glutamyl transpeptidase (GGT), thyroid-stimulating hormone (TSH), and glucose), and – if applicable (see Section **Fehler! Verweisquelle konnte nicht gefunden werden.** Exclusion criteria) – serum/urine pregnancy test must be assessed ~~from a routinely drawn blood/urine sample~~.<br>At screening, a first batch of blood samples for biomarker determination (i.e. two gel serum separation Vacutest® tubes of 5 mL (Yellow cap) and two plasma EDTA K2 Vacutest® tubes of 4 mL (Lavender caps)) is taken (as part of the PROOF biomarker sub-study, see Section **Fehler! Verweisquelle konnte nicht gefunden werden.**; total blood amount: 18 mL). Blood for biomarkers should be drawn only from patients with study-independent venous or arterial access. In case, the patient / LAR does not give informed consent (see Section **Fehler! Verweisquelle konnte nicht gefunden werden.** Subject Information and Informed Consent for details), blood samples for biomarker assessment will be discarded.	normalized ratio (INR), activated partial thromboplastin time (aPTT), D-dimers), and blood chemistry (sodium, potassium, creatinine, urea, uric acid, total bilirubin, direct bilirubin, total protein, albumin, C-reactive protein, troponin I or T, brain natriuretic peptide (BNP) or N-terminal prohormone of brain natriuretic peptide (NT-proBNP), creatine kinase (CK), aspartate transaminase (AST), alanine transaminase (ALT), alkaline phosphatase, lactate dehydrogenase (LDH), gamma-glutamyl transpeptidase (GGT), thyroid-stimulating hormone (TSH), and glucose), and – if applicable (see Section **Fehler! Verweisquelle konnte nicht gefunden werden.** Exclusion criteria) – serum/urine pregnancy test must be assessed.<br><br>At screening, a first batch of blood samples for biomarker determination (i.e. two gel serum separation Vacutest® tubes of 5 mL (Yellow cap) and two plasma EDTA K2 Vacutest® tubes of 4 mL (Lavender caps)) is taken (as part of the PROOF biomarker sub-study, see Section **Fehler! Verweisquelle konnte nicht gefunden werden.**; total blood amount: 18 mL). Blood for biomarkers should be drawn only from patients with study-independent venous or arterial access. In case, the patient / LAR does not give informed consent (see Section **Fehler! Verweisquelle konnte nicht gefunden werden.** Subject Information and Informed Consent for details), blood samples for biomarker assessment will be discarded.	
**9.6 V3 – During TBY (page 43)**		
A second batch of blood samples for biomarker determination (i.e. two gel serum separation Vacutest® tubes of 5 mL (Yellow cap) and two plasma EDTA K2 Vacutest® tubes of 4 mL (Lavender caps)) is taken (as part of the PROOF biomarker sub-study; see Section **Fehler! Verweisquelle konnte nicht gefunden werden.**; total	A second batch of blood samples for biomarker determination (i.e. two gel serum separation Vacutest® tubes of 5 mL (Yellow cap) and two plasma EDTA K2 Vacutest® tubes of 4 mL (Lavender caps)) is taken (as part of the PROOF biomarker sub-study; see Section **Fehler! Verweisquelle konnte nicht gefunden werden.**;	

Previous and new wording in track change modus	New wording	Comments/ reasons for substantial amendment
blood amount: 18 mL). Blood for biomarkers should be drawn only from patients with study-independent venous or arterial access.<br>Vital signs (incl. systolic and diastolic blood pressure, heart rate and respiratory rate, and SpO_2) as well as reason, flow-rate and method of O_2 supplementation are recorded as indicated in Section **Fehler! Verweisquelle konnte nicht gefunden werden.** Vital signs.	total blood amount: 18 mL). Blood for biomarkers should be drawn only from patients with study-independent venous or arterial access.<br>Vital signs (incl. systolic and diastolic blood pressure, heart rate and respiratory rate, and SpO_2) as well as reason, flow-rate and method of O_2 supplementation are recorded as indicated in Section **Fehler! Verweisquelle konnte nicht gefunden werden.** Vital signs.	
**9.8 V5 - 24±6 hours (Day 1) after start of NBHO (or randomization in the control arm) (page 44)**		
A third batch of blood samples for biomarker determination (i.e. two gel serum separation Vacutest® tubes of 5 mL (Yellow cap) and two plasma EDTA K2 Vacutest® tubes of 4 mL (Lavender caps)) is taken (as part of the PROOF biomarker sub-study; see Section **Fehler! Verweisquelle konnte nicht gefunden werden.**; total blood amount: 18 mL). Blood for biomarkers should be drawn only from patients with study-independent venous or arterial access.<br>A 12-lead-ECG is repeated.	A third batch of blood samples for biomarker determination (i.e. two gel serum separation Vacutest® tubes of 5 mL (Yellow cap) and two plasma EDTA K2 Vacutest® tubes of 4 mL (Lavender caps)) is taken (as part of the PROOF biomarker sub-study; see Section **Fehler! Verweisquelle konnte nicht gefunden werden.**; total blood amount: 18 mL). Blood for biomarkers should be drawn only from patients with study-independent venous or arterial access.<br>A 12-lead-ECG is repeated.	
**9.9 V6 – Day 5 ±2 after start of NBHO (or randomization in the control arm) or at discharge (whichever occurs first) (page 45)**		
A fourth batch of blood samples for biomarker determination (i.e. two gel serum separation Vacutest® tubes of 5 mL (Yellow cap) and two plasma EDTA K2 Vacutest® tubes of 4 mL (Lavender caps)) is taken (as part of the PROOF biomarker sub-study, see Section **Fehler! Verweisquelle konnte nicht gefunden werden.**; total blood amount: 18 mL). Blood sampling for biomarkers at V6 is done either via a routinely inserted venous access or	A fourth batch of blood samples for biomarker determination (i.e. two gel serum separation Vacutest® tubes of 5 mL (Yellow cap) and two plasma EDTA K2 Vacutest® tubes of 4 mL (Lavender caps)) is taken (as part of the PROOF biomarker sub-study, see Section **Fehler! Verweisquelle konnte nicht gefunden werden.**; total blood amount: 18 mL). Blood sampling for biomarkers at V6 is done either via a routinely inserted venous access	

Previous and new wording in track change modus	New wording	Comments/ reasons for substantial amendment
– combined with the abovementioned safety laboratory assessment – through venipuncture. However, biomarker samples at V6 are only drawn if the patient or the respective LAR consents to participation in the biomarker sub-study.	or – combined with the abovementioned safety laboratory assessment – through venipuncture. However, biomarker samples at V6 are only drawn if the patient or the respective LAR consents to participation in the biomarker sub-study.	
**10.18 Safety laboratory assessment**		
Blood samples will be analyzed at the respective trial sites. All parameters will be documented on appropriate eCRF-pages.<br>Further laboratory parameters may be determined at any time during the study at discretion of the responsible investigator. Pathological and clinically relevant findings will be documented as adverse events/serious adverse events.<br>Total study-dependent blood volume for safety laboratory assessment: 16.5 mL.<br>In women ≤ 55 years a urine or serum β-HCG test has to be done before initiation of study treatment, except if surgically sterile; in women >55 years pregnancy must be excluded only in case of increased probability e.g. due to in-vitro fertilization.	Blood samples will be analyzed at the respective trial sites. All parameters will be documented on appropriate eCRF-pages.<br>Further laboratory parameters may be determined at any time during the study at discretion of the responsible investigator. Pathological and clinically relevant findings will be documented as adverse events/serious adverse events.<br>Total study-dependent blood volume for safety laboratory assessment: 16.5 mL.<br>In women ≤ 55 years a urine or serum β-HCG test has to be done before initiation of study treatment, except if surgically sterile; in women >55 years pregnancy must be excluded only in case of increased probability e.g. due to in-vitro fertilization.	
**10.19 Biomarkers (Pharmacodynamicy) Substudy**		
**Procedure**: Participants will be asked to consent to donate 4 blood samples to the PROOF blood biobank, which will be centralized at the Neurovascular Research Laboratory at the Fundació Hospital Universitari Vall d'Hebron-Institut de Recerca (VHIR) in Barcelona, Spain. Participation in the Biomarker sub-study is optional and analysis of biomarker samples will only be done if the patient/LAR consents to participate in the substudy (see Sections **Fehler! Verweisquelle konnte nicht gefunden**	**Procedure**: Participants will be asked to consent to donate 4 blood samples to the PROOF blood biobank, which will be centralized at the Neurovascular Research Laboratory at the Fundació Hospital Universitari Vall d'Hebron-Institut de Recerca (VHIR) in Barcelona, Spain. Participation in the Biomarker sub-study is optional and analysis of biomarker samples will only be done if the patient/LAR consents to participate in the substudy (see Sections **Fehler! Verweisquelle konnte nicht gefunden**	

Previous and new wording in track change modus	New wording	Comments/ reasons for substantial amendment
**werden., Fehler! Verweisquelle konnte nicht gefunden werden., Fehler! Verweisquelle konnte nicht gefunden werden.).**<br><br>Serum and plasma blood samples will be collected from each Participant at the following visits:<br>• Screening visit: 18mL<br>• Visit 3 (during study treatment): 18mL<br>• Visit 5 (24 ±6 hours): 18mL<br>• Visit 6 (Day 5 ±2): 18ml<br><br>Total study-dependent blood volume for biomarker analysis: 72 mL<br><br>Blood samples will be collected and processed following the PROOF Biomarkers handling and storage instructions, to be provided by VHIR in order to harmonize these procedures among the Participating Sites. Biological samples will be labelled with the PROOF participant ID number at each Participating Site. The Participating Site must store the blood samples in a biological sample freezer at -80°C, until the end of Study recruitment period. After the last visit of their last patient, the Participating Site will send its blood samples to the PROOF blood biobank at VHIR. VHIR will engage a common shipment company to collect and ship the samples to VHIR from each Participating Site. Once the blood samples have arrived at VHIR they will be stored in a biological samples freezer at -80°C until they are used for determination of blood-based biomarkers.<br><br>At the end of the Study, information from blood-based biomarkers will be tested to identify "NBHO therapy-response safety and efficacy biomarkers"	**werden., Fehler! Verweisquelle konnte nicht gefunden werden., Fehler! Verweisquelle konnte nicht gefunden werden.**).<br><br>Serum and plasma blood samples will be collected from each Participant at the following visits:<br>• Screening visit: 18mL<br>• Visit 3 (during study treatment): 18mL<br>• Visit 5 (24 ±6 hours): 18mL<br>• Visit 6 (Day 5 ±2): 18ml<br><br>Total study-dependent blood volume for biomarker analysis: 72 mL<br><br>Blood samples will be collected and processed following the PROOF Biomarkers handling and storage instructions, to be provided by VHIR in order to harmonize these procedures among the Participating Sites. Biological samples will be labelled with the PROOF participant ID number at each Participating Site. The Participating Site must store the blood samples in a biological sample freezer at -80°C, until the end of Study recruitment period. After the last visit of their last patient, the Participating Site will send its blood samples to the PROOF blood biobank at VHIR. VHIR will engage a common shipment company to collect and ship the samples to VHIR from each Participating Site. Once the blood samples have arrived at VHIR they will be stored in a biological samples freezer at -80°C until they are used for determination of blood-based biomarkers.<br><br>At the end of the Study, information from blood-based biomarkers will be tested to identify "NBHO therapy-response safety and efficacy biomarkers"	

Previous and new wording in track change modus	New wording	Comments/ reasons for substantial amendment
The specific objectives of this sub-study are:<br>○ To create a larger European bio resource of stroke blood samples (PROOF-Bio-Bank) that will allow to biologically demonstrating some of the expected benefits of NBO.<br>○ To measure blood biomarkers related with the clinical and neuroimaging endpoints and to define the clinical utility of candidate biomarkers involving three main pathways (Oxidative stress, matrix metalloproteinases and inflammation).<br>○ To define a prototype to be used as a Point-of-Care (POC) device for blood markers to guide stroke NBHO therapy triage and management.<br><br>**Quality assurance**: The procedures performed in the Neurovascular Research Laboratory at VHIR will follow a specific standard operating procedure (SOP) for sample maintenance and monitoring. This SOP will take into account all of the quality control requirements established by the VHIR and will comply with all applicable laws and regulations.<br><br>**Data management plan:** After analysis, any remaining blood samples will be kept in the Neurovascular Research Laboratory in VHIR in Barcelona as a collection registered with Instituto de Salud Carlos III, from the Spanish Ministry of Health, to be used in future studies in the line of stroke biomarkers, if consent has been given by the patient/LAR. ~~Blood samples for assessment of pharmacodynamics and biomarkers will be collected, processed and stored at the respective trial sites according to the PROOF Biomarkers Study Protocol, which will be~~	The specific objectives of this sub-study are:<br>○ To create a larger European bio resource of stroke blood samples (PROOF-Bio-Bank) that will allow to biologically demonstrating some of the expected benefits of NBO.<br>○ To measure blood biomarkers related with the clinical and neuroimaging endpoints and to define the clinical utility of candidate biomarkers involving three main pathways (Oxidative stress, matrix metalloproteinases and inflammation).<br>○ To define a prototype to be used as a Point-of-Care (POC) device for blood markers to guide stroke NBHO therapy triage and management.<br><br>**Quality assurance**: The procedures performed in the Neurovascular Research Laboratory at VHIR will follow a specific standard operating procedure (SOP) for sample maintenance and monitoring. This SOP will take into account all of the quality control requirements established by the VHIR and will comply with all applicable laws and regulations.<br><br>**Data management plan:** After analysis, any remaining blood samples will be kept in the Neurovascular Research Laboratory in VHIR in Barcelona as a collection registered with Instituto de Salud Carlos III, from the Spanish Ministry of Health, to be used in future studies in the line of stroke biomarkers, if consent has been given by the patient/LAR.	

Previous and new wording in track change modus	New wording	Comments/ reasons for substantial amendment
~~provided to the sites with the PROOF study documents, until shipment to the central PROOF biobank in Barcelona. Total study dependent blood volume for biomarker analysis: 72 mL.~~		
**11.1.6 Grading of AEs (page 53)**		
The grading of AEs in this trial will be carried out on the basis of the 5-grade scale defined in the CTCAE ~~v4.03~~ 5.0:	The grading of AEs in this trial will be carried out on the basis of the 5-grade scale defined in the CTCAE 5.0:	
The grading of all AEs listed in the CTCAE ~~v4~~ v5.0 will be based on the information contained therein. The grading of all other AEs, i.e., those not listed in the CTCAE ~~v4~~v5.0 will be performed by a responsible investigator, based on definitions given above.	The grading of all AEs listed in the CTCAE v5.0 will be based on the information contained therein. The grading of all other AEs, i.e., those not listed in the CTCAE v5.0 will be performed by a responsible investigator, based on definitions given above.	
**12.1 Sample Size Calculation (page 56)**		
The sample size calculation is based on the (absolute) ischemic core growth volumes reported by Albers et al., Ann Neurol, 2016 [126] – namely from the SWIFT-PRIME subgroup of TBY patients which reached successful recanalization at the end of TBY procedure (14.8 mL (IQR 4.9 to 33.7), N=62), which represents the group of patients in which we assume significant NBHO efficacy (based on positive experimental NBHO studies, which were studies in which focal cerebral ischemia was transient (with a duration of up to 3 hours), as a result of recanalization therapy. The mean and standard deviation are estimated as 17.8 mL ± 21.4 mL from the quartiles, assuming the quantiles of the normal distribution.	The sample size calculation is based on the (absolute) ischemic core growth volumes reported by Albers et al., Ann Neurol, 2016 [126] – namely from the SWIFT-PRIME subgroup of TBY patients which reached successful recanalization at the end of TBY procedure (14.8 mL (IQR 4.9 to 33.7), N=62), which represents the group of patients in which we assume significant NBHO efficacy (based on positive experimental NBHO studies, which were studies in which focal cerebral ischemia was transient (with a duration of up to 3 hours), as a result of recanalization therapy. The mean and standard deviation are estimated as 17.8 mL ± 21.4 mL from the quartiles, assuming the quantiles of the normal distribution.	
Thus, in PROOF, we assume a reduction of the relative ischemic core growth by 50% (of 17.8 mL, resolving to 8.9 mL) in 75% of NBHO-treated subjects: assuming a rate of successful recanalization of 80% [2, 126] in those 93.75% of patients who are randomized to NBHO and undergo TBY	Thus, in PROOF, we assume a reduction of the relative ischemic core growth by 50% (of 17.8 mL, resolving to 8.9 mL) in 75% of NBHO-treated subjects: assuming a rate of successful recanalization of 80% [2, 126] in those 93.75% of patients who are randomized to NBHO and undergo	

Previous and new wording in track change modus	New wording	Comments/ reasons for substantial amendment
(0,8 * 0.9375 = 0.75). Adding the other 25% (20% of the 93.75% TBY patients with no/insufficient recanalization plus 6.25% of PROOF-patients in whom TBY is not attempted) plus control subjects means that the mean effect of 8.9 is reduced by 25 per cent (leading to a mean effect of 6.68 mL) as well as setting the standard deviation to satisfy the variability found in ~~Albers et al.~~ [126] (i.e. 21.4 mL), we would need 138 patients per arm for a one-sided level-alpha = 0.05 test to detect a treatment effect with a power of 80 per cent. Using an adaptive design (according to [127]) after interim analysis after 80 patients per arm (assuming 20 sites with accrual up to interim analysis), the trial may be stopped early for success (with a p-value of less than 0.0233, which is taken from Table 1, column 2, row 3 in [127])) or futility (with p ≥ 0.5) or be continued with 11 to 148 additional patients per arm. Sample size inflations have been considered for site effect calculation and patients with failed recanalization not profiting from treatment.	TBY (0,8 * 0.9375 = 0.75). Adding the other 25% (20% of the 93.75% TBY patients with no/insufficient recanalization plus 6.25% of PROOF-patients in whom TBY is not attempted) plus control subjects means that the mean effect of 8.9 is reduced by 25 per cent (leading to a mean effect of 6.68 mL) as well as setting the standard deviation to satisfy the variability found in [126] (i.e. 21.4 mL), we would need 138 patients per arm for a one-sided level-alpha = 0.05 test to detect a treatment effect with a power of 80 per cent. Using an adaptive design (according to [127]) after interim analysis after 80 patients per arm (assuming 20 sites with accrual up to interim analysis), the trial may be stopped early for success (with a p-value of less than 0.0233, which is taken from Table 1, column 2, row 3 in [127])) or futility (with p ≥ 0.5) or be continued with 11 to 148 additional patients per arm. Sample size inflations have been considered for site effect calculation and patients with failed recanalization not profiting from treatment.	
**12.2.2 Secondary analysis variables (page 58)**		
• Incidence of any intracranial hemorrhage in 24-hour follow-up imaging (hemorrhagic transformation (HI1 and HI2) and intra-parenchymal hemorrhage (PH1 and PH2), as well as remote intracerebral hemorrhage (3a), intraventricular hemorrhage (3b), subarachnoid hemorrhage (3c), subdural hemorrhage (3d) and epidural hemorrhage; see **Fehler! Verweisquelle konnte nicht gefunden werden.** ~~Table 1~~ in [101] [time frame: 24 hours]	• Incidence of any intracranial hemorrhage in 24-hour follow-up imaging (hemorrhagic transformation (HI1 and HI2) and intra-parenchymal hemorrhage (PH1 and PH2), as well as remote intracerebral hemorrhage (3a), intraventricular hemorrhage (3b), subarachnoid hemorrhage (3c), subdural hemorrhage (3d) and epidural hemorrhage; see **Fehler! Verweisquelle konnte nicht gefunden werden.** in [101] [time frame: 24 hours]	
• Exploratory analyses of biochemical biomarkers will be useful to develop a blood-based test to monitor efficacy	• Exploratory analyses of biochemical biomarkers will be useful to develop a blood-based test to monitor	

Previous and new wording in track change modus	New wording	Comments/ reasons for substantial amendment
and safety of NBHO treatment. The specific objectives of this sub-study are:<br>○ To create a larger European bio resource of stroke blood samples (PROOF-Bio-Bank) that will allow to biologically demonstrate some of the expected benefits of NBHO.<br>○ To measure blood biomarkers related with the clinical and neuroimaging endpoints and to define the clinical utility of candidate biomarkers involving three main pathways (Oxidative stress, matrix metalloproteinases and inflammation).<br>○ To define a prototype to be used as a Point-of-Care (POC) device for blood markers to guide stroke NBHO therapy triage and management. ~~The precise scope of data acquisition and analysis is described in the Biomarker Study Protocol.~~	efficacy and safety of NBHO treatment. The specific objectives of this sub-study are:<br>○ To create a larger European bio resource of stroke blood samples (PROOF-Bio-Bank) that will allow to biologically demonstrate some of the expected benefits of NBHO.<br>○ To measure blood biomarkers related with the clinical and neuroimaging endpoints and to define the clinical utility of candidate biomarkers involving three main pathways (Oxidative stress, matrix metalloproteinases and inflammation).<br>○ To define a prototype to be used as a Point-of-Care (POC) device for blood markers to guide stroke NBHO therapy triage and management.	
**12.5 Interim Analysis (page 60)**		
An interim analysis on the primary endpoint will be completed after 160 patients have been treated. If the null hypothesis can be rejected at the α_1 = 0.0233 level, the trial will end prematurely with a successful rejection of the null hypothesis. If the null hypothesis cannot be rejected at the α_0 = 0.5 level (i.e. the observed treatment effect is in direction of the null hypothesis), the trial will be stopped early for futility. In both cases, a complete analysis will be completed on 160 patients plus the number of patients that were accrued after the criterion for interim analysis (160 patients) was satisfied but before the decision of early stopping was made.	An interim analysis on the primary endpoint will be completed after 160 patients have been treated. If the null hypothesis can be rejected at the α_1 = 0.0233 level, the trial will end prematurely with a successful rejection of the null hypothesis. If the null hypothesis cannot be rejected at the α_0 = 0.5 level (i.e. the observed treatment effect is in direction of the null hypothesis), the trial will be stopped early for futility. In both cases, a complete analysis will be completed on 160 patients plus the number of patients that were accrued after the criterion for interim analysis (160 patients) was satisfied but before the decision of early stopping was made.	

Previous and new wording in track change modus	New wording	Comments/ reasons for substantial amendment
**14.2.2 Other Legal Bases (page 62)**		
The other legal bases of this clinical trial are as follows (including their amendments/ up-dates, if applicable):<br>• ICH Topic E6, Guideline for Good Clinical Practice, including post Step 4 errata, September 1997<br>• Directive 2001/20/EC (April 4, 2001)<br>• Commission Directive 2005/28/EC (April 8, 2005)<br>• National regulatory requirements/guidelines of the participating countries concerning Clinical Trials<br>• General Data Protection Regulation (EU 2016/679)<br>• General national regulatory requirements~~, e.g. data protection laws~~	The other legal bases of this clinical trial are as follows (including their amendments/ up-dates, if applicable):<br>• ICH Topic E6, Guideline for Good Clinical Practice, including post Step 4 errata, September 1997<br>• Directive 2001/20/EC (April 4, 2001)<br>• Commission Directive 2005/28/EC (April 8, 2005)<br>• National regulatory requirements/guidelines of the participating countries concerning Clinical Trials<br>• General Data Protection Regulation (EU 2016/679)<br>• General national regulatory requirements	
**15.2 Data Protection (page 66)**		
The data obtained in the study will be treated pursuant to the General Data Protection Regulation (EU 2016/679). During the clinical trial, subjects will be identified solely by means of their individual identification code (subject number, randomization number). Trial data stored on a computer will be stored in accordance with local data protection law and will be handled in strictest confidence. Distribution of these data to unauthorized persons has to be prevented strictly. The appropriate regulations of local data legislation will be fulfilled in its entirety.	The data obtained in the study will be treated pursuant to the General Data Protection Regulation (EU 2016/679). During the clinical trial, subjects will be identified solely by means of their individual identification code (subject number, randomization number). Trial data stored on a computer will be stored in accordance with local data protection law and will be handled in strictest confidence. Distribution of these data to unauthorized persons has to be prevented strictly. The appropriate regulations of local data legislation will be fulfilled in its entirety.	
**16.3 Reports (page 68)**		
Within the defined timeframe (e.g. for Germany within one year after completion of the trial (trial end is defined as last ~~patient~~subject out)) the competent authorities and the ethics committees will be supplied with this final report or a summary of the final report containing the principle results. Dependent on national regulations the trial report will be published in a clinical trial register via the competent authority. By signing this protocol, the investigators agree	Within the defined timeframe (e.g. for Germany within one year after completion of the trial (trial end is defined as last subject out)) the competent authorities and the ethics committees will be supplied with this final report or a summary of the final report containing the principle results. Dependent on national regulations the trial report will be published in a clinical trial register via the competent authority. By signing this protocol, the investigators agree	

Previous and new wording in track change modus	New wording	Comments/ reasons for substantial amendment
to disclose their names/ clinic address in the trial report.	to disclose their names/ clinic address in the trial report.	
