# Supplementary material for: Penumbral Rescue by normobaric O = O administration in patients with ischemic stroke and target mismatch proFile (PROOF): Study protocol of a phase IIb trial
Source: Int J Stroke. 2023 Aug 18;19(1):120–6. doi: 10.1177/17474930231185275 (PMC10759237; doi:10.1177/17474930231185275)
Supplement: sj-pdf-2-wso-10.1177_17474930231185275 – Supplemental material for Penumbral Rescue by normobaric O = O administration in patients with ischemic stroke and target mismatch proFile (PROOF): Study protocol of a phase IIb trial [file sj-pdf-2-wso-10.1177_17474930231185275.pdf]

Appendix 1 Description of the substantial amendment

Clinical Study Protocol "PROOF: Penumbral Rescue by Normobaric O=O Administration in Patients with Ischemic Stroke and Target Mismatch ProFile: A Phase II Proof-of-Concept Trial"

**Phase of study:**	**Phase II – proof-of-concept**
**EudraCT No.:**	**2017-001355-31**
**Study Registry Number:**	**NCT03500939**

The respective changes made to the Protocol Version 1.1/ 30.05.2018 to Version 1.2/ 14.03.2019 are tabulated below.

Previous and new wording in track change modus	New wording	Comments/ reasons for substantial amendment
**Administrative Structure (page 2)**		
University Hospital Tübingen<br>Dept. of Neurology with Focus on Neurovascular Diseases and Neurooncology Dept. of Neurology & Stroke	University Hospital Tübingen<br>Dept. of Neurology with Focus on Neurovascular Diseases and Neurooncology	
**Project Management: (page 2)**		
Dr. Maike Nilsson (Ph.D.) Dr. sc. hum. Anja Dietzel<br>Email: anja.dietzel maike.nilsson@med.uni-heidelberg.de	Dr. Maike Nilsson (Ph.D.)<br>Email: maike.nilsson@med.uni-heidelberg.de	
**Central Contact to local CROs (page 3)**		
ECRIN (European Clinical Research Infrastructure Network)<br>Dr. rer. nat. Linda Stoehr<br>European Correspondent for ECRIN<br>Alt-Moabit 96aKerpener Str.62<br>10559 Berlin50937 Cologne<br>Germany<br>Phone: 0049 30 4053 5400 004922147889331<br>Fax: 0049 30 6959 9990 004922147896510<br><br>Email: linda.stoehr@kksn.de linda.stoehr@uk-koeln.de	ECRIN (European Clinical Research Infrastructure Network)<br>Dr. rer. nat. Linda Stoehr<br>European Correspondent for ECRIN<br>Alt-Moabit 96a<br>10559 Berlin<br>Germany<br>Phone: 0049 30 4053 5400<br>Fax: 0049 30 6959 9990<br>Email: linda.stoehr@kksn.de	
**Data and Safety Monitoring Board (DSMB) (page 3)**		
Prof. Dr. med. Gerhard Schroth	Prof. Dr. med. Gerhard Schroth	
**Steering Committee (SC) (page 5)**		

Previous and new wording in track change modus	New wording	Comments/ reasons for substantial amendment
Eppdata GmbH<br>Dr. Paulo Dellani<br>Locksteder Steindamm 18 ~~Christoph-Probst-Weg 1~~<br>22529 ~~20251~~ Hamburg	Eppdata GmbH<br>Dr. Paulo Dellani<br>Locksteder Steindamm 18<br>22529 Hamburg	
**Central technical facilities to be involved in the conduct of the trial, in which the measurement or assessment of the main evaluation criteria are centralized: (page 5)**		
CORE IMAGING LABORATORY<br>~~For analysis of imaging data~~	CORE IMAGING LABORATORY	
CORE BIOMARKER LABORATORY<br>~~For analysis of biomarkers~~	CORE BIOMARKER LABORATORY	
**Study Population (page 11)**		
Inclusion Criteria<br>• LVO on CT angiography or MR angiography consistent with clinical signs and symptoms, i.e. either the terminal ICA with involvement of the M1-segment of the MCA/carotid-T, the proximal M1-segment, or the distal M1-segments (distal to perforating branches) Neither TBY nor IVT are a prerequisite for inclusion; patients not receiving TBY or IVT or both can be enrolled. Clinical treatment decisions should not delay study enrollment.<br>• Breastfeeding women can participate, but must be instructed to stop breastfeeding after randomization	Inclusion Criteria<br>• LVO on CT angiography or MR angiography consistent with clinical signs and symptoms, i.e. either the terminal ICA with involvement of the M1-segment of the MCA/carotid-T, the proximal M1-segment, or the distal M1-segments (distal to perforating branches) Neither TBY nor IVT are a prerequisite for inclusion; patients not receiving TBY or IVT or both can be enrolled. Clinical treatment decisions should not delay study enrollment.<br>• Breastfeeding women can participate, but must be instructed to stop breastfeeding after randomization	
**Exclusion criteria (page 12)**		

Previous and new wording in track change modus	New wording	Comments/ reasons for substantial amendment
Respiratory:<br>• Acute viral, bacterial or fungal pneumonia Acute pneumonia, alveolitis or pneumonitis of viral, bacterial, fungal or any other etiology	Respiratory:<br>• Acute pneumonia, alveolitis or pneumonitis of viral, bacterial, fungal or any other etiology	
**Trial Duration (page 13)**		
FSI (first subject in): Q2 2019 2018	FSI (first subject in): Q2 2019	
**Prevention, early detection and management of adverse events in the PROOF-trial (page 27)**		
Prevention: In the PROOF-trial, NBHO treatment is limited to a maximum of four hours (which is the longest planned exposure to 100% oxygen inhalation). This time frame was chosen in order to maintain the highest possible safety with regard to potential oxygen toxicity. Thus, only mild and fully reversible adverse effects of oxygen administration are to be expected in the PROOF trial.<br>Patients with a history of COPD and other pulmonary disorders are not to be enrolled in this trial (see Section **Fehler! Verweisquelle konnte nicht gefunden werden.** Exclusion Criteria). Use of concomitant medications which may increase the risk of adverse events are prohibited (see Section **Fehler! Verweisquelle konnte nicht gefunden werden.** Prior and Concomitant Medication). To avoid harm for unborn children, pregnancy must be excluded in all women ≤55 years (except if surgically sterile) and in women >55 years in case of increased probability for pregnancy (e.g. due to in-vitro fertilization). Breastfeeding women can participate, but must be instructed to stop breastfeeding after randomization.	Prevention: In the PROOF-trial, NBHO treatment is limited to a maximum of four hours (which is the longest planned exposure to 100% oxygen inhalation). This time frame was chosen in order to maintain the highest possible safety with regard to potential oxygen toxicity. Thus, only mild and fully reversible adverse effects of oxygen administration are to be expected in the PROOF trial.<br>Patients with a history of COPD and other pulmonary disorders are not to be enrolled in this trial (see Section **Fehler! Verweisquelle konnte nicht gefunden werden.** Exclusion Criteria). Use of concomitant medications which may increase the risk of adverse events are prohibited (see Section **Fehler! Verweisquelle konnte nicht gefunden werden.** Prior and Concomitant Medication). To avoid harm for unborn children, pregnancy must be excluded in all women ≤55 years (except if surgically sterile) and in women >55 years in case of increased probability for pregnancy (e.g. due to in-vitro fertilization). Breastfeeding women can participate, but must be instructed to stop breastfeeding after randomization.	
**RISK-Benefit (page 27/28)**		
**4.3.1 Risk-Benefit Assessment Considering the Most Recent**		

Previous and new wording in track change modus	New wording	Comments/ reasons for substantial amendment
**Literature**<br>This chapter will discuss any ambiguity regarding the risk-benefit assessment and consent modalities of the PROOF study trial considering most recently published review articles including one meta-analysis.<br>For the risk-benefit evaluation it is important to know – especially because patients that are unable to consent are to be included in the PROOF trial – that despite modern day reperfusion therapy with endovascular mechanical thrombectomy ± intravenous thrombolysis, 54% of patients are still left with long term disabilities and remain functionally dependent or even die (mRS ≥ 3) (e.g. [100]). Thus, there is an urgent need for new and adjuvant therapy for the PROOF trial population; a group of patients with the most severe strokes. These patients unfortunately lose a lot and therefore have a lot to gain from such developments.<br>All representatives of the PROOF study group are convinced that NBHO can only reduce brain damage in acute ischemic strokes if it is conducted as set out in the PROOF trial protocol and that we do not only expect a group effect but, in fact, a positive influence on the course of every patient's condition who is treated with NBHO. Moreover, with the help of our own meta-analysis of the original papers relevant for the potential PROOF study population (see Appendix 1), we can ascertain that using a high dose but – in the interest of patient security and maximum efficacy – a short duration, the oxygen therapy planned for the PROOF trial poses no significant risk to the patient suffering an acute ischemic stroke due to a proximal vessel occlusion.<br><br>**Requirements for successful oxygen treatment in animal experiments**<br>During animal testing, NBHO was proven to be very effective and reduced the volume of cerebral infarction by up to 50% in comparison to non-hyperoxygenated animals; however, only if three framework conditions were fulfilled. NBHO cannot stabilize and	**4.3.1 Risk-Benefit Assessment Considering the Most Recent Literature**<br>This chapter will discuss any ambiguity regarding the risk-benefit assessment and consent modalities of the PROOF study trial considering most recently published review articles including one meta-analysis.<br>For the risk-benefit evaluation it is important to know – especially because patients that are unable to consent are to be included in the PROOF trial – that despite modern day reperfusion therapy with endovascular mechanical thrombectomy ± intravenous thrombolysis, 54% of patients are still left with long term disabilities and remain functionally dependent or even die (mRS ≥ 3) (e.g. [100]). Thus, there is an urgent need for new and adjuvant therapy for the PROOF trial population; a group of patients with the most severe strokes. These patients unfortunately lose a lot and therefore have a lot to gain from such developments.<br>All representatives of the PROOF study group are convinced that NBHO can only reduce brain damage in acute ischemic strokes if it is conducted as set out in the PROOF trial protocol and that we do not only expect a group effect but, in fact, a positive influence on the course of every patient's condition who is treated with NBHO. Moreover, with the help of our own meta-analysis of the original papers relevant for the potential PROOF study population (see Appendix 1), we can ascertain that using a high dose but – in the interest of patient security and maximum efficacy – a short duration, the oxygen therapy planned for the PROOF trial poses no significant risk to the patient suffering an acute ischemic stroke due to a proximal vessel occlusion.<br><br>**Requirements for successful oxygen treatment in animal experiments**<br>During animal testing, NBHO was proven to be very effective and reduced the volume of cerebral infarction by up to 50% in comparison to non-hyperoxygenated animals; however, only if three	

Previous and new wording in track change modus	New wording	Comments/ reasons for substantial amendment
keep alive indefinitely the collateralized and critically malperfused and thus hypoxic margins of the area undergoing infarction, which is bound to perish (the so-called penumbra). NBHO was only consistently effective in animal experiments when the ischemia was transient and lasted a maximum of three hours, not so in delayed or missing vessel recanalization and brain tissue reperfusion (see Fig. 1 and compare final infarct versus failed recanalization in Fig. 3) [51].	framework conditions were fulfilled. NBHO cannot stabilize and keep alive indefinitely the collateralized and critically malperfused and thus hypoxic margins of the area undergoing infarction, which is bound to perish (the so-called penumbra). NBHO was only consistently effective in animal experiments when the ischemia was transient and lasted a maximum of three hours, not so in delayed or missing vessel recanalization and brain tissue reperfusion (see Fig. 1 and compare final infarct versus failed recanalization in Fig. 3) [51].	
**Figure 1 (page 28)**		
**Figure 1: Spatiotemporal evolution of threshold-derived apparent diffusion coefficient (ADC) and cerebral blood flow (CBF) lesion volumes in an experimental model of ischaemic stroke treated with 100% normobaric oxygen (NBO)**. Animals in this experiment were subjected to (A) permanent middle cerebral artery occlusion (pMCAO) or (B) transient MCAO (tMCAO) and received either NBO or room air (control condition). Asterisks denote significant between-group difference in ADC- and CBF-derived lesion volumes ($P < 0.05$). Note that the statistically significant positive effect of NBO is only translated onto a later time-point (in this case definitive infarct volume as determined by 24h triphenyl tetrazolium chloride (TTC)-staining)) if MCAO is transient and not permanent. (from [61])	**Figure 1: Spatiotemporal evolution of threshold-derived apparent diffusion coefficient (ADC) and cerebral blood flow (CBF) lesion volumes in an experimental model of ischaemic stroke treated with 100% normobaric oxygen (NBO)**. Animals in this experiment were subjected to (A) permanent middle cerebral artery occlusion (pMCAO) or (B) transient MCAO (tMCAO) and received either NBO or room air (control condition). Asterisks denote significant between-group difference in ADC- and CBF-derived lesion volumes ($P < 0.05$). Note that the statistically significant positive effect of NBO is only translated onto a later time-point (in this case definitive infarct volume as determined by 24h triphenyl tetrazolium chloride (TTC)-staining)) if MCAO is transient and not permanent. (from [61])	
**Figure 2 (page 29)**		
**Figure 2: Effect of treatment delay of normobaric hyperoxygenation in a rat tMCAO model.** **4A:** Schematic representation of the experiment protocol. All animals were anesthetized with 1 % halothane. White bars represent a breathing mixture containing 70 % nitrous oxide and 30 % oxygen. Black bars represent a breathing mixture containing 100 % oxygen. **4B:** Total lesion volumes at 48 hours after MCAO. (*$p < 0.05$ vs. normoxia group). TTC: 2 % 2,3,5-triphenyltetrazolium chloride. (from [101])	**Figure 2: Effect of treatment delay of normobaric hyperoxygenation in a rat tMCAO model.** **4A:** Schematic representation of the experiment protocol. All animals were anesthetized with 1 % halothane. White bars represent a breathing mixture containing 70 % nitrous oxide and 30 % oxygen. Black bars represent a breathing mixture containing 100 % oxygen. **4B:** Total lesion volumes at 48 hours after MCAO. (*$p < 0.05$ vs. normoxia group). TTC: 2 % 2,3,5-triphenyltetrazolium chloride. (from [101])	
Furthermore, NBHO in animal experiments was only effective when it was initiated early on after the start of ischemia (see Fig. 2 and compare rows 3 and 4 in Fig. 3) [65] and almost pure oxygen was	Furthermore, NBHO in animal experiments was only effective when it was initiated early on after the start of ischemia (see Fig. 2 and compare rows 3 and 4 in Fig. 3) [65] and almost pure oxygen was	

Previous and new wording in track change modus	New wording	Comments/ reasons for substantial amendment
breathed in normal atmospheric pressure environment. An inspiratory oxygen fraction of 70% (FiO_2 0.7) did not suffice, the ischemic penumbra was only sufficiently oxygenated at an $FiO_2 \geq 0.95$ (see Table 2) [53].	breathed in normal atmospheric pressure environment. An inspiratory oxygen fraction of 70% (FiO_2 0.7) did not suffice, the ischemic penumbra was only sufficiently oxygenated at an $FiO_2 \geq 0.95$ (see Table 2) [53].	
**Table 2 (page 29)**		
**Table 2: Blood gas and penumbral pO_2 measurement**<br><br>The ischemic penumbra can thus be stabilized by breathing almost pure oxygen early on ('freezing the penumbra'), but can only survive if the blocked vessel is recanalized within *three hours ('nothing can hold its breath forever') (see Fig. 3) [102].*	**Table 2: Blood gas and penumbral pO_2 measurement**<br><br>The ischemic penumbra can thus be stabilized by breathing almost pure oxygen early on ('freezing the penumbra'), but can only survive if the blocked vessel is recanalized within three hours ('nothing can hold its breath forever') (see Fig. 3) [102].	
**Figure 3 (page 30)**		
**Figure 3: The penumbral freezing paradigm.** The figure depicts an idealized time course of the ischaemic penumbra and core in various scenarios after stroke. In the real world, accurate mapping of the penumbra and core is challenging.<br><br>**The top row shows the typical natural history** of tissue evolution after acute middle cerebral artery (MCA) occlusion. By 3 hours after stroke onset, the centre of the MCA territory, where perfusion is lowest, is irreversibly damaged. This 'core' is surrounded by a rim of 'penumbra', where perfusion is better preserved owing to leptomeningeal collaterals, which are fed by the neighbouring anterior and posterior cerebral arteries. Without recanalization, more penumbra progresses to core tissue. eventually, the at-risk penumbra turns into core, and no salvageable tissue remains. The final infarct, which comprises the initial core and penumbra, is large, causing severe disability (modified Rankin Scale (mRS) score 5). The final infarct size is not the only determinant of final outcome: infarct topography, age, comorbidities, previous stroke and white matter ischaemic changes are also important factors. **The second row illustrates the benefits of recanalization** achieved at 4.5 hours, which stops core growth, resulting in a smaller final infarct and an mRS score of 3 — much better than with no reperfusion but still above the threshold for functional independence (mRS score 2). If recanalization fails, the outcome is the	**Figure 3: The penumbral freezing paradigm.** The figure depicts an idealized time course of the ischaemic penumbra and core in various scenarios after stroke. In the real world, accurate mapping of the penumbra and core is challenging.<br><br>**The top row shows the typical natural history** of tissue evolution after acute middle cerebral artery (MCA) occlusion. By 3 hours after stroke onset, the centre of the MCA territory, where perfusion is lowest, is irreversibly damaged. This 'core' is surrounded by a rim of 'penumbra', where perfusion is better preserved owing to leptomeningeal collaterals, which are fed by the neighbouring anterior and posterior cerebral arteries. Without recanalization, more penumbra progresses to core tissue. eventually, the at-risk penumbra turns into core, and no salvageable tissue remains. The final infarct, which comprises the initial core and penumbra, is large, causing severe disability (modified Rankin Scale (mRS) score 5). The final infarct size is not the only determinant of final outcome: infarct topography, age, comorbidities, previous stroke and white matter ischaemic changes are also important factors. **The second row illustrates the benefits of recanalization** achieved at 4.5 hours, which stops core growth, resulting in a smaller final infarct and an mRS score of 3 — much better than with no reperfusion but still above the threshold for functional independence (mRS score 2). If recanalization fails, the outcome is the	

Previous and new wording in track change modus	New wording	Comments/ reasons for substantial amendment
same as in the natural history scenario. **The third row shows the effects of freezing therapy by normobaric hyperoxygenation initiated on hospital admission**, which enables core growth to stop until recanalization is achieved 1.5 hours later. owing to extensive penumbral salvage, the final infarct is the same size as the core on admission, and the patient achieves functional independence (mRS score 2). Again, if thrombectomy fails, the final outcome is the same as in the natural history scenario. **The fourth row illustrates the theoretical effects of efficacious freezing therapy started prehospitally** 1.5 hours after stroke onset. This strategy allows maximal penumbral salvage and minimal final infarct, resulting in mild residual symptoms but no functional disability (mRS score 1).<br>**Nonetheless, in the PROOF phase II proof–of–concept study we decided against the in-the-field approach for several reasons and will leave this open for a follow-up phase III trial:** Firstly, the lack of prehospital brain imaging prior to initiation of oxygen therapy does not allow (1) the exclusion of patients with intracranial haemorrhage (as current experimental and clinical study data does not support use of oxygen therapy in haemorrhagic stroke), (2) the exclusion of patients with ischaemic stroke but without salvageable brain tissue (as no effect of oxygen therapy can be anticipated), (3) comprehensive baseline assessment which we judge crucial in a phase II proof–of–concept study. Secondly, although NBHO as foreseen in the PROOF trial (i.e. high-dose, but short duration, and stop after reperfusion) can be considered safe based on current experimental and clinical study data, nothing replaces the close monitoring by stroke experts not available in the prehospital environment. (modified from [102])	same as in the natural history scenario. **The third row shows the effects of freezing therapy by normobaric hyperoxygenation initiated on hospital admission**, which enables core growth to stop until recanalization is achieved 1.5 hours later. owing to extensive penumbral salvage, the final infarct is the same size as the core on admission, and the patient achieves functional independence (mRS score 2). Again, if thrombectomy fails, the final outcome is the same as in the natural history scenario. **The fourth row illustrates the theoretical effects of efficacious freezing therapy started prehospitally** 1.5 hours after stroke onset. This strategy allows maximal penumbral salvage and minimal final infarct, resulting in mild residual symptoms but no functional disability (mRS score 1).<br>**Nonetheless, in the PROOF phase II proof–of–concept study we decided against the in-the-field approach for several reasons and will leave this open for a follow-up phase III trial:** Firstly, the lack of prehospital brain imaging prior to initiation of oxygen therapy does not allow (1) the exclusion of patients with intracranial haemorrhage (as current experimental and clinical study data does not support use of oxygen therapy in haemorrhagic stroke), (2) the exclusion of patients with ischaemic stroke but without salvageable brain tissue (as no effect of oxygen therapy can be anticipated), (3) comprehensive baseline assessment which we judge crucial in a phase II proof–of–concept study. Secondly, although NBHO as foreseen in the PROOF trial (i.e. high-dose, but short duration, and stop after reperfusion) can be considered safe based on current experimental and clinical study data, nothing replaces the close monitoring by stroke experts not available in the prehospital environment. (modified from [102])	

Previous and new wording in track change modus	New wording	Comments/ reasons for substantial amendment
**Have previous human testing studies fulfilled the requirements?**<br>Not one of the previous human trials testing oxygen therapy has fulfilled the three framework conditions (see Table 3).<br>Only in one stroke study on humans was oxygen therapy started early – up to 4.5 hours after symptom onset – and reperfusion treatment performed [103], whilst the sole use of intravenous thrombolysis can bring about vessel recanalization and brain tissue reperfusion in only 40 to 50% of cases [104]. Therefore, it can be assumed that despite reperfusion treatment more than half of the patients included in this trial had permanent ischemia, where – as seen in animal experiments – oxygen therapy remains ineffective. In only two (other) human trials, oxygen therapy was administered with a sufficiently high dosage at an FiO_2 of 1.0, however, initiated relatively late after symptoms onset (thus salvageable less brain tissue present) and, above all, no reperfusion treatment was performed [75, 105].<br>Chu and colleagues concluded that the present clinical trial situation excludes a positive effect of oxygen therapy on acute ischemic strokes and that thus further studies are not necessary [106], which is simply and poignantly incorrect, as in no prior clinical trial including humans was oxygen therapy ever given the chance to succeed. Researchers Grensemann [107] and Stolmeijer [108] and their colleagues see this somewhat more differentiated and demand prospective and randomized clinical studies albeit the latter not regarding strokes. His review, dedicated to oxygen therapy in acute ischemic strokes, Shi [109] calls “ideas” for the design of future studies.<br>Nonetheless, in the few human clinical trials where at least one framework condition was fulfilled, the evidence points to a clinical benefit to acute ischemic strokes: Breathing oxygen in a dose of $FiO_2 \geq 0.95$, which was proven sufficient in animal experiments, led to a stabilization or even a regression of the ischemia (see Fig. 4) and an improvement in NIHHS scores [75]. However, this effect was	**Have previous human testing studies fulfilled the requirements?**<br>Not one of the previous human trials testing oxygen therapy has fulfilled the three framework conditions (see Table 3).<br>Only in one stroke study on humans was oxygen therapy started early – up to 4.5 hours after symptom onset – and reperfusion treatment performed [103], whilst the sole use of intravenous thrombolysis can bring about vessel recanalization and brain tissue reperfusion in only 40 to 50% of cases [104]. Therefore, it can be assumed that despite reperfusion treatment more than half of the patients included in this trial had permanent ischemia, where – as seen in animal experiments – oxygen therapy remains ineffective. In only two (other) human trials, oxygen therapy was administered with a sufficiently high dosage at an FiO_2 of 1.0, however, initiated relatively late after symptoms onset (thus salvageable less brain tissue present) and, above all, no reperfusion treatment was performed [75, 105].<br>Chu and colleagues concluded that the present clinical trial situation excludes a positive effect of oxygen therapy on acute ischemic strokes and that thus further studies are not necessary [106], which is simply and poignantly incorrect, as in no prior clinical trial including humans was oxygen therapy ever given the chance to succeed. Researchers Grensemann [107] and Stolmeijer [108] and their colleagues see this somewhat more differentiated and demand prospective and randomized clinical studies albeit the latter not regarding strokes. His review, dedicated to oxygen therapy in acute ischemic strokes, Shi [109] calls “ideas” for the design of future studies.<br>Nonetheless, in the few human clinical trials where at least one framework condition was fulfilled, the evidence points to a clinical benefit to acute ischemic strokes: Breathing oxygen in a dose of $FiO_2 \geq 0.95$, which was proven sufficient in animal experiments, led to a stabilization or even a regression of the ischemia (see Fig. 4) and an improvement in NIHHS scores [75]. However, this effect was	

Previous and new wording in track change modus	New wording	Comments/ reasons for substantial amendment
only temporary because no reperfusion treatment was performed on the patients ('nothing can hold its breath forever', compare Fig. 1 and final infarct versus failed recanalization in Fig. 3).	only temporary because no reperfusion treatment was performed on the patients ('nothing can hold its breath forever', compare Fig. 1 and final infarct versus failed recanalization in Fig. 3).	
**Figure 4 (page 32)**		
**Figure 4: Evolution of DWI-lesion volume during permanent MCA-occlusion treated with NBHO** Serial MRI findings in a patient with cardio-embolic right MCA stroke treated with NBO for 8 hours. Top, Baseline (pre-NBO) MRI, 13.1 hours after symptom onset, shows a large DWI lesion, a larger MTT lesion, and MCA occlusion (arrow) on head MRA. Middle, A second MRI after 3.75 hours (during NBO) shows 36 % reduction in the DWI lesion, stable MTT deficit, and persistent MCA occlusion. Bottom, A third MRI after 24 hours (post-NBO) shows reappearance of DWI abnormality in some areas of previous reversal; MTT image shows partial reperfusion (39 % MTT volume reduction, mainly in the ACA territory); MRA shows partial MCA recanalization. (from [75])	**Figure 4: Evolution of DWI-lesion volume during permanent MCA-occlusion treated with NBHO** Serial MRI findings in a patient with cardio-embolic right MCA stroke treated with NBO for 8 hours. Top, Baseline (pre-NBO) MRI, 13.1 hours after symptom onset, shows a large DWI lesion, a larger MTT lesion, and MCA occlusion (arrow) on head MRA. Middle, A second MRI after 3.75 hours (during NBO) shows 36 % reduction in the DWI lesion, stable MTT deficit, and persistent MCA occlusion. Bottom, A third MRI after 24 hours (post-NBO) shows reappearance of DWI abnormality in some areas of previous reversal; MTT image shows partial reperfusion (39 % MTT volume reduction, mainly in the ACA territory); MRA shows partial MCA recanalization. (from [75])	
**Figure 5 (32)**		
**Figure 5: Normobaric hyperoxia (NBO) reduced blood occludin and improved neurological functions in patients with acute ischemic stroke (AIS).** **A,** Blood occludin levels in normoxia and NBO-treated patients with AIS.N=8. **B,** National Institutes of Health Stroke Scale (NIHSS) scores of patients with AIS. N = 9. # $p < 0.05$ vs. normoxia group at the same time point. Data were presented as means ± SEM. (from [103])	**Figure 5: Normobaric hyperoxia (NBO) reduced blood occludin and improved neurological functions in patients with acute ischemic stroke (AIS).** **A,** Blood occludin levels in normoxia and NBO-treated patients with AIS.N=8. **B,** National Institutes of Health Stroke Scale (NIHSS) scores of patients with AIS. N = 9. # $p < 0.05$ vs. normoxia group at the same time point. Data were presented as means ± SEM. (from [103])	

Previous and new wording in track change modus	New wording	Comments/ reasons for substantial amendment
The only human study in which reperfusion treatment (by means of intravenous thrombolysis) was successful shows a reduction in laboratory parameters that point to blood-brain-barrier damage, as well as an improvement of the NIHSS score at later time points (see Fig. 5) [103].	The only human study in which reperfusion treatment (by means of intravenous thrombolysis) was successful shows a reduction in laboratory parameters that point to blood-brain-barrier damage, as well as an improvement of the NIHSS score at later time points (see Fig. 5) [103].	
**How does the PROOF trial differ?**<br>The PROOF study is the first human NBHO trial to consider all three framework requirements for a successful "freezing of the penumbra" and lasting conservation of positive effects through: (1) early reperfusion (compare Fig. 1 and final infarct versus failed recanalization in Fig. 3): Due to the selected inclusion and exclusion criteria, such as proximal vessel occlusion, a narrow therapeutic time window (three hours) and the maximum permitted age of 80 years, all patients included in PROOF will likely be treated by endovascular mechanical thrombectomy (TBY) ± intravenous thrombolysis, in which for > 80% of cases complete brain tissue reperfusion is reached, thus achieving transient ischemia, which is deemed necessary for successful oxygen therapy as concluded from animal experiments [104]. (2) Early start of oxygen therapy: the therapeutic time window of three hours after symptom onset was chosen for reasons of practicality and is indeed longer than the 30 minute time window proven effective in animal experiments (compare Fig. 2), however, in contrast to animal experiments, a "target mismatch" on cerebral imaging as stipulated in PROOF guarantees the presence of a substantial volume of salvageable penumbra which is threatened to decline (compare rows 3 and 4 in Fig. 3). (3) Sufficient oxygen dosage: through the high flow of 40 L/min or more, even patients who are agitated in the emergency situation and therefore breathe faster and deeper receive oxygen at the FiO_2 level of over 0.95, as proven successful in animal experiments (see Table 2).<br>Through the consistent implementation of all three framework conditions, the PROOF study is thus the first human trial where	**How does the PROOF trial differ?**<br>The PROOF study is the first human NBHO trial to consider all three framework requirements for a successful "freezing of the penumbra" and lasting conservation of positive effects through: (1) early reperfusion (compare Fig. 1 and final infarct versus failed recanalization in Fig. 3): Due to the selected inclusion and exclusion criteria, such as proximal vessel occlusion, a narrow therapeutic time window (three hours) and the maximum permitted age of 80 years, all patients included in PROOF will likely be treated by endovascular mechanical thrombectomy (TBY) ± intravenous thrombolysis, in which for > 80% of cases complete brain tissue reperfusion is reached, thus achieving transient ischemia, which is deemed necessary for successful oxygen therapy as concluded from animal experiments [104]. (2) Early start of oxygen therapy: the therapeutic time window of three hours after symptom onset was chosen for reasons of practicality and is indeed longer than the 30 minute time window proven effective in animal experiments (compare Fig. 2), however, in contrast to animal experiments, a "target mismatch" on cerebral imaging as stipulated in PROOF guarantees the presence of a substantial volume of salvageable penumbra which is threatened to decline (compare rows 3 and 4 in Fig. 3). (3) Sufficient oxygen dosage: through the high flow of 40 L/min or more, even patients who are agitated in the emergency situation and therefore breathe faster and deeper receive oxygen at the FiO_2 level of over 0.95, as proven successful in animal experiments (see Table 2).<br>Through the consistent implementation of all three framework conditions, the PROOF study is thus the first human trial where	

Previous and new wording in track change modus	New wording	Comments/ reasons for substantial amendment
NBHO has the chance of clinical success.<br><br>Additional aspects of the PROOF trial design, such as the determination time point (24 hours) and the choice of the primary end point (imaging and intra-individual control), start of therapy (not prior to hospital admission but and as soon as possible after cerebral imaging) and the duration of therapy (only until reperfusion), reduce the number of lost to follow-up, raise the detection sensitivity for an oxygen effect, thereby minimizing the number of required cases and maximizing patient safety and feasibility, and consequently the chance of success for PROOF and oxygen therapy.	NBHO has the chance of clinical success.<br><br>Additional aspects of the PROOF trial design, such as the determination time point (24 hours) and the choice of the primary end point (imaging and intra-individual control), start of therapy (not prior to hospital admission but and as soon as possible after cerebral imaging) and the duration of therapy (only until reperfusion), reduce the number of lost to follow-up, raise the detection sensitivity for an oxygen effect, thereby minimizing the number of required cases and maximizing patient safety and feasibility, and consequently the chance of success for PROOF and oxygen therapy.	
**Table 3 (page 34)**		
**Table 3: Implementation of prerequisites for successful oxygen therapy (OT) as known from animal experiments into randomized trials testing OT in patients with stroke, myocardial infarction and cardiac arrest**<br>Green, yellow, red colour indicates that prerequisites for successful OT were met, only partly addressed, not fulfilled. ☾ see respective study protocol, * IVT leads to recanalization in only 40 to 50 % compared to > 80 % with EVT or PCI [104], [110], ** Long therapeutic windows renders OT start before reperfusion unlikely, $ estimated value based on median oxygen exposure (2258 L) during first 12 hours divided by median flow-rate (6 L/min), see results, study population [111]<br><br>**EVT, endovascular mechanical thrombectomy; FiO_2, fraction of inspired oxygen; IQR, interquartile range; IVT, intravenous thrombolysis; OT, oxygen therapy; PCI, percutaneous coronary intervention; ROSC, return of spontaneous circulation; SD, standard deviation**	**Table 3: Implementation of prerequisites for successful oxygen therapy (OT) as known from animal experiments into randomized trials testing OT in patients with stroke, myocardial infarction and cardiac arrest**<br>Green, yellow, red colour indicates that prerequisites for successful OT were met, only partly addressed, not fulfilled. ☾ see respective study protocol, * IVT leads to recanalization in only 40 to 50 % compared to > 80 % with EVT or PCI [104], [110], ** Long therapeutic windows renders OT start before reperfusion unlikely, $ estimated value based on median oxygen exposure (2258 L) during first 12 hours divided by median flow-rate (6 L/min), see results, study population [111]<br><br>**EVT, endovascular mechanical thrombectomy; FiO_2, fraction of inspired oxygen; IQR, interquartile range; IVT, intravenous thrombolysis; OT, oxygen therapy; PCI, percutaneous coronary intervention; ROSC, return of spontaneous circulation; SD, standard deviation**	
**Earlier determination of the primary end point (24 hours)**<br>*Patients included in the PROOF trial will have suffered an*	**Earlier determination of the primary end point (24 hours)**<br>*Patients included in the PROOF trial will have suffered an ischemic*	

Previous and new wording in track change modus	New wording	Comments/ reasons for substantial amendment
ischemic stroke through a proximal vessel occlusion and are thus greatly affected (see above). Due to their old age, many patients will have stated in their living wills or to their next of kin that they do not wish to become in need of care as a result of a severe disability. In the event of unsuccessful acute treatment, therapy limitation in the best interest of the patient is common. However, the decision to limit therapy is made at the earliest after the assessment of the final infarct using cerebral imaging at 24 hours. Instead of the 3-month outcome preferably used in phase III stroke trials, in PROOF – a phase II proof-of-concept study – we will determine the primary endpoint after 24 hours. As a result, all patients will remain analyzable and therapy-independent distortion of study results due to decisions regarding (non-stratifiable) therapy limitations or withdrawal of care and their dissimilar distribution to each study arm, which became the downfall of the NCT00414726 trail by Singhal and colleagues, will be avoided. It is for exactly this reason that the German Society for neuro-intensive and emergency medicine (DGNI) also recently critically discussed the suitability of mortality as a measure of quality in neuro-intensive treatment, including stroke care (see Appendix 2: Sterblichkeitsrate – Qualitätsindikator in der NeuroIntensivmedizin?).	*stroke through a proximal vessel occlusion and are thus greatly affected (see above). Due to their old age, many patients will have stated in their living wills or to their next of kin that they do not wish to become in need of care as a result of a severe disability. In the event of unsuccessful acute treatment, therapy limitation in the best interest of the patient is common. However, the decision to limit therapy is made at the earliest after the assessment of the final infarct using cerebral imaging at 24 hours. Instead of the 3-month outcome preferably used in phase III stroke trials, in PROOF – a phase II proof-of-concept study – we will determine the primary endpoint after 24 hours. As a result, all patients will remain analyzable and therapy-independent distortion of study results due to decisions regarding (non-stratifiable) therapy limitations or withdrawal of care and their dissimilar distribution to each study arm, which became the downfall of the NCT00414726 trail by Singhal and colleagues, will be avoided. It is for exactly this reason that the German Society for neuro-intensive and emergency medicine (DGNI) also recently critically discussed the suitability of mortality as a measure of quality in neuro-intensive treatment, including stroke care (see Appendix 2: Sterblichkeitsrate – Qualitätsindikator in der NeuroIntensivmedizin?).*	
**Imaging and intra-individually controlled primary end point**<br>Patients with acute stroke receive cerebral imaging (either CT or MRI) as a part of clinical routine both when they are admitted and 24 hours afterwards; on admission including vascular imaging and, in case of a proximal vessel occlusion, including perfusion imaging in order to evaluate salvageable brain tissue and to plan reperfusion treatment. These are optimal conditions for PROOF: without additional effort and without delay in the extremely time-critical	**Imaging and intra-individually controlled primary end point**<br>Patients with acute stroke receive cerebral imaging (either CT or MRI) as a part of clinical routine both when they are admitted and 24 hours afterwards; on admission including vascular imaging and, in case of a proximal vessel occlusion, including perfusion imaging in order to evaluate salvageable brain tissue and to plan reperfusion treatment. These are optimal conditions for PROOF: without additional effort and without delay in the extremely time-critical	

Previous and new wording in track change modus	New wording	Comments/ reasons for substantial amendment
acute phase of stroke care the possibility to assess an intra-individually controlled (state of the art) primary imaging end point, the infarct growth from 0 to 24 hours, arises. In contrast to the 24-hour infarct volume, the infarct growth exactly corresponds to the brain tissue, which was initially threatened by decline in an individual patient but was enabled to survive through the acute therapy including study treatment [112]. In contrast to an early clinical end point, such as the 24-hour NIHSS score, infarct growth is not threatened through study treatment independent variables, such as anesthesia hangover (not uncommon after endovascular intervention) or aspiration pneumonia.<br>Therefore, PROOF uses all available possibilities to be able to prove a group effect in the smallest possible number of cases. Moreover, every individual study patient benefit from the use of MRI in PROOF in place of routine 24-hour CT through lack exposure and diagnostic superiority (with the potential for individual changes to therapy); MRI scans are only avoided in clinical routine for financial reasons.	acute phase of stroke care the possibility to assess an intra-individually controlled (state of the art) primary imaging end point, the infarct growth from 0 to 24 hours, arises. In contrast to the 24-hour infarct volume, the infarct growth exactly corresponds to the brain tissue, which was initially threatened by decline in an individual patient but was enabled to survive through the acute therapy including study treatment [112]. In contrast to an early clinical end point, such as the 24-hour NIHSS score, infarct growth is not threatened through study treatment independent variables, such as anesthesia hangover (not uncommon after endovascular intervention) or aspiration pneumonia.<br>Therefore, PROOF uses all available possibilities to be able to prove a group effect in the smallest possible number of cases. Moreover, every individual study patient benefit from the use of MRI in PROOF in place of routine 24-hour CT through lack exposure and diagnostic superiority (with the potential for individual changes to therapy); MRI scans are only avoided in clinical routine for financial reasons.	
**Rapid start of oxygen therapy within the hospital environment**<br>Fig. 2 and Fig. 3 suggest a pre-hospital start of oxygen therapy at the earliest possible time point. Even though this may seem safe [113], in the PROOF trial we begin oxygen therapy in the hospital environment as soon as possible after cerebral imaging and at the latest three hours after onset of symptoms: continuous clinical and technical monitoring by an experienced expert team of stroke specialists within the hospital environment guarantees maximum patient safety. Additionally, only cerebral imaging will allow the exclusion of patients who may not benefit from NBHO [102, 114]: stroke patients with cerebral hemorrhage, permanent ischemia, or no salvageable penumbra. As these constitute > 70% of stroke patients, the oxygen effect would (for a phase-two study) be hopelessly diluted and the sample size necessary for its proof enormous. A pre-hospitalization start of therapy should	**Rapid start of oxygen therapy within the hospital environment**<br>Fig. 2 and Fig. 3 suggest a pre-hospital start of oxygen therapy at the earliest possible time point. Even though this may seem safe [113], in the PROOF trial we begin oxygen therapy in the hospital environment as soon as possible after cerebral imaging and at the latest three hours after onset of symptoms: continuous clinical and technical monitoring by an experienced expert team of stroke specialists within the hospital environment guarantees maximum patient safety. Additionally, only cerebral imaging will allow the exclusion of patients who may not benefit from NBHO [102, 114]: stroke patients with cerebral hemorrhage, permanent ischemia, or no salvageable penumbra. As these constitute > 70% of stroke patients, the oxygen effect would (for a phase-two study) be hopelessly diluted and the sample size necessary for its proof enormous. A pre-hospitalization start of therapy should	

Previous and new wording in track change modus	New wording	Comments/ reasons for substantial amendment
consequently only be evaluated (in a phrase III study), when PROOF has brought the proof-of-concept and shown a positive effect of oxygen therapy in the target group of stroke patients, that – corresponding to animal experiments – should benefit: patients with a salvageable penumbra and transient ischemia. As oxygen is available worldwide at a low cost and easy to use, a subsequent trail (or NBHO per se) would even be worthwhile if the group effect was minimalized through dilution due to inclusion of all stroke patients and the necessary sample size or resulting number-needed-to-treat would be enormous.<br>The presence of a "target mismatch" on cerebral imaging prior to inclusion compensates for a – in comparison to the animal experiment – prolonged time window (compare Fig. 2), which, for reasons of practicality cannot be shorter in PROOF. After ascertaining the "target mismatch" on cerebral imaging, oxygen therapy must indeed be started as soon as possible as the penumbra may occasionally shrink rapidly (compare Fig. 3 and see paragraph 4.1. Patient selection in Poli et al. [115]).<br>Due to the inclusion and exclusion criteria (including proximal vessel occlusion, three-hour time window, age ≤ 80 years) patients included in PROOF will likely all be treated with TBY, and oxygen therapy – as required by the study protocol – will be stopped at the end of TBY as the continuation of oxygen therapy beyond reperfusion has not been shown to provide additional benefit (in animal experiments) [53]. Through constant optimization of acute stroke care, imaging-to-reperfusion time today lies under 90 minutes [116] meaning that the duration of oxygen therapy in PROOF – taking into account a short verbal patient information, screening and randomization (approx. 15 minutes) – should only last 75 minutes.<br>With every delay, both the volume of salvageable cerebral tissue (penumbra) and the duration of therapy are critically reduced and thus not only the potential positive effect of oxygen (in individuals), but also the chance of showing it in PROOF (group effect) diminish.<br>Instead of waiting until stroke physicians and neuro-	consequently only be evaluated (in a phrase III study), when PROOF has brought the proof-of-concept and shown a positive effect of oxygen therapy in the target group of stroke patients, that – corresponding to animal experiments – should benefit: patients with a salvageable penumbra and transient ischemia. As oxygen is available worldwide at a low cost and easy to use, a subsequent trail (or NBHO per se) would even be worthwhile if the group effect was minimalized through dilution due to inclusion of all stroke patients and the necessary sample size or resulting number-needed-to-treat would be enormous.<br>The presence of a "target mismatch" on cerebral imaging prior to inclusion compensates for a – in comparison to the animal experiment – prolonged time window (compare Fig. 2), which, for reasons of practicality cannot be shorter in PROOF. After ascertaining the "target mismatch" on cerebral imaging, oxygen therapy must indeed be started as soon as possible as the penumbra may occasionally shrink rapidly (compare Fig. 3 and see paragraph 4.1. Patient selection in Poli et al. [115]).<br>Due to the inclusion and exclusion criteria (including proximal vessel occlusion, three-hour time window, age ≤ 80 years) patients included in PROOF will likely all be treated with TBY, and oxygen therapy – as required by the study protocol – will be stopped at the end of TBY as the continuation of oxygen therapy beyond reperfusion has not been shown to provide additional benefit (in animal experiments) [53]. Through constant optimization of acute stroke care, imaging-to-reperfusion time today lies under 90 minutes [116] meaning that the duration of oxygen therapy in PROOF – taking into account a short verbal patient information, screening and randomization (approx. 15 minutes) – should only last 75 minutes.<br>With every delay, both the volume of salvageable cerebral tissue (penumbra) and the duration of therapy are critically reduced and thus not only the potential positive effect of oxygen (in individuals), but also the chance of showing it in PROOF (group effect) diminish.<br>Instead of waiting until stroke physicians and neuro-	

Previous and new wording in track change modus	New wording	Comments/ reasons for substantial amendment
interventionalists have conferred and the indication for TBY has been established (approx. 10 minutes), the PROOF inclusion and exclusion criteria require that likely all patients are treated with TBY and the ischemia – essential for successful oxygen therapy – is not permanent but transient.<br>Following knowledge gathered in animal experiments, the target stroke patient population chosen in PROOF – in contrast to other stroke patients (see above) – benefits from oxygen therapy on a relevant level. The chosen stroke patients are severely affected and they are unable to give consent; without exception either through a speech impairment or neglect to the point of anosognosia; in addition, they suffer from paralysis and the distress of the acute admission. There is insufficient time to implement a legally authorized representation by a next of kin and also the delay caused by detailed written informed consent would critically reduce the positive effect of the oxygen therapy.<br>PROOF has therefore chosen to enroll patients according to the (upcoming) EU regulation 536/2014, which allows inclusion of patients in a clinical trial without prior consent in case several conditions for emergency situations are fulfilled, and which is also in line with local regulations: e.g. §41 Abs. 1 Satz 2 of the German Drug Law (AMG) and §21 Nr. 3 Satz 3 of the Medicinal Devices Act (MPG) (see Section **Fehler! Verweisquelle konnte nicht gefunden werden.** Subject Information and Informed Consent). These laws directly address the type of emergency situation and the time dependent emergency therapy seen and evaluated in PROOF. All patients are severely affected and therefore unable give consent and the high-dose oxygen therapy – as thoroughly shown – can be expected to be of great benefit to each individual patient as long as it is initiated without delay. In the following we will show that no included patient is at risk due to the NBHO used the PROOF trial and therefore all requirements for emergency inclusion according to the (upcoming) EU regulation 536/2014 and local regulations are fulfilled.	interventionalists have conferred and the indication for TBY has been established (approx. 10 minutes), the PROOF inclusion and exclusion criteria require that likely all patients are treated with TBY and the ischemia – essential for successful oxygen therapy – is not permanent but transient.<br>Following knowledge gathered in animal experiments, the target stroke patient population chosen in PROOF – in contrast to other stroke patients (see above) – benefits from oxygen therapy on a relevant level. The chosen stroke patients are severely affected and they are unable to give consent; without exception either through a speech impairment or neglect to the point of anosognosia; in addition, they suffer from paralysis and the distress of the acute admission. There is insufficient time to implement a legally authorized representation by a next of kin and also the delay caused by detailed written informed consent would critically reduce the positive effect of the oxygen therapy.<br>PROOF has therefore chosen to enroll patients according to the (upcoming) EU regulation 536/2014, which allows inclusion of patients in a clinical trial without prior consent in case several conditions for emergency situations are fulfilled, and which is also in line with local regulations: e.g. §41 Abs. 1 Satz 2 of the German Drug Law (AMG) and §21 Nr. 3 Satz 3 of the Medicinal Devices Act (MPG) (see Section **Fehler! Verweisquelle konnte nicht gefunden werden.** Subject Information and Informed Consent). These laws directly address the type of emergency situation and the time dependent emergency therapy seen and evaluated in PROOF. All patients are severely affected and therefore unable give consent and the high-dose oxygen therapy – as thoroughly shown – can be expected to be of great benefit to each individual patient as long as it is initiated without delay. In the following we will show that no included patient is at risk due to the NBHO used the PROOF trial and therefore all requirements for emergency inclusion according to the (upcoming) EU regulation 536/2014 and local regulations are fulfilled.	

Previous and new wording in track change modus	New wording	Comments/ reasons for substantial amendment
The patient organization Stiftung Deutsche Schlaganfall-Hilfe (www.schlanganfall-hilfe.de) and S.A.F.E (www.safestroke.eu/research/) support the PROOF study and previous patient surveys highlight stroke patients' demand to evaluate oxygen therapy regarding its positive effects; 92% of those asked would also accept a deferred consent procedure after emergency patient inclusion in the trial [117].	The patient organization Stiftung Deutsche Schlaganfall-Hilfe (www.schlanganfall-hilfe.de) and S.A.F.E (www.safestroke.eu/research/) support the PROOF study and previous patient surveys highlight stroke patients' demand to evaluate oxygen therapy regarding its positive effects; 92% of those asked would also accept a deferred consent procedure after emergency patient inclusion in the trial [117].	
**Safety of normobaric oxygen therapy planned in PROOF**<br>Due to the PROOF inclusion and exclusion criteria (including proximal vessel occlusion, three-hour time window and age ≤ 80 years) all prospective patients will be treated with a TBY and oxygen therapy will, as stated in the protocol – be discontinued at the end of the TBY procedure; the prospective duration of oxygen therapy will thus be shorter than 75 minutes for all patients. Only in the most unlikely case that a patient suitable for PROOF is not treated with TBY, NBHO will be applied for four hours so that these isolated cases have the chance to benefit through "freezing the penumbra" until spontaneous reperfusion, which occurs in 20-30% and, thus, compared to TBY is less frequently and for the most part delayed. According to the Summary of Product Characteristics (SmPC), reversible (pulmonary) side effects are only to be expected after a therapy duration of > 6 hours and relevant (pulmonary) side effects only after > 12 hours (see Section **Fehler! Verweisquelle konnte nicht gefunden werden.** Risk-benefit Assessment).<br>A meta-analysis published by Chu and colleagues in The Lancet in 2018 has raised significant doubts regarding the safety of NBHO [106]. Altogether, the meta-analysis included 16,037 "acutely ill" adult patients and sweepingly concluded that oxygen therapy would lead to a higher mortality rate and causes damage to the patients, whilst a potpourri of illnesses was included in the analysis in an undifferentiated manner: Trauma, sepsis, stroke (ischemic as well as hemorrhagic), myocardial infarction, cardiac arrest, emergency	**Safety of normobaric oxygen therapy planned in PROOF**<br>Due to the PROOF inclusion and exclusion criteria (including proximal vessel occlusion, three-hour time window and age ≤ 80 years) all prospective patients will be treated with a TBY and oxygen therapy will, as stated in the protocol – be discontinued at the end of the TBY procedure; the prospective duration of oxygen therapy will thus be shorter than 75 minutes for all patients. Only in the most unlikely case that a patient suitable for PROOF is not treated with TBY, NBHO will be applied for four hours so that these isolated cases have the chance to benefit through "freezing the penumbra" until spontaneous reperfusion, which occurs in 20-30% and, thus, compared to TBY is less frequently and for the most part delayed. According to the Summary of Product Characteristics (SmPC), reversible (pulmonary) side effects are only to be expected after a therapy duration of > 6 hours and relevant (pulmonary) side effects only after > 12 hours (see Section **Fehler! Verweisquelle konnte nicht gefunden werden.** Risk-benefit Assessment).<br>A meta-analysis published by Chu and colleagues in The Lancet in 2018 has raised significant doubts regarding the safety of NBHO [106]. Altogether, the meta-analysis included 16,037 "acutely ill" adult patients and sweepingly concluded that oxygen therapy would lead to a higher mortality rate and causes damage to the patients, whilst a potpourri of illnesses was included in the analysis in an undifferentiated manner: Trauma, sepsis, stroke (ischemic as well as hemorrhagic), myocardial infarction, cardiac arrest, emergency	

**Previous and new wording in track change modus**	**New wording**	**Comments/ reasons for substantial amendment**
operations as well as a mixed collective of illnesses requiring intensive care. Grensemann [107] and Stolmeijer [108] indeed follow the same tenor, but do differentiate between illnesses and do not draw definite conclusions regarding stroke patients. Stolmeijer and colleagues even recognize a benefit, albeit only a slight one [108]. None of the reviews, however, recognize the quite different therapy goals of the collectively evaluated studies (i.e. avoidance of hypoxia versus normobaric hyper-oxygenation) and thus the oxygen therapy dosage (FiO_2 0.3 to 1.0) (see Table 5), the therapeutic time window (see Table 6) and the length of use (from 4 to 72 hours) (see Table 6) are neglected. Also, there is no differentiation of patients with transient ischemia receiving reperfusion treatment, permanent ischemia or missing penumbra (see Table 4).	operations as well as a mixed collective of illnesses requiring intensive care. Grensemann [107] and Stolmeijer [108] indeed follow the same tenor, but do differentiate between illnesses and do not draw definite conclusions regarding stroke patients. Stolmeijer and colleagues even recognize a benefit, albeit only a slight one [108]. None of the reviews, however, recognize the quite different therapy goals of the collectively evaluated studies (i.e. avoidance of hypoxia versus normobaric hyper-oxygenation) and thus the oxygen therapy dosage (FiO_2 0.3 to 1.0) (see Table 5), the therapeutic time window (see Table 6) and the length of use (from 4 to 72 hours) (see Table 6) are neglected. Also, there is no differentiation of patients with transient ischemia receiving reperfusion treatment, permanent ischemia or missing penumbra (see Table 4).	
**Table 4 (page 37)**		
**Table 4: Baseline characteristics of randomized trials testing OT in patients with stroke, myocardial infarction and cardiac arrest**<br>☾ studies not considered by Chu et al. [106], ☾☾ randomization based on birth numbers, ✢ reported by Chu et al. [106], ✢✢ terminated after enrolment of 85 (of 480) patients [see ClinicalTrials.gov, NCT00414726], " equals target condition, § extrapolated to number of randomized patients, §§ combined mean and standard deviation of individual study groups, * as per inclusion criterion, ** converted Scandinavian Stroke Scale scores [118]<br>AIS, acute ischemic stroke; AMI, acute myocardial infarction; IAT, intra-arterial thrombolysis; ICH, intracerebral hemorrhage; IQR, interquartile range; IVT, intravenous thrombolysis; LVO, large vessel occlusion; MCA, middle cerebral artery; n/a, not applicable; NR, not reported; NSTEMI, non-ST elevation myocardial infarction; OT, oxygen therapy; RCT, randomized controlled trial; ROSC, return of spontaneous circulation; SAH, subarachnoid hemorrhage; SD, standard deviation; SDH, subdural hematoma; STEMI, ST elevation myocardial infarction; TIA, transient ischemic attack; UK, United Kingdom; USA, United States of America; VF/VT-OHCA, out–of–hospital cardiac arrest with ventricular	**Table 4: Baseline characteristics of randomized trials testing OT in patients with stroke, myocardial infarction and cardiac arrest**<br>☾ studies not considered by Chu et al. [106], ☾☾ randomization based on birth numbers, ✢ reported by Chu et al. [106], ✢✢ terminated after enrolment of 85 (of 480) patients [see ClinicalTrials.gov, NCT00414726], " equals target condition, § extrapolated to number of randomized patients, §§ combined mean and standard deviation of individual study groups, * as per inclusion criterion, ** converted Scandinavian Stroke Scale scores [118]<br>AIS, acute ischemic stroke; AMI, acute myocardial infarction; IAT, intra-arterial thrombolysis; ICH, intracerebral hemorrhage; IQR, interquartile range; IVT, intravenous thrombolysis; LVO, large vessel occlusion; MCA, middle cerebral artery; n/a, not applicable; NR, not reported; NSTEMI, non-ST elevation myocardial infarction; OT, oxygen therapy; RCT, randomized controlled trial; ROSC, return of spontaneous circulation; SAH, subarachnoid hemorrhage; SD, standard deviation; SDH, subdural hematoma; STEMI, ST elevation myocardial infarction; TIA, transient ischemic attack; UK, United Kingdom; USA, United States of America; VF/VT-OHCA, out–of–hospital cardiac arrest with ventricular	

Previous and new wording in track change modus	New wording	Comments/ reasons for substantial amendment
fibrillation/pulseless ventricular tachycardia as initial rhythm	fibrillation/pulseless ventricular tachycardia as initial rhythm	
**Table 5 (page 38)**		
**Table 5: Dosing of Oxygen Therapy (OT) in randomized trials testing OT in patients with stroke, myocardial infarction and cardiac arrest**<br>☾ study not considered by Chu et al. [106], * combined mean and standard deviation of individual study groups, ✢ see respective study protocol, ✢✢ reported by Chu et al. [106]<br>FiO2, fraction of inspired oxygen; FM, face mask; IMV, invasive mechanical ventilation; IQR, interquartile range; n/a, not applicable; NC, nasal cannula; NR, nor reported; OT, oxygen therapy; PaO2, partial pressure of oxygen in the arterial blood; SD, standard deviation; SpO2, saturation of peripheral oxygen; VM, Venturi mask	**Table 5: Dosing of Oxygen Therapy (OT) in randomized trials testing OT in patients with stroke, myocardial infarction and cardiac arrest**<br>☾ study not considered by Chu et al. [106], * combined mean and standard deviation of individual study groups, ✢ see respective study protocol, ✢✢ reported by Chu et al. [106]<br>FiO2, fraction of inspired oxygen; FM, face mask; IMV, invasive mechanical ventilation; IQR, interquartile range; n/a, not applicable; NC, nasal cannula; NR, nor reported; OT, oxygen therapy; PaO2, partial pressure of oxygen in the arterial blood; SD, standard deviation; SpO2, saturation of peripheral oxygen; VM, Venturi mask	
**Table 6 (page 39)**		
**Table 6: Enrolment Procedure, Therapeutic Window and Duration of Oxygen Treatment (OT) in randomized trials testing OT in patients with stroke, myocardial infarction and cardiac arrest**<br>☾ studies not considered by Chu et al. [106], ☾☾ see respective study protocol, ✢ estimated value: mean symptom onset–to–ambulance arrival time (110.9 min) plus 15 min, see table 1 in [119], ✢✢ estimated value: median symptom onset–to–PCI time (150.5 min) minus median duration of pre-PCI OT (79.0 min), see table 2 in [110], § symptom onset–to–PCI, §§ symptom onset–to–ROSC, * symptom onset–to–randomization, ** symptom onset–to–brain imaging, $ estimated value: median supplemental O2 exposure (2258 L) during first 12 hours divided by median flow-rate (6 L/min) in OT group, see results, study population in [111]<br>ED, emergency department; EMS, emergency medical services; IAT, intra-arterial thrombolysis; IC, informed consent; IQR, interquartile range; IVT, intravenous thrombolysis; LAR, legally authorized representative; NR, nor reported; OT, oxygen therapy; PCI, percutaneous coronary intervention; ROSC, return of spontaneous circulation; SD, standard deviation; STEMI, ST elevation myocardial infarction	**Table 6: Enrolment Procedure, Therapeutic Window and Duration of Oxygen Treatment (OT) in randomized trials testing OT in patients with stroke, myocardial infarction and cardiac arrest**<br>☾ studies not considered by Chu et al. [106], ☾☾ see respective study protocol, ✢ estimated value: mean symptom onset–to–ambulance arrival time (110.9 min) plus 15 min, see table 1 in [119], ✢✢ estimated value: median symptom onset–to–PCI time (150.5 min) minus median duration of pre-PCI OT (79.0 min), see table 2 in [110], § symptom onset–to–PCI, §§ symptom onset–to–ROSC, * symptom onset–to–randomization, ** symptom onset–to–brain imaging, $ estimated value: median supplemental O2 exposure (2258 L) during first 12 hours divided by median flow-rate (6 L/min) in OT group, see results, study population in [111]<br>ED, emergency department; EMS, emergency medical services; IAT, intra-arterial thrombolysis; IC, informed consent; IQR, interquartile range; IVT, intravenous thrombolysis; LAR, legally authorized representative; NR, nor reported; OT, oxygen therapy; PCI, percutaneous coronary intervention; ROSC, return of spontaneous circulation; SD, standard deviation; STEMI, ST elevation myocardial infarction	

Previous and new wording in track change modus	New wording	Comments/ reasons for substantial amendment
To address the safety concerns and critically challenge our favorable risk assessment of Section **Fehler! Verweisquelle konnte nicht gefunden werden.** Risk-benefit Assessment, we have performed our own meta-analysis (see Appendix 1), which comprises all randomized controlled trials (RCT) relevant to the PROOF target patient population (i.e. ischemic stroke), that is to say – due to comparable age, accompanying illnesses and vascular risk factors – also RCT including patients with hemorrhagic stroke, myocardial infarction or cardiac arrest.<br>We analyzed mortality (in hospital, on day 30 and last follow-up), disability, infection, pneumonia and the duration of inpatient treatment – like in [106] – related to "any oxygen therapy" on the one hand and focused on high-flow oxygen therapy with "FiO_2 > 0.5" on the other as it approximates oxygen therapy planned within PROOF. Additionally, we evaluated mortality on day 90 as this represents the time of primary survey time point in all recent clinical phase III trials for ischemic stroke, including PROOF. Checking the original publications, we found discrepancies to Chu's meta-analysis; amongst other things, 2,667 patients from [89] were treated with oxygen therapy but left unconsidered in Chu's meta-analysis, all other differences are marked in Tables 4 and 5 as well as 7, 8 and 9.<br>The risk of bias of the underlying RCT was for the most part valued as positive by Chu [106] (see Table 10) but not by us (see Table 11) or Cabello's 2016 Cochrane meta-analysis of oxygen therapy for myocardial infarction [91]. In the latter and through a search of PubMed emerged two RCT of relevance [120, 121].<br>Our analyses consequently comprised the following: (I) the same studies with data as given in Chu et al. (max. of 14,340 patients), (II) data corrected according to original publications (up to 16,718 patients), (III) all RCT (up to 17,066) and (IV) all stroke RCT (up to 9,069 patients). In addition, we have repeated all of our analyses excluding the prematurely terminated NCT00414726 study (https://clinicaltrial.goc/ct2/show/NCT00414726).	To address the safety concerns and critically challenge our favorable risk assessment of Section **Fehler! Verweisquelle konnte nicht gefunden werden.** Risk-benefit Assessment, we have performed our own meta-analysis (see Appendix 1), which comprises all randomized controlled trials (RCT) relevant to the PROOF target patient population (i.e. ischemic stroke), that is to say – due to comparable age, accompanying illnesses and vascular risk factors – also RCT including patients with hemorrhagic stroke, myocardial infarction or cardiac arrest.<br>We analyzed mortality (in hospital, on day 30 and last follow-up), disability, infection, pneumonia and the duration of inpatient treatment – like in [106] – related to "any oxygen therapy" on the one hand and focused on high-flow oxygen therapy with "FiO_2 > 0.5" on the other as it approximates oxygen therapy planned within PROOF. Additionally, we evaluated mortality on day 90 as this represents the time of primary survey time point in all recent clinical phase III trials for ischemic stroke, including PROOF. Checking the original publications, we found discrepancies to Chu's meta-analysis; amongst other things, 2,667 patients from [89] were treated with oxygen therapy but left unconsidered in Chu's meta-analysis, all other differences are marked in Tables 4 and 5 as well as 7, 8 and 9.<br>The risk of bias of the underlying RCT was for the most part valued as positive by Chu [106] (see Table 10) but not by us (see Table 11) or Cabello's 2016 Cochrane meta-analysis of oxygen therapy for myocardial infarction [91]. In the latter and through a search of PubMed emerged two RCT of relevance [120, 121].<br>Our analyses consequently comprised the following: (I) the same studies with data as given in Chu et al. (max. of 14,340 patients), (II) data corrected according to original publications (up to 16,718 patients), (III) all RCT (up to 17,066) and (IV) all stroke RCT (up to 9,069 patients). In addition, we have repeated all of our analyses excluding the prematurely terminated NCT00414726 study (https://clinicaltrial.goc/ct2/show/NCT00414726).	

Previous and new wording in track change modus	New wording	Comments/ reasons for substantial amendment
The increased mortality in in the oxygen therapy arm, which led to the termination of the NCT00414726 study, can be traced back to an imbalance in the distribution of critical base line characteristics (poorer previous health, more comorbidities, more proximal vessel occlusions without reperfusion treatment). The consecutive distortion in mortality is a result of therapy limitations and not the result of oxygen therapy (see above, Appendix 2, Appendix 3, and [76]); 15 out of 24 deaths were due to the therapy limitations; this is a central aspect, which McEvoy disregards in his letter [122]. Singhal conducted the NCT00414726 and advises PROOF as part of the Scientific Advisory Board.<br>None of our analyses from (I) to (IV) for "any oxygen therapy", "FiO_2 > 0.5", "with NCT00414726" and "without NCT00414726" could confirm the detrimental effect regarding mortality (in-hospital, on day 30, day 90 and at last follow-up), disability, infection and pneumonia (see all analyses in Appendix 1) which was shown by Chu's meta-analysis for the mixed collective (that is to say including illnesses and patients that resemble stroke patients neither in pathophysiology nor regarding comorbidities or risk factors); only the length of hospitalization was significantly longer in the intervention arm, incidentally only after correction of the data according to the original publications.<br>An interesting observation is that the AVOID study [110], which is for the most part and, according to the original conclusions also in [106] and [107], interpreted as a study corroborating the harmfulness of oxygen therapy in myocardial infarction, yielded astonishingly positive results regarding mortality.<br>In their review regarding oxygen therapy in animal models of ischemic stroke, Weaver et al. [69] clearly showed that short term oxygen therapy until reperfusion – like it is planned in PROOF – neither lead to an increase in free radicals nor oxidative stress for neuronal tissue, but actually the opposite. The association between hyperoxygenation and aggravation of a hypoxic-ischemic encephalopathy in neonatal asphyxia shown by Kapadia and	The increased mortality in in the oxygen therapy arm, which led to the termination of the NCT00414726 study, can be traced back to an imbalance in the distribution of critical base line characteristics (poorer previous health, more comorbidities, more proximal vessel occlusions without reperfusion treatment). The consecutive distortion in mortality is a result of therapy limitations and not the result of oxygen therapy (see above, Appendix 2, Appendix 3, and [76]); 15 out of 24 deaths were due to the therapy limitations; this is a central aspect, which McEvoy disregards in his letter [122]. Singhal conducted the NCT00414726 and advises PROOF as part of the Scientific Advisory Board.<br>None of our analyses from (I) to (IV) for "any oxygen therapy", "FiO_2 > 0.5", "with NCT00414726" and "without NCT00414726" could confirm the detrimental effect regarding mortality (in-hospital, on day 30, day 90 and at last follow-up), disability, infection and pneumonia (see all analyses in Appendix 1) which was shown by Chu's meta-analysis for the mixed collective (that is to say including illnesses and patients that resemble stroke patients neither in pathophysiology nor regarding comorbidities or risk factors); only the length of hospitalization was significantly longer in the intervention arm, incidentally only after correction of the data according to the original publications.<br>An interesting observation is that the AVOID study [110], which is for the most part and, according to the original conclusions also in [106] and [107], interpreted as a study corroborating the harmfulness of oxygen therapy in myocardial infarction, yielded astonishingly positive results regarding mortality.<br>In their review regarding oxygen therapy in animal models of ischemic stroke, Weaver et al. [69] clearly showed that short term oxygen therapy until reperfusion – like it is planned in PROOF – neither lead to an increase in free radicals nor oxidative stress for neuronal tissue, but actually the opposite. The association between hyperoxygenation and aggravation of a hypoxic-ischemic encephalopathy in neonatal asphyxia shown by Kapadia and	

Previous and new wording in track change modus	New wording	Comments/ reasons for substantial amendment
colleagues [123] in a retrospective study is not to be worried about in PROOF as the brain's vulnerability to oxygen toxicity is – as it was shown not only by Felderhoff-Mueser et al. – tightly/strictly limited to the infantile phase of brain development, i.e. until end of the second week in rodents and the end of the third life year in humans [124], which is far off the age of patients included in PROOF.<br>In conclusion, our new analysis, supports our previous risk benefit analysis (see Section **Fehler! Verweisquelle konnte nicht gefunden werden.** Risk-benefit Assessment). In order to render trial participation in PROOF as safe as possible for study patients we have expanded the DSMB Charter according to the Tübingen University Hospital ethics committee's request to continuously monitor the mortality rate. This means that the DSMB and the sponsor will be simultaneously informed of every death. As the value of the mortality rate a measure of therapy success or harmfulness in neuro-intensive patients including stroke patients nevertheless seems limited (compare Appendix 2), every single death will be examined in detail taking into account patients' wishes for therapy limitations.<br>To enable a "security check" in PROOF even before a decision regarding a possible therapy limitation is made, we have decided to implement an additional frequent (i.e. at every DSMB meeting, see Section **Fehler! Verweisquelle konnte nicht gefunden werden.** Data and Safety Monitoring Board) evaluation of early neurological change (delta NIHSS prior to study intervention to 20 minutes) after the start of oxygen therapy (in the intervention arm) in connection with the primary end point, the infarct growth from prior to study intervention to 24 hours, in the DSMB Charter, so to be able to immediately detect potentially outcome-relevant short-term influences of oxygen therapy on e.g. cerebral perfusion and possibly end the study prematurely. Based on the many animal studies (see [102] for overview) and Singhal's human trial [75], we can, however, assume a stabilization [61, 62, 65-67, 84, 85, 125] or even an	colleagues [123] in a retrospective study is not to be worried about in PROOF as the brain's vulnerability to oxygen toxicity is – as it was shown not only by Felderhoff-Mueser et al. – tightly/strictly limited to the infantile phase of brain development, i.e. until end of the second week in rodents and the end of the third life year in humans [124], which is far off the age of patients included in PROOF.<br>In conclusion, our new analysis, supports our previous risk benefit analysis (see Section **Fehler! Verweisquelle konnte nicht gefunden werden.** Risk-benefit Assessment). In order to render trial participation in PROOF as safe as possible for study patients we have expanded the DSMB Charter according to the Tübingen University Hospital ethics committee's request to continuously monitor the mortality rate. This means that the DSMB and the sponsor will be simultaneously informed of every death. As the value of the mortality rate a measure of therapy success or harmfulness in neuro-intensive patients including stroke patients nevertheless seems limited (compare Appendix 2), every single death will be examined in detail taking into account patients' wishes for therapy limitations.<br>To enable a "security check" in PROOF even before a decision regarding a possible therapy limitation is made, we have decided to implement an additional frequent (i.e. at every DSMB meeting, see Section **Fehler! Verweisquelle konnte nicht gefunden werden.** Data and Safety Monitoring Board) evaluation of early neurological change (delta NIHSS prior to study intervention to 20 minutes) after the start of oxygen therapy (in the intervention arm) in connection with the primary end point, the infarct growth from prior to study intervention to 24 hours, in the DSMB Charter, so to be able to immediately detect potentially outcome-relevant short-term influences of oxygen therapy on e.g. cerebral perfusion and possibly end the study prematurely. Based on the many animal studies (see [102] for overview) and Singhal's human trial [75], we can, however, assume a stabilization [61, 62, 65-67, 84, 85, 125] or even an	

Previous and new wording in track change modus	New wording	Comments/ reasons for substantial amendment
improvement [53, 55, 61, 65, 126] of penumbral perfusion and we can also assume that the (due to the lack of reperfusion treatment only) transient effects – MR-tomographical and clinical stabilization to improvement – shown by Singhal [75] will be rendered permanent through the TBY treatment administered in PROOF, specifically by vessel recanalization and brain tissue reperfusion.	improvement [53, 55, 61, 65, 126] of penumbral perfusion and we can also assume that the (due to the lack of reperfusion treatment only) transient effects – MR-tomographical and clinical stabilization to improvement – shown by Singhal [75] will be rendered permanent through the TBY treatment administered in PROOF, specifically by vessel recanalization and brain tissue reperfusion.	
**Table 7 (page 41)**		
**Table 7: Mortality in randomized trials testing OT in patients with stroke, myocardial infarction and cardiac arrest**<br><br>☾ study not considered by Chu et al. [106]; ☾☾ mortality data not analyzed by Chu et al. [106]; ✢ reported by Chu et al. [106];<br>[1] figure 2 [127]; [2] figure 1 [119]; [3] tables S4 and 4 for mortality at discharge and at six months [110]; [4] table V plus two cases of cardiogenic shock (see results) [128], compare [129]; [5] study results, clinical course of myocardial infarction [130]; [6] results, intervention study [121]; [7] tables I and II [131]; [8] supplementary data, tertiary end points [132]; [9] results, survival plus two cases of sustained return of spontaneous circulation of less than 60 min [133]; [10] Figure 1, and table 2 / figure 4 / figure 2 for mortality at 7 / 30 / 90 days [134]; [11] results, normobaric hyperoxygenation reduced blood occludin and improved neurological functions in patients with acute ischemic stroke [103]; [12] material and methods, results, and table 2 [135]; [13] results, neurological outcome at one week [136] / figure 3 / figure 2 [88] for mortality at 7 / 30 and 90 days / six months; [14] [ClinicalTrials.gov identifier: NCT00414726], [Singhal AB et al., unpublished data]; [15] results [137]; [16] results, effect on physiologic parameters and stroke progression [120]; [17] table 2 [74]; [18] results, and appendix, individual patient data [75] and table 1 [138] for mortality at 7 / 90 days; [19] figure 2 / table 2 for mortality at 7, 30, 90 days / 12 months [73]<br>FU, follow up visit; OT, oxygen therapy	**Table 7: Mortality in randomized trials testing OT in patients with stroke, myocardial infarction and cardiac arrest**<br><br>☾ study not considered by Chu et al. [106]; ☾☾ mortality data not analyzed by Chu et al. [106]; ✢ reported by Chu et al. [106];<br>[1] figure 2 [127]; [2] figure 1 [119]; [3] tables S4 and 4 for mortality at discharge and at six months [110]; [4] table V plus two cases of cardiogenic shock (see results) [128], compare [129]; [5] study results, clinical course of myocardial infarction [130]; [6] results, intervention study [121]; [7] tables I and II [131]; [8] supplementary data, tertiary end points [132]; [9] results, survival plus two cases of sustained return of spontaneous circulation of less than 60 min [133]; [10] Figure 1, and table 2 / figure 4 / figure 2 for mortality at 7 / 30 / 90 days [134]; [11] results, normobaric hyperoxygenation reduced blood occludin and improved neurological functions in patients with acute ischemic stroke [103]; [12] material and methods, results, and table 2 [135]; [13] results, neurological outcome at one week [136] / figure 3 / figure 2 [88] for mortality at 7 / 30 and 90 days / six months; [14] [ClinicalTrials.gov identifier: NCT00414726], [Singhal AB et al., unpublished data]; [15] results [137]; [16] results, effect on physiologic parameters and stroke progression [120]; [17] table 2 [74]; [18] results, and appendix, individual patient data [75] and table 1 [138] for mortality at 7 / 90 days; [19] figure 2 / table 2 for mortality at 7, 30, 90 days / 12 months [73]<br>FU, follow up visit; OT, oxygen therapy	
**Table 8 (page 43)**		
**Table 8: Disability (modified Rankin Scale Score) in randomized trials testing OT in patients with stroke, myocardial infarction and cardiac**	**Table 8: Disability (modified Rankin Scale Score) in randomized trials testing OT in patients with stroke, myocardial infarction and cardiac**	

Previous and new wording in track change modus	New wording	Comments/ reasons for substantial amendment
**arrest**<br><br>✢ reported by Chu et al. [106]; [1] figure 2 for mRS at 90 days [134]; [2] table 2 for mRS at six months [135]; [3] [ClinicalTrials.gov identifier: NCT00414726], [Singhal AB et al., unpublished data]; [4] figure 2 for mRS at six months [88]; [5] table patient data [75], and table 1 [138] for mRS at 90 days<br><br>mRS, modified Rankin Scale score; OT, oxygen therapy	**arrest**<br><br>✢ reported by Chu et al. [106]; [1] figure 2 for mRS at 90 days [134]; [2] table 2 for mRS at six months [135]; [3] [ClinicalTrials.gov identifier: NCT00414726], [Singhal AB et al., unpublished data]; [4] figure 2 for mRS at six months [88]; [5] table patient data [75], and table 1 [138] for mRS at 90 days<br><br>mRS, modified Rankin Scale score; OT, oxygen therapy	
**Table 9 (page 43)**		
**Table 9: Tertiary outcomes in randomized trials testing OT in patients with stroke, myocardial infarction and cardiac arrest**<br><br>☾ length of hospital stay not analyzed by Chu et al. [106]; ☾☾ study not considered by Chu et al. [106]; ✢ reported by Chu et al. [106]; § estimated mean [139]; §§ estimated standard deviation [140]; * combined mean and standard deviation of patients with and without acute myocardial infarction [131]; [1] table 2 [127]; [2] tables 4 and 2 [110]; [3] tables I and II [131]; [4] supplementary data, tertiary end points [132]; [5] table 2 [134]; [6] results, other outcomes at one week [136]; [7] [ClinicalTrials.gov identifier: NCT00414726], [Singhal et al., unpublished data]; [8] table 2 and results [74]; [9] table 1 [73]<br><br>IQR, interquartile range; NR, not reported; OT, oxygen therapy; SAE, serious adverse events; SD, standard deviation	**Table 9: Tertiary outcomes in randomized trials testing OT in patients with stroke, myocardial infarction and cardiac arrest**<br><br>☾ length of hospital stay not analyzed by Chu et al. [106]; ☾☾ study not considered by Chu et al. [106]; ✢ reported by Chu et al. [106]; § estimated mean [139]; §§ estimated standard deviation [140]; * combined mean and standard deviation of patients with and without acute myocardial infarction [131]; [1] table 2 [127]; [2] tables 4 and 2 [110]; [3] tables I and II [131]; [4] supplementary data, tertiary end points [132]; [5] table 2 [134]; [6] results, other outcomes at one week [136]; [7] [ClinicalTrials.gov identifier: NCT00414726], [Singhal et al., unpublished data]; [8] table 2 and results [74]; [9] table 1 [73]<br><br>IQR, interquartile range; NR, not reported; OT, oxygen therapy; SAE, serious adverse events; SD, standard deviation	
**Table 10 (page 44)**		
**Table 10: Risk of bias in randomized trials testing OT in patients with stroke, myocardial infarction and cardiac arrest as assessed by Chu et al. [106]**<br><br>☾ study not considered by Chu et al. [106]; ✢ annotation and – consequently – the overall rating most likely relates to the unpublished NCT00414726 trial; † Data-driven early termination for either apparent benefit or harm; * For mortality outcomes at 30 days and at longest-follow up, there were 168 missing patients (29% of all patients randomized) for post-randomization exclusion, putting these two outcomes in this study at high risk of bias.	**Table 10: Risk of bias in randomized trials testing OT in patients with stroke, myocardial infarction and cardiac arrest as assessed by Chu et al. [106]**<br><br>☾ study not considered by Chu et al. [106]; ✢ annotation and – consequently – the overall rating most likely relates to the unpublished NCT00414726 trial; † Data-driven early termination for either apparent benefit or harm; * For mortality outcomes at 30 days and at longest-follow up, there were 168 missing patients (29% of all patients randomized) for post-randomization exclusion, putting these two outcomes in this study at high risk of bias.	

Previous and new wording in track change modus	New wording	Comments/ reasons for substantial amendment
**Table 11 (page 45)**		
**Table 11: Risk of Bias in randomized trials testing OT in patients with stroke, myocardial infarction and cardiac arrest as assessed by ourselves**<br><br>☾ study not considered by Chu et al. [106]; ⸭ we performed 'best case' and 'worst case' analyses to address this issue; § see [141]; §§ stratification based on: (1) brain imaging modality at baseline (CT vs. MRI), (2) side of LVO (left vs. right), (3) LVO location (terminal ICA vs. proximal M1 vs. distal M1) in conjunction with NIHSS at baseline (6-10 vs. 11-20 vs. ≥ 21), (4) study site<br>COPD, chronic obstructive pulmonary disease; ICA, internal carotid artery; ITT, intention to treat; IVT, intravenous thrombolysis; LVO, large vessel occlusion; MI, myocardial infarction; MRI, magnetic resonance imaging; mRS, modified Rankin Score; n.s., non-significantly; OT, oxygen therapy; RCT, randomized controlled trial; ROSC, return of spontaneous circulation; STEMI, ST elevation myocardial infarction; TIA, transient ischemic attack	**Table 11: Risk of Bias in randomized trials testing OT in patients with stroke, myocardial infarction and cardiac arrest as assessed by ourselves**<br><br>☾ study not considered by Chu et al. [106]; ⸭ we performed 'best case' and 'worst case' analyses to address this issue; § see [141]; §§ stratification based on: (1) brain imaging modality at baseline (CT vs. MRI), (2) side of LVO (left vs. right), (3) LVO location (terminal ICA vs. proximal M1 vs. distal M1) in conjunction with NIHSS at baseline (6-10 vs. 11-20 vs. ≥ 21), (4) study site<br>COPD, chronic obstructive pulmonary disease; ICA, internal carotid artery; ITT, intention to treat; IVT, intravenous thrombolysis; LVO, large vessel occlusion; MI, myocardial infarction; MRI, magnetic resonance imaging; mRS, modified Rankin Score; n.s., non-significantly; OT, oxygen therapy; RCT, randomized controlled trial; ROSC, return of spontaneous circulation; STEMI, ST elevation myocardial infarction; TIA, transient ischemic attack	
**6.2 Trial Duration and Schedule (page 49)**		
FSI (First Subject In) : Q2 2019 ~~2018~~	FSI (First Subject In) : Q2 2019	
**7.4 Inclusion Criteria (page 50)**		
• LVO on CT angiography or MR angiography consistent with clinical signs and symptoms, i.e. either the terminal ICA with involvement of the M1-segment of the MCA/carotid-T, the proximal M1-segment, or the distal M1-segments (distal to perforating branches).<br>Neither TBY nor IVT are a prerequisite for inclusion; patients not receiving TBY or IVT or both can be enrolled. Clinical treatment decisions should not delay study enrollment.	• LVO on CT angiography or MR angiography consistent with clinical signs and symptoms, i.e. either the terminal ICA with involvement of the M1-segment of the MCA/carotid-T, the proximal M1-segment, or the distal M1-segments (distal to perforating branches).<br>Neither TBY nor IVT are a prerequisite for inclusion; patients not receiving TBY or IVT or both can be enrolled. Clinical treatment decisions should not delay study enrollment.	
• Breastfeeding women can participate, but must be instructed to stop breastfeeding after randomization	• Breastfeeding women can participate, but must be instructed to stop breastfeeding after randomization	

Previous and new wording in track change modus	New wording	Comments/ reasons for substantial amendment
**7.5 Exclusion Criteria**		
Respiratory:<br>• Acute viral, bacterial or fungal pneumonia Acute pneumonia, alveolitis or pneumonitis of viral, bacterial, fungal or any other etiology	Respiratory:<br>• Acute pneumonia, alveolitis or pneumonitis of viral, bacterial, fungal or any other etiology	
**10.19 Biomarkers (Pharmacodynamics) Substudy (page 67)**		
**Procedure**: Participants will be asked to consent to donate 4 blood samples to the PROOF blood biobank, which will be centralized at the Neurovascular Research Laboratory at the Fundació Hospital Universitari Vall d'Hebron-Institut de Recerca (VHIR) in Barcelona, Spain. Participation in the Biomarker sub-study is optional and analysis of biomarker samples will only be done if the patient/LAR consents to participate in the substudy (see Sections **Fehler! Verweisquelle konnte nicht gefunden werden.**, **Fehler! Verweisquelle konnte nicht gefunden werden.**, **Fehler! Verweisquelle konnte nicht gefunden werden.**).<br><br>Blood samples collected will be used to measure the concentrations of candidate proteins in the blood that could be influenced by hyperoxygenation therapy. Several biomarkers among oxidative stress, matrix metalloproteinases and inflammatory markers (e.g. IL6, ADAMTS13, SAA, VCAM1, MMP-9, MMP-2, MMP-3, MMP-13, endostatin, MDA) will be evaluated through enzyme-linked immunosorbent assays.	**Procedure**: Participants will be asked to consent to donate 4 blood samples to the PROOF blood biobank, which will be centralized at the Neurovascular Research Laboratory at the Fundació Hospital Universitari Vall d'Hebron-Institut de Recerca (VHIR) in Barcelona, Spain. Participation in the Biomarker substudy is optional and analysis of biomarker samples will only be done if the patient/LAR consents to participate in the substudy (see Sections **Fehler! Verweisquelle konnte nicht gefunden werden.**, **Fehler! Verweisquelle konnte nicht gefunden werden.**, **Fehler! Verweisquelle konnte nicht gefunden werden.**).<br><br>Blood samples collected will be used to measure the concentrations of candidate proteins in the blood that could be influenced by hyperoxygenation therapy. Several biomarkers among oxidative stress, matrix metalloproteinases and inflammatory markers (e.g. IL6, ADAMTS13, SAA, VCAM1, MMP-9, MMP-2, MMP-3, MMP-13, endostatin, MDA) will be evaluated through enzyme-linked immunosorbent assays.	
Total study-dependent blood volume for biomarker analysis: 72 mL;	Total study-dependent blood volume for biomarker analysis: 72 mL;	

Previous and new wording in track change modus	New wording	Comments/ reasons for substantial amendment
biomarker blood is only drawn using a pre-existing vascular access; no venipuncture is required.	biomarker blood is only drawn using a pre-existing vascular access; no venipuncture is required.	
Blood samples will be collected and processed following the PROOF Biomarkers handling and storage instructions, to be provided by VHIR in order to harmonize these procedures among the Participating Sites. Biological samples will be labelled with the PROOF participant ID number at each Participating Site. The Participating Site must store the blood samples in a biological sample freezer at -80°C, until the end of Study recruitment period. After the last visit of their last patient, the Participating Site will send its blood samples to the PROOF blood biobank at VHIR. Thus, results of substudy analyses will become available only after the end of the PROOF main trial and will therefore not influence study treatment overall or on the individual patient level. VHIR will engage a common shipment company to collect and ship the samples to VHIR from each Participating Site. Once the blood samples have arrived at VHIR they will be stored in a freezer for biological samples at -80°C until they are used for determination of blood-based biomarkers.	Blood samples will be collected and processed following the PROOF Biomarkers handling and storage instructions, to be provided by VHIR in order to harmonize these procedures among the Participating Sites. Biological samples will be labelled with the PROOF participant ID number at each Participating Site. The Participating Site must store the blood samples in a biological sample freezer at -80°C, until the end of Study recruitment period. After the last visit of their last patient, the Participating Site will send its blood samples to the PROOF blood biobank at VHIR. Thus, results of substudy analyses will become available only after the end of the PROOF main trial and will therefore not influence study treatment overall or on the individual patient level. VHIR will engage a common shipment company to collect and ship the samples to VHIR from each Participating Site. Once the blood samples have arrived at VHIR they will be stored in a freezer for biological samples at -80°C until they are used for determination of blood-based biomarkers.	
**Data management:** VHIR will create the PROOF biomarker database with all of the results of the blood-based biomarkers determinations related to each PROOF Participant ID number that will be shared with KKS Heidelberg. The anonymized neuroimaging recovery biomarkers and clinical information will be stored by VHIR in order to perform statistical analysis at the end of the study. Procedure and data format shall be given in the data management plan of VHIR.	**Data management:** VHIR will create the PROOF biomarker database with all of the results of the blood-based biomarkers determinations related to each PROOF Participant ID number that will be shared with KKS Heidelberg. The anonymized neuroimaging recovery biomarkers and clinical information will be stored by VHIR in order to perform statistical analysis at the end of the study. Procedure and data format shall be given in the data management plan of VHIR.	
**Biomarker sample ~~Data~~management plan: After analysis,** any	**Biomarker sample management plan: After analysis,** any	

Previous and new wording in track change modus	New wording	Comments/ reasons for substantial amendment
remaining blood samples will be anonymized and kept in the Neurovascular Research Laboratory in VHIR in Barcelona as a collection registered with Instituto de Salud Carlos III, from the Spanish Ministry of Health, to be used in future studies in the line of stroke biomarkers, if consent has been given by the patient/LAR.	remaining blood samples will be anonymized and kept in the Neurovascular Research Laboratory in VHIR in Barcelona as a collection registered with Instituto de Salud Carlos III, from the Spanish Ministry of Health, to be used in future studies in the line of stroke biomarkers, if consent has been given by the patient/LAR.	
**10.21 (Serios) Adverse Events (page 68)**		
Wherever possible, adverse events will be reported on the basis of the Common Terminology Criteria for Adverse Events (CTCAE) v54.0.	Wherever possible, adverse events will be reported on the basis of the Common Terminology Criteria for Adverse Events (CTCAE) v5.0.	
**16.3 Reports**		
After conclusion of the trial (see Section **Fehler! Verweisquelle konnte nicht gefunden werden.** Trial Duration and Schedule), a report (or alternatively the publication) shall be written by the sponsor's delegate, the coordinating investigator and / or principal investigators. The report will include a statistical analysis and an appraisal of the results from a medical viewpoint. It will be based on the items listed in this trial protocol. The KKS Heidelberg will prepare the biometrical part of this report.<br>Within the defined timeframe (e.g. for Germany within one year after completion of the trial (trial end is defined as last subject out, see Section **Fehler! Verweisquelle konnte nicht gefunden werden.** Trial Duration and Schedule)) the competent authorities and the ethics committees will be supplied with this final report or a summary of the final report containing the principle results. Dependent on national regulations the trial report will be published in a clinical trial register via the competent authority. By signing this protocol, the investigators agree to disclose their names/ clinic	After conclusion of the trial (see Section **Fehler! Verweisquelle konnte nicht gefunden werden.** Trial Duration and Schedule), a report (or alternatively the publication) shall be written by the sponsor's delegate, the coordinating investigator and / or principal investigators. The report will include a statistical analysis and an appraisal of the results from a medical viewpoint. It will be based on the items listed in this trial protocol. The KKS Heidelberg will prepare the biometrical part of this report.<br>Within the defined timeframe (e.g. for Germany within one year after completion of the trial (trial end is defined as last subject out, see Section **Fehler! Verweisquelle konnte nicht gefunden werden.** Trial Duration and Schedule)) the competent authorities and the ethics committees will be supplied with this final report or a summary of the final report containing the principle results. Dependent on national regulations the trial report will be published in a clinical trial register via the competent authority. By signing this protocol, the investigators agree to disclose their names/ clinic	

Previous and new wording in track change modus	New wording	Comments/ reasons for substantial amendment
address in the trial report.	address in the trial report.	
**16.6 Information of Patients about Trial Results**		
In addition to any scientific publication, a comprehensible report of the main trial results and, in case of clinical relevance, the results of the bio marker substudy will be provided to all study participants when they become available.	In addition to any scientific publication, a comprehensible report of the main trial results and, in case of clinical relevance, the results of the bio marker substudy will be provided to all study participants when they become available.	
**19 Appendices**		
**19 Appendices**<br><br>*Appendix 1 Results of own meta-analysis of the randomized trials relevant for the potential PROOF study population*<br>*Appendix 2 Statement of the German society for neuro-intensive care and emergency medicine (DGNI) concerning the suitability of mortality as a measure of quality in neuro-intensive treatment, including stroke care*<br>*Appendix 3 Summary of Singhal's Phase IIb clinical trial of NBHO in acute ischemic stroke [ClinicalTrials.gov identifier: NCT00414726], unpublished data*	**19 Appendices**<br><br>***Appendix 1 Results of own meta-analysis of the randomized trials relevant for the potential PROOF study population***<br>***Appendix 2 Statement of the German society for neuro-intensive care and emergency medicine (DGNI) concerning the suitability of mortality as a measure of quality in neuro-intensive treatment, including stroke care***<br>***Appendix 3 Summary of Singhal's Phase IIb clinical trial of NBHO in acute ischemic stroke [ClinicalTrials.gov identifier: NCT00414726], unpublished data***	
