# Supplementary material for: Penumbral Rescue by normobaric O = O administration in patients with ischemic stroke and target mismatch proFile (PROOF): Study protocol of a phase IIb trial
Source: Int J Stroke. 2023 Aug 18;19(1):120–6. doi: 10.1177/17474930231185275 (PMC10759237; doi:10.1177/17474930231185275)
Supplement: sj-pdf-3-wso-10.1177_17474930231185275 – Supplemental material for Penumbral Rescue by normobaric O = O administration in patients with ischemic stroke and target mismatch proFile (PROOF): Study protocol of a phase IIb trial [file sj-pdf-3-wso-10.1177_17474930231185275.pdf]

Appendix 1 Description of the substantial amendment

Clinical Study Protocol "PROOF: Penumbral Rescue by Normobaric O=O Administration in Patients with Ischemic Stroke and Target Mismatch ProFile: A Phase II Proof-of-Concept Trial"
Phase of study: **Phase II – proof-of-concept**
EudraCT No.: **2017-001355-31**
Study Registry Number: **NCT03500939**

The respective changes made to the Protocol Version 1.2/ 14.03.2019 to Version 1.3/ 06.12.2019 are tabulated below.

Previous and new wording in track change modus	New wording	Comments/ reasons for substantial amendment
**Header**		
Version 1.~~2~~3 / ~~14~~06.~~03~~12.2019	Version 1.3 / 06.12.2019	
**ADMINISTRATIVE STRUCTURE**		
**Lead Monitoring**<br>Coordination Centre for Clinical trials (KKS)<br>~~Anna-Lena Gamer~~Dr. Karsten Thelen (lead)<br>Marsilius-Arkaden / Turm West<br>Im Neuenheimer Feld 130.3<br>69120 Heidelberg<br>Germany<br>Phone: 0049 6221 56 ~~34323~~35622<br>Fax: 0049 6221 56 33508<br>Email: ~~anna-lena.gamer~~karsten.thelen@med.uni-heidelberg.de	**Lead Monitoring**<br>Coordination Centre for Clinical trials (KKS)<br>Dr. Karsten Thelen (lead)<br>Marsilius-Arkaden / Turm West<br>Im Neuenheimer Feld 130.3<br>69120 Heidelberg<br>Germany<br>Phone: 0049 6221 56 35622<br>Fax: 0049 6221 56 33508<br>Email: karsten.thelen@med.uni-heidelberg.de	Change of staff
**ADMINISTRATIVE STRUCTURE**		
**Scientific Advisory Board (SAB)**<br>Prof. Dr. Jean-Claude Baron (Chair)<br>1 Rue Cabanis<br>75014, Paris<br>France<br>Phone: 0033 1 45656268<br>email: jean-claude.baron@inserm.fr<br><br>Prof. Dr. Aneesh Singhal (Co-Chair)<br>55 Fruit Street<br>02114, Boston (MA)<br>United States of America<br>Phone: 001 617 726 1728<br>Email: asinghal@mgh.harvard.edu	**Scientific Advisory Board (SAB)**<br>Prof. Dr. Jean-Claude Baron (Chair)<br>1 Rue Cabanis<br>75014, Paris<br>France<br>Phone: 0033 1 45656268<br>email: jean-claude.baron@inserm.fr<br><br>Prof. Dr. Aneesh Singhal (Co-Chair)<br>55 Fruit Street<br>02114, Boston (MA)<br>United States of America<br>Phone: 001 617 726 1728<br>Email: asinghal@mgh.harvard.edu	Appointment as Chair and Co-Chair to scientific Advisory Board
**ADMINISTRATIVE STRUCTURE**		
**Steering Committee (SC)**<br>Eppdata GmbH<br>Dr. ~~Paulo Dellani~~Frosti Palsson, PhD	**Steering Committee (SC)**<br>Eppdata GmbH<br>Dr. Frosti Palsson, PhD	Change of staff and correction of street name

Previous and new wording in track change modus	New wording	Comments/ reasons for substantial amendment
Loекstedter Steindamm 18<br>22529 -Hamburg<br>Germany<br>Phone: 0049 40 7410 – 25960<br>Fax: 0049 40 7410 – 40114<br>Email: f.palsson@eppdata.de<br>~~p.dellani@eppdata.de~~	Lokstedter Steindamm 18<br>22529 Hamburg<br>Germany<br>Phone: 0049 40 7410 – 25960<br>Fax: 0049 40 7410 – 40114<br>Email: f.palsson@eppdata.de	
**ADMINISTRATIVE STRUCTURE**		
**CORE IMAGING LABORATORY**<br>Eppdata GmbH<br>Dr. Frosti Palsson, PhD ~~Paulo Dellani~~<br>~~Christoph-Probst-Weg 1~~Lokstedter Steindamm 18<br>22529~~20251~~ Hamburg<br>Germany<br>Phone: 0049 40 7410 – 25960<br>Fax: 0049 40 7410 – 40114<br>Email: f.palsson@eppdata.de<br>~~p.dellani@eppdata.de~~	**CORE IMAGING LABORATORY**<br>Eppdata GmbH<br>Dr. Frosti Palsson, PhD<br>Lokstedter Steindamm 18<br>22529 Hamburg<br>Germany<br>Phone: 0049 40 7410 – 25960<br>Fax: 0049 40 7410 – 40114<br>Email: f.palsson@eppdata.de	change of staff and correction of core lab address
**ADMINISTRATIVE STRUCTURE**		
**National Coordinators**<br>~~***SWEDEN***~~<br>~~Vastra Gotlands Lans Landsting~~<br>~~Prof. Dr. Turgut Tatlisumak~~<br>~~2321000131, Regionens Hus~~<br>~~Vanersborg 462 80~~<br>~~Email: turgut.tatlisumak@neuro.gu.se~~		Removal of Swedish National Coordinator as Sweden is no longer participating in the PROOF trial.
**1 Protocol Synopsis, Objectives**		
Clinical safety endpoints: all-cause death at V6, and V7; stroke related death at V6, and V7; symptomatic intracranial hemorrhage until V6; vital signs (systolic and diastolic blood pressure, heart and respiratory rate, peripheral capillary oxygen saturation (SpO_2) and end-tidal	Clinical safety endpoints: all-cause death at V6, and V7; stroke related death at V6, and V7; symptomatic intracranial hemorrhage until V6; vital signs (systolic and diastolic blood pressure, heart and respiratory rate, peripheral capillary oxygen saturation (SpO_2) and end-tidal	

Previous and new wording in track change modus	New wording	Comments/ reasons for substantial amendment
carbon dioxide (etCO_2) at V1-V7, with body temperature at V1, V5, and V6; 12-lead electrocardiogram (ECG) at V1, and V5; [...]	carbon dioxide (etCO_2) at V1-V7, with body temperature at V1, V5, and V6; 12-lead electrocardiogram (ECG) at V1, and V5; [...]	
**1 Protocol Synopsis, Objectives**		
Secondary imaging efficacy endpoints: relative changes in ischemic core volume (in %) from baseline to 24 hours; absolute and relative ischemic core change from baseline to 24 hours using either NCCT or DWI-MRI (or CT/MR angiography) for ischemic core estimation at baseline; absolute and relative ischemic core change from baseline to 24 hours using cerebral blood flow (CBF) < 30% for ischemic core estimation at baseline in all patients, independent of imaging modality; penumbral salvage from baseline to 24 hours;_TICI (Thrombolysis in Cerebral Infarction perfusion scale grade) in patients who underwent TBY; revascularization on 24-hour follow-up imaging.	Secondary imaging efficacy endpoints: relative changes in ischemic core volume (in %) from baseline to 24 hours; absolute and relative ischemic core change from baseline to 24 hours using either NCCT or DWI-MRI (or CT/MR angiography) for ischemic core estimation at baseline; absolute and relative ischemic core change from baseline to 24 hours using cerebral blood flow (CBF) < 30% for ischemic core estimation at baseline in all patients, independent of imaging modality; penumbral salvage from baseline to 24 hours;_TICI (Thrombolysis in Cerebral Infarction perfusion scale grade) in patients who underwent TBY; revascularization on 24-hour follow-up imaging.	
**1 Protocol Synopsis, Investigational Medicinal Product**		
IMP: NBHO, i.e. inhalation of 100% oxygen at high flow (≥ 40 L/min) via a sealed non-rebreather face-mask with reservoir, or in case of intubation/ventilation for (study-independent) TBY, ventilation with an inspiratory oxygen fraction (FiO_2) of 1.0. NBHO is started within ~~3~~ 6 hours of stroke symptom onset (witnessed or last seen well) and within 30~~20~~ minutes after end of baseline brain imaging and applied until the end of TBY procedure (defined by removal of guide catheter from sheath) or, in case TBY is not attempted (defined as 'TBY was not attempted or intervention was stopped prior to any penetration or aspiration of the qualifying (i.e. intracranial) LVO'), 4 hours after start of study treatment.	IMP: NBHO, i.e. inhalation of 100% oxygen at high flow (≥ 40 L/min) via a sealed non-rebreather face-mask with reservoir, or in case of intubation/ventilation for (study-independent) TBY, ventilation with an inspiratory oxygen fraction (FiO_2) of 1.0. NBHO is started within 6 hours of stroke symptom onset (witnessed or last seen well) and within 30 minutes after end of baseline brain imaging and applied until the end of TBY procedure (defined by removal of guide catheter from sheath) or, in case TBY is not attempted (defined as 'TBY was not attempted or intervention was stopped prior to any penetration or aspiration of the qualifying (i.e. intracranial) LVO'), 4 hours after start of study treatment.	

Previous and new wording in track change modus	New wording	Comments/ reasons for substantial amendment
**1 Protocol Synopsis, Study population**		
Inclusion Criteria<br>• Age: > 18 ~~to 80~~ years<br>• ~~Clinical signs and symptoms consistent with the diagnosis of an a~~Acute anterior circulation ischemic stroke due to a Large vessel occlusion (LVO) on CT ~~angiography~~ or MR angiography ~~consistent with clinical signs and symptoms~~, i.e. either ~~the~~ **terminal internal carotid artery (ICA)** with ~~involvement of the~~ M1~~-segment of the MCA~~/carotid-T~~-involvement, the~~ **proximal M1~~-segment~~**, ~~or the~~ **distal M1~~-segments~~** (distal to perforating branches), or **M2/3 segment(s)**<br>• **If TBY is likely to be conducted**~~Neither TBY nor IVT are a prerequisite for inclusion; patients not receiving TBY or IVT or both can be enrolled. Clinical treatment decisions should not delay study enrollment.~~<br>• NIHSS score of ≥ 6 at screening<br>• ~~NIHSS item 1a (level of consciousness) of 0 or 1~~<br>• Alberta Stroke Program Early CT score (ASPECTS) of 7-10 on non-contrast CT or 6-10 on diffusion-weighted MRI (DWI-MRI)<br>• CT or MR perfusion (~~preferably~~ whole-brain or~~,~~ minimal coverage ≥ 75 mm) ~~or MR perfusion imaging~~ performed prior to NBHO<br>• NBHO can be initiated within ~~3~~ 6 hours of ~~certain stroke~~ symptom onset (witnessed or last seen well) and within ~~20~~ 30 minutes after last image ~~end~~ of baseline brain imaging ~~(i.e. within 20 minutes after last image)~~<br>• Pre-stroke mRS of 0 or 1<br>• Breastfeeding women ~~can participate, but~~ must ~~be instructed to~~ stop breastfeeding after randomization<br>~~Due to the emergency situation in which patients are enrolled and the presumed safety of the IMP as applied in the PROOF trial (see Section 4.3 Risk-benefit~~	Inclusion Criteria<br>• Age: > 18 years<br>• Acute anterior circulation ischemic stroke due to a Large vessel occlusion (LVO) on CT or MR angiography, i.e. either **terminal internal carotid artery (ICA)** with M1/carotid-T, **proximal M1**, **distal M1** (distal to perforating branches), or **M2/3 segment(s)**<br>• **If TBY is likely to be conducted**<br>• NIHSS score of ≥ 6 at screening<br>• Alberta Stroke Program Early CT score (ASPECTS) of 7-10 on non-contrast CT or 6-10 on diffusion-weighted MRI (DWI-MRI)<br>• CT or MR perfusion (whole-brain or minimal coverage ≥ 75 mm) performed prior to NBHO<br>• NBHO can be initiated within 6 hours of symptom onset (witnessed or last seen well) and within 30 minutes after last image of baseline brain imaging<br>• Pre-stroke mRS of 0 or 1<br>• Breastfeeding women must stop breastfeeding after randomization<br>Own written informed consent is not obtained prior to study inclusion but has to be gained as soon as possible. Patients who are able to give consent will be informed about trial participation orally and may consent to or decline participation. Patients unable to give consent will be enrolled through a deferred consent procedure (see Section **Fehler! Verweisquelle konnte nicht gefunden werden.** Subject Information and Informed Consent)	simplified wording for easy and fast understanding and adaption of longer therapeutic time window to 6 hours enhancing feasibility and potentially enrollment most likely without limiting NBHO efficacy

Previous and new wording in track change modus	New wording	Comments/ reasons for substantial amendment
Assessment), their oOwn written informed consent is not obtained prior to study inclusion but has to be gained as soon as possible. Patients who are able to give consent will be informed about trial participation orally and may consent to or decline participation. Patients unable to give consent will be enrolled through a deferred consent procedure (see Section **Fehler! Verweisquelle konnte nicht gefunden werden.** Subject Information and Informed Consent)		
**1 Protocol Synopsis, Study population, Exclusion criteria**		
Neurological:<br>• TBY procedure initiated (groin puncture) prior to randomization<br>• Rapid major improvement in neurological status to an NIHSS < 6 or evidence of vessel recanalization prior to randomization<br>• Any condition Seizures which at stroke onset if it makes the diagnosis of stroke doubtful and precludes obtaining an accurate baseline NIHSS or outcome assessment (e.g. seizures, dementia, psychiatric or neuromuscular disease)<br>• Acute neurological symptoms related to other pathology than ischemic stroke<br>• Evidence of iIntracranial hemorrhage (except of cerebral microbleeds), intracranial tumor (except small meningioma), and/or intracranial arteriovenous malformation as confirmed by baseline brain imaging<br>• Intracranial aneurysm or prior stent implantation in the vascular territory (upstream and downstream) affected by qualifying LVO<br>• TBY procedure initiated (groin puncture) prior to randomization<br>• Previously known or CT angiographic / MR angiographic visualization of ipsilateral high-grade	Neurological:<br>• TBY procedure initiated (groin puncture) prior to randomization<br>• Rapid major improvement in neurological status prior to randomization<br>• Any condition which precludes obtaining an accurate baseline NIHSS or outcome assessment (e.g. seizures, dementia, psychiatric or neuromuscular disease)<br>• Intracranial hemorrhage (except of cerebral microbleeds), intracranial tumor (except small meningioma), and/or intracranial arteriovenous malformation<br>• Intracranial aneurysm or prior stent implantation in the vascular territory (upstream and downstream) affected by qualifying LVO<br>• Suspected complete common carotid artery (CCA) occlusion, aortic dissection, cerebral vasculitis, septic embolism, or bacterial endocarditis<br>• Acute bilateral stroke or stroke in multiple vascular territories (except of clinically silent micro-lesions)	simplified wording for easy and fast understanding

Previous and new wording in track change modus	New wording	Comments/ reasons for substantial amendment
~~stenosis, complete cervical carotid occlusion, or flow-limiting carotid dissection~~<br>• Suspected complete common carotid artery (CCA) occlusion, aortic dissection, ~~or~~ cerebral vasculitis, septic embolism, or bacterial endocarditis ~~based on medical history or CT angiography / MR angiography~~<br>• ~~Clinical or imaging evidence of a~~Acute bilateral stroke or stroke in multiple ~~other~~ vascular territories ~~than qualifying LVO~~ (except of clinically silent micro-lesions ~~on DWI-MRI in patients who received MR-based acute brain imaging~~)<br>~~• Significant mass effect with midline shift as confirmed by brain imaging~~<br>~~Any co-existing neurological (especially neuromuscular) disorder~~		
**1 Protocol Synopsis, Study population, Exclusion criteria**		
Respiratory:<br>• Known history of chronic pulmonary disease (e.g. COPD, pulmonary fibrosis, alveolitis or pneumonitis)<br>~~• Any condition leading to hypoxic respiratory drive (e.g. neuromuscular disease)~~<br>• Prior to enrolment, > 2 L/min oxygen required to maintain peripheral oxygen saturation ≥ 95%<br>• Acute respiratory distress that may, in the clinical judgment of the investigator, interfere with the study intervention<br>• Acute pneumonia, alveolitis or pneumonitis of viral, bacterial, fungal or any other etiology<br>• ~~Endotracheal intubation at time of screening or anticipated intubation for other reasons than TBY procedure~~	Respiratory:<br>• Known history of chronic pulmonary disease (e.g. COPD, pulmonary fibrosis, alveolitis or pneumonitis)<br>• Prior to enrolment, > 2 L/min oxygen required to maintain peripheral oxygen saturation ≥ 95%<br>• Acute respiratory distress that may, in the clinical judgment of the investigator, interfere with the study intervention<br>• Acute pneumonia, alveolitis or pneumonitis of viral, bacterial, fungal or any other etiology	simplified wording for easy and fast understanding

Previous and new wording in track change modus	New wording	Comments/ reasons for substantial amendment
**1 Protocol Synopsis, Study population, Exclusion criteria**		
Other:<br>• Clinical suspicion of acute myocardial infarction (e.g. acute ~~pressure or tightness in the~~ chest~~,~~ pain ~~in the chest, back, jaw, and other areas of the upper body that lasts more than a few minutes or that goes away and comes back, shortness of breath~~)<br>• Baseline blood glucose of < 50 mg/dL (2.78 mmol) or > 400 mg/dL (22.20 mmol)<br>• Body temperature ≥ 38.0°C at screening<br>• ~~Presumed septic embolus, or suspicion of bacterial endocarditis~~<br>• History of severe allergy (more than rash) to contrast medium<br>• Current treatment with nitrofurantoin or amiodaron, paraquat poisoning, or history of treatment with bleomycin<br>• Pregnancy at screening, to be excluded (β-HCG in serum or urine) in all women ≤ 55 years except if surgically sterile; in women > 55 years pregnancy must be excluded only in case of increased probability e.g. due to in-vitro fertilization<br>• Any co-existing or terminal disease (except qualifying stroke) with anticipated life expectancy of less than 6 months<br>• Any pre-existing condition that may, in the clinical judgment of the investigator, not allow safe participation in the study (e.g. alcohol or substance abuse, co-existing disease) ~~or would complicate assessment of outcomes (e.g. dementia, psychiatric disease) or would confound the neurological or functional evaluations (e.g. dementia)~~<br>• Participation in another interventional (drug or device) study within the last four weeks	Other:<br>• Clinical suspicion of acute myocardial infarction (e.g. acute chest pain)<br>• Baseline blood glucose of < 50 mg/dL (2.78 mmol) or > 400 mg/dL (22.20 mmol)<br>• Body temperature ≥ 38.0°C at screening<br>• History of severe allergy (more than rash) to contrast medium<br>• Current treatment with nitrofurantoin or amiodaron, paraquat poisoning, or history of treatment with bleomycin<br>• Pregnancy at screening, to be excluded (β-HCG in serum or urine) in all women ≤ 55 years except if surgically sterile; in women > 55 years pregnancy must be excluded only in case of increased probability e.g. due to in-vitro fertilization<br>• Any co-existing or terminal disease (except qualifying stroke) with anticipated life expectancy of less than 6 months<br>• Any pre-existing condition that may, in the clinical judgment of the investigator, not allow safe participation in the study (e.g. alcohol or substance abuse, co-existing disease)<br>• Participation in another interventional (drug or device) study within the last four weeks<br><br>Prior participation in the PROOF trial	simplified wording for easy and fast understanding

Previous and new wording in track change modus	New wording	Comments/ reasons for substantial amendment
Prior participation in the PROOF trial ~~(no subject will be allowed to enroll in this trial more than once).~~		
**1 Protocol Synopsis, Trial duration**		
FSI (first subject in): ~~Q2~~ August 2019	FSI (first subject in): August 2019	
**2 Trial Schedule**		
		Clarification of oxygen administration in the flow chart and revision and insertion of additional foot notes.
**3 Abbreviations**		
~~4.3~~4.4 Data and Safety Monitoring Board (DSMB)	ACS - Acute coronary syndroms<br>CCA - Common carotid artery<br>etCO2 - end-tidal carbon dioxide<br>OT - oxygen therapy<br>PCI - percutaneous coronary intervention<br>STEMI - ST elevation myocardial infarction	Insertion of new abbreviations
**4.3.1 Risk-Benefit Assessment Considering the Most Recent Literature (page 33)**		
This chapter will discuss any ambiguity regarding the risk-benefit assessment and consent modalities of the PROOF study trial considering ~~most~~ recently published review articles including one meta-analysis as well as the most recently presented main results of the **New Zealand Oxygen in Acute Coronary Syndromes Trial (NZOTACS)** [100].	This chapter will discuss any ambiguity regarding the risk-benefit assessment and consent modalities of the PROOF study trial considering recently published review articles including one meta-analysis as well as the most recently presented main results of the **New Zealand Oxygen in Acute Coronary Syndromes Trial (NZOTACS)** [100].	Inclusion of latest research results
**4.3.1 Risk-Benefit Assessment Considering the Most Recent Literature (page 34)**		
The ischemic penumbra can thus be stabilized by breathing almost pure oxygen early on ('freezing the penumbra'), but, in animal models, could ~~an~~ only survive if the blocked vessel ~~is~~ was recanalized within three hours ('nothing can hold its breath forever') (see Fig. 3) [103]. In humans, however, significant penumbral salvage may also possible beyond the three-hour time window as shown in the two recent TBY studies DAWN [104] and DEFUSE-3 [105].	The ischemic penumbra can thus be stabilized by breathing almost pure oxygen early on ('freezing the penumbra'), but, in animal models, could only survive if the blocked vessel was recanalized within three hours ('nothing can hold its breath forever') (see Fig. 3) [103]. In humans, however, significant penumbral salvage may also possible beyond the three-hour time window as shown in	Inclusion of latest research results

Previous and new wording in track change modus	New wording	Comments/ reasons for substantial amendment
	the two recent TBY studies DAWN [104] and DEFUSE-3 [105].	
**4.3.1 Risk-Benefit Assessment Considering the Most Recent Literature (page 37)**		
The PROOF study is the first human NBHO trial to consider all three framework requirements for a successful “freezing of the penumbra” and lasting conservation of positive effects through: (1) early reperfusion (compare Fig. 1 and final infarct versus failed recanalization in Fig. 3): Due to the selected inclusion and exclusion criteria, such as proximal vessel occlusion, a narrow therapeutic time window (six ~~three~~ hours), small infarct core at screening, and enrolment of (older) patients only in the case that TBY is likely to be conducted~~the maximum permitted age of 80 years~~ , all patients included in PROOF will likely be treated by endovascular mechanical thrombectomy (TBY) ± intravenous thrombolysis, in which for > 80% of cases complete brain tissue reperfusion is reached, thus achieving transient ischemia, which is deemed necessary for successful oxygen therapy as concluded from animal experiments [107]. (2) Early start of oxygen therapy: the therapeutic time window of ~~three~~ six hours after symptom onset was chosen for reasons of practicality and is indeed longer than the 30 minute time window proven effective in animal experiments (compare Fig. 2), however, in contrast to animal experiments, a “target mismatch” on cerebral imaging as stipulated in PROOF guarantees the presence of a substantial volume of salvageable penumbra which is threatened to decline (compare rows 3 and 4 in Fig. 3 and the results of the two TBY trials DAWN [104] and DEFUSE-3 [105]). (3) Sufficient oxygen dosage: through the high flow of 40 L/min or more, even patients who are agitated in the emergency situation and therefore breathe faster and deeper receive oxygen at the FiO$_2$ level of over 0.95, as proven successful in animal experiments (see Table 2).	The PROOF study is the first human NBHO trial to consider all three framework requirements for a successful “freezing of the penumbra” and lasting conservation of positive effects through: (1) early reperfusion (compare Fig. 1 and final infarct versus failed recanalization in Fig. 3): Due to the selected inclusion and exclusion criteria, such as proximal vessel occlusion, a narrow therapeutic time window (six hours), small infarct core at screening, and enrolment of (older) patients only in the case that TBY is likely to be conducted, all patients included in PROOF will likely be treated by endovascular mechanical thrombectomy (TBY) ± intravenous thrombolysis, in which for > 80% of cases complete brain tissue reperfusion is reached, thus achieving transient ischemia, which is deemed necessary for successful oxygen therapy as concluded from animal experiments [107]. (2) Early start of oxygen therapy: the therapeutic time window of six hours after symptom onset was chosen for reasons of practicality and is indeed longer than the 30 minute time window proven effective in animal experiments (compare Fig. 2), however, in contrast to animal experiments, a “target mismatch” on cerebral imaging as stipulated in PROOF guarantees the presence of a substantial volume of salvageable penumbra which is threatened to decline (compare rows 3 and 4 in Fig. 3 and the results of the two TBY trials DAWN [104] and DEFUSE-3 [105]). (3) Sufficient oxygen dosage: through the high flow of 40 L/min or more, even patients who are agitated in the emergency situation and therefore breathe faster and deeper receive oxygen at the FiO$_2$ level of over 0.95, as proven successful in animal experiments (see Table 2).	
**4.3.1 Risk-Benefit Assessment Considering the Most Recent Literature (page 39)**		

Previous and new wording in track change modus	New wording	Comments/ reasons for substantial amendment
Due to the inclusion and exclusion criteria (including proximal vessel occlusion, sixthree-hour time window, small infarct core, and likelihood of conduction of age ≤ 80 years TBY) patients included in PROOF will likely all be treated with TBY, and oxygen therapy – as required by the study protocol – will be stopped at the end of TBY as the continuation of oxygen therapy beyond reperfusion has not been shown to provide additional benefit (in animal experiments) [53]	Due to the inclusion and exclusion criteria (including proximal vessel occlusion, six-hour time window, small infarct core, and likelihood of conduction of TBY) patients included in PROOF will likely all be treated with TBY, and oxygen therapy – as required by the study protocol – will be stopped at the end of TBY as the continuation of oxygen therapy beyond reperfusion has not been shown to provide additional benefit (in animal experiments) [53]	
**4.3.1 Risk-Benefit Assessment Considering the Most Recent Literature (page 40)**		
**Safety of normobaric oxygen therapy planned in PROOF** Due to the PROOF inclusion and exclusion criteria (including proximal vessel occlusion, threesix-hour time window, small infarct core, and likelihood of conduction of TBYage ≤ 80 years) nearly all prospective patients will be treated with a TBY and oxygen therapy will, as stated in the protocol – be discontinued at the end of the TBY procedure; the prospective duration of oxygen therapy will thus be shorter than 75 minutes for nearly all patients. Only in the most unlikely case that a patient suitable for PROOF is not treated with TBY, NBHO will be applied for four hours so that these isolated cases have the chance to benefit through “freezing the penumbra” until spontaneous reperfusion, which occurs in 20-30% and, thus, compared to TBY is less frequently and for the most part delayed. According to the Summary of Product Characteristics (SmPC), reversible (pulmonary) side effects are only to be expected after a NBHOtherapy duration of > 6 hours and relevant (pulmonary) side effects only after > 12 hours (see Section **Fehler! Verweisquelle konnte nicht gefunden werden.** Risk-benefit Assessment).	**Safety of normobaric oxygen therapy planned in PROOF** Due to the PROOF inclusion and exclusion criteria (including proximal vessel occlusion, six-hour time window, small infarct core, and likelihood of conduction of TBY) nearly all prospective patients will be treated with a TBY and oxygen therapy will, as stated in the protocol – be discontinued at the end of the TBY procedure; the prospective duration of oxygen therapy will thus be shorter than 75 minutes for nearly all patients. Only in the most unlikely case that a patient suitable for PROOF is not treated with TBY, NBHO will be applied for four hours so that these isolated cases have the chance to benefit through “freezing the penumbra” until spontaneous reperfusion, which occurs in 20-30% and, thus, compared to TBY is less frequently and for the most part delayed. According to the Summary of Product Characteristics (SmPC), reversible (pulmonary) side effects are only to be expected after a NBHO duration of > 6 hours and relevant (pulmonary) side effects only after > 12 hours (see Section **Fehler! Verweisquelle konnte nicht gefunden werden.** Risk-benefit Assessment).	
**4.3.1 Risk-Benefit Assessment Considering the Most Recent Literature (page 45)**		
Only very recently the results of the **New Zealand Oxygen in Acute Coronary Syndromes Trial** (NZOTACS) have been presented at the annual congress of the European	Only very recently the results of the **New Zealand Oxygen in Acute Coronary Syndromes Trial** (NZOTACS) have been presented at the annual congress of the European	

Previous and new wording in track change modus	New wording	Comments/ reasons for substantial amendment
Society of Cardiology (ESC, Aug 29th to Sep 2nd 2019 in Paris, France) [100]. 40,872 patients with suspected ACS were enrolled. 4,159 patients (10%) had the final diagnosis of ST elevation myocardial infarction (STEMI), 10,218 of Non-STEMI, 3,284 of unstable angina, 19,519 had no ACS and 3,692 were not classified. 20,304 were treated with high-flow oxygen. Overall, 30-day mortality was same in the low and the high oxygen groups. 30-day mortality by final diagnosis however, was same for no-ACS and Non-STEMI, but was reduced in STEMI patients who were treated with high-flow oxygen compared to low oxygen (Odds ratio 0.81, 95% confidence interval 0.66-1.00) [100]. These results do first of all confirm the overall safety of NBHO in a (huge) population very similar to PROOF candidates, i.e. patients with a similar condition and similar vascular risk factors. Second, the beneficial NBHO effects observed in NZOTACS in STEMI patients reinforce us in our choice of focusing on TBY candidates with LVO-associated anterior circulation ischemic stroke, i.e. stroke patients very much resembling STEMI patients in whom vessel recanalization and tissue reperfusion is achieved by percutaneous coronary intervention (PCI).	Society of Cardiology (ESC, Aug 29th to Sep 2nd 2019 in Paris, France) [100]. 40,872 patients with suspected ACS were enrolled. 4,159 patients (10%) had the final diagnosis of ST elevation myocardial infarction (STEMI), 10,218 of Non-STEMI, 3,284 of unstable angina, 19,519 had no ACS and 3,692 were not classified. 20,304 were treated with high-flow oxygen. Overall, 30-day mortality was same in the low and the high oxygen groups. 30-day mortality by final diagnosis however, was same for no-ACS and Non-STEMI, but was reduced in STEMI patients who were treated with high-flow oxygen compared to low oxygen (Odds ratio 0.81, 95% confidence interval 0.66-1.00) [100]. These results do first of all confirm the overall safety of NBHO in a (huge) population very similar to PROOF candidates, i.e. patients with a similar condition and similar vascular risk factors. Second, the beneficial NBHO effects observed in NZOTACS in STEMI patients reinforce us in our choice of focusing on TBY candidates with LVO-associated anterior circulation ischemic stroke, i.e. stroke patients very much resembling STEMI patients in whom vessel recanalization and tissue reperfusion is achieved by percutaneous coronary intervention (PCI).	
**4.5 Steering Committee (SC)**		
**Composition:** the SC is chaired by the Coordinator and notably comprises all Work Package (WP) leaders<br>• Holm Graessner (EKUT), leader of WP2 (Coordination and innovation management) (Deputy: Monika Glauch)<br>• Sven Poli (EKUT), Coordinator leader of WP3 (Trial preparation) (Deputy: Florian Härtig)<br>• Johannes Hüsing (UKL HD), responsible biometrician (Deputy: ~~Anja Dietzel~~Maike Nilsson (UKL-HD), leader of WP4 (Performance of the clinical trial))<br>• ~~Paulo Dellani~~Frosti Palsson (Eppdata), leader of WP5 (Brain Imaging and outcome assessments) (Deputy: Jens Fiehler)	**Composition:** the SC is chaired by the Coordinator and notably comprises all Work Package (WP) leaders<br>• Holm Graessner (EKUT), leader of WP2 (Coordination and innovation management) (Deputy: Monika Glauch)<br>• Sven Poli (EKUT), Coordinator leader of WP3 (Trial preparation) (Deputy: Florian Härtig)<br>• Johannes Hüsing (UKL HD), responsible biometrician (Deputy: Maike Nilsson (UKL-HD), leader of WP4 (Performance of the clinical trial))<br>• Frosti Palsson (Eppdata), leader of WP5 (Brain Imaging and outcome assessments) (Deputy: Jens Fiehler)	

Previous and new wording in track change modus	New wording	Comments/ reasons for substantial amendment
• Joan Montaner (VHIR), leader of WP6 (Biomarkers) (Deputy: Alejandro Bustamante)	• Joan Montaner (VHIR), leader of WP6 (Biomarkers) (Deputy: Alejandro Bustamante)	
**5.2 Secondary Objectives and Secondary Endpoints**		
Secondary imaging efficacy endpoints:<br>• relative changes in ischemic core volume (in %) from baseline to 24 hours;<br>• absolute and relative ischemic core change from baseline to 24 hours using either NCCT or DWI-MRI (or CT/MR angiography) for ischemic core estimation at baseline;<br>• absolute and relative ischemic core change from baseline to 24 hours using cerebral blood flow (CBF) < 30% for ischemic core estimation at baseline in all patients, independent of imaging modality;<br>• penumbral salvage from baseline to 24 hours;<br>• TICI (Thrombolysis in Cerebral Infarction perfusion scale grade) in patients who underwent mechanical thrombectomy (TBY);<br>• revascularization on 24-hour follow-up imaging.	Secondary imaging efficacy endpoints:<br>• relative changes in ischemic core volume (in %) from baseline to 24 hours;<br>• absolute and relative ischemic core change from baseline to 24 hours using either NCCT or DWI-MRI (or CT/MR angiography) for ischemic core estimation at baseline;<br>• absolute and relative ischemic core change from baseline to 24 hours using cerebral blood flow (CBF) < 30% for ischemic core estimation at baseline in all patients, independent of imaging modality;<br>• penumbral salvage from baseline to 24 hours;<br>• TICI (Thrombolysis in Cerebral Infarction perfusion scale grade) in patients who underwent mechanical thrombectomy (TBY);<br>• revascularization on 24-hour follow-up imaging.	
**6.1 Trial Design**		
**Intervention arm:** NBHO (i.e. inhalation of 100% oxygen at high flow (≥ 40 L/min) via a sealed non-rebreather face-mask with reservoir, or in case of intubation/ventilation for (study-independent) TBY, ventilation with an inspiratory	**Intervention arm:** NBHO (i.e. inhalation of 100% oxygen at high flow (≥ 40 L/min) via a sealed non-rebreather face-mask with reservoir, or in case of intubation/ventilation for (study-independent) TBY, ventilation with an inspiratory	The longer therapeutic time window of 6 hours enhances feasibility and potentially

Previous and new wording in track change modus	New wording	Comments/ reasons for substantial amendment
oxygen fraction (FiO_2) of 1.0) started within ~~3~~ 6 hours after certain stroke symptom onset (witnessed or last seen well) and within ~~20~~ 30 minutes after end of baseline brain imaging and applied until the end of TBY procedure (defined by removal of guide catheter from sheath) or, in case TBY is not attempted (defined as ‘TBY was not attempted or intervention was stopped prior to any penetration or aspiration of the qualifying (i.e. intracranial) LVO’), 4 hours after start of study treatment.	oxygen fraction (FiO_2) of 1.0) started within 6 hours after certain stroke symptom onset (witnessed or last seen well) and within 30 minutes after end of baseline brain imaging and applied until the end of TBY procedure (defined by removal of guide catheter from sheath) or, in case TBY is not attempted (defined as ‘TBY was not attempted or intervention was stopped prior to any penetration or aspiration of the qualifying (i.e. intracranial) LVO’), 4 hours after start of study treatment.	enrollment most likely without limiting NBHO efficacy
**6.2 Trial Duration and Schedule**		
FSI (first subject in): ~~Q2~~ August 2019	FSI (first subject in): August 2019	
**7.4 Inclusion Criteria**		
• Age: > 18 ~~to 80~~ years	• Age: > 18 years	• Opened for patients > 80 years, because older patients are nowadays also successfully treated with TBY and achieve comparable outcomes.
• ~~Clinical signs and symptoms consistent with the diagnosis of an a~~Acute anterior circulation ischemic stroke due to an LVO on CT ~~angiography~~ or MR angiography~~ consistent with clinical signs and symptoms~~, i.e. either ~~the~~ terminal ICA with ~~involvement of the~~ M1~~-segment of the MCA~~/carotid-T, ~~the~~ proximal M1~~-segment~~, ~~the distal M1-segments (distal to perforating branches), or M2-segment(s) with high likelihood of TBY.or the~~ distal M1~~-segments~~ (distal to perforating branches), or **M2/3 segment(s)**	• Acute anterior circulation ischemic stroke due to an LVO on CT or MR angiography, i.e. either terminal ICA with M1/carotid-T, proximal M1, distal M1 (distal to perforating branches), or **M2/3 segment(s)**	• Simplified wording for easy and fast understanding<br>• Opened for M2/3 segment(s), because more distal LVO are nowadays also successfully treated with TBY and achieve comparable outcomes.
• If TBY is likely to be conducted* (*However, ~~-~~ ~~N~~neither TBY nor IVT are a prerequisite for inclusion; patients not receiving TBY or IVT or both can be*	• If TBY is likely to be conducted* (**However, neither TBY nor IVT are a prerequisite for inclusion; patients not receiving TBY or IVT or both can be enrolled.*	• New inclusion criterion emphasizing the (pathophysiological) need for enrolling TBY candidates for

Previous and new wording in track change modus	New wording	Comments/ reasons for substantial amendment
enrolled. Clinical treatment decisions should not delay study enrollment).	*Clinical treatment decisions should not delay study enrollment*).	achieving transient ischemia and thus, translating NBHO-related stabilization of the penumbra into reduced final infarct volumes (compare trial rationale in protocol section 4.2 and sample size calculation in section 12.1). This inclusion criterion became relevant due to no upper age limit and inclusion of more distal LVO (i.e. M2/3) in the amended protocol version.<br>• Still, neither TBY nor IVT will be a prerequisite for inclusion, i.e. patients not receiving TBY or IVT or both can be enrolled. We decided so, as interdisciplinary clinical treatment decisions inevitably delay study enrollment and thereby critically shorten NBHO duration, which consequently leads to the reduction of NBHO-related group differences and thus reduces power.
• ~~NIHSS item 1a (level of consciousness) of 0 or 1~~		• We deleted this inclusion criterion because on the one hand, unilateral anterior circulation stroke (inclusion criterion) does not provoke decreased level of consciousness.

Previous and new wording in track change modus	New wording	Comments/ reasons for substantial amendment
		• On the other hand, decreased level of consciousness may still result from other causes, independent of the qualifying stroke. Latter are unwanted in PROOF because of confounding effects on clinical outcomes or on initial or follow-up assessment. These unwanted confounding causes, however are already covered by the following exclusion criteria. Thus, no NIHSS item 1a-specific criterion is needed.<br>Compare exclusion criteria:<br>• Any condition which precludes obtaining an accurate baseline NIHSS or outcome assessment (e.g. seizures, dementia, psychiatric or neuromuscular disease)<br>• Acute bilateral stroke or stroke in multiple vascular territories (except of clinically silent micro-lesions)
• Alberta Stroke Program Early CT score (ASPECTS) of 7-10 on NCnon-contrast CT or 6-10 on diffusion-weighted MRI (DWI-MRI)	• ASPECTS of 7-10 on NCCT or 6-10 on DWI-MRI	
• CT or MR perfusion (preferably whole-brain or, minimal coverage ≥ 75 mm) or MR perfusion imaging performed prior to NBHO	• CT or MR perfusion (whole-brain or minimal coverage ≥ 75 mm) prior to NBHO	Simplified wording for easy and fast understanding

Previous and new wording in track change modus	New wording	Comments/ reasons for substantial amendment
• NBHO can be initiated within ~~3~~ 6 hours of ~~certain stroke~~ symptom onset (witnessed or last seen well) and within ~~20~~ 30 minutes after ~~end~~ last image of baseline brain imaging ~~(i.e. within 20 minutes after last image)~~	• NBHO can be initiated within 6 hours of symptom onset (witnessed or last seen well) and within 30 minutes after last image of baseline brain imaging	• Simplified wording for easy and fast understanding<br>• The longer therapeutic time window of 6 hours enhances feasibility and potentially enrollment most likely without limiting NBHO efficacy.
• Breastfeeding women ~~can participate, but~~ must ~~be instructed to~~ stop breastfeeding after randomization	• Breastfeeding women must stop breastfeeding after randomization	Simplified wording for easy and fast understanding
• ~~Due to the emergency situation in which patients are enrolled and the presumed safety of the IMP as applied in the PROOF trial (see Section 4.3 Risk-benefit Assessment), their o~~Own written informed consent is not obtained prior to study inclusion but has to be gained as soon as possible. Patients who are able to give consent will be informed about trial participation orally and may consent to or decline participation. Patients unable to give consent will be enrolled through a deferred consent procedure (see Section **Fehler! Verweisquelle konnte nicht gefunden werden.** Subject Information and Informed Consent)	• Own written informed consent is not obtained prior to study inclusion but has to be gained as soon as possible. Patients who are able to give consent will be informed about trial participation orally and may consent to or decline participation. Patients unable to give consent will be enrolled through a deferred consent procedure (see Section **Fehler! Verweisquelle konnte nicht gefunden werden.** Subject Information and Informed Consent)	Simplified wording for easy and fast understanding
**7.5 Exclusion Criteria, Neurological**		
• TBY procedure initiated (groin puncture)~~attempted~~ prior to randomization	• TBY procedure initiated (groin puncture) prior to randomization	This exclusion criterion remains unchanged but has been moved to first position
• Rapid major improvement in neurological status ~~to an NIHSS < 6 or evidence of vessel recanalization~~ prior to randomization	Rapid major improvement in neurological status prior to randomization	• Simplified wording for easy and fast understanding<br>• We deleted the addition 'to an NIHSS < 6' because it is redundant to the inclusion criterion 'NIHSS ≥ 6'

Previous and new wording in track change modus	New wording	Comments/ reasons for substantial amendment
		• We deleted 'or evidence of vessel recanalization' because it is covered by 'rapid major improvement in neurological status' as its clinical equivalent
• Any condition ~~Seizures at stroke onset if it makes the diagnosis of stroke doubtful and~~which precludes obtaining an accurate baseline NIHSS or outcome assessment (e.g. seizures, dementia, psychiatric or neuromuscular disease)<br>~~• Acute neurological symptoms related to other pathology than ischemic stroke~~<br>~~Any co-existing neurological (especially neuromuscular) disorder~~	• Any condition which precludes obtaining an accurate baseline NIHSS or outcome assessment (e.g. seizures, dementia, psychiatric or neuromuscular disease)	We summarized the content of multiple exclusion criteria in a single exclusion criterion with simplified wording in order to avoid redundancy and for easy and fast understanding
• ~~Evidence of i~~Intracranial hemorrhage (except of cerebral microbleeds), intracranial tumor (except small meningioma), and/or intracranial arteriovenous malformation ~~as confirmed by baseline brain imaging~~	• Intracranial hemorrhage (except of cerebral microbleeds), intracranial tumor (except small meningioma), and/or intracranial arteriovenous malformation	Simplified wording for easy and fast understanding
~~• Previously known or CT angiographic / MR angiographic visualization of ipsilateral high-grade stenosis, complete cervical carotid occlusion, or flow-limiting carotid dissection~~<br>• Suspected complete CCA occlusion, aortic dissection, ~~or~~ cerebral vasculitis, septic embolism, or bacterial endocarditis ~~based on medical history or CT angiography / MR angiography~~	• Suspected complete CCA occlusion, aortic dissection, cerebral vasculitis, septic embolism, or bacterial endocarditis	• We summarized the content of multiple exclusion criteria in a single exclusion criterion with simplified wording for easy and fast understanding<br>• Patients with ipsilateral high-grade stenosis of the ICA are no longer excluded from study participation, because patients with tandem stenosis are nowadays also successfully treated with TBY and achieve comparable outcomes independent of the etiology of the cervical ICA

Previous and new wording in track change modus	New wording	Comments/ reasons for substantial amendment
		lesion (atherosclerosis vs. dissection) (e.g. [4, 5])
• ~~Clinical or imaging evidence of a~~Acute bilateral stroke or stroke in ~~other~~ multiple vascular territories ~~than qualifying LVO~~ (except of clinically silent micro-lesions ~~on DWI-MRI in patients who received MR-based acute brain imaging~~)	• Acute bilateral stroke or stroke in multiple vascular territories (except of clinically silent micro-lesions)	Simplified wording for easy and fast understanding
~~Significant mass effect with midline shift as confirmed by brain imaging~~		We deleted this exclusion criterion, because 'significant mass effect with midline shift' is a condition only seen in subacute stroke, which is not compatible with the inclusion time window of '6 hours'
**7.5 Exclusion Criteria, Respiratory**		
• ~~Any condition leading to hypoxic respiratory drive (e.g. neuromuscular disease)~~		• We deleted this exclusion criterion because only three conditions are likely to underly hypoxic respiratory drive, i.e. chronic pulmonary disease (e.g. COPD), neuromuscular disease and alcohol or substance abuse. All these conditions are already covered by the following three other exclusion criteria:<br>• Any condition which precludes obtaining an accurate baseline NIHSS or outcome assessment (e.g. seizures, dementia, psychiatric or neuromuscular disease)

Previous and new wording in track change modus	New wording	Comments/ reasons for substantial amendment
		• Known history of chronic pulmonary disease (e.g. COPD, pulmonary fibrosis, alveolitis or pneumonitis)<br>• Any pre-existing condition that may, in the clinical judgment of the investigator, not allow safe participation in the study (e.g. alcohol or substance abuse, co-existing disease)
~~Endotracheal intubation at time of screening or anticipated intubation for other reasons than TBY procedure~~		• We deleted this exclusion criterion, because of redundancy of content with the following exclusion criteria:<br>• Acute respiratory distress that may, in the clinical judgment of the investigator, interfere with the study intervention<br>• Any condition which precludes obtaining an accurate baseline NIHSS or outcome assessment (e.g. seizures, dementia, psychiatric or neuromuscular disease)<br>• Acute bilateral stroke or stroke in multiple vascular territories (except of clinically silent micro-lesions)
**7.5 Exclusion Criteria, <u>Other</u>**		
• Clinical suspicion of acute myocardial infarction (e.g. ~~pressure or tightness in the~~<u>acute</u> chest~~,~~ pain ~~in the chest, back, jaw, and other areas of the upper~~	• Clinical suspicion of acute myocardial infarction (e.g. acute chest pain)	Simplified wording for easy and fast understanding

Previous and new wording in track change modus	New wording	Comments/ reasons for substantial amendment
~~body that lasts more than a few minutes or that goes away and comes back, shortness of breath~~)		
• ~~Presumed septic embolus, or suspicion of bacterial endocarditis~~<br>• Any pre-existing condition that may, in the clinical judgment of the investigator, not allow safe participation in the study (e.g. alcohol or substance abuse, co-existing disease) ~~or would complicate assessment of outcomes (e.g. dementia, psychiatric disease) or would confound the neurological or functional evaluations (e.g. dementia)~~		We deleted this exclusion criteria because of redundancy or integration of content in other exclusion criteria.
• Prior participation in the PROOF trial ~~(no subject will be allowed to enroll in this trial more than once).~~	• Prior participation in the PROOF trial ~~(no subject will be allowed to enroll in this trial more than once).~~	Simplified wording for easy and fast understanding
**7.6 Pre-specified Measures in Case of Slow Recruitment**		
To ensure enrolment, in the amended protocol version, we widened the therapeutic time window for NBHO to six hours, skipped the upper age limit, and allowed more distal and also tandem arterial occlusions in case the PROOF candidate is likely to receive TBY treatment. ~~in~~ In case of continued slow recruitment, the following inclusion criteria may also be adapted: (1) ~~upper age limit may be increased to 85 years or skipped, (2)~~ pre-stroke mRS may include 0-2, and (~~3~~2) ASPECTS may be opened for e.g. 6-10 on non-contrast CT or 5-10 on DWI.	To ensure enrolment, in the amended protocol version, we widened the therapeutic time window for NBHO to six hours, skipped the upper age limit, and allowed more distal and also tandem arterial occlusions in case the PROOF candidate is likely to receive TBY treatment. In case of continued slow recruitment, the following inclusion criteria may also be adapted: (1) pre-stroke mRS may include 0-2, and (2) ASPECTS may be opened for e.g. 6-10 on non-contrast CT or 5-10 on DWI.	
**8.4.2 Dosage Schedule**		
**Intervention arm:** NBHO (i.e. inhalation of 100% oxygen at high flow (≥ 40 L/min) via a sealed non-rebreather face-mask with reservoir, or in case of intubation/ventilation for (study-independent) TBY, ventilation with an inspiratory oxygen fraction (FiO_2) of 1.0) started within ~~3~~ 6 hours after certain stroke symptom onset (witnessed or last seen well) and within ~~20~~ 30 minutes after end of baseline brain imaging and applied until the end of TBY procedure (defined by removal of guide catheter from sheath) or, in	**Intervention arm:** NBHO (i.e. inhalation of 100% oxygen at high flow (≥ 40 L/min) via a sealed non-rebreather face-mask with reservoir, or in case of intubation/ventilation for (study-independent) TBY, ventilation with an inspiratory oxygen fraction (FiO_2) of 1.0) started within 6 hours after certain stroke symptom onset (witnessed or last seen well) and within 30 minutes after end of baseline brain imaging and applied until the end of TBY procedure (defined by removal of guide catheter from sheath) or, in case TBY is	• The longer therapeutic time window of 6 hours enhances feasibility and potentially enrollment most likely without limiting NBHO efficacy.<br>• The definition of “TBY not attempted” is now clarified.

Previous and new wording in track change modus	New wording	Comments/ reasons for substantial amendment
case TBY is not attempted (defined as 'TBY was not attempted or intervention was stopped prior to any penetration or aspiration of the qualifying (i.e. intracranial) LVO'), 4 hours after start of study treatment.	not attempted (defined as 'TBY was not attempted or intervention was stopped prior to any penetration or aspiration of the qualifying (i.e. intracranial) LVO'), 4 hours after start of study treatment.	
**8.4.3 Compliance**		
Compliance will be recorded by the treating investigator. ~~This includes the documentation of vital signs including SpO_2 every 15 minutes (±5) for the first 6 hours, then hourly (±15 minutes) until end of hour 24.~~ Oxygen administration (reason, flow-rate or FiO_2 in ventilated patients, and mask-type/method) will be continuously recorded until end of hour 6, i.e. every change of oxygen administration must be documented. Additionally, SpO_2, and – if available – $etCO_2$ must be documented at each time point of change of oxygen administration. Then, oxygen administration will be recorded hourly (±15 minutes) – together with vital signs – until end of hour 24.~~O_2 administration (including reason, flow-rate and mask-type) will be documented at the same time points.~~ The results will be systematically documented in the patient's medical record and in the eCRF.	Compliance will be recorded by the treating investigator. Oxygen administration (reason, flow-rate or FiO_2 in ventilated patients, and mask-type/method) will be continuously recorded until end of hour 6, i.e. every change of oxygen administration must be documented. Additionally, SpO_2, and – if available – $etCO_2$ must be documented at each time point of change of oxygen administration. Then, oxygen administration will be recorded hourly (±15 minutes) – together with vital signs – until end of hour 24. The results will be systematically documented in the patient's medical record and in the eCRF.	
The following periods and doses will count as protocol violations and lead to exclusion from per-protocol analyses in the NBHO group if administered until the end of TBY procedure (defined by removal of guide catheter from sheath) or, in case TBY is not attempted (defined as 'TBY was not attempted or intervention was stopped prior to any penetration or aspiration of the qualifying (i.e. intracranial) LVO'), during the first 4 hours after start of study treatment:<br><br>Non-rebreather face-mask with reservoir:<br>• > 15-30 L/min for more than one hour<br>• > 10-15 L/min for more than 30 minutes<br>• ~~<~~ ≤ 10 L/min for more than 15 minutes	The following periods and doses will count as protocol violations and lead to exclusion from per-protocol analyses in the NBHO group if administered until the end of TBY procedure (defined by removal of guide catheter from sheath) or, in case TBY is not attempted (defined as 'TBY was not attempted or intervention was stopped prior to any penetration or aspiration of the qualifying (i.e. intracranial) LVO'), during the first 4 hours after start of study treatment:<br><br>Non-rebreather face-mask with reservoir:<br>• > 15-30 L/min for more than one hour<br>• > 10-15 L/min for more than 30 minutes<br>• ≤ 10 L/min for more than 15 minutes	

**Previous and new wording in track change modus**	**New wording**	**Comments/ reasons for substantial amendment**
Simple face-mask (without reservoir) or (partial) rebreather face-mask with reservoir:<br>• > 20 L/min for more than one hour<br>• > 10-20 L/min for more than 30 minutes<br>• < ≤ 10 L/min for more than 15 minutes	Simple face-mask (without reservoir) or (partial) rebreather face-mask with reservoir:<br>• > 20 L/min for more than one hour<br>• > 10-20 L/min for more than 30 minutes<br>• ≤ 10 L/min for more than 15 minutes	
Additionally, prolongation of NBHO for more than 1 hour after end of TBY procedure (defined by removal of guide catheter from sheath) or, in case TBY is not attempted (defined as 'TBY was not attempted or intervention stopped prior to any penetration or aspiration of the qualifying (i.e. intracranial) LVO'), NBHO administration time in total for more than 5 hours, will equally count as protocol violation and lead to exclusion from per-protocol analyses in the NBHO group.<br><br>Conclusion: The per-protocol definition of NBHO is thus the use of either > 30 L/min via non-rebreather face-mask with reservoir or – in case of intubation – an FiO_2 of > 0.8 until either the end of the TBY procedure plus max. 1 hour or, in case TBY is not attempted (defined as 'TBY was not attempted or intervention was stopped prior to any penetration or aspiration of the qualifying (i.e. intracranial) LVO'), for a maximal duration of 5 hours.	Additionally, prolongation of NBHO for more than 1 hour after end of TBY procedure (defined by removal of guide catheter from sheath) or, in case TBY is not attempted (defined as 'TBY was not attempted or intervention stopped prior to any penetration or aspiration of the qualifying (i.e. intracranial) LVO'), NBHO administration time in total for more than 5 hours, will equally count as protocol violation and lead to exclusion from per-protocol analyses in the NBHO group.<br><br>Conclusion: The per-protocol definition of NBHO is thus the use of either > 30 L/min via non-rebreather face-mask with reservoir or – in case of intubation – an FiO_2 of > 0.8 until either the end of the TBY procedure plus max. 1 hour or, in case TBY is not attempted (defined as 'TBY was not attempted or intervention was stopped prior to any penetration or aspiration of the qualifying (i.e. intracranial) LVO'), for a maximal duration of 5 hours.	
In the control group, patients will be excluded from per-protocol analyses if oxygen supplementation until the end of TBY procedure (defined by removal of guide catheter from sheath) or, in case TBY is not attempted (defined as 'TBY was not attempted or intervention was stopped prior to any penetration or aspiration of the qualifying (i.e. intracranial) LVO'), during the first 4 hours after randomization is applied at the following a flow-rates of ≥ 4 L/min (any device) or – in case of intubation – FiO_2 : is ≥ 0.4 for more than one hour.	In the control group, patients will be excluded from per-protocol analyses if oxygen supplementation until the end of TBY procedure (defined by removal of guide catheter from sheath) or, in case TBY is not attempted (defined as 'TBY was not attempted or intervention was stopped prior to any penetration or aspiration of the qualifying (i.e. intracranial) LVO'), during the first 4 hours after randomization is applied at the following flow-rates or – in case of intubation – FiO_2:	

Previous and new wording in track change modus	New wording	Comments/ reasons for substantial amendment
Any face-mask or nasal cannula<br>• ≥ 4 to < 6 L/min for more than one hour<br>• ≥ 6 to < 8 L/min for more than 30 minutes<br>• ≥ 8 L/min for more than 15 minutes<br><br>in case of intubated patients:<br>• 0.4 ≤ FiO_2 < 0.6 for more than one hour<br>• 0.6 ≤ FiO_2 < 0.8 for more than 30 minutes<br>• 0.8 ≤ FiO_2 ≤ 1.0 for more than 15 minutes	Any face-mask or nasal cannula<br>• ≥ 4 to < 6 L/min for more than one hour<br>• ≥ 6 to < 8 L/min for more than 30 minutes<br>• ≥ 8 L/min for more than 15 minutes<br><br>in case of intubated patients:<br>• 0.4 ≤ FiO_2 < 0.6 for more than one hour<br>• 0.6 ≤ FiO_2 < 0.8 for more than 30 minutes<br>• 0.8 ≤ FiO_2 ≤ 1.0 for more than 15 minutes	
**8.5.1 Randomization method**		
Minimization [149] will be used to consider several strata when allocating treatment. In 10 per cent randomly chosen cases, the procedure will pick the treatment not assigned by the algorithm. Variables used in minimization will be:<br><br>• brain imaging modality at baseline (CT vs. MRI)<br>• side of large vessel occlusion (LVO) (left vs. right)<br>• intracranial LVO location (terminal internal carotid artery (ICA) with involvement of the M1-segment of the middle cerebral artery (MCA)/carotid-T vs. proximal M1-segment vs. distal M1-segment (distal of perforating branches) vs. M2/3-segment(s)<br>• NIHSS at baseline: 6-10, 11-20, 21 and more. LVO location and NIHSS will be used in conjunction for the algorithm, i.e. balance will be aimed for in every one of the nine NIHSS/LVO locations.<br>• Time window 0-3 hours vs. > 3-6 hours<br>• study site	Minimization [149] will be used to consider several strata when allocating treatment. In 10 per cent randomly chosen cases, the procedure will pick the treatment not assigned by the algorithm. Variables used in minimization will be:<br><br>• brain imaging modality at baseline (CT vs. MRI)<br>• side of large vessel occlusion (LVO) (left vs. right)<br>• intracranial LVO location (terminal internal carotid artery (ICA) with involvement of the M1-segment of the middle cerebral artery (MCA)/carotid-T vs. proximal M1-segment vs. distal M1-segment (distal of perforating branches) vs. M2/3-segment(s)<br>• NIHSS at baseline: 6-10, 11-20, 21 and more. LVO location and NIHSS will be used in conjunction for the algorithm, i.e. balance will be aimed for in every one of the nine NIHSS/LVO locations.<br>• Time window 0-3 hours vs. > 3-6 hours<br>• study site	
**9.1 Pre-Screening**		

Previous and new wording in track change modus	New wording	Comments/ reasons for substantial amendment
The participating study sites will maintain a pre-screening log. All patients with acute anterior circulation stroke due to and LVO that are treated with TBY present at the respective study center but not enrolled into the PROOF study within three hours after symptom onset will be entered anonymously. In addition to a consecutive pre-screening number, age, ASPECTS, and pre-stroke mRS, NIHSS, intracranial LVO location, presence and cause of an extracranial ICA occlusion or high-grade stenosis, and the reason(s) for non-inclusion will be documented. The pre-screening logs of the participating study centers will may be monitored regularly and reviewed by the SC in order to help implement protocol amendments in case of slow recruitment (compare Section **Fehler! Verweisquelle konnte nicht gefunden werden.** Pre-specified Measures in Case of Slow Recruitment). In order to support this monitoring process, the respective study site personnel will transfer pre-screening logs to the eCRF and provide the total absolute number of TBY-treated ischemic stroke patients extracted from the local patient information system or TBY database during site monitoring.	The participating study sites will maintain a pre-screening log. All patients with acute anterior circulation stroke due to LVO that are treated with TBY at the respective study center but not enrolled into the PROOF study will be entered anonymously. In addition to a consecutive pre-screening number, age, ASPECTS, pre-stroke mRS, NIHSS, intracranial LVO location, presence and cause of an extracranial ICA occlusion or high-grade stenosis, and the reason(s) for non-inclusion will be documented. The pre-screening logs of the participating study centers will be monitored regularly and reviewed by the SC in order to help implement protocol amendments in case of slow recruitment (compare Section **Fehler! Verweisquelle konnte nicht gefunden werden.** Pre-specified Measures in Case of Slow Recruitment). In order to support this monitoring process, the respective study site personnel will transfer pre-screening logs to the eCRF and provide the total absolute number of TBY-treated ischemic stroke patients extracted from the local patient information system or TBY database during site monitoring.	
**9.2 Screening Visit**		
For safety reasons, vital signs (incl. systolic and diastolic blood pressure, heart rate and respiratory rate, SpO_2, $etCO_2$, and tympanic temperature), blood samples incl. full blood count (white blood cells, platelet count, erythrocytes, hemoglobin, hematocrit), coagulation (international normalized ratio (INR), activated partial thromboplastin time (aPTT), D-dimers), and blood chemistry (sodium, potassium, creatinine, urea, uric acid, total bilirubin, direct bilirubin, total protein, albumin, C-reactive protein, troponin I or T, brain natriuretic peptide (BNP) or N-terminal prohormone of brain natriuretic peptide (NT-proBNP), creatine kinase (CK), aspartate transaminase (AST), alanine transaminase (ALT), alkaline phosphatase, lactate	For safety reasons, vital signs (incl. systolic and diastolic blood pressure, heart rate and respiratory rate, SpO_2, $etCO_2$, and tympanic temperature), blood samples incl. full blood count (white blood cells, platelet count, erythrocytes, hemoglobin, hematocrit), coagulation (international normalized ratio (INR), activated partial thromboplastin time (aPTT), D-dimers), and blood chemistry (sodium, potassium, creatinine, urea, uric acid, total bilirubin, direct bilirubin, total protein, albumin, C-reactive protein, troponin I or T, brain natriuretic peptide (BNP) or N-terminal prohormone of brain natriuretic peptide (NT-proBNP), creatine kinase (CK), aspartate transaminase (AST), alanine transaminase (ALT), alkaline phosphatase, lactate	

Previous and new wording in track change modus	New wording	Comments/ reasons for substantial amendment
dehydrogenase (LDH), gamma-glutamyl transpeptidase (GGT), thyroid-stimulating hormone (TSH), and glucose), and – if applicable (see Section **Fehler! Verweisquelle konnte nicht gefunden werden.** Exclusion criteria) – serum/urine pregnancy test must be assessed.<br>All documented changes of pre-randomization Pre-screening O_2 administration (incl. pre-hospital) is are documented (if possible in 15-minute intervals) incl. reason, flow-rate, mask-type, duration, and SpO_2, and – if available – $etCO_2$.	dehydrogenase (LDH), gamma-glutamyl transpeptidase (GGT), thyroid-stimulating hormone (TSH), and glucose), and – if applicable (see Section **Fehler! Verweisquelle konnte nicht gefunden werden.** Exclusion criteria) – serum/urine pregnancy test must be assessed.<br>All documented changes of pre-randomization O_2 administration (incl. pre-hospital) are documented incl. reason, flow-rate, mask-type, duration, SpO_2, and – if available – $etCO_2$.	
**9.4 V1 – Initiation of study treatment**		
In case the patient is randomized into the intervention arm, NBHO must be started within 3 6 hours after certain stroke symptom onset (witnessed or last seen well) and within 20 30 minutes after end of baseline brain imaging; see Section **Fehler! Verweisquelle konnte nicht gefunden werden.** Dosage Schedule for details.<br>Patients that are randomized to standard treatment will receive either no O_2 supplementation (if $SpO_2 \geq 95\%$) or low-flow O_2 supplementation (if $SpO_2 \leq 94\%$) to maintain $SpO_2 \geq 95\%$ according to ESO guidelines. In case of TBY-related intubation/ventilation, the initial FiO_2 of 0.3 may be gradually increased if $SpO_2 \leq 94\%$; see Section **Fehler! Verweisquelle konnte nicht gefunden werden.** Dosage Schedule for details.<br>A 12-lead electrocardiogram is performed either prior to initiation of study treatment or within one hour after start of NBHO.<br>Vital signs (incl. systolic and diastolic blood pressure, heart rate and respiratory rate, and SpO_2, and – if available – $etCO_2$) as well as reason, flow-rate and method of O_2 supplementation are recorded over the following 24 hours in this manner: every 15 30 ±5 10 minutes for the first six hours after start of NBHO (or randomization in the control arm), then hourly ±15 minutes until 24 hours after start of NBHO (or randomization in the control arm).	In case the patient is randomized into the intervention arm, NBHO must be started within 6 hours after certain stroke symptom onset (witnessed or last seen well) and within 30 minutes after end of baseline brain imaging; see Section **Fehler! Verweisquelle konnte nicht gefunden werden.** Dosage Schedule for details.<br>Patients that are randomized to standard treatment will receive either no O_2 supplementation (if $SpO_2 \geq 95\%$) or low-flow O_2 supplementation (if $SpO_2 \leq 94\%$) to maintain $SpO_2 \geq 95\%$ according to ESO guidelines. In case of TBY-related intubation/ventilation, the initial FiO_2 of 0.3 may be gradually increased if $SpO_2 \leq 94\%$; see Section **Fehler! Verweisquelle konnte nicht gefunden werden.** Dosage Schedule for details.<br>A 12-lead electrocardiogram is performed either prior to initiation of study treatment or within one hour after start of NBHO.<br>Vital signs (incl. systolic and diastolic blood pressure, heart rate and respiratory rate, SpO_2, and – if available – $etCO_2$) are recorded over the following 24 hours in this manner: every 30 ±10 minutes for the first six hours after start of NBHO (or randomization in the control arm), then hourly ±15 minutes until 24 hours after start of NBHO (or randomization in the control arm).	

Previous and new wording in track change modus	New wording	Comments/ reasons for substantial amendment
Oxygen administration (reason, flow-rate or FiO_2 in ventilated patients, and mask-type/method) will be continuously recorded until end of hour 6, i.e. every change of oxygen administration must be documented. Additionally, SpO_2, and – if available – $etCO_2$ must be documented at each time point of change of oxygen administration. Then, oxygen administration will be recorded hourly (±15 minutes) – together with vital signs – until end of hour 24.<br>Concomitant medication, invasive procedures and AE/SAE will be reported continuously.	Oxygen administration (reason, flow-rate or FiO_2 in ventilated patients, and mask-type/method) will be continuously recorded until end of hour 6, i.e. every change of oxygen administration must be documented. Additionally, SpO_2, and – if available – $etCO_2$ must be documented at each time point of change of oxygen administration. Then, oxygen administration will be recorded hourly (±15 minutes) – together with vital signs – until end of hour 24.<br>Concomitant medication, invasive procedures and AE/SAE will be reported continuously.	
**9.5 V2 – 20 ±10 minutes after start of NBHO (or randomization in the control arm)**		
10 to 30 minutes after initiation of NBHO (or randomization in the control arm) and before start of endovascular intervention including sedation and/or endotracheal intubation/mechanical ventilation, physical and neurological examination (incl. NIHSS) are repeated in order to detect early improvement (or deterioration) likely associated with study treatment.<br>If the patient is already sedated or under general anesthesia for the thrombectomy procedure, this must be recorded in the eCRF.<br>Vital signs ~~(incl. systolic and diastolic blood pressure, heart rate and respiratory rate, and SpO_2)~~ as well as ~~reason, flow-rate and method of~~ O_2 supplementation are recorded as indicated in Section **Fehler! Verweisquelle konnte nicht gefunden werden.** Vital signs and Section **Fehler! Verweisquelle konnte nicht gefunden werden.** Oxygen administration.<br>Concomitant medication, invasive procedures and AE/SAE will be reported continuously.	10 to 30 minutes after initiation of NBHO (or randomization in the control arm) and before start of endovascular intervention including sedation and/or endotracheal intubation/mechanical ventilation, physical and neurological examination (incl. NIHSS) are repeated in order to detect early improvement (or deterioration) likely associated with study treatment.<br>If the patient is already sedated or under general anesthesia for the thrombectomy procedure, this must be recorded in the eCRF.<br>Vital signs as well as O_2 supplementation are recorded as indicated in Section **Fehler! Verweisquelle konnte nicht gefunden werden.** Vital signs and Section **Fehler! Verweisquelle konnte nicht gefunden werden.** Oxygen administration.<br>Concomitant medication, invasive procedures and AE/SAE will be reported continuously.	
**9.6 V3 – During TBY**		
Vital signs ~~(incl. systolic and diastolic blood pressure, heart rate and respiratory rate, and SpO_2)~~ as well as ~~reason, flow-rate and method of~~ O_2 supplementation are recorded as	Vital signs as well as O_2 supplementation are recorded as indicated in Section **Fehler! Verweisquelle konnte nicht gefunden werden.** Vital signs and Section **Fehler!**	

Previous and new wording in track change modus	New wording	Comments/ reasons for substantial amendment
indicated in Section **Fehler! Verweisquelle konnte nicht gefunden werden.** Vital signs and Section **Fehler! Verweisquelle konnte nicht gefunden werden.** Oxygen administration.	**Verweisquelle konnte nicht gefunden werden.** Oxygen administration.	
**9.7 V4 – End of study treatment**		
V4 is to be performed at end of study treatment, i.e. after end of TBY procedure (defined by removal of guide catheter from sheath) or, in case TBY is not attempted (defined as 'TBY was not attempted or intervention was stopped prior to any penetration or aspiration of the qualifying (i.e. intracranial) LVO'), 4 hours ±15 minutes after start of study treatment (i.e. start of NBHO or time of randomization for the control group), or in case study treatment is prematurely terminated.<br>Physical and neurological examination (incl. NIHSS) is repeated.<br>Vital signs ~~(incl. systolic and diastolic blood pressure, heart rate and respiratory rate, and SpO_2)~~ as well as ~~reason, flow-rate and method of~~ O_2 supplementation are recorded as indicated in Section **Fehler! Verweisquelle konnte nicht gefunden werden.** Vital signs and Section **Fehler! Verweisquelle konnte nicht gefunden werden.** Oxygen administration.	V4 is to be performed at end of study treatment, i.e. after end of TBY procedure (defined by removal of guide catheter from sheath) or, in case TBY is not attempted (defined as 'TBY was not attempted or intervention was stopped prior to any penetration or aspiration of the qualifying (i.e. intracranial) LVO'), 4 hours ±15 minutes after start of study treatment (i.e. start of NBHO or time of randomization for the control group), or in case study treatment is prematurely terminated.<br>Physical and neurological examination (incl. NIHSS) is repeated.<br>Vital signs as well as O_2 supplementation are recorded as indicated in Section **Fehler! Verweisquelle konnte nicht gefunden werden.** Vital signs and Section **Fehler! Verweisquelle konnte nicht gefunden werden.** Oxygen administration.	
**9.8 V5 – 24 ±6 hours (Day 1) after start of NBHO (or randomization in the control arm)**		
Vital signs ~~(incl. systolic and diastolic blood pressure, heart rate and respiratory rate, and SpO_2)~~ as well as ~~reason, flow-rate and method of~~ O_2 supplementation are recorded as indicated in Section **Fehler! Verweisquelle konnte nicht gefunden werden.** Vital signs and Section **Fehler! Verweisquelle konnte nicht gefunden werden.** Oxygen administration.	Vital signs as well as O_2 supplementation are recorded as indicated in Section **Fehler! Verweisquelle konnte nicht gefunden werden.** Vital signs and Section **Fehler! Verweisquelle konnte nicht gefunden werden.** Oxygen administration.	
**9.9 V6 – Day 5 ±2 after start of NBHO (or randomization in the control arm) or at discharge (whichever occurs first)**		
Vital signs ~~(incl. systolic and diastolic blood pressure, heart rate and respiratory rate, and SpO_2)~~ as well as ~~reason, flow-~~	Vital signs as well as O_2 supplementation are recorded as indicated in Section **Fehler! Verweisquelle konnte nicht**	

Previous and new wording in track change modus	New wording	Comments/ reasons for substantial amendment
rate and method of O_2 supplementation are recorded as indicated in Section **Fehler! Verweisquelle konnte nicht gefunden werden.** Vital signs and Section **Fehler! Verweisquelle konnte nicht gefunden werden.** Oxygen administration.	**gefunden werden.** Vital signs and Section **Fehler! Verweisquelle konnte nicht gefunden werden.** Oxygen administration.	
**9.10 V7 – Day 90 ±10 after start of NBHO (or randomization in the control arm)**		
Vital signs (incl. systolic and diastolic blood pressure, heart rate and respiratory rate, and SpO_2) as well as reason, flow-rate and method of O_2 supplementation are recorded as indicated in Section **Fehler! Verweisquelle konnte nicht gefunden werden.** Vital signs and Section **Fehler! Verweisquelle konnte nicht gefunden werden.** Oxygen administration.	Vital signs as well as O_2 supplementation are recorded as indicated in Section **Fehler! Verweisquelle konnte nicht gefunden werden.** Vital signs and Section **Fehler! Verweisquelle konnte nicht gefunden werden.** Oxygen administration.	
**Table 13**: Items to be assessed during V7 if performed face-to-face versus via phone	**Table 13**: Items to be assessed during V7 if performed face-to-face versus via phone	• Specification of vital signs to be collected at face-to-face and phone visit.<br>• Specification of record methods of oxygen administration<br>• Revision of foot note 6.
**10. Vital Signs**		
Vital signs (systolic and diastolic blood pressure, heart rate and respiratory rate, SpO_2eripheral capillary oxygen saturation, and – if available – $etCO_2$) determined on predefined study days and time points (see Section **Fehler! Verweisquelle konnte nicht gefunden werden.** Trial Schedule) will be documented as numerical values on appropriate eCRF-pages.	Vital signs (systolic and diastolic blood pressure, heart rate and respiratory rate, SpO_2, and – if available – $etCO_2$) determined on predefined study days and time points (see Section **Fehler! Verweisquelle konnte nicht gefunden werden.** Trial Schedule) will be documented as numerical values on appropriate eCRF-pages.	
**10.15 Oxygen administration**		
Oxygen administration including reason (either NBHO or standard of care oxygen supplementation), flow-rate or FiO_2 in case of ventilation, and mask-type/method are documented in the eCRF at prespecified time points (see	Oxygen administration including reason (either NBHO or standard of care oxygen supplementation), flow-rate or FiO_2 in case of ventilation, and mask-type/method are documented in the eCRF at prespecified time points (see	

Previous and new wording in track change modus	New wording	Comments/ reasons for substantial amendment
Section **Fehler! Verweisquelle konnte nicht gefunden werden.** Trial Schedule), including all documented pre-randomization and pre-hospital changes of O_2 administration with reason, flow-rate, mask-type, duration, ~~and~~ SpO_2~~-~~, and – if available – $etCO_2$~~(if possible in 15-minute intervals)~~.	Section **Fehler! Verweisquelle konnte nicht gefunden werden.** Trial Schedule), including all documented pre-randomization and pre-hospital changes of O_2 administration with reason, flow-rate, mask-type, duration, SpO_2, and – if available – $etCO_2$.	
**12.2.2 Secondary analysis variables, secondary imaging efficacy analyses:**		
• PP only: absolute difference in ischemic core volume (in mL) [time frame: 24 hours]<br>• relative changes in ischemic core volume (in %) [time frame: 24 hours]<br>• absolute and relative ischemic core change using either NCCT or DWI-MRI (or CT/MR angiography) for ischemic core estimation at baseline (i.e. NCCT (or CT angiography) will substitute for CBF < 30% in patients with CT-based imaging at baseline, and MR angiography may substitute for DWI-MRI) [time frame: 24 hours]<br>• absolute and relative ischemic core change using CBF < 30% for ischemic core estimation at baseline in all patients, independent of imaging modality (i.e. DWI will be substituted by MR perfusion CBF < 30%) [time frame: 24 hours]<br>• penumbral salvage defined as (penumbra volume at baseline – infarct core volume at 24 hours) / (penumbra volume at baseline – ischemic core volume at baseline) [time frame: 24 hours]<br>• of patients who received TBY: proportion of TICI on DSA (final run) (as suggested in [173]) [time frame: 2 to 4 hours]<br>• revascularization rate on 24-hour follow-up MRA (or CTA if available) [time frame: 24 hours	• PP only: absolute difference in ischemic core volume (in mL) [time frame: 24 hours]<br>• relative changes in ischemic core volume (in %) [time frame: 24 hours]<br>• absolute and relative ischemic core change using either NCCT or DWI-MRI (or CT/MR angiography) for ischemic core estimation at baseline (i.e. NCCT (or CT angiography) will substitute for CBF < 30% in patients with CT-based imaging at baseline, and MR angiography may substitute for DWI-MRI) [time frame: 24 hours]<br>• absolute and relative ischemic core change using CBF < 30% for ischemic core estimation at baseline in all patients, independent of imaging modality (i.e. DWI will be substituted by MR perfusion CBF < 30%) [time frame: 24 hours]<br>• penumbral salvage defined as (penumbra volume at baseline – infarct core volume at 24 hours) / (penumbra volume at baseline – ischemic core volume at baseline) [time frame: 24 hours]<br>• of patients who received TBY: proportion of TICI on DSA (final run) (as suggested in [173]) [time frame: 2 to 4 hours]<br>• revascularization rate on 24-hour follow-up MRA (or CTA if available) [time frame: 24 hours	
**12.3 Definition of Trial Population to be analyzed**		
Other subsets of the full analysis set comprise patients with the following characteristics:	Other subsets of the full analysis set comprise patients with the following characteristics:	

Previous and new wording in track change modus	New wording	Comments/ reasons for substantial amendment
• TICI 2b/3 at end of TBY and target mismatch profile (i.e. baseline penumbra (T_{max} > 6 seconds)-: core (CBF < 30% compared to healthy tissue) ratio ≥ 1.2 and volume ≥ 15 mL; voxel-based post-hoc analyses) vs. TICI 2b/3 at end of TBY and no target mismatch profile<br>• TICI 3 at end of TBY and target mismatch profile vs. TICI 3 at end of TBY and no target mismatch profile<br>• TICI 2b/3 at end of TBY and baseline ischemic core volume ≤ 100 mL and target mismatch profile vs. TICI 2b/3 at end of TBY and large ischemic core at baseline (> 100 mL) and no target mismatch profile<br>• TICI 3 at end of TBY and baseline ischemic core volume ≤ 100 mL and target mismatch profile vs. TICI 3 at end of TBY and large ischemic core at baseline (> 100 mL) and no target mismatch profile<br>• TICI 2b/3 at end of TBY vs. TICI 0-2a at end of TBY or TBY not attempted<br>• TICI 2a-3 at end of TBY vs. TICI 0-1 at end of TBY or TBY not attempted<br>• Patients with complete reperfusion of target mismatch area (correlation of CT or MR perfusion with DSA (final run)) vs. patients with no or incomplete reperfusion of target mismatch area<br>• ASPECTS ≤ 8 on baseline NCCT or ≤ 7 on baseline DWI vs. ASPECTS ≥ 9 on baseline NCCT or ≥ 8 on baseline DWI<br>• intubation/ventilation vs. conscious sedation<br>• IVT vs. no-IVT<br>• age ≤ 60 vs. > 60<br>• age ≤ 70 vs. > 70<br>• age ≤ 80 vs. > 80<br>• NIHSS at baseline < 10 vs. 10-20 vs. > 20, +/- cross-classification with LVO-location	• TICI 2b/3 at end of TBY and target mismatch profile (i.e. baseline penumbra (T_{max} > 6 seconds): core (CBF < 30% compared to healthy tissue) ratio ≥ 1.2 and volume ≥ 15 mL; voxel-based post-hoc analyses) vs. TICI 2b/3 at end of TBY and no target mismatch profile<br>• TICI 3 at end of TBY and target mismatch profile vs. TICI 3 at end of TBY and no target mismatch profile<br>• TICI 2b/3 at end of TBY and baseline ischemic core volume ≤ 100 mL and target mismatch profile vs. TICI 2b/3 at end of TBY and large ischemic core at baseline (> 100 mL) and no target mismatch profile<br>• TICI 3 at end of TBY and baseline ischemic core volume ≤ 100 mL and target mismatch profile vs. TICI 3 at end of TBY and large ischemic core at baseline (> 100 mL) and no target mismatch profile<br>• TICI 2b/3 at end of TBY vs. TICI 0-2a at end of TBY or TBY not attempted<br>• TICI 2a-3 at end of TBY vs. TICI 0-1 at end of TBY or TBY not attempted<br>• Patients with complete reperfusion of target mismatch area (correlation of CT or MR perfusion with DSA (final run)) vs. patients with no or incomplete reperfusion of target mismatch area<br>• ASPECTS ≤ 8 on baseline NCCT or ≤ 7 on baseline DWI vs. ASPECTS ≥ 9 on baseline NCCT or ≥ 8 on baseline DWI<br>• intubation/ventilation vs. conscious sedation<br>• IVT vs. no-IVT<br>• age ≤ 60 vs. > 60<br>• age ≤ 70 vs. > 70<br>• age ≤ 80 vs. > 80	

Previous and new wording in track change modus	New wording	Comments/ reasons for substantial amendment
• intracranial LVO location: terminal ICA with involvement of the M1-segment of the MCA/carotid-T vs. proximal M1-segment vs. distal M1-segments (distal to perforating branches) vs. M2/3-segment(s)<br>• Time window 0-3 vs. > 3-6 hours<br>• Tandem stenosis/extracranial occlusion vs. no tandem stenosis/extracranial occlusion	• NIHSS at baseline < 10 vs. 10-20 vs. > 20, +/- cross-classification with LVO-location<br>• intracranial LVO location: terminal ICA with involvement of the M1-segment of the MCA/carotid-T vs. proximal M1-segment vs. distal M1-segments (distal to perforating branches) vs. M2/3-segment(s)<br>• Time window 0-3 vs. > 3-6 hours<br>Tandem stenosis/extracranial occlusion vs. no tandem stenosis/extracranial occlusion	
**17 Signatures**		
		Insertion of print names of sponsor representative, coordinating investigator and biometrician
**19 Appendices**		
Version 1.1 3 / 26.0206.12.2019	Version 1.3 / 06.12.2019	Correction of version in header
