# Supplementary material for: Penumbral Rescue by normobaric O = O administration in patients with ischemic stroke and target mismatch proFile (PROOF): Study protocol of a phase IIb trial
Source: Int J Stroke. 2023 Aug 18;19(1):120–6. doi: 10.1177/17474930231185275 (PMC10759237; doi:10.1177/17474930231185275)
Supplement: sj-pdf-4-wso-10.1177_17474930231185275 – Supplemental material for Penumbral Rescue by normobaric O = O administration in patients with ischemic stroke and target mismatch proFile (PROOF): Study protocol of a phase IIb trial [file sj-pdf-4-wso-10.1177_17474930231185275.pdf]

Appendix 1 Description of the substantial amendment

Clinical Study Protocol "PROOF: Penumbral Rescue by Normobaric O=O Administration in Patients with Ischemic Stroke and Target Mismatch ProFile: A Phase II Proof-of-Concept Trial"
Phase of study: Phase II – proof-of-concept
EudraCT No.: 2017-001355-31
Study Registry Number: NCT03500939

The respective changes made to the Protocol Version 1.3/ 06.12.2019 to Version 1.4/ 17.03.2021 are tabulated below.

Previous and new wording in track change modus	New wording	Comments/ reasons for substantial amendment
**Header**		
Version 1.4 / 06.12.201917.03.2021	Version 1.4 / 17.03.2021	
**Footer**		
		Addition of University Hospital Tübingen logo and update of KKS Heidelberg logo.
**ADMINISTRATIVE STRUCTURE**		
**Coodinating investigator (Leiter der klinischen Prüfung*)**<br>University Hospital Tübingen<br>Dept. of Neurology with Focus on Neurovascular Diseases and Neurooncology<br>PD Dr. med. Sven Poli<br>Hoppe-Seyler-Str. 3<br>72076 Tübingen<br>Germany<br>Phone: 0049 172 4682284<br>Fax: 0049 7071 29 25047<br>Email: sven.poli@uni-tuebingen.de	**Coodinating investigator (Leiter der klinischen Prüfung*)**<br>University Hospital Tübingen<br>Dept. of Neurology with Focus on Neurovascular Diseases and Neurooncology<br>PD Dr. med. Sven Poli<br>Hoppe-Seyler-Str. 3<br>72076 Tübingen<br>Germany<br>Phone: 0049 172 4682284<br>Fax: 0049 7071 29 25047<br>Email: sven.poli@uni-tuebingen.de	Update of academic title
**ADMINISTRATIVE STRUCTURE**		
**Project Management:**<br>Coordination Centre for Clinical trials (KKS)<br>Dr. Maike Nilsson, (Ph.D.)<br>Marsilius-Arkaden / Turm West<br>Im Neuenheimer Feld 130.3<br>69120 Heidelberg<br>Germany<br>Phone: 0049 6221 56 32671<br>Fax: 0049 6221 56 33508<br>Email: maike.nilsson@med.uni-heidelberg.de	**Project Management:**<br>Coordination Centre for Clinical trials (KKS)<br>Dr. Maike Nilsson, Ph.D.<br>Marsilius-Arkaden / Turm West<br>Im Neuenheimer Feld 130.3<br>69120 Heidelberg<br>Germany<br>Phone: 0049 6221 56 32671<br>Fax: 0049 6221 56 33508<br>Email: maike.nilsson@med.uni-heidelberg.de	Correction of citation of academic title
**ADMINISTRATIVE STRUCTURE**		
**Pharmacovigilance:**	**Pharmacovigilance:**	Correction of mail address

Previous and new wording in track change modus	New wording	Comments/ reasons for substantial amendment
Coordination Centre for Clinical trials (KKS)<br>Julia Merkle-Lock<br>Marsilius-Arkaden / Turm West<br>Im Neuenheimer Feld 130.3<br>69120 Heidelberg<br>Germany<br>Phone: 0049 6221 56 35117<br>Fax: **0049 6221 56 33725 (SAE-FAX!)**<br>Email: pharmakovigilanz.KKS@med.uni-heidelberg.de~~julia.merkle-lock@med.uni-heidelberg.de~~	Coordination Centre for Clinical trials (KKS)<br>Julia Merkle-Lock<br>Marsilius-Arkaden / Turm West<br>Im Neuenheimer Feld 130.3<br>69120 Heidelberg<br>Germany<br>Phone: 0049 6221 56 35117<br>Fax: **0049 6221 56 33725 (SAE-FAX!)**<br>Email: pharmakovigilanz.KKS@med.uni-heidelberg.de	
**ADMINISTRATIVE STRUCTURE**		
**Independent Ethical Advisory Board (IEAB)**<br>Prof. Dr. Matthew Schrag~~Prof. Dr. med. Eivind Berge~~<br>Vanderbilt University School of Medicine~~Kirkeveien 166~~<br>Medical Research Building III~~NO-0407 Oslo~~<br>465 21st Avenue S, Suite 6160 (Office 6158C)~~Norway~~<br>Nashville, TN 37240<br>United States of America~~Phone: 0047 2211 9100~~<br>Email: matthew.schrag@vanderbilt.edu~~Fax: 0047 22118280~~<br>~~Email: eivind.berge@medisin.uio.no~~	**Independent Ethical Advisory Board (IEAB)**<br>Prof. Dr. Matthew Schrag<br>Vanderbilt University School of Medicine<br>Medical Research Building III<br>465 21st Avenue S, Suite 6160 (Office 6158C)<br>Nashville, TN 37240, United States of America<br>Email: matthew.schrag@vanderbilt.edu	change of staff due to the death of Prof. Dr. Eivind Berge
**ADMINISTRATIVE STRUCTURE**		

Previous and new wording in track change modus	New wording	Comments/ reasons for substantial amendment
**National Coordinators**<br>***SWITZERLAND***<br>Centre Hospitalier Universitaire Vaudois<br>Prof. Dr. Patrik Michel<br>Rue du Bugnon 21<br>Lausanne 1005<br>Email: patrik.michel@chuv.ch<br><br>***SWITZERLAND***<br>Centre Hospitalier Universitaire Vaudois<br>Prof. Dr. Patrik Michel<br>Rue du Bugnon 46<br>1011 Lausanne<br>Email: patrik.michel@chuv.ch	***SWITZERLAND***<br>Centre Hospitalier Universitaire Vaudois<br>Prof. Dr. Patrik Michel<br>Rue du Bugnon 46<br>1011 Lausanne<br>Email: patrik.michel@chuv.ch	Correction of address
**1 Protocol Synopsis, Objectives**		
Secondary imaging efficacy endpoints: relative changes in ischemic core volume (in %) from baseline to 24 hours; absolute and relative ischemic core change from baseline to 24 hours using either NCCT or DWI-MRI (or CT/MR angiography source images and DWI) for ischemic core estimation at baseline; absolute and relative ischemic core change from baseline to 24 hours using cerebral blood flow (CBF)<br>< 30% for ischemic core estimation at baseline in all patients, independent of imaging modality; penumbral salvage from baseline to 24 hours; TICI (Thrombolysis in Cerebral Infarction perfusion scale grade) in patients who underwent TBY; revascularization on 24-hour follow-up imaging.	Secondary imaging efficacy endpoints: relative changes in ischemic core volume (in %) from baseline to 24 hours; absolute and relative ischemic core change from baseline to 24 hours using either NCCT or DWI-MRI (or CT angiography source images and DWI) for ischemic core estimation at baseline; absolute and relative ischemic core change from baseline to 24 hours using cerebral blood flow (CBF)<br>< 30% for ischemic core estimation at baseline in all patients, independent of imaging modality; penumbral salvage from baseline to 24 hours; TICI (Thrombolysis in Cerebral Infarction perfusion scale grade) in patients who underwent TBY; revascularization on 24-hour follow-up imaging.	Correction of clerical error: MR angiography was never planned for ischemic core estimation. DWI is the correct modality.<br><br>Clarification by adding "source images": because only CT angiography source images but not reconstructions of CT angiography can be used for core estimation.
**1 Protocol Synopsis, Investigational Medicinal Product**		
IMP: NBHO, i.e. inhalation of 100% oxygen at high flow (≥ 40 L/min) via a sealed non-rebreather face-mask with reservoir, or in case of	IMP: NBHO, i.e. inhalation of 100% oxygen at high flow (≥ 40 L/min) via a sealed non-rebreather face-mask with reservoir, or in case of	Adaption according to current ESMINT/ESO and AHA/ASA guidelines [159, 160] will also facilitate recruitment. In the DAWN and the

Previous and new wording in track change modus	New wording	Comments/ reasons for substantial amendment
intubation/ventilation for (study-independent) TBY, ventilation with an inspiratory oxygen fraction (FiO_2) of 1.0. NBHO is started within 6 hours of stroke symptom onset (witnessed~~ or last seen well~~) or symptom recognition (in case of wake-up or unknown onset stroke), and within 30 minutes after end of baseline brain imaging and applied until the end of TBY procedure (defined by removal of guide catheter from sheath) or, in case TBY is not attempted (defined as ‘TBY was not attempted or intervention was stopped prior to any penetration or aspiration of the qualifying (i.e. intracranial) LVO’), 4 hours after start of study treatment.	intubation/ventilation for (study-independent) TBY, ventilation with an inspiratory oxygen fraction (FiO_2) of 1.0. NBHO is started within 6 hours of stroke symptom onset (witnessed) or symptom recognition (in case of wake-up or unknown onset stroke), and within 30 minutes after end of baseline brain imaging and applied until the end of TBY procedure (defined by removal of guide catheter from sheath) or, in case TBY is not attempted (defined as ‘TBY was not attempted or intervention was stopped prior to any penetration or aspiration of the qualifying (i.e. intracranial) LVO’), 4 hours after start of study treatment.	DEFUSE 3 randomized controlled trials, endovascular mechanical thrombectomy was clearly beneficial in patients with wake-up stroke/unknown onset stroke due to significant volumes of salvageable ischemic brain tissue [106][107]. Consequently, beneficial effects of NBHO can equally be assumed in wake-up stroke/unknown onset stroke patients with small ischemic core at baseline as per PROOF inclusion criteria. In order to avoid any imbalance, we adapted variable used in minimization (see 8.5.1; “Time window known < 6h vs. unknown/wake-up”)
**1 Protocol Synopsis, Study population**		
Inclusion Criteria<br>• Age: ~~>~~ ≥ 18 years<br>• Acute anterior circulation ischemic stroke due to a Large vessel occlusion (LVO) on CT or MR angiography, i.e. either **terminal internal carotid artery (ICA)** with M1/carotid-T, **proximal M1**, **distal M1** (distal to perforating branches), or **M2/3 segment(s)**<br>• **If TBY is likely to be conducted**<br>• NIHSS score of ≥ 6 at screening<br>• Alberta Stroke Program Early CT score (ASPECTS) of ~~7~~6-10 on non-contrast CT or ~~6~~5-10 on diffusion-weighted MRI (DWI-MRI)<br>• If recommended by the attending physician, CT or MR perfusion ~~(whole-brain or minimal coverage ≥ 75 mm)~~should be performed prior to NBHO<br>• NBHO can be initiated within 6 hours of symptom onset (witnessed~~ or last seen well~~) or symptom	Inclusion Criteria<br>• Age: ≥ 18 years<br>• Acute anterior circulation ischemic stroke due to a Large vessel occlusion (LVO) on CT or MR angiography, i.e. either **terminal internal carotid artery (ICA)** with M1/carotid-T, **proximal M1**, **distal M1** (distal to perforating branches), or **M2/3 segment(s)**<br>• **If TBY is likely to be conducted**<br>• NIHSS score of ≥ 6 at screening<br>• Alberta Stroke Program Early CT score (ASPECTS) of 6-10 on non-contrast CT or 5-10 on diffusion-weighted MRI (DWI-MRI)<br>• If recommended by the attending physician, CT or MR perfusion should be performed prior to NBHO<br>• NBHO can be initiated within 6 hours of symptom onset (witnessed) or symptom recognition (in case of wake-up or unknown onset stroke), and	Correction of a clerical error: all adult patients including 18-year-old patients may be enrolled as it was previously the case in protocol version 1.2<br><br>Adaptions according to current ESMINT/ESO and AHA/ASA guidelines [159, 160].<br>• Today, ASPECTS of 6 on NCCT (or 5 on DWI-MRI) are routinely treated with endovascular mechanical thrombectomy. High treatment effects due to significant volumes of salvageable ischemic brain tissue (Goyal et la., Lancet 2016) indicate that beneficial effects of NBHO can equally be assumed in patients with an – still high enough – ASPECTS of 6 on NCCT (or 5 on DWI-MRI). This adaption had already been pre-specified in the previous protocol

Previous and new wording in track change modus	New wording	Comments/ reasons for substantial amendment
recognition (in case of wake-up or unknown onset stroke), and within 30 minutes after last image of baseline brain imaging<br>• Pre-stroke mRS of 0 ~~or 1~~to 2<br>• Breastfeeding women must stop breastfeeding after randomization<br>• Own written informed consent is not obtained prior to study inclusion but has to be gained as soon as possible. Patients who are able to give consent will be informed about trial participation orally and may consent to or decline participation. Patients unable to give consent will be enrolled through a deferred consent procedure (see Section 14.5 Subject Information and Informed Consent)	within 30 minutes after last image of baseline brain imaging<br>• Pre-stroke mRS of 0 to 2<br>• Breastfeeding women must stop breastfeeding after randomization<br>• Own written informed consent is not obtained prior to study inclusion but has to be gained as soon as possible. Patients who are able to give consent will be informed about trial participation orally and may consent to or decline participation. Patients unable to give consent will be enrolled through a deferred consent procedure (see Section 14.5 Subject Information and Informed Consent)	version for facilitate recruitment (see Chapter 7.6 of protocol version 1.3).<br>• According to current guidelines perfusion imaging is only obligatory for specific subgroups such as patients with wake-up or unknown onset strokes, but not in patients with known stroke onset <6h. In cases no perfusion imaging will be available, CT angiography source images will be used for ischemic core estimation at baseline (see Chapter 10.4)<br>• Adaption according to current ESMINT/ESO and AHA/ASA guidelines [159, 160] will also facilitate recruitment. In the DAWN and the DEFUSE 3 randomized controlled trials, endovascular mechanical thrombectomy was clearly beneficial in patients with wake-up stroke/unknown onset stroke due to significant volumes of salvageable ischemic brain tissue [106][107]. Consequently, beneficial effects of NBHO can equally be assumed in wake-up stroke/unknown onset stroke patients with small ischemic core at baseline as per PROOF inclusion criteria. In order to avoid any imbalance, we adapted variable used in minimization (see 8.5.1; “Time window known < 6h vs. unknown/wake-up”)

Previous and new wording in track change modus	New wording	Comments/ reasons for substantial amendment
		Today, patients with a pre-stroke modified Rankin Scale (mRS) score of 2 are routinely treated with endovascular mechanical thrombectomy. Mild pre-existing disability (as signified by the mRS of 2) did not limit recovery after stroke (Goyal et la., Lancet 2016). This adaption had already been pre-specified in the previous protocol version for facilitate recruitment (see Chapter 7.6 of protocol version 1.3).
**1 Protocol Synopsis, Study population, Exclusion criteria**		
Neurological:<br>• Acute bilateral stroke or stroke in multiple vascular territories (except of clinically silent ~~micro-~~lesions)	Neurological:<br>• Acute bilateral stroke or stroke in multiple vascular territories (except of clinically silent lesions)	Correction of wording in order to avoid unnecessary exclusion of patients due to overly strict interpretation of "micro". The primary (imaging) endpoint is unaffected by this adaption. Because most silent ischemic lesions are small, affection of secondary clinical endpoints can be assumed to be low. Nonetheless, we added the pre-specified subgroup analysis of patients with or without silent ischemic lesions in order to address any no-matter-how-small impact on clinical outcomes (compare Chapter 12.3)
**1 Protocol Synopsis, Study population, Exclusion criteria**		
Respiratory:<br>• Acute or chronic pulmonary disease or respiratory distress that may, in the clinical judgement of the investigator, interfere with the study intervention (e.g. acute pneumonia, COPD flare-up etc.) ~~Known history of chronic pulmonary disease (e.g. COPD, pulmonary fibrosis, alveolitis or pneumonitis)~~<br>• Prior to enrolment, > 2 L/min oxygen required to maintain peripheral oxygen saturation ≥ 95%	Respiratory:<br>• Acute or chronic pulmonary disease or respiratory distress that may, in the clinical judgement of the investigator, interfere with the study intervention (e.g. acute pneumonia, COPD flare-up etc.)<br>• Prior to enrolment, > 2 L/min oxygen required to maintain peripheral oxygen saturation ≥ 95%	Simplification of wording without substantive change

Previous and new wording in track change modus	New wording	Comments/ reasons for substantial amendment
~~Acute respiratory distress that may, in the clinical judgment of the investigator, interfere with the study intervention~~<br>~~Acute pneumonia, alveolitis or pneumonitis of viral, bacterial, fungal or any other etiology~~		
**1 Protocol Synopsis, Trial duration**		
Total trial duration: ~~60~~ 84 months<br>Duration of clinical phase: ~~36~~ 48 months<br>Beginning of the preparation phase: Q1 2017<br>FSI (first subject in): August 2019<br>LSI (last subject in): Q1 2023~~1~~<br>LSO (last subject out): Q2 2023~~1~~<br>DBL (database lock): Q3 2023~~1~~<br>Statistical analyses completed: Q4 2023~~1~~<br>Trial report completed: Q4 2023~~1~~	Total trial duration: 84 months<br>Duration of clinical phase: 48 months<br>Beginning of the preparation phase: Q1 2017<br>FSI (first subject in): August 2019<br>LSI (last subject in): Q1 2023<br>LSO (last subject out): Q2 2023<br>DBL (database lock): Q3 2023<br>Statistical analyses completed: Q4 2023<br>Trial report completed: Q4 2023	Adaption according to current time line. Delays were due to unforeseeable administrative hurdles, Covid-19 pandemic and lastly critical DSMB evaluation as well as low recruitment. Adaptions of inclusion/exclusion criteria will not only enhance generalizability of results but further increase feasibility of study conduction and thereby, guarantee sufficient recruitment and – at least partly – to recover the gap: 92 (48%), 46 (24%), and 22 (12%) of a total of 190 patients who underwent endovascular mechanical thrombectomy due to acute anterior circulation large vessel occlusion at EKUT in between July 2 2019 and Feb 6 2021 might have been enrolled as per inclusion and exclusion criteria of protocol versions 1.4, 1.3, and 1.2, respectively.
**1 Protocol Synopsis, Statistical Analysis**		
Efficacy of NBHO treatment: ITT analysis of difference of ischemic core growth (defined as the difference in ischemic core volume (in mL) from baseline to 24 hours) between groups; brain tissue not included in CT perfusion (or, in case CT perfusion is not available or of insufficient quality, CT angiography source images) or MR diffusion at baseline will be excluded from lesion volume measurements.	Efficacy of NBHO treatment: ITT analysis of difference of ischemic core growth (defined as the difference in ischemic core volume (in mL) from baseline to 24 hours) between groups; brain tissue not included in CT perfusion (or, in case CT perfusion is not available or of insufficient quality, CT angiography source images) or MR diffusion at baseline will be excluded from lesion volume measurements.	Adaptions according to current ESMINT/ESO and AHA/ASA guidelines [159, 160] in which perfusion imaging is only obligatory for specific subgroups such as patients with wake-up or unknown onset strokes, but not in patients with known stroke onset <6h. In cases no perfusion imaging will be available, CT angiography source images will be used for ischemic

Previous and new wording in track change modus		New wording		Comments/ reasons for substantial amendment
				core estimation at baseline (see Chapter 10.4). This adaption will not only facilitate enrollment but also compensate for drop-outs due to insufficient quality of perfusion imaging, which has been observed in ~10% of enrolled cases.
**2 Trial Schedule**				
				Revision of flow chart and foot notes to reflect the protocol changes.
**3 Abbreviations**				
ACS	Acute coronary syndromes	ACS	Acute coronary syndromes	Correction of spelling of abbreviation "ACS"
AE	Adverse Event	AE	Adverse Event	Correction of alphabetic order of the list of abbreviations, i.e., shift of abbreviation "AE"
AESI	Adverse Event of Special Interest	AESI	Adverse Event of Special Interest	Insertion of new abbreviations "AESI" to "VENI"
COVID-19	Coronavirus	COVID-19	Coronavirus	
CTA	Computed Tomography Angiography	CTA	Computed Tomography Angiography	
EVT	Endovascular Treatment	EVT	Endovascular Treatment	
FFP2/3	Filtering Face Piece 2/3	FFP2/3	Filtering Face Piece 2/3	
LND	Late Neurological Deterioration	LND	Late Neurological Deterioration	
LNI	Late Neurological Improvement	LNI	Late Neurological Improvement	
MACE	Major Adverse Cardiovascular Events	MACE	Major Adverse Cardiovascular Events	
$PaCO_2$	Partial pressure of carbon dioxide	$PaCO_2$	Partial pressure of carbon dioxide	
PaO_2	Partial pressure of oxygen	PaO_2	Partial pressure of oxygen	
POC	Point-of-Care	POC	Point-of-Care	
RCT	Randomized Controlled Trial	RCT	Randomized Controlled Trial	
SaO_2	Oxygen saturation	SaO_2	Oxygen saturation	
SARS-CoV-2	Severe Acute Respiratory Syndrome-Corona Virus-2	SARS-CoV-2	Severe Acute Respiratory Syndrome-Corona Virus-2	
sICH	Symptomatic Intracranial Hemorrhage	sICH	Symptomatic Intracranial Hemorrhage	
SND	Subacute Neurological Deterioration	SND	Subacute Neurological Deterioration	
SNI	Subacute Neurological Improvement	SNI	Subacute Neurological Improvement	
sNIHSS-EMS	Shortened NIHSS for emergency medical services	sNIHSS-EMS	Shortened NIHSS for emergency medical services	

Previous and new wording in track change modus	New wording	Comments/ reasons for substantial amendment
V Visit<br>VEND Very Early Neurological Deterioration<br>VENI Very Early Neurological Improvement	V Visit<br>VEND Very Early Neurological Deterioration<br>VENI Very Early Neurological Improvement	
**4.3. Risk-Benefit Assessment (page 26)**		
Blood sampling for biomarker-analysis at Screening~~,~~ ~~V3~~ and V5 is drawn from a study-independent venous or arterial access only, i.e. not associated with any risk~~! Blood sampling for biomarkers at V6 is done either via a routinely inserted venous access or – combined with safety laboratory assessment – through venipuncture (with the abovementioned risks). However, biomarker samples at V6 are only drawn if the patient or the respective LAR consents to the participation in the biomarker sub-study~~.<br><br>Study-dependent arterial blood samples:<br>Arterial blood gases are drawn from a study-independent arterial access only. This means that an arterial blood gas analysis is performed at V3 as a part of clinical routine nearly only in patients undergoing TBY. If an arterial access is still available, an additional study-dependent blood gas analysis is performed at V5.<br>No arterial access is established as a study procedure.<br><br>The total amount of blood drawn solely for study purposes amounts to ~~90~~54.5 mL in case the patient consents to participation in the biomarker sub-study or to ~~54~~36.5 mL if participation in the biomarker sub-study is refused prior to V5 or ~~72~~18.5 mL if participation in the biomarker sub-study is refused at screening~~prior to V6~~ (see Sections 10.18 Laboratory assessment, 10.19 Biomarkers (Pharmacodynamics), and 10.20 Arterial blood gases (Pharmacokinetics) as well as 14 for details).	Blood sampling for biomarker-analysis at Screening and V5 is drawn from a study-independent venous or arterial access only, i.e. not associated with any risk.<br><br>Study-dependent arterial blood samples:<br>Arterial blood gases are drawn from a study-independent arterial access only. This means that an arterial blood gas analysis is performed at V3 as a part of clinical routine nearly only in patients undergoing TBY. If an arterial access is still available, an additional study-dependent blood gas analysis is performed at V5.<br>No arterial access is established as a study procedure.<br><br>The total amount of blood drawn solely for study purposes amounts to 54.5 mL in case the patient consents to participation in the biomarker sub-study or to 36.5 mL if participation in the biomarker sub-study is refused prior to V5 or 18.5 mL if participation in the biomarker sub-study is refused at screening see Sections 10.18 Laboratory assessment, 10.19 Biomarkers (Pharmacodynamics), and 10.20 Arterial blood gases (Pharmacokinetics) as well as 14 for details).<br>Adverse effects due to the removal of this small amount of blood are not to be expected.	Biomarker substudy has been simplified in order to ensure its conduction.<br>Consequently, blood volume for biomarkers has been reduced from 72 to 36 mL and max. total blood volume has been reduced accordingly from 90.5 mL to now 54.5 mL (see also Table 14 of the study protocol).

**Previous and new wording in track change modus**	**New wording**	**Comments/ reasons for substantial amendment**
Adverse effects due to the removal of this small amount of blood are not to be expected.		
**4.3. Risk-Benefit Assessment (page 28)**		
In case of negative effects of NBHO on neurological outcome, we will be able to detect these through frequent safety assessments during the trial (provided to the DSMB and the sponsor every 6 months). Additionally, every death, parenchymal hematoma and remote intracranial bleeding (see Appendix 14 for Heidelberg bleeding classification [101]) will be reported expedited to the DSMB. Imaging and clinical outcome data will be reviewed by the DSMB. In case of an excess of unfavorable outcomes (e.g. increased mortality, bleeding risk, high rates of SAE, etc.) the trial will be suspended for in-depth DSMB review in order to prevent harm from further trial participants. If it is determined that the excess in safety outcomes is related to NBHO, the trial will be discontinued.	In case of negative effects of NBHO on neurological outcome, we will be able to detect these through frequent safety assessments during the trial (provided to the DSMB and the sponsor every 6 months). Additionally, every death, parenchymal hematoma and remote intracranial bleeding (see Appendix 14 for Heidelberg bleeding classification [101]) will be reported expedited to the DSMB. Imaging and clinical outcome data will be reviewed by the DSMB. In case of an excess of unfavorable outcomes (e.g. increased mortality, bleeding risk, high rates of SAE, etc.) the trial will be suspended for in-depth DSMB review in order to prevent harm from further trial participants. If it is determined that the excess in safety outcomes is related to NBHO, the trial will be discontinued.	Intensified safety monitoring has been implemented according to DSMB's requirements.
**4.3.1 Risk-Benefit Assessment Considering the Literature until December 6th, 2019 (page 29)**		
**Risk-Benefit Assessment Considering the ~~Most Recent~~ Literature until December 6, 2019** This chapter will discuss any ambiguity regarding the risk-benefit assessment and consent modalities of the PROOF study trial considering recently published review articles including one meta-analysis as well as the ~~most recently presented~~ main results of the **New Zealand Oxygen in Acute Coronary Syndromes Trial (NZOTACS)** that had been first presented at the European Society of Cardiology congress in Paris, France, in 2019 [102].	**Risk-Benefit Assessment Considering the Literature until December 6, 2019** This chapter will discuss any ambiguity regarding the risk-benefit assessment and consent modalities of the PROOF study trial considering recently published review articles including one meta-analysis as well as the main results of the **New Zealand Oxygen in Acute Coronary Syndromes Trial (NZOTACS)** that had been first presented at the European Society of Cardiology congress in Paris, France, in 2019 [102]	Revision of subsection header<br><br>The NZOTACS trial has been published in the British Medical Journal on March 2nd, 2021 [102]. Accordingly, we updated the sentence and the respective reference without any change of content.
**4.3.1 Risk-Benefit Assessment Considering the Literature until December 6th, 2019 (page 32)**		

Previous and new wording in track change modus	New wording	Comments/ reasons for substantial amendment
Nonetheless, in the few human clinical trials where at least one framework condition was fulfilled, the evidence points to a clinical benefit to acute ischemic strokes: Breathing oxygen in a dose of $FiO_2 \geq 0.95$, which was proven sufficient in animal experiments, led to a stabilization or even a regression of the ischemia (see Fig. 4) and an improvement in ~~NIHHS~~ NIHSS scores [76]. However, this effect was only temporary because no reperfusion treatment was performed on the patients ('nothing can hold its breath forever', compare Fig. 1 and final infarct versus failed recanalization in Fig. 3).	Nonetheless, in the few human clinical trials where at least one framework condition was fulfilled, the evidence points to a clinical benefit to acute ischemic strokes: Breathing oxygen in a dose of $FiO_2 \geq 0.95$, which was proven sufficient in animal experiments, led to a stabilization or even a regression of the ischemia (see Fig. 4) and an improvement in NIHSS scores [76]. However, this effect was only temporary because no reperfusion treatment was performed on the patients ('nothing can hold its breath forever', compare Fig. 1 and final infarct versus failed recanalization in Fig. 3).	Correction of spelling mistake
**4.3.1 Risk-Benefit Assessment Considering the Literature until December 6th, 2019 (page 33)**		
**How does the PROOF trial differ?** The PROOF study is the first human NBHO trial to consider all three framework requirements for a successful "freezing of the penumbra" and lasting conservation of positive effects through: (1) early reperfusion (compare Fig. 1 and final infarct versus failed recanalization in Fig. 3): Due to the selected inclusion and exclusion criteria, such as proximal vessel occlusion, a narrow therapeutic time window (six hours) after symptom onset or – in case of wake-up or unknown onset stroke – after symptom recognition, small infarct core at screening, and enrolment of (older) patients only in the case that TBY is likely to be conducted, all patients included in PROOF will likely be treated by endovascular mechanical thrombectomy (TBY) ± intravenous thrombolysis, in which for > 80% of cases complete brain tissue reperfusion is reached, thus achieving transient ischemia, which is deemed necessary for successful oxygen therapy as concluded from animal experiments [109].	**How does the PROOF trial differ?** The PROOF study is the first human NBHO trial to consider all three framework requirements for a successful "freezing of the penumbra" and lasting conservation of positive effects through: (1) early reperfusion (compare Fig. 1 and final infarct versus failed recanalization in Fig. 3): Due to the selected inclusion and exclusion criteria, such as proximal vessel occlusion, a narrow therapeutic time window (six hours) after symptom onset or – in case of wake-up or unknown onset stroke – after symptom recognition, small infarct core at screening, and enrolment of (older) patients only in the case that TBY is likely to be conducted, all patients included in PROOF will likely be treated by endovascular mechanical thrombectomy (TBY) ± intravenous thrombolysis, in which for > 80% of cases complete brain tissue reperfusion is reached, thus achieving transient ischemia, which is deemed necessary for	Adaption according to current ESMINT/ESO and AHA/ASA guidelines [159, 160] will also facilitate recruitment. In the DAWN and the DEFUSE 3 randomized controlled trials, endovascular mechanical thrombectomy was clearly beneficial in patients with wake-up stroke/unknown onset stroke due to significant volumes of salvageable ischemic brain tissue [106][107]. Consequently, beneficial effects of NBHO can equally be assumed in wake-up stroke/unknown onset stroke patients with small ischemic core at baseline as per PROOF inclusion criteria. In order to avoid any imbalance, we adapted variable used in minimization (see 8.5.1; "Time window known < 6h vs. unknown/wake-up")

Previous and new wording in track change modus	New wording	Comments/ reasons for substantial amendment
	successful oxygen therapy as concluded from animal experiments [109].	
**4.3.1 Risk-Benefit Assessment Considering the Literature until December 6th, 2019 (page 35)**		
**Rapid start of oxygen therapy within the hospital environment**<br>Fig. 2 and Fig. 3 suggest a pre-hospital start of oxygen therapy at the earliest possible time point. Even though this may seem safe [118], in the PROOF trial we begin oxygen therapy in the hospital environment as soon as possible after cerebral imaging and at the latest ~~three~~ six hours after onset of symptoms or – in case of wake-up or unknown onset stroke – after symptom recognition: continuous clinical and technical monitoring by an experienced expert team of stroke specialists within the hospital environment guarantees maximum patient safety.	**Rapid start of oxygen therapy within the hospital environment**<br>Fig. 2 and Fig. 3 suggest a pre-hospital start of oxygen therapy at the earliest possible time point. Even though this may seem safe [118], in the PROOF trial we begin oxygen therapy in the hospital environment as soon as possible after cerebral imaging and at the latest six hours after onset of symptoms or – in case of wake-up or unknown onset stroke – after symptom recognition: continuous clinical and technical monitoring by an experienced expert team of stroke specialists within the hospital environment guarantees maximum patient safety.	Correction of clerical error; the six hour inclusion window has already been an adaption made in protocol version 1.3<br><br>Adaption according to current ESMINT/ESO and AHA/ASA guidelines [159, 160] will also facilitate recruitment. In the DAWN and the DEFUSE 3 randomized controlled trials, endovascular mechanical thrombectomy was clearly beneficial in patients with wake-up stroke/unknown onset stroke due to significant volumes of salvageable ischemic brain tissue [106][107]. Consequently, beneficial effects of NBHO can equally be assumed in wake-up stroke/unknown onset stroke patients with small ischemic core at baseline as per PROOF inclusion criteria. In order to avoid any imbalance, we adapted variable used in minimization (see 8.5.1; “Time window known < 6h vs. unknown/wake-up”)
**4.3.1 Risk-Benefit Assessment Considering the Literature until December 6th, 2019 (page 40)**		

Previous and new wording in track change modus	New wording	Comments/ reasons for substantial amendment
In order to render trial participation in PROOF as safe as possible for study patients we have expanded the DSMB Charter according to the Tübingen University Hospital ethics committee's (protocol version 1.3) and the DSMB's requests (protocol version 1.4) to continuously monitor the mortality rate and the rates of parenchymal hematoma and remote intracranial bleeding (see Appendix 14 for Heidelberg bleeding classification [101]), respectively. This means that the DSMB and the sponsor will be simultaneously informed of every death, every parenchymal hematoma, and every remote intracranial bleeding.	In order to render trial participation in PROOF as safe as possible for study patients we have expanded the DSMB Charter according to the Tübingen University Hospital ethics committee's (protocol version 1.3) and the DSMB's requests (protocol version 1.4) to continuously monitor the mortality rate and the rates of parenchymal hematoma and remote intracranial bleeding (see Appendix 14 for Heidelberg bleeding classification [101]), respectively. This means that the DSMB and the sponsor will be simultaneously informed of every death, every parenchymal hematoma, and every remote intracranial bleeding.	Intensified safety monitoring has been implemented according to DSMB's requirements.
**4.3.1 Risk-Benefit Assessment Considering the Literature until December 6th, 2019 (page 40)**		
To enable a "security check" in PROOF even before a decision regarding a possible therapy limitation is made, we have decided to implement an additional frequent (i.e. at every DSMB meeting, see Section 4.4 Data and Safety Monitoring Board) evaluation of early neurological change (delta NIHSS from screening to V2~~prior to study intervention to 20 minutes~~) after the start of oxygen therapy (in the intervention arm) in connection with the primary end point, the infarct growth from prior to study intervention to 24 hours, in the DSMB Charter, so to be able to immediately detect potentially outcome-relevant short-term influences of oxygen therapy on e.g. cerebral perfusion and possibly end the study prematurely.	To enable a "security check" in PROOF even before a decision regarding a possible therapy limitation is made, we have decided to implement an additional frequent (i.e. at every DSMB meeting, see Section 4.4 Data and Safety Monitoring Board) evaluation of early neurological change (delta NIHSS from screening to V2) after the start of oxygen therapy (in the intervention arm) in connection with the primary end point, the infarct growth from prior to study intervention to 24 hours, in the DSMB Charter, so to be able to immediately detect potentially outcome-relevant short-term influences of oxygen therapy on e.g. cerebral perfusion and possibly end the study prematurely.	Revision to reflect changes of amended protocol V1.4, i.e., time window of V2 has been slightly widened (from 10 min to 5 min after NBHO/randomization until start of endovascular intervention) for enhanced flexibility in order to avoid interference with hyperacute stroke work-up and facilitate conduction of V2.
**4.3.1 Risk-Benefit Assessment Considering the Literature until December 6th, 2019 (page 41)**		
~~Only very recently the results of the **New Zealand Oxygen in Acute Coronary Syndromes Trial** (NZOTACS) have been presented at the annual~~	40,872 patients with suspected ACS were enrolled into the **New Zealand Oxygen in Acute Coronary Syndromes Trial** (NZOTACS) [102].	The NZOTACS trial has been published in the British Medical Journal on March 2nd, 2021 [102]. Accordingly, we updated the

Previous and new wording in track change modus	New wording	Comments/ reasons for substantial amendment
congress of the European Society of Cardiology (ESC, Aug 29th to Sep 2nd 2019 in Paris, France) [102]. 40,872 patients with suspected ACS were enrolled into the **New Zealand Oxygen in Acute Coronary Syndromes Trial** (NZOTACS) [102].		sentence and the respective reference without any change of content.
**4.3.2 Updated Risk-Benefit Assessment Considering the Literature until March 17th, 2021 (pages 46/47)**		
A PubMed search on March 17th, 2021, using the criteria ("oxygen therapy" OR "normobaric oxygen") AND ("acute ischemic stroke" OR “acute stroke”) revealed 5 new publications since December 6, 2019.<br><br>Of these, two were review articles on hyperbaric oxygen therapy [147, 148], and not NBHO which is the treatment that is applied in PROOF. Furthermore, no recent studies were included in the review articles. Consequently, neither review article contributes to the risk-benefit assessment of the PROOF trial.<br><br>One experimental study reported NBHO mediated reduction of hyperglycolysis through modulation of the adenosine monophosphate-activated protein kinase signaling pathway, and thereby alleviation of oxidative injury in a rat model of acute ischemic stroke [149].<br><br>In line with two previous retrospective studies (N=2643 and N=554, see Section 4.3 Risk-benefit Assessment) [87, 88], one retrospective study on early oxygenation levels in 1479 acute ischemic stroke patients showed association of lower SpO_2/FiO_2 levels in the first few ours of admission and mortality, however, no association for high SpO_2 (99% to 100%) compared to normal SpO_2 (96% to 98%) [150].	A PubMed search on March 17th, 2021, using the criteria ("oxygen therapy" OR "normobaric oxygen") AND ("acute ischemic stroke" OR “acute stroke”) revealed 5 new publications since December 6, 2019.<br><br>Of these, two were review articles on hyperbaric oxygen therapy [147, 148], and not NBHO which is the treatment that is applied in PROOF. Furthermore, no recent studies were included in the review articles. Consequently, neither review article contributes to the risk-benefit assessment of the PROOF trial.<br><br>One experimental study reported NBHO mediated reduction of hyperglycolysis through modulation of the adenosine monophosphate-activated protein kinase signaling pathway, and thereby alleviation of oxidative injury in a rat model of acute ischemic stroke [149].<br><br>In line with two previous retrospective studies (N=2643 and N=554, see Section 4.3 Risk-benefit Assessment) [87, 88], one retrospective study on early oxygenation levels in 1479 acute ischemic stroke patients showed association of lower SpO_2/FiO_2 levels in the first few ours of admission and mortality, however, no association for high SpO_2 (99% to 100%) compared to normal SpO_2 (96% to 98%) [150].	Updated risk-benefit assessment considering the most recent publications. Overall, no safety concerns arise, and potential neuroprotective effects of NBHO are strongly supported by a recent randomized controlled study [151].

Previous and new wording in track change modus	New wording	Comments/ reasons for substantial amendment
Last but not least, in an RCT [151], in which 180 acute ischemic stroke patients who underwent successful recanalization of an anterior circulation LVO within 6 hours of symptom onset were enrolled, and that compared immediate post-reperfusion high-flow oxygen (15 L/min over 6 hours) with routine low-flow oxygen supplementation (3 L/min), NBHO treatment improved functional outcomes (common odds ratio 2.2 [95% CI 1.26 – 3.87] favoring the distribution of global disability scores on the mRS at 90 days), and reduced mortality at 90 days (13.9% absolute difference, rate ratio 0.35 [95% CI 0.13 – 0.93]) as well as infarct volumes as determined by MRI (median infarct volume 9.4 mL vs. 20.5 mL in the control group, beta coefficient -20.24 [95% CI -35.93 – -4.55]). No significant differences were seen in the rate of symptomatic ICH, pneumonia, urinary infection, and seizures between the two treatment groups [151].<br>Although this RCT did not test the oxygen treatment approach that is evaluated in PROOF (i.e. penumbral freezing by NBHO until reperfusion), it confirms the results of the few experimental studies that tested NBHO in the post-reperfusion phase [54, 71, 149, 152], strongly mitigates concerns that oxygen might aggravate reperfusion injury when continued after successful reperfusion, e.g. by an – hypothesized but never confirmed [70] – increase of radical oxygen species in the previously ischemic and, thus, particularly vulnerable brain tissue, and, most importantly, underlines the DSMB's judgment that hemorrhagic infarction which has been observed in 10 (25%) of the first 40 PROOF patients were most likely unrelated to treatment with NBHO. Importantly, the number of PROOF patients who suffered from any kind of hemorrhagic infarction (10/40) or	Last but not least, in an RCT [151], in which 180 acute ischemic stroke patients who underwent successful recanalization of an anterior circulation LVO within 6 hours of symptom onset were enrolled, and that compared immediate post-reperfusion high-flow oxygen (15 L/min over 6 hours) with routine low-flow oxygen supplementation (3 L/min), NBHO treatment improved functional outcomes (common odds ratio 2.2 [95% CI 1.26 – 3.87] favoring the distribution of global disability scores on the mRS at 90 days), and reduced mortality at 90 days (13.9% absolute difference, rate ratio 0.35 [95% CI 0.13 – 0.93]) as well as infarct volumes as determined by MRI (median infarct volume 9.4 mL vs. 20.5 mL in the control group, beta coefficient -20.24 [95% CI -35.93 – -4.55]). No significant differences were seen in the rate of symptomatic ICH, pneumonia, urinary infection, and seizures between the two treatment groups [151].<br>Although this RCT did not test the oxygen treatment approach that is evaluated in PROOF (i.e. penumbral freezing by NBHO until reperfusion), it confirms the results of the few experimental studies that tested NBHO in the post-reperfusion phase [54, 71, 149, 152], strongly mitigates concerns that oxygen might aggravate reperfusion injury when continued after successful reperfusion, e.g. by an – hypothesized but never confirmed [70] – increase of radical oxygen species in the previously ischemic and, thus, particularly vulnerable brain tissue, and, most importantly, underlines the DSMB's judgment that hemorrhagic infarction which has been observed in 10 (25%) of the first 40 PROOF patients were most likely unrelated to treatment with NBHO. Importantly, the number of PROOF patients who suffered from any kind of hemorrhagic infarction (10/40) or	

Previous and new wording in track change modus	New wording	Comments/ reasons for substantial amendment
symptomatic ICH (3/10) is so far within the range that can be expected in the trials' patient population. Anterior circulation ischemic stroke patients who undergo endovascular thrombectomy for treatment of the underlying acute large vessel occlusion represent one of the most severely affected subgroups of ischemic stroke and are at high risk of large infarcts and reperfusion injury. Similar or even higher numbers of ICH have been reported in recent publications, e.g., 31.9% any ICH and 4.4% symptomatic ICH in [153].<br><br>A second PubMed search on March 17th, 2021, using the criteria ("oxygen therapy" OR "normobaric oxygen") AND ("acute myocardial infarction"), which was conducted in order to gather further data on safety in a patient population with similar age, vascular risk factors and comorbidities, revealed 6 new publications since December 6, 2019, including three publications of relevance that reported results of prospective studies evaluating normobaric hyperoxygenation.<br><br>First, the results of the NZOTACS trial which we had already discussed at the very end of Section 4.3.1 [102].<br><br>Second, 1-year clinical outcomes of the 100 anterior STEMI patients who were enrolled into the single-arm IC-HOT study and treated with PCI followed by a 60-min infusion of hyperoxygenated blood (760 to 1000 mmHg) into the left main coronary artery better compared to that of propensity score matched controls: both, the composite endpoint of all-cause death, new-onset heart failure, or hospitalization for heart failure (0.0% vs. 12.3%, p = 0.001), as well as each individually were lower in IC-HOT patients [154]. There were no significant differences between groups	symptomatic ICH (3/10) is so far within the range that can be expected in the trials' patient population. Anterior circulation ischemic stroke patients who undergo endovascular thrombectomy for treatment of the underlying acute large vessel occlusion represent one of the most severely affected subgroups of ischemic stroke and are at high risk of large infarcts and reperfusion injury. Similar or even higher numbers of ICH have been reported in recent publications, e.g., 31.9% any ICH and 4.4% symptomatic ICH in [153].<br><br>A second PubMed search on March 17th, 2021, using the criteria ("oxygen therapy" OR "normobaric oxygen") AND ("acute myocardial infarction"), which was conducted in order to gather further data on safety in a patient population with similar age, vascular risk factors and comorbidities, revealed 6 new publications since December 6, 2019, including three publications of relevance that reported results of prospective studies evaluating normobaric hyperoxygenation.<br><br>First, the results of the NZOTACS trial which we had already discussed at the very end of Section 4.3.1 [102].<br><br>Second, 1-year clinical outcomes of the 100 anterior STEMI patients who were enrolled into the single-arm IC-HOT study and treated with PCI followed by a 60-min infusion of hyperoxygenated blood (760 to 1000 mmHg) into the left main coronary artery better compared to that of propensity score matched controls: both, the composite endpoint of all-cause death, new-onset heart failure, or hospitalization for heart failure (0.0% vs. 12.3%, p = 0.001), as well as each individually were lower in IC-HOT patients [154]. There were no significant differences between groups	

Previous and new wording in track change modus	New wording	Comments/ reasons for substantial amendment
in the 1-year rates of reinfarction or clinically driven target vessel revascularization [154].<br><br>Third, results of the pre-specified subgroup analysis of patients with COPD (n=296) who had been enrolled into the large randomized controlled DETOX-AMI trial (N=6629) and treated with either oxygen or ambient air resemble the main trial results showing no benefit but also support safety of NBHO even in the presence of pulmonary disease [155].	in the 1-year rates of reinfarction or clinically driven target vessel revascularization [154].<br><br>Third, results of the pre-specified subgroup analysis of patients with COPD (n=296) who had been enrolled into the large randomized controlled DETOX-AMI trial (N=6629) and treated with either oxygen or ambient air resemble the main trial results showing no benefit but also support safety of NBHO even in the presence of pulmonary disease [155].	
**4.3.3 Risk-Benefit Assessment of the PROOF trial during the COVID-19 pandemic (page 47)**		
Patients who are enrolled in the PROOF trial suffer from acute ischemic stroke and require immediate emergency treatment independently of trial participation. During emergency transport and hospital admission, respective national and hospital regulations are respected in order to minimize the risk of a SARS-CoV-2 transmission. Preventive measures may include wearing medical face masks, face shields/goggle, plastic aprons and gloves as well as the conduct of rapid antigen tests in patients. Another measure to stop the COVID-19 pandemic are SARS-CoV-2 vaccines, which have just been approved in Europe. These vaccines are becoming increasingly available to medical staff and elderly people; however, the vaccination scheme might differ between the countries involved in the PROOF trial.<br><br>There is no additional study-specific risk of spreading the SARS-CoV-2 virus in patients randomized into the control group, as these patients solely receive standard care (treatment as usual). Due to higher than usual oxygen flowrates, NBHO might increase the formation of potentially SARS-CoV-2	Patients who are enrolled in the PROOF trial suffer from acute ischemic stroke and require immediate emergency treatment independently of trial participation. During emergency transport and hospital admission, respective national and hospital regulations are respected in order to minimize the risk of a SARS-CoV-2 transmission. Preventive measures may include wearing medical face masks, face shields/goggle, plastic aprons and gloves as well as the conduct of rapid antigen tests in patients. Another measure to stop the COVID-19 pandemic are SARS-CoV-2 vaccines, which have just been approved in Europe. These vaccines are becoming increasingly available to medical staff and elderly people; however, the vaccination scheme might differ between the countries involved in the PROOF trial.<br><br>There is no additional study-specific risk of spreading the SARS-CoV-2 virus in patients randomized into the control group, as these patients solely receive standard care (treatment as usual). Due to higher than usual oxygen flowrates, NBHO might increase the formation of potentially SARS-CoV-2	Insertion of risk-benefit-assessment to show that the COVID-19 pandemic does not interfere with the conduct of the trial and the patients' health will be not endangered when taking part.

Previous and new wording in track change modus	New wording	Comments/ reasons for substantial amendment
contaminated aerosol. Thus, wearing FFP2/3 face masks according to national and local regulations during NBHO is strongly recommended to PROOF investigators.<br><br>All PROOF study visits but V7 are conducted during the inpatient stay, and a phone interview might replace V7 in-person visit, so that the enrolled patients and the medical staff are not exposed to a greater risk of SARS-CoV-2 infection compared to routine treatment. Besides, participation in the PROOF trial does not interfere with COVID-19 related travel restrictions.	contaminated aerosol. Thus, wearing FFP2/3 face masks according to national and local regulations during NBHO is strongly recommended to PROOF investigators.<br><br>All PROOF study visits but V7 are conducted during the inpatient stay, and a phone interview might replace V7 in-person visit, so that the enrolled patients and the medical staff are not exposed to a greater risk of SARS-CoV-2 infection compared to routine treatment. Besides, participation in the PROOF trial does not interfere with COVID-19 related travel restrictions.	
**4.4 Data and Safety Monitoring Board (DSMB) (page 48)**		
**Role:** The DSMB ensures the ethical conduct of the trial and protects the rights and welfare of the patients. All deaths, parenchymal hematoma and remote intracranial bleeding (see Appendix 14 for Heidelberg bleeding classification [101]) will be reported expedited to the DSMB. After validation of the expedited reported bleeding events by Eppdata (core imaging laboratory), according patient narratives are prepared in a timely manner by the concerned trial site and provided to the DSMB. In addition, tThe DSMB will receive periodic blinded and – upon request – un-blinded reports of clinical trial entry, accrual and drop-outs (withdrawals and lost to follow-ups), adverse events and any other relevant data from the clinical trial as well as the Brain Imaging and outcome assessment (WP 5).	**Role:** The DSMB ensures the ethical conduct of the trial and protects the rights and welfare of the patients. All deaths, parenchymal hematoma and remote intracranial bleeding (see Appendix 14 for Heidelberg bleeding classification [101]) will be reported expedited to the DSMB. After validation of the expedited reported bleeding events by Eppdata (core imaging laboratory), according patient narratives are prepared in a timely manner by the concerned trial site and provided to the DSMB. In addition, the DSMB will receive periodic blinded and – upon request – un-blinded reports of clinical trial entry, accrual and drop-outs (withdrawals and lost to follow-ups), adverse events and any other relevant data from the clinical trial as well as the Brain Imaging and outcome assessment (WP 5).	Intensified safety monitoring has been implemented according to DSMB's requirements.
**Meetings:** The DSMB will meet at 6-monthly intervals via teleconference to review and evaluate the quality of data collected during the clinical trial and assess reports on (serious) adverse events (all SAE, all non-serious AEs with a causal relationship to the IMP, all	**Meetings:** The DSMB will meet at 6-monthly intervals via teleconference to review and evaluate the quality of data collected during the clinical trial and assess reports on (serious) adverse events (all SAE, all non-serious AEs with a causal relationship to the IMP, all	Intensified safety monitoring has been implemented according to DSMB's requirements.

Previous and new wording in track change modus	New wording	Comments/ reasons for substantial amendment
parenchymal hematoma and remote intracranial bleeding, and cumulated other non-serious AEs) as per the DSMB charter developed for the clinical trial and provide advices to the SC.	parenchymal hematoma and remote intracranial bleeding, and cumulated other non-serious AEs) as per the DSMB charter developed for the clinical trial and provide advices to the SC.	
**4.5. Steering Committee (SC) (page 48)**		
**Composition:** the SC is chaired by the Coordinator and notably comprises all Work Package (WP) leaders<br>• Holm Graessner (EKUT), leader of WP2 (Coordination and innovation management) (Deputy: Monika Glauch)<br>• Sven Poli (EKUT), Coordinator leader of WP3 (Trial preparation) (Deputy: Johannes TünnerhoffFlorian Härtig)<br>• Johannes Hüsing (UKL-HD), responsible biometrician (Deputy: Maike Nilsson (UKL-HD), leader of WP4 (Performance of the clinical trial))<br>• Frosti Palsson (Eppdata), leader of WP5 (Brain Imaging and outcome assessments) (Deputy: Jens Fiehler)<br>• Joan Montaner (VHIR), leader of WP6 (Biomarkers) (Deputy: Alejandro Bustamante)	**Composition:** the SC is chaired by the Coordinator and notably comprises all Work Package (WP) leaders<br>• Holm Graessner (EKUT), leader of WP2 (Coordination and innovation management) (Deputy: Monika Glauch)<br>• Sven Poli (EKUT), Coordinator leader of WP3 (Trial preparation) (Deputy: Johannes Tünnerhoff)<br>• Johannes Hüsing (UKL HD), responsible biometrician (Deputy: Maike Nilsson (UKL-HD), leader of WP4 (Performance of the clinical trial))<br>• Frosti Palsson (Eppdata), leader of WP5 (Brain Imaging and outcome assessments) (Deputy: Jens Fiehler)<br>• Joan Montaner (VHIR), leader of WP6 (Biomarkers) (Deputy: Alejandro Bustamante)	Change of staff
**5.1 Primary objective and Primary Endpoint (page 50)**		
**Primary endpoint:** Efficacy of NBHO treatment will be determined by an ITT analysis of ischemic core growth defined as the difference in ischemic core volume (in mL) from baseline to 24 hours; brain tissue not included in CT perfusion (or, in case CT perfusion is not available or of insufficient quality, CT angiography source images) or MR diffusion at baseline will be excluded from lesion volume measurements. Two independent neuroradiologists blinded for treatment allocation will assess ischemic core volume.	**Primary endpoint:** Efficacy of NBHO treatment will be determined by an ITT analysis of ischemic core growth defined as the difference in ischemic core volume (in mL) from baseline to 24 hours; brain tissue not included in CT perfusion (or, in case CT perfusion is not available or of insufficient quality, CT angiography source images) or MR diffusion at baseline will be excluded from lesion volume measurements. Two independent neuroradiologists blinded for treatment allocation will assess ischemic core volume.	Adaptions according to current ESMINT/ESO and AHA/ASA guidelines [159, 160] in which perfusion imaging is only obligatory for specific subgroups such as patients with wake-up or unknown onset strokes, but not in patients with known stroke onset <6h. In cases no perfusion imaging will be available, CT angiography source images will be used for ischemic core estimation at baseline (see Chapter 10.4). This adaption will not only facilitate enrollment but also compensate for drop-outs due to insufficient quality of

Previous and new wording in track change modus	New wording	Comments/ reasons for substantial amendment
		perfusion imaging, which has been observed in ~10% of enrolled cases.
**5.2 Secondary Objectives and Secondary Endpoints (pages 50/51)**		
**Secondary clinical efficacy endpoints (~~clinical assessment~~NIHSS at V5 and mRS at V7 are conducted by a blinded rater, see 8.5.2 Blinding procedure):**<br>• Survival at V6 and V7;<br>• NIHSS at V2, V4*, V5*, V6*, V7; *with and without exclusion of patients still under sedation or anesthesia<br>• Stroke subtype classification at V6 or V7;<br>• mRS at V6 and V7;<br>• Barthel Index, taken at V6 and V7;<br>• MoCA at V7 (controlled for IQCODE at V5);<br>• SIS-16 at V7;<br>• EQ-5D-5L at V7;<br>• MADRS at V7;<br>• PaO_2 at V3 and V5;	**Secondary clinical efficacy endpoints (NIHSS at V5 and mRS at V7 are conducted by a blinded rater, see 8.5.2 Blinding procedure):**<br>• Survival at V6 and V7;<br>• NIHSS at V2, V4*, V5*, V6*, V7; *with and without exclusion of patients still under sedation or anesthesia<br>• Stroke subtype classification at V6 or V7;<br>• mRS at V6 and V7;<br>• Barthel Index, taken at V6 and V7;<br>• MoCA at V7 (controlled for IQCODE at V5);<br>• SIS-16 at V7;<br>• EQ-5D-5L at V7;<br>• MADRS at V7;<br>• PaO_2 at V3 and V5;	In order to enhance feasibility of study conduction, decision was made to focus blinded secondary clinical endpoint assessment solely on the NIHSS at V5, which is the key secondary endpoint of the PROOF study, and the modified Rankin Scale score at V7, which represents the most commonly used clinical endpoint in (phase 3) stroke studies.<br><br>In order to account for confounding effects of sedation/ anaesthesia on neurological status, especially during and shortly after endovascular thrombectomy, we will conduct NIHSS analysis with and without exclusion of sedated/anaesthetized patients
Secondary imaging efficacy endpoints:<br>• relative changes in ischemic core volume (in %) from baseline to 24 hours;<br>• absolute and relative ischemic core change from baseline to 24 hours using either NCCT or DWI-MRI (or CT~~/MR~~ angiography source images and DWI) for ischemic core estimation at baseline;<br>• absolute and relative ischemic core change from baseline to 24 hours using cerebral blood flow (CBF) < 30% for ischemic core estimation at baseline in all patients, independent of imaging modality;	Secondary imaging efficacy endpoints:<br>• relative changes in ischemic core volume (in %) from baseline to 24 hours;<br>• absolute and relative ischemic core change from baseline to 24 hours using either NCCT or DWI-MRI (or CT angiography source images and DWI) for ischemic core estimation at baseline;<br>• absolute and relative ischemic core change from baseline to 24 hours using cerebral blood flow (CBF) < 30% for ischemic core estimation at baseline in all patients, independent of imaging modality;	Correction of clerical error: MR angiography was never planned for ischemic core estimation. DWI is the correct modality.<br><br>Clarification by adding “source images”: because only CT angiography source images but not reconstructions of CT angiography can be used for core estimation.

Previous and new wording in track change modus	New wording	Comments/ reasons for substantial amendment
• penumbral salvage from baseline to 24 hours;<br>• TICI (Thrombolysis in Cerebral Infarction perfusion scale grade) in patients who underwent mechanical thrombectomy (TBY);<br>• revascularization on 24-hour follow-up imaging.	• penumbral salvage from baseline to 24 hours;<br>• TICI (Thrombolysis in Cerebral Infarction perfusion scale grade) in patients who underwent mechanical thrombectomy (TBY);<br>revascularization on 24-hour follow-up imaging	
**6.1 Trial design (page 52)**		
**Intervention arm:** NBHO (i.e. inhalation of 100% oxygen at high flow (≥ 40 L/min) via a sealed non-rebreather face-mask with reservoir, or in case of intubation/ventilation for (study-independent) TBY, ventilation with an inspiratory oxygen fraction (FiO_2) of 1.0) started within 6 hours after certain stroke symptom onset (witnessed~~ or last seen well~~) or after symptom recognition (in case of wake-up or unknown onset stroke), and within 30 minutes after end of baseline brain imaging and applied until the end of TBY procedure (defined by removal of guide catheter from sheath) or, in case TBY is not attempted (defined as 'TBY was not attempted or intervention was stopped prior to any penetration or aspiration of the qualifying (i.e. intracranial) LVO'), 4 hours after start of study treatment.	**Intervention arm:** NBHO (i.e. inhalation of 100% oxygen at high flow (≥ 40 L/min) via a sealed non-rebreather face-mask with reservoir, or in case of intubation/ventilation for (study-independent) TBY, ventilation with an inspiratory oxygen fraction (FiO_2) of 1.0) started within 6 hours after certain stroke symptom onset (witnessed) or after symptom recognition (in case of wake-up or unknown onset stroke), and within 30 minutes after end of baseline brain imaging and applied until the end of TBY procedure (defined by removal of guide catheter from sheath) or, in case TBY is not attempted (defined as 'TBY was not attempted or intervention was stopped prior to any penetration or aspiration of the qualifying (i.e. intracranial) LVO'), 4 hours after start of study treatment.	Adaption according to current ESMINT/ESO and AHA/ASA guidelines [159, 160] will also facilitate recruitment. In the DAWN and the DEFUSE 3 randomized controlled trials, endovascular mechanical thrombectomy was clearly beneficial in patients with wake-up stroke/unknown onset stroke due to significant volumes of salvageable ischemic brain tissue [106][107]. Consequently, beneficial effects of NBHO can equally be assumed in wake-up stroke/unknown onset stroke patients with small ischemic core at baseline as per PROOF inclusion criteria. In order to avoid any imbalance, we adapted variable used in minimization (see 8.5.1; "Time window known < 6h vs. unknown/wake-up")
**6.2 Trial Duration and Schedule (page 52)**		
The duration of the trial for each subject is expected to be three months (from enrolment within 6 hours of acute ischemic stroke (or symptom recognition in case of wake-up or unknown onset stroke) to day 90 follow-up).	The duration of the trial for each subject is expected to be three months (from enrolment within 6 hours of acute ischemic stroke (or symptom recognition in case of wake-up or unknown onset stroke) to day 90 follow-up).	Adaption according to current ESMINT/ESO and AHA/ASA guidelines [159, 160] will also facilitate recruitment. In the DAWN and the DEFUSE 3 randomized controlled trials, endovascular mechanical thrombectomy was clearly beneficial in patients with wake-up stroke/unknown onset stroke due to significant volumes of salvageable ischemic

Previous and new wording in track change modus	New wording	Comments/ reasons for substantial amendment
		brain tissue [106][107]. Consequently, beneficial effects of NBHO can equally be assumed in wake-up stroke/unknown onset stroke patients with small ischemic core at baseline as per PROOF inclusion criteria. In order to avoid any imbalance, we adapted variable used in minimization (see 8.5.1; “Time window known < 6h vs. unknown/wake-up”)
Total trial duration: ~~60~~ 84 months<br>Duration of clinical phase: ~~36~~ 48 months<br>Beginning of the preparation phase: Q1 2017<br>FSI (first subject in): August 2019<br>LSI (last subject in): Q1 2023~~1~~<br>LSO (last subject out): Q2 2023~~1~~<br>DBL (database lock): Q3 2023~~1~~<br>Statistical analyses completed: Q4 2023~~1~~<br>Trial report completed: Q4 2023~~1~~	Total trial duration: 84 months<br>Duration of clinical phase: 48 months<br>Beginning of the preparation phase: Q1 2017<br>FSI (first subject in): August 2019<br>LSI (last subject in): Q1 2023<br>LSO (last subject out): Q2 2023<br>DBL (database lock): Q3 2023<br>Statistical analyses completed: Q4 2023<br>Trial report completed: Q4 2023	Adaption according to current time line. Delays were due to unforeseeable administrative hurdles, Covid-19 pandemic and lastly critical DSMB evaluation as well as low recruitment. Adaptions of inclusion/exclusion criteria will not only enhance generalizability of results but further increase feasibility of study conduction and thereby, guarantee sufficient recruitment and – at least partly – to recover the gap: 92 (48%), 46 (24%), and 22 (12%) of a total of 190 patients who underwent endovascular mechanical thrombectomy due to acute anterior circulation large vessel occlusion at EKUT in between July 2 2019 and Feb 6 2021 might have been enrolled as per inclusion and exclusion criteria of protocol versions 1.4, 1.3, and 1.2, respectively.
**7.2 Centers (page 53)**		
The study will be conducted on a multinational and multicenter basis.<br>Recruitment and treatment of subjects will be performed in clinical trial centers in the following countries: Germany, Belgium, the Czech Republic, Finland, France, Spain~~, Sweden~~ and Switzerland.	The study will be conducted on a multinational and multicenter basis.<br>Recruitment and treatment of subjects will be performed in clinical trial centers in the following countries: Germany, Belgium, the Czech Republic, Finland, France, Spain and Switzerland.	Removal of Sweden, because the country is not participating in the PROOF trial.

Previous and new wording in track change modus	New wording	Comments/ reasons for substantial amendment
**7.3 General Criteria for Subject's Selection (page 53)**		
The PROOF trial will include previously independent acute anterior circulation ischemic stroke patients (due to LVO) within a time window of ~~three~~ six hours from symptom onset or, in case of wake-up or unknown onset stroke, symptom recognition to initiation of study treatment.	The PROOF trial will include previously independent acute anterior circulation ischemic stroke patients (due to LVO) within a time window of six hours from symptom onset or, in case of wake-up or unknown onset stroke, symptom recognition to initiation of study treatment.	Correction of clerical error; the six-hour inclusion window has already been an adaption made in protocol version 1.3<br><br>Adaption according to current ESMINT/ESO and AHA/ASA guidelines [159, 160] will also facilitate recruitment. In the DAWN and the DEFUSE 3 randomized controlled trials, endovascular mechanical thrombectomy was clearly beneficial in patients with wake-up stroke/unknown onset stroke due to significant volumes of salvageable ischemic brain tissue [106][107]. Consequently, beneficial effects of NBHO can equally be assumed in wake-up stroke/unknown onset stroke patients with small ischemic core at baseline as per PROOF inclusion criteria. In order to avoid any imbalance, we adapted variable used in minimization (see 8.5.1; "Time window known < 6h vs. unknown/wake-up")
**7.4 Inclusion Criteria (page 53)**		
• Age: ~~>~~ ≥ 18 years	• Age: ≥ 18 years	Correction of a clerical error: all adult patients including 18-year-old patients may be enrolled as it was previously the case in protocol version 1.2
• ASPECTS of 6~~7~~-10 on NCCT or ~~6~~5-10 on DWI-MRI	• ASPECTS of 6-10 on NCCT or 5-10 on DWI-MRI	Today, ASPECTS of 6 on NCCT (or 5 on DWI-MRI) are routinely treated with endovascular mechanical thrombectomy. High treatment effects due to significant volumes of salvageable ischemic brain tissue (Goyal et la., Lancet 2016) indicate that beneficial effects of NBHO can equally be assumed in patients with an – still high enough – ASPECTS of 6 on NCCT (or 5 on

Previous and new wording in track change modus	New wording	Comments/ reasons for substantial amendment
		DWI-MRI). This adaption had already been pre-specified in the previous protocol version for facilitate recruitment (see Chapter 7.6 of protocol version 1.3).
• If recommended by the attending physician, CT or MR perfusion ~~(whole-brain or minimal coverage ≥ 75 mm)~~should be performed prior to NBHO	• If recommended by the attending physician, CT or MR perfusion should be performed prior to NBHO	According to current guidelines perfusion imaging is only obligatory for specific subgroups such as patients with wake-up or unknown onset strokes, but not in patients with known stroke onset <6h. In cases no perfusion imaging will be available, CT angiography source images will be used for ischemic core estimation at baseline (see Chapter 10.4)
• NBHO can be initiated within 6 hours of symptom onset (witnessed~~ or last seen well~~) or symptom recognition (in case of wake-up or unknown onset stroke), and within 30 minutes after last image of baseline brain imaging	• NBHO can be initiated within 6 hours of symptom onset (witnessed) or symptom recognition (in case of wake-up or unknown onset stroke), and within 30 minutes after last image of baseline brain imaging	Adaption according to current ESMINT/ESO and AHA/ASA guidelines [159, 160] will also facilitate recruitment. In the DAWN and the DEFUSE 3 randomized controlled trials, endovascular mechanical thrombectomy was clearly beneficial in patients with wake-up stroke/unknown onset stroke due to significant volumes of salvageable ischemic brain tissue [106][107]. Consequently, beneficial effects of NBHO can equally be assumed in wake-up stroke/unknown onset stroke patients with small ischemic core at baseline as per PROOF inclusion criteria. In order to avoid any imbalance, we adapted variable used in minimization (see 8.5.1; “Time window known < 6h vs. unknown/wake-up”)
• Pre-stroke mRS of 0 ~~or 1~~to 2	• Pre-stroke mRS of 0 to 2	Today, patients with a pre-stroke modified Rankin Scale (mRS) score of 2 are routinely treated with endovascular mechanical thrombectomy. Mild pre-existing disability (as signified by the mRS of 2) did not limit recovery after stroke (Goyal et la., Lancet

Previous and new wording in track change modus	New wording	Comments/ reasons for substantial amendment
		2016). This adaption had already been pre-specified in the previous protocol version for facilitate recruitment (see Chapter 7.6 of protocol version 1.3).
**7.5 Exclusion Criteria, Neurological (page 53)**		
• Rapid major improvement in neurological status* prior to randomization (*in case but NIHSS score remains ≥ 6, enrolment might still be possible if persistent LVO is confirmed on repeated vessel imaging*)	• Rapid major improvement in neurological status* prior to randomization (*in case but NIHSS score remains ≥ 6, enrolment might still be possible if persistent LVO is confirmed on repeated vessel imaging*)	We added this explanation in order to make clear to the investigators that in case of rapid major neurological improvement, enrollment is only allowed in case of proven persisting large vessel occlusion. Patients with persisting LVO are at high risk for deterioration that might be decreased by NBHO.
**7.5 Exclusion Criteria, Respiratory (page 54)**		
• Acute or chronic pulmonary disease or respiratory distress that may, in the clinical judgement of the investigator, interfere with the study intervention (e.g. acute pneumonia, COPD flare-up etc.)~~Known history of chronic pulmonary disease (e.g. COPD, pulmonary fibrosis, alveolitis or pneumonitis)~~<br>• Prior to enrolment, > 2 L/min oxygen required to maintain peripheral oxygen saturation ≥ 95%<br>• ~~Acute respiratory distress that may, in the clinical judgment of the investigator, interfere with the study intervention~~<br>• ~~Acute pneumonia, alveolitis or pneumonitis of viral, bacterial, fungal or any other etiology~~	• Acute or chronic pulmonary disease or respiratory distress that may, in the clinical judgement of the investigator, interfere with the study intervention (e.g. acute pneumonia, COPD flare-up etc.)<br>• Prior to enrolment, > 2 L/min oxygen required to maintain peripheral oxygen saturation ≥ 95%	Simplified wording for easy and fast understanding
**7.6 Pre-specified Measures in Case of Slow Recruitment (page 54)**		
To ensure enrolment, in the amended protocol version 1.3, we widened the therapeutic time window for NBHO to six hours, skipped the upper age limit, and allowed more distal and also tandem arterial occlusions in case the PROOF candidate is likely to receive TBY treatment.	To ensure enrolment, in the amended protocol version 1.3, we widened the therapeutic time window for NBHO to six hours, skipped the upper age limit, and allowed more distal and also tandem arterial occlusions in case the PROOF candidate is likely to receive TBY treatment.	Adaptions according to current ESMINT/ESO and AHA/ASA guidelines [159, 160] reflect current clinical practice and will not only increase recruitment rate but also generalizability of PROOF study

Previous and new wording in track change modus	New wording	Comments/ reasons for substantial amendment
To further enhance recruitment, in the amended protocol version 1.4, we additionally allow inclusion of, first, patients with an mRS of 2, second, patients with an ASPECTS of 6 on non-contrast CT or 5 on DWI, third, patients in whom CT perfusion is not conducted, and, fourth, patients with wake-up or unknown onset stroke. First and second adaption had already been pre-specified in protocol version 1.3 ("In case of continued slow recruitment, the following inclusion criteria may also be adapted: (1) pre-stroke mRS may include 0-2, and (2) ASPECTS may be opened for e.g. 6-10 on non-contrast CT or 5-10 on DWI.") and – together with third and fourth adaption – follow the recommendations of the most recent international TBY guidelines [159, 160], and, thus, current TBY practice. Accordingly, future guideline recommendations may (as yet unknown) make further adaptions of the study protocol necessary in future in order to continuously allow best possible recruitment and generalizability of study results by enrolling a patient population that is most representative for current clinical/TBY practice.	To further enhance recruitment, in the amended protocol version 1.4, we additionally allow inclusion of, first, patients with an mRS of 2, second, patients with an ASPECTS of 6 on non-contrast CT or 5 on DWI, third, patients in whom CT perfusion is not conducted, and, fourth, patients with wake-up or unknown onset stroke. First and second adaption had already been pre-specified in protocol version 1.3 ("In case of continued slow recruitment, the following inclusion criteria may also be adapted: (1) pre-stroke mRS may include 0-2, and (2) ASPECTS may be opened for e.g. 6-10 on non-contrast CT or 5-10 on DWI.") and – together with third and fourth adaption – follow the recommendations of the most recent international TBY guidelines [159, 160], and, thus, current TBY practice. Accordingly, future guideline recommendations may (as yet unknown) make further adaptions of the study protocol necessary in future in order to continuously allow best possible recruitment and generalizability of study results by enrolling a patient population that is most representative for current clinical/TBY practice.	results. See above for detailed explanation of each individual adaption.
**7.7.1 Withdrawal of Patients from the Trial (page 55)**		
If a patient does not attend a visit, the reason should be clarified. If the patient wants to withdraw, the reason should be documented in the patients file and in the CRF, as long as the patient is willing to state it. ~~If the patient withdraws, the reason should be asked for in detail and documented in detail.~~	If a patient does not attend a visit, the reason should be clarified. If the patient wants to withdraw, the reason should be documented in the patients file and in the CRF, as long as the patient is willing to state it.	Removal of a duplicated sentence and clarification that a patient should not be forced to state a reason for the withdrawal.
**Table 12: Definition of screening-failures, drop-outs, protocol deviations (page 56)**		
		Update according to revised protocol.
**7.7.3 Premature Closure of the Clinical Trial or a Site (pages 56/57)**		
The DSMB can recommend interruption or termination of the study based on, first, the results of a tight case by case follow-up evaluation of patients'	The DSMB can recommend interruption or termination of the study based on, first, the results of a tight case by case follow-up evaluation of patients'	insertion was requested by the French CA. Slightly revised to reflect amended protocol version 1.4.

Previous and new wording in track change modus	New wording	Comments/ reasons for substantial amendment
mortality, intracranial bleedings, and early neurological effects (see Section 4.3.1 on page 40 second last and last paragraph), second, the results of the intermittent SAE evaluation or, third, of accumulating information on the above-mentioned reasons.	mortality, intracranial bleedings, and early neurological effects (see Section 4.3.1 on page 40 second last and last paragraph), second, the results of the intermittent SAE evaluation or, third, of accumulating information on the above-mentioned reasons.	
**8.4.1 Assignment of Identification Codes (page 58)**		
When the patient is included in the study (all inclusion criteria fit and none of the exclusion criteria), he/she will be given a ~~consecutive patient number~~patient ID consisting of a three-digit code of the study site followed by a consecutive randomization number. In case of a reassignment of an ID due to misspecification, the randomization service may be used again for the same patient (with the outcome of randomization pre-specified). Patients withdrawn from the study retain their number. New patients must always be allocated a new screening/randomization number.	When the patient is included in the study (all inclusion criteria fit and none of the exclusion criteria), he/she will be given a patient ID consisting of a three-digit code of the study site followed by a consecutive randomization number. In case of a reassignment of an ID due to misspecification, the randomization service may be used again for the same patient (with the outcome of randomization pre-specified). Patients withdrawn from the study retain their number. New patients must always be allocated a new screening/randomization number.	Clarification of the procedure
**8.4.2 Dosage Schedule (page 58)**		
**Intervention arm:** NBHO (i.e. inhalation of 100% oxygen at high flow (≥ 40 L/min) via a sealed non-rebreather face-mask with reservoir, or in case of intubation/ventilation for (study-independent) TBY, ventilation with an inspiratory oxygen fraction (FiO_2) of 1.0) started within 6 hours after certain stroke symptom onset (witnessed~~ or last seen well~~) or after symptom recognition (in case of wake-up or unknown onset stroke), and within 30 minutes after end of baseline brain imaging and applied until the end of TBY procedure (defined by removal of guide catheter from sheath) or, in case TBY is not attempted (defined as 'TBY was not attempted or intervention was stopped prior to any penetration or aspiration of	**Intervention arm:** NBHO (i.e. inhalation of 100% oxygen at high flow (≥ 40 L/min) via a sealed non-rebreather face-mask with reservoir, or in case of intubation/ventilation for (study-independent) TBY, ventilation with an inspiratory oxygen fraction (FiO_2) of 1.0) started within 6 hours after certain stroke symptom onset (witnessed) or after symptom recognition (in case of wake-up or unknown onset stroke), and within 30 minutes after end of baseline brain imaging and applied until the end of TBY procedure (defined by removal of guide catheter from sheath) or, in case TBY is not attempted (defined as 'TBY was not attempted or intervention was stopped prior to any penetration or aspiration of the qualifying	Adaption according to current ESMINT/ESO and AHA/ASA guidelines [159, 160] will also facilitate recruitment. In the DAWN and the DEFUSE 3 randomized controlled trials, endovascular mechanical thrombectomy was clearly beneficial in patients with wake-up stroke/unknown onset stroke due to significant volumes of salvageable ischemic brain tissue [106][107]. Consequently, beneficial effects of NBHO can equally be assumed in wake-up stroke/unknown onset stroke patients with small ischemic core at baseline as per PROOF inclusion criteria. In order to avoid any imbalance, we adapted variable used in minimization (see 8.5.1;

Previous and new wording in track change modus	New wording	Comments/ reasons for substantial amendment
the qualifying (i.e. intracranial) LVO'), 4 hours after start of study treatment.	(i.e. intracranial) LVO'), 4 hours after start of study treatment.	"Time window known < 6h vs. unknown/wake-up")
**Figure 8: Study Flow Chart (page 59)**		
		Figure was revised to reflect the amended protocol version V1.4.
**8.4.3 Compliance (pages 59/60)**		
Compliance will be recorded by the treating investigator. Oxygen administration (reason, flow-rate or FiO_2 in ventilated patients, and mask-type/method) will be continuously recorded until end of hour 24~~6~~, i.e. every change of oxygen administration must be documented. Additionally, SpO_2, and – if available – $etCO_2$ must be documented at each time point of change of oxygen administration.~~ Then, oxygen administration will be recorded hourly (±15 minutes) – together with vital signs – until end of hour 24.~~ The results will be systematically documented in the patient's medical record and in the eCRF.	Compliance will be recorded by the treating investigator. Oxygen administration (reason, flow-rate or FiO_2 in ventilated patients, and mask-type/method) will be continuously recorded until end of hour 24~~6~~, i.e. every change of oxygen administration must be documented. Additionally, SpO_2, and – if available – $etCO_2$ must be documented at each time point of change of oxygen administration. The results will be systematically documented in the patient's medical record and in the eCRF.	High-flow oxygen (i.e., NBHO) is the IMP of the PROOF study. Continuous documentation of oxygen supplementation until hour 24 will allow best possible evaluation of oxygen efficacy and safety.
Additionally, prolongation of NBHO for more than 1 hour after end of TBY procedure (defined by removal of guide catheter from sheath) or, in case TBY is not attempted (defined as 'TBY was not attempted or intervention stopped prior to any penetration or aspiration of the qualifying (i.e. intracranial) LVO'), NBHO administration time in total for more than 5 hours, will equally count as protocol violation and lead to exclusion from per-protocol analyses in the NBHO group. The coordinating investigator and the respective national coordinator ensure that local study teams are appropriately trained to stop NBHO as soon as possible after the end of TBY procedure (defined by removal of guide catheter from sheath).	Additionally, prolongation of NBHO for more than 1 hour after end of TBY procedure (defined by removal of guide catheter from sheath) or, in case TBY is not attempted (defined as 'TBY was not attempted or intervention stopped prior to any penetration or aspiration of the qualifying (i.e. intracranial) LVO'), NBHO administration time in total for more than 5 hours, will equally count as protocol violation and lead to exclusion from per-protocol analyses in the NBHO group. The coordinating investigator and the respective national coordinator ensure that local study teams are appropriately trained to stop NBHO as soon as possible after the end of TBY procedure (defined by removal of guide catheter from sheath).	insertion was requested by the French CA. Slightly revised to clarify that coordinating investigator and national coordinators should ensure appropriate training.
**8.5.1 Randomization method (page 61)**		

Previous and new wording in track change modus	New wording	Comments/ reasons for substantial amendment
Minimization [161] will be used to consider several strata when allocating treatment. In 10 per cent randomly chosen cases, the procedure will pick the treatment not assigned by the algorithm. Variables used in minimization will be:<br>• brain imaging modality at baseline (CT vs. MRI)<br>• side of large vessel occlusion (LVO) (left vs. right)<br>• intracranial LVO location (terminal internal carotid artery (ICA) with involvement of the M1-segment of the middle cerebral artery (MCA)/carotid-T vs. proximal M1-segment vs. distal M1-segment (distal of perforating branches) vs. M2/3-segment(s)<br>• NIHSS at baseline: 6-10, 11-20, 21 and more. LVO location and NIHSS will be used in conjunction for the algorithm, i.e. balance will be aimed for in every one of the nine NIHSS/LVO locations.<br>• Time window ~~0-3 hours~~ known < 6h vs. ~~> 3-6 hours~~unknown/wake-up<br>• study site	Minimization [161] will be used to consider several strata when allocating treatment. In 10 per cent randomly chosen cases, the procedure will pick the treatment not assigned by the algorithm. Variables used in minimization will be:<br>• brain imaging modality at baseline (CT vs. MRI)<br>• side of large vessel occlusion (LVO) (left vs. right)<br>• intracranial LVO location (terminal internal carotid artery (ICA) with involvement of the M1-segment of the middle cerebral artery (MCA)/carotid-T vs. proximal M1-segment vs. distal M1-segment (distal of perforating branches) vs. M2/3-segment(s)<br>• NIHSS at baseline: 6-10, 11-20, 21 and more. LVO location and NIHSS will be used in conjunction for the algorithm, i.e. balance will be aimed for in every one of the nine NIHSS/LVO locations.<br>• Time window known < 6h vs. unknown/wake-up<br>• study site	In order to avoid any imbalance between patients with known and unknown symptom onset, we adapted variable used in minimization (see 8.5.1; “Time window known < 6h vs. unknown/wake-up”)
**8.5.2 Blinding procedure (page 62)**		
Raters of ~~clinical outcome~~ the NIHSS at V5 (24 hours) and the mRS at V7 (Day 90) should be blinded to the respective treatment and chosen by the investigator in a way that no outcome-rater was involved in the emergency treatment of a respective patient. Raters must be trained and certified for NIHSS and mRS.	Raters of ~~clinical outcome~~ the NIHSS at V5 (24 hours) and the mRS at V7 (Day 90) should be blinded to the respective treatment and chosen by the investigator in a way that no outcome-rater was involved in the emergency treatment of a respective patient. Raters must be trained and certified for NIHSS and mRS.	In order to enhance feasibility of study conduction, decision was made to focus blinded secondary clinical endpoint assessment solely on the NIHSS at V5, which is the key secondary endpoint of the PROOF study, and the modified Rankin Scale score at V7, which represents the most commonly used clinical endpoint in (phase 3) stroke studies.
**9.4 V1 – Initiation of study treatment (page 64)**		

Previous and new wording in track change modus	New wording	Comments/ reasons for substantial amendment
In case the patient is randomized into the intervention arm, NBHO must be started within 6 hours after certain stroke symptom onset (witnessed~~ or last seen well~~) or after symptom recognition (in case of wake-up or unknown onset stroke), and within 30 minutes after end of baseline brain imaging; see Section 8.4.2 Dosage Schedule for details.<br>Patients that are randomized to standard treatment will receive either no O_2 supplementation (if $SpO_2 \geq 95\%$) or low-flow O_2 supplementation (if $SpO_2 \leq 94\%$) to maintain $SpO_2 \geq 95\%$ according to ESO guidelines. In case of TBY-related intubation/ventilation, the initial FiO_2 of 0.3 may be gradually increased if $SpO_2 \leq 94\%$; see Section 8.4.2 Dosage Schedule for details.<br>A 12-lead electrocardiogram is performed either prior to initiation of study treatment or within six ~~one~~ hours after start of NBHO.<br>Vital signs (incl. systolic and diastolic blood pressure, heart rate and respiratory rate, SpO_2, and – if available – $etCO_2$) are recorded ~~over the following 24 hours in this manner:~~ every 30 ±10 minutes for the first six hours after start of NBHO (or randomization in the control arm)~~, then hourly ±15 minutes until 24 hours after start of NBHO (or randomization in the control arm)~~.<br>Oxygen administration (reason, flow-rate or FiO_2 in ventilated patients, and mask-type/method) will be continuously recorded until end of hour 24~~6~~, i.e. every change of oxygen administration must be documented. Additionally, SpO_2, and – if available – $etCO_2$ must be documented at each time point of change of oxygen administration.~~ Then, oxygen administration will be recorded hourly (±15 minutes) – together with vital signs – until end of hour 24.~~	In case the patient is randomized into the intervention arm, NBHO must be started within 6 hours after certain stroke symptom onset (witnessed) or after symptom recognition (in case of wake-up or unknown onset stroke), and within 30 minutes after end of baseline brain imaging; see Section 8.4.2 Dosage Schedule for details.<br>Patients that are randomized to standard treatment will receive either no O_2 supplementation (if $SpO_2 \geq 95\%$) or low-flow O_2 supplementation (if $SpO_2 \leq 94\%$) to maintain $SpO_2 \geq 95\%$ according to ESO guidelines. In case of TBY-related intubation/ventilation, the initial FiO_2 of 0.3 may be gradually increased if $SpO_2 \leq 94\%$; see Section 8.4.2 Dosage Schedule for details.<br>A 12-lead electrocardiogram is performed either prior to initiation of study treatment or within six hours after start of NBHO.<br>Vital signs (incl. systolic and diastolic blood pressure, heart rate and respiratory rate, SpO_2, and – if available – $etCO_2$) are recorded every 30 ±10 minutes for the first six hours after start of NBHO (or randomization in the control arm).<br>Oxygen administration (reason, flow-rate or FiO_2 in ventilated patients, and mask-type/method) will be continuously recorded until end of hour 24, i.e. every change of oxygen administration must be documented. Additionally, SpO_2, and – if available – $etCO_2$ must be documented at each time point of change of oxygen administration.<br>Concomitant medication, invasive procedures and AE/SAE will be reported continuously.	Adaption according to current ESMINT/ESO and AHA/ASA guidelines [159, 160] will also facilitate recruitment. In the DAWN and the DEFUSE 3 randomized controlled trials, endovascular mechanical thrombectomy was clearly beneficial in patients with wake-up stroke/unknown onset stroke due to significant volumes of salvageable ischemic brain tissue [106][107]. Consequently, beneficial effects of NBHO can equally be assumed in wake-up stroke/unknown onset stroke patients with small ischemic core at baseline as per PROOF inclusion criteria. In order to avoid any imbalance, we adapted variable used in minimization (see 8.5.1; "Time window known < 6h vs. unknown/wake-up")<br><br>In order to avoid any delay of reperfusion therapy, ECG is routinely conducted after endovascular thrombectomy in many cases. According to this clinical practice, we widened the time window for ECG conduction in PROOF.<br><br>High-flow oxygen (i.e., NBHO) is the IMP of the PROOF study. Continuous documentation of oxygen supplementation until hour 24 will allow best possible evaluation of oxygen efficacy and safety.

Previous and new wording in track change modus	New wording	Comments/ reasons for substantial amendment
Concomitant medication, invasive procedures and AE/SAE will be reported continuously.		
**9.5 V2 – ≥ 5 minutes after start of NBHO (or randomization in the control arm) and before start of endovascular intervention (page 64)**		
~~10 to 30~~≥ 5 minutes after initiation of NBHO (or randomization in the control arm) and before start of endovascular intervention including sedation and/or endotracheal intubation/mechanical ventilation, ~~physical and neurological examination (incl.~~ NIHSS assessment is~~) are~~ repeated in order to detect early improvement (or deterioration) likely associated with study treatment. The shortened NIHSS for emergency medical services (sNIHSS-EMS [1]) may be chosen over full NIHSS assessment to speed up V2 assessment. V2 assessment should not be conducted if it interferes with routine stroke management.<br>If the patient is already sedated or under general anesthesia for the thrombectomy procedure, this must be recorded in the eCRF.<br>Vital signs as well as O_2 supplementation are recorded as indicated in Section 10.2 Vital signs and Section 10.15 Oxygen administration.<br>Concomitant medication, invasive procedures and AE/SAE will be reported continuously.	≥ 5 minutes after initiation of NBHO (or randomization in the control arm) and before start of endovascular intervention including sedation and/or endotracheal intubation/mechanical ventilation, NIHSS assessment is repeated in order to detect early improvement (or deterioration) likely associated with study treatment. The shortened NIHSS for emergency medical services (sNIHSS-EMS [1]) may be chosen over full NIHSS assessment to speed up V2 assessment. V2 assessment should not be conducted if it interferes with routine stroke management.<br>If the patient is already sedated or under general anesthesia for the thrombectomy procedure, this must be recorded in the eCRF.<br>Vital signs as well as O_2 supplementation are recorded as indicated in Section 10.2 Vital signs and Section 10.15 Oxygen administration.<br>Concomitant medication, invasive procedures and AE/SAE will be reported continuously.	Time window of V2 has been slightly widened (from 10 min to 5 min after NBHO/randomization until start of endovascular intervention) for enhanced flexibility in order to avoid interference with hyperacute stroke work-up and facilitate conduction of V2.<br><br>For the same reason, shortened NIHSS assessment may substitute for full NIHSS assessment.
**9.6 V3 – During TBY (page 64)**		
V3 is to be performed during NBHO or control treatment, respectively, either during TBY procedure (i.e. time from groin puncture to removal of guide catheter from sheath) ~~30 ±15 minutes after groin puncture~~ or, in case TBY is not attempted, 90 ±30 minutes after ~~start of study treatment (i.e. start of NBHO or time of~~ randomization~~ for the control group)~~.	V3 is to be performed during NBHO or control treatment, respectively, either during TBY procedure (i.e. time from groin puncture to removal of guide catheter from sheath) or, in case TBY is not attempted, 90 ±30 minutes after randomization.<br>Arterial blood gas analysis (incl. pH, PaO_2, SaO_2, $PaCO_2$, actual HCO_3^-, and actual base excess) is	Widened time window of V3 for enhanced flexibility in order to avoid interference with endovascular thrombectomy and facilitate conduction of V2.<br><br>Biomarker substudy has been simplified in order to ensure its conduction. Biomarker blood sampling will on be conducted at

Previous and new wording in track change modus	New wording	Comments/ reasons for substantial amendment
Arterial blood gas analysis (incl. pH, PaO_2, SaO_2, $PaCO_2$, actual HCO_3^-, and actual base excess) is performed with blood drawn from patients with study-independent arterial access (e.g. for TBY procedure). ~~A second batch of blood samples for biomarker determination (i.e. two gel serum separation Vacutest® tubes of 5 mL (Yellow cap) and two plasma EDTA K2 Vacutest® tubes of 4 mL (Lavender caps)) is taken (as part of the PROOF biomarker sub-study; see Section 10.19; total blood amount: 18 mL). Blood for biomarkers should be drawn only from patients with study-independent venous or arterial access.~~ Vital signs as well as O_2 supplementation are recorded as indicated in Section 10.2 Vital signs and Section 10.15 Oxygen administration. Concomitant medication, invasive procedures and AE/SAE will be reported continuously.	performed with blood drawn from patients with study-independent arterial access (e.g. for TBY procedure). Vital signs as well as O_2 supplementation are recorded as indicated in Section 10.2 Vital signs and Section 10.15 Oxygen administration. Concomitant medication, invasive procedures and AE/SAE will be reported continuously.	screening and V5 which directly effects required blood volume (see also Table 14 of the study protocol).
**9.7 V4 – End of study treatment (page 64)**		
V4 is to be performed within 60 min ~~at end of study treatment, i.e.~~ after end of TBY procedure (defined by removal of guide catheter from sheath) or, in case TBY is not attempted (defined as 'TBY was not attempted or intervention was stopped prior to any penetration or aspiration of the qualifying (i.e. intracranial) LVO'), 4 hours ±15 minutes after ~~start of study treatment (i.e.~~ start of NBHO (or time of randomization for the control group), or earlier, in case study treatment is prematurely terminated. Physical and neurological examination (incl. NIHSS) is repeated. Vital signs as well as O_2 supplementation are recorded as indicated in Section 10.2 Vital signs and Section 10.15 Oxygen administration.	V4 is to be performed within 60 min after end of TBY procedure (defined by removal of guide catheter from sheath) or, in case TBY is not attempted (defined as 'TBY was not attempted or intervention was stopped prior to any penetration or aspiration of the qualifying (i.e. intracranial) LVO'), 4 hours ±15 minutes after start of NBHO (or time of randomization for the control group), or earlier, in case study treatment is prematurely terminated. Physical and neurological examination (incl. NIHSS) is repeated. Vital signs as well as O_2 supplementation are recorded as indicated in Section 10.2 Vital signs and Section 10.15 Oxygen administration.	Widened time window of V4 for enhanced flexibility in order to avoid interference with endovascular thrombectomy and facilitate conduction of V4.<br><br>Addition of 'earlier' clarifies correct timing of V4 in case of premature termination of study treatment.

Previous and new wording in track change modus	New wording	Comments/ reasons for substantial amendment
Concomitant medication, invasive procedures and AE/SAE will be reported continuously.	Concomitant medication, invasive procedures and AE/SAE will be reported continuously.	
**9.8 V5 – 24 ±6 hours (Day 1) after start of NBHO (or randomization in the control arm) (page 65)**		
~~Clinical~~ NIHSS assessments at V5 should be performed by an investigator blinded to study treatment.<br>At day one after start of NBHO (or randomization in the control arm), the patient receives a first follow-up visit. For determination of the primary outcome parameter (infarct core volume assessment in between 18 and 72~~at 24 hours (22 to 36~~ hours after baseline imaging~~)~~), MRI follow-up brain imaging must be performed including DWI, ADC, FLAIR, T2*, TOF angiography as minimal standard. ~~Only in case MRI is contraindicated (e.g. in patients with pacemaker), non-contrast CT may be performed alternatively.~~If ~~brain imaging~~MRI is not feasible in between 18 and 72 ~~at 24 hours (22 to 36~~ hours~~)~~, which is defined as minor protocol deviation, MRI ~~(or CT)~~ is to be performed the soonest possible until ~~V6~~discharge.	NIHSS assessments at V5 should be performed by an investigator blinded to study treatment.<br>At day one after start of NBHO (or randomization in the control arm), the patient receives a first follow-up visit. For determination of the primary outcome parameter (infarct core volume assessment in between 18 and 72 hours after baseline imaging), MRI follow-up brain imaging must be performed including DWI, ADC, FLAIR, T2*, TOF angiography as minimal standard. If MRI is not feasible in between 18 and 72 hours, which is defined as minor protocol deviation, MRI is to be performed the soonest possible until discharge.	In order to enhance feasibility of study conduction, decision was made to focus blinded secondary clinical endpoint assessment solely on the NIHSS at V5, which is the key secondary endpoint of the PROOF study, and the modified Rankin Scale score at V7, which represents the most commonly used clinical endpoint in (phase 3) stroke studies.<br><br>Based on our experiences during PROOF conduction and feedback of all participating centers, we widened the time window for conduction of the follow-up MRI (V5), which is indispensable for optimal primary endpoint assessment in PROOF. Limited MRI capacity especially on weekends prohibited its timely conduction. Infarct volume assessment at V5 based on (study independent) CT should be avoided due to its much lower resolution, and only substitute for MRI in case of contraindications (e.g., cardiac pacemakers).
Concomitant medication, invasive procedures and AE/SAE will be reported continuously.<br>Arterial blood gas analysis (incl. pH, PaO_2, SaO_2, $PaCO_2$, actual HCO_3^-, and actual base excess) is performed with blood drawn from patients with study-independent arterial access (e.g. for TBY procedure) (as part of the PROOF trial; total blood amount: 2 mL).	Concomitant medication, invasive procedures and AE/SAE will be reported continuously.<br>Arterial blood gas analysis (incl. pH, PaO_2, SaO_2, $PaCO_2$, actual HCO_3^-, and actual base excess) is performed with blood drawn from patients with study-independent arterial access (e.g. for TBY procedure) (as part of the PROOF trial; total blood amount: 2 mL).	Correction due to simplified biomarker substudy (see also Table 14 of the study protocol).<br><br>IQCODE may easily be conducted by phone interview. Clarification facilitates IQCODE assessment especially in times of Covid-19 visiting restrictions.

Previous and new wording in track change modus	New wording	Comments/ reasons for substantial amendment
A ~~third~~ second batch of blood samples for biomarker determination (i.e. two gel serum separation Vacutest® tubes of 5 mL (Yellow cap) and two plasma EDTA K2 Vacutest® tubes of 4 mL (Lavender caps)) is taken (as part of the PROOF biomarker sub-study; see Section 10.19; total blood amount: 18 mL). Blood for biomarkers should be drawn only from patients with study-independent venous or arterial access.<br>A 12-lead-ECG is repeated.<br>For assessment of pre-existing dementia, the Informant Questionnaire on Cognitive Decline in the Elderly (IQCODE) will be performed; closest relatives should be interviewed (face-to-face or by phone) within ~~24~~ 48 hours ~~(±24)~~ after stroke onset.	A second batch of blood samples for biomarker determination (i.e. two gel serum separation Vacutest® tubes of 5 mL (Yellow cap) and two plasma EDTA K2 Vacutest® tubes of 4 mL (Lavender caps)) is taken (as part of the PROOF biomarker sub-study; see Section 10.19; total blood amount: 18 mL). Blood for biomarkers should be drawn only from patients with study-independent venous or arterial access.<br>A 12-lead-ECG is repeated.<br>For assessment of pre-existing dementia, the Informant Questionnaire on Cognitive Decline in the Elderly (IQCODE) will be performed; closest relatives should be interviewed (face-to-face or by phone) within 48 hours after stroke onset.	Simplified wording, i.e., 48 hours instead of 24 hours (±24)
**9.9 V6 – Day 5 ±2 after start of NBHO (or randomization in the control arm) or at discharge (whichever occurs first) (page 66)**		
~~A fourth batch of blood samples for biomarker determination (i.e. two gel serum separation Vacutest® tubes of 5 mL (Yellow cap) and two plasma EDTA K2 Vacutest® tubes of 4 mL (Lavender caps)) is taken (as part of the PROOF biomarker sub-study, see Section 10.19; total blood amount: 18 mL). Blood sampling for biomarkers at V6 is done either via a routinely inserted venous access or – combined with the abovementioned safety laboratory assessment – through venipuncture. However, biomarker samples at V6 are only drawn if the patient or the respective LAR consents to participation in the biomarker sub-study.~~		Biomarker substudy has been simplified in order to ensure its conduction. Biomarker blood sampling will on be conducted at screening and V5 which directly effects required blood volume (see also Table 14 of the study protocol).
**9.10 V7 – Day 90 ±10 after start of NBHO (or randomization in the control arm) (page 66)**		

Previous and new wording in track change modus	New wording	Comments/ reasons for substantial amendment
~~Clinical assessments at V7 follow-up should be performed by an investigator blinded to study treatment.~~ All study patients will receive clinical follow-up at day 90 ±10 after start of NBHO (or randomization in the control arm). Physical and neurological examination (incl. NIHSS) are repeated. Additionally, mRS, Barthel Index, MoCA, SIS-16, EQ-5D-5L and MADRS are assessed. The mRS should be performed by an investigator blinded to study treatment.	All study patients will receive clinical follow-up at day 90 ±10 after start of NBHO (or randomization in the control arm). Physical and neurological examination (incl. NIHSS) are repeated. Additionally, mRS, Barthel Index, MoCA, SIS-16, EQ-5D-5L and MADRS are assessed. The mRS should be performed by an investigator blinded to study treatment.	In order to enhance feasibility of study conduction, decision was made to focus blinded secondary clinical endpoint assessment solely on the NIHSS at V5, which is the key secondary endpoint of the PROOF study, and the modified Rankin Scale score at V7, which represents the most commonly used clinical endpoint in (phase 3) stroke studies.
		Accordingly, Table 13 was updated and the foot notes were revised.
**10.4 Brain Imaging acquisition and assessment (pages 67/68)**		
Acquisition: Brain imaging modality at screening as per standard care, i.e. CT- or MRI-based. Minimal standard care includes non-contrast CT, CT angiography and, if recommended by the attending physician, CT perfusion~~, (ideally whole-brain, minimum coverage ≥ 75 mm)~~ or DWI, apparent diffusion coefficient (ADC), fluid-attenuated inversion recovery (FLAIR), T2*, time-of-flight (TOF) angiography and, if recommended by the attending physician, gadolinium-enhanced MR perfusion, respectively. eASPECTS software (Brainomix, Oxford, UK), or a comparable application, is recommended for automated and fast determination of ASPECTS (see inclusion criteria). Extracranial CT- or MRI-based vessel imaging at baseline (not required for study participation, see inclusion criteria), digital subtraction angiography during TBY procedure as well as any other additional (study-independent) brain or vessel imaging	Acquisition: Brain imaging modality at screening as per standard care, i.e. CT- or MRI-based. Minimal standard care includes non-contrast CT, CT angiography and, if recommended by the attending physician, CT perfusion, or DWI, apparent diffusion coefficient (ADC), fluid-attenuated inversion recovery (FLAIR), T2*, time-of-flight (TOF) angiography and, if recommended by the attending physician, gadolinium-enhanced MR perfusion, respectively. eASPECTS software (Brainomix, Oxford, UK), or a comparable application, is recommended for automated and fast determination of ASPECTS (see inclusion criteria). Extracranial CT- or MRI-based vessel imaging at baseline (not required for study participation, see inclusion criteria), digital subtraction angiography during TBY procedure as well as any other additional (study-independent) brain or vessel imaging	According to current guidelines perfusion imaging is only obligatory for specific subgroups such as patients with wake-up or unknown onset strokes, but not in patients with known stroke onset <6h. In cases no perfusion imaging will be available, CT angiography source images will be used for ischemic core estimation at baseline.<br><br>Clarification that automated ASPECTS assessment at study sites can be done with any application of any company.<br><br>Based on our experiences during PROOF conduction and feedback of all participating centers, we widened the time window for conduction of the follow-up MRI (V5), which is indispensable for optimal primary endpoint assessment in PROOF. Limited MRI capacity especially on weekends prohibited

Previous and new wording in track change modus	New wording	Comments/ reasons for substantial amendment
performed during the course of the study (until day 30) must be provided to the imaging core lab.<br>MRI follow-up brain imaging (infarct volume assessment at V524 hours (22 to 36 hours) after baseline brain imaging must be performed in between 18 and 72 hours after baseline brain imaging including DWI, ADC, FLAIR, T2*, TOF angiography as minimal standard (only in case MRI is contraindicated (e.g. in patients with pacemaker), non-contrast CT may be performed alternatively. If brain imaging is not feasible in between at 24 hours (22 to 3618 and 72 hours), which is defined as minor protocol deviation, MRI (or CT) is to be performed the soonest possible until day 5 or discharge.	performed during the course of the study (until day 30) must be provided to the imaging core lab.<br>MRI follow-up brain imaging (infarct volume assessment at V5) must be performed in between 18 and 72 hours after baseline brain imaging including DWI, ADC, FLAIR, T2*, TOF angiography as minimal standard. If brain imaging is not feasible in between 18 and 72 hours), which is defined as minor protocol deviation, MRI is to be performed the soonest possible until discharge.	its timely conduction. Infarct volume assessment at V5 based on (study independent) CT should be avoided due to its much lower resolution, and only substitute for MRI in case of contraindications (e.g., cardiac pacemakers).
Assessment:<br>All brain imaging assessments are performed by two independent neuroradiologists blinded to treatment allocation. A detailed description can be found in the Image Interpretation Guidelines.<br>For the primary endpoint analysis, infarct core volume at baseline will be assessed on either CBF maps using the < 30% threshold (or on CT angiography source images* in case CT perfusion scans are of insufficient quality or in patients in whom no CT perfusion had been conducted) or on DWI, depending on the respective baseline brain imaging modality, i.e. CT or MRI. Infarct volume at follow-up will be primarily determined on MRI (FLAIR in conjunction with DWI). Only if no follow-up MRI is available, infarct volume will be assessed on the follow-up non-contrast CT that is closest to the 24-hour time point.<br>* The ability of CT angiography to show intracranial arterial occlusion and collateral blood flow in patients with acute stroke has been established more than 20 years ago [165]. Only recently, multiphase CT	Assessment:<br>All brain imaging assessments are performed by two independent neuroradiologists blinded to treatment allocation. A detailed description can be found in the Image Interpretation Guidelines.<br>For the primary endpoint analysis, infarct core volume at baseline will be assessed on either CBF maps using the < 30% threshold (or on CT angiography source images* in case CT perfusion scans are of insufficient quality or in patients in whom no CT perfusion had been conducted) or on DWI, depending on the respective baseline brain imaging modality, i.e. CT or MRI. Infarct volume at follow-up will be primarily determined on MRI (FLAIR in conjunction with DWI). Only if no follow-up MRI is available, infarct volume will be assessed on the follow-up non-contrast CT that is closest to the 24-hour time point.<br>* The ability of CT angiography to show intracranial arterial occlusion and collateral blood flow in patients with acute stroke has been established more than 20 years ago [165]. Only recently, multiphase CT	Core Imaging Laboratory approach for ischemic core estimation at baseline depending on imaging modality including CT angiography source imaging that became necessary due to adaptions according to current ESMINT/ESO and AHA/ASA guidelines [159, 160] in which perfusion imaging is only obligatory for specific subgroups such as patients with wake-up or unknown onset strokes, but not in patients with known stroke onset <6h. In cases no perfusion imaging will be available, CT angiography source images will be used for ischemic core estimation at baseline. This adaption will not only facilitate enrollment but also compensate for drop-outs due to insufficient quality of perfusion imaging, which has been observed in ~10% of enrolled cases.

Previous and new wording in track change modus	New wording	Comments/ reasons for substantial amendment
angiography has been observed to predict tissue fate regionally in acute ischemic stroke patients similar to CT perfusion [166, 167]. Others recently observed that also single-phase CT angiography is comparable to multiphase CT angiography for the selection of patients with ischemic cores of < 31 mL and < 70 mL, and ≥ 100 mL [168]. The authors conclude that this technique allows the selection of patients for endovascular therapy by accurately predicting lower infarct core volume cutoffs. Although the scientific basis is still limited, CT angiography source images are a valuable diagnostic alternative in patients for whom CT perfusion cannot be obtained. It will be used as an alternative method for core volume identification in patients without CT perfusion within the PROOF study.	angiography has been observed to predict tissue fate regionally in acute ischemic stroke patients similar to CT perfusion [166, 167]. Others recently observed that also single-phase CT angiography is comparable to multiphase CT angiography for the selection of patients with ischemic cores of < 31 mL and < 70 mL, and ≥ 100 mL [168]. The authors conclude that this technique allows the selection of patients for endovascular therapy by accurately predicting lower infarct core volume cutoffs. Although the scientific basis is still limited, CT angiography source images are a valuable diagnostic alternative in patients for whom CT perfusion cannot be obtained. It will be used as an alternative method for core volume identification in patients without CT perfusion within the PROOF study.	
**10.9 IQCODE (Informant Questionnaire on Cognitive Decline in the Elderly) (page 68)**		
The 16-item IQCODE (http://rsph.anu.edu.au/research/tools-resources/informant-questionnaire-cognitive-decline-elderly) is an informant questionnaire that seeks to retrospectively ascertain change in cognitive and functional performance over a 10-year time period.[176] 16-item IQCODE is designed as a brief screen for potential dementia, usually administered as a questionnaire given to the relevant proxy. For each item the chosen proxy scores change on a five-point ordinal hierarchical scale, with responses ranging from 1: 'has become much better' to 5: 'has become much worse'. This gives a sum-score of 16 to 80 that can be averaged by the total number of completed items to give a final score of 1.0 to 5.0, where higher scores indicate greater decline. The closest available proxy should be interviewed (face-	The 16-item IQCODE (http://rsph.anu.edu.au/research/tools-resources/informant-questionnaire-cognitive-decline-elderly) is an informant questionnaire that seeks to retrospectively ascertain change in cognitive and functional performance over a 10-year time period.[176] 16-item IQCODE is designed as a brief screen for potential dementia, usually administered as a questionnaire given to the relevant proxy. For each item the chosen proxy scores change on a five-point ordinal hierarchical scale, with responses ranging from 1: 'has become much better' to 5: 'has become much worse'. This gives a sum-score of 16 to 80 that can be averaged by the total number of completed items to give a final score of 1.0 to 5.0, where higher scores indicate greater decline. The closest available proxy should be interviewed (face-	IQCODE may easily be conducted by phone interview. Clarification facilitates IQCODE assessment especially in times of Covid-19 visiting restrictions.<br><br>Simplified wording, i.e., 48 hours instead of 24 hours (±24)

Previous and new wording in track change modus	New wording	Comments/ reasons for substantial amendment
to-face or by phone) within ~~24~~ 48 hours ~~(± 24 hours)~~ after stroke onset (see Appendix ~~4~~7).	to-face or by phone) within 48 hours after stroke onset (see 7).	
**10.19 Biomarkers (Pharmacodynamics) Substudy (page 71)**		
Serum and plasma blood samples will be collected from each Participant at the following visits:<br>• Screening visit: 18mL<br>• ~~Visit 3 (during study treatment): 18mL~~<br>• Visit 5 (24 ±6 hours): 18mL<br>• ~~Visit 6 (Day 5 ±2): 18ml~~<br><br>Total study-dependent blood volume for biomarker analysis: ~~72~~ 36 mL; biomarker blood is only drawn using a pre-existing vascular access; no venipuncture is required.	Serum and plasma blood samples will be collected from each Participant at the following visits:<br>• Screening visit: 18mL<br>• Visit 5 (24 ±6 hours): 18mL<br><br>Total study-dependent blood volume for biomarker analysis: 36 mL; biomarker blood is only drawn using a pre-existing vascular access; no venipuncture is required.	Biomarker substudy has been simplified in order to ensure its conduction. Consequently, blood volume for biomarkers has been reduced from 72 to 36 mL and max. total blood volume has been reduced accordingly from 90.5 mL to now 54.5 mL (see also Table 14 of the study protocol).
**10.20 Arterial blood gases (Pharmacokinetics) (page 72)**		
		Table 14 was revised and the blood volumes updated according to the simplified biomarker study.
**11.1.2 Serious Adverse Events and Adverse Events of Special Interest (page 74)**		
Following events are defined as **adverse events of special interest (AESI)** in the PROOF trial and have to be reported by investigator in accordance with the requirements for the reporting of SAE:<br>- parenchymal hematoma 1 (class 1c, Heidelberg bleeding classification [101])<br>- parenchymal hematoma 2 (class 2, Heidelberg bleeding classification [101])<br>- parenchymal hematoma remote from infarcted brain tissue (class 3, Heidelberg bleeding classification [101])<br>- intraventricular hemorrhage (class 3b, Heidelberg bleeding classification [101])<br>- subarachnoid hemorrhage (class 3c, Heidelberg bleeding classification [101])	Following events are defined as **adverse events of special interest (AESI)** in the PROOF trial and have to be reported by investigator in accordance with the requirements for the reporting of SAE:<br>- parenchymal hematoma 1 (class 1c, Heidelberg bleeding classification [101])<br>- parenchymal hematoma 2 (class 2, Heidelberg bleeding classification [101])<br>- parenchymal hematoma remote from infarcted brain tissue (class 3, Heidelberg bleeding classification [101])<br>- intraventricular hemorrhage (class 3b, Heidelberg bleeding classification [101])<br>- subarachnoid hemorrhage (class 3c, Heidelberg bleeding classification [101])	Adaption of AESI according to DSMB's requirements in order to capture all parenchymal hematomas and all remote intracranial bleedings even if asymptomatic.

Previous and new wording in track change modus	New wording	Comments/ reasons for substantial amendment
- subdural hematoma (class 3c, Heidelberg bleeding classification [101])<br>- intracranial hemorrhages (ICH) with neurological deterioration if the ICH is the predominant cause of the deterioration. For this,<br>- ~~In addition to all characteristics that have to be documented for each SAE,~~ a classification ~~of AESI (ICH)~~ according to the **ECASS III** and **Heidelberg bleeding classification** will be provided on the SAE-form.	- subdural hematoma (class 3c, Heidelberg bleeding classification [101])<br>intracranial hemorrhages (ICH) with neurological deterioration if the ICH is the predominant cause of the deterioration. For this, a classification according to the **ECASS III** and **Heidelberg bleeding classification** will be provided on the SAE-form.	
**11.3 Reporting of Serious Adverse Events by Investigator (page 76)**		
All SAE must be reported by the investigator to the responsible Safety Officer at the KKS Heidelberg within 24 hours after the SAE becomes known using the "Serious Adverse Event" form. The initial report must be as complete as possible including details of the current illness and (serious) adverse event and an assessment of the causal relationship between the event and the trial medication.<br><br>The reporting will be performed by faxing a completed 'SAE Form' to the KKS Heidelberg. Fax number:<br>**+49 (0) 6221 – 56 – 33725**<br>Only in case of technical faults in fax transmission, the SAE Form can be also submitted by e-mail:<br>**pharmakovigilanz.KKS@med.uni-heidelberg.de**	All SAE must be reported by the investigator to the responsible Safety Officer at the KKS Heidelberg within 24 hours after the SAE becomes known using the "Serious Adverse Event" form. The initial report must be as complete as possible including details of the current illness and (serious) adverse event and an assessment of the causal relationship between the event and the trial medication.<br><br>The reporting will be performed by faxing a completed 'SAE Form' to the KKS Heidelberg. Fax number:<br>**+49 (0) 6221 – 56 – 33725**<br>Only in case of technical faults in fax transmission, the SAE Form can be also submitted by e-mail:<br>**pharmakovigilanz.KKS@med.uni-heidelberg.de**	Addition that SAE forms can also be submitted by e-mail in case of technical faults in fax transmission.
**11.4 Expedited Reporting (page 76)**		

Previous and new wording in track change modus	New wording	Comments/ reasons for substantial amendment
All SAEs will be subject to a second assessment by a designated person, who will be independent from the reporting investigator. The designated person for the present trial, referred to as the second assessor is: Prof. Dr. med. ~~Dr. Sven Poli~~Christine Meyer-Zürn (Dept. of Cardiology, University Hospital Basel, Switzerland).	All SAEs will be subject to a second assessment by a designated person, who will be independent from the reporting investigator. The designated person for the present trial, referred to as the second assessor is: Prof. Dr. med. Christine Meyer-Zürn (Dept. of Cardiology, University Hospital Basel, Switzerland).	Change of staff for this responsibility according to DSMB's requirement to avoid unblinding of the coordinating investigator.
**12.2.1 Primary analysis variable (page 78)**		
Efficacy of NBHO treatment will be determined by an ITT analysis of difference of ischemic core growth (defined as the difference in ischemic core volume (in mL) from baseline to 24 hours) between groups; brain tissue not included in CT perfusion or MR diffusion at baseline will be excluded from lesion volume measurements; in case of premature death (i.e. death without repeat scan), patients will be treated as worst individual outcome of ischemic core growth, i.e. the growth value will be set to the difference in volume between the initial core at baseline and the initial volume at Tmax > 6 seconds or equivalent volume on CT angiography source images in case CT perfusion is of insufficient quality or was not conducted.	Efficacy of NBHO treatment will be determined by an ITT analysis of difference of ischemic core growth (defined as the difference in ischemic core volume (in mL) from baseline to 24 hours) between groups; brain tissue not included in CT perfusion or MR diffusion at baseline will be excluded from lesion volume measurements; in case of premature death (i.e. death without repeat scan), patients will be treated as worst individual outcome of ischemic core growth, i.e. the growth value will be set to the difference in volume between the initial core at baseline and the initial volume at Tmax > 6 seconds or equivalent volume on CT angiography source images in case CT perfusion is of insufficient quality or was not conducted.	Core Imaging Laboratory approach for primary endpoint analysis in case of CT angiography source imaging being used for ischemic core estimation at baseline. Adaptions became necessary due to adaptions according to current ESMINT/ESO and AHA/ASA guidelines [159, 160] in which perfusion imaging is only obligatory for specific subgroups such as patients with wake-up or unknown onset strokes, but not in patients with known stroke onset <6h. In cases no perfusion imaging will be available, CT angiography source images will be used for ischemic core estimation at baseline (see Chapter 10.4). This adaption will not only facilitate enrollment but also compensate for drop-outs due to insufficient quality of perfusion imaging, which has been observed in ~10% of enrolled cases.
**12.2.2 Secondary analysis variable (pages 79/80)**		
Clinical efficacy analyses:<br>• Survival at V6 and V7 (90 days after randomization)<br>• Utility-weighted mRS at day 5/discharge and at day 90 [189]~~(Chaisinanunkul et al. Stroke.~~	Clinical efficacy analyses:<br>• Survival at V6 and V7 (90 days after randomization)	Citation replaced by corresponding EndNote citation<br><br>Adaption according to new time windows of V2 and V4

Previous and new wording in track change modus	New wording	Comments/ reasons for substantial amendment
2015 Aug;46(8):2238-43) [time frames: day5/discharge, and day 90]<br>• categorical shift in the mRS at day 5/discharge and at day 90 [time frames: day5/discharge, and day 90]<br>• mRS at day 5/discharge and at day 90, dichotomized 0 to 1 (no significant disability) vs. 2 to 6 [time frames: day5/discharge, and day 90]<br>• mRS at day 5/discharge and at day 90, dichotomized 0 to 2 (good outcome) vs. 3 to 6 [time frames: day5/discharge, and day 90]<br>• Barthel Index (BI) at day 5/discharge* and at day 90* [time frames: day5/discharge, and day 90] **in case of premature death an BI score of 0 will be applied*<br>• NIHSS at 20 minutesV2*, at V4*, at 24 hours*, at day 5/discharge* and at day 90* [time frames: 20 5 minutes, 24 hours, day5/discharge, and day 90] *(*in case of premature death an NIHSS score of 42 will be applied)*§<br>• Difference of change in NIHSS score from baseline to V2 and from V2 to V5 between groups *(*in case of premature death an NIHSS score of 42 will be applied)*§<br>• very early neurological improvement (VENI) at 20 minutesV2 / early neurological improvement (ENI) at 24 hours / subacute neurological improvement (SNI) at day 5/discharge and late neurological improvement (LNI) at day 90; proportion of subjects with NIHSS drop of ≥ 4 and ≥ 8 from baseline or NIHSS score 0 to 3 [time frames: 20 5 minutes, 24 hours, day5/discharge, and day 90]§	• Utility-weighted mRS at day 5/discharge and at day 90 [189] [time frames: day5/discharge, and day 90]<br>• categorical shift in the mRS at day 5/discharge and at day 90 [time frames: day5/discharge, and day 90]<br>• mRS at day 5/discharge and at day 90, dichotomized 0 to 1 (no significant disability) vs. 2 to 6 [time frames: day5/discharge, and day 90]<br>• mRS at day 5/discharge and at day 90, dichotomized 0 to 2 (good outcome) vs. 3 to 6 [time frames: day5/discharge, and day 90]<br>• Barthel Index (BI) at day 5/discharge* and at day 90* [time frames: day5/discharge, and day 90] **in case of premature death an BI score of 0 will be applied*<br>• NIHSS at V2*, at V4*, at 24 hours*, at day 5/discharge* and at day 90* [time frames: 5 minutes, 24 hours, day5/discharge, and day 90] *(*in case of premature death an NIHSS score of 42 will be applied)*§<br>• Difference of change in NIHSS score from baseline to V2 and from V2 to V5 between groups *(*in case of premature death an NIHSS score of 42 will be applied)*§<br>• very early neurological improvement (VENI) at V2 / early neurological improvement (ENI) at 24 hours / subacute neurological improvement (SNI) at day 5/discharge and late neurological improvement (LNI) at day 90; proportion of subjects with NIHSS drop of ≥ 4 and ≥ 8 from baseline or NIHSS score 0 to 3 [time frames: 5 minutes, 24 hours, day5/discharge, and day 90]§	"§" In order to account for confounding effects of sedation/ anaesthesia on neurological status, especially during and shortly after endovascular thrombectomy, we will conduct NIHSS analysis with and without exclusion of sedated/anaesthetized patients

Previous and new wording in track change modus	New wording	Comments/ reasons for substantial amendment
• Complete recovery defined as a decrease in total NIHSS score to 3 or lower at 24 hours[§]<br>• Montreal Cognitive Assessment (MoCA) score corrected for pre-stroke cognitive status (IQCODE), EQ-5D-5L (EuroQol), Stroke Impact Scale (SIS)-16 and Montgomery-Åsberg Depression Rating Scale (MADRS) score at day 90 [time frame: day 90] will be analyzed using a linear regression model<br>• PaO_2 at 90 minutes and 24 hours<br><br>[§] *with and without exclusion of patients under sedation or anesthesia*	• Complete recovery defined as a decrease in total NIHSS score to 3 or lower at 24 hours[§]<br>• Montreal Cognitive Assessment (MoCA) score corrected for pre-stroke cognitive status (IQCODE), EQ-5D-5L (EuroQol), Stroke Impact Scale (SIS)-16 and Montgomery-Åsberg Depression Rating Scale (MADRS) score at day 90 [time frame: day 90] will be analyzed using a linear regression model<br>• PaO_2 at 90 minutes and 24 hours<br><br>[§] *with and without exclusion of patients under sedation or anesthesia*	
Clinical safety analyses:<br>• all-cause death at day 5/discharge and day 90<br>• stroke-related death at day 5/discharge and day 90<br>• symptomatic intracranial hemorrhage (sICH) as per ECASS III definition and per Heidelberg bleeding classification [101]~~(von Kummer et al. Stroke. 2015;46: 2981-6;~~ (see Appendix ~~11~~14)~~)~~ at 24 hours*, until day 5/discharge* will be analyzed using logistic regression models [time frames: 24 hours, day 5/discharge] **in case of missing follow-up brain imaging due to premature death, sICH will be replaced by "sICH or death without repeat scan"*<br>• mRS at day 5/discharge and at day 90, dichotomized 5 to 6 (severe disability or death) vs. 0 to 4 [time frames: day5/discharge, and day 90]	Clinical safety analyses:<br>• all-cause death at day 5/discharge and day 90<br>• stroke-related death at day 5/discharge and day 90<br>• symptomatic intracranial hemorrhage (sICH) as per ECASS III definition and per Heidelberg bleeding classification [101] (see Appendix 14) at 24 hours*, until day 5/discharge* will be analyzed using logistic regression models [time frames: 24 hours, day 5/discharge] **in case of missing follow-up brain imaging due to premature death, sICH will be replaced by "sICH or death without repeat scan"*<br>• mRS at day 5/discharge and at day 90, dichotomized 5 to 6 (severe disability or death) vs. 0 to 4 [time frames: day5/discharge, and day 90]	Citation replaced by corresponding EndNote citation<br><br>Adaption according to new time window of V2<br><br>Correction of abbreviation<br><br>In order to account for confounding effects of sedation/ anaesthesia on neurological status, especially during and shortly after endovascular thrombectomy, we will conduct NIHSS analysis with and without exclusion of sedated/anaesthetized patients

Previous and new wording in track change modus	New wording	Comments/ reasons for substantial amendment
• very early neurological deterioration (VEND) at ~~20 minutes~~V2 / early neurological deterioration (END) at 24 hours / subacute neurological deterioration (SND~~I~~) at day 5/discharge and late neurological deterioration (LND) at day 90; proportion of subjects with NIHSS increase of ≥ 4 and ≥ 8 from baseline [time frames: ~~20~~ 5 minutes, 24 hours, day5/discharge, and day 90] *(with and without exclusion of patients under sedation or anesthesia)*<br>• decompressive hemicraniectomy until day 5/discharge and day 90 [time frames: day5/discharge, and day 90day]<br>• any SAE until 24 hours, day 5/discharge and day 90 [time frames: 24 hours, day5/discharge, and day 90]<br>• respiratory SAE until 24 hours, day 5/discharge and day 90 [time frames: 24 hours, day5/discharge, and day 90]<br>• pneumonia until day 5/discharge and day 90 [time frames: day5/discharge, and day 90]<br>• respiratory failure leading to ventilation or death until 24 hours, day 5/discharge and day 90 [time frames: 24 hours, day5/discharge, and day 90]<br>• duration of ventilation (invasive only and invasive/non-invasive combined) until day 5/discharge and day 90 [time frames: day5/discharge, and day 90]<br>• length of stay in ICU until day 5/discharge and day 90 [time frames: day5/discharge, and day 90]<br>• length of stay in hospital [time frame: day 90]	• very early neurological deterioration (VEND) at V2 / early neurological deterioration (END) at 24 hours / subacute neurological deterioration (SND) at day 5/discharge and late neurological deterioration (LND) at day 90; proportion of subjects with NIHSS increase of ≥ 4 and ≥ 8 from baseline [time frames: 5 minutes, 24 hours, day5/discharge, and day 90] (*with and without exclusion of patients under sedation or anesthesia*)<br>• decompressive hemicraniectomy until day 5/discharge and day 90 [time frames: day5/discharge, and day 90day]<br>• any SAE until 24 hours, day 5/discharge and day 90 [time frames: 24 hours, day5/discharge, and day 90]<br>• respiratory SAE until 24 hours, day 5/discharge and day 90 [time frames: 24 hours, day5/discharge, and day 90]<br>• pneumonia until day 5/discharge and day 90 [time frames: day5/discharge, and day 90]<br>• respiratory failure leading to ventilation or death until 24 hours, day 5/discharge and day 90 [time frames: 24 hours, day5/discharge, and day 90]<br>• duration of ventilation (invasive only and invasive/non-invasive combined) until day 5/discharge and day 90 [time frames: day5/discharge, and day 90]<br>• length of stay in ICU until day 5/discharge and day 90 [time frames: day5/discharge, and day 90]<br>• length of stay in hospital [time frame: day 90]	

Previous and new wording in track change modus	New wording	Comments/ reasons for substantial amendment
• myocardial infarction until 24 hours, day 5/discharge and day 90 [time frames: 24 hours, day5/discharge, and day 90]<br>• major adverse cardiovascular events (MACE) including recurrent stroke, myocardial infarction and cardiovascular death until 24 hours, day 5/discharge and day 90 [time frames: 24 hours, day5/discharge, and day 90]	• myocardial infarction until 24 hours, day 5/discharge and day 90 [time frames: 24 hours, day5/discharge, and day 90]<br>• major adverse cardiovascular events (MACE) including recurrent stroke, myocardial infarction and cardiovascular death until 24 hours, day 5/discharge and day 90 [time frames: 24 hours, day5/discharge, and day 90]	
Secondary imaging efficacy analyses:<br>• PP only: absolute difference in ischemic core volume (in mL) [time frame: 24 hours]<br>• relative changes in ischemic core volume (in %) [time frame: 24 hours]<br>• absolute and relative ischemic core change using either NCCT or DWI-MRI (or CT~~/MR~~ angiography source images and DWI) for ischemic core estimation at baseline (i.e. NCCT (or CT angiography source images) will substitute for CBF < 30% in patients with CT-based imaging at baseline~~, and MR angiography may substitute for DWI-MRI~~) [time frame: 24 hours]<br>• absolute and relative ischemic core change using CBF < 30% for ischemic core estimation at baseline in all patients, independent of imaging modality (i.e. DWI will be substituted by MR perfusion CBF < 30%) [time frame: 24 hours]<br>• penumbral salvage defined as (penumbra volume at baseline – infarct core volume at 24 hours) / (penumbra volume at baseline – ischemic core volume at baseline) [time frame: 24 hours]	Secondary imaging efficacy analyses:<br>• PP only: absolute difference in ischemic core volume (in mL) [time frame: 24 hours]<br>• relative changes in ischemic core volume (in %) [time frame: 24 hours]<br>• absolute and relative ischemic core change using either NCCT or DWI-MRI (or CT angiography source images and DWI) for ischemic core estimation at baseline (i.e. NCCT (or CT angiography source images) will substitute for CBF < 30% in patients with CT-based imaging at baseline) [time frame: 24 hours]<br>• absolute and relative ischemic core change using CBF < 30% for ischemic core estimation at baseline in all patients, independent of imaging modality (i.e. DWI will be substituted by MR perfusion CBF < 30%) [time frame: 24 hours]<br>• penumbral salvage defined as (penumbra volume at baseline – infarct core volume at 24 hours) / (penumbra volume at baseline – ischemic core volume at baseline) [time frame: 24 hours]	Correction of clerical error: MR angiography was never planned for ischemic core estimation. DWI is the correct modality.<br><br>Clarification by adding “source images”: because only CT angiography source images but not reconstructions of CT angiography can be used for core estimation.

Previous and new wording in track change modus	New wording	Comments/ reasons for substantial amendment
• of patients who received TBY: proportion of TICI on DSA (final run) (as suggested in [190]) [time frame: 2 to 4 hours]<br>• revascularization rate on 24-hour follow-up MRA (or CTA if available) [time frame: 24 hours]	• of patients who received TBY: proportion of TICI on DSA (final run) (as suggested in [190]) [time frame: 2 to 4 hours]<br>• revascularization rate on 24-hour follow-up MRA (or CTA if available) [time frame: 24 hours]	
Predefined exploratory analyses:<br>• Exploratory analyses of imaging (including clot perviousness [191, 192], clot burden, and collateral status [193]) will help to understand the pathophysiology of NBHO in stroke. The precise scope of data acquisition and analysis is described in the imaging protocol and Image Interpretation Guidelines.<br>• Exploratory analyses of biochemical biomarkers will be useful to develop a blood-based test to monitor efficacy and safety of NBHO treatment. The specific objectives of this sub-study are:<br>○ To create a larger European bio resource of stroke blood samples (PROOF-Bio-Bank) that will allow to biologically demonstrate some of the expected benefits of NBHO.<br>○ To measure blood biomarkers related with the clinical and neuroimaging endpoints and to define the clinical utility of candidate biomarkers involving three main pathways (Oxidative stress, matrix metalloproteinases and inflammation).<br>To define a prototype to be used as a Point-of-Care (POC) device for blood markers to guide stroke NBHO therapy triage and management	Predefined exploratory analyses:<br>• Exploratory analyses of imaging (including clot perviousness [191, 192], clot burden, and collateral status [193]) will help to understand the pathophysiology of NBHO in stroke. The precise scope of data acquisition and analysis is described in the imaging protocol and Image Interpretation Guidelines.<br>• Exploratory analyses of biochemical biomarkers will be useful to develop a blood-based test to monitor efficacy and safety of NBHO treatment. The specific objectives of this sub-study are:<br>○ To create a larger European bio resource of stroke blood samples (PROOF-Bio-Bank) that will allow to biologically demonstrate some of the expected benefits of NBHO.<br>○ To measure blood biomarkers related with the clinical and neuroimaging endpoints and to define the clinical utility of candidate biomarkers involving three main pathways (Oxidative stress, matrix metalloproteinases and inflammation).<br>To define a prototype to be used as a Point-of-Care (POC) device for blood markers to guide stroke NBHO therapy triage and management	Specification of new exploratory analyses
**12.3 Definition of Trial Population to be analyzed (page 81/82)**		

Previous and new wording in track change modus	New wording	Comments/ reasons for substantial amendment
Other subsets of the full analysis set comprise patients with the following characteristics:<br>• TICI 2b/3 at end of TBY and target mismatch profile (i.e. baseline penumbra (T_{max} > 6 seconds or equivalent volume on CT angiography in case of missing or low quality CT perfusion): core (CBF < 30% compared to healthy tissue or equivalent volume on CT angiography in case of missing or low quality CT perfusion, or DWI, depending on respective imaging modality) ratio ≥ 1.2 and volume ≥ 15 mL; voxel-based post-hoc analyses) vs. TICI 2b/3 at end of TBY and no target mismatch profile<br>• TICI 3 at end of TBY and target mismatch profile vs. TICI 3 at end of TBY and no target mismatch profile<br>• TICI 2b/3 at end of TBY and baseline ischemic core volume ≤ 100 mL and target mismatch profile vs. TICI 2b/3 at end of TBY and large ischemic core at baseline (> 100 mL) and no target mismatch profile<br>• TICI 3 at end of TBY and baseline ischemic core volume ≤ 100 mL and target mismatch profile vs. TICI 3 at end of TBY and large ischemic core at baseline (> 100 mL) and no target mismatch profile<br>• TICI 2b/3 at end of TBY vs. TICI 0-2a at end of TBY or TBY not attempted<br>• TICI 2a-3 at end of TBY vs. TICI 0-1 at end of TBY or TBY not attempted<br>• Patients with complete reperfusion of target mismatch area (correlation of CT or MR perfusion with DSA (final run)) vs. patients with no or incomplete reperfusion of target mismatch area	Other subsets of the full analysis set comprise patients with the following characteristics:<br>• TICI 2b/3 at end of TBY and target mismatch profile (i.e. baseline penumbra (T_{max} > 6 seconds or equivalent volume on CT angiography in case of missing or low quality CT perfusion): core (CBF < 30% compared to healthy tissue or equivalent volume on CT angiography in case of missing or low quality CT perfusion, or DWI, depending on respective imaging modality) ratio ≥ 1.2 and volume ≥ 15 mL; voxel-based post-hoc analyses) vs. TICI 2b/3 at end of TBY and no target mismatch profile<br>• TICI 3 at end of TBY and target mismatch profile vs. TICI 3 at end of TBY and no target mismatch profile<br>• TICI 2b/3 at end of TBY and baseline ischemic core volume ≤ 100 mL and target mismatch profile vs. TICI 2b/3 at end of TBY and large ischemic core at baseline (> 100 mL) and no target mismatch profile<br>• TICI 3 at end of TBY and baseline ischemic core volume ≤ 100 mL and target mismatch profile vs. TICI 3 at end of TBY and large ischemic core at baseline (> 100 mL) and no target mismatch profile<br>• TICI 2b/3 at end of TBY vs. TICI 0-2a at end of TBY or TBY not attempted<br>• TICI 2a-3 at end of TBY vs. TICI 0-1 at end of TBY or TBY not attempted<br>• Patients with complete reperfusion of target mismatch area (correlation of CT or MR perfusion with DSA (final run)) vs. patients with no or incomplete reperfusion of target mismatch area	Core Imaging Laboratory approach for mismatch analysis in case of CT angiography source imaging being used for ischemic core estimation at baseline. Adaptions became necessary due to adaptions according to current ESMINT/ESO and AHA/ASA guidelines [159, 160] in which perfusion imaging is only obligatory for specific subgroups such as patients with wake-up or unknown onset strokes, but not in patients with known stroke onset <6h. In cases no perfusion imaging will be available, CT angiography source images will be used for ischemic core estimation at baseline (see Chapter 10.4). This adaption will not only facilitate enrollment but also compensate for drop-outs due to insufficient quality of perfusion imaging, which has been observed in ~10% of enrolled cases.<br><br>New pre-specified subgroup analysis of patients within the very early (0-3 hours) and early (0-6 hours) time window after symptom onset or after symptom recognition.<br><br>New pre-specified subgroup analysis of patients with or without silent ischemic lesions in order to address any no-matter-how-small impact on clinical outcomes due to silent ischemic lesions.

Previous and new wording in track change modus	New wording	Comments/ reasons for substantial amendment
• ASPECTS ≤ 8 on baseline NCCT or ≤ 7 on baseline DWI vs. ASPECTS ≥ 9 on baseline NCCT or ≥ 8 on baseline DWI<br>• intubation/ventilation vs. conscious sedation<br>• IVT vs. no-IVT<br>• age ≤ 60 vs. > 60<br>• age ≤ 70 vs. > 70<br>• age ≤ 80 vs. > 80<br>• NIHSS at baseline < 10 vs. 10-20 vs. > 20, +/- cross-classification with LVO-location<br>• intracranial LVO location: terminal ICA with involvement of the M1-segment of the MCA/carotid-T vs. proximal M1-segment vs. distal M1-segments (distal to perforating branches) vs. M2/3-segment(s)<br>• Time window 0-3 vs. > 3-6 hours vs. unknown stroke onset and 0-3 hours since symptom recognition vs. unknown stroke onset and > 3-6 hours since symptom recognition<br>• Tandem stenosis/extracranial occlusion vs. no tandem stenosis/extracranial occlusion<br>• Patients with vs. without silent ischemic lesions	• ASPECTS ≤ 8 on baseline NCCT or ≤ 7 on baseline DWI vs. ASPECTS ≥ 9 on baseline NCCT or ≥ 8 on baseline DWI<br>• intubation/ventilation vs. conscious sedation<br>• IVT vs. no-IVT<br>• age ≤ 60 vs. > 60<br>• age ≤ 70 vs. > 70<br>• age ≤ 80 vs. > 80<br>• NIHSS at baseline < 10 vs. 10-20 vs. > 20, +/- cross-classification with LVO-location<br>• intracranial LVO location: terminal ICA with involvement of the M1-segment of the MCA/carotid-T vs. proximal M1-segment vs. distal M1-segments (distal to perforating branches) vs. M2/3-segment(s)<br>• Time window 0-3 vs. > 3-6 hours vs. unknown stroke onset and 0-3 hours since symptom recognition vs. unknown stroke onset and > 3-6 hours since symptom recognition<br>• Tandem stenosis/extracranial occlusion vs. no tandem stenosis/extracranial occlusion<br>• Patients with vs. without silent ischemic lesions	
**12.4 Statistical Methods (page 82)**		
For sensitivity analysis, a likelihood ratio test for the treatment variable in a linear model with 24-hour infarct core volume as the response and baseline infarct core volume plus stroke subtype classification and baseline hemoglobin levels as additional explanatory variables as well as the factors used for treatment allocation (see Section 8.5.1 Randomization method).<br>The primary analysis will be carried out on the full analysis set. As a sensitivity analysis, the analysis will	For sensitivity analysis, a likelihood ratio test for the treatment variable in a linear model with 24-hour infarct core volume as the response and baseline infarct core volume plus stroke subtype classification and baseline hemoglobin levels as additional explanatory variables as well as the factors used for treatment allocation (see Section 8.5.1 Randomization method).<br>The primary analysis will be carried out on the full analysis set. As a sensitivity analysis, the analysis will	To evaluate whether hemoglobin level do influence NBHO effects

Previous and new wording in track change modus	New wording	Comments/ reasons for substantial amendment
be repeated on the per-protocol subset. Further secondary analyses on the primary variable comprise the analysis restricted on the following subsets of the full analysis set given in Section 12.3 Definition of Trial Population to be analyzed.	be repeated on the per-protocol subset. Further secondary analyses on the primary variable comprise the analysis restricted on the following subsets of the full analysis set given in Section 12.3 Definition of Trial Population to be analyzed.	
**13.1 Data Collection (page 84)**		
All protocol-required information collected during the trial must be entered into the eCRF by the investigator or a designated representative. Patient data will be documented pseudonymously. The investigator, or a designated representative, should complete the eCRF pages as soon as possible after the information is collected, preferably on the same day when a trial subject is seen for an examination, treatment, or any other trial procedure, but latest within 5 working days. Any pending entries must be completed immediately after the final examination. Explanation should be given for all missing data.<br>Brain images of screening, V5 and all unscheduled brain images should be uploaded within 5 working days to the core imaging laboratory Eppdata.	All protocol-required information collected during the trial must be entered into the eCRF by the investigator or a designated representative. Patient data will be documented pseudonymously. The investigator, or a designated representative, should complete the eCRF pages as soon as possible after the information is collected, preferably on the same day when a trial subject is seen for an examination, treatment, or any other trial procedure, but latest within 5 working days. Any pending entries must be completed immediately after the final examination. Explanation should be given for all missing data.<br>Brain images of screening, V5 and all unscheduled brain images should be uploaded within 5 working days to the core imaging laboratory Eppdata.	Adaption of time frame for eCRF completion and imaging upload according to DSMB's requirements in order to allow faster safety evaluation.
**14.5 Subject Information and Informed Consent (pages 86/87)**		
2 If the patient is unable to communicate, this must be documented in the applicable document **("ICF" variant 1)** by the investigator.<br>a. In case, a LAR is available and present, he/she has to be informed about the trial orally and in writing and written informed consent has to be obtained **(ICF Variant 2).**<br>b. In case, no LAR is available at the time of trial inclusion, the patient may then be included in the trial ("deferred consent"), but the investigator, however, should – if possible – consider the patient's	3 If the patient is unable to communicate, this must be documented in the applicable document **("ICF" variant 1)** by the investigator.<br>a. In case, a LAR is available and present, he/she has to be informed about the trial orally and in writing and written informed consent has to be obtained **(ICF Variant 2).**<br>b. In case, no LAR is available at the time of trial inclusion, the patient may then be included in the trial ("deferred consent"), but the investigator, however, should – if possible – consider the patient's	Deletion of the word impartial to match the subsequent revised definition of witness in the protocol v1.4.

Previous and new wording in track change modus	New wording	Comments/ reasons for substantial amendment
presumed will (e.g. by asking a close relative accompanying the patient) when enrolling a patient following this deferred consent procedure. In case, a patient is included by deferred consent, the investigator has to fill out the "assessment of patient's ability to consent form" **("ICF" variant 1),** which also has to be signed by an impartial witness**.**	presumed will (e.g. by asking a close relative accompanying the patient) when enrolling a patient following this deferred consent procedure. In case, a patient is included by deferred consent, the investigator has to fill out the "assessment of patient's ability to consent form" **("ICF" variant 1),** which also has to be signed by a witness**.**	
1. Consent by the study participant (**ICF Variant 3**): The Investigator will obtain a freely given written consent from each subject after an appropriate explanation of the aims, methods, anticipated benefits, potential hazards and any other aspect of the study which is relevant to the subject's decision to participate. The informed consent form must be signed, with name and date noted by the subject, before the subject is exposed to any further study-related procedure. If the patient is unable to write, oral presentation and explanation of the content of the informed consent form and of the data protection information must take place in the presence of an impartial witness. The witness and the physician conducting the informed consent discussions must also sign and personally date the consent document. The witness might be any employee of the trial site who is not part of the study team (i.e. not listed in the delegation log) or any other person not working at the trail site must not be in any way dependent on the sponsor of the trial, the trial site or any member of the investigating team (e. g. an employee at the trial site).	1. Consent by the study participant (**ICF Variant 3**): The Investigator will obtain a freely given written consent from each subject after an appropriate explanation of the aims, methods, anticipated benefits, potential hazards and any other aspect of the study which is relevant to the subject's decision to participate. The informed consent form must be signed, with name and date noted by the subject, before the subject is exposed to any further study-related procedure. If the patient is unable to write, oral presentation and explanation of the content of the informed consent form and of the data protection information must take place in the presence of a witness. The witness and the physician conducting the informed consent discussions must also sign and personally date the consent document.<br>The witness might be any employee of the trial site who is not part of the study team (i.e. not listed in the delegation log) or any other person not working at the trail site.	We defined the word "witness" more generously, realizing that local hospital SOPs prohibited access from external persons including (e.g. relatives) (not only) during hospital admission due to the COVID-10 pandemic related restrictions, so that in some countries like Belgium no patient inclusion had been possible at all given the previous tight definition of witness. A witness can be now any employee who is not part of the study team or any other external person to allow patient enrolment.
**15.2 Data Protection (page 90)**		

Previous and new wording in track change modus	New wording	Comments/ reasons for substantial amendment
The data obtained in the study will be treated pursuant to the General Data Protection Regulation (EU 2016/679). During the clinical trial, subjects will be identified solely by means of their individual identification code (~~subject number, randomization number~~Patient ID). Trial data stored on a computer will be stored in accordance with local data protection law and will be handled in strictest confidence. Distribution of these data to unauthorized persons has to be prevented strictly. The appropriate regulations of local data legislation will be fulfilled in its entirety.	The data obtained in the study will be treated pursuant to the General Data Protection Regulation (EU 2016/679). During the clinical trial, subjects will be identified solely by means of their individual identification code (Patient ID). Trial data stored on a computer will be stored in accordance with local data protection law and will be handled in strictest confidence. Distribution of these data to unauthorized persons has to be prevented strictly. The appropriate regulations of local data legislation will be fulfilled in its entirety.	Protocol was changed to clarify that enrolled patients will solely be identified by their Patient ID.
**15.3 Monitoring (pages 90/91)**		
Remote monitoring: Should in-person monitoring visits not be feasible due to regulatory restrictions (such as current Covid-19 pandemic related restrictions), either a combined remote and on-site monitoring visit or a full remote monitoring visit may be conducted alternatively. Remote source data verification as part of remote monitoring visits needs to be agreed upon between the sponsor, the national sponsor representative and the study site, as well as patients in the ICF. Trial documents (e.g. monitoring plan) will be adjusted to reflect remote activities. The ICH GCP requirements and applicable data protection and privacy regulations must be met in any case and for any selected monitoring approach. In case of a remote verification of source data, the patients need to agree to it in the informed consent form.	Remote monitoring: Should in-person monitoring visits not be feasible due to regulatory restrictions (such as current Covid-19 pandemic related restrictions), either a combined remote and on-site monitoring visit or a full remote monitoring visit may be conducted alternatively. Remote source data verification as part of remote monitoring visits needs to be agreed upon between the sponsor, the national sponsor representative and the study site, as well as patients in the ICF. Trial documents (e.g. monitoring plan) will be adjusted to reflect remote activities. The ICH GCP requirements and applicable data protection and privacy regulations must be met in any case and for any selected monitoring approach. In case of a remote verification of source data, the patients need to agree to it in the informed consent form.	Addition of a paragraph about remote monitoring to ensure that the patient safety and wellbeing and data integrity can be still checked when in-person monitoring is not feasible due to local COVID-19 restrictions.
**17 Signatures (page 93)**		
		Update of academic title
**20 Appendices**		

Previous and new wording in track change modus	New wording	Comments/ reasons for substantial amendment
		Consecutive numbering of all appendices, which led to the correction of numbering and insertion of all appendices in a table of content.

Abbreviations:

ADC	Apparent Diffusion Coefficient
AE	Adverse Event
AESI	Adverse Event of Special Interest
AHA	American Heart Association
ASA	American Stroke Association
ASPECTS	Alberta Stroke Program Early CT score
CA	Competent Authority
CBF	Cerebral Blood Flow
COPD	Chronic Obstructive Pulmonary Disease
COVID-19	Coronavirus Disease 2019
CRF	Case Report Form
CT	Computed Tomography
CTA	Computed Tomography Angiography
DBL	Data Base Lock
DSA	Digital Subtraction Angiography
DSMB	Data Safety Monitoring Board
DWI-MRI	Diffusion Weighted Imaging - Magnetic Resonance Imaging
ECASS III	European Cooperative Acute Stroke Study III
ECG	Electrocardiography
eCRF	electronic Case Report Form
EDTA	Ethylenediamine tetraacetic acid

EKUT	Eberhard Karls Universität Tübingen
END	Early Neurological Deterioration
ENI	Early Neurological Improvement
EQ-5D-5L	EuroQol Questionnaire-five dimensions-five levels of severity
ESMINT	European Society for Minimally Invasive Neurological Therapy
ESO	European Stroke Organisation
$etCO_2$	end-tidal carbon dioxide
EVT	EndoVascular Treatment
FFP2/3	Filtering FacePiece 2/3
FiO_2	Inspiratory oxygen fraction
FLAIR	Fluid-Attenuated Inversion Recovery
FSI	First Subject In
HCO_3^-	Hydrogen carbonate
ICA	Internal Carotid Artery
ICH	Intracranial hemorrhage
ICH GCP	International Council on Harmonization of Technical Requirements for Registration of Pharmaceuticals for Human Use harmonized tripartite guideline on GCP
ICU	Intensive Care Unit
IEAB	Independent Ethical Advisory Board
IMP	Investigational Medicinal Product
IQCODE	Informant Questionnaire on Cognitive Decline in the Elderly
ITT	Intention To Treat
KKS	Koordinierungszentrum für Klinische Studien (Coordination Centre for Clinical Trials)
LAR	Legally Authorized Representative
LND	Late Neurological Deterioration
LSI	Last Subject In
LSO	Last Subject Out

LVO	Large Vessel Occlusion
MACE	Major Adverse Cardiovascular Events
MADRS	Montgomery–Åsberg Depression Rating Scale
MCA	Middle Cerebral Artery
MoCA	Montreal Cognitive Assessment
mRS	modified Rankin Scale
NBHO	NormoBaric HyperOxygenation
NCCT	Non-Contrast Computed Tomography
NIHSS	National Institutes of Health Stroke Scale
$PaCO_2$	Partial pressure of carbon dioxide
PaO_2	Partial pressure of oxygen
POC	Point-of-Care
RCT	Randomized controlled trial
SAE	Serious Adverse Event
SaO_2	Oxygen saturation
SARS-CoV-2	Severe Acute Respiratory Syndrome CoronaVirus 2
SC	Steering Committee
sICH	symptomatic IntraCranial Hemorrhage
SIS-16	Stroke Impact Scale 16
SND	Subacute Neurological Deterioration
SNI	Subacute Neurological Improvement
sNIHSS-EMS	shortened NIHSS for emergency medical services
SpO_2	peripheral oxygen saturation
T2*	T2 star
TBY	Endovascular mechanical thrombectomy
TICI	Thrombolysis in Cerebral Infarction
TOF	time-of-flight

UKL-HD	University Hospital Heidelberg
V	Visit
VEND	Very Early Neurological Deterioration
VENI	Very Early Neurological Improvement
VHIR	Fundacio Hospital Universitari Vall d´Hebron Barcelona – Institut de Recerca, Spain
WP	Work Package

Literature:

1. Purrucker, J.C., et al., Design and validation of a clinical scale for prehospital stroke recognition, severity grading and prediction of large vessel occlusion: the shortened NIH Stroke Scale for emergency medical services. BMJ Open, 2017. 7(9): p. e016893.
54. Liu, S., et al., Electron paramagnetic resonance-guided normobaric hyperoxia treatment protects the brain by maintaining penumbral oxygenation in a rat model of transient focal cerebral ischemia. J Cereb Blood Flow Metab, 2006. 26(10): p. 1274-84.
70. Weaver, J. and K.J. Liu, Does normobaric hyperoxia increase oxidative stress in acute ischemic stroke? A critical review of the literature. Med Gas Res, 2015. 5: p. 11.
71. Flynn, E.P. and R.N. Auer, Eubaric hyperoxemia and experimental cerebral infarction. Ann Neurol, 2002. 52(5): p. 566-72.
76. Singhal, A.B., et al., A pilot study of normobaric oxygen therapy in acute ischemic stroke. Stroke, 2005. 36(4): p. 797-802.
87. Young, P., The association between early arterial oxygenation and mortality in ventilated patients with acute ischaemic stroke. Crit Care Resusc, 2012. 14(1): p. 14-19.
88. Rincon, F., Association between hyperoxia and mortality after stroke: a multicenter cohort study. Crit Care Med, 2014. 42(2): p. 387-396.
101. von Kummer, R., et al., The Heidelberg Bleeding Classification: Classification of Bleeding Events After Ischemic Stroke and Reperfusion Therapy. Stroke, 2015. 46(10): p. 2981-6.
102. Stewart, R.A.H., et al., High flow oxygen and risk of mortality in patients with a suspected acute coronary syndrome: pragmatic, cluster randomised, crossover trial. BMJ, 2021. 372: p. n355.
106. Nogueira, R.G., et al., Thrombectomy 6 to 24 Hours after Stroke with a Mismatch between Deficit and Infarct. N Engl J Med, 2018. 378(1): p. 11-21.
107. Albers, G.W., et al., Thrombectomy for Stroke at 6 to 16 Hours with Selection by Perfusion Imaging. N Engl J Med, 2018. 378(8): p. 708-718.
109. Rha, J.H. and J.L. Saver, The impact of recanalization on ischemic stroke outcome: a meta-analysis. Stroke, 2007. 38(3): p. 967-73.
118. Chan, Y.F., et al., Supplemental oxygen delivery to suspected stroke patients in pre hospital and emergency department settings. Med Gas Res, 2014. 4: p. 16.
147. Mijajlovic, M.D., et al., Hyperbaric oxygen therapy in acute stroke: is it time for Justitia to open her eyes? Neurol Sci, 2020. 41(6): p. 1381-1390.
148. Cozene, B., et al., An Extra Breath of Fresh Air: Hyperbaric Oxygenation as a Stroke Therapeutic. Biomolecules, 2020. 10(9).
149. Cheng, Z., et al., Normobaric oxygen therapy attenuates hyperglycolysis in ischemic stroke. Neural Regen Res, 2021. 16(6): p. 1017-1023.
150. Akca, O., et al., Association of Early Oxygenation Levels with Mortality in Acute Ischemic Stroke - A Retrospective Cohort Study. J Stroke Cerebrovasc Dis, 2020. 29(2): p. 104556.
151. Cheng, Z., et al., Adjuvant High-Flow Normobaric Oxygen After Mechanical Thrombectomy for Anterior Circulation Stroke: a Randomized Clinical Trial. Neurotherapeutics, 2021.
152. Beker, M.C., et al., Effects of normobaric oxygen and melatonin on reperfusion injury: role of cerebral microcirculation. Oncotarget, 2015. 6(31): p. 30604-14.
153. Neuberger, U., et al., Risk factors of intracranial hemorrhage after mechanical thrombectomy of anterior circulation ischemic stroke. Neuroradiology, 2019. 61(4): p. 461-469.
154. Chen, S., et al., One-year outcomes of supersaturated oxygen therapy in acute anterior myocardial infarction: The IC-HOT study. Catheter Cardiovasc Interv, 2020.

155. Andell, P., et al., Oxygen therapy in suspected acute myocardial infarction and concurrent normoxemic chronic obstructive pulmonary disease: a prespecified subgroup analysis from the DETO2X-AMI trial. Eur Heart J Acute Cardiovasc Care, 2020. 9(8): p. 984-992.
159. Turc, G., et al., European Stroke Organisation (ESO) - European Society for Minimally Invasive Neurological Therapy (ESMINT) Guidelines on Mechanical Thrombectomy in Acute Ischemic Stroke. J Neurointerv Surg, 2019.
160. Powers, W.J., et al., Guidelines for the Early Management of Patients With Acute Ischemic Stroke: 2019 Update to the 2018 Guidelines for the Early Management of Acute Ischemic Stroke: A Guideline for Healthcare Professionals From the American Heart Association/American Stroke Association. Stroke, 2019. 50(12): p. e344-e418.
161. Taves, D.R., Minimization: a new method of assigning patients to treatment and control groups. Clin Pharmacol Ther, 1974. 15(5): p. 443-53.
165. Knauth, M., et al., Potential of CT angiography in acute ischemic stroke. AJNR Am J Neuroradiol, 1997. 18(6): p. 1001-10.
166. d'Esterre, C.D., et al., Regional Comparison of Multiphase Computed Tomographic Angiography and Computed Tomographic Perfusion for Prediction of Tissue Fate in Ischemic Stroke. Stroke, 2017. 48(4): p. 939-945.
167. Almekhlafi, M.A., et al., Imaging Triage of Patients with Late-Window (6-24 Hours) Acute Ischemic Stroke: A Comparative Study Using Multiphase CT Angiography versus CT Perfusion. AJNR Am J Neuroradiol, 2020. 41(1): p. 129-133.
168. Lee, S.J., et al., Optimal Multiphase Computed Tomographic Angiography-based Infarct Core Estimations for Acute Ischemic Stroke. Sci Rep, 2019. 9(1): p. 15243.
176. Jorm, A.F., The Informant Questionnaire on cognitive decline in the elderly (IQCODE): a review. Int Psychogeriatr, 2004. 16(3): p. 275-93.
189. Chaisinanunkul, N., et al., Adopting a Patient-Centered Approach to Primary Outcome Analysis of Acute Stroke Trials Using a Utility-Weighted Modified Rankin Scale. Stroke, 2015. 46(8): p. 2238-43.
190. Higashida, R.T., et al., Trial design and reporting standards for intra-arterial cerebral thrombolysis for acute ischemic stroke. Stroke, 2003. 34(8): p. e109-37.
191. Santos, E.M.M., et al., Added value of multiphase CTA imaging for thrombus perviousness assessment. Neuroradiology, 2018. 60(1): p. 71-79.
192. Chen, Z., et al., Thrombus Permeability on Dynamic CTA Predicts Good Outcome after Reperfusion Therapy. AJNR Am J Neuroradiol, 2018. 39(10): p. 1854-1859.
193. Tan, I.Y., et al., CT angiography clot burden score and collateral score: correlation with clinical and radiologic outcomes in acute middle cerebral artery infarct. AJNR Am J Neuroradiol, 2009. 30(3): p. 525-31.
