# Supplementary material for: Penumbral Rescue by normobaric O = O administration in patients with ischemic stroke and target mismatch proFile (PROOF): Study protocol of a phase IIb trial
Source: Int J Stroke. 2023 Aug 18;19(1):120–6. doi: 10.1177/17474930231185275 (PMC10759237; doi:10.1177/17474930231185275)
Supplement: sj-pdf-6-wso-10.1177_17474930231185275 – Supplemental material for Penumbral Rescue by normobaric O = O administration in patients with ischemic stroke and target mismatch proFile (PROOF): Study protocol of a phase IIb trial [file sj-pdf-6-wso-10.1177_17474930231185275.pdf]

Supplemental Material to

Penumbral **R**escue by Normobaric **O=O** Administration in Patients with Ischemic Stroke and Target Mismatch Pro**F**ile (PROOF): Study Protocol of a Phase IIb Trial

Author Affiliations:

[1] Department of Neurology & Stroke, Eberhard-Karls University, Tübingen, Germany
[2] Hertie Institute for Clinical Brain Research, Eberhard-Karls University, Tübingen, Germany
[3] Department of Neurology, Hôpital Sainte-Anne, Université de Paris, Paris, France
[4] Department of Neurology, Massachusetts General Hospital, Harvard Medical School, Boston, MA, United States of America
[5] Department of Neurology, Helsinki University Hospital and University of Helsinki, Finland
[6] Department of Neurology, Vall d'Hebron University Hospital, Barcelona, Spain
[7] Department of Neurosciences, Experimental Neurology, KU Leuven, University of Leuven, Leuven, Belgium
[8] Department of Neurology, University Hospitals Leuven, Leuven, Belgium
[9] Department of Neurology, GHU Paris Psychiatrie et Neurosciences INSERM U1266 Université Paris Cité FHU NeuroVasc, Paris, France
[10] Department of Neurology, St. Anne's University Hospital Brno and Masaryk University, Brno, Czech Republic
[11] Neurosciences Cliniques, Centre Hospitalier Universitaire Vaudois, Lausanne, Switzerland
[12] Department of Clinical Neuroscience, Institute of Neuroscience and Physiology, Sahlgrenska Academy at University of Gothenburg, Gothenburg, Sweden
[13] Department of Neurology, Sahlgrenska University Hospital, Gothenburg, Sweden
[14] Department of Neurology and Center for Stroke Research Berlin, Charité Universitätsmedizin Berlin, Berlin, Germany
[15] Institute for Stroke and Dementia Research (ISD), University Hospital, LMU Munich, Munich, Germany
[16] Munich Cluster for Systems Neurology (SyNergy), Munich, Germany
[17] German Center for Neurodegenerative Diseases (DZNE, Munich), Munich, Germany
[18] German Centre for Cardiovascular Research (DZHK, Munich), Munich, Germany
[19] Department of Neurology, Alfried Krupp Hospital, Essen, Germany
[20] Department of Brain Sciences, Imperial College London, London, United Kingdom
[21] Coordinating Centre for Clinical Trials, University of Heidelberg, Heidelberg, Germany
[22] Landeskrebsregister Nordrhein-Westfalen, Bochum, Germany
[23] Center for Rare Diseases, Eberhard-Karls University, Tübingen, Germany
[24] Neuroradiology, University Hospital Hamburg-Eppendorf, Hamburg, Germany
[25] Eppdata GmbH, Hamburg, Germany
[26] Vall d'Hebron Institut de Recerca, Neurovascular Research Lab, Barcelona, Spain,
[27] Department of Anesthesiology and Intensive Care Medicine, Eberhard-Karls University, Tübingen, Germany

PROOF Investigators who meet the criteria for authorship

**Name, academic degree**	**Affiliation**	**Contribution**
Adedolapo Kamaldeen Adeyemi, MD	Department of Neurology & Stroke, Eberhard-Karls University, Tübingen, Germany	Acquisition of data, critical review
Katharina Althaus, MD	Department of Neurology, University Hospital of Ulm, Germany	Acquisition of data, critical review
Juan Francisco Arenillas, MD PhD	Hospital Clinico Universitario de Valladolid, Valladolid, Spain	Acquisition of data, critical review
Heinrich J. Audebert, MD	Center for Stroke Research Berlin, Charité, University Berlin, Germany	Scientific advice, critical review
Jean-Claude Baron, MD PhD	Department of Neurology, Hôpital Sainte-Anne, Université de Paris, Paris, France	Trial conceptualization and preparation, scientific advice, critical review
Benjamin Bender, MD	Department of diagnostic and interventional Neuroradiology, Eberhard-Karls University, Tübingen, Germany	Acquisition of data, critical review
Frank Benedikt, MD	Department of Neurology, University Hospital Essen, Essen, Germany	Acquisition of data, critical review
Gabriel Broocks, MD	Department of Neuroradiology, University Hospital Hamburg-Eppendorf, Hamburg, Germany	Acquisition of data, critical review
Ina Burghaus, PhD	Coordinating Centre for Clinical Trials, University of Heidelberg, Heidelberg, Germany	Design of methodology, critical review
Pere Cardona Portela, MD PhD	Department of Neurology, Hospital University de Bellvitge, Barcelona, Spain	Acquisition of data, critical review
Milani Deb-Chatterji, MD	Department of Neurology, University Hospital Hamburg-Eppendorf, Hamburg, Germany	Acquisition of data, critical review
Martina Cviková	Department of Neurology, St. Anne's University Hospital in Brno, Faculty of Medicine Masaryk University, Brno, Czech Republic	Acquisition of data, critical review
Luc Defreyne, MD PhD	Department of Vascular and Interventional Radiology, Ghent University Hospital, Ghent, Belgium	Acquisition of data, critical review
Veerle De Herdt, MD PhD	Department of Neurology, Ghent University Hospital, Ghent, Belgium	Acquisition of data, critical review
Olivier Detante, MD PhD	Neurology, CHU Grenoble Alpes, Grenoble, France; Univ. Grenoble Alpes, Inserm, U1216, Grenoble Institut Neurosciences, Grenoble, France	Acquisition of data, critical review
Martin Dichgans, MD PhD	Institute for Stroke and Dementia Research (ISD), University Hospital, LMU Munich, Munich, Germany; Munich Cluster for Systems Neurology	Scientific advice, critical review

	(SyNergy), Munich, Germany; German Center for Neurodegenerative Diseases (DZNE, Munich), Munich, Germany; German Centre for Cardiovascular Research (DZHK, Munich), Munich, Germany	
Ulrike Ernemann, MD	Department of diagnostic and interventional Neuroradiology, Eberhard-Karls University, Tübingen, Germany	Critical review
Jens Fiehler, MD	Department of Neuroradiology, University Hospital Hamburg-Eppendorf, Hamburg, Germany; Eppdata GmbH, Hamburg, Germany	Trial preparation, lead core lab (imaging), steering committee, critical review
Fabian Flottmann, MD	Department of Neuroradiology, University Hospital Hamburg-Eppendorf, Hamburg, Germany	Acquisition of data, critical review
Lídia García Guillamón	Vall d'Hebron Institut de Recerca, Barcelona, Spain	Acquisition of data, critical review
Monika Glauch	Center for Rare Diseases, Eberhard-Karls University, Tübingen, Germany; Research Management, University of Tuebingen, Tuebingen, Germany	Trial preparation, project management, critical review
Alexandra Gomez Exposito, MD	Department of Neurology & Stroke, Eberhard-Karls University, Tübingen, Germany	Acquisition of data, critical review
Benjamin Gory, MD PhD	Department of Diagnostic and Therapeutic Neuroradiology, Centre Hospital Regional Universitaire de Nancy, Université de Lorraine, INSERM U1254, Nancy, France	Acquisition of data, critical review
Holm Graeßner, PhD	Center for Rare Diseases, Eberhard-Karls University, Tübingen, Germany; Research Management, University of Tuebingen, Tuebingen, Germany	Funding acquisition, trial preparation, project management, steering committee, critical review
Sylvie Grand, MD	Univ. Grenoble Alpes, Inserm, U1216, Grenoble Institut Neurosciences, Grenoble, France; Neuroradiology / MRI department, CHU Grenoble Alpes, Grenoble, France	Acquisition of data, critical review
Michal Haršány, MD PhD	Department of Neurology, St. Anne's University Hospital in Brno, Faculty of Medicine Masaryk University, Brno, Czech Republic; International Clinical Research Centre, St. Anne's University Hospital in Brno, Brno, Czech Republic	Acquisition of data, critical review
Florian Härtig, MD	Department of Anesthesiology and Intensive Care Medicine, Eberhard-Karls University, Tübingen, Germany	Funding acquisition, trial conceptualization, trial preparation, critical review
Till Karsten Hauser, MD	Department of diagnostic and interventional Neuroradiology, Eberhard-Karls University, Tübingen, Germany	Acquisition of data, critical review

Olivier Heck, MD	Neuroradiology / MRI department, CHU Grenoble Alpes, Grenoble, France	Acquisition of data, critical review
Dimitri Hemelsoet, MD	Department of Neurology, Ghent University Hospital, Ghent, Belgium	Acquisition of data, critical review
Florian Hennersdorf, MD	Department of diagnostic and interventional Neuroradiology, Eberhard-Karls University, Tübingen, Germany	Acquisition of data, critical review
Julia Hoppe	Department of Neurology, University Hospital Hamburg-Eppendorf, Hamburg, Germany	Acquisition of data, critical review
Johannes Hüsing, PhD	Coordinating Centre for Clinical Trials, University of Heidelberg, Heidelberg, Germany; Landeskrebsregister Nordrhein-Westfalen, Bochum, Germany	Design of methodology, steering committee, critical review
Pia Kalmbach	Department of Neurology & Stroke, Eberhard-Karls University, Tübingen, Germany	Acquisition of data, critical review
Lars Kellert, MD	Department of Neurology, Ludwig Maximilian University, LMU, Munich, Germany	Acquisition of data, critical review
Martin Köhrmann, MD	Department of Neurology, University Hospital Essen, Essen, Germany	Acquisition of data, critical review
Markus Kowarik, MD	Department of Neurology & Stroke, Eberhard-Karls University, Tübingen, Germany; Hertie Institute for Clinical Brain Research, Eberhard-Karls University, Tübingen, Germany	Acquisition of data, critical review
Blanca Lara Rodriguez, MD	Department of Neurology, Hospital University de Bellvitge, Barcelona, Spain	Acquisition of data, critical review
Loic Legris, MD	Neurology, CHU Grenoble Alpes, Grenoble, France; Univ. Grenoble Alpes, Inserm, U1216, Grenoble Institut Neurosciences, Grenoble, France	Acquisition of data, critical review
Robin Lemmens, MD PhD	Department of Neurosciences, Experimental Neurology, KU Leuven, University of Leuven, Leuven, Belgium; Department of Neurology, University Hospitals Leuven, Leuven, Belgium	Trial preparation, national coordinator (Belgium), critical review
Tobias Lindig, MD	Department of diagnostic and interventional Neuroradiology, Eberhard-Karls University, Tübingen, Germany	Acquisition of data, critical review
Steffen P. Luntz, MD	Coordinating Centre for Clinical Trials, University of Heidelberg, Heidelberg, Germany	Project management, critical review
Jay Lusk, Research Fellow	Duke University School of Medicine, Durham, NC, USA	Scientific advice, critical review
Brian Mac Grory, MD	Duke Clinical Research Institute, Durham, NC, USA<br>Department of Neurology, Duke University School of Medicine, Durham, NC, USA	Scientific advice, critical review

Andreas Manger, MD	Department of Anesthesiology and Intensive Care Medicine, Eberhard-Karls University, Tübingen, Germany	Acquisition of data, critical review
Nicolas Martinez-Majander, MD PhD	Department of Neurology, Helsinki University Hospital, University of Helsinki, Helsinki, Finland	Acquisition of data, critical review
Joshua Mbroh, MD	Department of Neurology & Stroke, Eberhard-Karls University, Tübingen, Germany	Acquisition of data, critical review
Annerose Mengel, MD	Department of Neurology & Stroke, Eberhard-Karls University, Tübingen, Germany	Acquisition of data, critical review
Johannes Meyne, MD	Department of Neurology, University Hospital Schleswig-Holstein, Kiel, Germany	Acquisition of data, critical review
Patrik Michel, MD PhD	Neurosciences Cliniques, Centre Hospitalier Universitaire Vaudois, Lausanne, Switzerland	Trial preparation, national coordinator (Switzerland), critical review
Robert Mikulik, MD PhD	Department of Neurology, St. Anne's University Hospital Brno, Brno, Czech Republic	Trial preparation, national coordinator (Czech Republic), acquisition of data, critical review
Carlos Molina Cateriano, MD PhD	Department of Neurology, Vall d'Hebron University Hospital, Barcelona, Spain	Trial preparation, national coordinator (Spain), acquisition of data, critical review
Joan Montaner, MD PhD	Vall d'Hebron Institut de Recerca, Neurovascular Research Lab, Barcelona, Spain	Trial preparation, lead core lab (biomarker), steering committee, acquisition of data, critical review
Susanne Müller, MD	Department of Neurology, University Hospital of Ulm, Germany	Acquisition of data, critical review
Sibu Mundiyanapurath, MD	Department of Neurology, Heidelberg University Hospital, Heidelberg, Germany	Acquisition of data, critical review
Olivier Naggara, MD PhD	Department of Neuroradiology, GHU Paris Psychiatrie et Neurosciences INSERM U1266 Université Paris Cité	Acquisition of data, critical review
Krassen Nedeltchev, MD PhD	Department of Neurology, Kantonspital Aarau, Aarau, and University of Bern, Switzerland	Critical review
Thanh N. Nguyen, MD PhD	Department of Radiology, Boston Medical Center, Boston, Massachusetts, USA; Department of Neurology, Boston Medical Center, Boston, Massachusetts, USA	Scientific advice, critical review
Maike A. Nilsson, PhD	Coordinating Centre for Clinical Trials, University of Heidelberg, Heidelberg, Germany	Trial preparation, project management, critical review

Michael Obadia, MD PhD	Department of Neurology and Stroke Center, Hôpital fondation Adolphe de Rothschild, Paris, France	Acquisition of data, critical review
Khouloud Poli, MD	Department of Neurology & Stroke, Eberhard-Karls University, Tübingen, Germany	Acquisition of data, critical review
Sven Poli, MD MSc	Department of Neurology & Stroke, Eberhard-Karls University, Tübingen, Germany; Hertie Institute for Clinical Brain Research, Eberhard-Karls University, Tübingen, Germany	Funding acquisition, trial conceptualization and preparation, principal investigator, project management, steering committee, drafting of manuscript, acquisition of data, critical review
Jan C. Purrucker, MD MSc	Department of Neurology, Heidelberg University Hospital, Heidelberg, Germany	Acquisition of data, critical review
Silja Räty, MD PhD	Department of Neurology, Helsinki University Hospital, University of Helsinki, Helsinki, Finland	Acquisition of data, critical review
Sébastian Richard, MD PhD	Centre Hospital Regional Universitaire de Nancy, Nancy, France	Acquisition of data, critical review
Hardy Richter, MD	Department of Infectiology, Eberhard-Karls-University, Tuebingen, Germany	Acquisition of data, critical review
Clotilde Schilte, MD	Department of Anaesthesia and Critical Care, CHU Grenoble Alpes, Grenoble, France	Acquisition of data, critical review
Eckhard Schlemm, MBBS PhD	Department of Neurology, University Medical Center Hamburg-Eppendorf, Hamburg, Germany	Acquisition of data, critical review
Aneesh B. Singhal, MD	Department of Neurology, Massachusetts General Hospital, Harvard Medical School, Boston, USA	Trial conceptualization and preparation, scientific advice, critical review
Linda Stöhr, PhD	European Clinical Research Infrastructure Network (ECRIN), Paris, France	Trial preparation, project management, critical review
Benjamin Stolte, MD	Department of Neurology, University Hospital Essen, Essen, Germany	Acquisition of data, critical review
Daniel Strbian, MD PhD	Department of Neurology, Helsinki University Hospital, University of Helsinki, Helsinki, Finland	Trial preparation, national coordinator (Finland), critical review
Marek Sykora, MD PhD	Department of Neurology, St. John's Hospital, Vienna, Austria	Scientific advice, critical review
Turgut Tatlisumak, MD PhD	Department of Neurology, Helsinki University Hospital and University of Helsinki, Finland; Department of Clinical Neuroscience, Institute of Neuroscience and Physiology, Sahlgrenska Academy at University of Gothenburg, Gothenburg, Sweden; Department of Neurology,	Trial preparation, national coordinator (Sweden), critical review

	Sahlgrenska University Hospital, Gothenburg, Sweden	
Götz Thomalla, MD	Department of Neurology, University Hospital Hamburg-Eppendorf, Hamburg, Germany	Acquisition of data, critical review
Liisa Tomppo, MD PhD	Department of Neurology, Helsinki University Hospital, University of Helsinki, Helsinki, Finland	Acquisition of data, critical review
Johannes Tünnerhoff, MD	Department of Neurology & Stroke, Eberhard-Karls University, Tübingen, Germany	Drafting of manuscript, scientific advice, critical review
Guillaume Turc, MD PhD	Department of Neurology, Hôpital Sainte-Anne, Université de Paris, Paris, France; Department of Neurology, GHU Paris Psychiatrie et Neurosciences INSERM U1266 Université Paris Cité FHU NeuroVasc, Paris, France	Trial preparation, national coordinator (France), acquisition of data, critical review
Noel van Horn	Department of Neuroradiology, University Hospital Hamburg-Eppendorf, Hamburg, Germany	Acquisition of data, critical review
Roland Veltkamp, MD	Department of Neurology, Alfried Krupp Hospital, Essen, Germany; Department of Brain Sciences, Imperial College London, London, UK	Scientific advice, critical review
Julia Zeller	Department of Neurology & Stroke, Eberhard-Karls University, Tübingen, Germany	Project management, acquisition of data, critical review
Ulf Ziemann, MD	Department of Neurology & Stroke, Eberhard-Karls University, Tübingen, Germany; Hertie Institute for Clinical Brain Research, Eberhard-Karls University, Tübingen, Germany	Critical review
Christine S. Zuern, MD	Department of Cardiology, Universitätsspital Basel, Switzerland	Trial preparation, scientific advice, critical review

Further PROOF Investigators

Name, academic degree	Affiliation	Contribution
Wagih Ben Hassen, MD PhD	Department of Neuroradiology, GHU Paris Psychiatrie et Neurosciences INSERM U1266 Université Paris Cité, Paris, France	Acquisition of data
Thorsten Benz	Coordinating Centre for Clinical Trials, University of Heidelberg, Heidelberg, Germany	Data management
Ansgar Brandhorst	Coordinating Centre for Clinical Trials, University of Heidelberg, Heidelberg, Germany	Data management
Judith Cendrero	Vall d'Hebron Institut de Recerca, Barcelona, Spain	Acquisition of data
Bastian Cheng, MD	Department of Neurology, University Hospital Hamburg-Eppendorf, Hamburg, Germany	Acquisition of data
Pauline Cuisenier, MD	Neurology, CHU Grenoble Alpes, Grenoble, France	Acquisition of data
Isabelle Favre-Wiki, MD	Neurology, CHU Grenoble Alpes, Grenoble, France	Acquisition of data
Katia Garambois, MD	Neurology, CHU Grenoble Alpes, Grenoble, France	Acquisition of data
Märit Jensen, MD	Department of Neurology, University Hospital Hamburg-Eppendorf, Hamburg, Germany	Acquisition of data
Ana Nuñez Guillen	Department of Neurology, Hospital University de Bellvitge, Barcelona, Spain	Acquisition of data
Andres Paipa Merchan	Department of Neurology, Hospital University de Bellvitge, Barcelona, Spain	Acquisition of data
Elena Pala	Vall d'Hebron Institut de Recerca, Neurovascular Research Lab, Barcelona, Spain	Acquisition of data
Olalla Pancorbo	Vall d'Hebron Institut de Recerca, Barcelona, Spain	Acquisition of data
Ana Penalba Morenilla	Vall d'Hebron Institut de Recerca, Neurovascular Research Lab, Barcelona, Spain	Acquisition of data
Helena Quesada Garcia	Department of Neurology, Hospital University de Bellvitge, Barcelona, Spain	Acquisition of data
Vivien Richter, MD	Department of diagnostic and interventional Neuroradiology, Eberhard-Karls University, Tübingen, Germany	Acquisition of data
Maximilian Schell, MD	Department of Neurology, University Hospital Hamburg-Eppendorf, Hamburg, Germany	Acquisition of data
Andrea Schirmer	Department of Neurology, University Hospital of Ulm, Germany	Acquisition of data

Gerli Sibolt	Department of Neurology, Helsinki University Hospital, University of Helsinki, Helsinki, Finland	Acquisition of data
Wendy Stoop, MSc	Department of Neurology, Ghent University Hospital, Ghent, Belgium	Acquisition of data
Johannes Wischmann, MD	Department of Neurology, Ludwig Maximilian University, LMU, Munich, Germany	Acquisition of data
Umberto Zanolini, MD	Department of Neuroradiology, University Hospital Hamburg-Eppendorf, Hamburg, Germany	Acquisition of data

Independent Ethical Advisory Board

Peter Rosenberger, MD (chair)	Department of Anesthesiology and Intensive Care Medicine, Eberhard-Karls University, Tübingen, Germany
Matthew Schrag, MD PhD	Department of Neurology, Vanderbilt University School of Medicine, Nashville, USA
Gary Randal, PhD	Stroke Alliance for Europe (SAFE), Brussels, Belgium
Eivind Berge, MD PhD (deceased)	Department of Cardiology, Oslo University Hospital, Oslo, Norway

Data Safety Monitoring Board

Werner Hacke, MD PhD (chair)	Department of Neurology, Heidelberg University Hospital, Heidelberg, Germany
Valeria Caso, MD PhD	Stroke Unit, Santa Maria Della Misericordia Hospital, University of Perugia, Perugia, Italy
David Petroff, PhD	Clinical Trial Centre Leipzig, Faculty of Medicine, Leipzig University, Germany
Christine Roffe, MD PhD	Stroke Research in Stoke, Faculty of Medicine & Health Sciences, Keele University, Staffordshire, UK
Gerhard Schroth, MD	Department of Neuroradiology, University of Bern, Bern, Switzerland

Disclosures

Katharina Althaus	Speakers' honoraria/consulting fees from Alexion, BMS/Pfizer, Daiichi Sankyo, Portola (all outside of the submitted work)
Benjamin Bender	Co-founder and CTO of AIRAmed GmbH, Tübingen (outside of the submitted work)
Gabriel Brooks	Consultant for Eppdata, Consultant for Balt
Milani Deb-Chatterji	Research grants from the Werner Otto Stiftung (outside of the submitted work)
Fabian Flottmann	Consultant for Eppdata GmbH (outside of the submitted work)
Lars Kellert	Funding for travel or speaker honoraria from Alexion, AstraZeneca, Bayer Vital, Boehringer Ingelheim, Bristol-Meyer-Squibb, Daiichi Sankyo and Pfizer, and funding for research from Boehringer Ingelheim (all outside of the submitted work)
Robin Lemmens	No personal disclosures but institutional fees for consultancy from iSchemaView (outside of the submitted work)
Tobias Lindig	Co-founder and CTO of AIRAmed GmbH, Tübingen (outside of the submitted work)
Johannes Meyne	Speakers' honoraria/consulting fees from Boehringer-Ingelheim, BMS/Pfizer and Daiichi Sankyo (outside of the submitted work)
Krassen Nedeltechev	Advisory boards: Bayer, Boehringer Ingelheim, Daichii Sankyo, BMS/Pfizer and unrestricted educational grants: Bayer, Daichii Sankyo, BMS/Pfizer, Alexion (all outside of the submitted work)
Thanh N. Nguyen	Research support from SVIN, Medtronic, advisory board for Idorsia (all outside of the submitted work)
Sven Poli	Research support from BMS/Pfizer, Boehringer-Ingelheim, Daiichi Sankyo, German Federal Joint Committee Innovation Fund, and German Federal Ministry of Education and Research, Helena Laboratories and Werfen as well as speakers' honoraria/consulting fees from Alexion, AstraZeneca, Bayer, Boehringer-Ingelheim, BMS/Pfizer, Daiichi Sankyo, Portola, and Werfen (all outside of the submitted work).
Jan C. Purrucker	Consultation fees and travel expenses from Akcea, Bayer, Boehringer Ingelheim, Daiichi Sankyo, and BMS/Pfizer (all outside of the submitted work)
Sébastian Richard	Consultation fees from Boehringer Ingelheim France, Bristol-Meyers-Squibb, and Pfizer SAS
Aneesh B. Singhal	Supported by NIH-NINDS (U10-NS086729) (outside of the submitted work)
Benjamin Stolte	Speakers' honoraria and consulting fees from Alexion and Biogen (all outside of the submitted work)
Daniel Strbian	Advisory board participation for AstraZeneca and unrestricted educational grant from Boehringer Ingelheim (all outside of the submitted work)
Turgut Tatlisumak	Academic grants from the University of Gothenburg, Salhgrenska University Hospital, European Union, Sigrid Juselius Foundation, and Wennerström's Foundation as well as personal fees from Bayer, Boehringer Ingelheim, Bristol Myers Squibb, Inventiva, and Portola Pharma (all outside of the submitted work)

Götz Thomalla	Consultation and lecturer fees from Acandis, Alexion, Amazon, Bayer, Boehringer Ingelheim, Bristol Myers Squibb, Daiichii Sankyo, Portola, Stryker, and grant support from the European Union, German Research Foundation (DFG), German Federal Ministry of Education and Research (BMBF), and the German Innovation Fund (all outside of the submitted work)
Ulf Ziemann	Research grants from BMS, European Research Council, German Federal Ministry of Education and Research, German Research Foundation, Janssen Pharmaceuticals, and Takeda, and personal consulting fees from Bayer, CorTec, and Pfizer (all outside the submitted work)

All other authors did report no disclosures and no conflict of interest.
