# Supplementary material for: Penumbral Rescue by normobaric O = O administration in patients with ischemic stroke and target mismatch proFile (PROOF): Study protocol of a phase IIb trial
Source: Int J Stroke. 2023 Aug 18;19(1):120–6. doi: 10.1177/17474930231185275 (PMC10759237; doi:10.1177/17474930231185275)
Supplement: sj-pdf-7-wso-10.1177_17474930231185275 – Supplemental material for Penumbral Rescue by normobaric O = O administration in patients with ischemic stroke and target mismatch proFile (PROOF): Study protocol of a phase IIb trial [file sj-pdf-7-wso-10.1177_17474930231185275.pdf]

# CLINICAL TRIAL PROTOCOL

## PROOF: "Penumbral Rescue by Normobaric O=O Administration in Patients with Ischemic Stroke and Target Mismatch ProFile: A Phase II Proof-of-Concept Trial"

| | |
| --- | --- |
| **Phase of study:** | Phase II – proof-of-concept |
| **EudraCT No.:** | 2017-001355-31 |
| **Study Registry Number:** | NCT03500939 |


**Acknowledgment**

This work is part of the project "PROOF - Penumbral Rescue by Normobaric O=O Administration in Patients with Ischemic Stroke and Target Mismatch ProFile: A Phase II Proof-of-Concept Trial"

Supported by the European Commission HORIZON 2020, contract no. 733379. The opinions expressed and arguments employed herein do not necessarily reflect the official views of the European Commission.


**GCP Statement:** The study will be conducted in compliance with Good Clinical Practices (ICH-GCP) and the Declaration of Helsinki, and in accordance with applicable legal and regulatory requirements, including archiving of essential documents.


**CONFIDENTIAL:** This protocol contains confidential information and is intended solely for the guidance of the clinical investigation. This protocol may not be disclosed to parties not associated with the clinical investigation or used for any purpose without the prior written consent of the Principal Investigator/ Coordinating Investigator.

# ADMINISTRATIVE STRUCTURE


**Sponsor:**

Eberhard-Karls University Tübingen
Medical Faculty represented by University Hospital Tübingen and its Commercial Director:
Dipl.-Volksw. Gabriele Sonntag
Geissweg 5
72076 Tübingen
Germany
Phone: 0049 7071 29 82005
Fax: 0049 7071 29 3966
Email: gabriele.sonntag@med.uni-tuebingen.de

**Coordinating Investigator (Leiter der klinischen Prüfung*)**

University Hospital Tübingen
Dept. of Neurology with Focus on Neurovascular Diseases and Neurooncology
PD Dr. med. Sven Poli
Hoppe-Seyler-Str. 3
72076 Tübingen
Germany
Phone: 0049 172 4682284
Fax: 0049 7071 29 25047
Email: sven.poli@uni-tuebingen.de


* According to § 40 German Drug Law (AMG)


**Project Management:**

Coordination Centre for Clinical trials (KKS)
Dr. Maike Nilsson, Ph.D.
Marsilius-Arkaden / Turm West
Im Neuenheimer Feld 130.3
69120 Heidelberg
Germany
Phone: 0049 6221 56 32671
Fax: 0049 6221 56 33508
Email: maike.nilsson@med.uni-heidelberg.de

**Biometrics:**

Coordination Centre for Clinical trials (KKS)
Dr. rer. medic. Johannes Hüsing
Marsilius-Arkaden / Turm West
Im Neuenheimer Feld 130.3
69120 Heidelberg
Germany
Phone: 0049 6221 56 34506
Fax: 0049 6221 56 33508
Email: johannes.huesing@med.uni-heidelberg.de

**Data Management:**

Coordination Centre for Clinical trials (KKS)
Evelin Deeg (lead)
Marsilius-Arkaden / Turm West
Im Neuenheimer Feld 130.3
69120 Heidelberg
Germany
Phone: 0049 6221 56 36215
Fax: 0049 6221 56 33508
Email: evelin.deeg@med.uni-heidelberg.de

**Pharmacovigilance:**

Coordination Centre for Clinical trials (KKS)
Julia Merkle-Lock
Marsilius-Arkaden / Turm West
Im Neuenheimer Feld 130.3
69120 Heidelberg
Germany
Phone: 0049 6221 56 35117
Fax: **0049 6221 56 33725 (SAE-FAX!)**
Email: pharmakovigilanz.KKS@med.uni-heidelberg.de

**Lead Monitoring**

Coordination Centre for Clinical trials (KKS)

Dr. Karsten Thelen (lead)
Marsilius-Arkaden / Turm West
Im Neuenheimer Feld 130.3
69120 Heidelberg
Germany
Phone: 0049 6221 56 35622
Fax: 0049 6221 56 33508
Email: karsten.thelen@med.uni-heidelberg.de

**Central Contact to local CROs**

ECRIN (European Clinical Research Infrastructure Network)

Dr. rer. nat. Linda Stoehr
European Correspondent for ECRIN
Alt-Moabit 96a
10559 Berlin
Germany
Phone: 0049 30 4053 5400
Fax: 0049 30 6959 9990
Email: linda.stoehr@kksn.de

**Data and Safety Monitoring Board (DSMB)**

Prof. Dr. med. Werner Hacke (Chair)
Im Neuenheimer Feld 400
69120 Heidelberg
Germany
Phone: 0049 6221 56 8211
Fax: 0049 6221 56 5348
Email: werner.hacke@med.uni-heidelberg.de

Prof. Dr. Valeria Caso
Piazza Menghini 1
06129 Perugia
Italy
Phone: 0039 3398371093
Fax: 0039 075578 2765
Email: vcaso@hotmail.com

Prof. Dr. Christine Roffe
1 Thornburrow Drive
ST4 7QB, Stoke-on-Trent
United Kingdom
Phone: 0044 1782 671655
Fax: 0044 1782 671619
Email: christine.roffe@uhnm.nhs.uk

Prof. Dr. med. Gerhard Schroth
Freiburgstrasse 4
3010, Bern
Switzerland
Phone:  0041 31 632 7890
Fax: 0041 31 632 4872
Email: gerhard.schroth@insel.ch

Dr. rer. nat. habil. David Petroff
Härtelstr. 16-18
04107, Leipzig
Germany
Phone: 0049 341 97 16354
Fax: 0049 341 97 16189
Email: david.petroff@zks.uni-leipzig.de

## Independent Ethical Advisory Board (IEAB)

Prof. Dr. med. Peter Rosenberger (Chair)
Hoppe-Seyler-Str. 3
72076 Tübingen
Germany
Phone: 0049 7071 29 86622

Fax: 0049 7071 29 5533
Email: peter.rosenberger@med.uni-tuebingen.de

Prof. Dr. Matthew Schrag
Vanderbilt University School of Medicine
Medical Research Building III
465 21st Avenue S, Suite 6160 (Office 6158C)
Nashville, TN 37240
United States of America
Email: matthew.schrag@vanderbilt.edu

Dr. Gary Randall
Church Lane
B61 8RA Bromsgrove
United Kingdom
Phone: 0044 1527 903905
Email: Gary.randall@stroke.org.uk

## Scientific Advisory Board (SAB)

Prof. Dr. Jean-Claude Baron (Chair)
1 Rue Cabanis
75014, Paris
France
Phone: 0033 1 45656268
email: jean-claude.baron@inserm.fr

Prof. Dr. Aneesh Singhal (Co-Chair)
55 Fruit Street
02114, Boston (MA)
United States of America
Phone: 001 617 726 1728
Email: asinghal@mgh.harvard.edu

Prof. Dr. med. Heinrich Audebert
Hindenburgdamm 30
12200, Berlin
Germany
Phone: 0049 30 8445 2277
Fax: 0049 30 8445 4264
Email: heinrich.audebert@charite.de

Prof. Dr. med. Martin Dichgans
Feodor-Lynen-Straße 17
81377, Munich
Germany
Phone: 0049 89 4400 46019
Fax: 0049 89 4400 46040
Email: martin.dichgans@med.uni-muenchen.de

Prof. Dr. med. Roland Veltkamp
Imperial College, South Kensington Campus
SW7 2AZ, London
United Kingdom
Phone: 0044 20 3313 1566

Email: r.veltkamp@imperial.ac.uk

**Steering Committee (SC)**


Eberhard-Karls Universität Tübingen
Dr. med. Sven Poli
Hoppe-Seyler-Straße 3
72076 Tübingen
Germany
Phone: 0049 7071 29 83106
Fax: 0049 7071 29 25047
Email: sven.poli@uni-tuebingen.de

Eberhard-Karls Universität Tübingen
Dr. Holm Graessner
Calwerstr. 7
72076 Tübingen
Germany
Phone: 0049 7071 29 72308
Fax: 0049 7071 29 25061
Email: holm.graessner@med.uni-tuebingen.de

Coordination Centre for Clinical trials (KKS)
Dr. rer. medic. Johannes Hüsing
Im Neuenheimer Feld 130.3
69120 Heidelberg
Germany
Phone: 0049 6221 56 34506
Fax: 0049 6221 56 33508
Email: johannes.huesing@med.uni-heidelberg.de

Eppdata GmbH
Dr. Frosti Palsson, PhD
Lokstedter Steindamm 18
22529 Hamburg
Germany
Phone: 0049 40 7410 – 25960
Fax: 0049 40 7410 – 40114
Email: f.palsson@eppdata.de

Fundatio Hospital Universitari Vall d'Hebron
Dr. Joan Montaner, MD PhD
Passeig de la Vall d'Hebron, 119-129
08035, Barcelona
Spain
Phone: 0034 934 894 073
Fax: 0034 934 894 015
Email: Joan.montaner@vhir.org


**Central technical facilities to be involved in the conduct of the trial, in which the measurement or assessment of the main evaluation criteria are centralized:**

***CORE IMAGING LABORATORY***


Eppdata GmbH
Dr. Frosti Palsson, PhD
Lokstedter Steindamm 18
22529 Hamburg
Germany
Phone: 0049 40 7410 – 25960


***CORE BIOMARKER LABORATORY***


Fundatio Hospital Universitari Vall d'Hebron
Dr. Joan Montaner, MD PhD
Passeig de la Vall d'Hebron, 119-129
08035 Barcelona
Spain
Phone: 0034 934 894 073

Fax: 0049 40 7410 – 40114
Email: f.palsson@eppdata.de

Fax: 0034 934 894 015
Email: joan.montaner@vhir.org

## National Coordinators

***BELGIUM***
Katholieke Universiteit Leuven
Prof. Dr. Robin Lemmens
Oude Markt 13
Leuven 3000
Email: robin.lemmens@uzleuven.be

***CZECH REPUBLIC***
Fakultni Nemocnice U SV. Anny V Brne
Prof. Dr. Robert Mikulik
Pekarska 53
Brno 65691
Email: robert.mikulik@fnusa.cz

***FRANCE***
Centre Hospitalier Saint Anne De Paris
Prof. Dr. Guillaume Turc
Rue Cabanis 1
75674 Paris
Email: g.turc@ch-sainte-anne.fr

***FINLAND***
Helsinki University Hospital
Prof. Dr. Daniel Strbian
15675350, Stenbackinkatu 9
Helsinki 00029
Email: daniel.strbian@hus.fi

***SPAIN***
Hospital Universitari Vall d´Hebron
Prof. Dr. Carlos A. Molina, MD, PhD
Passeig de la Vall d'Hebron, 119-129
08035 Barcelona
Email: cmolina@vhebron.net

***SWITZERLAND***
Centre Hospitalier Universitaire Vaudois
Prof. Dr. Patrik Michel
Rue du Bugnon 46
1011 Lausanne
Email: patrik.michel@chuv.ch

## Participating Sites

All participating sites will be listed in a separate document.

# TABLE OF CONTENTS


**1 PROTOCOL SYNOPSIS .......... 10**

**2 TRIAL SCHEDULE .......... 14**

**3 ABBREVIATIONS .......... 16**

**4 INTRODUCTION .......... 20**

4.1 Scientific Background .......... 20
4.2 Trial Rationale/ Justification .......... 23
4.3 Risk-benefit Assessment .......... 24
***4.3.1 Risk-Benefit Assessment Considering the Literature until December 6th, 2019*** .......... *29*
***4.3.2 Updated Risk-Benefit Assessment Considering the Literature until March 17th, 2021*** *46*
***4.3.3 Risk-Benefit Assessment of the PROOF trial during the COVID-19 pandemic*** .......... *47*
4.4 Data and Safety Monitoring Board (DSMB) .......... 48
4.5 Steering Committee (SC) .......... 48

**5 TRIAL OBJECTIVES AND ENDPOINTS .......... 50**

5.1 Primary Objective and Primary Endpoint .......... 50
5.2 Secondary Objectives and Secondary Endpoints .......... 50

**6 TRIAL DESIGN AND DESCRIPTION .......... 52**

6.1 Trial design .......... 52
6.2 Trial Duration and Schedule .......... 52

**7 SELECTION OF SUBJECTS AND CENTRES .......... 53**

7.1 Number of Subjects .......... 53
7.2 Centers .......... 53
7.3 General Criteria for Subjects' Selection .......... 53
7.4 Inclusion Criteria .......... 53
7.5 Exclusion Criteria .......... 53
7.6 Pre-specified Measures in Case of Slow Recruitment .......... 54
7.7 Criteria for Withdrawal from the Trial or Discontinuation of Treatment .......... 54
***7.7.1 Withdrawal of Patients from the Trial*** .......... *54*
***7.7.2 Discontinuation of Trial Treatment*** .......... *55*
***7.7.3 Premature Closure of the Clinical Trial or a Site*** .......... *56*

**8 INVESTIGATIONAL MEDICINAL PRODUCT (IMP) .......... 58**

8.1 Study medication .......... 58
***8.1.1 General Information*** .......... *58*
***8.1.2 Characterization of study medication*** .......... *58*
8.2 Packaging and Labeling .......... 58
8.3 Supplies and Drug Accountability .......... 58
8.4 Administration of study medication .......... 58
***8.4.1 Assignment of Identification Codes*** .......... *58*
***8.4.2 Dosage Schedule*** .......... *58*
***8.4.3 Compliance*** .......... *59*
***8.4.4 Prior and Concomitant Diseases*** .......... *61*
***8.4.5 Prior and Concomitant Medication*** .......... *61*
***8.4.6 Adjustments to dosage of the IMP in the individual trial subject*** .......... *61*
8.5 Randomization and Blinding .......... 61
***8.5.1 Randomization method*** .......... *61*
***8.5.2 Blinding procedure*** .......... *62*
***8.5.3 Unblinding*** .......... *62*
***8.5.4 Emergency Treatment*** .......... *62*

**9 DESCRIPTION OF TRIAL VISITS .......... 63**

9.1 Pre-Screening .......... 63
9.2 Screening Visit .......... 63
9.3 Randomization .......... 63
9.4 V1 – Initiation of study treatment .......... 64

9.5 V2 – ≥ 5 MINUTES AFTER START OF NBHO (OR RANDOMIZATION IN THE CONTROL ARM) AND BEFORE START OF ENDOVASCULAR INTERVENTION ..... 64
9.6 V3 – DURING TBY ..... 64
9.7 V4 – END OF STUDY TREATMENT ..... 64
9.8 V5 – 24 ±6 HOURS (DAY 1) AFTER START OF NBHO (OR RANDOMIZATION IN THE CONTROL ARM)..... 65
9.9 V6 – DAY 5 ±2 AFTER START OF NBHO (OR RANDOMIZATION IN THE CONTROL ARM) OR AT DISCHARGE (WHICHEVER OCCURS FIRST) ..... 65
9.10 V7 – DAY 90 ±10 AFTER START OF NBHO (OR RANDOMIZATION IN THE CONTROL ARM)..... 66

**10 METHODS OF DATA COLLECTION ..... 67**
10.1 PHYSICAL/NEUROLOGICAL EXAMINATION ..... 67
10.2 VITAL SIGNS ..... 67
10.3 NATIONAL INSTITUTE OF HEALTH STROKE SCALE (NIHSS) ..... 67
10.4 BRAIN IMAGING ACQUISITION AND ASSESSMENT ..... 67
10.5 STROKE SUBTYPE CLASSIFICATION ..... 68
10.6 LENGTH OF ICU/HOSPITAL STAY / DURATION OF VENTILATION ..... 68
10.7 MODIFIED RANKIN SCALE (MRS) ..... 68
10.8 BARTHEL INDEX ..... 68
10.9 IQCODE (INFORMANT QUESTIONNAIRE ON COGNITIVE DECLINE IN THE ELDERLY) ..... 68
10.10 MONTREAL COGNITIVE ASSESSMENT (MOCA) ..... 69
10.11 STROKE IMPACT SCALE (SIS)-16 ..... 69
10.12 EQ-5D-5L ..... 69
10.13 MONTGOMERY–ÅSBERG DEPRESSION RATING SCALE (MADRS) ..... 69
10.14 TYMPANIC TEMPERATURE ..... 69
10.15 OXYGEN ADMINISTRATION ..... 69
10.16 CONCOMITANT MEDICATION AND INVASIVE PROCEDURES ..... 70
10.17 12-LEAD ECG ..... 70
10.18 SAFETY LABORATORY ASSESSMENT ..... 70
10.19 BIOMARKERS (PHARMACODYNAMICS) SUBSTUDY ..... 71
10.20 ARTERIAL BLOOD GASES (PHARMACOKINETICS) ..... 72
10.21 (SERIOUS) ADVERSE EVENTS ..... 72

**11 ADVERSE EVENTS ..... 73**
11.1 DEFINITIONS ..... 73
***11.1.1 Adverse Event ..... 73***
***11.1.2 Serious Adverse Events and Adverse Events of Special Interest ..... 73***
***11.1.3 Serious Adverse Reaction ..... 74***
***11.1.4 Expectedness ..... 74***
***11.1.5 Suspected Unexpected Serious Adverse Reaction (SUSAR) ..... 74***
***11.1.6 Grading of AEs ..... 74***
***11.1.7 Relationship and Outcome of AEs ..... 75***
11.2 PERIOD OF OBSERVATION AND DOCUMENTATION ..... 76
11.3 REPORTING OF SERIOUS ADVERSE EVENTS BY INVESTIGATOR ..... 76
11.4 EXPEDITED REPORTING ..... 76

**12 STATISTICAL PROCEDURES ..... 78**
12.1 SAMPLE SIZE CALCULATION ..... 78
12.2 ANALYSIS VARIABLES ..... 78
***12.2.1 Primary analysis variable ..... 78***
***12.2.2 Secondary analysis variables ..... 79***
12.3 DEFINITION OF TRIAL POPULATION TO BE ANALYZED ..... 81
12.4 STATISTICAL METHODS ..... 82
12.5 INTERIM ANALYSES ..... 82

**13 DATA MANAGEMENT ..... 84**
13.1 DATA COLLECTION ..... 84
13.2 DATA HANDLING ..... 84
13.3 ARCHIVING OF ESSENTIAL DOCUMENTS ..... 84

**14 ETHICAL AND LEGAL ASPECTS ..... 85**
14.1 GOOD CLINICAL PRACTICE ..... 85
14.2 LEGAL BASES ..... 85
***14.2.1 Declaration of Helsinki*** ..... *85*
***14.2.2 Other Legal Bases*** ..... *85*
14.3 APPROVAL OF TRIAL PROTOCOL AND AMENDMENTS ..... 85
14.4 NOTIFICATION OF REGULATORY AUTHORITIES ..... 85
14.5 SUBJECT INFORMATION AND INFORMED CONSENT ..... 86
14.6 INSURANCE ..... 88
14.7 CONTINUOUS INFORMATION TO THE ETHICS COMMITTEE AND THE COMPETENT AUTHORITY ..... 88
**15 QUALITY CONTROL AND QUALITY ASSURANCE ..... 90**
15.1 DIRECT ACCESS TO SOURCE DOCUMENTS ACCORDING TO ICH GCP ..... 90
15.2 DATA PROTECTION ..... 90
15.3 MONITORING ..... 90
15.4 INSPECTIONS AND AUDITS ..... 91
15.5 RESPONSIBILITIES OF THE INVESTIGATOR ..... 91
**16 ADMINISTRATIVE AGREEMENTS ..... 92**
16.1 FINANCING OF THE TRIAL ..... 92
16.2 FINANCIAL DISCLOSURE ..... 92
16.3 REPORTS ..... 92
16.4 REGISTRATION OF THE TRIAL ..... 92
16.5 PUBLICATION ..... 92
16.6 INFORMATION OF PATIENTS ABOUT TRIAL RESULTS ..... 92
**17 SIGNATURES ..... 93**
**18 DECLARATION OF INVESTIGATOR ..... 94**
**19 REFERENCES ..... 95**
**20 APPENDICES ..... I**

# 1 PROTOCOL SYNOPSIS

| | |
|---|---|
| **TITLE** | Penumbral Rescue by Normobaric O=O Administration in Patients with ischemic Stroke and Target Mismatch ProFile: A Phase II Proof-of-Concept Trial |
| **CLINICAL TRIAL CODE** | PROOF |
| **EUDRACT NO.** | 2017-001355-31 |
| **INDICATION** | Acute ischemic stroke<br>MedDRA-code: 10061256 and 10055221 ICD-code: I63.3/4 |
| **OBJECTIVES** | The main objective of the PROOF trial is to investigate efficacy and safety of normobaric hyperoxygenation (NBHO) as a neuroprotective treatment in patients with acute ischemic stroke due to large vessel occlusion likely to receive endovascular mechanical thrombectomy (TBY) in a randomized controlled clinical phase IIb trial. The trial is designed as a proof-of-concept study, replicating insights from those preclinical studies in which NBHO showed positive effects: early NBHO initiation, and transient ischemia (i.e. NBHO treatment as an adjunct until timely and successful reperfusion).<br>Primary objective and endpoint:<br>To demonstrate an effect of NBHO on penumbral salvage in ischemic stroke.<br>The primary endpoint is the ischemic core growth defined as the difference in ischemic core volume (in mL) from baseline to 24 hours; intention-to-treat analysis.<br>Secondary objectives and endpoints:<br>To show clinical efficacy and safety of NBHO adjunct to standard treatment. The following endpoints will be collected and compared between treatment groups.<br>Key secondary efficacy endpoint is the change in National Institutes of Health Stroke Scale (NIHSS) scores from baseline to 24 hours.<br>Secondary clinical efficacy endpoints: Survival at visit 6 (V6), and V7; NIHSS score (neurological status) at V2, V4, V5, V6, and V7; modified Rankin Scale (mRS) score, and Barthel Index score at V6 and V7; MoCA at V7 (controlled for Informant Questionnaire on Cognitive Decline in the Elderly (IQCODE) at V5), Stroke Impact Scale 16 (SIS-16), EuroQoL Questionnaire (EQ-5D-5L), and Montgomery–Åsberg Depression Rating Scale (MADRS) scores at V7; $PaO_2$ at V3, and V5.<br>Clinical safety endpoints: all-cause death at V6, and V7; stroke related death at V6, and V7; symptomatic intracranial hemorrhage until V6; vital signs (systolic and diastolic blood pressure, heart and respiratory rate, peripheral capillary oxygen saturation ($SpO_2$) and end-tidal carbon dioxide ($etCO_2$) at V1-V7, with body temperature at V1, V5, and V6; 12-lead electrocardiogram (ECG) at V1, and V5; laboratory (blood count, clinical chemistry, coagulation) at V5, and V6; length of ICU stay, hospital stay, and duration of ventilation at V6, and V7; concomitant invasive procedures (e.g. intravenous/intra-arterial thrombolysis, thrombectomy, stenting, carotid surgery, decompressive hemicraniectomy, cardioversion, patent foramen ovale (PFO) closure) until V7.<br>Secondary imaging efficacy endpoints: relative changes in ischemic core volume (in %) from baseline to 24 hours; absolute and relative ischemic core change from baseline to 24 hours using either NCCT or DWI-MRI (or |

| | |
|---|---|
| | CT angiography source images and DWI) for ischemic core estimation at baseline; absolute and relative ischemic core change from baseline to 24 hours using cerebral blood flow (CBF) < 30% for ischemic core estimation at baseline in all patients, independent of imaging modality; penumbral salvage from baseline to 24 hours; TICI (Thrombolysis in Cerebral Infarction perfusion scale grade) in patients who underwent TBY; revascularization on 24-hour follow-up imaging.<br><br>Imaging safety endpoints: new microbleeds on 24-hour follow-up imaging; any intracranial hemorrhage on 24-hour follow-up imaging; peri-interventional occurrence of vasospasms; ischemic lesions in new territories on 24-hour follow-up imaging.<br><br>Exploratory objectives:<br>• Exploratory analyses of imaging (to be defined in imaging protocol and Interpretation Guidelines).<br>• Exploratory analyses of biochemical biomarkers to develop a blood-based test to monitor efficacy and safety of NBHO treatment |
| **PHASE** | Clinical Phase IIb |
| **INVESTIGATIONAL MEDICINAL PRODUCT** | IMP: NBHO, i.e. inhalation of 100% oxygen at high flow (≥ 40 L/min) via a sealed non-rebreather face-mask with reservoir, or in case of intubation/ventilation for (study-independent) TBY, ventilation with an inspiratory oxygen fraction ($FiO_2$) of 1.0. NBHO is started within 6 hours of stroke symptom onset (witnessed) or symptom recognition (in case of wake-up or unknown onset stroke), and within 30 minutes after end of baseline brain imaging and applied until the end of TBY procedure (defined by removal of guide catheter from sheath) or, in case TBY is not attempted (defined as 'TBY was not attempted or intervention was stopped prior to any penetration or aspiration of the qualifying (i.e. intracranial) LVO'), 4 hours after start of study treatment.<br><br>Control: oxygen supplementation if $SpO_2$ ≤ 94% at 2 to 4 L/min via nasal cannula according to guidelines of the European Stroke Organisation (ESO), or in case of TBY-related intubation/ventilation, ventilation with an initial $FiO_2$ of 0.3 to be gradually increased if $SpO_2$ ≤ 94%. |
| **REFERENCE DRUG** | N/A |
| **STUDY DESIGN** | Prospective, multicenter, adaptive phase IIb, parallel group, randomized (1:1), standard treatment-controlled, open-label, clinical trial with blinded endpoint assessment (PROBE design). |
| **STUDY POPULATION** | Inclusion Criteria<br>• Age: ≥ 18 years<br>• Acute anterior circulation ischemic stroke due to a Large vessel occlusion (LVO) on CT or MR angiography, i.e. either **terminal internal carotid artery (ICA)** with M1/carotid-T, **proximal M1**, **distal M1** (distal to perforating branches), or **M2/3 segment(s)**<br>• **If TBY is likely to be conducted**<br>• NIHSS score of ≥ 6 at screening<br>• Alberta Stroke Program Early CT score (ASPECTS) of 6-10 on non-contrast CT or 5-10 on diffusion-weighted MRI (DWI-MRI)<br>• If recommended by the attending physician, CT or MR perfusion should be performed prior to NBHO |

| | |
|---|---|
| | • NBHO can be initiated within 6 hours of symptom onset (witnessed) or symptom recognition (in case of wake-up or unknown onset stroke), and within 30 minutes after last image of baseline brain imaging<br>• Pre-stroke mRS of 0 to 2<br>• Breastfeeding women must stop breastfeeding after randomization<br>• Own written informed consent is not obtained prior to study inclusion but has to be gained as soon as possible. Patients who are able to give consent will be informed about trial participation orally and may consent to or decline participation. Patients unable to give consent will be enrolled through a deferred consent procedure (see Section 14.5 Subject Information and Informed Consent)<br><br>Exclusion Criteria<br><br>Neurological:<br><br>• TBY procedure initiated (groin puncture) prior to randomization<br>• Rapid major improvement in neurological status prior to randomization<br>• Any condition which precludes obtaining an accurate baseline NIHSS or outcome assessment (e.g. seizures, dementia, psychiatric or neuromuscular disease)<br>• Intracranial hemorrhage (except of cerebral microbleeds), intracranial tumor (except small meningioma), and/or intracranial arteriovenous malformation<br>• Intracranial aneurysm or prior stent implantation in the vascular territory (upstream and downstream) affected by qualifying LVO<br>• Suspected complete common carotid artery (CCA) occlusion, aortic dissection, cerebral vasculitis, septic embolism, or bacterial endocarditis<br>• Acute bilateral stroke or stroke in multiple vascular territories (except of clinically silent lesions)<br><br>Respiratory:<br><br>• Acute or chronic pulmonary disease or respiratory distress that may, in the clinical judgement of the investigator, interfere with the study intervention (e.g. acute pneumonia, COPD flare-up etc.)<br>• Prior to enrolment, > 2 L/min oxygen required to maintain peripheral oxygen saturation ≥ 95%<br><br>Other:<br><br>• Clinical suspicion of acute myocardial infarction (e.g. acute chest pain)<br>• Baseline blood glucose of < 50 mg/dL (2.78 mmol) or > 400 mg/dL (22.20 mmol)<br>• Body temperature ≥ 38.0°C at screening<br>• History of severe allergy (more than rash) to contrast medium<br>• Current treatment with nitrofurantoin or amiodaron, paraquat poisoning, or history of treatment with bleomycin<br>• Pregnancy at screening, to be excluded (β-HCG in serum or urine) in all women ≤ 55 years except if surgically sterile; in women > 55 years pregnancy must be excluded only in case of increased probability e.g. due to in-vitro fertilization<br>• Any co-existing or terminal disease (except qualifying stroke) with anticipated life expectancy of less than 6 months<br>• Any pre-existing condition that may, in the clinical judgment of the investigator, not allow safe participation in the study (e.g. alcohol or substance abuse, co-existing disease)<br>• Participation in another interventional (drug or device) study within the last four weeks |

| | |
|---|---|
| | • Prior participation in the PROOF trial |
| **SAMPLE SIZE** | 456 subjects, i.e. 228 subjects per arm (subject to reconsideration at interim analysis at 160 subjects included) |
| **TRIAL DURATION** | Total trial duration: 84 months<br>Duration of clinical phase: 48 months<br>Beginning of the preparation phase: Q1 2017<br>FSI (first subject in): August 2019<br>LSI (last subject in): Q1 2023<br>LSO (last subject out): Q2 2023<br>DBL (database lock): Q3 2023<br>Statistical analyses completed: Q4 2023<br>Trial report completed: Q4 2023 |
| **STATISTICAL ANALYSIS** | Efficacy of NBHO treatment: ITT analysis of difference of ischemic core growth (defined as the difference in ischemic core volume (in mL) from baseline to 24 hours) between groups; brain tissue not included in CT perfusion (or, in case CT perfusion is not available or of insufficient quality, CT angiography source images) or MR diffusion at baseline will be excluded from lesion volume measurements.<br>Clinical efficacy of NBHO treatment: ITT-analysis of the difference of change in NIHSS score from baseline to 24 hours (key secondary efficacy endpoint) between groups.<br>For both endpoints: Parameter test on treatment group variable in linear model with treatment allocation factors and treatment as explanatory variables. Re-randomization test as similar to a permutation test mimicking the allocation on the same sequence of patients.<br>Adverse events will be tabulated against treatment group using number of events and number of subjects with events. |
| **FINANCING** | The trial will be financed using funds of the SC1-PM-09-2016 grant by the European Commission within the Horizon 2020 program. |
| **SUBTRIALS** | • Brain Imaging and outcome Assessments (Work Package (WP) 5 according to grant by the European Commission. Details are described in Imaging Protocol and Image Interpretation Guidelines<br>• Biomarkers (WP6); see Section 10.19 |

# 2 TRIAL SCHEDULE

<table>
<tr><th></th><th>S</th><th>R</th><th>V1[1]</th><th>V2[2]</th><th>V3[3]</th><th>V4[4]</th><th>V5[5]</th><th>V6[6]</th><th>V7[5,7]</th></tr>
<tr><td>Test and Assessments</td><td></td><td></td><td>Start of study treatment (Day 0, hour 0)</td><td>Before TBY[2]</td><td>During TBY[3]</td><td>End of study treatment[4]</td><td>24 ±6 hours (Day 1)</td><td>Day 5 ±2/ discharge</td><td>Day 90 ±10 days</td></tr>
<tr><td>Deferred consent</td><td>X</td><td></td><td></td><td></td><td></td><td></td><td></td><td></td><td></td></tr>
<tr><td>Informed consent</td><td>(X)[8]</td><td></td><td></td><td></td><td></td><td></td><td colspan="3">X[8]</td></tr>
<tr><td>Demographics and medical history</td><td>X</td><td></td><td></td><td></td><td></td><td></td><td></td><td></td><td></td></tr>
<tr><td>Physical and neurological examination</td><td>X</td><td></td><td></td><td></td><td></td><td>X</td><td>X</td><td>X</td><td>X[7]</td></tr>
<tr><td>NIHSS</td><td>X</td><td></td><td></td><td>X[2]</td><td></td><td>X</td><td>X[5]</td><td>X</td><td>X[7]</td></tr>
<tr><td>Brain imaging</td><td>X[9,10]</td><td></td><td></td><td></td><td></td><td></td><td>X[10,11]<br>18-72 h</td><td></td><td></td></tr>
<tr><td>Check inclusion / exclusion criteria</td><td>X</td><td></td><td></td><td></td><td></td><td></td><td></td><td></td><td></td></tr>
<tr><td>Confirm eligibility & randomization</td><td></td><td>X</td><td></td><td></td><td></td><td></td><td></td><td></td><td></td></tr>
<tr><td>Study treatment administration</td><td></td><td></td><td colspan="4">-------------------------X-------------------------</td><td></td><td></td><td></td></tr>
<tr><td>Stroke subtype classification[12]</td><td></td><td></td><td></td><td></td><td></td><td></td><td></td><td>X</td><td>X</td></tr>
<tr><td>Length of ICU/hospital stay / duration of ventilation[13]</td><td></td><td></td><td></td><td></td><td></td><td></td><td></td><td>X</td><td>X</td></tr>
<tr><td>mRS, Barthel Index</td><td></td><td></td><td></td><td></td><td></td><td></td><td></td><td>X</td><td>X[5]</td></tr>
<tr><td>IQCODE</td><td></td><td></td><td></td><td></td><td></td><td></td><td>X[14]</td><td></td><td></td></tr>
<tr><td>MoCA, SIS-16, EQ-5D-5L, MADRS</td><td></td><td></td><td></td><td></td><td></td><td></td><td></td><td></td><td>X</td></tr>
<tr><td>12-lead ECG</td><td></td><td></td><td>X[15]</td><td></td><td></td><td></td><td>X</td><td></td><td></td></tr>
<tr><td>Vital signs[16]</td><td>X</td><td></td><td colspan="4">-------------------------X-------------------------</td><td>X</td><td>X</td><td>X[7]</td></tr>
<tr><td>Recording of oxygen administration (reason, flow-rate/$FiO_2$, mask-type/method)[17]</td><td>X[18]</td><td></td><td colspan="5">---------------------------------X---------------------------------</td><td>X</td><td>X</td></tr>
<tr><td>Tympanic temperature</td><td>X</td><td></td><td></td><td></td><td></td><td></td><td>X</td><td>X</td><td></td></tr>
<tr><td>Laboratory assessment[19]</td><td>X[20]</td><td></td><td></td><td></td><td></td><td></td><td>X[20]</td><td>X</td><td></td></tr>
<tr><td>Arterial blood gases</td><td></td><td></td><td></td><td></td><td>X[21]</td><td></td><td>X[21]</td><td></td><td></td></tr>
<tr><td>Concomitant medication and invasive procedures[22]</td><td>X</td><td></td><td>X</td><td>X</td><td>X</td><td>X</td><td>X</td><td>X</td><td>X</td></tr>
<tr><td>AE reporting</td><td></td><td></td><td>X</td><td>X</td><td>X</td><td>X</td><td>X</td><td>X</td><td>X[23]</td></tr>
<tr><td>SAE reporting</td><td></td><td></td><td>X</td><td>X</td><td>X</td><td>X</td><td>X</td><td>X</td><td>X</td></tr>
<tr><td>Biomarkers (WP6)[24]</td><td>X[25]</td><td></td><td></td><td></td><td></td><td></td><td>X[25]</td><td></td><td></td></tr>
<tr><td>Pregnancy test[26]</td><td>X</td><td></td><td></td><td></td><td></td><td></td><td></td><td></td><td></td></tr>
<tr><td colspan="10">S = screening, R = randomization, V = visit, TBY = endovascular mechanical Thrombectomy</td></tr>
</table>

1. Start of study treatment is defined as start of NBHO or time of randomization for the control group.
2. V2 is to be performed ≥ 5 min after start of NBHO or randomization, and **before** start of endovascular intervention including sedation and/or endotracheal intubation/mechanical ventilation, if V2 assessment does not interfere with routine stroke management. Shortened NIHSS for emergency medical services (sNIHSS-EMS [1]) may be chosen over full NIHSS assessment to speed up V2 assessment.
3. V3 is to be performed during NBHO or control treatment, respectively, either **during** TBY procedure (i.e. time from groin puncture to removal of guide catheter from sheath) or, in case TBY is not attempted, 90 ±30 min after randomization.
4. V4 is to be performed within 60 min after end of TBY procedure (defined by **removal of guide catheter from sheath**) or, in case TBY is not attempted (defined as 'TBY was not attempted or intervention was stopped prior to any penetration or aspiration of the qualifying (i.e. intracranial) LVO'), 4 hours ±15 minutes after start of NBHO (or time of randomization for the control group), or earlier, in case study treatment is prematurely terminated.
5. NIHSS assessment at V5 and mRS assessment at V7 should be performed by an investigator blinded to study treatment
6. V6 is to be performed on day 5 ±2, or earlier if subject is to be discharged, but must occur prior to discharge.
7. In case a face-to-face visit is not feasible, a phone interview should be performed at V7. Latter excludes physical and neurological examination incl. NIHSS and assessment of vital signs.
8. Informed Consent has to be obtained as soon as possible; appointment of a legally authorized representative (LAR) must be initiated within 72 hours after randomization.
9. Brain imaging modality at screening as per standard care, i.e. CT- or MRI-based. Minimal standard care includes non-contrast CT, CT angiography and, if recommended by the attending physician, CT perfusion, or DWI, apparent diffusion coefficient (ADC), fluid-attenuated inversion recovery (FLAIR), T2*, time-of-flight (TOF) angiography and, if recommended by the attending physician, gadolinium-enhanced MR perfusion, respectively.
   eASPECTS software (Brainomix, Oxford, UK), or a comparable application, is recommended for automated and fast determination of ASPECTS (see inclusion criteria).
10. Extracranial CT- or MRI-based vessel imaging at baseline (not required for study participation, see inclusion criteria), digital subtraction angiography during TBY procedure as well as any other additional (study-independent) brain or vessel imaging performed during the course of the study (until day 30) must be provided to the imaging core lab.
11. MRI follow-up brain imaging for infarct core volume assessment must be performed in between 18 and 72 h after baseline brain imaging, and should include DWI, ADC, FLAIR, T2*, TOF angiography as minimal standard. If MRI is not feasible in between 18 and 72 h, MRI is to be performed the soonest possible until discharge.
12. Should be completed at V6, but, if results are incomplete, it may be completed at V7. Stroke etiology as classified by Trial of Org 10172 in Acute Stroke Treatment (TOAST) criteria.
13. Intensive care unit (ICU) is defined as a ward with capacity for mechanical ventilation and/or continuous monitoring of vital parameters (including stroke units); invasive and non-invasive mechanical ventilation is reported separately.
14. IQCODE (Informant Questionnaire on Cognitive Decline in the Elderly): closest relatives should be interviewed (face-to-face or by phone) within 48 hours after stroke onset.
15. Baseline 12-lead ECG should be recorded before or within 6 h after start of study treatment.
16. Close neurological observation for at least 24 h is routinely conducted in all stroke patients. Vital signs (systolic and diastolic blood pressure, heart rate and respiratory rate, $SpO_2$, and – if available – $etCO_2$) will be recorded every 30 minutes (±10) for the first 6 hours, then at V5, V6, and V7. Pathologic vital signs in between the visits will be assessed as AE or SAE depending on seriousness.
17. Oxygen administration including reason, flow-rate or $FiO_2$ in ventilated patients, and mask-type/method will be continuously recorded until end of hour 24, i.e. every change of oxygen administration must be documented. Additionally, $SpO_2$, and – if available – $etCO_2$ must be documented at each time point of change of oxygen administration.
18. All available pre-randomization changes of oxygen administration should be documented (including pre-hospital $O_2$ administration) with reason, flow-rate, mask-type, duration, $SpO_2$, and – if available – $etCO_2$.
19. Laboratory parameters to be assessed: full blood count (white blood cells, platelet count, erythrocytes, hemoglobin, hematocrit), coagulation (international normalized ratio (INR), activated partial thromboplastin time (aPTT), D-dimers), and blood chemistry (sodium, potassium, creatinine, urea, uric acid, total bilirubin, direct bilirubin, total protein, albumin, C-reactive protein, troponin I or T, brain natriuretic peptide (BNP) or N-terminal prohormone of brain natriuretic peptide (NT-proBNP), creatine kinase (CK), aspartate transaminase (AST), alanine transaminase (ALT), alkaline phosphatase, lactate dehydrogenase (LDH), gamma-glutamyl transpeptidase (GGT), thyroid-stimulating hormone (TSH), and glucose)
20. Blood sampling (from an indwelling arterial/venous catheter or via venipuncture) at screening and V5 are regarded as study-independent procedures.
21. Arterial blood gases (pH, $PaO_2$, arterial oxygen saturation ($SaO_2$), $PaCO_2$, actual $HCO_3^-$, and actual base excess) only in patients with study-independent arterial access (e.g. for TBY procedure)
22. Any invasive procedure (e.g. intravenous/intra-arterial thrombolysis, thrombectomy, stenting, carotid surgery, decompressive hemicraniectomy, cardioversion, patent foramen ovale (PFO) closure)
23. All AEs including SAEs have to be documented until V6. After that, only non-serious AEs with a causal relationship to the IMP and all SAEs have to be documented (see Section 11.2 for details).
24. Compare Section 10.19 and Biomarker sample handling instructions
25. Blood for biomarkers should be drawn only from patients with study-independent venous or arterial access. It has to be assured, that the samples for biomarker assessment are not shipped to the central lab before the patient or his/her LAR gives consent. In case the patient / LAR does not consent, samples have to be discarded.
26. Urine or serum β-HCG, in women ≤ 55 years except if surgically sterile; in women >55 years pregnancy must be excluded only in case of increased probability e.g. due to in-vitro fertilization

# 3 ABBREVIATIONS

| | |
|---|---|
| ACS | Acute coronary syndromes |
| ADC | Apparent diffusion coefficient |
| AE | Adverse Event |
| AESI | Adverse Event of Special Interest |
| ALT | Alanine aminotransferase |
| AMG | Arzneimittelgesetz (German Drug Law) |
| AMI | Acute myocardial infarction |
| aPTT | Activated partial thromboplastin time |
| ASPECTS | Alberta Stroke Program Early CT score |
| AST | Aspartate aminotransferase |
| ATC | Anatomical-Therapeutic-Chemical Code, part of WHO-DRL (Drug Reference List) |
| β-hCG | Beta-Human chorionic gonadotropin |
| BI | Barthel Index |
| BNP | brain natriuretic peptide |
| CBF | Cerebral blood flow |
| CBV | Cerebral blood volume |
| CCA | Common carotid artery |
| CK | Creatine kinase |
| COPD | Chronic obstructive pulmonary disease |
| COVID-19 | Coronavirus Disease 2019 |
| CRA | Clinical Research Associate |
| CRF | Case Report Form |
| CRO | Contract Research Organisation |
| CT | Computed tomography |
| CTA | Computed Tomography Angiography |
| CTCAE | Common Terminology Criteria for Adverse Events |
| CV | Curriculum Vitae |
| DBL | Data Base Lock |
| DSA | Digital subtraction angiography |
| DSMB | Data Safety Monitoring Board |
| DSUR | Development Safety Update Report |
| DWI | diffusion weighted imaging |
| EC | Ethics Committee |
| ECASS | European Cooperative Acute Stroke Study |
| ECG | Electrocardiography |
| EKUT | Eberhard Karls Universität Tübingen |
| END | early neurological deterioration |
| ENI | early neurological improvement |
| EQ-5D-5L | EuroQoL Questionnaire-five dimensions-five levels of severity |
| ESO | European Stroke Organisation |

| | |
| --- | --- |
| $etCO_2$ | end-tidal carbon dioxide |
| EVT | Endovascular treatment |
| FD | Financial Disclosure |
| FFP2/3 | Filtering Face Piece 2/3 |
| $FiO_2$ | Inspiratory oxygen fraction |
| FLAIR | Fluid-attenuated inversion recovery |
| FSI | First Subject In |
| GCP | Good Clinical Practice |
| GCP-V | GCP-Verordnung (Good Clinical Practice Ordinance) |
| GGT | Gamma-glutamyl transpeptidase |
| HEENT | Head, eye, ear, nose and throat exam |
| IB | Investigator´s Brochure |
| ICA | Internal carotid artery |
| ICH | Intracranial hemorrhage |
| ICH GCP | International Council on Harmonization of Technical Requirements for Registration of Pharmaceuticals for Human Use harmonized tripartite guideline on GCP |
| ICMJE | International Committee of Medical Journal Editors |
| ICU | Intensive Care Unit |
| IEAB | Independent Ethical Advisory Board |
| IMP | Investigational Medicinal Product |
| INN | International Nonproprietary Name |
| IPR | Intellectual property rights |
| ISF | Investigator Site File |
| ISRCTN | International Standard Randomized Controlled Trial Number |
| IQCODE | Informant Questionnaire on Cognitive Decline in the Elderly |
| ITT | Intention To Treat |
| IVT | Intravenous Thrombolysis |
| KKS | Koordinierungszentrum für Klinische Studien (Coordination Centre for Clinical Trials) |
| LAR | Legally Authorized Representative |
| LDH | Lactate dehydrogenase |
| LKP | Leiter der Klinischen Prüfung (Coordinating Investigator according to AMG) |
| LND | Late Neurological Deterioration |
| LNI | Late Neurological Improvement |
| LSI | Last Subject In |
| LSO | Last Subject Out |
| LVO | Large vessel occlusion |
| MACE | Major Adverse Cardiovascular Events |
| MADRS | Montgomery–Åsberg Depression Rating Scale |
| MCA | middle cerebral artery |
| MedDRA | Medical Dictionary for Regulatory Activities |
| MoCA | Montreal Cognitive Assessment |
| MRI | magnetic resonance imaging |

| | |
|---|---|
| mRS | modified Rankin Scale |
| N/A | Not applicable |
| NBHO | normobaric hyperoxygenation |
| NCCT | non-contrast computed tomography |
| NIHSS | National Institutes of Health Stroke Scale |
| NT-proBNP | N-terminal prohormone of brain natriuretic peptide |
| OT | oxygen therapy |
| $PaCO_2$ | Partial pressure of carbon dioxide |
| $PaO_2$ | Partial pressure of oxygen |
| PCI | Percutaneous coronary intervention |
| PFO | Patent foramen ovale |
| PI | Principal Investigator |
| pmRS | pre-stroke modified Rankin Scale |
| POC | Point-of-Care |
| PP | per-Protocol |
| PROBE | Prospective randomized open blinded end-point |
| Q | quarter (time span) |
| RCT | Randomized Controlled Trial |
| rt-PA | recombinant tissue-plasminogen activator |
| SAB | Scientific Advisory Board |
| SAE | Serious Adverse Event |
| $SaO_2$ | Oxygen saturation |
| SARS-CoV-2 | Severe Acute Respiratory Syndrome-Corona Virus-2 |
| SC | Steering Committee |
| sICH | Symptomatic Intracranial Hemorrhage |
| SIS-16 | Stroke Impact Scale 16 |
| SmPC | Summary of Product Characteristics |
| SND | Subacute Neurological Deterioration |
| SNI | Subacute Neurological Improvement |
| sNIHSS-EMS | Shortened NIHSS for emergency medical services |
| $SpO_2$ | peripheral oxygen saturation |
| STEMI | ST elevation myocardial infarction |
| SUSAR | Suspected Unexpected Serious Adverse Reaction |
| TBY | Endovascular mechanical thrombectomy |
| TICI | Thrombolysis in Cerebral Infarction |
| TMF | Trial Master File |
| TOAST | Trial of ORG 10172 in Acute Stroke Treatment |
| TOF | time-of-flight |
| TSH | Thyroid-stimulating hormone |
| T2* | T2 star |
| UKL-HD | University Hospital Heidelberg |

| | |
|---|---|
| V | Visit |
| VEND | Very Early Neurological Deterioration |
| VENI | Very Early Neurological Improvement |
| VHIR | Fundacio Hospital Universitari Vall d´Hebron Barcelona – Institut de Recerca, Spain |
| WHO | World Health Organization |
| WP | Work package |

# 4 INTRODUCTION

## 4.1 Scientific Background

Stroke is a common, non-communicable disease, causing chronic and severe disability. During the acute phase of stroke, outcome-modifying treatment strategies may have significant impact on long-term disability. This has been proven by studies of intravenous thrombolysis (IVT) and endovascular mechanical thrombectomy (TBY) applied within 4.5 to 6 hours after stroke onset.[2, 3] However, due to strict contraindications for these therapies, only a small percentage of stroke patients are eligible for IVT and TBY, and many patients will suffer unfavorable outcomes despite treatment.[3] Albeit extensive research efforts have been undertaken, effective (additional) neuroprotection to extend treatment time windows and improve clinical outcomes is lacking.

**Epidemiology and Burden of the Disease:** Epidemiological studies suggest a global incidence of ischemic stroke of more than 11,000,000 each year.[4] Together with other cardio-vascular diseases, it remains one of the most common causes of death in high-income countries. It is estimated that stroke causes around 460,000 deaths per year in the EU, making it the second most common cause of death only after coronary heart disease.[5] Despite a marked decrease in mortality due to ischemic stroke during recent decades in European countries (from 39- 110 per 100,000 in 1990 to 23-62 per 100,000 in 2010) the burden of stroke morbidity is still significant. Stroke still accounts for around 800 life years lost to disability (DALY) per 100,000 people in Central, 1400 per 100,000 in Eastern and 300 per 100,000 in Western Europe.[4] Within the European Union cerebrovascular diseases cause around €38,000 in direct health care costs per patient, and thus created a total financial burden of €19 billion in 2009. Indirect costs caused by cerebrovascular diseases comprise productivity losses due to morbidity and mortality and are believed to amount to an additional €8 billion in 2009.[5]

**Pathophysiology of Stroke:** (adapted from Poli S. et Veltkamp R., Curr Mol Med, 2009 [6]) Ischemic stroke is a chronic condition (disability) caused by an acute occlusion of cerebral arteries leading to an interruption of blood flow and oxygen supply to brain tissue. The severity and the duration of the impairment of blood flow with the consecutive insufficient supply of oxygen to the brain tissue are primary determinants of brain damage.[7, 8] Because of the high energy demand of neurons and their limited capacity for energy storage, cellular hypoxia quickly leads to the breakdown of the oxidative mitochondrial metabolism and to anoxic cell death in the densely ischemic infarct core. In the less oligemic and initially viable peripheral ischemic zone, the so-called penumbra, various cascades can induce secondary tissue damage. Due to its already compromised blood supply, the penumbra is particularly vulnerable to additional hemodynamic and metabolic challenges. For example, peri-infarct depolarizations can induce secondary hypoxia and tissue damage in focal cerebral ischemia.[8, 9]

On the cellular level, deleterious mechanisms include the neurotoxicity of excitatory neurotransmitters, the increased production of reactive oxygen species and the activation of inflammatory and apoptotic pathways. Because of their delayed onset, some of these processes are amenable to therapy and have hence received considerable attention during the last 15 years. However, so far translational research has failed to establish neuroprotective treatment strategies in ischemic stroke and early reopening of the occluded cerebral artery by IVT with recombinant tissue-plasminogen activator (rt-PA) or catheter-based TBY are the only proven effective therapy in ischemic stroke.[10]

The obvious consequence of early recanalisation is that oxygen supply to the ischemic tissue is restored, penumbral tissue is saved from dying and thus the area of definite infarction is limited. Smaller strokes typically cause less severe or even no chronic deficits. Larger strokes lead to a high degree of long-lasting disability.

Unfortunately, the outcome-modifying therapeutic impact of IVT and TBY is limited to a narrow time window and a large percentage of stroke patients are not eligible for such treatment.

In addition to recanalising strategies like IVT with rt-PA and catheter-based endovascular TBY, neuroprotective strategies aim at increasing penumbral salvage through either prolonging survival times of neuronal tissue during ischemic conditions ("freezing the penumbra") until reperfusion – increasing the number of patients eligible for IVT and TBY and improving their long-term outcome – or attenuating secondary injury conveyed to healthy brain by post-infarction processes (e.g. inflammation).

Besides stroke unit care and rehabilitative measures (e.g. physiotherapy), no evidence exists for any effective neuro-restorative treatment that may be administered during the chronic phase of stroke.

The lack of neuroprotective agents in ischemic stroke [11] limits the efficacy of standard stroke treatment. Hence, stroke keeps causing death and major disability and poses a huge challenge to patients, caregivers and society. There is an urgent need for a "game-changer" strategy, with the development of disease-

modifier treatment with neuroprotective and/or neuro-restorative effects that can help to avoid this dramatic situation in stroke.
As ischemic cell death rendering stroke a potentially disabling and costly chronic disorder is primarily mediated by hypoxia, increasing oxygen supply to the penumbra seems THE logical approach.
Neuronal energy production depends almost exclusively on oxidative phosphorylation in the mitochondria.[7] As the critical oxygen tension required for mitochondrial function is very low (1.5 mmHg) [12-14], improving the oxygen delivery to ischemic-hypoxic tissue appears to be a simple and plausible therapeutic concept to shift the ischemic threshold for cell death.
Consequently, the proposed PROOF project will investigate potential neuroprotective properties of normobaric hyperoxygenation (NBHO) (i.e. inspiration of near 100% $FiO_2$) in the setting of ischemic stroke.

**Pathophysiological Role of Oxygen in Cerebral Ischemia:** (adapted from Poli S. et Veltkamp R., Curr Mol Med, 2009 [6]) Understanding the regional variations of oxygen supply and demand and their underlying mechanisms is crucial with respect to the effects of NBHO in both the healthy and especially the ischemic brain.[15, 16] Although it represents only 2% of the body's weight, the brain is supplied with about 15% of the cardiac output and consumes roughly 20% of the oxygen used by the whole body.[14, 17] This continuous demand of blood flow and oxygen supply is reflected by a high vulnerability to ischemia.
In fact, brain tissue oxygen is depleted within seconds after complete interruption of blood flow.[16, 18, 19] On average, in man, global cerebral blood flow (CBF) is 40–60 mL/100 g/min and global cerebral metabolic rate for oxygen ($CMRO_2$) is 3–4 mL $O_2$/100 g/min.[17, 20] In thiopental-induced inhibition of synaptic transmission in non-human primates, about half of the oxygen consumed by the brain is linked to synaptic activity.[21] Fundamental neuronal and glial functions including intracellular enzyme reactions, biosynthesis of proteins, and the maintenance of the trans-membrane ionic equilibrium (e.g. ATP dependent Na+-K+ pump) account for the other 50% of global $CMRO_2$.[15, 21, 22]
In the healthy organism, the level of arterial blood oxygenation expressed as the oxygen partial pressure ($PaO_2$) is linearly related to that of the brain tissue (cerebral $PtO_2$) and may thus serve as a surrogate biomarker for brain oxygenation. Under normoxic atmospheric conditions, the $PaO_2$ is around 100 mmHg and the cerebrovenous oxygen level ($PcvO_2$) is 35 to 40 mmHg.[17] Cerebral $PtO_2$ varies between 90 mmHg and much less than 35 mmHg mainly depending on the distance of the measuring probe from the capillary.[14] Furthermore, physiologic $PtO_2$ differs significantly between brain regions ranging from near 0 mmHg to arterial values as measured by surface and inserted electrodes.[19, 23-26] This heterogeneity correlates with the local capillary density and regional CBF which themselves correlate with neuronal density and ultimately with cellular metabolism.[27-29]
Whereas the oxygen extraction fraction (OEF) in the healthy brain is approximately 40% for both grey and white matter [36], CBF as well as $CMRO_2$ is two to four times higher in grey matter than in white matter (≈60 versus 30 mL/100 g/min and 4 versus 1 mL/100 g/min, respectively) reflecting their different metabolic needs.[30, 31] Hence, the decline of $PtO_2$ after complete circulatory arrest is considerably slower in the white matter than in the rapidly metabolizing and oxygen consuming grey matter with its high capillary and cellular density.[19]
Different thresholds of cerebral oligemia have been defined over the past decades.[8, 32, 33] Complete energy failure with breakdown of the cellular ion equilibrium, anoxic depolarization and consecutive necrotic infarction manifest with CBF values of less than 12 mL/100 g/min in the ischemic core of focal cerebral ischemia.[34] However already the decline of cortical CBF just below normal levels is associated with alterations of gene expression and partial inhibition of protein synthesis.[35] Mean CBF values below 23 mL/100 g/min provoked EEG slowing, and EEG flattening occurred if CBF fell below 15 ml/100 g/min.[36, 37] Excitatory neurotransmitters are released at a residual CBF of about 18 mL/100 g/min.[38] At this blood flow threshold oxygen supply becomes insufficient resulting in depletion of high energy phosphates such as phosphocreatine and ATP, accumulation of lactic acid with decreased brain tissue pH, and membrane depolarization.[8] In addition, reduced heat conduction in the malperfused penumbra can induce local hyperthermia (2-3 °C) triggering a vicious circle with further progression of ischemia / hypoxia.[39, 40]
When breathing room air (21% oxygen) at one atmosphere absolute (1 ATA), about 97% of the oxygen transported in the blood is chemically bound to hemoglobin (Hb) and only 3% is physically dissolved in the blood plasma.[14] Considering an average hemoglobin concentration of 14 g/100 mL blood, the oxygen binding capacity of hemoglobin of 1.39 mL $O_2$/ g Hb at 37°C, and a physiological arterial Hb saturation of 97% ($SaO_2$), the total volume of oxygen bound to hemoglobin for every 100 mL of blood is about 18.9 mL. Under these circumstances, only 0.3 mL $O_2$ are physically dissolved per 100 mL of blood.[14] Consequently, only 2-3% of the cerebral metabolic rate for oxygen ($CMRO_2$) are covered by the dissolved oxygen fraction under normoxic conditions.[41]

Diffusion of oxygen along the pressure gradient is the driving force behind oxygen transport from the air in the alveoli to the blood and from there to the tissue and the mitochondria.[14, 42] When breathing 100% instead of 21% oxygen at 1 ATA (NBHO) or 100% oxygen at even higher ambient pressure (HBO), not only arterial oxygen pressure ($PaO_2$) but also brain tissue oxygen levels ($PtO_2$) increase.[43-48] For a pressure of up to 6 ATA and a constant CBF a linear relationship (6:1) was shown between the inspired oxygen pressure ($PiO_2$) and cerebral $PtO_2$.[49]
As hemoglobin is already nearly saturated ($SaO_2$ ~97%) under normoxic atmospheric conditions (21% $O_2$ at 1 ATA), normobaric or hyperbaric oxygenation can hardly increase the amount of oxygen bound to hemoglobin. Instead, the proportion of physically dissolved oxygen becomes more relevant. Given that the Bunsen solubility coefficient of oxygen in blood (37°C) is 0.003 mL $O_2$/ mmHg/ mL blood, increasing $PaO_2$ from 100 mmHg to 663 mmHg (100% $O_2$ at 1 ATA) raises the physically dissolved oxygen fraction from 0.3 mL to about 2 mL per 100 mL of blood.
Based on these physiological principles, it has been hypothesized for decades that the surplus of physically dissolved oxygen by NBHO may provide sufficient oxygen to hypoxic-ischemic brain areas. Increasing the dissolved oxygen plasma fraction by oxygen therapy may be particularly relevant in focal cerebral ischemia [50], because capillary plasma flow was maintained in spite of a substantial reduction of cortical CBF.[51]

**Evidence from Preclinical Trials:** NBHO as a disease modifying therapeutic strategy was evaluated in a number of experimental models of transient and permanent middle cerebral artery filament occlusion (tMCAO and pMCAO) as well as thromboembolic stroke models.
In summary, treatment with NBHO in animal models of transient MCAO of up to three hours and thromboembolic models with recanalisation through IVT (see [6, 52, 53] for review):

- Significantly raised penumbral $PtO_2$ [54, 55], increased the cerebral oxyhemoglobin concentration, CBF [4][56] as well as cerebral blood volume [9], reduced peri-infarct depolarization in ischemic core and penumbra [56, 57], led to a restitution of penumbral purine nucleotide levels (ATP/ADP and GTP/GDP ratios) [58, 59], reduced ATP depletion [60] as well as lactate concentrations and preserved N-acetyl-aspartate (NAA) levels within regions of ischemia, suggesting improvement of oxidative metabolism [61],
- reduced markers of apoptotic cell death (e.g. ISEL-positivity) in hypoperfused tissue [62] and improved histological (selective neuronal loss, blood brain barrier function, hemorrhagic transformation, etc.) [52, 62-65], neuroimaging [62, 66] as well as functional outcomes [52, 67],
- did not augment markers of oxidative stress after ischemia(-reperfusion) like heme oxygenase-1, protein carbonyl, hydroethidine or 8-hydroxy-20-deoxyguanine [54, 63, 68, 69] (see [70] for review). Other indirect markers of oxidative stress such as matrix metalloproteinase (MMP)-2, MMP-9 and caspase-8, were either unchanged or even decreased after NBO in experimental ischemia [54, 68],
- did not lead to post-ischemic hemorrhagic transformation after IVT in thromboembolic stroke models [64, 65],
- extended the time window for successful reperfusion from one hour (room air) to 3 hours (NBHO) [68] by “freezing the penumbra” demonstrated by sustained PWI/DWI mismatch on MR imaging [62] and most importantly
- led to significant infarct core volume reductions, ranging from 35 to 50%.[56, 62, 63, 67, 68, 71, 72]

NBHO treatment in prolonged tMCAO (≥ 4 hours) did not show consistently significant effects on infarct core volume reduction.[64, 68] Studies investigating the effects of NBHO on infarct core volume in permanent MCAO, either found no significant effect [66, 68] and one a marginal though significant effect when NBHO treatment was extended from three to six hours.[62]

**Evidence from Clinical Trials:** In clinical trials, low-flow oxygen supplementation and ventilation with an $FiO_2$ of 0.4, which are both not to be confused with NBHO, did not improve outcomes in ischemic stroke patients.[73-75]
Trials evaluating NBHO did not adapt insights gained in preclinical studies as initiation of oxygen therapy was delayed and patients eligible for IVT and TBY were excluded [76-78], which made penumbral rescue through early initiation of NBHO and rapid and effective recanalisation unlikely. Furthermore, Padma et al. chose an oxygen flow-rate of 10L/min, which is too low to achieve sufficient hyperoxygenation.
Singhal and colleagues enrolled 16 ischemic stroke patients with proximal vessel occlusions (terminal ICA or proximal MCA) who were ineligible for IVT and in whom TBY was not attempted into a randomized pilot trial.[76] Nine of those patients were treated with NBHO (i.e. ≥ 40 L/min of $O_2$ via a simple face-mask), which was initiated between 2 and 14 hours after symptom onset and administered for a maximum of 8 hours. Arterial blood gases were drawn for clinical reasons in 3 patients: the $PaO_2$ was 368 and 420 mmHg in two NBHO patients (indicating hyperoxygenation) and 99 mmHg in the control patient (representing a physiological value under respiration of room air or low-flow $O_2$ (1-3 L/min) to maintain $SpO_2$ ≥ 95%).

In the treatment group of this trial, NBHO led to:

- a transient improvement of clinical status (NIHSS) which was noted as early as 15-20 minutes after initiation of NBHO but only until 24 hours after stroke and not beyond this time point.
- a decrease in infarct core volume (as determined by DWI-MRI) and an increase in penumbral salvage (as determined by DWI/PWI-mismatch) during treatment (imaging at t = 4 hours after treatment initiation), and
- a reduction of lactate concentration and a preservation of NAA in the ischemic brain tissue suggesting NBHO-mediated improvement of aerobic metabolism and restored mitochondrial function respectively.[61]

Based on these encouraging results, Singhal and colleagues initiated a phase II trial, which was prematurely terminated after enrolment of 85 (out of planned 240) patients (data unpublished). Despite being stopped over safety concerns (excess mortality in the NBHO group) by the DSMB, it was later concluded by a blinded external medical monitor that observed fatalities in the treatment group were not related to NBHO but due to withdrawal of care in patients with massive infarcts at baseline or moribund admission status. In other cases, death was adjudicated to pre-existing conditions, such as cancer.
Analysis of prespecified endpoints showed that, NBHO did

- not impair clinical outcomes as determined by NIHSS, mRS and Barthel Index
- not worsen imaging-based parameters such as ischemic core growth, recanalisation/reperfusion rates
- not show significant differences regarding safety endpoints such as brain hemorrhage, brain edema or systemic (including respiratory) adverse events

A number of experts regretted the premature termination of that study and called for the conduction of new clinical NBHO trials.[77]

## 4.2 Trial Rationale/ Justification

Our interpretation of the available preclinical and clinical data is that NBHO exerts neuroprotective effects on ischemic but still viable brain tissue. However, a proof-of-concept for this assumption is still lacking. We believe that previous clinical studies have failed to show strong and long-lasting effects of NBHO in stroke patients because evidence from successful preclinical application of NBHO were ignored in the design of these trials. In order to successfully translate results of positive experimental NBHO studies into clinical practice, their relevant features must be adapted: (1) early initiation of NBHO in (2) transient focal ischemia (early recanalisation therapy).

The goal of PROOF is to develop a novel neuroprotective strategy aimed at penumbral salvage in patients with acute occlusion of proximal cerebral arteries and identifiable ischemic tissue at risk.
This setting may serve as a model for ischemic stroke in general, which – as a disease causing major chronic disability – poses a huge burden for individuals, society and healthcare systems.
PROOF may thus lay the foundation for improving care for an even larger proportion of ischemic stroke patients (pre-hospital treatment initiation in an unselected stroke population, i.e. pre-imaging), which would have a significant impact on individuals and healthcare system in the European Union and beyond.
A multitude of preclinical studies on neuroprotectives have shown positive effects in animal models. However, translating these treatment strategies into clinical practice has failed so far.[11]
Even though experimental data suggest feasibility of neuroprotection, improving clinical outcomes after ischemic stroke by adapting preclinical evidence and thus preventing stroke mortality and major chronic disability has been and stays a huge challenge.
For our trial, we assume an effect of NBHO based on the observations made in experimental and clinical studies: NBHO "freezes" the penumbra but reperfusion of ischemic tissue must occur in order to achieve lasting neuroprotection as "nothing can hold its breath forever".
From a pragmatic point of view, NBHO is cheap, easy to apply and can be integrated into standard diagnostic and therapeutic work-up without interfering with established and effective treatments.
At present, no neuroprotective treatments are available. If our academic phase IIb study in a highly-selected population demonstrates a disease-modifying effect, NBHO as a therapeutic strategy could potentially be offered to the population of patients with stroke as a whole (e.g. in the context of a phase III trial). Considering its low costs and ease of use, NBHO may thus impact stroke care worldwide.

## 4.3 Risk-benefit Assessment

In the following, potential adverse effects that may be caused by oxygen treatment but are either mild and fully reversible or not expected to occur in the PROOF-trial are discussed. They are summarized in Table 1. Overall, the inhalation of 100% oxygen at normal atmospheric pressure (1 ATA ≈100kPa ≈760mmHg ≈1.000mbar) over a maximum of four hours – as planned in the PROOF trial – should not have any clinically relevant negative impact on our study patients.

Normobaric hyperoxygenation has been extensively studied in a wide variety of stroke and non-stroke conditions over the course of the last century (compare Section 8.1.1 General Information). In summary, clinically relevant adverse effects unrelated to the underlying condition are only registered beyond six hours of exposure to NBHO; they are summarized in the Medical Oxygen Summary of Product Characteristics.

In the PROOF trial, NBHO will be applied over a maximum of four hours. Respiration of 100% oxygen leads to asymptomatic decrease of muco-ciliary clearance of the airways after six to eight hours of exposure. Symptoms of an irritation of the tracheal and bronchial mucous membranes which may be associated with a feeling of tightness in the chest may be observed after twelve hours of NBHO. All changes and symptoms present at this stage are fully reversible and will resolve after discontinuation of treatment.
More severe side effects, like the Lorrain Smith effect (i.e. pulmonary toxicity including resorption atelectasis and pulmonary inflammation leading to build-up of interstitial and alveolar fluid and thus to a decrease of the total area of capillary gas exchange with clinically apparent respiratory failure) only occur beyond 17 hours of NBHO.
Excessive oxygen supplementation in patients with reduced chemoreceptor-sensitivity (e.g. patients with chronic obstructive pulmonary disease) may lead to reduced respiratory drive and subsequently to hypercapnia, acidosis and – in extreme cases – to hypercapnic coma, which is why patients with a history of chronic lung disease or even mild but acute respiratory distress are excluded from study participation (compare Section 7.4 Inclusion Criteria and 7.5 Exclusion Criteria).
The application of a face-mask may cause mechanical irritation to the skin of the patient. The clinical impact of this, however, is mild as irritations will resolve with removal of the face-mask. Claustrophobia may be caused by the application of a face-mask during NBHO treatment. Symptoms of skin irritation and claustrophobia may warrant the discontinuation of study treatment. This decision must be left to the discretion of the attending site investigator.
An increase of systemic $pO_2$ may in theory subject the brain to increased oxidative stress, which may lead to the build-up of reactive oxygen species and subsequently promote direct tissue damage. However, experimental data from animal models of NBHO (and hyperbaric hyperoxygenation) suggests the exact opposite: NBHO did not augment formation of reactive oxygen or nitrogen species or markers of oxidative stress after ischemia-reperfusion (such as hemeoxygenase-1, protein carbonyl, hydroethidine or 8-hydroxy-20-deoxyguanine). Other indirect markers of oxidative stress such as matrix metalloproteinase 2 and 9 and caspase-8, were either unchanged or even decreased through NBHO.[70]
NBHO is known to cause vasoconstriction of intracranial brain-supplying arteries in the healthy brain.[79] This effect may potentially aggravate ischemic-hypoxic conditions in already hypoperfused areas of the brain during acute ischemic stroke. Experimental as well as clinical data indicate that NBHO may well lead to vasoconstriction in healthy areas of the brain but also that physiological vessel reagibility to $pO_2$- and $pCO_2$-changes is lost in ischemic brain tissue.[80, 81] In fact, in 1968, Lassen and Pálvölgyi observed an *inverse* steal effect in stroke patients during hypocapnia (i.e. a shift of cerebral blood flow towards ischemic areas) and called it the “Robin Hood effect” (“take from the rich, give to the poor”).[82] This might also be true for NBHO, as there is preclinical evidence for an increase in penumbral blood flow during hyperoxia.[56, 76] Imaging data from Singhal’s NBHO-pilot study showing an increase in penumbral blood flow and volume as well as reversibility of DWI-lesions during NBHO strongly supports the existence of this beneficial oxygen effect also in humans with ischemic stroke.[76]
In the same study, asymptomatic petechial hemorrhage was noted on follow-up MR-imaging at 24 hours after stroke in four of nine (44%) patients treated with NBHO and in one of seven (14%) patients in the control group; no case of symptomatic intracranial hemorrhage was reported in the NBHO-group. The finding was not statistically significant but raised safety concerns.
The occurrence of these microbleeds was later attributed to spontaneous recanalisation of the large vessel occlusions causing the stroke in these patients and thus confounded. In clinical imaging studies evaluating occurrence of hemorrhagic changes due to reperfusion treatment (intravenous thrombolysis or TBY), the incidence of new microbleeds was associated with successful vessel recanalization and either not or positively with neurological outcome; leading to the conclusion that microbleed occurrence is simply an indicator of early reperfusion and not in itself clinically relevant.[83, 84] Results from experimental studies

led to the conclusion that combination of NBHO and recanalising therapies, such as intravenous thrombolysis, may actually mediate an additive beneficial effect on stroke outcomes.[85, 86]
A phase II-NBHO RCT evaluating 8 hours of high-flow oxygen versus medical air delivered at 30-45 L/min via a simple face-mask, starting within 9 hours of stroke onset, was initiated in 2007 [NCT00414726]. This trial aimed to enroll 240 patients but was stopped by the safety committee after only 85 patients had been enrolled (43 NBHO, 42 Air) due to a higher mortality rate in the NBHO arm. Later blinded re-evaluation did not indicate any link between mortality and NBHO and attributed the imbalance in deaths to early withdrawal of care due to massive infarcts or moribund admission status. Analysis of several pre-specified safety endpoints did not show significant differences between groups. While the primary efficacy outcomes did not differ between the NBHO and standard treatment arm, a pre-specified voxel-based MRI analysis showed that the apparent diffusion coefficient (ADC) values within ischemic regions improved during NBHO, suggesting benefit. A number of experts have criticized the premature termination and called for a repeated trial.[77]

Other trials often cited in the context of NBHO including an RCT conducted in Taiwan (N=46), which found mild hyperoxygenation ($FiO_2$ of 0.4) to be associated with lower mortality and fewer complications in patients with large MCA infarctions [75] and two recent retrospective studies (N=2643 and N=554), which found no influence of hyperoxia on mortality and length of hospital stay in ventilated AIS patients [87, 88], support a positive safety profile of hyperoxygenation.
Two further prospective trials (N=550 and N=301), did not actually focus on hyperoxia as an acute treatment but aimed to prevent post-stroke hypoxia and maintain normal oxygen saturation. These trials compared low-flow (2-3 L/min) oxygen supplementation to room air delivered from 24 to 72 hours after ischemic or hemorrhagic stroke and provided inconclusive results.[74, 89] The recent confirmatory phase III Stroke Oxygen Study (N=8003) with the same objective showed no benefit of – but also no safety issues with – low-flow supplemental oxygen.[90]

Stroke and <u>acute myocardial infarction</u> (AMI): distinct organ, but same pathophysiology (arterial occlusion and ischemic necrosis) and (catheter-based reperfusion) therapy, i.e. TBY and percutaneous coronary intervention (PCI).[91] Additionally, patients suffering stroke or AMI exhibit similar cardiovascular risk factors and co-morbidities.
In 2016, a Cochrane systematic review meta-analyzed five RCTs that compared non-hypoxic patients with suspected or proven AMI who were treated with oxygen (at 4 to 8 L/min until end of PCI or over up to 24 hours) to AMI-patients who inhaled ambient-air and received supplemental oxygen only during hypoxic episodes; the five RCTs involved a total of 1173 participants, 32 of whom died.[92] Similar death rates were found in both groups, suggesting oxygen neither helps nor harms. Nevertheless, the authors emphasized that the included RCTs were not big enough and recommended to test oxygen in a large-scale trial to definitively answer the question whether oxygen therapy is beneficial or harmful in AMI. Since then, the SOCCER phase II trial (N=94; 10 L/min until end of PCI) and – most recently – the big DETO2X-AMI phase III trial (N=6629; 6 L/min over 6 to 12 hours, median duration: 11.6 hours) were completed and published.[93-95] Both RCTs found no beneficial effects of oxygen therapy, but, on the other hand clearly confirmed its safety in AMI-patients. In the DETO2X-AMI trial, the 1-year mortality (5% oxygen vs. 5.1% air), 1-year re-AMI rate (3.8 vs. 3.3%) as well as peak troponin levels during hospitalization (947 vs. 983 ng/L) were similar in both study groups; these results were consistent across all predefined subgroups.

The following adverse effects associated with hyperoxygenation are not expected to occur in the PROOF trial: direct nervous system toxicity (i.e. the Paul Bert effect associated with disturbance of hearing and vision, vertigo, nausea/vomiting agitation, confusion, epileptic seizures) as well as barotrauma of the lung and the middle ear only occur during hyperbaric conditions. Retrolental fibroplasia (i.e. Terry's syndrome associated with severe visual impairment or blindness) is only described in (premature) neonates.

**Additional study-dependent risks**
For reasons of safety, assessment of pharmacodynamics and -kinetics as well as biomarker-analysis, patients enrolled in the PROOF trial will undergo sampling and analysis of venous and arterial blood. Depending on time-point and use, withdrawal of blood is either part of clinical routine or study-dependent.
Preferably, all scheduled blood samples (study-dependent or -independent) will be taken from routinely inserted indwelling venous or arterial catheters.
All patients will have study-independent venous access at Screening and until at least V5, if not even V6. Study-dependent blood sampling through venipuncture will therefore only be necessary in few cases at V6. Due to the TBY procedure, most patients will have arterial-access at V3, maybe even at V5.

Study-dependent venous blood samples:
If at V6 safety laboratory assessments are not regarded as clinical routine and the patient does not have a routinely inserted indwelling venous catheter, blood for study-dependent laboratory assessments is drawn through venipuncture. Blood samples for study-dependent safety laboratory assessments at V6 are only drawn if the patient or the respective LAR gives written informed consent to participation in the PROOF trial. Very minimal risks may be associated with venipuncture: A common complication of venipuncture is pain. This is usually mild, but in very rare cases permanent nerve injury can result. Occasionally, a patient will bleed longer/more than normal after a venipuncture. This is more common in patients treated with anticoagulants. Some patients will develop a subcutaneous hematoma. Inflammation or infection of the tissue below the skin surface (cellulitis) or the vein (phlebitis) is a rare complication of venipuncture, but the risk is given whenever the skin is broken. Sepsis – a serious blood infection – is also possible following venipuncture. Proper disinfection of the site prior to pricking the skin greatly reduces the risk of infection-related complications. Some patients experience severe anxiety when faced with being stuck with a needle. As consequence, they may experience a vasovagal reaction such as sweating, numbness and tingling of hands and feet, nausea, vomiting, low blood pressure, syncope. Generally, the adverse effects described above do not lead to any relevant long-term impairment because they are either transient or easily treated (e.g. with antibiotics).

Blood sampling for biomarker-analysis at Screening and V5 is drawn from a study-independent venous or arterial access only, i.e. not associated with any risk.

Study-dependent arterial blood samples:
Arterial blood gases are drawn from a study-independent arterial access only. This means that an arterial blood gas analysis is performed at V3 as a part of clinical routine nearly only in patients undergoing TBY. If an arterial access is still available, an additional study-dependent blood gas analysis is performed at V5. No arterial access is established as a study procedure.

The total amount of blood drawn solely for study purposes amounts to 54.5 mL in case the patient consents to participation in the biomarker sub-study or to 36.5 mL if participation in the biomarker sub-study is refused prior to V5 or 18.5 mL if participation in the biomarker sub-study is refused at screening (see Sections 10.18 Laboratory assessment, 10.19 Biomarkers (Pharmacodynamics), and 10.20 Arterial blood gases (Pharmacokinetics) as well as Table 14 for details).
Adverse effects due to the removal of this small amount of blood are not to be expected.

**Table 1:** Potential adverse events of oxygen treatment

| Adverse effect | Symptoms | Clinical impact | Expected in PROOF-trial? | Expected frequency in PROOF-trial | Comment |
|---|---|---|---|---|---|
| decreased muco-ciliary clearance | none | none | unlikely | unknown | occurs only > 6 hours of NBHO |
| upper/lower airway irritation | coughing, dyspnea, chest tightness | low | unlikely | less than occasionally | occurs occasionally (≥1/1.000 but < 1/100) in patients exposed to NHBO for > 12 hours |
| pulmonary inflammation, edema, atelectasis, pleuritis (Lorrain Smith effect) [96, 97] | pain, coughing, dyspnea, respiratory failure | high | no | Ø | occurs only after NBHO > 17 hours |
| decreased respiratory drive [97] | respiratory failure, drowsiness, acidosis, hypercapnic coma | moderate to high | unlikely | rare<br>(≥ 1/10.000 but<br>< 1:1.000) | occurs only in patients with a history of pulmonary/neuromuscular disease (excluded from PROOF-trial (see Section 7.5).<br>early signs are detected by continuous monitoring |
| mechanical irritation through face-mask | skin irritation | low | yes | unknown | may warrant NBHO discontinuation |
| claustrophobia | agitation, hyperventilation, heart rate and blood pressure disturbances | low to moderate | yes | unknown | may warrant NBHO discontinuation |
| direct central nervous system toxicity (Paul Bert effect) [98] | disturbance of hearing and vision, vertigo, nausea/vomiting agitation, confusion, epileptic seizures | moderate to high | no | Ø | occurs only under hyperbaric conditions ($pO_2$ ≥ 1.4 ATA ≈ 1000 mmHg ≈ 140 hPa ≈ 1,400 mbar) |
| barotrauma of middle ear and/or lungs[97] | pain, hearing loss, dyspnea, respiratory failure, gas embolism | moderate to high | no | Ø | occurs only under hyperbaric conditions |
| retrolental fibroplasia (Terry syndrome) [97, 99, 100] | visual impairment, blindness | moderate to high | no | Ø | occurs only in neonates |

**Prevention, early detection and management of adverse events in the PROOF-trial**

Prevention: In the PROOF-trial, NBHO treatment is limited to a maximum of four hours (which is the longest planned exposure to 100% oxygen inhalation). This time frame was chosen in order to maintain the highest possible safety with regard to potential oxygen toxicity. Thus, only mild and fully reversible adverse effects of oxygen administration are to be expected in the PROOF trial.
Patients with a history of COPD and other pulmonary disorders are not to be enrolled in this trial (see Section 7.5 Exclusion Criteria). Use of concomitant medications which may increase the risk of adverse events are prohibited (see Section 8.4.5 Prior and Concomitant Medication). To avoid harm for unborn children, pregnancy must be excluded in all women ≤55 years (except if surgically sterile) and in women >55 years in case of increased probability for pregnancy (e.g. due to in-vitro fertilization). Breastfeeding women can participate, but must be instructed to stop breastfeeding after randomization.

Early detection: All trial patients will receive care on specialized stroke units or intensive care units. Personnel on these units, where patients will be treated during application of NBHO and continuously monitored for changes in neurological status and vital parameters, are experienced in dealing with complications during treatment of critically in patients.
In the unlikely case of acute respiratory failure due to NBHO, early changes of oxygen saturation, decrease in respiratory rate (and hypercapnia) will be observable on continuous and non-continuous monitoring. Should respiratory failure occur due to NBHO during the treatment period, NBHO will be discontinued to avoid clinical impact (see Section 7.7.2 Discontinuation of Trial Treatment).
In case of negative effects of NBHO on neurological outcome, we will be able to detect these through frequent safety assessments during the trial (provided to the DSMB and the sponsor every 6 months). Additionally, every death, parenchymal hematoma and remote intracranial bleeding (see Appendix 14 for Heidelberg bleeding classification [101]) will be reported expedited to the DSMB. Imaging and clinical outcome data will be reviewed by the DSMB. In case of an excess of unfavorable outcomes (e.g. increased mortality, bleeding risk, high rates of SAE, etc.) the trial will be suspended for in-depth DSMB review in order to prevent harm from further trial participants. If it is determined that the excess in safety outcomes is related to NBHO, the trial will be discontinued.

Management: For patients who do not tolerate NBHO, a stepwise decrease of oxygen flow-rate or change of mask-type is recommended in Section 7.7.2 Discontinuation of Trial Treatment. In participants who experience clinically relevant respiratory distress or failure, the investigator will immediately determine the cause and reduce oxygen flow-rate, change the type of face-mask or discontinue study treatment (see Section 7.7.2 Discontinuation of Trial Treatment). Participants who experience respiratory failure may have to undergo non-invasive or invasive mechanical ventilation. The participants' vital function parameters will be monitored continuously until resolution of the event. If possible, all participants will undergo imaging and clinical follow up as scheduled in the study protocol.

**Potential benefits of the PROOF-trial**
Potential benefits of NBHO have been discussed in length in the introduction to this study protocol. They may be summarized as follows. On the patient level: With promising preclinical and clinical data of NBHO treatment in ischemic stroke, half of the patients enrolled in the PROOF trial will receive a therapy with potential additional benefit for their clinical outcome. On the population level: Prospectively, given positive trial results, the PROOF-trial may impact stroke treatment guidelines thus improve stroke care worldwide.

Patients randomized into the PROOF trial will either receive NBHO adjunct to standard therapy or standard therapy alone. Thus, PROOF-patients will not be disadvantaged with regard to established standard diagnostics and treatment.

**Overall risk/benefit analysis**
Overall, with a maximum treatment duration of up to four hours, we do not expect to see any severe adverse effects of NBHO. Less severe and fully reversible adverse effects of NBHO may be seen over the course of the trial. These are unlikely to have a negative impact on neurological outcome.
In summary, the controlled risks described above are by far outweighed by the prospect of identifying a potentially highly effective neuroprotective strategy in ischemic stroke.

#### *4.3.1* Risk-Benefit Assessment Considering the Literature until December 6th, 2019

This chapter will discuss any ambiguity regarding the risk-benefit assessment and consent modalities of the PROOF study trial considering recently published review articles including one meta-analysis as well as the main results of the **New Zealand Oxygen in Acute Coronary Syndromes Trial (NZOTACS)** that had been first presented at the European Society of Cardiology congress in Paris, France, in 2019 [102].
For the risk-benefit evaluation it is important to know – especially because patients that are unable to consent are to be included in the PROOF trial – that despite modern day reperfusion therapy with endovascular mechanical thrombectomy ± intravenous thrombolysis, 54% of patients are still left with long term disabilities and remain functionally dependent or even die (mRS ≥ 3) (e.g. [103]). Thus, there is an urgent need for new and adjuvant therapy for the PROOF trial population; a group of patients with the most severe strokes. These patients unfortunately lose a lot and therefore have a lot to gain from such developments.
All representatives of the PROOF study group are convinced that NBHO can only reduce brain damage in acute ischemic strokes if it is conducted as set out in the PROOF trial protocol and that we do not only expect a group effect but, in fact, a positive influence on the course of every patient's condition who is treated with NBHO. Moreover, with the help of our own meta-analysis of the original papers relevant for the potential PROOF study population (see Appendix 1), we can ascertain that using a high dose but – in the interest of patient security and maximum efficacy – a short duration, the oxygen therapy planned for the PROOF trial poses no significant risk to the patient suffering an acute ischemic stroke due to a proximal vessel occlusion.

**Requirements for successful oxygen treatment in animal experiments**
During animal testing, NBHO was proven to be very effective and reduced the volume of cerebral infarction by up to 50% in comparison to non-hyperoxygenated animals; however, only if three framework conditions were fulfilled. NBHO cannot stabilize and keep alive indefinitely the collateralized and critically malperfused and thus hypoxic margins of the area undergoing infarction, which is bound to perish (the so-called penumbra). NBHO was only consistently effective in animal experiments when the ischemia was transient and lasted a maximum of three hours, not so in delayed or missing vessel recanalization and brain tissue reperfusion (see Fig. 1 and compare final infarct versus failed recanalization in Fig. 3) [52].

**Permanent MCA-Occlusion** **Transient (180min) MCA-Occlusion**

Due to copright restrictions this figure is not included.
Please refer to figure 3 in Henniger et al. 2007 [62]

**Figure 1: Spatiotemporal evolution of threshold-derived apparent diffusion coefficient (ADC) and cerebral blood flow (CBF) lesion volumes in an experimental model of ischaemic stroke treated with 100% normobaric oxygen (NBO)**. Animals in this experiment were subjected to (A) permanent middle cerebral artery occlusion (pMCAO) or (B) transient MCAO (tMCAO) and received either NBO or room air (control condition). Asterisks denote significant between-group difference in ADC- and CBF-derived lesion volumes ($P < 0.05$). Note that the statistically significant positive effect of NBO is only translated onto a later time-point (in this case definitive infarct volume as determined by 24h triphenyl tetrazolium chloride (TTC)-staining)) if MCAO is transient and not permanent. (from [62])

**A: Experimental Protocol**

Due to copyright restrictions this figure is not included.
Please refer to figure 1 in Singhal et al. 2002 [104]

**B: Infarct Sizes**

Due to copyright restrictions this figure is not included.
Please refer to figure 6 in Singhal et al. 2002 [104]

**Figure 2: Effect of treatment delay of normobaric hyperoxygenation in a rat tMCAO model. 4A:** Schematic representation of the experiment protocol. All animals were anesthetized with 1 % halothane. White bars represent a breathing mixture containing 70 % nitrous oxide and 30 % oxygen. Black bars represent a breathing mixture containing 100 % oxygen. **4B:** Total lesion volumes at 48 hours after MCAO. (*p < 0.05 vs. normoxia group). TTC: 2 % 2,3,5-triphenyltetrazolium chloride. (from [104])

Furthermore, NBHO in animal experiments was only effective when it was initiated early on after the start of ischemia (see Fig. 2 and compare rows 3 and 4 in Fig. 3) [66] and almost pure oxygen was breathed in normal atmospheric pressure environment. An inspiratory oxygen fraction of 70% ($FiO_2$ 0.7) did not suffice, the ischemic penumbra was only sufficiently oxygenated at an $FiO_2 \geq 0.95$ (see Table 2) [54].

**Table 2: Blood gas and penumbral $pO_2$ measurement**

Due to copyright restrictions this table is not included.
Please refer to table 1 in Liu et al. 2006 [54]

(from [54])

The ischemic penumbra can thus be stabilized by breathing almost pure oxygen early on ('freezing the penumbra'), but, in animal models, could only survive if the blocked vessel was recanalized within three hours ('nothing can hold its breath forever') (see Fig. 3) [105]. In humans, however, significant penumbral salvage may also possible beyond the three-hour time window as shown in the two recent TBY studies DAWN [106] and DEFUSE-3 [107].

Due to copyright restrictions this figure is not included.
Please refer to Box 1 in Baron 2018 [54]

**Figure 3: The penumbral freezing paradigm.** The figure depicts an idealized time course of the ischaemic penumbra and core in various scenarios after stroke. In the real world, accurate mapping of the penumbra and core is challenging.

**The top row shows the typical natural history** of tissue evolution after acute middle cerebral artery (MCA) occlusion. By 3 hours after stroke onset, the centre of the MCA territory, where perfusion is lowest, is irreversibly damaged. This ‘core’ is surrounded by a rim of ‘penumbra’, where perfusion is better preserved owing to leptomeningeal collaterals, which are fed by the neighbouring anterior and posterior cerebral arteries. Without recanalization, more penumbra progresses to core tissue. eventually, the at-risk penumbra turns into core, and no salvageable tissue remains. The final infarct, which comprises the initial core and penumbra, is large, causing severe disability (modified Rankin Scale (mRS) score 5). The final infarct size is not the only determinant of final outcome: infarct topography, age, comorbidities, previous stroke and white matter ischaemic changes are also important factors. **The second row illustrates the benefits of recanalization** achieved at 4.5 hours, which stops core growth, resulting in a smaller final infarct and an mRS score of 3 — much better than with no reperfusion but still above the threshold for functional independence (mRS score 2). If recanalization fails, the outcome is the same as in the natural history scenario. **The third row shows the effects of freezing therapy by normobaric hyperoxygenation initiated on hospital admission**, which enables core growth to stop until recanalization is achieved 1.5 hours later. owing to extensive penumbral salvage, the final infarct is the same size as the core on admission, and the patient achieves functional independence (mRS score 2). Again, if thrombectomy fails, the final outcome is the same as in the natural history scenario. **The fourth row illustrates the theoretical effects of efficacious freezing therapy started prehospitally** 1.5 hours after stroke onset. This strategy allows maximal penumbral salvage and minimal final infarct, resulting in mild residual symptoms but no functional disability (mRS score 1).

**Nonetheless, in the PROOF phase II proof–of–concept study we decided against the in-the-field approach for several reasons and will leave this open for a follow-up phase III trial:** Firstly, the lack of prehospital brain imaging prior to initiation of oxygen therapy does not allow (1) the exclusion of patients with intracranial haemorrhage (as current experimental and clinical study data does not support use of oxygen therapy in haemorrhagic stroke), (2) the exclusion of patients with ischaemic stroke but without salvageable brain tissue (as no effect of oxygen therapy can be anticipated), (3) comprehensive baseline assessment which we judge crucial in a phase II proof–of–concept study. Secondly, although NBHO as foreseen in the PROOF trial (i.e. high-dose, but short duration, and stop after reperfusion) can be considered safe based on current experimental and clinical study data, nothing replaces the close monitoring by stroke experts not available in the prehospital environment. (modified from **[105]**)

**Have previous human testing studies fulfilled the requirements?**

Not one of the previous human trials testing oxygen therapy has fulfilled the three framework conditions (see Table 3).

Only in one stroke study on humans was oxygen therapy started early – up to 4.5 hours after symptom onset – and reperfusion treatment performed [108], whilst the sole use of intravenous thrombolysis can bring about vessel recanalization and brain tissue reperfusion in only 40 to 50% of cases [109]. Therefore, it can be assumed that despite reperfusion treatment more than half of the patients included in this trial had permanent ischemia, where – as seen in animal experiments – oxygen therapy remains ineffective. In only two (other) human trials, oxygen therapy was administered with a sufficiently high dosage at an $FiO_2$ of 1.0, however, initiated relatively late after symptoms onset (thus salvageable less brain tissue present) and, above all, no reperfusion treatment was performed [76, 110].

Chu and colleagues concluded that the present clinical trial situation excludes a positive effect of oxygen therapy on acute ischemic strokes and that thus further studies are not necessary [111], which is simply and poignantly incorrect, as in no prior clinical trial including humans was oxygen therapy ever given the chance to succeed. Researchers Grensemann [112] and Stolmeijer [113] and their colleagues see this somewhat more differentiated and demand prospective and randomized clinical studies albeit the latter not regarding strokes. His review, dedicated to oxygen therapy in acute ischemic strokes, Shi [114] calls “ideas” for the design of future studies.

Nonetheless, in the few human clinical trials where at least one framework condition was fulfilled, the evidence points to a clinical benefit to acute ischemic strokes: Breathing oxygen in a dose of $FiO_2 \geq 0.95$, which was proven sufficient in animal experiments, led to a stabilization or even a regression of the ischemia (see Fig. 4) and an improvement in NIHSS scores [76]. However, this effect was only temporary because no reperfusion treatment was performed on the patients (‘nothing can hold its breath forever’, compare Fig. 1 and final infarct versus failed recanalization in Fig. 3).

Due to copyright restrictions this figure is not included. Please refer to figure 2 in Singhal et al. 2005 [76]

**Figure 4: Evolution of DWI-lesion volume during permanent MCA-occlusion treated with NBHO**

Serial MRI findings in a patient with cardio-embolic right MCA stroke treated with NBO for 8 hours. Top, Baseline (pre-NBO) MRI, 13.1 hours after symptom onset, shows a large DWI lesion, a larger MTT lesion, and MCA occlusion (arrow) on head MRA. Middle, A second MRI after 3.75 hours (during NBO) shows 36 % reduction in the DWI lesion, stable MTT deficit, and persistent MCA occlusion. Bottom, A third MRI after 24 hours (post-NBO) shows reappearance of DWI abnormality in some areas of previous reversal; MTT image shows partial reperfusion (39 % MTT volume reduction, mainly in the ACA territory); MRA shows partial MCA recanalization. (from [76])

Due to copyright restrictions this figure is not included. Please refer to figure 6 in Shi et al. 2017 [108]

**Figure 5: Normobaric hyperoxia (NBO) reduced blood occludin and improved neurological functions in patients with acute ischemic stroke (AIS).**
**A**, Blood occludin levels in normoxia and NBO-treated patients with AIS. N = 8.
**B**, National Institutes of Health Stroke Scale (NIHSS) scores of patients with AIS. N = 9.
# $p < 0.05$ vs. normoxia group at the same time point. Data were presented as means ± SEM. (from [108])

The only human study in which reperfusion treatment (by means of intravenous thrombolysis) was successful shows a reduction in laboratory parameters that point to blood-brain-barrier damage, as well as an improvement of the NIHSS score at later time points (see Fig. 5) [108].

**How does the PROOF trial differ?**
The PROOF study is the first human NBHO trial to consider all three framework requirements for a successful “freezing of the penumbra” and lasting conservation of positive effects through: (1) early reperfusion (compare Fig. 1 and final infarct versus failed recanalization in Fig. 3): Due to the selected inclusion and exclusion criteria, such as proximal vessel occlusion, a narrow therapeutic time window (six hours) after symptom onset or – in case of wake-up or unknown onset stroke – after symptom recognition, small infarct core at screening, and enrolment of (older) patients only in the case that TBY is likely to be conducted, all patients included in PROOF will likely be treated by endovascular mechanical thrombectomy (TBY) ± intravenous thrombolysis, in which for > 80% of cases complete brain tissue reperfusion is reached, thus achieving transient ischemia, which is deemed necessary for successful oxygen therapy as concluded from animal experiments [109]. (2) Early start of oxygen therapy: the therapeutic time window of six hours after symptom onset was chosen for reasons of practicality and is indeed longer than the 30 minute time window proven effective in animal experiments (compare Fig. 2), however, in contrast to animal experiments, a “target mismatch” on cerebral imaging as stipulated in PROOF guarantees the presence of a substantial volume of salvageable penumbra which is threatened to decline (compare rows 3 and 4 in Fig. 3 and the results of the two TBY trials DAWN [106] and DEFUSE-3 [107]). (3) Sufficient oxygen dosage: through the high flow of 40 L/min or more, even patients who are agitated in the emergency situation and therefore breathe faster and deeper receive oxygen at the $FiO_2$ level of over 0.95, as proven successful in animal experiments (see Table 2).

Through the consistent implementation of all three framework conditions, the PROOF study is thus the first human trial where NBHO has the chance of clinical success.

Additional aspects of the PROOF trial design, such as the determination time point (24 hours) and the choice of the primary end point (imaging and intra-individual control), start of therapy (not prior to hospital admission but and as soon as possible after cerebral imaging) and the duration of therapy (only until reperfusion), reduce the number of lost to follow-up, raise the detection sensitivity for an oxygen effect, thereby minimizing the number of required cases and maximizing patient safety and feasibility, and consequently the chance of success for PROOF and oxygen therapy.

**Table 3: Implementation of prerequisites for successful oxygen therapy (OT) as known from animal experiments into randomized trials testing OT in patients with stroke, myocardial infarction and cardiac arrest**

| | Efficacy | | | Efficacy / safety | Safety |
|---|---|---|---|---|---|
| **Publication,** author (year) | **Early start of OT** ☾ | **Reperfusion attempted after initiation of OT** | **FiO2 ≥ 0.95** | **OT stopped after reperfusion** | **Short OT duration,** mean or median were applicable |
| **Acute myocardial infarction** | | | | | |
| Hofmann et al (2017) | < 6 h | PCI in ~ 2/3 of cases * | 0.5 | no | median 11.6 h |
| Khoshnood et al (2016) | < 6 h | PCI in all cases * | 0.7 | yes | mean 1.49 h |
| Stub et al (2012) | < 12 h | PCI in ~ 3/4 of cases * | 0.6 | study-unrelated OT after PCI | 6.3 h $ |
| Ranchord et al (2012) | < 12 h | PCI in ~ 2/3, and IVT in ~ 1/4 of cases * | 0.5 | no | 6 h ☾ |
| Ukholkina et al (2005) | < 12 h | OT before PCI in ~ 1/2 of cases * | 0.3 – 0.4 | no | 3 or 3.5 h ☾ |
| Wilson et al (1997) | < 24 h | OT after reperfusion | 0.4 | no | 24 h ☾ |
| Rawles et al (1976) | < 24 h | OT after reperfusion | 0.5 | no | 24 h ☾ |
| **Cardiac arrest** | | | | | |
| Young et al (2014) | < 20 min | OT after ROSC | 1.0 | no | 1 h ☾ |
| Kuisma et al (2006) | < 1 h | OT after ROSC | 1.0 | no | 1 h ☾ |
| **Acute ischemic stroke** | | | | | |
| Roffe et al (2017) | < 24 h | no ** | 0.3 | no | 30 or 72 h ☾ |
| Shi et al (2017) | < 4.5 h | IVT in all cases * | 0.7 | short duration of OT | 4 h ☾ |
| Mazdeh et al (2015) | < 12 h | no ** | 0.5 | no | 12 h ☾ |
| Ali et al (2014) | < 12 h | no ** | 0.3 | no | 72 h ☾ |
| NCT00414726 | < 9 h | reperfusion therapy not allowed | 1.0 | no | 8 h ☾ |
| Padma et al (2010) | < 12 h | reperfusion therapy not allowed | 0.7 | no | 12 h ☾ |
| Roffe et al (2010) | < 72 h | no ** | 0.3 | no | 12 h ☾ |
| Chiu et al (2006) | < 48 h | no ** | 0.4 | no | median 96 h |
| Singhal et al (2005) | < 12 h | reperfusion therapy not allowed | 1.0 | no | 8 h ☾ |
| Rønning et al (1999) | < 24 h | no ** | 0.3 | no | 24 h ☾ |
| PROOF | < 6 h | all EVT candidates * | 1.0 | yes | ~75 min to max. 4 h ☾ |

Green, yellow, red colour indicates that prerequisites for successful OT were met, only partly addressed, not fulfilled. ☾ see respective study protocol, * IVT leads to recanalization in only 40 to 50 % compared to > 80 % with EVT or PCI [109], [115], ** Long therapeutic windows renders OT start before reperfusion unlikely, $ estimated value based on median oxygen exposure (2258 L) during first 12 hours divided by median flow-rate (6 L/min), see results, study population [116]

EVT, endovascular mechanical thrombectomy; $FiO_2$, fraction of inspired oxygen; IQR, interquartile range; IVT, intravenous thrombolysis; OT, oxygen therapy; PCI, percutaneous coronary intervention; ROSC, return of spontaneous circulation; SD, standard deviation

**Earlier determination of the primary end point (24 hours)**
Patients included in the PROOF trial will have suffered an ischemic stroke through a proximal vessel occlusion and are thus greatly affected (see above). Due to their old age, many patients will have stated in their living wills or to their next of kin that they do not wish to become in need of care as a result of a severe disability. In the event of unsuccessful acute treatment, therapy limitation in the best interest of the patient is common. However, the decision to limit therapy is made at the earliest after the assessment of the final infarct using cerebral imaging at 24 hours. Instead of the 3-month outcome preferably used in phase III stroke trials, in PROOF – a phase II proof-of-concept study – we will determine the primary endpoint after 24 hours. As a result, all patients will remain analyzable and therapy-independent distortion of study results due to decisions regarding (non-stratifiable) therapy limitations or withdrawal of care and their dissimilar distribution to each study arm, which became the downfall of the NCT00414726 trail by Singhal and colleagues, will be avoided. It is for exactly this reason that the German Society for neuro-intensive and emergency medicine (DGNI) also recently critically discussed the suitability of mortality as a measure of quality in neuro-intensive treatment, including stroke care (see Appendix 2: Sterblichkeitsrate – Qualitätsindikator in der NeuroIntensivmedizin?).

**Imaging and intra-individually controlled primary end point**
Patients with acute stroke receive cerebral imaging (either CT or MRI) as a part of clinical routine both when they are admitted and 24 hours afterwards; on admission including vascular imaging and, in case of a proximal vessel occlusion, including perfusion imaging in order to evaluate salvageable brain tissue and to plan reperfusion treatment. These are optimal conditions for PROOF: without additional effort and without delay in the extremely time-critical acute phase of stroke care the possibility to assess an intra-individually controlled (state of the art) primary imaging end point, the infarct growth from 0 to 24 hours, arises. In contrast to the 24-hour infarct volume, the infarct growth exactly corresponds to the brain tissue, which was initially threatened by decline in an individual patient but was enabled to survive through the acute therapy including study treatment [117]. In contrast to an early clinical end point, such as the 24-hour NIHSS score, infarct growth is not threatened through study treatment independent variables, such as anesthesia hangover (not uncommon after endovascular intervention) or aspiration pneumonia.
Therefore, PROOF uses all available possibilities to be able to prove a group effect in the smallest possible number of cases. Moreover, every individual study patient benefit from the use of MRI in PROOF in place of routine 24-hour CT through lack exposure and diagnostic superiority (with the potential for individual changes to therapy); MRI scans are only avoided in clinical routine for financial reasons.

**Rapid start of oxygen therapy within the hospital environment**
Fig. 2 and Fig. 3 suggest a pre-hospital start of oxygen therapy at the earliest possible time point. Even though this may seem safe [118], in the PROOF trial we begin oxygen therapy in the hospital environment as soon as possible after cerebral imaging and at the latest six hours after onset of symptoms or – in case of wake-up or unknown onset stroke – after symptom recognition: continuous clinical and technical monitoring by an experienced expert team of stroke specialists within the hospital environment guarantees maximum patient safety. Additionally, only cerebral imaging will allow the exclusion of patients who may not benefit from NBHO [105, 119]: stroke patients with cerebral hemorrhage, permanent ischemia, or no salvageable penumbra. As these constitute > 70% of stroke patients, the oxygen effect would (for a phase-two study) be hopelessly diluted and the sample size necessary for its proof enormous. A pre-hospitalization start of therapy should consequently only be evaluated (in a phrase III study), when PROOF has brought the proof-of-concept and shown a positive effect of oxygen therapy in the target group of stroke patients, that – corresponding to animal experiments – should benefit: patients with a salvageable penumbra and transient ischemia. As oxygen is available worldwide at a low cost and easy to use, a subsequent trail (or NBHO per se) would even be worthwhile if the group effect was minimalized through dilution due to inclusion of all stroke patients and the necessary sample size or resulting number-needed-to-treat would be enormous.
The presence of a “target mismatch” on cerebral imaging prior to inclusion compensates for a – in comparison to the animal experiment – prolonged time window (compare Fig. 2), which, for reasons of practicality cannot be shorter in PROOF. After ascertaining the “target mismatch” on cerebral imaging, oxygen therapy must indeed be started as soon as possible as the penumbra may occasionally shrink rapidly (compare Fig. 3 and see paragraph 4.1. Patient selection in Poli et al. [120]).
Due to the inclusion and exclusion criteria (including proximal vessel occlusion, six-hour time window, small infarct core, and likelihood of conduction of TBY) patients included in PROOF will likely all be treated with TBY, and oxygen therapy – as required by the study protocol – will be stopped at the end of TBY as the continuation of oxygen therapy beyond reperfusion has not been shown to provide additional benefit (in

animal experiments) [54]. Through constant optimization of acute stroke care, imaging-to-reperfusion time today lies under 90 minutes [121] meaning that the duration of oxygen therapy in PROOF – taking into account a short verbal patient information, screening and randomization (approx. 15 minutes) – should only last 75 minutes.
With every delay, both the volume of salvageable cerebral tissue (penumbra) and the duration of therapy are critically reduced and thus not only the potential positive effect of oxygen (in individuals), but also the chance of showing it in PROOF (group effect) diminish.
Instead of waiting until stroke physicians and neuro-interventionalists have conferred and the indication for TBY has been established (approx. 10 minutes), the PROOF inclusion and exclusion criteria require that likely all patients are treated with TBY and the ischemia – essential for successful oxygen therapy – is not permanent but transient.
Following knowledge gathered in animal experiments, the target stroke patient population chosen in PROOF – in contrast to other stroke patients (see above) – benefits from oxygen therapy on a relevant level. The chosen stroke patients are severely affected and they are unable to give consent; without exception either through a speech impairment or neglect to the point of anosognosia; in addition, they suffer from paralysis and the distress of the acute admission. There is insufficient time to implement a legally authorized representation by a next of kin and also the delay caused by detailed written informed consent would critically reduce the positive effect of the oxygen therapy.
PROOF has therefore chosen to enroll patients according to the (upcoming) EU regulation 536/2014, which allows inclusion of patients in a clinical trial without prior consent in case several conditions for emergency situations are fulfilled, and which is also in line with local regulations: e.g. §41 Abs. 1 Satz 2 of the German Drug Law (AMG) and §21 Nr. 3 Satz 3 of the Medicinal Devices Act (MPG) (see Section 14.5 Subject Information and Informed Consent). These laws directly address the type of emergency situation and the time dependent emergency therapy seen and evaluated in PROOF. All patients are severely affected and therefore unable give consent and the high-dose oxygen therapy – as thoroughly shown – can be expected to be of great benefit to each individual patient as long as it is initiated without delay. In the following we will show that no included patient is at risk due to the NBHO used the PROOF trial and therefore all requirements for emergency inclusion according to the (upcoming) EU regulation 536/2014 and local regulations are fulfilled.
The patient organization Stiftung Deutsche Schlaganfall-Hilfe (www.schlanganfall-hilfe.de) and S.A.F.E (www.safestroke.eu/research/) support the PROOF study and previous patient surveys highlight stroke patients' demand to evaluate oxygen therapy regarding its positive effects; 92% of those asked would also accept a deferred consent procedure after emergency patient inclusion in the trial [122].

**Safety of normobaric oxygen therapy planned in PROOF**
Due to the PROOF inclusion and exclusion criteria (including proximal vessel occlusion, six-hour time window, small infarct core, and likelihood of conduction of TBY) nearly all prospective patients will be treated with a TBY and oxygen therapy will, as stated in the protocol – be discontinued at the end of the TBY procedure; the prospective duration of oxygen therapy will thus be shorter than 75 minutes for nearly all patients. Only in the most unlikely case that a patient suitable for PROOF is not treated with TBY, NBHO will be applied for four hours so that these isolated cases have the chance to benefit through "freezing the penumbra" until spontaneous reperfusion, which occurs in 20-30% and, thus, compared to TBY is less frequently and for the most part delayed. According to the Summary of Product Characteristics (SmPC), reversible (pulmonary) side effects are only to be expected after a NBHO duration of > 6 hours and relevant (pulmonary) side effects only after > 12 hours (see Section 4.3 Risk-benefit Assessment).
A meta-analysis published by Chu and colleagues in The Lancet in 2018 has raised significant doubts regarding the safety of NBHO [111]. Altogether, the meta-analysis included 16,037 "acutely ill" adult patients and sweepingly concluded that oxygen therapy would lead to a higher mortality rate and causes damage to the patients, whilst a potpourri of illnesses was included in the analysis in an undifferentiated manner: Trauma, sepsis, stroke (ischemic as well as hemorrhagic), myocardial infarction, cardiac arrest, emergency operations as well as a mixed collective of illnesses requiring intensive care. Grensemann [112] and Stolmeijer [113] indeed follow the same tenor, but do differentiate between illnesses and do not draw definite conclusions regarding stroke patients. Stolmeijer and colleagues even recognize a benefit, albeit only a slight one [113]. None of the reviews, however, recognize the quite different therapy goals of the collectively evaluated studies (i.e. avoidance of hypoxia versus normobaric hyper-oxygenation) and thus the oxygen therapy dosage ($FiO_2$ 0.3 to 1.0) (see Table 5), the therapeutic time window (see Table 6) and the length of use (from 4 to 72 hours) (see Table 6) are neglected. Also, there is no differentiation of patients with transient ischemia receiving reperfusion treatment, permanent ischemia or missing penumbra (see Table 4).

**Table 4: Baseline characteristics of randomized trials testing OT in patients with stroke, myocardial infarction and cardiac arrest**

| **Publication,** author (year) | **Study acronym** | **Study type** | **Centers** | **Blinding** | **Country** | **Target condition** | **N** enrolled | **Study population,** % (n) | **Women,** % (n) | **Age,** mean ± SD or median (IQR) | **NIHSS liberal OT,** mean ± SD or median (IQR) | **NIHSS conservative CTRL,** mean ± SD or median (IQR) |
|---|---|---|---|---|---|---|---|---|---|---|---|---|
| **Acute myocardial infarction** | | | | | | | | | | | | |
| Hofmann et al (2017) | DETO2X-AMI | RCT | multi | open | Sweden | suspected AMI | 6629 | STEMI 44.5 (2952), NSTEMI 31.0 (2058), no AMI 24.4 (1619) | 30.5 (2023) | 68 (59 – 76) | n/a | n/a |
| Khoshnood et al (2016) | SOCCER | RCT | 2 | single-blind | Sweden | STEMI | 160 | " | 34.4 (55) **33.8 (54)** ✣ | 66 ± 12 §§ | n/a | n/a |
| Stub et al (2012) | AVOID | RCT | multi | open | Australia | suspected STEMI | 638 **624** ✣ | STEMI 70.1 (447), NSTEMI 3.8 (24), no AMI 17.7 (113), unknown 8.5 (54) | 22.7 (145) § **22.8 (142)** ✣ | 63 (54 – 72) | n/a | n/a |
| Ranchord et al (2012) | OPTIMISE | RCT | 2 | open | New Zealand, UK | STEMI | 148 | STEMI 93.2 (138), no AMI 3.4 (5), unknown 3.4 (5) | 25.7 (38) § **31.8 (47)** ✣ | 61 ± 13 §§ | n/a | n/a |
| Ukholkina et al (2005) | - | RCT | 1 | open | Russia | AMI | 137 | " | 16.1 (22) | 54 ± 10 §§ **mean 55** ✣ | n/a | n/a |
| **Wilson et al (1997)** ☾ | - | RCT | 1 | open | UK | STEMI | 50 | " | 40 (20) § | mean 64 §§ | n/a | n/a |
| Rawles et al (1976) | - | RCT | 1 | double-blind | UK | suspected AMI | 200 | AMI 78.5 (157), no AMI 21.5 (43) | 20 (40) | 55 ± 8 §§ **mean 56** ✣ | n/a | n/a |
| **Cardiac arrest** | | | | | | | | | | | | |
| Young et al (2014) | HOT OR NOT | RCT | multi | single-blind | New Zealand | VF/VT-OHCA after ROSC | 18 | " | 6 (1) | 66 ± 17 §§ | n/a | n/a |
| Kuisma et al (2006) | - | RCT | 1 | open | Finland | VF/VT-OHCA after ROSC | 32 **28** ✣ | " | 19 (6) § | 63 ± 11 §§ | n/a | n/a |
| **Acute ischemic stroke** | | | | | | | | | | | | |
| Roffe et al (2017) | SO2S | RCT | multi | open, blinded endpoint | UK | stroke | 8003 **5336** ✣ | AIS 81.9 (6555), no AIS 13.1 (1048) [TIA 2.1 (168), ICH 7.3 (588), no stroke 3.6 (292)], unknown 5 (400) | 45.0 (3605) **45.1 (2404)** ✣ | 72 ± 13 | 5 (3 – 9) | 5 (3 – 9) |
| Shi et al (2017) | - | RCT | 1 | open | China | AIS, IVT candidates | 18 | " | 28 (5) | mean 60, range 47 – 75 | mean 12, range 5 – 20 | mean 12.3, range 7 – 18 |
| Mazdeh et al (2015) | - | RCT | 1 | open | Iran | stroke | 52 | AIS 73 (38), ICH 25 (13), unknow 2 (1) | 44 (23) **46 (24)** ✣ | 40 to 70 * | 7 to 9 * | 7 to 9 * |
| Ali et al (2014) | - | RCT | 1 | open | UK | stroke | 301 | AIS 84.4 (254), no AIS 13.3 (40) [TIA 2.0 (6), ICH 7.6 (23), SDH 0.3 (1), no stroke 3.3 (10)], unknown 2.3 (7) | 53.2 (160) § | 72 ± 12 | 6 (3 – 10) | 5 (3 – 10) |
| NCT00414726 | - | RCT | 1 | double-blind | USA | AIS, ineligible for IVT/IAT | 85 ✣✣ | LVO present in 46 (39) | 52 (44) | 74 ± 14 §§ | 11 (4 – 26) | 12 (4 – 23) |
| Padma et al (2010) | - | RCT | 1 | open | India | AIS, ineligible for IVT | 40 | " | NR | 56 ± 13 | mean 14.25 | mean 12.7 |
| **Roffe et al (2010)** ☾ | - | RCT | 1 | open | UK | stroke | 63 | AIS 57 (36), no AIS 41 (26) [ICH 37 (23), SDH 3 (2), no stroke 2 (1)], unknown 2 (1) | 41 (26) § | 74 ± 9 | 8 (6 – 14) ** | 15 (5 – 18) ** |
| **Chiu et al (2006)** ☾ | - | non-RCT | 1 | open | Taiwan | AIS, > 1/3 of MCA territory | 46 | " | 70 (32) | 79 ± 11 §§ | 20.8 ± 3.8 | 18.9 ± 6 |
| Singhal et al (2005) | - | RCT | 1 | open | USA | AIS with perfusion-diffusion mismatch, ineligible for IVT/IAT | 16 | LVO present in all patients | 56 (9) | mean 68, range 37 – 97 | 14 (12 – 18) | 11 (11 – 13) |
| Rønning et al (1999) | - | quasi-RCT ☾☾ | 1 | open | Norway | stroke | 550 | AIS 88 (484), ICH 12 (66) | 46.9 (258) | 76 ± 7 §§ | 8 (5 – 16) ** | 7 (4 – 15) ** |
| PROOF study group | PROOF | RCT | ~ 25 | open | Europe | AIS with LVO and target mismatch | 456 | | | ≤ 80 * | ≥ 6 * | ≥ 6 * |

☾ studies not considered by Chu et al. [111], ☾☾ randomization based on birth numbers, ✣ reported by Chu et al. [111], ✣✣ terminated after enrolment of 85 (of 480) patients [see ClinicalTrials.gov, NCT00414726], " equals target condition, § extrapolated to number of randomized patients, §§ combined mean and standard deviation of individual study groups, * as per inclusion criterion, ** converted Scandinavian Stroke Scale scores [123]

AIS, acute ischemic stroke; AMI, acute myocardial infarction; IAT, intra-arterial thrombolysis; ICH, intracerebral hemorrhage; IQR, interquartile range; IVT, intravenous thrombolysis; LVO, large vessel occlusion; MCA, middle cerebral artery; n/a, not applicable; NR, not reported; NSTEMI, non-ST elevation myocardial infarction; OT, oxygen therapy; RCT, randomized controlled trial; ROSC, return of spontaneous circulation; SAH, subarachnoid hemorrhage; SD, standard deviation; SDH, subdural hematoma; STEMI, ST elevation myocardial infarction; TIA, transient ischemic attack; UK, United Kingdom; USA, United States of America; VF/VT-OHCA, out–of–hospital cardiac arrest with ventricular fibrillation/pulseless ventricular tachycardia as initial rhythm

**Table 5: Dosing of Oxygen Therapy (OT) in randomized trials testing OT in patients with stroke, myocardial infarction and cardiac arrest**

| | **Conservative OT** | | | | **Liberal OT** | | | | |
|---|---|---|---|---|---|---|---|---|---|
| **Publication,** author (year) | **Conservative OT regimen** | **FiO2** | **SpO2 % baseline,** mean ± SD or median (IQR) | **SpO2 % during conservative OT,** mean ± SD or median (IQR) | **Mask type** | **Flow-rate,** L/min ✣ | **FiO2** | **SpO2 % baseline,** mean ± SD or median (IQR) | **SpO2 % during liberal OT,** mean ± SD or median (IQR) |
| **Acute myocardial infarction** | | | | | | | | | |
| Hofmann et al (2017) | air | 0.21 | 97 (95 – 98) | 97 (95 – 98) | FM | 6 | 0.5 | 97 (95 – 98) | 99 (97 – 100) |
| Khoshnood et al (2016) | air via FM | 0.21 | 98 ± 2 | 97 ± 2 | FM | 10 | 0.7<br>0.74 ✣✣ | 98 ± 2 | 99 ± 1 |
| Stub et al (2012) | air | 0.21 | 98 (96 – 99) | 98 (96 – 99) | FM | 8 | 0.6 | 98 (97 – 99) | 100 (99 – 100) |
| Ranchord et al (2012) | O2 titrated to SpO2 93 – 96 % | ≥ 0.21 | NR | NR | FM | 6 | 0.5 | NR | NR |
| Ukholkina et al (2005) | air | 0.21 | mean 93 | mean 94 | NC | 3 – 6 | 0.3 – 0.4 | mean 94 | mean 99 |
| Wilson et al (1997) ☾ | air | 0.21 | NR | mean of lowest 81, range 65 – 88 | FM | 4 | 0.4 | NR | mean of lowest 85, range 77 – 89 |
| Rawles et al (1976) | air via FM at 6 L/min | 0.21 | NR | PaO2: 67 ± 9 mmHg [9.0 ± 1.2 kPa] * | FM | 6 | 0.5 | NR | PaO2: 153 ± 44 mmHg [20.4 ± 5.9 kPa] * |
| **Cardiac arrest** | | | | | | | | | |
| Young et al (2014) | IMV at FiO2 titrated to SpO2 90 – 94 % | ≥ 0.4 | NR | 80 (76 – 90) | IMV | n/a | prehospitally 1.0 | NR | 96 (92 – 97) |
| Kuisma et al (2006) | IMV at FiO2 0.3 | 0.3 | NR | 98 ± 1, PaO2: mean 158 mmHg [21.1 kPa] | IMV | n/a | 1.0 | NR | 100 ± 1, PaO2: mean 373 mmHg [49.7 kPa] |
| **Acute ischemic stroke** | | | | | | | | | |
| Roffe et al (2017) | air | 0.21 | 97 ± 2 | mean of highest 98 | NC | 2 – 3 | 0.3 | 97 ± 2 | mean of highest 99 |
| Shi et al (2017) | air | 0.21 | NR | NR | FM | 10 | 0.7<br>0.69 ✣✣ | NR | NR |
| Mazdeh et al (2015) | air | 0.21 | NR | NR | VM | n/a | 0.5 | NR | NR |
| Ali et al (2014) | air | 0.21 | 96 ± 2 | 96 ± 2 | NC | 2 – 3 | 0.3 | 96 ± 2 | 97 ± 1 |
| NCT00414726 | air via FM at 30 – 45 L/min | 0.21 | NR | NR | FM | 30 - 45 | 1.0 | NR | NR |
| Padma et al (2010) | air | 0.21 | NR | NR | FM | 10 | 0.7<br>0.55 ✣✣ | NR | NR |
| Roffe et al (2010) ☾ | air | 0.21 | 96 ± 2 | 94 (94 – 95) | NC | 2 | 0.3 | 95 ± 2 | 97 (95 – 98) |
| Chiu et al (2006) ☾ | O2 via NC at 2 L/min | 0.3 | NR | NR | VM | n/a | 0.4 | NR | NR |
| Singhal et al (2005) | air | 0.21 | NR | PaO2 in one patient: 99 mmHg [13.2 kPa] | FM | 45 | 1.0 | NR | PaO2 in two patients: 368 and 420 mmHg [49.1 and 56.0 kPa] |
| Rønning et al (1999) | air | 0.21 | NR | NR | NC | 3 | 0.3 | NR | NR |
| PROOF study group | air | 0.21 | | | non-rebreather FM with reservoir | ≥ 40 | 1.0 | | |

☾ study not considered by Chu et al. [111], * combined mean and standard deviation of individual study groups, ✣ see respective study protocol, ✣✣ reported by Chu et al. [111]

FiO2, fraction of inspired oxygen; FM, face mask; IMV, invasive mechanical ventilation; IQR, interquartile range; n/a, not applicable; NC, nasal cannula; NR, nor reported; OT, oxygen therapy; PaO2, partial pressure of oxygen in the arterial blood; SD, standard deviation; SpO2, saturation of peripheral oxygen; VM, Venturi mask

**Table 6: Enrolment Procedure, Therapeutic Window and Duration of Oxygen Treatment (OT) in randomized trials testing OT in patients with stroke, myocardial infarction and cardiac arrest**

| | | | Liberal OT | | | | |
|---|---|---|---|---|---|---|---|
| **Publication,** author (year) | **Site of study treatment initiation** | **Enrolment procedure,** % (n) where applicable | **max. time window to initiate liberal OT,** hours ☾☾ | **symptom onset–to–liberal OT,** hours, mean ± SD or median (IQR) | **Liberal OT started before reperfusion (therapy),** % (n) where applicable | **Duration of liberal OT,** hours, mean ± SD or median (IQR) | **Liberal OT stopped after reperfusion** |
| **Acute myocardial infarction** | | | | | | | |
| Hofmann et al (2017) | pre- or in-hospital | oral IC in ambulance (EMS) or ED; written IC within 24 hours | 6 | median 4.1 | yes, 65.9 (2183) underwent PCI | 11.6 (6 – 12) | no |
| Khoshnood et al (2016) | prehospital | oral IC in ambulance (EMS); written IC within 72 hours | 6 | mean 2.1 ✣ | yes, in all cases | 1.49 ± 0.6 | yes, OT stopped at end of PCI |
| Stub et al (2012) | prehospital | oral IC in ambulance (EMS); delayed written IC | 12 | mean 1.2 ✣✣ | yes, 73.9 (235) underwent PCI | pre-PCI OT: 1.3 (1.0 – 1.6), total OT: 6.3 $ | no, study unrelated post-PCI OT |
| Ranchord et al (2012) | in-hospital | short written IC; detailed written IC after acute STEMI therapy | 12 | NR | yes, 25 (18) received IVT, 67 (48) underwent PCI | 6 ☾☾ | no |
| Ukholkina et al (2005) | in-hospital | NR | 12 | 4.6 ± 0.3 § | yes, 48 (28) received OT 30 min before PCI | pre-PCI OT: 0.5 hours in 28 patients, post-PCI OT in all patients: 3 hours ☾☾ | no |
| Wilson et al (1997) ☾ | in-hospital | NR | 24 | NR | no | 24 ☾☾ | no |
| Rawles et al (1976) | in-hospital | NR | 24 | NR | no | 24 ☾☾ | no |
| **Cardiac arrest** | | | | | | | |
| Young et al (2014) | prehospital | consent waiver; assent from next of kin after ICU arrival; delayed written IC from patients where possible | < 20 min after ROSC | 0.5 ± 0.3 §§ | no | < 1 $$ | no |
| Kuisma et al (2006) | prehospital | consent waiver | < 1 hour after ROSC | 0.3 ± 0.1 §§ | no | 1 ☾☾ | no |
| **Acute ischemic stroke** | | | | | | | |
| Roffe et al (2017) | in-hospital | written IC by patient in 87.4 (6991), or assent from next of kin, caregiver, or LAR in 12.6 (1012); reassessments at 1 week and 3 months | 24 | continuous OT 20.7 (1.9 – 25.6), nocturnal OT 20.5 (12.1 – 25.5) * | no | 72 hours or 3 x 10 hours during first three nights ☾☾ | no |
| Shi et al (2017) | in-hospital | written IC | 4.5 | mean 3.2, range 1 – 4.5 * | yes, in all cases | 4 ☾☾ | (yes) |
| Mazdeh et al (2015) | in-hospital | written IC | 12 | NR | no | 12 ☾☾ | no |
| Ali et al (2014) | in-hospital | written IC by patient or assent from next of kin; reassessment at 1 week | 24 | 17.8 ± 8.1 * | no | 72 ☾☾ | no |
| NCT00414726 | in-hospital | written IC by patient or assent from LAR | 9 | 4.3 ± 2.2 ** | no, exclusion of IVT/IAT candidates | 8 ☾☾ | permanent ischemia |
| Padma et al (2010) | in-hospital | written IC | 12 | 8.5 ± 2.2 * | no, exclusion of IVT candidates | 12 ☾☾ | permanent ischemia |
| Roffe et al (2010) ☾ | in-hospital | written IC by patient or assent from next of kin; reassessment at 1 week | 72 | 45.6 ± 20.4 | no | 12 during first night ☾☾ | no |
| Chiu et al (2006) ☾ | in-hospital | written IC | 48 | mean 13.7, median 13, range 3 – 41.5 | no | mean 132.9, median 96, range 48 – 168.5 | no |
| Singhal et al (2005) | in-hospital | written IC | 12 | NR | no, exclusion of IVT/IAT candidates | 8 ☾☾ | permanent ischemia |
| Rønning et al (1999) | in-hospital | written IC by patient or next of kin | 24 | 0 – 3 hours: 49 patients (19 %), 3 – 6 hours: 72 patients (27.9 %), > 6 hours or unknown: 137 patients (46.9 %) * | no | 24 ☾☾ | no |
| PROOF study group | in-hospital | oral IC in ED, written IC within 72 hours | 3 | | yes, all EVT candidates | ~75 min to max. 4 hours ☾☾ | yes, OT to be stopped at end of EVT |

☾ studies not considered by Chu et al. [111], ☾☾ see respective study protocol, ✣ estimated value: mean symptom onset–to–ambulance arrival time (110.9 min) plus 15 min, see table 1 in [124], ✣✣ estimated value: median symptom onset–to–PCI time (150.5 min) minus median duration of pre-PCI OT (79.0 min), see table 2 in [115], § symptom onset–to–PCI, §§ symptom onset–to–ROSC, * symptom onset–to–randomization, ** symptom onset–to–brain imaging, $ estimated value: median supplemental O2 exposure (2258 L) during first 12 hours divided by median flow-rate (6 L/min) in OT group, see results, study population in [116]

ED, emergency department; EMS, emergency medical services; IAT, intra-arterial thrombolysis; IC, informed consent; IQR, interquartile range; IVT, intravenous thrombolysis; LAR, legally authorized representative; NR, nor reported; OT, oxygen therapy; PCI, percutaneous coronary intervention; ROSC, return of spontaneous circulation; SD, standard deviation; STEMI, ST elevation myocardial infarction

To address the safety concerns and critically challenge our favorable risk assessment of Section 4.3 Risk-benefit Assessment, we have performed our own meta-analysis (see Appendix 1), which comprises all randomized controlled trials (RCT) relevant to the PROOF target patient population (i.e. ischemic stroke), that is to say – due to comparable age, accompanying illnesses and vascular risk factors – also RCT including patients with hemorrhagic stroke, myocardial infarction or cardiac arrest. We analyzed mortality (in hospital, on day 30 and last follow-up), disability, infection, pneumonia and the duration of

inpatient treatment – like in [111] – related to "any oxygen therapy" on the one hand and focused on high-flow oxygen therapy with "$FiO_2$ > 0.5" on the other as it approximates oxygen therapy planned within PROOF. Additionally, we evaluated mortality on day 90 as this represents the time of primary survey time point in all recent clinical phase III trials for ischemic stroke, including PROOF. Checking the original publications, we found discrepancies to Chu's meta-analysis; amongst other things, 2,667 patients from [90] were treated with oxygen therapy but left unconsidered in Chu's meta-analysis, all other differences are marked in Tables 4 and 5 as well as 7, 8 and 9.
The risk of bias of the underlying RCT was for the most part valued as positive by Chu [111] (see Table 10) but not by us (see Table 11) or Cabello's 2016 Cochrane meta-analysis of oxygen therapy for myocardial infarction [92]. In the latter and through a search of PubMed emerged two RCT of relevance [125, 126].
Our analyses consequently comprised the following: (I) the same studies with data as given in Chu et al. (max. of 14,340 patients), (II) data corrected according to original publications (up to 16,718 patients), (III) all RCT (up to 17,066) and (IV) all stroke RCT (up to 9,069 patients). In addition, we have repeated all of our analyses excluding the prematurely terminated NCT00414726 study (https://clinicaltrial.goc/ct2/show/NCT00414726).
The increased mortality in in the oxygen therapy arm, which led to the termination of the NCT00414726 study, can be traced back to an imbalance in the distribution of critical base line characteristics (poorer previous health, more comorbidities, more proximal vessel occlusions without reperfusion treatment). The consecutive distortion in mortality is a result of therapy limitations and not the result of oxygen therapy (see above, Appendix 2, Appendix 3, and [77]); 15 out of 24 deaths were due to the therapy limitations; this is a central aspect, which McEvoy disregards in his letter [127]. Singhal conducted the NCT00414726 and advises PROOF as part of the Scientific Advisory Board.
None of our analyses from (I) to (IV) for "any oxygen therapy", "$FiO_2$ > 0.5", "with NCT00414726" and "without NCT00414726" could confirm the detrimental effect regarding mortality (in-hospital, on day 30, day 90 and at last follow-up), disability, infection and pneumonia (see all analyses in Appendix 1) which was shown by Chu's meta-analysis for the mixed collective (that is to say including illnesses and patients that resemble stroke patients neither in pathophysiology nor regarding comorbidities or risk factors); only the length of hospitalization was significantly longer in the intervention arm, incidentally only after correction of the data according to the original publications.
An interesting observation is that the AVOID study [115], which is for the most part and, according to the original conclusions also in [111] and [112], interpreted as a study corroborating the harmfulness of oxygen therapy in myocardial infarction, yielded astonishingly positive results regarding mortality.
In their review regarding oxygen therapy in animal models of ischemic stroke, Weaver et al. [70] clearly showed that short term oxygen therapy until reperfusion – like it is planned in PROOF – neither lead to an increase in free radicals nor oxidative stress for neuronal tissue, but actually the opposite. The association between hyperoxygenation and aggravation of a hypoxic-ischemic encephalopathy in neonatal asphyxia shown by Kapadia and colleagues [128] in a retrospective study is not to be worried about in PROOF as the brain's vulnerability to oxygen toxicity is – as it was shown not only by Felderhoff-Mueser et al. – tightly/strictly limited to the infantile phase of brain development, i.e. until end of the second week in rodents and the end of the third life year in humans [129], which is far off the age of patients included in PROOF.
In conclusion, our new analysis, supports our previous risk benefit analysis (see Section 4.3 Risk-benefit Assessment). In order to render trial participation in PROOF as safe as possible for study patients we have expanded the DSMB Charter according to the Tübingen University Hospital ethics committee's (protocol version 1.3) and the DSMB's requests (protocol version 1.4) to continuously monitor the mortality rate and the rates of parenchymal hematoma and remote intracranial bleeding (see Appendix 14 for Heidelberg bleeding classification [101]), respectively. This means that the DSMB and the sponsor will be simultaneously informed of every death, every parenchymal hematoma, and every remote intracranial bleeding. As the value of the mortality rate a measure of therapy success or harmfulness in neuro-intensive patients including stroke patients nevertheless seems limited (compare Appendix 2), every single death will be examined in detail taking into account patients' wishes for therapy limitations.
To enable a "security check" in PROOF even before a decision regarding a possible therapy limitation is made, we have decided to implement an additional frequent (i.e. at every DSMB meeting, see Section 4.4 Data and Safety Monitoring Board) evaluation of early neurological change (delta NIHSS from screening to V2) after the start of oxygen therapy (in the intervention arm) in connection with the primary end point, the infarct growth from prior to study intervention to 24 hours, in the DSMB Charter, so to be able to immediately detect potentially outcome-relevant short-term influences of oxygen therapy on e.g. cerebral perfusion and possibly end the study prematurely. Based on the many animal studies (see [105] for overview) and Singhal's human trial [76], we can, however, assume a stabilization [62, 63, 66-68, 85, 86, 130] or even an improvement [54, 56, 62, 66, 131] of penumbral perfusion and we can also assume that the (due to the lack

of reperfusion treatment only) transient effects – MR-tomographical and clinical stabilization to improvement – shown by Singhal [76] will be rendered permanent through the TBY treatment administered in PROOF, specifically by vessel recanalization and brain tissue reperfusion.
40,872 patients with suspected ACS were enrolled into the **New Zealand Oxygen in Acute Coronary Syndromes Trial** (NZOTACS) [102]. 4,159 patients (10%) had the final diagnosis of ST elevation myocardial infarction (STEMI), 10,218 of Non-STEMI, 3,284 of unstable angina, 19,519 had no ACS and 3,692 were not classified. 20,304 were treated with high-flow oxygen. Overall, 30-day mortality was same in the low and the high oxygen groups. 30-day mortality by final diagnosis however, was same for no-ACS and Non-STEMI, but was reduced in STEMI patients who were treated with high-flow oxygen compared to low oxygen (Odds ratio 0.81, 95% confidence interval 0.66-1.00) [102]. These results do first of all confirm the overall safety of NBHO in a (huge) population very similar to PROOF candidates, i.e. patients with a similar condition and similar vascular risk factors. Second, the beneficial NBHO effects observed in NZOTACS in STEMI patients reinforce us in our choice of focusing on TBY candidates with LVO-associated anterior circulation ischemic stroke, i.e. stroke patients very much resembling STEMI patients in whom vessel recanalization and tissue reperfusion is achieved by percutaneous coronary intervention (PCI).

**Table 7: Mortality in randomized trials testing OT in patients with stroke, myocardial infarction and cardiac arrest**

| Publication, author (year) | N enrolled | Longest FU at, months | Deaths liberal OT in-hospital, n/analyzed n | Deaths conservativ OT in-hospital, n/analyzed n | Deaths liberal OT 30 days, n/analyzed n | Deaths conservative OT 30 days, n/analyzed n | Deaths liberal OT 90 days, n/analyzed n | Deaths conservative OT 90 days, n/analyzed n | Deaths liberal OT longest FU, n/analyzed n | Deaths conservative OT longest FU, n/analyzed n | Source of data |
|---|---|---|---|---|---|---|---|---|---|---|---|
| **Acute myocardial infarction** | | | | | | | | | | | |
| Hofmann et al (2017) | 6629 | 12 | 53/3311 | 44/3318 | 73/3311 | 67/3318 | 93/3311 | 94/3318 | 166/3311 | 168/3318 | 1 |
| Khoshnood et al (2016) | 160 | 6 | 3/85 | 3/75 | 3/85 ☾☾ | 3/75 ☾☾ | 3/85 | 3/75 | 3/85 | 3/75 | 2 |
| Stub et al (2012) | 638<br>624 ✣ | 6 | 5/312 | 11/312 | NR<br>12/312 ✣ | NR<br>14/312 ✣ | | | 8/218<br>14/312 ✣ | 13/223<br>18/312 ✣ | 3 |
| Ranchord et al (2012) | 148 | 1 | | | BC: 1/72<br>WC: 3/72<br>1/72 ✣ | BC: 4/76<br>WC: 2/76<br>2/76 ✣ | | | | | 4 |
| Ukholkina et al (2005) | 137 | in-hospital | 1/58 | 0/79 | | | | | | | 5 |
| Wilson et al (1997) ☾ | 50 | in-hospital | BC: 0/25<br>WC: 1/25 | BC: 1/25<br>WC: 0/25 | | | | | | | 6 |
| Rawles et al (1976) | 200 | in-hospital | 9/105 | 3/95 | | | | | | | 7 |
| **Cardiac arrest** | | | | | | | | | | | |
| Young et al (2014) | 18 | in-hospital | 5/9 | 4/8 | | | | | | | 8 |
| Kuisma et al (2006) | 32<br>28 ✣ | in-hospital | BC: 4/16<br>WC: 6/16<br>4/14 ✣ | BC: 6/16<br>WC: 4/16<br>4/14 ✣ | | | | | | | 9 |
| **Acute ischemic stroke** | | | | | | | | | | | |
| Roffe et al (2017) | 8003<br>5336 ✣ | 3 | 85/5297<br>50/2668 ✣ | 45/2664<br>45/2668 ✣ | 245/5301<br>138/2668 ✣ | 140/2665<br>140/2668 ✣ | 493/5128<br>268/2668 ✣ | 246/2549<br>256/2668 ✣ | | | 10 |
| Shi et al (2017) | 18 | in-hospital | 0/9 | 0/9 | | | | | | | 11 |
| Mazdeh et al (2015) | 52 | 6 | | | | | | | 5/26 | 3/25 | 12 |
| Ali et al (2014) | 301 | 6 | 5/148<br>5/155 ✣ | 4/141<br>4/146 ✣ | 12/148<br>13/155 ✣ | 11/141<br>11/146 ✣ | 18/148 | 17/141 | 22/126<br>26/155 ✣ | 21/130<br>23/146 ✣ | 13 |
| NCT00414726 | 85 | 3 | 14/43 | 4/42 | 14/43 | 4/42 | 17/43 | 7/42 | | | 14 |
| Padma et al (2010) | 40 | 3 | 0/20 | 2/20 | 0/20 | 2/20 | 0/20 | 2/20 | | | 15 |
| Roffe et al (2010) ☾ | 63 | in-hospital | 2/29 | 3/30 | | | | | | | 16 |
| Chiu et al (2006) ☾ | 46 | in-hospital | 1/17 | 7/29 | | | | | | | 17 |
| Singhal et al (2005) | 16 | 3 | 0/9 | 1/7 | 1/9 | 1/7 | 2/9<br>2/9 ✣ | 1/7<br>2/7 ✣ | | | 18 |
| Rønning et al (1999) | 550 | 12 | 36/292 | 27/258 | 55/292 | 38/258 | 66/292 | 46/258 | 91/292 | 70/258 | 19 |

☾ study not considered by Chu et al. [111]; ☾☾ mortality data not analyzed by Chu et al. [111]; ✣ reported by Chu et al. [111];

[1] figure 2 [132]; [2] figure 1 [124]; [3] tables S4 and 4 for mortality at discharge and at six months [115]; [4] table V plus two cases of cardiogenic shock (see results) [133], compare [134]; [5] study results, clinical course of myocardial infarction [135]; [6] results, intervention study [126]; [7] tables I and II [136]; [8] supplementary data, tertiary end points [137]; [9] results, survival plus two cases of sustained return of spontaneous circulation of less than 60 min [138]; [10] Figure 1, and table 2 / figure 4 / figure 2 for mortality at 7 / 30 / 90 days [139]; [11] results, normobaric hyperoxygenation reduced blood occludin and improved neurological functions in patients with acute ischemic stroke [108]; [12] material and methods, results, and table 2 [140]; [13] results, neurological outcome at one week [141] / figure 3 / figure 2 [89] for mortality at 7 / 30 and 90 days / six months; [14] [ClinicalTrials.gov identifier: NCT00414726], [Singhal AB et al., unpublished data]; [15] results [142]; [16] results, effect on physiologic parameters and stroke progression [125]; [17] table 2 [75]; [18] results, and appendix, individual patient data [76] and table 1 [143] for mortality at 7 / 90 days; [19] figure 2 / table 2 for mortality at 7, 30, 90 days / 12 months [74]

FU, follow up visit; OT, oxygen therapy

**Table 8: Disability (modified Rankin Scale Score) in randomized trials testing OT in patients with stroke, myocardial infarction and cardiac arrest**

| | | | | | Liberal OT | | | | | | | Conservative OT | | | | | | | |
|---|---|---|---|---|---|---|---|---|---|---|---|---|---|---|---|---|---|---|---|
| **Publication,** author (year) | **N** enrolled | **Liberal OT n** analyzed | **Conservative OT n** analyzed | **mRS assessed at,** months | **mRS 0,** n | **mRS 1,** n | **mRS 2,** n | **mRS 3,** n | **mRS 4,** n | **mRS 5,** n | **mRS 6,** n | **mRS 0,** n | **mRS 1,** n | **mRS 2,** n | **mRS 3,** n | **mRS 4,** n | **mRS 5,** n | **mRS 6,** n | **Source of data** |
| **Acute ischemic stroke** | | | | | | | | | | | | | | | | | | | |
| Roffe et al (2017) | 8003<br>5336 ✣ | 5128<br>2567 ✣ | 2549 | 3 | 605<br>313 ✣ | 1399<br>690 ✣ | 637<br>322 ✣ | 883<br>461 ✣ | 795<br>376 ✣ | 316<br>148 ✣ | 493<br>257 ✣ | 336 | 671 | 330 | 415 | 395 | 156 | 246 | 1 |
| Mazdeh et al (2015) | 52 | 26 | 25 | 6 | 6 | 5 | 1 | 4 | 3 | 2 | 5 | 4 | 1 | 5 | 0 | 7 | 5 | 3 | 2 |
| NCT00414726 | 85 | 43 | 41 | 3 | 3 | 5 | 6 | 5 | 7 | 0 | 17 | 2 | 9 | 8 | 10 | 3 | 2 | 7 | 3 |
| Ali et al (2014) | 301 | 126 | 130 | 6 | 10 | 29 | 16 | 21 | 20 | 8 | 22 | 12 | 30 | 16 | 27 | 10 | 14 | 21 | 4 |
| Singhal et al (2005) | 16 | 9 | 7 | 3 | 0 | 4 | 0 | 0 | 2 | 1 | 2 | 0 | 1 | 0 | 0 | 3 | 2 | 1 | 5 |

✣ reported by Chu et al. [111]; [1] figure 2 for mRS at 90 days [139]; [2] table 2 for mRS at six months [140]; [3] [ClinicalTrials.gov identifier: NCT00414726], [Singhal AB et al., unpublished data]; [4] figure 2 for mRS at six months [89]; [5] table patient data [76], and table 1 [143] for mRS at 90 days

mRS, modified Rankin Scale score; OT, oxygen therapy

**Table 9: Tertiary outcomes in randomized trials testing OT in patients with stroke, myocardial infarction and cardiac arrest**

| **Publication,** author (year) | **N** enrolled | **hospital-acquired infection liberal OT,** n | **hospital-acquired infection conservative OT,** n | **hospital-acquired pneumonia liberal OT,** n | **hospital-acquired pneumonia conservative OT,** n | **respiratory failure liberal OT,** n | **respiratory failure conservative OT,** n | **length of hospital stay liberal OT,** days, mean ± SD | **length of hospital stay conservative OT,** days, mean ± SD | **Source of data** |
|---|---|---|---|---|---|---|---|---|---|---|
| **Acute myocardial infarction** | | | | | | | | | | |
| Hofmann et al (2017) ☾ | 6629 | | | | | | | 3.3 § ± 9.6 §§, n = 3311, median 3, range 0 – 68 | 3.4 § ± 13.4 §§, n = 3318, median 3, range 0 – 95 | 1 |
| Stub et al (2012) | 638<br>624 ✣ | 0/218 (sepsis)<br>7/312 ✣ | 1/223 (sepsis)<br>4/312 ✣ | NR<br>2/312 ✣ | NR<br>1/312 ✣ | | | 4.4 § ± 0.7 §§, n = 218, median 4, IQR 4 – 5<br>4 ± 0.7 ✣ | 4 § ± 1.5 §§, n = 223, median 4, IQR 3 – 5 | 2 |
| Rawles et al (1976) | 200 | | | | | | | 15.0 ± 5.7, n = 105 *<br>16.2 ± 5.4, n = 80 ✣ | 14.0 ± 5.6, n = 95 *<br>14.9 ± 5.3, n = 77 ✣ | 3 |
| **Cardiac arrest** | | | | | | | | | | |
| Young et al (2014) | 18 | | | | | | | 5.1 § ± 5.0 §§, n = 9, median 5.4, IQR 2.1 – 7.9<br>6.5 ± 5.8 ✣ | 11.7 § ± 23.5 §§, n = 8, median 4.0, IQR 1.2 – 27.4<br>12.4 ± 14.5 ✣ | 4 |
| **Acute ischemic stroke** | | | | | | | | | | |
| Roffe et al (2017) | 8003<br>5336 ✣ | 793/5267 (antibiotics)<br>400/2668 ✣ | 403/2649 (antibiotics)<br>403/2668 ✣ | | | | | | | 5 |
| Ali et al (2014) | 301 | 27/148 (antibiotics) | 22/141 (antibiotics) | | | | | | | 6 |
| NCT00414726 | 85 | 18/43 | 14/42 | | | 3/43 (respiratory SAE) | 2/42 (respiratory SAE) | 8 ± 4, n = 43 | 7 ± 5, n = 42 | 7 |
| Chiu et al (2006) ☾☾ | 46 | 4/17 (fever) | 15/29 (fever) | 1/17 | 6/29 | 3/17 | 8/29 | 17.2 ± 15.3 §§, n = 16, median 13, range 4 – 58 | 21.1 ± 16.2 §§, n = 22, median 13, range 3 – 65 | 8 |
| Rønning et al (1999) | 550 | | | | | | | 8.6 ± 6, n = 292 | 8.7 ± 7, n = 258 | 9 |

☾ length of hospital stay not analyzed by Chu et al. [111]; ☾☾ study not considered by Chu et al. [111]; ✣ reported by Chu et al. [111]; § estimated mean [144]; §§ estimated standard deviation [145]; * combined mean and standard deviation of patients with and without acute myocardial infarction [136]; [1] table 2 [132]; [2] tables 4 and 2 [115]; [3] tables I and II [136]; [4] supplementary data, tertiary end points [137]; [5] table 2 [139]; [6] results, other outcomes at one week [141]; [7] [ClinicalTrials.gov identifier: NCT00414726], [Singhal et al., unpublished data]; [8] table 2 and results [75]; [9] table 1 [74]

IQR, interquartile range; NR, not reported; OT, oxygen therapy; SAE, serious adverse events; SD, standard deviation

**Table 10: Risk of bias in randomized trials testing OT in patients with stroke, myocardial infarction and cardiac arrest as assessed by Chu et al. [111]**

| Publication, author (year) | Randomization | Allocation concealment | Blinding | Incomplete data | Selective reporting | Other bias | Overall |
|---|---|---|---|---|---|---|---|
| **Acute myocardial infarction** | | | | | | | |
| Hofmann et al (2017) | + | + | + | + | + | + | Low |
| Khoshnood et al (2016) | + | + | + | + | + | + | Low |
| Stub et al (2012) | + | + | + | + * | + | + | Low |
| Ranchord et al (2012) | + | + | + | + | + | + | Low |
| Ukholkina et al (2005) | + | + | + | + | + | + | Low |
| **Wilson et al (1997) ☾** | | | | | | | |
| Rawles et al (1976) | + | + | + | + | + | + | Low |
| **Cardiac arrest** | | | | | | | |
| Young et al (2014) | + | + | + | + | + | + | Low |
| Kuisma et al (2006) | + | + | + | + | + | + | Low |
| **Acute ischemic stroke** | | | | | | | |
| Roffe et al (2017) | + | + | + | + | + | + | Low |
| Shi et al (2017) | + | + | + | + | + | + | Low |
| Mazdeh et al (2015) | + | + | + | + | + | + | Low |
| Ali et al (2014) | + | + | + | + | + | + | Low |
| NCT00414726 | + | + | + | + | + | - † | High |
| Padma et al (2010) | + | + | + | + | + | + | Low |
| **Roffe et al (2010) ☾** | | | | | | | |
| **Chiu et al (2006) ☾** | | | | | | | |
| Singhal et al (2005) | + | + | + | + | + | - †, ✣ | High ✣ |
| Rønning et al (1999) | - | - | + | + | + | + | High |

☾ study not considered by Chu et al. [111]; ✣ annotation and – consequently – the overall rating most likely relates to the unpublished NCT00414726 trial; † Data-driven early termination for either apparent benefit or harm; * For mortality outcomes at 30 days and at longest-follow up, there were 168 missing patients (29% of all patients randomized) for post-randomization exclusion, putting these two outcomes in this study at high risk of bias.

**Table 11: Risk of Bias in randomized trials testing OT in patients with stroke, myocardial infarction and cardiac arrest as assessed by ourselves**

| Publication, author (year) | Randomization | Allocation concealment | Blinding | | Incomplete data | | Selective reporting | Other bias | Overall |
|---|---|---|---|---|---|---|---|---|---|
| | | | Personell | Outcome assessment | Mortality | modified Rankin Score | | | |
| **Acute myocardial infarction** | | | | | | | | | |
| Hofmann et al (2017) | Unrestricted 1:1 | Central randomization | Open | Mortality: SWEDEHEART registry | Deaths reported for all randomized patients | | All patients randomized reported (ITT) | Baseline: no noteworthy differences | Low |
| Khoshnood et al (2016) | Blocked sequences of 3 vs. 3; easily checkable | Full blocks handed out as sealed envelopes; last randomisation in small block can be inferred | Participants | Mortality: SWEDEHEART registry | Deaths reported for all randomized patients | | Baseline characteristics and mortality reported for all patients randomized, primary endpoint analysis limited to 95/160 patients with cardiac MRI | Baseline: diabetes, hypertension, previous stroke/TIA n.s. more in conservative OT group | Low |
| Stub et al (2012) | Computer generated sequence of blocks of size 10 | Sealed envelopes, only three envelopes at once handed out | Open | Mortality at six months | Mortality unknown for 14 and 211 patients at discharge and six months, respectively | | 14 patients retracted consent, six-months mortality unknown for 211/638 patients; primary endpoint (troponin) determined in 405 of 447 or 471 patients with STEMI or any MI, respectively; infarct growth analysis limited to 139/439 patients with cardiac MRI | Baseline: no noteworthy differences | High |
| Ranchord et al (2012) | Computer generated sequence | Full blocks handed out as sealed envelopes | Open | Mortality: open | Mortality unknown for twelve patients incl. two with cardiogenic shock and unknown group allocation ✣ | | Twelve patients excluded after randomization, no outcome data available; infarct mass analysis limited to 71/148 patients with cardiac MRI | Baseline: smokers n.s. more in liberal OT group, and anterior MI in conservative OT group | High |
| Ukholkina et al (2005) | No description of randomization sequence generation | No description of method | Open | Open | Deaths reported for all randomized patients | | All patients randomized reported (ITT) | Unbalanced baseline characteristics: more Killip II classified patients § and longer time-to-intervention times in liberal OT group | High |
| Wilson et al (1997) ☾ | No description of randomization sequence generation | Sealed envelopes | Open | Open | Unknown group allocation of the one patient who died ✣ | | Group allocation for eight excluded patients unknown (four withdrew consent, two incomplete data, one died, one cerebrovascular accident) | Similar age, sex, diabetes and smoking at baseline; no further informations given | Medium |
| Rawles et al (1976) | No description of randomization sequence generation | Sealed envelopes | Double-blind | Double-blind | Deaths reported for all randomized patients | | All randomized patients reported | No table with baseline characteristics | Medium |
| **Cardiac arrest** | | | | | | | | | |
| Young et al (2014) | Blocked sequences of 3 vs. 3; easily checkable | Full blocks handed out as sealed envelopes; last randomisation in small block can be inferred | Participants | Open | Mortality unknown for one patient excluded after randomization | | One patient excluded after randomization due to 'non-eligibility' with no data available | Baseline: n.s. longer response times in liberal OT group | High |
| Kuisma et al (2006) | Patients randomized by ambulance physician; exact procedure unknown | Method not reported | Open | Open | Mortality unknown for four patients incl. two cases with sustained ROSC < 60 min ✣ | | Four patients excluded after randomization (two false randomisation, two with sustained ROSC < 60 min) | Unbalanced baseline characteristics: more pre-hospital IVT in conservative OT group | High |
| **Acute ischemic stroke** | | | | | | | | | |
| Roffe et al (2017) | Stratified 1:1:1 | Central web-based minimized randomization | Open | Mortality and mRS at three months | Mortality unknown for 44, 37 and 326 patients at 7, 30 and 90 days, respectively | Modified Rankin Score at 90 days unknown for 326 patients | Many patients lost to follow-up; mortality and mRS at 90 days unknown for 326 patients | Baseline: no noteworthy differences | High |
| Shi et al (2017) | No description of randomization sequence generation | No description of method | Open | Open | Deaths reported for all randomized patients | | All patients randomized reported (ITT) | Baseline: no noteworthy differences | Low |
| Mazdeh et al (2015) | No description of randomization sequence generation | No description of method | Open | Open | One patient excluded, no data available | One patient excluded, no data available | One patient excluded, no data available | Baseline: no noteworthy differences | Medium |
| Ali et al (2014) | Computer generated sequence | Central phone- (first 153 participants), then central web-based randomization (last 148 participants) | Open | Mortality and mRS at six months | Mortality unknown for 12 and 45 patients until day 90 and at six months, respectively | Six-months mRS unknown for 45 patients | Many patients lost to follow-up; mortality and mRS at 90 days unknown for 45 patients | Baseline: no noteworthy differences | High |
| NCT00414726 | Computer generated sequence | No description of method | Double-blind | Double-blind | Deaths reported for all randomized patients | Missing mRS at 90 days for one/85 patients | All patients randomized reported (ITT) | Unbalanced baseline characteristics: more pre-morbid mRS 3 or 4 (19 vs. 10 %), more LVO (56 vs. 36 %), and more COPD (16 vs. 2%) in liberal OT group | High |
| Padma et al (2010) | Block randomization | Equal allocation | Open | Open | Deaths reported for all randomized patients | | All patients randomized reported (ITT) | Similar NIHSS and modified Rankin Score at baseline; no further informations given | Low |
| Roffe et al (2010) ☾ | Computer generated sequence | No description of method | Participants | Open | Mortality unknown for four patients | | Four patients excluded after randomization (one lost to follow-up, three non-stroke diagnosis) | Unbalanced baseline characteristics: stroke severity and body mass index lower in liberal OT group | High |
| Chiu et al (2006) ☾ | No randomization | No randomization | Open | Open | Deaths reported for all enrolled patients; not an RCT | | All patients enrolled reported; not an RCT | Baseline: no noteworthy differences | High |
| Singhal et al (2005) | No description of randomization sequence generation | Sealed envelopes | Open | Open | Deaths reported for all randomized patients | mRS reported for all randomized patients | All patients randomized reported (ITT) | Baseline: no noteworthy differences | Low |
| Rønning et al (1999) | 'Quasi' randomization | Allocation based on date of birth | Open | Open | Deaths reported for all randomized patients | | All patients randomized reported (ITT) | Baseline: no noteworthy differences | High |
| PROOF study group | Stratified 1:1 | Central randomization | Open | Blinded outcome assessment | Deaths will be reported for all randomized patients | mRS will be reported for all randomized patients | All patients randomized reported (ITT) | Baseline: due to stratification no noteworthy differences are expected with regard to the most relevant baseline characteristics §§ | Low |

☾ study not considered by Chu et al. [111]; ✣ we performed 'best case' and 'worst case' analyses to address this issue; § see [146]; §§ stratification based on: (1) brain imaging modality at baseline (CT vs. MRI), (2) side of LVO (left vs. right), (3) LVO location (terminal ICA vs. proximal M1 vs. distal M1) in conjunction with NIHSS at baseline (6-10 vs. 11-20 vs. ≥ 21), (4) study site

COPD, chronic obstructive pulmonary disease; ICA, internal carotid artery; ITT, intention to treat; IVT, intravenous thrombolysis; LVO, large vessel occlusion; MI, myocardial infarction; MRI, magnetic resonance imaging; mRS, modified Rankin Score; n.s., non-significantly; OT, oxygen therapy; RCT, randomized controlled trial; ROSC, return of spontaneous circulation; STEMI, ST elevation myocardial infarction; TIA, transient ischemic attack

### *4.3.2* Updated Risk-Benefit Assessment Considering the Literature until March 17th, 2021

A PubMed search on March 17th, 2021, using the criteria ("oxygen therapy" OR "normobaric oxygen") AND ("acute ischemic stroke" OR “acute stroke”) revealed 5 new publications since December 6, 2019.

Of these, two were review articles on hyperbaric oxygen therapy [147, 148], and not NBHO which is the treatment that is applied in PROOF. Furthermore, no recent studies were included in the review articles. Consequently, neither review article contributes to the risk-benefit assessment of the PROOF trial.

One experimental study reported NBHO mediated reduction of hyperglycolysis through modulation of the adenosine monophosphate-activated protein kinase signaling pathway, and thereby alleviation of oxidative injury in a rat model of acute ischemic stroke [149].

In line with two previous retrospective studies (N=2643 and N=554, see Section 4.3 Risk-benefit Assessment) [87, 88], one retrospective study on early oxygenation levels in 1479 acute ischemic stroke patients showed association of lower $SpO_2/FiO_2$ levels in the first few ours of admission and mortality, however, no association for high $SpO_2$ (99% to 100%) compared to normal $SpO_2$ (96% to 98%) [150].

Last but not least, in an RCT [151], in which 180 acute ischemic stroke patients who underwent successful recanalization of an anterior circulation LVO within 6 hours of symptom onset were enrolled, and that compared immediate post-reperfusion high-flow oxygen (15 L/min over 6 hours) with routine low-flow oxygen supplementation (3 L/min), NBHO treatment improved functional outcomes (common odds ratio 2.2 [95% CI 1.26 – 3.87] favoring the distribution of global disability scores on the mRS at 90 days), and reduced mortality at 90 days (13.9% absolute difference, rate ratio 0.35 [95% CI 0.13 – 0.93]) as well as infarct volumes as determined by MRI (median infarct volume 9.4 mL vs. 20.5 mL in the control group, beta coefficient -20.24 [95% CI -35.93 – -4.55]). No significant differences were seen in the rate of symptomatic ICH, pneumonia, urinary infection, and seizures between the two treatment groups [151].

Due to copyright restrictions this table is not included. Please refer to figure 2 in Cheng et al. 2021 [151]

**Figure 6: Modified Rankin Scale score in acute ischemic stroke (AIS) patients after vessel recanalization at 90 days.** 0 indicating no symptoms, 1 no clinically significant disability, 2 slight disability, 3 moderate disability, 4 moderately severe disability, 5 severe disability, and 6 death. Significant difference between the NBHO and control groups was noted in the overall distribution of scores (common odds ratio, indicating the odds of improvement of 1 point on the modified Rankin Scale, 2.2; 95% confidence interval, 1.26 to 3.87), favoring NBHO (from [151])

Due to copyright restrictions this table is not included. Please refer to figure 3 in Cheng et al. 2021 [151]

**Figure 7: Infarct volume at 24 hours after vessel recanalization.** Comparison on infarct volume at 24 h after vessel recanalization between NBHO treated patients and patients in the control group, demonstrating a significant reduction of infarct volume determined by MRI in the NBHO group (from [151])

Although this RCT did not test the oxygen treatment approach that is evaluated in PROOF (i.e. penumbral freezing by NBHO until reperfusion), it confirms the results of the few experimental studies that tested NBHO in the post-reperfusion phase [54, 71, 149, 152], strongly mitigates concerns that oxygen might aggravate reperfusion injury when continued after successful reperfusion, e.g. by an – hypothesized but never confirmed [70] – increase of radical oxygen species in the previously ischemic and, thus, particularly vulnerable brain tissue, and, most importantly, underlines the DSMB’s judgment that hemorrhagic infarction which has been observed in 10 (25%) of the first 40 PROOF patients were most likely unrelated to treatment with NBHO. Importantly, the number of PROOF patients who suffered from any kind of hemorrhagic

infarction (10/40) or symptomatic ICH (3/10) is so far within the range that can be expected in the trials' patient population. Anterior circulation ischemic stroke patients who undergo endovascular thrombectomy for treatment of the underlying acute large vessel occlusion represent one of the most severely affected subgroups of ischemic stroke and are at high risk of large infarcts and reperfusion injury. Similar or even higher numbers of ICH have been reported in recent publications, e.g., 31.9% any ICH and 4.4% symptomatic ICH in [153].

A second PubMed search on March 17$^{th}$, 2021, using the criteria ("oxygen therapy" OR "normobaric oxygen") AND ("acute myocardial infarction"), which was conducted in order to gather further data on safety in a patient population with similar age, vascular risk factors and comorbidities, revealed 7 new publications since December 6, 2019, including three publications of relevance that reported results of prospective studies evaluating normobaric hyperoxygenation.

First, the results of the NZOTACS trial which we had already discussed at the very end of Section 4.3.1 [102].

Second, 1-year clinical outcomes of the 100 anterior STEMI patients who were enrolled into the single-arm IC-HOT study and treated with PCI followed by a 60-min infusion of hyperoxygenated blood (760 to 1000 mmHg) into the left main coronary artery better compared to that of propensity score matched controls: both, the composite endpoint of all-cause death, new-onset heart failure, or hospitalization for heart failure (0.0% vs. 12.3%, p = 0.001), as well as each individually were lower in IC-HOT patients [154]. There were no significant differences between groups in the 1-year rates of reinfarction or clinically driven target vessel revascularization [154].

Third, results of the pre-specified subgroup analysis of patients with COPD (n=296) who had been enrolled into the large randomized controlled DETOX-AMI trial (N=6629) and treated with either oxygen or ambient air resemble the main trial results showing no benefit but also support safety of NBHO even in the presence of pulmonary disease [155].

### *4.3.3* Risk-Benefit Assessment of the PROOF trial during the COVID-19 pandemic

Patients who are enrolled in the PROOF trial suffer from acute ischemic stroke and require immediate emergency treatment independently of trial participation. During emergency transport and hospital admission, respective national and hospital regulations are respected in order to minimize the risk of a SARS-CoV-2 transmission. Preventive measures may include wearing medical face masks, face shields/goggle, plastic aprons and gloves as well as the conduct of rapid antigen tests in patients. Another measure to stop the COVID-19 pandemic are SARS-CoV-2 vaccines, which have just been approved in Europe. These vaccines are becoming increasingly available to medical staff and elderly people; however, the vaccination scheme might differ between the countries involved in the PROOF trial.

There is no additional study-specific risk of spreading the SARS-CoV-2 virus in patients randomized into the control group, as these patients solely receive standard care (treatment as usual). Due to higher than usual oxygen flowrates, NBHO might increase the formation of potentially SARS-CoV-2 contaminated aerosol. Thus, wearing FFP2/3 face masks according to national and local regulations during NBHO is strongly recommended to PROOF investigators.

All PROOF study visits but V7 are conducted during the inpatient stay, and a phone interview might replace V7 in-person visit, so that the enrolled patients and the medical staff are not exposed to a greater risk of SARS-CoV-2 infection compared to routine treatment. Besides, participation in the PROOF trial does not interfere with COVID-19 related travel restrictions.

## 4.4 Data and Safety Monitoring Board (DSMB)

**Composition:** The DSMB will consist of 5 independent experts in the field of clinical trials including one coordinator:

- Werner Hacke, University Hospital Heidelberg, Germany. Fields of expertise: acute stroke, thrombolysis.
- Valeria Caso, Universitầ degli Studi di Perugia, Italy. Fields of expertise: stroke neurology including cervical artery dissections and treatment and prevention of stroke in women
- Christine Roffe, Keele University Staffordshire, UK. Fields of expertise: acute stroke, oxygen supplementation in stroke patients, stroke rehabilitation and ethics
- Gerhard Schroth, University Hospital Bern, Switzerland: Fields of expertise: endovascular interventions including mechanical thrombectomy.
- David Petroff, Zentrum für Klinische Studien, University of Leipzig. Fields of expertise: biostatistics.

**Role:** The DSMB ensures the ethical conduct of the trial and protects the rights and welfare of the patients. All deaths, parenchymal hematoma and remote intracranial bleeding (see Appendix 14 for Heidelberg bleeding classification [101]) will be reported expedited to the DSMB. After validation of the expedited reported bleeding events by Eppdata (core imaging laboratory), according patient narratives are prepared in a timely manner by the concerned trial site and provided to the DSMB. In addition, the DSMB will receive periodic blinded and – upon request – un-blinded reports of clinical trial entry, accrual and drop-outs (withdrawals and lost to follow-ups), adverse events and any other relevant data from the clinical trial as well as the Brain Imaging and outcome assessment (WP 5). We anticipate that these reports will be sent at 6-monthly intervals after the first patient entry. In addition, suspected unexpected serious adverse events (SUSARs) will be reported to the DSMB in a timely manner for consideration for their effect on trial continuation. The DSMB will provide independent, competent, and timely review of the data quality and of safety of the clinical trial. The DSMB will have appropriate independence from political, social, institutional, professional, and market influences, as well as vis-à-vis the sponsor. Site-specific amendments may require special treatment.

**Meetings:** The DSMB will meet at 6-monthly intervals via teleconference to review and evaluate the quality of data collected during the clinical trial and assess reports on (serious) adverse events (all SAE, all non-serious AEs with a causal relationship to the IMP, all parenchymal hematoma and remote intracranial bleeding, and cumulated other non-serious AEs) as per the DSMB charter developed for the clinical trial and provide advices to the SC. Special meetings will be held after the recruitment of 20 patients, and during the interim analysis after the accrual of 160 patients. If these meetings don't fit the 6 months schedule, they will be called for as additional sessions. DSMB will review any reported serious and non-serious AEs (cumulated for non-serious AEs with no causal relationship to the IMP) and any drop-outs (withdrawals and lost to follow-ups). They will analyze the potential causal links with IMP and notify frequent and/or unexpected AEs to the centers and the Sponsor. In addition to annual safety reports, safety interim analyses will be provided to the DSMB and the sponsor every 6 months.

When requested, emergency reviews of data for safety– related issues may be requested by the sponsor. After the conclusion of the review, the DSMB will provide recommendations to the SC regarding ongoing scientific and ethical integrity of the clinical trial based on the data reviewed and the progress reports of the clinical trial.

## 4.5 Steering Committee (SC)

The steering committee (SC) is comprised of the coordinating investigator and his supporting co-investigators, clinical experts not directly involved in the clinical trial and the responsible biometrician. The steering committee is responsible for the scientific integrity of the study protocol, the quality of the study conduct as well as for the quality of the final study report. The Steering committee will decide on the recommendations made by the DSMB.

**Composition:** the SC is chaired by the Coordinator and notably comprises all Work Package (WP) leaders
• Holm Graessner (EKUT), leader of WP2 (Coordination and innovation management) (Deputy: Monika Glauch)
• Sven Poli (EKUT), Coordinator leader of WP3 (Trial preparation) (Deputy: Johannes Tünnerhoff)

• Johannes Hüsing (UKL-HD), responsible biometrician (Deputy: Maike Nilsson (UKL-HD), leader of WP4 (Performance of the clinical trial))
• Frosti Palsson (Eppdata), leader of WP5 (Brain Imaging and outcome assessments) (Deputy: Jens Fiehler)
• Joan Montaner (VHIR), leader of WP6 (Biomarkers) (Deputy: Alejandro Bustamante)

**Role:** The SC is in charge of the day-to-day management of PROOF activities. It prepares material to be discussed by the General Assembly and ensures that these decisions are properly implemented while taking account of guidelines and ethical, gender and intellectual property rights (IPR) issues.
The SC is also in charge of the day-to-day progress of the WPs and use of the corresponding human and financial resources. The SC members will report on the progress of the WPs at regular six-monthly meetings and through annual written technical and financial reports. Furthermore, the SC will review the project results (deliverables) and oversee the corresponding IPR. The project management team will provide support to the SC for these tasks.
Decision-making: The SC's decisions are taken by a simple majority vote. Each project partner represented on the SC (i.e. each committee member) will have one vote. In the event of a tied vote, the Coordinator shall have a casting vote.

**Meetings:** The SC will meet twice a year, unless intermediate meetings are in the interest of the project in order to review project progress, consider any risks related to the WPs and, as required, identify solutions and/ or alternative options. The SC will work interactively using a dedicated project intranet and audio-visual tools maintained and provided by the PMT. The PMT will operate as the SC’s secretariat.

# 5 TRIAL OBJECTIVES AND ENDPOINTS

## 5.1 Primary Objective and Primary Endpoint

The main Objective of the PROOF trial is to investigate efficacy and safety of NBHO as a neuroprotective treatment in the early phase of ischemic stroke in a randomized controlled clinical phase IIb trial. The trial is designed as a proof-of-concept study, replicating insights from those preclinical studies in which NBHO showed positive effects: early NBHO initiation, and transient ischemia (i.e. NBHO treatment as an adjunct until timely and successful reperfusion).

**Primary endpoint:** Efficacy of NBHO treatment will be determined by an ITT analysis of ischemic core growth defined as the difference in ischemic core volume (in mL) from baseline to 24 hours; brain tissue not included in CT perfusion (or, in case CT perfusion is not available or of insufficient quality, CT angiography source images) or MR diffusion at baseline will be excluded from lesion volume measurements. Two independent neuroradiologists blinded for treatment allocation will assess ischemic core volume.

## 5.2 Secondary Objectives and Secondary Endpoints

Secondary objectives of the PROOF trial are to show clinical efficacy and safety of NBHO adjunct to standard treatment. The following endpoints will be collected and compared between treatment groups.

**Key secondary efficacy endpoint:** will be the change in NIHSS score from baseline to 24 hours as early neurological improvement was noted in previous trials of NBHO in ischemic stroke and is predictive of long-term outcome.[156] However, assessment at later time points may be compromised through high drop-out rates (e.g. due to decompressive hemicraniectomy or withdrawal of care).

**Secondary clinical efficacy endpoints (NIHSS at V5 and mRS at V7 are conducted by a blinded rater, see 8.5.2 Blinding procedure):**

- Survival at V6 and V7;
- NIHSS at V2, V4*, V5*, V6*, V7; *with and without exclusion of patients still under sedation or anesthesia
- Stroke subtype classification at V6 or V7;
- mRS at V6 and V7;
- Barthel Index, taken at V6 and V7;
- MoCA at V7 (controlled for IQCODE at V5);
- SIS-16 at V7;
- EQ-5D-5L at V7;
- MADRS at V7;
- $PaO_2$ at V3 and V5;

Clinical safety endpoints:

- all-cause death at V6 and V7;
- stroke related death at V6 and V7;
- symptomatic intracranial hemorrhage until V6; as per ECASS III definition and per Heidelberg bleeding classification [101] (see Appendix 14)
- vital signs (systolic and diastolic blood pressure, heart and respiratory rate, $SpO_2$, $etCO_2$) at V1-V7:
- with body temperature at V1, V5 and V6;
- 12-lead ECG at V1 and V5;
- laboratory (blood count, clinical chemistry, coagulation) at V5 and V6;
- Length of ICU* stay, hospital stay, and duration of ventilation at V6 and V7; *ICU is defined as a ward with capacity for mechanical ventilation and/or continuous monitoring of vital parameters (including stroke units).

- concomitant invasive procedures (e.g. intravenous/intra-arterial thrombolysis, thrombectomy, stenting, carotid surgery, decompressive hemicraniectomy, cardioversion, patent foramen ovale (PFO) closure) until V7.

Secondary imaging efficacy endpoints:

- relative changes in ischemic core volume (in %) from baseline to 24 hours;
- absolute and relative ischemic core change from baseline to 24 hours using either NCCT or DWI-MRI (or CT angiography source images and DWI) for ischemic core estimation at baseline;
- absolute and relative ischemic core change from baseline to 24 hours using cerebral blood flow (CBF) < 30% for ischemic core estimation at baseline in all patients, independent of imaging modality;
- penumbral salvage from baseline to 24 hours;
- TICI (Thrombolysis in Cerebral Infarction perfusion scale grade) in patients who underwent mechanical thrombectomy (TBY);
- revascularization on 24-hour follow-up imaging.

Imaging safety endpoints:

- new microbleeds on 24-hour follow-up MRI (vs. baseline T2*w MRI), only possible in patients who had MRI at baseline as well as at 24 hours
- any intracranial hemorrhage on 24-hour follow-up imaging;
- peri-interventional occurrence of vasospasms;
- ischemic lesions in new territories on 24-hour follow-up imaging [157]

Exploratory imaging endpoints: compare Imaging Protocol and Image Interpretation Guidelines

Exploratory biomarker endpoints: compare Biomarker Study Protocol

# 6 TRIAL DESIGN AND DESCRIPTION

## 6.1 Trial design

**Study design:** Prospective, multicenter adaptive phase IIb, parallel group, randomized (1:1), standard treatment-controlled, open-label, clinical trial with blinded endpoint assessment (PROBE design). Randomization will be stratified (see Section 8.5.1 Randomization method).

**Intervention arm:** NBHO (i.e. inhalation of 100% oxygen at high flow (≥ 40 L/min) via a sealed non-rebreather face-mask with reservoir, or in case of intubation/ventilation for (study-independent) TBY, ventilation with an inspiratory oxygen fraction ($FiO_2$) of 1.0) started within 6 hours after certain stroke symptom onset (witnessed) or after symptom recognition (in case of wake-up or unknown onset stroke), and within 30 minutes after end of baseline brain imaging and applied until the end of TBY procedure (defined by removal of guide catheter from sheath) or, in case TBY is not attempted (defined as 'TBY was not attempted or intervention was stopped prior to any penetration or aspiration of the qualifying (i.e. intracranial) LVO'), 4 hours after start of study treatment.

**Control arm:** oxygen supplementation if $SpO_2$ ≤ 94% at 2 to 4 L/min via nasal cannula according to guidelines of the European Stroke Organisation (ESO) [158], or in case of TBY-related intubation/ventilation, ventilation with an initial $FiO_2$ of 0.3 to be gradually increased if $SpO_2$ ≤ 94%.

## 6.2 Trial Duration and Schedule

The duration of the trial for each subject is expected to be three months (from enrolment within 6 hours of acute ischemic stroke (or symptom recognition in case of wake-up or unknown onset stroke) to day 90 follow-up).

The overall duration of the trial is expected to be approximately three years. Recruitment of subjects will start in Q2 2018. The actual overall or recruitment duration may vary. An interim analysis will be conducted following the enrolment of 160 patients. Depending on the results of a re-calculation based on the results of this analysis, the final patient sample size is determined. The study end is defined as "last subject out" (LSO).

| | |
| --- | --- |
| Total trial duration: | 84 months |
| Duration of the clinical phase: | 48 months |
| Beginning of the preparation phase: | Q1 2017 |
| FSI (First Subject In): | August 2019 |
| LSI (Last Subject In): | Q1 2023 |
| LSO (Last Subject Out): | Q2 2023 |
| DBL (Data Base Lock): | Q3 2023 |
| Statistical analyses completed: | Q4 2023 |

# 7 SELECTION OF SUBJECTS AND CENTRES

## 7.1 Number of Subjects

As calculated in Section 12.1 Sample Size Calculation, 456 subjects will be enrolled in the clinical trial, i.e. 228 subjects per treatment group.

An interim analysis is planned after inclusion of 160 patients in the full analysis set. If the study continues after adaptation, the final analysis will be performed after inclusion of up to 296 additional patients, i.e. up to 148 subjects per treatment group.

## 7.2 Centers

The study will be conducted on a multinational and multicenter basis.
Recruitment and treatment of subjects will be performed in clinical trial centers in the following countries: Germany, Belgium, the Czech Republic, Finland, France, Spain and Switzerland.

## 7.3 General Criteria for Subjects' Selection

The PROOF trial will include previously independent acute anterior circulation ischemic stroke patients (due to LVO) within a time window of six hours from symptom onset or, in case of wake-up or unknown onset stroke, symptom recognition to initiation of study treatment.

## 7.4 Inclusion Criteria

Subjects meeting all of the following criteria will be considered for admission to the trial.

- Age: ≥ 18 years
- Acute anterior circulation ischemic stroke due to an LVO on CT or MR angiography, i.e. either **terminal ICA** with **M1/carotid-T**, **proximal M1**, **distal M1** (distal to perforating branches), or **M2/3 segment(s)**
- **If TBY is likely to be conducted*** (**However, neither TBY nor IVT are a prerequisite for inclusion; patients not receiving TBY or IVT or both can be enrolled. Clinical treatment decisions should not delay study enrollment*).
- NIHSS score of ≥ 6 at screening
- ASPECTS of 6-10 on NCCT or 5-10 on DWI-MRI
- If recommended by the attending physician, CT or MR perfusion should be performed prior to NBHO
- NBHO can be initiated within 6 hours of symptom onset (witnessed) or symptom recognition (in case of wake-up or unknown onset stroke), and within 30 minutes after last image of baseline brain imaging
- Pre-stroke mRS of 0 to 2
- Breastfeeding women must stop breastfeeding after randomization
- Own written informed consent is not obtained prior to study inclusion but has to be gained as soon as possible. Patients who are able to give consent will be informed about trial participation orally and may consent to or decline participation. Patients unable to give consent will be enrolled through a deferred consent procedure (see Section 14.5 Subject Information and Informed Consent)

## 7.5 Exclusion Criteria

Subjects presenting with any of the following criteria will not be included in the trial:

<u>Neurological:</u>

- TBY procedure initiated (groin puncture) prior to randomization
- Rapid major improvement in neurological status* prior to randomization (**in case but NIHSS score remains ≥ 6, enrolment might still be possible if persistent LVO is confirmed on repeated vessel imaging*)
- Any condition which precludes obtaining an accurate baseline NIHSS or outcome assessment (e.g. seizures, dementia, psychiatric or neuromuscular disease)
- Intracranial hemorrhage (except of cerebral microbleeds), intracranial tumor (except small meningioma), and/or intracranial arteriovenous malformation
- Intracranial aneurysm or prior stent implantation in the vascular territory (upstream and downstream) affected by qualifying LVO

- Suspected complete CCA occlusion, aortic dissection, cerebral vasculitis, septic embolism, or bacterial endocarditis
- Acute bilateral stroke or stroke in multiple vascular territories (except of clinically silent lesions)

Respiratory:
- Acute or chronic pulmonary disease or respiratory distress that may, in the clinical judgement of the investigator, interfere with the study intervention (e.g. acute pneumonia, COPD flare-up etc.)
- Prior to enrolment, > 2 L/min oxygen required to maintain peripheral oxygen saturation ≥ 95%
-

Other:
- Clinical suspicion of acute myocardial infarction (e.g. acute chest pain)
- Baseline blood glucose of < 50 mg/dL (2.78 mmol) or > 400 mg/dL (22.20 mmol)
- Body temperature ≥ 38.0°C at screening
- History of severe allergy (more than rash) to contrast medium
- Current treatment with nitrofurantoin or amiodaron, paraquat poisoning, or history of treatment with bleomycin
- Pregnancy at screening, to be excluded (β-HCG in serum or urine) in all women ≤ 55 years except if surgically sterile; in women >55 years pregnancy must be excluded only in case of increased probability e.g. due to in-vitro fertilization
- Any co-existing or terminal disease (except qualifying stroke) with anticipated life expectancy of less than 6 months
- Any pre-existing condition that may, in the clinical judgment of the investigator, not allow safe participation in the study (e.g. alcohol or substance abuse, co-existing disease)
- Participation in another interventional (drug or device) study within the last four weeks
- Prior participation in the PROOF trial

## 7.6 Pre-specified Measures in Case of Slow Recruitment

To ensure enrolment, in the amended protocol version 1.3, we widened the therapeutic time window for NBHO to six hours, skipped the upper age limit, and allowed more distal and also tandem arterial occlusions in case the PROOF candidate is likely to receive TBY treatment.
To further enhance recruitment, in the amended protocol version 1.4, we additionally allow inclusion of, first, patients with an mRS of 2, second, patients with an ASPECTS of 6 on non-contrast CT or 5 on DWI, third, patients in whom CT perfusion is not conducted, and, fourth, patients with wake-up or unknown onset stroke. First and second adaption had already been pre-specified in protocol version 1.3 ("In case of continued slow recruitment, the following inclusion criteria may also be adapted: (1) pre-stroke mRS may include 0-2, and (2) ASPECTS may be opened for e.g. 6-10 on non-contrast CT or 5-10 on DWI.") and – together with third and fourth adaption – follow the recommendations of the most recent international TBY guidelines [159, 160], and, thus, current TBY practice. Accordingly, future guideline recommendations may (as yet unknown) make further adaptions of the study protocol necessary in future in order to continuously allow best possible recruitment and generalizability of study results by enrolling a patient population that is most representative for current clinical/TBY practice.
The decision for adaption will be reviewed by the IEAB and the SAB, and suggested to the General Assembly by the Steering Committee. In case of approval by the General Assembly, the protocol will be amended accordingly and submitted to the Ethics Committees and Competent Authorities for approval (see Section 14.3). In order to facilitate sensible protocol amendments, a pre-screening log will be maintained by the participating study centers (compare Section 9.1 Pre-Screening).

## 7.7 Criteria for Withdrawal from the Trial or Discontinuation of Treatment

### *7.7.1* Withdrawal of Patients from the Trial

Any patient can withdraw from the trial at any time without personal disadvantages and without having to give a reason. Patients can either withdraw (or be withdrawn by their LAR) from the treatment, from follow-up, or both. Patients or their LAR have to give informed consent as soon as possible after having been included in the trial. In case the patient is not able to give consent, the appointment of an LAR has to be initiated within 72 hours after randomization. In case the patient has been included in the trial by deferred consent procedure (see Section 14.5), and the patient (recovered ability to consent) or LAR does not give

consent, the patient has to be withdrawn from the trial. In this case, the patient or LAR shall be asked whether he/she allows the use of data acquired so far. In case the patient has been included by deferred consent procedure but dies before installation of an LAR, the patient's relatives (or if applicable the designated (planned) LAR) should be asked whether the data acquired so far might be used. Furthermore, already drawn biomarker samples have to be discarded, if the patient / LAR declines to participate in the biomarker sub-study.
The time of withdrawal from the trial must be documented in the patient file and on the CRF and sponsor as well as Coordinating Investigator and DSMB must be informed in written form.

In all patients who leave the study prematurely, a withdrawal examination should be carried out. The patient must be asked to consent to this last examination. The withdrawal examination must be documented in the CRF.
If a patient does not attend a visit, the reason should be clarified. If the patient wants to withdraw, the reason should be documented in the patients file and in the CRF, as long as the patient is willing to state it.
For documentation of AE and SAEs see Sections 11.2 Period of Observation and Documentation and 11.1.7 Relationship and Outcome of AEs.

No patients may be removed from the study by the investigator. For the intention-to-treat analyses (ITT), data from patients randomized into the NBHO arm of the trial will be analyzed as treated with NBHO, even if oxygen therapy is altered or discontinued, and even if the patient experienced intolerable adverse events due to NBHO.

### *7.7.2* Discontinuation of Trial Treatment

The investigator can also discontinue trial treatment (NBHO) after considering the risk-to-benefit ratio, if he/she no longer considers the further treatment of the patient according to study protocol justifiable. The date of and the primary reason for the withdrawal, as well as the observations available at the time of discontinuation of trial treatment are to be documented on the CRF. Reasons leading to the discontinuation of trial treatment can include the following (**<u>one</u> primary reason must be determined**):

- **Lack of patient's cooperation** regarding NBHO
  If the patient does not tolerate NBHO treatment via high-flow oxygen through a sealed non-rebreather mask with reservoir, the investigator must offer alternative means of keeping blood oxygen levels as high as possible. The method of oxygen therapy continuation and measures to increase patients' comfort are at the respective investigator's discretion.
  The investigator should attempt to evaluate if the issue originates from the high flow-rate or irritation by the face-mask itself.
  Initially, the investigator should slightly reduce oxygen flow-rate (by 5-10 L/min) and re-assess the patient's compliance under these conditions. Prior to switching the method of oxygen application, oxygen flow-rate should be further reduced stepwise to as low as < 10 L/min but not < 6 L/min. Please note that this may constitute a protocol violation (compare Section 8.4.3 Compliance), therefore, a re-increase in flow-rate should be attempted as tolerated.
  If reduction of oxygen flow-rate does not lead to a significant improvement of compliance, oxygen flow-rate should be re-increased to ≥ 40 L/min and the rubber valves of the non-rebreather mask should be removed, which will transform the mask into a partial rebreather mask. This may sufficiently increase the patient's breathing comfort whilst avoiding a huge drop in inspiratory oxygen concentration. (Please note that this may constitute a protocol violation! - compare Section 8.4.3 Compliance)
  In case removal of the rubber valves does not lead to a sufficient improvement of compliance, the investigator should switch to a simple face-mask. When switching to a different mask-type, oxygen should be restarted with the maximum flow-rate of ≥ 40 L/min and then decreased stepwise until tolerated by the patient. (Please note that this may constitute a protocol violation! - compare Section 8.4.3 Compliance)
  If the compliance of the patient is only improved by reduction of oxygen flow-rate, a sealed non-rebreather mask with reservoir should be used with the highest tolerated flow-rate.
  Technique (i.e. type of mask), oxygen flow-rate, duration (exact time of start and ending), and $SpO_2$ must be documented in the eCRF.

- **Intolerable adverse events** due to NBHO
  In case the respective investigator deems the continuation of NBHO unsafe due to adverse events caused by NBHO, he may discontinue study treatment. Date, time and reason for discontinuation must be documented in the CRF. If oxygen therapy may be continued at lower flow-rates, this should be attempted.

**Table 12:** Definition of screening-failures, drop-outs, protocol deviations

| **Term** | **Definition** | **Comment/ Sample** |
| --- | --- | --- |
| Screening-failure | Non-eligibility determined during screening:<br>Patient will be recorded in the pre-screening log (ISF and pre-screening log section in the eCRF). | - |
| Drop-out | Participation terminated completely, including follow-up<br>Possible reasons:<br>• Patient withdraws consent **(withdrawal)**<br>• Patient moved/cannot be contacted<br>• Follow-up-interventions cannot be performed due to medical reasons or non-compliance of patient<br>Drop-out after completion of study intervention: **Lost to follow-up**<br>Final examination will be performed, if patient agrees. Study-Completion / Withdrawal-form will be completed. | ITT-sample<br>PP-sample (if study intervention completed) |
| Discontinuation of study intervention | Discontinuation of study intervention, follow-up as per protocol. | ITT |
| Protocol deviation | Major deviations:<br>If exclusion criteria become evident after enrollment, and safety of the participant is affected, or if the diagnosis does not any more relate to the indication listed in the protocol affecting the benefit/risk negatively, the study intervention has to be discontinued. FU-examinations should still be performed.<br>Study treatment is not performed as per protocol (see 8.4.3 Compliance) or the FU-imaging for primary endpoint assessment (V5) is performed outside 18 to 72-hours window.<br><br>Minor deviations:<br>Other protocol deviations (errors in timing of visits/ missing samples/ missing examinations) do not result in discontinuation of study intervention | ITT (assessment of protocol deviations by LKP/ PI together with BM)<br><br>PP-analysis (if minor deviations) |

### *7.7.3* Premature Closure of the Clinical Trial or a Site

If new information on the risk-to-benefit ratio of the drug or on the treatment methods used in the study is obtained in the meantime and safety concerns arise, the sponsor reserves the right to interrupt or terminate the project. Premature termination is also possible if the sponsor notices and agrees upon that patient recruitment is insufficient and that this cannot be expedited by appropriate measures (compare Sections 7.6 Pre-specified Measures in Case of Slow Recruitment and 9.1 Pre-Screening).
Premature termination of a single center is also possible if the sponsor notices that the conduction of the trial is not compliant with ICH-GCP and / or is not according to the protocol, the patient recruitment and / or the quality of the data is insufficient.

The DSMB can recommend interruption or termination of the study based on, first, the results of a tight case by case follow-up evaluation of patients' mortality, intracranial bleedings, and early neurological effects (see Section 4.3.1 on page 40 second last and last paragraph), second, the results of the intermittent SAE evaluation or, third, of accumulating information on the above-mentioned reasons.
The ethics committees (EC) and the competent authorities must be informed about the premature closure of the trial or one of the treatment arms.
All involved investigators have to be informed immediately about a cessation / suspension of the trial. The decision is binding to all trial centers and investigators.

# 8 INVESTIGATIONAL MEDICINAL PRODUCT (IMP)

## 8.1 Study medication

### *8.1.1* General Information

Oxygen used for medical purposes is a diatomic gas applied via the natural or an artificial airway in concentrations between 21% (as in atmospheric air) and 100% depending on the type and severity of the disorder that necessitates oxygen supplementation. It is used for prevention or treatment of acute or chronic hypoxia or hypoxemia as it may occur in general anesthesia, primary or secondary respiratory failure, cardiovascular disease, anemia, hemolysis or impairment of cellular oxygen-dependent metabolism like shock or cyanide/carbon monoxide intoxication.
Due to its ubiquitous use, the oxygen used in the PROOF trial will be available at each trial site (independent of the study).

### *8.1.2* Characterization of study medication

| | |
|---|---|
| Proprietary name: | N/A |
| International Non-proprietary Name (INN): | Oxygen (for medical use) |
| ATC code, if officially registered: | V03AN01 |
| Manufacturer: | various |
| Pharmaceutical formulation: | $O_2$ |
| Mode of administration: | inhalation |
| Batch no.: | N/A |
| Storage instructions: | depending on respective trial site |
| Placebo: | N/A |
| Comparator: | N/A |

## 8.2 Packaging and Labeling

N/A

## 8.3 Supplies and Drug Accountability

N/A

## 8.4 Administration of study medication

### *8.4.1* Assignment of Identification Codes

All patients who seem suitable for study participation and take part in the screening, will receive a screening number. At the end of the screening phase the eligibility of the patient is assessed finally.
When the patient is included in the study (all inclusion criteria fit and none of the exclusion criteria), he/she will be given a patient ID consisting of a three-digit code of the study site followed by a consecutive randomization number. In case of a reassignment of an ID due to misspecification, the randomization service may be used again for the same patient (with the outcome of randomization pre-specified). Patients withdrawn from the study retain their number. New patients must always be allocated a new screening/randomization number.
For allocation to a treatment arm (randomization) see Section 9.3 Randomization.

### *8.4.2* Dosage Schedule

**Intervention arm:** NBHO (i.e. inhalation of 100% oxygen at high flow (≥ 40 L/min) via a sealed non-rebreather face-mask with reservoir, or in case of intubation/ventilation for (study-independent) TBY, ventilation with an inspiratory oxygen fraction ($FiO_2$) of 1.0) started within 6 hours after certain stroke symptom onset (witnessed) or after symptom recognition (in case of wake-up or unknown onset stroke), and within 30 minutes after end of baseline brain imaging and applied until the end of TBY procedure (defined by removal of guide catheter from sheath) or, in case TBY is not attempted (defined as ‘TBY was

not attempted or intervention was stopped prior to any penetration or aspiration of the qualifying (i.e. intracranial) LVO'), 4 hours after start of study treatment.

If the patient does not tolerate NBHO treatment via high-flow oxygen through a sealed non-rebreather mask with reservoir, the investigator must offer alternative means of keeping blood oxygen levels as high as possible (compare Section 7.7.1 Withdrawal of Patients from Treatment). However, technique (i.e. type of mask), oxygen flow-rate, duration (exact time of start and ending), and $SpO_2$ must be documented in the CRF.

<u>Maximum duration of NBHO treatment:</u> 4 hours

<u>Maximum dose allowed:</u> N/A (NBHO is defined as inhalation of 100% oxygen at high flow (≥ 40 L/min) via a sealed non-rebreather face-mask with reservoir, or in case of intubation/ventilation for (study-independent) TBY, ventilation with an inspiratory oxygen fraction ($FiO_2$) of 1.0)

**Control arm:** Oxygen supplementation if $SpO_2 \leq 94\%$ at 2 to 4 L/min via nasal cannula according to guidelines of the European Stroke Organisation (ESO).[158]
In case of TBY-related intubation/ventilation, start ventilation with an initial $FiO_2$ of 0.3 to be gradually increased if $SpO_2 \leq 94\%$.

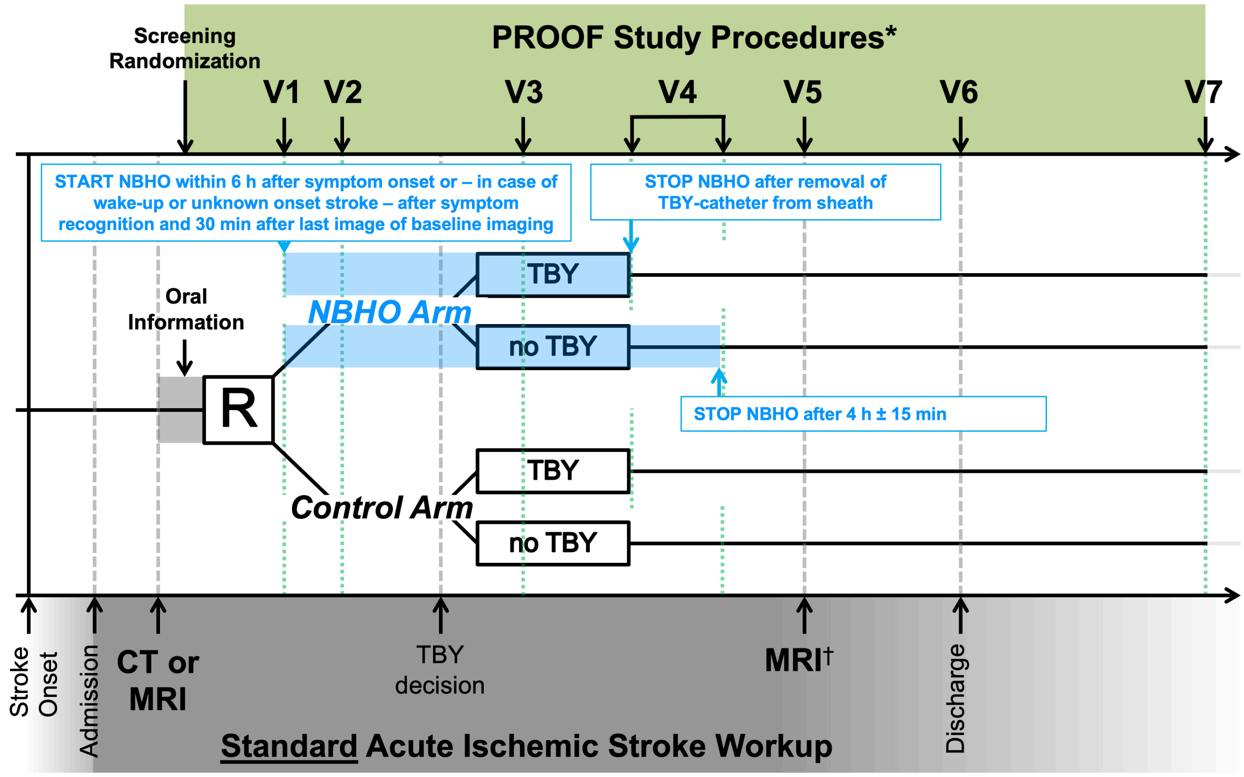


V1-7 = study visits 1-7, NBHO = normobaric hyperoxygenation (study treatment), TBY = endovascular mechanical thrombectomy, CT = computed tomography, MRI = magnetic resonance imaging
* study treatment consists of NBHO for up to 4 h ± 15 min. No further study-dependent treatment is applied. Section 9 provides a detailed description of all study procedures.
** standard acute ischemic stroke workup consists of acute and post-acute diagnostic measures (incl. CT/MR-imaging with perfusion studies at baseline and 24 h follow-up imaging) and acute and post-acute therapy (incl. intravenous thrombolysis and thrombectomy) as well as stroke unit care according to current therapeutic guidelines.
*** 24 h follow-up imaging is deemed standard of care. In PROOF, an MRI should be performed. Only to be replaced by CT if a patient cannot undergo an MRI (e.g. due to a cardiac pacemaker)
**Blue bars indicate start, duration and end of NBHO (study treatment). Note that it is only applied in the intervention arm of the trial and that the end of NBHO depends on whether the patient is undergoes (study-independent) TBY or not.**

**Figure 8:** Study Flow-Chart

### *8.4.3* Compliance

Compliance will be recorded by the treating investigator. Oxygen administration (reason, flow-rate or $FiO_2$ in ventilated patients, and mask-type/method) will be continuously recorded until end of hour 24, i.e. every change of oxygen administration must be documented. Additionally, $SpO_2$, and – if available – $etCO_2$ must

be documented at each time point of change of oxygen administration. The results will be systematically documented in the patient's medical record and in the eCRF.

The following periods and doses will count as protocol violations and lead to exclusion from per-protocol analyses in the NBHO group if administered until the end of TBY procedure (defined by removal of guide catheter from sheath) or, in case TBY is not attempted (defined as 'TBY was not attempted or intervention was stopped prior to any penetration or aspiration of the qualifying (i.e. intracranial) LVO'), during the first 4 hours after start of study treatment:

Non-rebreather face-mask with reservoir:
- > 15-30 L/min for more than one hour
- > 10-15 L/min for more than 30 minutes
- ≤ 10 L/min for more than 15 minutes

Simple face-mask (without reservoir) or (partial) rebreather face-mask with reservoir:
- > 20 L/min for more than one hour
- > 10-20 L/min for more than 30 minutes
- ≤ 10 L/min for more than 15 minutes

Nasal cannula
- any flow-rate for more than 15 minutes

in case of intubated patients:
- $0.8 \geq FiO_2 > 0.6$ for more than one hour
- $0.6 \geq FiO_2 > 0.4$ for more than 30 minutes
- $0.4 \geq FiO_2 > 0.2$ for more than 15 minutes

Additionally, prolongation of NBHO for more than 1 hour after end of TBY procedure (defined by removal of guide catheter from sheath) or, in case TBY is not attempted (defined as 'TBY was not attempted or intervention stopped prior to any penetration or aspiration of the qualifying (i.e. intracranial) LVO'), NBHO administration time in total for more than 5 hours, will equally count as protocol violation and lead to exclusion from per-protocol analyses in the NBHO group. The coordinating investigator and the respective national coordinator ensure that local study teams are appropriately trained to stop NBHO as soon as possible after the end of TBY procedure (defined by removal of guide catheter from sheath).

Conclusion: The per-protocol definition of NBHO is thus the use of either > 30 L/min via non-rebreather face-mask with reservoir or – in case of intubation – an $FiO_2$ of > 0.8 until either the end of the TBY procedure plus max. 1 hour or, in case TBY is not attempted (defined as 'TBY was not attempted or intervention was stopped prior to any penetration or aspiration of the qualifying (i.e. intracranial) LVO'), for a maximal duration of 5 hours.

In the control group, patients will be excluded from per-protocol analyses if oxygen supplementation until the end of TBY procedure (defined by removal of guide catheter from sheath) or, in case TBY is not attempted (defined as 'TBY was not attempted or intervention was stopped prior to any penetration or aspiration of the qualifying (i.e. intracranial) LVO'), during the first 4 hours after randomization is applied at the following flow-rates or – in case of intubation – $FiO_2$:

Any face-mask or nasal cannula
- ≥ 4 to < 6 L/min for more than one hour
- ≥ 6 to < 8 L/min for more than 30 minutes
- ≥ 8 L/min for more than 15 minutes

in case of intubated patients:
- $0.4 \leq FiO_2 < 0.6$ for more than one hour
- $0.6 \leq FiO_2 < 0.8$ for more than 30 minutes
- $0.8 \leq FiO_2 \leq 1.0$ for more than 15 minutes

#### *8.4.4* Prior and Concomitant Diseases

Relevant additional diseases present at the time of trial inclusion are regarded as concomitant diseases and will be documented on the appropriate pages of the eCRF. Included are conditions that are seasonal, cyclic, or intermittent (e.g. seasonal allergies; intermittent headache).

Abnormalities, which appear for the first time or worsen (intensity, frequency) during the trial are adverse events (AEs) and must be documented on the appropriate pages of the eCRF.

#### *8.4.5* Prior and Concomitant Medication

The treatment of accompanying illnesses is permissible unless this interferes with the trial medication, which can increase the risk of adverse events.

In particular, the following drug groups are **not permitted** as concomitant medication:

- During NBHO, administration of bleomycin is contraindicated.
- Nitrofurantoin is contraindicated during application of NBHO and the following week; it is easily replaced by other antibiotics.

Application of amiodaron is **permitted under the following restrictions:**

- During NBHO and the following week, amiodaron should only be administered if the treatment is considered to be of vital importance. If amiodaron is administered during study intervention, NBHO should be discontinued to avoid pulmonary AEs.

If concomitant drugs are administered, these must be recorded in the patient file and in the eCRF, stating

- The type (preferably the generic name / INN, or trade name)
- The route of administration
- The regimen including: dosage schedule (in case of continuous dosage: total dose and duration of application), daily dose (if not indicated by the type), and form of application
- The indication
- The duration (start / stop)

#### *8.4.6* Adjustments to dosage of the IMP in the individual trial subject

No a priori dosage adjustments should be made in any trial subject receiving NBHO (see Section 8.4.2 Dosage Schedule).

### 8.5 Randomization and Blinding

#### *8.5.1* Randomization method

Patients will be randomized to a treatment arm in a 1:1 ratio. The randomization procedure will be provided by a web-based service http://randomizer.at with study-specific roles assigned to study personnel. It ensures allocation concealment in that it requires the entry of the stratification parameters before disclosing the result of the assignment. A study-specific manual will be prepared before initiation of the study.

Minimization [161] will be used to consider several strata when allocating treatment. In 10 per cent randomly chosen cases, the procedure will pick the treatment not assigned by the algorithm. Variables used in minimization will be:

- brain imaging modality at baseline (CT vs. MRI)
- side of large vessel occlusion (LVO) (left vs. right)
- intracranial LVO location (terminal internal carotid artery (ICA) with involvement of the M1-segment of the middle cerebral artery (MCA)/carotid-T vs. proximal M1-segment vs. distal M1-segment (distal of perforating branches) vs. M2/3-segment(s)
- NIHSS at baseline: 6-10, 11-20, 21 and more. LVO location and NIHSS will be used in conjunction for the algorithm, i.e. balance will be aimed for in every one of the nine NIHSS/LVO locations.
- Time window known < 6h vs. unknown/wake-up
- study site

### *8.5.2* Blinding procedure

Staff that is involved in the emergency treatment of patients will not be blinded to the treatment allocation as blinding would only be possible by comparing NBHO to high-flow air, which does not represent standard stroke treatment (compare [158]) and, by itself, is known to exert clinically relevant effects on respiration and ventilation.[162, 163]

Outcome-raters at the image core laboratory (i.e. Eppdata) will be blinded to the respective treatment as they do not receive any information about randomization and the prior clinical course. The image core lab will implement further measures to avoid bias (e.g. random presentation of baseline and follow-up images of different patients).

Raters of the NIHSS at V5 (24 hours) and the mRS at V7 (Day 90) should be blinded to the respective treatment and chosen by the investigator in a way that no outcome-rater was involved in the emergency treatment of a respective patient. Raters must be trained and certified for NIHSS and mRS.

### *8.5.3* Unblinding

Due to the nature of treatment, no unblinding is necessary during the treatment phase. The blinded outcome-raters will not be given a method of unblinding.

### *8.5.4* Emergency Treatment

During and following a subject's participation in the trial, the investigator should ensure that adequate medical care is provided to a subject for any AE including clinically significant laboratory values. The investigator should inform a subject when medical care is needed for intercurrent illness(es) of which the investigator becomes aware.

# 9 DESCRIPTION OF TRIAL VISITS

## 9.1 Pre-Screening

The participating study sites will maintain a pre-screening log. All patients with acute anterior circulation stroke due to LVO that are treated with TBY at the respective study center but not enrolled into the PROOF study will be entered anonymously. In addition to a consecutive pre-screening number, age, ASPECTS, pre-stroke mRS, NIHSS, intracranial LVO location, presence and cause of an extracranial ICA occlusion or high-grade stenosis, and the reason(s) for non-inclusion will be documented. The pre-screening logs of the participating study centers will be monitored regularly and reviewed by the SC in order to help implement protocol amendments in case of slow recruitment (compare Section 7.6 Pre-specified Measures in Case of Slow Recruitment). In order to support this monitoring process, the respective study site personnel will transfer pre-screening logs to the eCRF and provide the total absolute number of TBY-treated ischemic stroke patients extracted from the local patient information system or TBY database during site monitoring.

## 9.2 Screening Visit

At Screening (which occurs parallel to acute diagnostics and study-independent treatment of the index stroke), demographics and medical history incl. concomitant medication and invasive procedures will be assessed, physical and neurological examination (incl. NIHSS) as well as brain imaging incl. perfusion studies will be performed as per clinical routine.
Patients entering the screening phase will receive a unique screening number for pseudonymization. If a patient fulfills all inclusion and no exclusion criteria, he/she may be randomized and treated accordingly. Informed consent will be obtained as soon as possible according to the procedure described in Section 14.5 Subject Information and Informed Consent.

For safety reasons, vital signs (incl. systolic and diastolic blood pressure, heart rate and respiratory rate, $SpO_2$, $etCO_2$, and tympanic temperature), blood samples incl. full blood count (white blood cells, platelet count, erythrocytes, hemoglobin, hematocrit), coagulation (international normalized ratio (INR), activated partial thromboplastin time (aPTT), D-dimers), and blood chemistry (sodium, potassium, creatinine, urea, uric acid, total bilirubin, direct bilirubin, total protein, albumin, C-reactive protein, troponin I or T, brain natriuretic peptide (BNP) or N-terminal prohormone of brain natriuretic peptide (NT-proBNP), creatine kinase (CK), aspartate transaminase (AST), alanine transaminase (ALT), alkaline phosphatase, lactate dehydrogenase (LDH), gamma-glutamyl transpeptidase (GGT), thyroid-stimulating hormone (TSH), and glucose), and – if applicable (see Section 7.5 Exclusion criteria) – serum/urine pregnancy test must be assessed.
All documented changes of pre-randomization $O_2$ administration (incl. pre-hospital) are documented incl. reason, flow-rate, mask-type, duration, $SpO_2$, and – if available – $etCO_2$.
In patients who receive $O_2$ supplementation at screening for unclear reasons, which is not uncommon in the emergency setting of acute stroke, the investigator may re-assess the indication for $O_2$ administration at flow-rates > 2 L/min by reduction or discontinuation of $O_2$ flow. If the patient is able to maintain an $SpO_2$ of ≥ 95% at $O_2$ flow-rates of ≤ 2 L/min, he or she may be randomized given all other eligibility criteria are met.
At screening, a first batch of blood samples for biomarker determination (i.e. two gel serum separation Vacutest® tubes of 5 mL (Yellow cap) and two plasma EDTA K2 Vacutest® tubes of 4 mL (Lavender caps)) is taken (as part of the PROOF biomarker sub-study, see Section 10.19; total blood amount: 18 mL). Blood for biomarkers should be drawn only from patients with study-independent venous or arterial access. In case, the patient / LAR does not give informed consent (see Section 14.5 Subject Information and Informed Consent for details), blood samples for biomarker assessment will be discarded.

## 9.3 Randomization

Eligibility is confirmed by the investigator immediately after brain imaging. Subjects that fulfill all inclusion criteria and none of the exclusion criteria will be randomized using a web-based service (http://randomizer.at). Criteria to be used for minimization are referred in Section 8.5.1 Randomization method. The information “study site” will be provided automatically by the investigator’s individual login. Other information incl. the screening number must be provided by the investigator.

## 9.4 V1 – Initiation of study treatment

In case the patient is randomized into the intervention arm, NBHO must be started within 6 hours after certain stroke symptom onset (witnessed) or after symptom recognition (in case of wake-up or unknown onset stroke), and within 30 minutes after end of baseline brain imaging; see Section 8.4.2 Dosage Schedule for details.
Patients that are randomized to standard treatment will receive either no $O_2$ supplementation (if $SpO_2$ ≥ 95%) or low-flow $O_2$ supplementation (if $SpO_2$ ≤ 94%) to maintain $SpO_2$ ≥ 95% according to ESO guidelines. In case of TBY-related intubation/ventilation, the initial $FiO_2$ of 0.3 may be gradually increased if $SpO_2$ ≤ 94%; see Section 8.4.2 Dosage Schedule for details.
A 12-lead electrocardiogram is performed either prior to initiation of study treatment or within six hours after start of NBHO.
Vital signs (incl. systolic and diastolic blood pressure, heart rate and respiratory rate, $SpO_2$, and – if available – $etCO_2$) are recorded every 30 ±10 minutes for the first six hours after start of NBHO (or randomization in the control arm).
Oxygen administration (reason, flow-rate or $FiO_2$ in ventilated patients, and mask-type/method) will be continuously recorded until end of hour 24, i.e. every change of oxygen administration must be documented. Additionally, $SpO_2$, and – if available – $etCO_2$ must be documented at each time point of change of oxygen administration.
Concomitant medication, invasive procedures and AE/SAE will be reported continuously.

## 9.5 V2 – ≥ 5 minutes after start of NBHO (or randomization in the control arm) and before start of endovascular intervention

≥ 5 minutes after initiation of NBHO (or randomization in the control arm) and before start of endovascular intervention including sedation and/or endotracheal intubation/mechanical ventilation, NIHSS assessment is repeated in order to detect early improvement (or deterioration) likely associated with study treatment. The shortened NIHSS for emergency medical services (sNIHSS-EMS [1]) may be chosen over full NIHSS assessment to speed up V2 assessment. V2 assessment should not be conducted if it interferes with routine stroke management.
If the patient is already sedated or under general anesthesia for the thrombectomy procedure, this must be recorded in the eCRF.
Vital signs as well as $O_2$ supplementation are recorded as indicated in Section 10.2 Vital signs and Section 10.15 Oxygen administration.
Concomitant medication, invasive procedures and AE/SAE will be reported continuously.

## 9.6 V3 – During TBY

V3 is to be performed during NBHO or control treatment, respectively, either during TBY procedure (i.e. time from groin puncture to removal of guide catheter from sheath) or, in case TBY is not attempted, 90 ±30 minutes after randomization.
Arterial blood gas analysis (incl. pH, $PaO_2$, $SaO_2$, $PaCO_2$, actual $HCO_3^-$, and actual base excess) is performed with blood drawn from patients with study-independent arterial access (e.g. for TBY procedure).
Vital signs as well as $O_2$ supplementation are recorded as indicated in Section 10.2 Vital signs and Section 10.15 Oxygen administration.
Concomitant medication, invasive procedures and AE/SAE will be reported continuously.

## 9.7 V4 – End of study treatment

V4 is to be performed within 60 min after end of TBY procedure (defined by removal of guide catheter from sheath) or, in case TBY is not attempted (defined as 'TBY was not attempted or intervention was stopped prior to any penetration or aspiration of the qualifying (i.e. intracranial) LVO'), 4 hours ±15 minutes after start of NBHO (or time of randomization for the control group), or earlier, in case study treatment is prematurely terminated.
Physical and neurological examination (incl. NIHSS) is repeated.
Vital signs as well as $O_2$ supplementation are recorded as indicated in Section 10.2 Vital signs and Section 10.15 Oxygen administration.
Concomitant medication, invasive procedures and AE/SAE will be reported continuously.

## 9.8 V5 – 24 ±6 hours (Day 1) after start of NBHO (or randomization in the control arm)

NIHSS assessments at V5 should be performed by an investigator blinded to study treatment.
At day one after start of NBHO (or randomization in the control arm), the patient receives a first follow-up visit. For determination of the primary outcome parameter (infarct core volume assessment in between 18 and 72 hours after baseline imaging), MRI follow-up brain imaging must be performed including DWI, ADC, FLAIR, T2*, TOF angiography as minimal standard. If MRI is not feasible in between 18 and 72 hours, which is defined as minor protocol deviation, MRI is to be performed the soonest possible until discharge.

For determination of the key secondary endpoint, physical and neurological examination (incl. NIHSS) are repeated, tympanic temperature is measured.
For safety reasons, blood samples incl. full blood count (white blood cells, platelet count, erythrocytes, hemoglobin, hematocrit), coagulation (international normalized ratio (INR), activated partial thromboplastin time (aPTT), D-dimers), and blood chemistry (sodium, potassium, creatinine, urea, uric acid, total bilirubin, direct bilirubin, total protein, albumin, C-reactive protein, troponin I or T, brain natriuretic peptide (BNP) or N-terminal prohormone of brain natriuretic peptide (NT-proBNP), creatine kinase (CK), aspartate transaminase (AST), alanine transaminase (ALT), alkaline phosphatase, lactate dehydrogenase (LDH), gamma-glutamyl transpeptidase (GGT), thyroid-stimulating hormone (TSH), and glucose) must be assessed from a routinely drawn blood/urine sample.
Vital signs as well as $O_2$ supplementation are recorded as indicated in Section 10.2 Vital signs and Section 10.15 Oxygen administration.
Concomitant medication, invasive procedures and AE/SAE will be reported continuously.
Arterial blood gas analysis (incl. pH, $PaO_2$, $SaO_2$, $PaCO_2$, actual $HCO_3^-$, and actual base excess) is performed with blood drawn from patients with study-independent arterial access (e.g. for TBY procedure) (as part of the PROOF trial; total blood amount: 2 mL).
A second batch of blood samples for biomarker determination (i.e. two gel serum separation Vacutest® tubes of 5 mL (Yellow cap) and two plasma EDTA K2 Vacutest® tubes of 4 mL (Lavender caps)) is taken (as part of the PROOF biomarker sub-study; see Section 10.19; total blood amount: 18 mL). Blood for biomarkers should be drawn only from patients with study-independent venous or arterial access.
A 12-lead-ECG is repeated.
For assessment of pre-existing dementia, the Informant Questionnaire on Cognitive Decline in the Elderly (IQCODE) will be performed; closest relatives should be interviewed (face-to-face or by phone) within 48 hours after stroke onset.
In case of deferred consent, if the patient has not regained the ability to provide his / her own informed consent to further study participation and no legally authorized representative is available, the procedure to install a legally authorized representative must be initiated 72 hours after admission at the latest.

## 9.9 V6 – Day 5 ±2 after start of NBHO (or randomization in the control arm) or at discharge (whichever occurs first)

V6 is to be performed on day 5, or earlier if subject is to be discharged, but must occur prior to discharge.
Physical and neurological examination (incl. NIHSS) and tympanic temperature are repeated.
Additionally, mRS and Barthel Index are assessed.
Stroke subtype classification, length of ICU (defined as a ward with capacity for mechanical ventilation and/or continuous monitoring of vital parameters (including stroke units)) stay, hospital stay and duration of mechanical ventilation (invasive and non-invasive) should be documented at this visit, but, if results are incomplete, it may be completed at V7.
For safety reasons, blood samples incl. full blood count (white blood cells, platelet count, erythrocytes, hemoglobin, hematocrit), coagulation (international normalized ratio (INR), activated partial thromboplastin time (aPTT), D-dimers), and blood chemistry (sodium, potassium, creatinine, urea, uric acid, total bilirubin, direct bilirubin, total protein, albumin, C-reactive protein, troponin I or T, brain natriuretic peptide (BNP) or N-terminal prohormone of brain natriuretic peptide (NT-proBNP), creatine kinase (CK), aspartate transaminase (AST), alanine transaminase (ALT), alkaline phosphatase, lactate dehydrogenase (LDH), gamma-glutamyl transpeptidase (GGT), thyroid-stimulating hormone (TSH), and glucose) are taken (as part of the PROOF trial; total blood amount: 16.5 mL). If at V6 safety laboratory assessments are not regarded as clinical routine and the patient does not have a routinely inserted indwelling venous catheter, blood for study-dependent laboratory assessments is drawn through venipuncture. Blood samples for study-dependent safety laboratory assessments at V6 are only drawn if the patient or the respective LAR gives written informed consent to participation in the PROOF trial.

Vital signs as well as $O_2$ supplementation are recorded as indicated in Section 10.2 Vital signs and Section 10.15 Oxygen administration.
Concomitant medication, invasive procedures and AE/SAE will be reported continuously.

## 9.10 V7 – Day 90 ±10 after start of NBHO (or randomization in the control arm)

All study patients will receive clinical follow-up at day 90 ±10 after start of NBHO (or randomization in the control arm).
Physical and neurological examination (incl. NIHSS) are repeated.
Additionally, mRS, Barthel Index, MoCA, SIS-16, EQ-5D-5L and MADRS are assessed. The mRS should be performed by an investigator blinded to study treatment.
In case stroke subtype classification, length of ICU stay, hospital stay and duration of mechanical ventilation (invasive and non-invasive) have not been documented at V6, the missing information should be completed at this visit.
Vital signs as well as $O_2$ supplementation are recorded as indicated in Section 10.2 Vital signs and Section 10.15 Oxygen administration.
Concomitant medication at the time of visit as well as invasive procedures, all non-serious AEs with a causal relationship to the IMP and all SAEs that occurred between V6 and this visit will be reported (see Section 11.2 Period of Observation and Documentation).
In case a face-to-face visit is not feasible, a phone interview should be performed. Latter excludes physical and neurological examination (incl. NIHSS) and the assessment of vital signs.

**Table 13:** Items to be assessed during V7 if performed face-to-face versus via phone

| | **Face-to-Face** | **Phone** |
|---|---|---|
| Physical and neurological examination incl. NIHSS | x | - |
| Stroke subtype classification[1] | x | x |
| Length of ICU/hospital stay / duration of ventilation[2] | x | x |
| mRS[3] | x[3] | x[3] |
| Barthel Index | x | x |
| MoCA | x | x[4] |
| SIS-16 | x | x |
| EQ-5D-5L | x | x[5] |
| MADRS | x | x |
| Vital signs (systolic and diastolic blood pressure, heart rate and respiratory rate, $SpO_2$, and – if available – $etCO_2$) | x | if available |
| Recording of oxygen administration (reason, flow-rate/$FiO_2$, mask-type/method) | x | x |
| Concomitant medication and invasive procedures[6] | x | x |
| AE reporting[7] | x | x |
| SAE reporting | x | x |

1. if not completed at day 5 or before discharge, or if results are incomplete, it should be completed at day 90. Stroke etiology as classified by Trial of Org 10172 in Acute Stroke Treatment (TOAST) criteria.
2. intensive care unit (ICU) is defined as a ward with capacity for mechanical ventilation and/or continuous monitoring of vital parameters (including stroke units); invasive and non-invasive mechanical ventilation is reported separately.
3. mRS assessments at V7 follow-up should be performed by an investigator blinded to study treatment.
4. MoCA 5-minute protocol
5. EQ-5D-5L telephone interview version
6. any invasive procedure (e.g. intravenous/intra-arterial thrombolysis, thrombectomy, stenting, carotid surgery, decompressive hemicraniectomy, cardioversion, patent foramen ovale (PFO) closure)
7. only non-serious AEs with a causal relationship to the IMP and all SAEs have to be documented (see Section 11.2 Period of Observation and Documentation for details)

# 10 METHODS OF DATA COLLECTION

## 10.1 Physical/neurological examination

Following parameters will be examined on the predefined study days and documented in the corresponding form: general appearance, skin, HEENT (head, eye, ear, nose and throat exam), neck, respiratory, cardiovascular, gastrointestinal, back, extremities incl. reflexes. Pathological and clinically relevant findings will be documented as adverse events/serious adverse events.

## 10.2 Vital signs

Vital signs (systolic and diastolic blood pressure, heart rate and respiratory rate, $SpO_2$, and – if available – $etCO_2$) determined on predefined study days and time points (see Section 2 Trial Schedule) will be documented as numerical values on appropriate eCRF-pages.

Furthermore, vital signs may be recorded at any time, if medically imperative for clarification of clinical signs and symptoms. Pathological and clinically relevant findings will be documented as adverse events/ serious adverse events.

## 10.3 National Institute of Health Stroke Scale (NIHSS)

The NIHSS (http://www.nihstrokescale.org) is a reliable tool for rapidly evaluating the effects of acute cerebral infarction.[164] In anterior circulation stroke, early NIHSS at 24 hours corresponds well with long-term outcome and quality of life.[156] Raters will be required to be certified in the use of the NIHSS. A trained observer rates the study subject's ability to answer questions and perform activities relating to level of consciousness, language, visual-field loss, extraocular movement, motor strength, ataxia, dysarthria, sensory loss, and extinction and inattention (formerly neglect). There are 15 items. Ratings for each item are scored with 3 to 5 grades, with 0 as normal and a maximum possible total severity score of 42 for all items (see Appendix 4). There is an allowance for untestable items.

## 10.4 Brain Imaging acquisition and assessment

Acquisition: Brain imaging modality at screening as per standard care, i.e. CT- or MRI-based. Minimal standard care includes non-contrast CT, CT angiography and, if recommended by the attending physician, CT perfusion, or DWI, apparent diffusion coefficient (ADC), fluid-attenuated inversion recovery (FLAIR), T2*, time-of-flight (TOF) angiography and, if recommended by the attending physician, gadolinium-enhanced MR perfusion, respectively.
eASPECTS software (Brainomix, Oxford, UK), or a comparable application, is recommended for automated and fast determination of ASPECTS (see inclusion criteria).

Extracranial CT- or MRI-based vessel imaging at baseline (not required for study participation, see inclusion criteria), digital subtraction angiography during TBY procedure as well as any other additional (study-independent) brain or vessel imaging performed during the course of the study (until day 30) must be provided to the imaging core lab.

MRI follow-up brain imaging (infarct volume assessment at V5) must be performed in between 18 and 72 hours after baseline brain imaging including DWI, ADC, FLAIR, T2*, TOF angiography as minimal standard. If brain imaging is not feasible in between 18 and 72 hours), which is defined as minor protocol deviation, MRI is to be performed the soonest possible until discharge.

Assessment:

All brain imaging assessments are performed by two independent neuroradiologists blinded to treatment allocation. A detailed description can be found in the Image Interpretation Guidelines.

For the primary endpoint analysis, infarct core volume at baseline will be assessed on either CBF maps using the < 30% threshold (or on CT angiography source images* in case CT perfusion scans are of insufficient quality or in patients in whom no CT perfusion had been conducted) or on DWI, depending on the respective baseline brain imaging modality, i.e. CT or MRI. Infarct volume at follow-up will be primarily determined on MRI (FLAIR in conjunction with DWI). Only if no follow-up MRI is available, infarct volume will be assessed on the follow-up non-contrast CT that is closest to the 24-hour time point.

* The ability of CT angiography to show intracranial arterial occlusion and collateral blood flow in patients with acute stroke has been established more than 20 years ago [165]. Only recently, multiphase CT angiography has been observed to predict tissue fate regionally in acute ischemic stroke patients similar to CT perfusion [166, 167]. Others recently observed that also single-phase CT angiography is comparable to multiphase CT angiography for the selection of patients with ischemic cores of < 31 mL and < 70 mL, and ≥ 100 mL [168]. The authors conclude that this technique allows the selection of patients for endovascular therapy by accurately predicting lower infarct core volume cutoffs. Although the scientific basis is still limited, CT angiography source images are a valuable diagnostic alternative in patients for whom CT perfusion cannot be obtained. It will be used as an alternative method for core volume identification in patients without CT perfusion within the PROOF study.

## 10.5 Stroke subtype classification

Stroke subtype classification should be completed at V6 (Day 5 or before discharge), but, if results are incomplete, it may be completed at V7 (Day 90 ± 10). Stroke etiology as classified by TOAST criteria.

## 10.6 Length of ICU/hospital stay / duration of ventilation

The length of ICU and hospital stay is documented in days with day 1 being defined as the day of admission and the last day of the stay being defined as the day of discharge. Duration of ventilation is documented in hours (commenced but not completed hours are counted); invasive and non-invasive mechanical ventilation is reported separately.

ICU is defined as a ward with capacity for mechanical ventilation and/or continuous monitoring of vital parameters (including stroke units).

## 10.7 Modified Rankin Scale (mRS)

Rather than neurological function, the mRS (http://www.rankinscale.org) measures independence through an interview-based assessment of specific tasks pre- and post-stroke, which takes less than 5 minutes to perform.[169, 170] The scale consists of 7 grades, from 0 to 6, with 0 corresponding to no symptoms and 6 corresponding to death. Raters will be required to be certified in the use of the mRS (see Appendix 5). Phone assessment of the mRS has been validated.[171, 172]

## 10.8 Barthel Index

The BI (http://www.pmidcalc.org/?sid=14258950&newtest=Y) consists of 10 items that measure a person's daily functioning, specifically the activities of daily living and mobility.[173] The items include feeding, moving from wheelchair to bed and returning, grooming, transferring to and from a toilet, bathing, walking on a level surface, going up and down stairs, dressing, and maintaining continence of bowels and bladder. The assessment can be used to determine a baseline level of functioning and can be used to monitor change in activities of daily living over time. The items are weighted according to a scheme developed by the authors. The person receives a score based on whether they have received help while doing the task. The scores for each of the items are summed to create a total score up to a maximum of 100. The higher the score, the more "independent" the person is. The BI takes less than 5 minutes to perform (see Appendix 6Appendix ). Phone assessment of the BI has been validated.[174, 175]

## 10.9 IQCODE (Informant Questionnaire on Cognitive Decline in the Elderly)

The 16-item IQCODE (http://rsph.anu.edu.au/research/tools-resources/informant-questionnaire-cognitive-decline-elderly) is an informant questionnaire that seeks to retrospectively ascertain change in cognitive and functional performance over a 10-year time period.[176] 16-item IQCODE is designed as a brief screen for potential dementia, usually administered as a questionnaire given to the relevant proxy. For each item the chosen proxy scores change on a five-point ordinal hierarchical scale, with responses ranging from 1: 'has become much better' to 5: 'has become much worse'. This gives a sum-score of 16 to 80 that can be averaged by the total number of completed items to give a final score of 1.0 to 5.0, where higher scores indicate greater decline. The closest available proxy should be interviewed (face-to-face or by phone) within 48 hours after stroke onset (see Appendix 7).

## 10.10 Montreal Cognitive Assessment (MoCA)

The MoCA (http://www.mocatest.org) is a global cognitive screening test [177] with a high sensitivity for detecting mental decline due to stroke.[178] It screens 8 domains: visuospatial/executive, naming, memory, attention, language, abstraction, delayed recall, and orientation. The assessment takes approximately 10 minutes. The highest possible total score is 30 points, and the assessment is available in 31 languages (see Appendix 8). A phone version of the MoCA, the MoCA 5-minute protocol, has been validated (see Appendix 9).[179]

## 10.11 Stroke Impact Scale (SIS)-16

The SIS-16 (http://www.kumc.edu/school-of-medicine/preventive-medicine-and-public-health/research-and-community-engagement/stroke-impact-scale/sis-16.html) is a 16-item physical dimension instrument that was developed as a brief, stand-alone tool for measuring the physical aspects of stroke recovery.[180] The 16 physical aspects are rated on a 1 to 5 scale as follows: not difficult at all (5), a little difficult (4), somewhat difficult (3), very difficult (2), and could not do at all (1). The SIS-16 takes 5 to 10 minutes to complete and is also available in a proxy version that can be used when patients are unable to answer themselves (see Appendix 10). Phone assessment of the SIS has been validated.[181]

## 10.12 EQ-5D-5L

The EQ-5D-5L (https://euroqol.org/eq-5d-instruments/eq-5d-5l-about) is provided by the EuroQol Research Foundation) and essentially consists of 2 pages: the EQ-5D descriptive system and the EQ visual analogue scale (EQ VAS).[182] The descriptive system comprises five dimensions: mobility, self-care, usual activities, pain/discomfort and anxiety/depression. Each dimension has 5 levels: no problems, slight problems, moderate problems, severe problems and extreme problems. The patient is asked to indicate his/her health state by ticking the box next to the most appropriate statement in each of the five dimensions. This decision results in a 1-digit number that expresses the level selected for that dimension. The digits for the five dimensions can be combined into a 5-digit number that describes the patient's health state. The EQ VAS records the patient's self-rated health on a vertical visual analogue scale, where the endpoints are labeled 'The best health you can imagine' and 'The worst health you can imagine'. The VAS can be used as a quantitative measure of health outcome that reflect the patient's own judgment. The EQ-5D has been validated in stroke (see Appendix 11). For assessment via phone, the telephone interview version of the EQ-5D-5L is available (see Appendix 12).

## 10.13 Montgomery–Åsberg Depression Rating Scale (MADRS)

The MADRS is a ten-item diagnostic questionnaire to measure the severity of depressive episodes in patients with mood disorders.[183] The questionnaire includes questions on the following symptoms 1. Apparent sadness 2. Reported sadness 3. Inner tension 4. Reduced sleep 5. Reduced appetite 6. Concentration difficulties 7. Lassitude 8. Inability to feel 9. Pessimistic thoughts 10. Suicidal thoughts. The MADRS has been used in recent stroke trials.[184] (see Appendix 13). Phone assessment of the MADRS has been validated.[185, 186]

## 10.14 Tympanic temperature

Tympanic temperature will be used as a surrogate for body temperature and determined according to the respective site's clinical standards at the prespecified time points.

## 10.15 Oxygen administration

Oxygen administration including reason (either NBHO or standard of care oxygen supplementation), flow-rate or $FiO_2$ in case of ventilation, and mask-type/method are documented in the eCRF at prespecified time points (see Section 0 Trial Schedule), including all documented pre-randomization and pre-hospital changes of $O_2$ administration with reason, flow-rate, mask-type, duration, $SpO_2$, and – if available – $etCO_2$.

## 10.16 Concomitant medication and invasive procedures

Concomitant medication is documented as indicated in Section 8.4.5 Prior and Concomitant Medication. Additionally, invasive procedures such as intravenous/intra-arterial thrombolysis, thrombectomy, stenting, carotid surgery, decompressive hemicraniectomy, cardioversion, patent foramen ovale (PFO) closure are documented on designated eCRF-sheets including date and time of intervention start, indication, and complications.

## 10.17 12-lead ECG

12-lead ECGs are to be performed according to local clinical standards. Only pathological and clinically relevant findings in 12-lead ECG determined on predefined study days will be documented on appropriate eCRF-pages. No records of numerical values, such as heart rate, particular times and intervals will be collected.

12-lead ECG may be recorded at any time at discretion of the responsible investigator, if medically imperative for clarification of clinical signs and symptoms. Pathological and clinically relevant findings will be documented as adverse events/ serious adverse events.

## 10.18 Safety laboratory assessment

The following parameters will be determined at the local laboratory of the respective trial site using routine blood samples drawn at Screening and V5. At V6 however, blood sampling is regarded as study-dependent.

Laboratory parameters to be assessed:

- Full blood count (ca. 3 mL of whole blood in EDTA):
    - white blood cells
    - platelet count
    - erythrocytes
    - hemoglobin
    - hematocrit
- Coagulation (ca. 3 mL of whole blood in Citrate):
    - international normalized ratio (INR),
    - activated partial thromboplastin time (aPTT),
    - D-dimers
- Blood chemistry (ca. 10.5 mL of whole blood; 7.5 mL in Li-Heparin and 3 mL in Na-F):
    - sodium
    - potassium
    - creatinine
    - urea
    - uric acid
    - total bilirubin
    - direct bilirubin
    - total protein
    - albumin
    - C-reactive protein
    - troponin I or T
    - brain natriuretic peptide (BNP) or N-terminal prohormone of brain natriuretic peptide (NT-proBNP)
    - creatine kinase (CK)
    - aspartate transaminase (AST)
    - alanine transaminase (ALT)
    - alkaline phosphatase
    - lactate dehydrogenase (LDH)
    - gamma-glutamyl transpeptidase (GGT)
    - thyroid-stimulating hormone (TSH)
    - glucose

Blood samples will be analyzed at the respective trial sites. All parameters will be documented on appropriate eCRF-pages.

Further laboratory parameters may be determined at any time during the study at discretion of the responsible investigator. Pathological and clinically relevant findings will be documented as adverse events/serious adverse events.

Total study-dependent blood volume for safety laboratory assessment: 16.5 mL.

In women ≤ 55 years a urine or serum β-HCG test has to be done before initiation of study treatment, except if surgically sterile; in women >55 years pregnancy must be excluded only in case of increased probability e.g. due to in-vitro fertilization.

## 10.19 Biomarkers (Pharmacodynamics) Substudy

**Procedure**: Participants will be asked to consent to donate 4 blood samples to the PROOF blood biobank, which will be centralized at the Neurovascular Research Laboratory at the Fundació Hospital Universitari Vall d'Hebron-Institut de Recerca (VHIR) in Barcelona, Spain. Participation in the Biomarker substudy is optional and analysis of biomarker samples will only be done if the patient/LAR consents to participate in the substudy (see Sections 4.3, 9, 14.5).

Blood samples collected will be used to measure the concentrations of candidate proteins in the blood that could be influenced by hyperoxygenation therapy. Several biomarkers among oxidative stress, matrix metalloproteinases and inflammatory markers (e.g. IL6, ADAMTS13, SAA, VCAM1, MMP-9, MMP-2, MMP-3, MMP-13, endostatin, MDA) will be evaluated through enzyme-linked immunosorbent assays.

Serum and plasma blood samples will be collected from each Participant at the following visits:

- Screening visit: 18mL
- Visit 5 (24 ±6 hours): 18mL

Total study-dependent blood volume for biomarker analysis: 36 mL; biomarker blood is only drawn using a pre-existing vascular access; no venipuncture is required.

Blood samples will be collected and processed following the PROOF Biomarkers handling and storage instructions, to be provided by VHIR in order to harmonize these procedures among the Participating Sites. Biological samples will be labelled with the PROOF participant ID number at each Participating Site. The Participating Site must store the blood samples in a biological sample freezer at -80°C, until the end of Study recruitment period. After the last visit of their last patient, the Participating Site will send its blood samples to the PROOF blood biobank at VHIR. Thus, results of substudy analyses will become available only after the end of the PROOF main trial and will therefore not influence study treatment overall or on the individual patient level. VHIR will engage a common shipment company to collect and ship the samples to VHIR from each Participating Site. Once the blood samples have arrived at VHIR they will be stored in a freezer for biological samples at -80°C until they are used for determination of blood-based biomarkers.

At the end of the Study, information from blood-based biomarkers will be tested to identify "NBHO therapy-response safety and efficacy biomarkers"

The specific objectives of this sub-study are:

- To create a larger European bio resource of stroke blood samples (PROOF-Bio-Bank) that will allow to biologically demonstrating some of the expected benefits of NBO.
- To measure blood biomarkers related with the clinical and neuroimaging endpoints and to define the clinical utility of candidate biomarkers involving three main pathways (Oxidative stress, matrix metalloproteinases and inflammation).
  To define a prototype to be used as a Point-of-Care (POC) device for blood markers to guide stroke NBHO therapy triage and management.

**Quality assurance**: The procedures performed in the Neurovascular Research Laboratory at VHIR will follow a specific standard operating procedure (SOP) for sample maintenance and monitoring. This SOP will take into account all of the quality control requirements established by the VHIR and will comply with all applicable laws and regulations.

**Data management:** VHIR will create the PROOF biomarker database with all of the results of the blood-based biomarkers determinations related to each PROOF Participant ID number that will be shared with

KKS Heidelberg. The anonymized neuroimaging recovery biomarkers and clinical information will be stored by VHIR in order to perform statistical analysis at the end of the study. Procedure and data format shall be given in the data management plan of VHIR.

**Biomarker sample management plan: After analysis,** any remaining blood samples will be anonymized and kept in the Neurovascular Research Laboratory in VHIR in Barcelona as a collection registered with Instituto de Salud Carlos III, from the Spanish Ministry of Health, to be used in future studies in the line of stroke biomarkers, if consent has been given by the patient/LAR.

## 10.20 Arterial blood gases (Pharmacokinetics)

Arterial blood gases will only be assessed in patients with study-independent arterial access (e.g. for TBY procedure). Arterial blood gas analysis includes the following pharmacokinetic parameters, which will be determined or calculated: pH, $PaO_2$, arterial oxygen saturation ($SaO_2$), $PaCO_2$, actual $HCO_3^-$, and actual base excess. Total study-dependent blood volume for arterial blood gas analysis: 2 mL.

**Table 14:** Study-related laboratory assessments from patients' blood

| | S | R | V1 | V2 | V3 | V4 | V5[2] | V6[4] | V7 | Total |
|---|---|---|---|---|---|---|---|---|---|---|
| Safety laboratory | 0 mL[1] | | | | | | 0 mL[1] | 16.5 mL | | 16.5 mL |
| Arterial blood gases | | | | | 0 mL[1] | | 2 mL[3] | | | 2 mL |
| **Total** | - | - | - | - | - | - | 2 mL | 16.5 mL | - | **18.5 mL** |
| Biomarkers (WP6) | 18 mL | | | | | | 18 mL | | | 36 mL |
| **Total including biomarkers (WP6)** | 18 mL | - | - | - | - | - | 20 mL | 16.5 mL | - | **54.5 mL** |

[1]Safety laboratory assessments at Screening (S) and V5 as well as arterial blood gas analysis during TBY (V3) are study-independent procedures

[2]Blood sampling from Screening to V5 does not require study-dependent venipuncture; all patients will have a venous plus/minus an arterial access or both as part of clinical routine.

[3]Arterial blood gas analysis at V5 will only be performed if an arterial access is available as part of clinical routine.

[4]At V6 blood sampling and – if required (i.e. no study-independent venous access in place) – venipuncture will only be conducted if the patient or the respective LAR gives written informed consent.

## 10.21 (Serious) Adverse Events

for definition see Section 11.1 Definitions.

Adverse events will be interrogated for at each contact between the responsible investigator and the study subject. Furthermore, all pathological and clinically relevant findings in physical and neurological examinations, vital signs, 12-lead ECGs, clinical chemistry, hematology, and clotting will be documented as adverse events.

Wherever possible, adverse events will be reported on the basis of the Common Terminology Criteria for Adverse Events (CTCAE) v5.0.

AEs occurring until Visit 6 will be recorded by start and end time of day (in hour and minute). In case SAEs occur during until V6 they have to be documented as AEs as well.

Between V6 and V7, only SAE and non-serious AEs with a causal relationship to study treatment must be documented. Non-serious AEs which are not related to study treatment are only documented until V6.

# 11 ADVERSE EVENTS

## 11.1 Definitions

### *11.1.1* Adverse Event

According to ICH-GCP, an adverse event (AE) is defined as follows: Any untoward medical occurrence in a subject administered a pharmaceutical product and which does not necessarily have a causal relationship with this treatment. An AE can therefore be any unfavorable and unintended sign (including an abnormal laboratory finding), symptom, or disease temporally associated with the use of a medicinal (investigational) product, whether or not related to the medicinal (investigational) product.

An AE may be:
- New symptoms/ medical conditions
- New diagnosis
- Changes of laboratory parameters

The criteria that should be considered when determining whether an abnormal test finding should be reported as adverse event are as follows:
- Test result is associated with accompanying symptoms, and/or
- test result requires diagnostic testing or medical/surgical intervention, and/or
- test result leads to a change in trial dosing outside the protocol-stipulated dose adjustments, or discontinuation from the trial, significant additional concomitant drug treatment, or other therapy, and/or
- test result is considered clinically relevant at the discretion of the investigator or sponsor
- Intercurrent diseases and accidents
- Worsening of medical conditions/ diseases existing before clinical trial start
- Recurrence of disease
- Increase of frequency or intensity of episodic diseases.

A pre-existing disease or symptom will not be considered an adverse event unless there will be an untoward change in its intensity, frequency or quality. This change will be documented by an investigator.
Surgical procedures themselves are not AEs; they are therapeutic measures for conditions that require surgery. The condition for which the surgery is required may be an AE. Planned surgical measures permitted by the clinical trial protocol and the condition(s) leading to these measures are not AEs, if the condition leading to the measure was present prior to inclusion into the trial. In the latter case the condition should be reported as medical history.
AEs are classified as "non-serious" or "serious".

### *11.1.2* Serious Adverse Events and Adverse Events of Special Interest

A serious adverse event (SAE) is one that at any dose:
- Results in death
- Is life-threatening (the term life-threatening refers to an event in which the subject was at risk of death at the time of event and not to an event which hypothetically might have caused death if it was more severe)
- Requires hospitalization or prolongation of existing hospitalization*
- Results in persistent or significant disability/ incapacity**
- Is a congenital anomaly/ birth defect or
- Is otherwise medically relevant

* Hospitalization for performing protocol-required procedures or administration of study treatment is not classified as an SAE. Hospitalizations for disease-related procedures (surgery, imaging, laboratory tests) or any procedures planned before entry into the study are not considered SAEs. Hospitalizations for social reasons in the absence of an adverse event are not classified as SAEs either.
** Persistent or significant disability or incapacity means that there is a substantial disruption of a person's ability to carry out normal life functions. The irreversible injury of an organ function (e.g. paresis, diabetes, cardiac arrhythmia) fulfills this criterion.

Medical and scientific judgment should be exercised in deciding whether expedited reporting is appropriate in other situations - such as important medical events that may not be immediately life threatening or result

in death or hospitalization but may jeopardize the patient or may require intervention to prevent one of the other outcomes listed above. These should also usually be considered serious (examples of such events are intensive treatment in an emergency room or at home for allergic bronchospasm; blood dyscrasias or convulsions that do not result in hospitalization; or development of drug dependency or drug abuse).

Following events are defined as **adverse events of special interest (AESI)** in the PROOF trial and have to be reported by investigator in accordance with the requirements for the reporting of SAE:

- parenchymal hematoma 1 (class 1c, Heidelberg bleeding classification [101])
- parenchymal hematoma 2 (class 2, Heidelberg bleeding classification [101])
- parenchymal hematoma remote from infarcted brain tissue (class 3, Heidelberg bleeding classification [101])
- intraventricular hemorrhage (class 3b, Heidelberg bleeding classification [101])
- subarachnoid hemorrhage (class 3c, Heidelberg bleeding classification [101])
- subdural hematoma (class 3c, Heidelberg bleeding classification [101])
- intracranial hemorrhages (ICH) with neurological deterioration <u>if the ICH is the predominant cause of the deterioration.</u> For this, a classification according to the **ECASS III** and **Heidelberg bleeding classification** will be provided on the SAE-form.

### *11.1.3* Serious Adverse Reaction

SAEs that potentially may be attributed to the investigational medicinal product (IMP) are to be classified as Serious Adverse Reactions (SARs).

### *11.1.4* Expectedness

An ‘unexpected’ adverse reaction is one the nature or severity of which is not consistent with the applicable product information, e.g., Investigator’s Brochure (IB) or Summary of medical Product Characteristics (SmPC).

### *11.1.5* Suspected Unexpected Serious Adverse Reaction (SUSAR)

SAEs that are both ‘suspected’, i.e., possibly related to the study drug (investigational medicinal product (IMP)) and ‘unexpected’, i.e., the nature and/ or severity of which is not consistent with the applicable product information are to be classified as Suspected Unexpected Serious Adverse Reactions (SUSARs).
In case either the investigator who primarily reported the SAE or the second assessor classifies the SAE as ‘suspected’ *[(i.e., either as ‘definitely’ or ‘probable’ or ‘possible’ related to IMP or ’not assessable’)]* and the SAE is unexpected, it will be categorized as a SUSAR.
All SUSARs are subject to an expedited reporting to the responsible ethics committee(s), the competent authorities in all member states concerned and to all participating investigators.

### *11.1.6* Grading of AEs

The grading of AEs in this trial will be carried out on the basis of the 5-grade scale defined in the CTCAE 5.0:

| | |
|---|---|
| Grade 1: | Mild |
| Grade 2: | Moderate |
| Grade 3: | Severe |
| Grade 4: | Life threatening or causing disability |
| Grade 5: | AE resulting in death |

The grading of all AEs listed in the CTCAE v5.0 will be based on the information contained therein. The grading of all other AEs, i.e., those not listed in the CTCAE v5.0 will be performed by a responsible investigator, based on definitions given above.
Clarification of the difference in meaning between "serious" and "severe":
The terms “serious” and “severe” are not synonymous. The term ‘severe’ should be used to describe the intensity (severity) of a specific event; the event itself, however, may be of relatively minor significance (such as severe headache). This is not the same as “serious”, which is based on the existence of one of the above-mentioned seriousness criteria.

The investigator has two possibilities dependent on the clinical situation to:

a) document a new AE for every changed grade and/or

b) document just 1 AE with highest relevant or most frequently observed grade and comment "intermittent".

### *11.1.7* Relationship and Outcome of AEs

The investigator will evaluate each AE that occurred after administration of the IMP regarding the relationship with the administration of the IMP:
The assessment if there is a relationship between the investigational product administered and the AE/SAE is a clinical decision that is done on the basis of information available at that time.
A "no" includes the following aspects:

1. There is a clear alternative explanation, e.g. mechanical bleeding in the surgical area

or

2. Missing plausibility. Examples: a study participant was struck by a car without any signs of disorientation that might have led to the accident. Development of cancer only a few days after first application of study medication.

A "yes" should be chosen if there is a reasonable possibility for a causal relationship between the investigation product and the AE or if a relationship cannot be excluded.

All subjects who have reportable AEs, whether considered associated with the use of the trial medication or not, must be monitored to determine the **outcome**. The clinical course of the AE will be followed up until resolution or normalization of changed laboratory parameters or until it has changed to a stable condition. This also holds for on-going AEs/SAEs of withdrawn subjects.
The outcome of an AE at the time of the last observation will be classified as:

| | |
|---|---|
| Recovered / resolved: | All signs and symptoms of an AE disappeared without any sequels at the time of the last interrogation. |
| Recovering / resolving: | The intensity of signs and symptoms has been diminishing and / or their clinical pattern has been changing up to the time of the last interrogation in a way typical for its resolution. |
| Not recovered/not resolved: | Signs and symptoms of an AE are mostly unchanged or worsened at the time of the last interrogation. |
| Recovered / resolved with sequel: | Actual signs and symptoms of an AE disappeared but there are sequels related to the AE. |
| Fatal: | Resulting in death. If there is more than one adverse event only the adverse event leading to death (possibly related) will be characterized as 'fatal'. |
| Unknown | The outcome is unknown or implausible and the information cannot be supplemented or verified. |

The action taken with the IMP will be assigned to one of the following categories:

| | |
|---|---|
| Dose not changed: | No change in the dose of the IMP. |
| Dose reduced: | Reduction in the dose of the IMP. |
| Dose increased: | Increase in the dose of the IMP. |
| Drug withdrawn: | Discontinuation of the IMP. |
| Unknown: | The information is unknown or implausible and it cannot be supplemented or verified |
| Not applicable: | The question is implausible (e.g. the subject is dead). |

The term "countermeasures" refers to the specific actions taken to treat or alleviate adverse events or to avoid their sequels. Following categories will be used to categorize the countermeasures to adverse events:

| | |
|---|---|
| None: | No action taken. |

| | |
|---|---|
| Drug treatment: | Newly-prescribed medication or change in dose of a medication. |
| Others: | Other countermeasures, e.g. an operative procedure. |

## 11.2 Period of Observation and Documentation

Adverse events (AEs) will be ascertained by the investigators using non-leading questions, noted as spontaneously reported by the patients to the medical staff or observed during any measurements on all study days.

The observation period begins with the first administration of the IMP/standard therapy (before the first administration of the IMP/standard therapy: medical history) and ends as follows:

- Non-serious AEs with a causal relationship to study treatment (Adverse Reactions) and all SAEs have to be documented until the last study visit, i.e. 90 days after study treatment.
- Non-serious AEs with no causal relationship to study treatment have to be documented until V6 (day 5/discharge.

AEs including SAEs will be documented in the patient file and in the CRF. SAEs must be documented additionally on the SAE Form (see also 11.3). All subjects who present AEs, whether considered associated with the use of the trial medication or not, will be monitored by the responsible investigator to determine their outcome; this applies to withdrawals too (see also 8.1.7).
AEs occurring before the 5 days visit will be recorded by start and end time of day (in hour and minute). All AEs occurring at a later stage will be recorded with start and end date.
The end date of the SAE is defined typically the same as for AEs. The end date of the SAE must not be later than the end date of the corresponding AE.

AEs and SAEs that are on-going at the time of death are considered not resolved or resolving.

All SAEs and their relevance for the benefit/risk assessment of the study will be evaluated continuously during the study and for the final report. All SAEs will be documented in the "Serious Adverse Event" form (see 10.3).

## 11.3 Reporting of Serious Adverse Events by Investigator

All SAE must be reported by the investigator to the responsible Safety Officer at the KKS Heidelberg within 24 hours after the SAE becomes known using the "Serious Adverse Event" form. The initial report must be as complete as possible including details of the current illness and (serious) adverse event and an assessment of the causal relationship between the event and the trial medication.

The reporting will be performed by faxing a completed 'SAE Form' to the KKS Heidelberg. Fax number:

**+49 (0) 6221 – 56 – 33725**

Only in case of technical faults in fax transmission, the SAE Form can be also submitted by e-mail:

**pharmakovigilanz.KKS@med.uni-heidelberg.de**

## 11.4 Expedited Reporting

SUSARs are to be reported to the responsible ethics committees, the competent authorities in all member states concerned and to all participating investigators and if applicable to further bodies according to international law within defined timelines, i.e. they are subject to an expedited reporting.

All SAEs will be subject to a second assessment by a designated person, who will be independent from the reporting investigator. The designated person for the present trial, referred to as the second assessor is: Prof. Dr. med. Christine Meyer-Zürn (Dept. of Cardiology, University Hospital Basel, Switzerland).

The second assessor will fill out a 'Second Assessment Form' for each SAE and send it back per fax to the responsible person at the KKS Heidelberg within 48 hours, fax-number:

**+49(0)6221 – 56 – 33725**

The 'Second Assessment Form' will contain the following information:

I) assessment of relationship between SAE and IMP (causality)

II) assessment of relationship between SAE and underlying disease

III) assessment of expectedness of SAE (derived from IB or SmPC)

IV) statement if the benefit/ risk assessment for the trial did change as a result of SAE.

The expedited reporting (to competent authorities, responsible ethics committees in all member states concerned and investigators) will be carried out by a responsible Safety Officer at KKS Heidelberg. Only SUSARs occurring after administration of IMP will undergo expedited reporting. Details concerning the reporting of SUSARs will be described in a separate document "Safety Manual".

# 12 STATISTICAL PROCEDURES

## 12.1 Sample Size Calculation

The test of the primary hypothesis is outlined in Section 12.4 Statistical Methods. As relatively little is known about the true effect of NBHO on infarct core volume, an interim analysis with adaptive calculation of sample size is planned.

The sample size calculation is based on the (absolute) ischemic core growth volumes reported by Albers et al., Ann Neurol, 2016 [187] – namely from the SWIFT-PRIME subgroup of TBY patients which reached successful recanalization at the end of TBY procedure (14.8 mL (IQR 4.9 to 33.7), N=62), which represents the group of patients in which we assume significant NBHO efficacy (based on positive experimental NBHO studies, which were studies in which focal cerebral ischemia was transient (with a duration of up to 3 hours), as a result of recanalization therapy. The mean and standard deviation are estimated as 17.8 mL ± 21.4 mL from the quartiles, assuming the quantiles of the normal distribution.

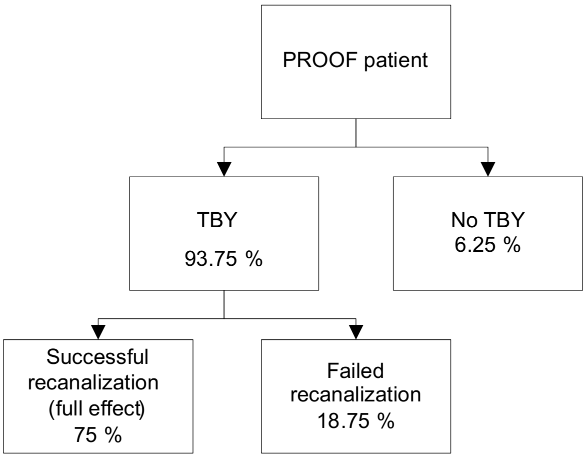


Thus, in PROOF, we assume a reduction of the relative ischemic core growth by 50% (of 17.8 mL, resolving to 8.9 mL) in 75% of NBHO-treated subjects: assuming a rate of successful recanalization of 80% [3, 187] in those 93.75% of patients who are randomized to NBHO and undergo TBY (0,8 * 0.9375 = 0.75). Adding the other 25% (20% of the 93.75% TBY patients with no/insufficient recanalization plus 6.25% of PROOF-patients in whom TBY is not attempted) plus control subjects means that the mean effect of 8.9 is reduced by 25 per cent (leading to a mean effect of 6.68 mL) as well as setting the standard deviation to satisfy the variability found in [187] (i.e. 21.4 mL), we would need 138 patients per arm for a one-sided level-alpha = 0.05 test to detect a treatment effect with a power of 80 per cent. Using an adaptive design (according to [188]) after interim analysis after 80 patients per arm (assuming 20 sites with accrual up to interim analysis), the trial may be stopped early for success (with a p-value of less than 0.0233, which is taken from Table 1, column 2, row 3 in [188])) or futility (with p ≥ 0.5) or be continued with 11 to 148 additional patients per arm. Sample size inflations have been considered for site effect calculation and patients with failed recanalization not profiting from treatment.

## 12.2 Analysis Variables

### *12.2.1* Primary analysis variable

Efficacy of NBHO treatment will be determined by an ITT analysis of difference of ischemic core growth (defined as the difference in ischemic core volume (in mL) from baseline to 24 hours) between groups; brain tissue not included in CT perfusion or MR diffusion at baseline will be excluded from lesion volume measurements; in case of premature death (i.e. death without repeat scan), patients will be treated as worst individual outcome of ischemic core growth, i.e. the growth value will be set to the difference in volume between the initial core at baseline and the initial volume at Tmax > 6 seconds or equivalent volume on CT angiography source images in case CT perfusion is of insufficient quality or was not conducted.

Missing values will be replaced for the primary endpoint using multiple imputation. Imputation uses the conditional distribution of ischemic core volume at 24 hours given baseline core volume for the intention to treat approach and given treatment group and baseline core volume for the per protocol approach. In total,

10 replicates will be generated. As a sensitivity analysis, the expected response when generating imputations will be increased or decreased by the clinically relevant difference of 6.68 mL.

### *12.2.2* Secondary analysis variables

Clinical efficacy of NBHO treatment will be determined by an ITT-analysis of the difference of change in NIHSS score from baseline to 24 hours (key secondary efficacy endpoint) between groups; in case of premature death (i.e. death prior to V5), an NIHSS score of 42 will be applied.

The following pre-specified analyses will be performed (PP and ITT):

Clinical efficacy analyses:

- Survival at V6 and V7 (90 days after randomization)
- Utility-weighted mRS at day 5/discharge and at day 90 [189] [time frames: day5/discharge, and day 90]
- categorical shift in the mRS at day 5/discharge and at day 90 [time frames: day5/discharge, and day 90]
- mRS at day 5/discharge and at day 90, dichotomized 0 to 1 (no significant disability) vs. 2 to 6 [time frames: day5/discharge, and day 90]
- mRS at day 5/discharge and at day 90, dichotomized 0 to 2 (good outcome) vs. 3 to 6 [time frames: day5/discharge, and day 90]
- Barthel Index (BI) at day 5/discharge* and at day 90* [time frames: day5/discharge, and day 90] **in case of premature death an BI score of 0 will be applied*
- NIHSS at V2*, at V4*, at 24 hours*, at day 5/discharge* and at day 90* [time frames: 5 minutes, 24 hours, day5/discharge, and day 90] *(*in case of premature death an NIHSS score of 42 will be applied)*§
- Difference of change in NIHSS score from baseline to V2 and from V2 to V5 between groups *(*in case of premature death an NIHSS score of 42 will be applied)*§
- very early neurological improvement (VENI) at V2 / early neurological improvement (ENI) at 24 hours / subacute neurological improvement (SNI) at day 5/discharge and late neurological improvement (LNI) at day 90; proportion of subjects with NIHSS drop of ≥ 4 and ≥ 8 from baseline or NIHSS score 0 to 3 [time frames: 5 minutes, 24 hours, day5/discharge, and day 90]§
- Complete recovery defined as a decrease in total NIHSS score to 3 or lower at 24 hours§
- Montreal Cognitive Assessment (MoCA) score corrected for pre-stroke cognitive status (IQCODE), EQ-5D-5L (EuroQol), Stroke Impact Scale (SIS)-16 and Montgomery-Åsberg Depression Rating Scale (MADRS) score at day 90 [time frame: day 90] will be analyzed using a linear regression model
- $PaO_2$ at 90 minutes and 24 hours

§ *with and without exclusion of patients under sedation or anesthesia*

Clinical safety analyses:

- all-cause death at day 5/discharge and day 90
- stroke-related death at day 5/discharge and day 90
- symptomatic intracranial hemorrhage (sICH) as per ECASS III definition and per Heidelberg bleeding classification [101] (see Appendix 14) at 24 hours*, until day 5/discharge* will be analyzed using logistic regression models [time frames: 24 hours, day 5/discharge] **in case of missing follow-up brain imaging due to premature death, sICH will be replaced by "sICH or death without repeat scan"*
- mRS at day 5/discharge and at day 90, dichotomized 5 to 6 (severe disability or death) vs. 0 to 4 [time frames: day5/discharge, and day 90]
- very early neurological deterioration (VEND) at V2 / early neurological deterioration (END) at 24 hours / subacute neurological deterioration (SND) at day 5/discharge and late neurological deterioration (LND) at day 90; proportion of subjects with NIHSS increase of ≥ 4 and ≥ 8 from baseline [time frames: 5 minutes, 24 hours, day5/discharge, and day 90] (*with and without exclusion of patients under sedation or anesthesia)*

- decompressive hemicraniectomy until day 5/discharge and day 90 [time frames: day5/discharge, and day 90day]
- any SAE until 24 hours, day 5/discharge and day 90 [time frames: 24 hours, day5/discharge, and day 90]
- respiratory SAE until 24 hours, day 5/discharge and day 90 [time frames: 24 hours, day5/discharge, and day 90]
- pneumonia until day 5/discharge and day 90 [time frames: day5/discharge, and day 90]
- respiratory failure leading to ventilation or death until 24 hours, day 5/discharge and day 90 [time frames: 24 hours, day5/discharge, and day 90]
- duration of ventilation (invasive only and invasive/non-invasive combined) until day 5/discharge and day 90 [time frames: day5/discharge, and day 90]
- length of stay in ICU until day 5/discharge and day 90 [time frames: day5/discharge, and day 90]
- length of stay in hospital [time frame: day 90]
- myocardial infarction until 24 hours, day 5/discharge and day 90 [time frames: 24 hours, day5/discharge, and day 90]
- major adverse cardiovascular events (MACE) including recurrent stroke, myocardial infarction and cardiovascular death until 24 hours, day 5/discharge and day 90 [time frames: 24 hours, day5/discharge, and day 90]

<u>Secondary imaging efficacy analyses:</u>

- PP only: absolute difference in ischemic core volume (in mL) [time frame: 24 hours]
- relative changes in ischemic core volume (in %) [time frame: 24 hours]
- absolute and relative ischemic core change using either NCCT or DWI-MRI (or CT angiography source images and DWI) for ischemic core estimation at baseline (i.e. NCCT (or CT angiography source images) will substitute for CBF < 30% in patients with CT-based imaging at baseline) [time frame: 24 hours]
- absolute and relative ischemic core change using CBF < 30% for ischemic core estimation at baseline in all patients, independent of imaging modality (i.e. DWI will be substituted by MR perfusion CBF < 30%) [time frame: 24 hours]
- penumbral salvage defined as (penumbra volume at baseline – infarct core volume at 24 hours) / (penumbra volume at baseline – ischemic core volume at baseline) [time frame: 24 hours]
- of patients who received TBY: proportion of TICI on DSA (final run) (as suggested in [190]) [time frame: 2 to 4 hours]
- revascularization rate on 24-hour follow-up MRA (or CTA if available) [time frame: 24 hours]

<u>Imaging safety analyses:</u>

- of patients who received MR-based acute brain imaging: new microbleeds on 24-hour follow-up MRI (vs. baseline T2*w MRI) [time frame: 24 hours]
- Incidence of any intracranial hemorrhage in 24-hour follow-up imaging (hemorrhagic transformation (HI1 and HI2) and intra-parenchymal hemorrhage (PH1 and PH2), as well as remote intracerebral hemorrhage (3a), intraventricular hemorrhage (3b), subarachnoid hemorrhage (3c), subdural hemorrhage (3d) and epidural hemorrhage; see Table 1 in [101] [time frame: 24 hours]
- of patients who received TBY: occurrence of vasospasms on DSA (final run) [time frame: 2 to 4 hours]
- ischemic lesions in new vascular territories in 24-hour DWI/FLAIR-MRI (as described in [157]) [time frame: 24 hours]
- occurrence of vasospasms in DSA (final run)

<u>Predefined exploratory analyses:</u>

- Exploratory analyses of imaging (including clot perviousness [191, 192], clot burden, and collateral status [193]) will help to understand the pathophysiology of NBHO in stroke. The precise scope of data acquisition and analysis is described in the imaging protocol and Image Interpretation Guidelines.

- Exploratory analyses of biochemical biomarkers will be useful to develop a blood-based test to monitor efficacy and safety of NBHO treatment. The specific objectives of this sub-study are:
  - To create a larger European bio resource of stroke blood samples (PROOF-Bio-Bank) that will allow to biologically demonstrate some of the expected benefits of NBHO.
  - To measure blood biomarkers related with the clinical and neuroimaging endpoints and to define the clinical utility of candidate biomarkers involving three main pathways (Oxidative stress, matrix metalloproteinases and inflammation).
  - To define a prototype to be used as a Point-of-Care (POC) device for blood markers to guide stroke NBHO therapy triage and management.

## 12.3 Definition of Trial Population to be analyzed

The primary analysis will be performed for the full analysis set which comprises all patients randomized into the trial. In this set, every patient is analyzed according to the group randomized into. Exceptions to this rule can be made for patients for whom non-eligibility became apparent after randomization, provided all three of the following conditions hold:

- the entry criterion was measured prior to randomization;
- the detection of the relevant eligibility violations can be made completely objectively;
- all subjects receive equal scrutiny for eligibility violations; (This may be difficult to ensure in an open-label study, or even in a double-blind study if the data are un-blinded prior to this scrutiny, emphasizing the importance of the blind review.)

The per-protocol set will comprise all patients who were treated according to the randomized treatment as outlined in the protocol. Specifically, patients have to be eligible according to in- and exclusion criteria. Before the study data base is locked, rules for selecting the per-protocol set will – if not already pre-defined in this protocol – be agreed upon by the steering committee (see Section 4.5 Steering Committee). The same committee will decide on a case-by-case base about exceptions to the full analysis set.
The safety set will comprise all patients who have received NHBO at least for a period of 1 hour, and will allocate the patients to the treatment they actually received, regardless of randomization.
For definition of "premature withdrawal" see Section 7.7.1 Withdrawal of Patients from the Trial; add definition of screening failure and drop-out.

Other subsets of the full analysis set comprise patients with the following characteristics:

- TICI 2b/3 at end of TBY and target mismatch profile (i.e. baseline penumbra ($T_{max}$ > 6 seconds or equivalent volume on CT angiography in case of missing or low quality CT perfusion): core (CBF < 30% compared to healthy tissue or equivalent volume on CT angiography in case of missing or low quality CT perfusion, or DWI, depending on respective imaging modality) ratio ≥ 1.2 and volume ≥ 15 mL; voxel-based post-hoc analyses) vs. TICI 2b/3 at end of TBY and no target mismatch profile
- TICI 3 at end of TBY and target mismatch profile vs. TICI 3 at end of TBY and no target mismatch profile
- TICI 2b/3 at end of TBY and baseline ischemic core volume ≤ 100 mL and target mismatch profile vs. TICI 2b/3 at end of TBY and large ischemic core at baseline (> 100 mL) and no target mismatch profile
- TICI 3 at end of TBY and baseline ischemic core volume ≤ 100 mL and target mismatch profile vs. TICI 3 at end of TBY and large ischemic core at baseline (> 100 mL) and no target mismatch profile
- TICI 2b/3 at end of TBY vs. TICI 0-2a at end of TBY or TBY not attempted
- TICI 2a-3 at end of TBY vs. TICI 0-1 at end of TBY or TBY not attempted
- Patients with complete reperfusion of target mismatch area (correlation of CT or MR perfusion with DSA (final run)) vs. patients with no or incomplete reperfusion of target mismatch area
- ASPECTS ≤ 8 on baseline NCCT or ≤ 7 on baseline DWI vs. ASPECTS ≥ 9 on baseline NCCT or ≥ 8 on baseline DWI
- intubation/ventilation vs. conscious sedation
- IVT vs. no-IVT
- age ≤ 60 vs. > 60
- age ≤ 70 vs. > 70
- age ≤ 80 vs. > 80
- NIHSS at baseline < 10 vs. 10-20 vs. > 20, +/- cross-classification with LVO-location

- intracranial LVO location: terminal ICA with involvement of the M1-segment of the MCA/carotid-T vs. proximal M1-segment vs. distal M1-segments (distal to perforating branches) vs. M2/3-segment(s)
- Time window 0-3 vs. > 3-6 hours vs. unknown stroke onset and 0-3 hours since symptom recognition vs. unknown stroke onset and > 3-6 hours since symptom recognition
- Tandem stenosis/extracranial occlusion vs. no tandem stenosis/extracranial occlusion
- Patients with vs. without silent ischemic lesions

## 12.4 Statistical Methods

The primary analysis will be performed by testing the following null hypothesis: The effect of NHBO compared with standard treatment on infarct core volume is at least 0 ml. This hypothesis will be tested at the global $\alpha = 0.05$ level (for the actual levels see Section 12.5 Interim Analyses) using a re-randomization test where the allocation algorithm will be repeated on the patient data in same sequential order, and the distribution of the difference of 24-hour infarct core volume between allocation groups will be generated using the empirical cumulative distribution function (ecdf). The one-sided p-value will be computed as the minimum of ecdf and 1 – ecdf of the difference and compared with the appropriate level (see Section 12.5 Interim Analyses).

For sensitivity analysis, a likelihood ratio test for the treatment variable in a linear model with 24-hour infarct core volume as the response and baseline infarct core volume plus stroke subtype classification and baseline hemoglobin levels as additional explanatory variables as well as the factors used for treatment allocation (see Section 8.5.1 Randomization method).

The primary analysis will be carried out on the full analysis set. As a sensitivity analysis, the analysis will be repeated on the per-protocol subset. Further secondary analyses on the primary variable comprise the analysis restricted on the following subsets of the full analysis set given in Section 12.3 Definition of Trial Population to be analyzed.

Secondary analyses will be performed on the following variables in the following fashion:

Survival will be depicted using Kaplan-Meier curves and analyzed using a Cox proportional-hazard model with the same explanatory variables as for the primary analysis.

Change of NIHSS to baseline will be taken as the response in a linear model using treatment group using the same explanatory variables as for the primary analysis.

Absolute changes in core volume, penumbral salvage and reversibility will be modeled using a linear model in a similar way as for the primary end point. Relative changes will be modeled using a log-linear model for the ratio of 24-hour / baseline measurements with the same explanatory variables as for the absolute changes. The analyses will be repeated for the PP collective.

TICI score will be tabulated against treatment group. It will be taken as a response variable in a proportional odds model for ordinal variables using treatment group and variables used for minimization as explanatory variables.

Safety endpoints will be tabulated against treatment actually received (safety set, see Section 12.3 Definition of Trial Population to be analyzed) using number of events and number of subjects with events.

Biometric analysis is defined in the statistical analysis plan, which accompanies the study protocol.

## 12.5 Interim Analyses

An interim analysis on the primary endpoint will be completed after 160 patients have been treated. If the null hypothesis can be rejected at the $\alpha_1 = 0.0233$ level, the trial will end prematurely with a successful rejection of the null hypothesis. If the null hypothesis cannot be rejected at the $\alpha_0 = 0.5$ level (i.e. the observed treatment effect is in direction of the null hypothesis), the trial will be stopped early for futility. In both cases, a complete analysis will be completed on 160 patients plus the number of patients that were accrued after the criterion for interim analysis (160 patients) was satisfied but before the decision of early stopping was made.

If the null hypothesis is accepted at the $\alpha_1$ but rejected at the $\alpha_0$ level, the trial continues with an additional sample size based on the p-value obtained in the interim analysis. The $\alpha_2$ level used for the planning of the

rest of the trial will be set to $c_\alpha/p$, where $c_\alpha = 0.0087$, with a power of 65 per cent, in order to maintain the total level of $\alpha = 0.05$ and an overall power of 80 per cent.

The results of the interim analysis will be calculated by the project statistician and forwarded to the DSMB. The results will not be disseminated inside or outside the study team.

# 13 DATA MANAGEMENT

## 13.1 Data Collection

All entries in the eCRF must be verifiable by source documents. Exceptions to this rule can be defined by the sponsor in advance. A detailed list will be provided in the Investigator Site File. Regardless, there must be a minimum documentation, which provides information on study participation and includes all medical information necessary for appropriate medical care outside of the clinical trial in the patient record.
In addition, source documents must mention that the patient has been included in an investigational study. Finally, there must be no data that are inconsistent between eCRF and source documents.
The investigator is responsible for ensuring that all sections of the eCRF are completed correctly and that entries can be verified against source data.
All protocol-required information collected during the trial must be entered into the eCRF by the investigator or a designated representative. Patient data will be documented pseudonymously. The investigator, or a designated representative, should complete the eCRF pages as soon as possible after the information is collected, preferably on the same day when a trial subject is seen for an examination, treatment, or any other trial procedure, but latest within 5 working days. Any pending entries must be completed immediately after the final examination. Explanation should be given for all missing data.
Brain images of screening, V5 and all unscheduled brain images should be uploaded within 5 working days to the core imaging laboratory Eppdata.

## 13.2 Data Handling

Data entries will undergo an automated online check for plausibility and consistency. In case of implausibility, 'warnings' will be produced during data entry (edit checks). A responsible investigator or a designated representative will be obliged either to correct the implausible data or to confirm its authenticity and to give appropriate explanation. The responsible data manager will check all explanations and resolves the warnings if the explanation is appropriate. The responsible monitor can generate special questions ("monitor query") that will be sent back to the responsible investigator. The investigator or a designated representative will have to answer them all. The responsible monitor will check all answers and resolve the monitor query if the answer is appropriate. In the same manner, queries can be generated by the data manager ("DM query").

The investigator has to confirm the accuracy of all data by signing sections online in the eCRF.

All missing data or inconsistencies will be reported back to the center(s) and have to be clarified by the responsible investigator prior to database lock. If no further corrections are to be made in the database it will be declared locked and used for statistical analysis.

All data management activities will be done according to the current SOPs of the KKS Heidelberg. Details of data management will be defined in the Data Management Plan.

## 13.3 Archiving of Essential Documents

The investigator(s) will archive all trial data (source data and Investigator Site File (ISF) including subject identification list and relevant correspondence according to Section 4.9 of the ICH Consolidated Guideline on GCP (E6) and to local law or regulations.
The sponsor or other owner like investigators of the data shall retain all other documentation pertaining to the trial for at least 10 years according to local regulations. These procedures shall include:

- the protocol including the rationale, objectives and statistical design and methodology of the trial, with conditions under which it is performed and managed, and details of the investigational product used.
- standard operating procedures
- all written opinions on the protocol and procedures,
- final report,
- audit certificate(s), if available.
- all other relevant documents of the trial master file, according to the ICH-GCP guideline

Any change of data ownership shall be documented. All data shall be made available if requested by relevant authorities.

The trial master file (TMF) including all essential documents will be archived by the Sponsor.

# 14 ETHICAL AND LEGAL ASPECTS

## 14.1 Good Clinical Practice

The procedures set out in this trial protocol, pertaining to the conduct, evaluation, and documentation of this trial, are designed to ensure that all persons involved in the trial abide by ICH harmonized tripartite guideline on Good Clinical Practice (ICH-GCP) and the ethical principles described in the applicable version of the Declaration of Helsinki. The trial will be carried out in keeping with local legal and regulatory requirements.

## 14.2 Legal bases

The study has to be conducted in compliance with the protocol, ICH-GCP and the applicable regulatory requirements.

### *14.2.1* Declaration of Helsinki

The study will be carried out in conformity with the “Ethical principles for medical research involving human subjects” of the 18th World Medical Association General Assembly in Helsinki (version 1964 including all amendments. The applicable version for the respective country will be taken into consideration.

### *14.2.2* Other Legal Bases

The other legal bases of this clinical trial are as follows (including their amendments/ up-dates, if applicable):

- ICH Topic E6, Guideline for Good Clinical Practice, including post Step 4 errata, September 1997
- Directive 2001/20/EC (April 4, 2001)
- Commission Directive 2005/28/EC (April 8, 2005)
- National regulatory requirements/guidelines of the participating countries concerning Clinical Trials
- General Data Protection Regulation (EU 2016/679)
- General national regulatory requirements

The Coordinating Investigator and all investigators will be given an up-to-date SmPC containing full details of the status of the pre-clinical and clinical knowledge of the study medication. As soon as new information is obtained, an updated version will be supplied or an amendment added to the existing SmPC.

## 14.3 Approval of Trial Protocol and Amendments

Before the start of the trial, the trial protocol, informed consent document, and any other appropriate documents will be submitted to the independent Ethics Committees (EC) as well as to the competent authorities.

A written favorable vote of the EC and an approval by the competent authorities are a prerequisite for initiation of this clinical trial. The statement of the EC should contain the title of the trial, the trial code, the trial site, and a list of reviewed documents. It must mention the date on which the decision was made and must be officially signed by a committee member. This documentation must also include a list of members of the EC present on the applicable EC meeting and a GCP compliance statement.

The investigator and the KKS Heidelberg (or institution responsible for regulatory submission, respectively) will keep a record of all communication with the EC and the regulatory authorities.

Before the first subject is enrolled in the trial, all ethical and legal requirements must be met.

All planned substantial changes will be submitted to the EC and the competent authorities in writing as protocol amendments. They have to be signed by the sponsor and biometrician and approved by the EC and the competent authorities.

## 14.4 Notification of Regulatory Authorities

In addition to the approval of the competent authority further notifications must be done or approvals received prior, during and at the end of the trial as required by local law of each participating country.

Each investigator is obliged to inform the sponsor about his/her legal obligations to notify/inform his/her local authorities or applicable boards and must fulfill this duty in all cases where this responsibility is not delegated by contract to the Sponsor or another institution (e.g. contract research organization (CRO)).

## 14.5 Subject Information and Informed Consent

In the PROOF trial, stroke patients present in an emergency situation not allowing for any delay of diagnostic work-up or therapy. Additionally, due to severe stroke symptoms, the vast majority of patients who meet the eligibility criteria of the PROOF trial are assumed to be unable to give consent in the acute admission phase and legally authorized representatives (LAR) will not be available in most cases. The choice of these eligibility criteria (i.e. including only patients with routinely TBY-treatable LVO) was inevitable in order to replicate experimental transient AIS, the only animal model in which NBHO was effective.

According to the (upcoming) EU regulation 536/2014 patients can be included in a clinical trial without prior consent in case several conditions for emergency situations are fulfilled. This is also in line with local regulations: e.g. §41 of the German Drug Law allows the start of a treatment in an emergency situation without prior consent in case the immediate treatment is necessary to save the patient's life, recover the patient's health or ease the patient's suffering. The consent has to be obtained as soon as the patient is able to give consent or a LAR is available.

Further local regulations (e.g. Czech Act 378/2007 on Pharmaceuticals, Swiss Human Research Act, Belgian Article 9 of the Law on Experiment in Human dated 7th May 2004, Spanish Royal Decree 1090/2015, Finish Medical Research Act, French Law 2004-806) also allow for this "deferred" consent process in emergency situations with patients unable to consent.

In the PROOF trial the following situation is given:

- Patients present with a condition with a high risk for death or disability.
- Efficacy of stroke treatment is highly time-dependent, which is – according to preclinical data – also true for NBHO; therefore, delay of treatment initiation would not be justifiable.
- Patients will potentially benefit from study treatment (NBHO), i.e. participating in the trial.
- No serious adverse effects of NBHO are to be expected in the PROOF trial (see Section 4.3 Risk-benefit Assessment).
- Obtaining written informed consent prior to NBHO from the patients themselves will delay start of the study treatment and potentially render this study intervention inefficacious or at least significantly reduce its effect on ischemic core growth, in the worst case leading to a negative trial.

Therefore, the investigator is allowed to enroll a patient utilizing emergency informed consent procedures as described below:

1 In case the patient is able to communicate, prior to study enrolment, he/she will be informed orally about the trial. Patients who seem to understand the trial's implications will be asked to give witnessed oral consent, which is documented in the applicable document **("ICF" Variant 1)** by the investigator and a witness; patients objecting trial participation will be documented as Pre-Screening Failure. In case, a LAR is available at this time, he/she has to be informed about the trial orally and in writing and written informed consent has to be obtained **(ICF Variant 2).**

2 If the patient is unable to communicate, this must be documented in the applicable document **("ICF" variant 1)** by the investigator.
   a. In case, a LAR is available and present, he/she has to be informed about the trial orally and in writing and written informed consent has to be obtained **(ICF Variant 2).**
   b. In case, no LAR is available at the time of trial inclusion, the patient may then be included in the trial ("deferred consent"), but the investigator, however, should – if possible – consider the patient's presumed will (e.g. by asking a close relative accompanying the patient) when enrolling a patient following this deferred consent procedure. In case, a patient is included by deferred consent, the investigator has to fill out the "assessment of patient's ability to consent form" **("ICF" variant 1),** which also has to be signed by a witness**.**

As soon as possible, the patient/LAR must be informed and his/her consent is requested for the continuation of the patient's trial participation. In case the patient is not able to give consent, the appointment of an LAR must be initiated within 72 hours after randomization. In case the patient/LAR does not consent, no further trial specific investigations can be performed and no further trial data can be obtained (also refer to Section

7.7.1 Withdrawal of patients from the Trial). In this case, the patient or LAR shall be asked whether he/she allows the use of data acquired so far and his/her decision has to be documented on the applicable ICF).

Blood samples for biomarker analyses will be stored at the respective study site until informed consent is obtained. In case of consent to the participation in the biomarker sub-study (as part of the informed consent form of the main study) the biomarker samples will be shipped to the biomarker core lab (see Section 10.19). In case the patient / LAR does not consent to the participation in the biomarker sub-study, already drawn biomarker samples must be discarded.

In case the patient has been included by deferred consent procedure but dies before installation of an LAR, the patient's relatives (or if applicable the designated (planned) LAR) should be asked whether the data acquired so far might be used (**"ICF" variant 5**).

The following procedure has to be performed as soon as the patient is able to give consent again or a LAR is available:

<u>1. Consent by the study participant (**ICF Variant 3**):</u>
The Investigator will obtain a freely given written consent from each subject after an appropriate explanation of the aims, methods, anticipated benefits, potential hazards and any other aspect of the study which is relevant to the subject's decision to participate. The informed consent form must be signed, with name and date noted by the subject, before the subject is exposed to any further study-related procedure. If the patient is unable to write, oral presentation and explanation of the content of the informed consent form and of the data protection information must take place in the presence of a witness. The witness and the physician conducting the informed consent discussions must also sign and personally date the consent document.

The witness might be any employee of the trial site who is not part of the study team (i.e. not listed in the delegation log) or any other person not working at the trail site.

<u>2. Consent by the LAR:</u>
For patients, not able to give consent, written consent must be obtained from the LAR (**ICF Variant 2** in case the LAR is available and present before randomization or **ICF Variant 4** in case of "deferred consent"). The investigator will perform an appropriate explanation of the aims, methods, anticipated benefits, potential hazards and any other aspect of the study, which is relevant to the LAR's decision. The informed consent form (LAR version) must be signed, with name and date noted by the LAR, before the subject is exposed to any (further) study-related procedure.
As soon as the patient is able to give consent at his/her own, the informed consent procedure has to be repeated and the consent form must be signed and dated by the patient.

After reading the informed consent document, the patient/LAR must give consent in writing. The patient's/LAR's consent must be confirmed by the personally dated signature of the patient/LAR and by the personally dated signature of the physician conducting the informed consent discussion.

A copy of the signed informed consent document must be given to the patient/LAR; the original will be filed by the investigator. The documents must be in a language understandable to the patient/LAR and must specify who informed the patient/LAR.

The patient/LAR will be informed as soon as possible if new information may influence his/her decision to participate in the trial. The communication of this information should be documented.

The patient/LAR should have the opportunity to consult the investigator, or a physician member of the investigating team about the details of the clinical trial. The informed consent to participate in the clinical trial may be withdrawn by the patient/LAR verbally in the presence of, or in written form directed to, the investigator or a physician member of the investigating team at any time during the trial. The patient must not entail any disadvantage therefore or be coerced or unduly influenced to continue to participate. Furthermore, the patient/LAR is not obligated to disclose reasons for the withdrawal of the consent.

If the patient has a primary physician, the investigator should inform him or her about the patient's participation in the trial, provided the patient/LAR agrees hereto.

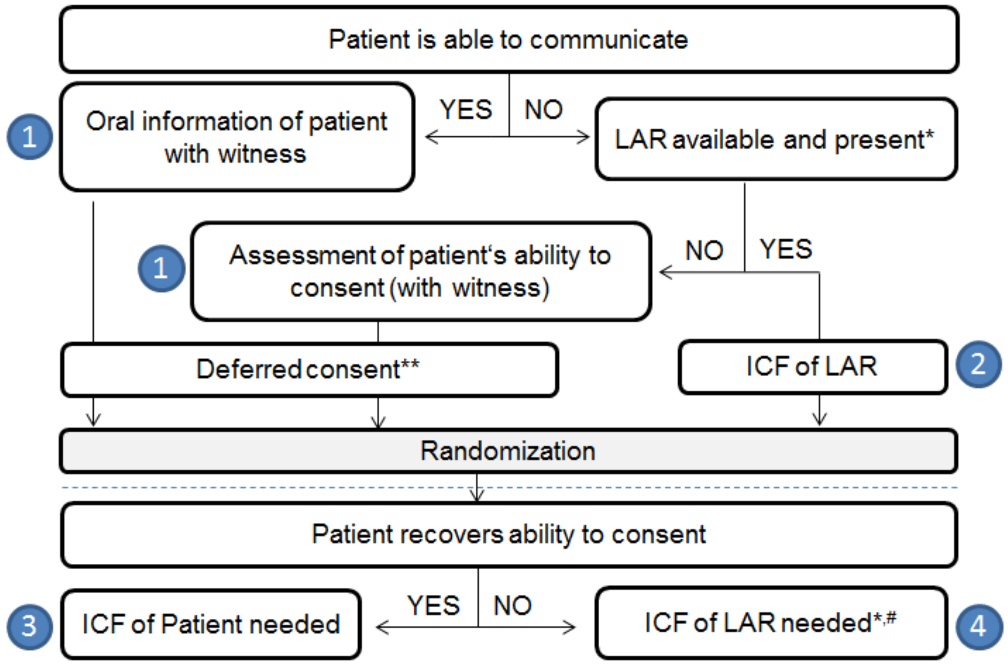


*In case the patient is not able to give consent and no LAR has been appointed so far, the appointment of an LAR must be initiated within 72 hours after randomization.
** In case the patient has been included via deferred consent and the patient / LAR later declines participation, he or she has to be asked whether the data acquired so far might be used
# In case the patient dies before an LAR could be appointed, the patient's relatives (or the planned LAR) should be asked whether the data acquired so far might be used (5)

Numbers in circles indicate the ICF variant to be used;
LAR Legally Authorized Representative; ICF Informed Consent Form

**Figure 9:** Informed consent procedure

## 14.6 Insurance

Prior to the start of the trial the sponsor has to subscribe to an insurance policy covering, in its terms and provisions, its legal liability for injuries caused to participating persons and arising out of this research performed strictly in accordance with the scientific protocol as well as with applicable laws and regulations in each country where the trial is conducted and according to professional standards.

Any impairment of health, which might occur in consequence of trial participation, must be notified to the insurance company. The subject is responsible for notification. The insured person will agree with all appropriate measures serving for clarification of the cause and the extent of damage as well as the reduction of damage.

During the conduct of the trial, the subject must not undergo other clinical treatment except for cases of emergency. The subject is bound to inform the investigator immediately about any adverse events and drugs additionally taken. The terms and conditions of the insurance should be delivered to the subject.

The insurance company has to be informed about all amendments that could affect subjects' safety.

## 14.7 Continuous Information to the Ethics Committee and the Competent Authority

The responsible EC, the competent authorities and all participating investigators will be informed of all suspected unexpected serious adverse reactions (SUSARs) occurring during the trial. Both institutions will be informed in case the risk/ benefit assessment did change or any other new and significant hazards for subjects' safety or welfare did occur. Furthermore, a report on the subject's safety will be submitted once a year – Development Safety Update Report (DSUR).

The EC and the regulatory authorities must be informed of the end of the trial. They will be provided with a summary of trial results within one year after the end of clinical phase (LSO) or within the time frame required according to local law of participating countries.

# 15 QUALITY CONTROL AND QUALITY ASSURANCE

The sponsor, the investigators, and all involved study personnel agree to conduct this clinical trial in accordance with the ICH Guideline for Good Clinical Practice.

## 15.1 Direct Access to Source Documents According to ICH GCP

According to ICH-GCP the investigator(s)/institution(s) must provide direct access to source data/documents for trial related monitoring, audits and regulatory inspection. Each subject has consented - via written informed consent - to direct access to his/her original medical records for trial-related monitoring, audit and regulatory inspection. Content of the protocol must be the identification of any data to be recorded directly on the eCRFs (i.e., no prior written or electronic record of data), and to be considered to be source data (see Section 13.1 Data Collection).
In the absence of either an audit-trail or limited access for the monitor the electronic record of data must be printed out.

## 15.2 Data Protection

The data obtained in the study will be treated pursuant to the General Data Protection Regulation (EU 2016/679). During the clinical trial, subjects will be identified solely by means of their individual identification code (Patient ID). Trial data stored on a computer will be stored in accordance with local data protection law and will be handled in strictest confidence. Distribution of these data to unauthorized persons has to be prevented strictly. The appropriate regulations of local data legislation will be fulfilled in its entirety.

The subject consents in writing to release the investigator from his/her professional discretion in so far as to allow inspection of original data for monitoring purposes by health authorities and authorized persons (inspectors, clinical monitors, auditors). Authorized persons (inspectors, clinical monitors, auditors) may inspect the subject-related data collected during the trial ensuring the appropriate effective data protection law.

The investigator will maintain a subject identification list (subject numbers with the corresponding subject names) to enable records to be identified. Subjects who did not consent (see Section 14.5 Subject Information and Informed Consent) to circulate their pseudonymized data will not be included into the trial.

This protocol, the eCRF and other trial-related documents and material must be handled with strict confidentiality and not be disclosed to third parties except with the express prior consent of Sponsor. In particular, it must be ensured that the study medication is kept out of reach of third parties. Staffs of the investigators involved in this study are also bound by this agreement.

## 15.3 Monitoring

Monitoring will be done by on-site and off-site visits and frequent communication (letters, telephone, fax, e-mail) by a clinical research associate (CRA) according to SOPs of the KKS. The CRA will ensure that the trial is conducted according to the protocol and regulatory requirements by review of source documents, entries into the eCRF and essential documents. Therefore, the investigator must allow the CRA to verify these documents (compare Section 15.1 Direct Access to Source Documents According to ICH GCP) and must provide support to the CRA at all times. The CRA will document the visits in a report for the sponsor. The site will be provided with a follow-up letter of the findings and the necessary actions to be taken.

As the monitoring strategy will consider current aspects of risk-based quality management,
frequency and details of monitoring as well as central procedures in context of the risk-based quality management and the rationale for the chosen monitoring strategy will be defined by all participating functions such as data manager, biometrician, CRA, project manager, safety officer and safety assistant and the sponsor and described in applicable manuals (e.g. monitoring manual, data management plan and further manuals (if necessary)).
If there are major findings during monitoring or an audit, the investigational site might be closed by the Sponsor Representative.

Remote monitoring: Should in-person monitoring visits not be feasible due to regulatory restrictions (such as current Covid-19 pandemic related restrictions), either a combined remote and on-site monitoring visit

or a full remote monitoring visit may be conducted alternatively. Remote source data verification as part of remote monitoring visits needs to be agreed upon between the sponsor, the national sponsor representative and the study site, as well as patients in the ICF. Trial documents (e.g. monitoring plan) will be adjusted to reflect remote activities. The ICH GCP requirements and applicable data protection and privacy regulations must be met in any case and for any selected monitoring approach. In case of a remote verification of source data, the patients need to agree to it in the informed consent form.

## 15.4 Inspections and Audits

Regulatory authorities and/ or auditors authorized by the sponsor may request access to all source documents, CRFs, and other trial documentation. Direct access to these documents must be guaranteed by the investigator who must provide support at all times for these activities.
The investigator will inform the sponsor immediately about a planned inspection.

## 15.5 Responsibilities of the Investigator

The investigator ensures that all team members are informed adequately about the protocol, all amendments to the protocol, the study procedures und study specific duties and tasks.

The investigator will maintain a list to delegate tasks to the team members.

# 16 ADMINISTRATIVE AGREEMENTS

## 16.1 Financing of the Trial

The trial will be financed using funds of the SC1-PM-09-2016 grant by the European Commission within the Horizon 2020 program.

## 16.2 Financial Disclosure

Before the start of the trial, the investigator will disclose to the sponsor any proprietary or financial interests he or she might hold in the sponsor, the funding institution, the investigational product(s) or any commercial organization being involved in the clinical trial. The investigator has also to confirm that he/she has not entered into any financial arrangement, whereby the value of compensation paid could affect the outcome of the clinical trial.

The investigator agrees to update this information in case of significant changes.

## 16.3 Reports

After conclusion of the trial (see Section 6.2 Trial Duration and Schedule), a report (or alternatively the publication) shall be written by the sponsor's delegate, the coordinating investigator and / or principal investigators. The report will include a statistical analysis and an appraisal of the results from a medical viewpoint. It will be based on the items listed in this trial protocol. The KKS Heidelberg will prepare the biometrical part of this report.
Within the defined timeframe (e.g. for Germany within one year after completion of the trial (trial end is defined as last subject out, see Section 6.2 Trial Duration and Schedule)) the competent authorities and the ethics committees will be supplied with this final report or a summary of the final report containing the principle results. Dependent on national regulations the trial report will be published in a clinical trial register via the competent authority. By signing this protocol, the investigators agree to disclose their names/ clinic address in the trial report.

## 16.4 Registration of the Trial

Prior to the beginning of the clinical phase (FPI) the coordinating investigator will register the trial at http://www.clinicaltrials.gov.Thus the trial will be given a unique ISRCTN, which is a prerequisite for a publication in a peer-review paper. If further registrations are necessary according to local requirements each trial site will be responsible thereof.

## 16.5 Publication

All information concerning the trial is confidential before publication. The publication strategy is described in a separate Consortium Agreement. The sponsor is responsible to strive for publication of the results, irrespective of the outcome.

## 16.6 Information of Patients About Trial Results

In addition to any scientific publication, a comprehensible report of the main trial results and, in case of clinical relevance, the results of the bio marker substudy will be provided to all study participants when they become available.

## 17 SIGNATURES

The present trial protocol was subject to critical review and has been approved in the present version by the persons undersigned. The containing information is consistent with:

- the current risk-benefit assessment of the investigational medicinal product,
- the moral, ethical, and scientific principles governing clinical research as set out in the latest relevant version of Declaration of Helsinki, the principles of the guidelines of ICH Good Clinical Practices and the applicable legal and regulatory requirements.

The investigator will be supplied with details of any significant or new finding including AEs relating to treatment with the investigational medicinal product.

It will be ensured that the first subject is enrolled only after all ethical and regulatory requirements are fulfilled. Written consent from all subjects or witness if subject can only consent orally is received after detailed oral and written information and according to the requirements of local law. All study participants will be informed on the type of encoding their personal data (pseudo-anonymization) and who receives or has access to such data. Subjects who do not agree to this data encoding and transfer will not be enrolled into the trial. In this context, it will be assured that all investigational sites comply with the local regulatory requirements for data protection.

No subjects in a relationship of any dependence to the investigator or sponsor will be included.

Via current versions of the clinical trial protocol and the SmPC it will be ensured that all principal investigators are informed about the pharmacological-toxicological assessments and results regarding the benefits and risks of the clinical trial.

| | | | |
|---|---|---|---|
| Date: | 18.03.2021 | Signature: | [signature] |
| | | Name (block letters): | PD Dr. med. Sven Poli |
| | | Function: | Sponsor Representative<br>delegated to the PI/LKP by internal IIT contract |
| Date: | 18.03.2021 | Signature: | [signature] |
| | | Name (block letters): | PD Dr. med. Sven Poli |
| | | Function: | Coordinating investigator<br>(LKP according to §40 AMG) |
| Date: | 2021-03-19 | Signature: | [signature] |
| | | Name (block letters): | Dr. rer. medic. Johannes Hüsing |
| | | Function: | Biometrician |

# 18 DECLARATION OF INVESTIGATOR

I have read the above trial protocol and confirm that it contains all information to properly conduct the clinical trial. I pledge to conduct the clinical trial according to the protocol.

I will enroll the first subject only after all ethical and regulatory requirements are fulfilled. I will obtain written consent for trial participation from all subjects or witness if subject can only consent orally after detailed oral and written information and according to the requirements of local law. All study participants will be informed on the type of encoding their personal data (pseudo-anonymization) and who receives or has access to such data. Subjects who do not agree to this data encoding and transfer will not be enrolled into the trial. In this context, I confirm that my investigational site complies with all local regulatory requirements for data protection.

Furthermore, I declare that to the best of my knowledge no subjects in a relationship of any dependence to the investigator or sponsor will be included.

I know the requirements for accurate notification of serious adverse events and I will document and notify such events as described in the protocol.

I declare that I am informed about the pharmacological-toxicological assessments and results regarding the benefits and risks of the clinical trial by reading the description in the clinical trial protocol and in the current version of the SmPC. I ensure that all investigators/ relevant staff at my site will be informed of this results and possibly new risks that are forwarded by the sponsor later on (e.g. via new version of the SmPC).

I confirm that every staff will be adequately trained to guaranty compliance to the trial protocol incl. subsequent amendments.

I will retain all trial-related documents and source data as described. I will provide a current Curriculum Vitae (CV) before the start of the trial. I agree that the CV and Financial Disclosure (FD) may be submitted to the responsible EC.

As the clinical trial and the results have to be published in a clinical trial register and forwarded to the competent authorities, I agree that my name and clinic address will be part of this final trial (summary) report / public register and are disclosed for that purpose.

| | | | |
| --- | --- | --- | --- |
| Date: | ____________________ | Signature: | ___________________________ |
| | | Name (block letters): | ___________________________ |
| | | Function: | Principal Investigator (PI) |
| | | Investigational Site (address): | ___________________________ |
| | | | ___________________________ |
| | | | ___________________________ |
| Date: | ____________________ | Signature: | ___________________________ |
| | | Name (block letters): | ___________________________ |
| | | Function: | Deputy of the PI |

## 19 REFERENCES

**References**


1. Purrucker, J.C., et al., *Design and validation of a clinical scale for prehospital stroke recognition, severity grading and prediction of large vessel occlusion: the shortened NIH Stroke Scale for emergency medical services.* BMJ Open, 2017. **7**(9): p. e016893.
2. Emberson, J., et al., *Effect of treatment delay, age, and stroke severity on the effects of intravenous thrombolysis with alteplase for acute ischaemic stroke: a meta-analysis of individual patient data from randomised trials.* Lancet, 2014. **384**(9958): p. 1929-35.
3. Goyal, M., et al., *Endovascular thrombectomy after large-vessel ischaemic stroke: a meta-analysis of individual patient data from five randomised trials.* Lancet, 2016.
4. Derrick A. Bennett, et al., *The Global Burden of Ischemic Stroke - Findings of the GBD 2010 Study.* Global Heart, 2014. **9**(1): p. 107-112.
5. Nichols M, et al., *European Cardiovascular Disease Statistics 2012.* European Heart Network, 2012.
6. Poli, S. and R. Veltkamp, *Oxygen Therapy in Acute Ischemic Stroke - Experimental Efficacy and Molecular Mechanisms.* Current Molecular Medicine, 2009. **9**(2): p. 227-241.
7. Dirnagl, U., C. Iadecola, and M.A. Moskowitz, *Pathobiology of ischaemic stroke: an integrated view.* Trends Neurosci, 1999. **22**(9): p. 391-7.
8. Hossmann, K.A., *Viability thresholds and the penumbra of focal ischemia.* Ann Neurol, 1994. **36**(4): p. 557-65.
9. Hossmann, K.A., *Periinfarct depolarizations.* Cerebrovasc Brain Metab Rev, 1996. **8**(3): p. 195-208.
10. Hacke, W., et al., *Association of outcome with early stroke treatment: pooled analysis of ATLANTIS, ECASS, and NINDS rt-PA stroke trials.* Lancet, 2004. **363**(9411): p. 768-74.
11. O'Collins, V., et al., *1,026 Experimental Treatments in Acute Stroke.* Ann Neurol, 2006. **59**(3): p. 467-477.
12. Chance, B., et al., *Basic principles of tissue oxygen determination from mitochondrial signals.* Adv Exp Med Biol, 1973. **37A**: p. 277-92.
13. Hempel, F.G., et al., *Oxidation of cerebral cytochrome aa3 by oxygen plus carbon dioxide at hyperbaric pressures.* J Appl Physiol, 1977. **43**(5): p. 873-9.
14. Zauner, A., et al., *Brain oxygenation and energy metabolism: part I-biological function and pathophysiology.* Neurosurgery, 2002. **51**(2): p. 289-301; discussion 302.
15. Nemoto, E.M., et al., *Suppression of cerebral metabolic rate for oxygen (CMRO2) by mild hypothermia compared with thiopental.* J Neurosurg Anesthesiol, 1996. **8**(1): p. 52-9.
16. Nemoto, E.M. and K. Betterman, *Basic physiology of hyperbaric oxygen in brain.* Neurol Res, 2007. **29**(2): p. 116-26.
17. Erecinska, M. and I.A. Silver, *Tissue oxygen tension and brain sensitivity to hypoxia.* Respir Physiol, 2001. **128**(3): p. 263-76.
18. Erecinska, M. and I.A. Silver, *Ions and energy in mammalian brain.* Prog Neurobiol, 1994. **43**(1): p. 37-71.
19. Nemoto, E.M., et al., *Regional brain PO2 after global ischemia in monkeys: evidence for regional differences in critical perfusion pressures.* Stroke, 1979b. **10**(1): p. 44-52.
20. Ito, H., et al., *Database of normal human cerebral blood flow, cerebral blood volume, cerebral oxygen extraction fraction and cerebral metabolic rate of oxygen measured by positron emission tomography with 15O-labelled carbon dioxide or water, carbon

*monoxide and oxygen: a multicentre study in Japan.* Eur J Nucl Med Mol Imaging, 2004. **31**(5): p. 635-43.

21. Nemoto, E.M., et al., *Compartmentation of whole brain blood flow and oxygen and glucose metabolism in monkeys.* J Neurosurg Anesthesiol, 1994. **6**(3): p. 170-4.
22. Astrup, J., *Energy-requiring cell functions in the ischemic brain. Their critical supply and possible inhibition in protective therapy.* J Neurosurg, 1982. **56**(4): p. 482-97.
23. Cater, D.B., C.M. Grigson, and D.A. Watkinson, *Changes of oxygen tension in tumours induced by vasoconstrictor and vasodilator drugs.* Acta radiol, 1962. **58**: p. 401-34.
24. Erdmann, W., J. Heidenreich, and H. Metzger, *[H2-clearance and pO2-measurements in the brain tissue of rats and cats with the same Pt-microelectrode].* Pflugers Arch, 1969. **307**(2): p. R51-2.
25. Leniger-Follert, E., D.W. Lubbers, and W. Wrabetz, *Regulation of local tissue PO2 of the brain cortex at different arterial O2 pressures.* Pflugers Arch, 1975. **359**(1-2): p. 81-95.
26. Nemoto, E.M., S. Frinak, and F. Taylor, *Postischemic brain oxygenation with barbiturate therapy in rats.* Crit Care Med, 1979a. **7**(8): p. 339-45.
27. Borowsky, I.W. and R.C. Collins, *Metabolic anatomy of brain: a comparison of regional capillary density, glucose metabolism, and enzyme activities.* J Comp Neurol, 1989. **288**(3): p. 401-13.
28. Klein, B., et al., *Interdependency of local capillary density, blood flow, and metabolism in rat brains.* Am J Physiol, 1986. **251**(6 Pt 2): p. H1333-40.
29. Shinozuka, T., E.M. Nemoto, and P.M. Winter, *Mechanisms of cerebrovascular O2 sensitivity from hyperoxia to moderate hypoxia in the rat.* J Cereb Blood Flow Metab, 1989. **9**(2): p. 187-95.
30. Hoedt-Rasmussen, K., E. Sveinsdottir, and N.A. Lassen, *Regional cerebral blood flow in man determined by intra-arterial injection of radioactive inert gas.* Circ Res, 1966. **18**(3): p. 237-47.
31. Kety, S.S., *Regional cerebral blood flow: estimation by means of nonmetabolized diffusible tracers--an overview.* Semin Nucl Med, 1985. **15**(4): p. 324-8.
32. Branston, N.M., et al., *Relationship between the cortical evoked potential and local cortical blood flow following acute middle cerebral artery occlusion in the baboon.* Exp Neurol, 1974. **45**(2): p. 195-208.
33. Matsumoto, K., et al., *Flow thresholds for extracellular purine catabolite elevation in cat focal ischemia.* Brain Res, 1992. **579**(2): p. 309-14.
34. Heiss, W.D., et al., *Which targets are relevant for therapy of acute ischemic stroke?* Stroke, 1999. **30**(7): p. 1486-9.
35. Baron, J.C., *Perfusion thresholds in human cerebral ischemia: historical perspective and therapeutic implications.* Cerebrovasc Dis, 2001. **11 Suppl 1**: p. 2-8.
36. Sundt, T.M., Jr., et al., *Cerebral blood flow measurements and electroencephalograms during carotid endarterectomy.* J Neurosurg, 1974. **41**(3): p. 310-20.
37. Trojaborg, W. and G. Boysen, *Relation between EEG, regional cerebral blood flow and internal carotid artery pressure during carotid endarterectomy.* Electroencephalogr Clin Neurophysiol, 1973. **34**(1): p. 61-9.
38. Zauner, A., et al., *Glutamate release and cerebral blood flow after severe human head injury.* Acta Neurochir Suppl, 1996. **67**: p. 40-4.
39. Nemoto, E.M., et al., *Hyperthermia and hypermetabolism in focal cerebral ischemia.* Adv Exp Med Biol, 2005. **566**: p. 83-9.
40. Watson, J.C., et al., *Real-time detection of vascular occlusion and reperfusion of the brain during surgery by using infrared imaging.* J Neurosurg, 2002. **96**(5): p. 918-23.

41. Dexter, F., et al., *The brain uses mostly dissolved oxygen during profoundly hypothermic cardiopulmonary bypass.* Ann Thorac Surg, 1997. **63**(6): p. 1725-9.
42. Krogh, A., *The Number and Distribution of Capillaries in Muscles with Calculations of the Oxygen Pressure Head Necessary for Supplying the Tissue.* J Physiol, 1919. **52**(6): p. 409–415.
43. Menzel, M., et al., *Increased inspired oxygen concentration as a factor in improved brain tissue oxygenation and tissue lactate levels after severe human head injury.* J Neurosurg, 1999. **91**(1): p. 1-10.
44. Mulkey, D.K., et al., *Oxygen measurements in brain stem slices exposed to normobaric hyperoxia and hyperbaric oxygen.* J Appl Physiol, 2001. **90**(5): p. 1887-99.
45. Niklas, A., et al., *Continuous measurements of cerebral tissue oxygen pressure during hyperbaric oxygenation--HBO effects on brain edema and necrosis after severe brain trauma in rabbits.* J Neurol Sci, 2004. **219**(1-2): p. 77-82.
46. Reinert, M., et al., *Effects of cerebral perfusion pressure and increased fraction of inspired oxygen on brain tissue oxygen, lactate and glucose in patients with severe head injury.* Acta Neurochir (Wien), 2003. **145**(5): p. 341-9; discussion 349-50.
47. Sunami, K., et al., *Hyperbaric oxygen reduces infarct volume in rats by increasing oxygen supply to the ischemic periphery.* Crit Care Med, 2000. **28**(8): p. 2831-6.
48. van Hulst, R.A., et al., *Oxygen tension under hyperbaric conditions in healthy pig brain.* Clin Physiol Funct Imaging, 2003. **23**(3): p. 143-8.
49. Demchenko, I.T., et al., *Cerebral blood flow and brain oxygenation in rats breathing oxygen under pressure.* J Cereb Blood Flow Metab, 2005. **25**(10): p. 1288-300.
50. Veltkamp, R., et al., *Hyperbaric oxygen decreases infarct size and behavioral deficit after transient focal cerebral ischemia in rats.* Brain Res, 2000. **853**(1): p. 68-73.
51. Theilen, H., H. Schrock, and W. Kuschinsky, *Gross persistence of capillary plasma perfusion after middle cerebral artery occlusion in the rat brain.* J Cereb Blood Flow Metab, 1994. **14**(6): p. 1055-61.
52. Ejaz, S., et al., *Normobaric hyperoxia markedly reduces brain damage and sensorimotor deficits following brief focal ischaemia.* Brain, 2016. **139**(Pt 3): p. 751-64.
53. Singhal, A.B., *A review of oxygen therapy in ischemic stroke.* Neurol Res, 2007b. **29**(2): p. 173-83.
54. Liu, S., et al., *Electron paramagnetic resonance-guided normobaric hyperoxia treatment protects the brain by maintaining penumbral oxygenation in a rat model of transient focal cerebral ischemia.* J Cereb Blood Flow Metab, 2006. **26**(10): p. 1274-84.
55. Liu, S., et al., *Interstitial pO2 in ischemic penumbra and core are differentially affected following transient focal cerebral ischemia in rats.* J Cereb Blood Flow Metab, 2004. **24**(3): p. 343-9.
56. Shin, H.K., et al., *Normobaric hyperoxia improves cerebral blood flow and oxygenation, and inhibits peri-infarct depolarizations in experimental focal ischaemia.* Brain, 2007. **130**(Pt 6): p. 1631-42.
57. Takano, T., et al., *Cortical spreading depression causes and coincides with tissue hypoxia.* Nat Neurosci, 2007. **10**(6): p. 754-62.
58. Günther, A., et al., *Early biochemical and histological changes during hyperbaric or normobaric reoxygenation after in vitro ischaemia in primary corticoencephalic cell cultures of rats.* Brain Res, 2002. **946**(1): p. 130-8.
59. Günther, A., et al., *Hyperbaric and normobaric reoxygenation of hypoxic rat brain slices--impact on purine nucleotides and cell viability.* Neurochem Int, 2004. **45**(8): p. 1125-32.

60. Sun, L., et al., *Oxygen therapy improves energy metabolism in focal cerebral ischemia.* Brain Res, 2011. **1415**: p. 103-8.
61. Singhal, A.B., et al., *Magnetic resonance spectroscopy study of oxygen therapy in ischemic stroke.* Stroke, 2007. **38**(10): p. 2851-4.
62. Henninger, N., et al., *Normobaric hyperoxia delays perfusion/diffusion mismatch evolution, reduces infarct volume, and differentially affects neuronal cell death pathways after suture middle cerebral artery occlusion in rats.* J Cereb Blood Flow Metab, 2007. **27**(9): p. 1632-42.
63. Singhal, A.B., et al., *Effects of normobaric hyperoxia in a rat model of focal cerebral ischemia-reperfusion.* J Cereb Blood Flow Metab, 2002b. **22**(7): p. 861-8.
64. Liang, J., et al., *Normobaric hyperoxia slows blood-brain barrier damage and expands the therapeutic time window for tissue-type plasminogen activator treatment in cerebral ischemia.* Stroke, 2015. **46**(5): p. 1344-51.
65. Sun, L., et al., *Oxygen therapy reduces secondary hemorrhage after thrombolysis in thromboembolic cerebral ischemia.* J Cereb Blood Flow Metab, 2010. **30**(9): p. 1651-60.
66. Singhal, A.B., et al., *Normobaric hyperoxia reduces MRI diffusion abnormalities and infarct size in experimental stroke.* Neurology, 2002a. **58**(6): p. 945-52.
67. Esposito, E., et al., *Effects of normobaric oxygen on the progression of focal cerebral ischemia in rats.* Exp Neurol, 2013. **249**: p. 33-8.
68. Kim, H.Y., A.B. Singhal, and E.H. Lo, *Normobaric hyperoxia extends the reperfusion window in focal cerebral ischemia.* Ann Neurol, 2005. **57**(4): p. 571-5.
69. Sun, L., G. Wolferts, and R. Veltkamp, *Oxygen therapy does not increase production and damage induced by reactive oxygen species in focal cerebral ischemia.* Neurosci Lett, 2014. **577**: p. 1-5.
70. Weaver, J. and K.J. Liu, *Does normobaric hyperoxia increase oxidative stress in acute ischemic stroke? A critical review of the literature.* Med Gas Res, 2015. **5**: p. 11.
71. Flynn, E.P. and R.N. Auer, *Eubaric hyperoxemia and experimental cerebral infarction.* Ann Neurol, 2002. **52**(5): p. 566-72.
72. Jin, X., et al., *Normobaric hyperoxia combined with minocycline provides greater neuroprotection than either alone in transient focal cerebral ischemia.* Exp Neurol, 2013. **240**: p. 9-16.
73. Roffe, C., et al., *The stroke oxygen supplementation (SO2S) study: a multicentre, prospective, randomised, open, blinded-endpoint study of routine oxygen supplementation in the first 72 hours after stroke*, in *European Stroke Conference*. 2014: Nice.
74. Ronning, O.M. and B. Guldvog, *Should stroke victims routinely receive supplemental oxygen? A quasi-randomized controlled trial.* Stroke, 1999. **30**(10): p. 2033-7.
75. Chiu, E.H., et al., *Venturi mask adjuvant oxygen therapy in severe acute ischemic stroke.* Arch Neurol, 2006. **63**(5): p. 741-4.
76. Singhal, A.B., et al., *A pilot study of normobaric oxygen therapy in acute ischemic stroke.* Stroke, 2005. **36**(4): p. 797-802.
77. Samson, K., *News From the AAN Annual Meeting: Why a Trial of Normobaric Oxygen in Acute Ischemic Stroke Was Halted Early.* Neurol Today, 2013. **13**(10): p. 34-35.
78. Padma, M.V., *Normobaric oxygen therapy in acute ischemic stroke: A pilot study in Indian patients.* Annals of the Indian Academy of Neurology, 2010. **13**(4): p. 284-288.
79. Floyd, T.F., et al., *Independent cerebral vasoconstrictive effects of hyperoxia and accompanying arterial hypocapnia at 1 ATA.* J Appl Physiol (1985), 2003. **95**(6): p. 2453-61.

80. Alexandrov, A.V., et al., *Reversed Robin Hood syndrome in acute ischemic stroke patients.* Stroke, 2007. **38**(11): p. 3045-8.
81. Wu, O., et al., *Dynamic functional cerebral blood volume responses to normobaric hyperoxia in acute ischemic stroke.* J Cereb Blood Flow Metab, 2012. **32**(9): p. 1800-9.
82. Lassen, N.A. and R. Palvolgyi, *Cerebral steal during hypercapnia and the inverse reaction during hypocapnia observed by the 133 xenon technique in man.* Scand J Clin Lab Invest Suppl, 1968. **102**: p. XIII:D.
83. Bai, Q., et al., *Susceptibility-weighted imaging for cerebral microbleed detection in super-acute ischemic stroke patients treated with intravenous thrombolysis.* Neurol Res, 2013. **35**(6): p. 586-93.
84. Shi, Z.S., et al., *New Cerebral Microbleeds After Mechanical Thrombectomy for Large-Vessel Occlusion Strokes.* Medicine (Baltimore), 2015. **94**(47): p. e2180.
85. Fujiwara, N., et al., *Combination therapy with normobaric oxygen (NBO) plus thrombolysis in experimental ischemic stroke.* BMC Neurosci, 2009. **10**: p. 79.
86. Henninger, N., et al., *Normobaric hyperoxia and delayed tPA treatment in a rat embolic stroke model.* J Cereb Blood Flow Metab, 2009. **29**(1): p. 119-29.
87. Young, P., *The association between early arterial oxygenation and mortality in ventilated patients with acute ischaemic stroke.* Crit Care Resusc, 2012. **14**(1): p. 14-19.
88. Rincon, F., *Association between hyperoxia and mortality after stroke: a multicenter cohort study.* Crit Care Med, 2014. **42**(2): p. 387-396.
89. Ali, K., et al., *The stroke oxygen pilot study: a randomized controlled trial of the effects of routine oxygen supplementation early after acute stroke--effect on key outcomes at six months.* PLoS One, 2014. **8**(6): p. e59274.
90. Roffe, C., et al., *Effect of Routine Low-Dose Oxygen Supplementation on Death and Disability in Adults With Acute Stroke.* JAMA, 2017. **318**(12): p. 1125-35.
91. Widimsky, P., R. Coram, and A. Abou-Chebl, *Reperfusion therapy of acute ischaemic stroke and acute myocardial infarction: similarities and differences.* Eur Heart J, 2014. **35**(3): p. 147-55.
92. Cabello, J.B., et al., *Oxygen therapy for acute myocardial infarction.* Cochrane Database Syst Rev, 2016. **12**: p. CD007160.
93. Khoshnood, A., et al., *Effects of oxygen therapy on wall-motion score index in patients with ST elevation myocardial infarction-the randomized SOCCER trial.* Echocardiography, 2017. **34**(8): p. 1130-1137.
94. Khoshnood, A., et al., *Effect of oxygen therapy on myocardial salvage in ST elevation myocardial infarction: the randomized SOCCER trial.* Eur J Emerg Med, 2016.
95. Hofmann, R., et al., *Oxygen Therapy in Suspected Acute Myocardial Infarction.* N Engl J Med, 2017. **377**(13): p. 1240-1249.
96. Smith, J.L., *The pathological effects due to increase of oxygen tension in the air breathed.* J Physiol, 1899. **24**(1): p. 19-35.
97. Linde, *Conoxia® Medical Oxygen Fachinformation (SmPC)*. 2014. p. 1-3.
98. Bert, P., *Barometric pressure - Researches in Experimental Physiology*. 1943, Columbus, Ohio: College Book Company.
99. Terry, T.L., *Retrolental Fibroplasia in the Premature Infant: V. Further Studies on Fibroplastic Overgrowth of the Persistent Tunica Vasculosa Lentis.* Trans Am Ophthalmol Soc, 1944. **42**: p. 383-96.
100. Ashton, N., B. Ward, and G. Serpell, *Effect of oxygen on developing retinal vessels with particular reference to the problem of retrolental fibroplasia.* Br J Ophthalmol, 1954. **38**(7): p. 397-432.

101. von Kummer, R., et al., *The Heidelberg Bleeding Classification: Classification of Bleeding Events After Ischemic Stroke and Reperfusion Therapy.* Stroke, 2015. **46**(10): p. 2981-6.
102. Stewart, R.A.H., et al., *High flow oxygen and risk of mortality in patients with a suspected acute coronary syndrome: pragmatic, cluster randomised, crossover trial.* BMJ, 2021. **372**: p. n355.
103. Goyal, M., et al., *Endovascular thrombectomy after large-vessel ischaemic stroke: a meta-analysis of individual patient data from five randomised trials.* Lancet, 2016. **387**(10029): p. 1723-31.
104. Singhal, A.B., et al., *Normobaric hyperoxia reduces MRI diffusion abnormalities and infarct size in experimental stroke.* Neurology, 2002. **58**(6): p. 945-52.
105. Baron, J.C., *Protecting the ischaemic penumbra as an adjunct to thrombectomy for acute stroke.* Nat Rev Neurol, 2018. **14**(6): p. 325-337.
106. Nogueira, R.G., et al., *Thrombectomy 6 to 24 Hours after Stroke with a Mismatch between Deficit and Infarct.* N Engl J Med, 2018. **378**(1): p. 11-21.
107. Albers, G.W., et al., *Thrombectomy for Stroke at 6 to 16 Hours with Selection by Perfusion Imaging.* N Engl J Med, 2018. **378**(8): p. 708-718.
108. Shi, S., et al., *Normobaric Hyperoxia Reduces Blood Occludin Fragments in Rats and Patients With Acute Ischemic Stroke.* Stroke, 2017. **48**(10): p. 2848-2854.
109. Rha, J.H. and J.L. Saver, *The impact of recanalization on ischemic stroke outcome: a meta-analysis.* Stroke, 2007. **38**(3): p. 967-73.
110. Singhal, A.B., *Normobaric Oxygen Therapy in Acute Ischemic Stroke Trial.* ClinicalTrials.gov Identifier: NCT00414726, unpublished.
111. Chu, D.K., et al., *Mortality and morbidity in acutely ill adults treated with liberal versus conservative oxygen therapy (IOTA): a systematic review and meta-analysis.* Lancet, 2018. **391**(10131): p. 1693-1705.
112. Grensemann, J., V. Fuhrmann, and S. Kluge, *Oxygen Treatment in Intensive Care and Emergency Medicine.* Dtsch Arztebl Int, 2018. **115**(27-28): p. 455-462.
113. Stolmeijer, R., et al., *A Systematic Review of the Effects of Hyperoxia in Acutely Ill Patients: Should We Aim for Less?* Biomed Res Int, 2018. **2018**: p. 7841295.
114. Shi, S.H., et al., *Normobaric oxygen treatment in acute ischemic stroke: a clinical perspective.* Med Gas Res, 2016. **6**(3): p. 147-153.
115. Stub, D., et al., *Air Versus Oxygen in ST-Segment-Elevation Myocardial Infarction.* Circulation, 2015. **131**(24): p. 2143-50.
116. Nehme, Z., et al., *Effect of supplemental oxygen exposure on myocardial injury in ST-elevation myocardial infarction.* Heart, 2016. **102**(6): p. 444-51.
117. Albers, G.W., et al., *Relationships Between Imaging Assessments and Outcomes in Solitaire With the Intention for Thrombectomy as Primary Endovascular Treatment for Acute Ischemic Stroke.* Stroke, 2015. **46**(10): p. 2786-94.
118. Chan, Y.F., et al., *Supplemental oxygen delivery to suspected stroke patients in pre hospital and emergency department settings.* Med Gas Res, 2014. **4**: p. 16.
119. Fujiwara, N., et al., *Effect of normobaric oxygen therapy in a rat model of intracerebral hemorrhage.* Stroke, 2011. **42**(5): p. 1469-72.
120. Poli, S., et al., *Normobaric hyperoxygenation: a potential neuroprotective therapy for acute ischemic stroke?* Expert Rev Neurother, 2017. **17**(12): p. 1131-1134.
121. Holodinsky, J.K., et al., *Decreasing Door-to-Reperfusion Times.* Endovascular Today, 2017. **16**(1): p. 3.

122. Ali, K., *What patients want: consumer involvement in the design of a randomized controlled trial of routine oxygen supplementation after acute stroke.* Stroke, 2006. **37**(3): p. 865-871.
123. Gray, L.J., et al., *Interconversion of the National Institutes of Health Stroke Scale and Scandinavian Stroke Scale in acute stroke.* J Stroke Cerebrovasc Dis, 2009. **18**(6): p. 466-8.
124. Khoshnood, A., et al., *Effect of oxygen therapy on myocardial salvage in ST elevation myocardial infarction: the randomized SOCCER trial.* Eur J Emerg Med, 2018. **25**(2): p. 78-84.
125. Roffe, C., et al., *A randomized controlled trial of the effect of fixed-dose routine nocturnal oxygen supplementation on oxygen saturation in patients with acute stroke.* J Stroke Cerebrovasc Dis, 2010. **19**(1): p. 29-35.
126. Wilson, A.T. and K.S. Channer, *Hypoxaemia and supplemental oxygen therapy in the first 24 hours after myocardial infarction: the role of pulse oximetry.* J R Coll Physicians Lond, 1997. **31**(6): p. 657-61.
127. McEvoy, J.W., *Excess oxygen in acute illness: adding fuel to the fire.* Lancet, 2018. **391**(10131): p. 1640-1642.
128. Kapadia, V.S., et al., *Perinatal asphyxia with hyperoxemia within the first hour of life is associated with moderate to severe hypoxic-ischemic encephalopathy.* J Pediatr, 2013. **163**(4): p. 949-54.
129. Felderhoff-Mueser, U., et al., *Oxygen causes cell death in the developing brain.* Neurobiol Dis, 2004. **17**(2): p. 273-82.
130. Tiwari, Y.V., et al., *Effects of stroke severity and treatment duration in normobaric hyperoxia treatment of ischemic stroke.* Brain Res, 2016. **1635**: p. 121-9.
131. Baskerville, T.A., et al., *Influence of 100% and 40% oxygen on penumbral blood flow, oxygen level, and T2*-weighted MRI in a rat stroke model.* J Cereb Blood Flow Metab, 2011. **31**(8): p. 1799-806.
132. Hofmann, R., L. Svensson, and S.K. James, *Oxygen Therapy in Suspected Acute Myocardial Infarction.* N Engl J Med, 2018. **378**(2): p. 201-202.
133. Ranchord, A.M., et al., *High-concentration versus titrated oxygen therapy in ST-elevation myocardial infarction: a pilot randomized controlled trial.* Am Heart J, 2012. **163**(2): p. 168-75.
134. Cabello, J.B., et al., *Oxygen therapy for acute myocardial infarction.* Cochrane Database Syst Rev, 2013(8): p. CD007160.
135. Ukholkina, G.B., et al., *[Effect of oxygenotherapy used in combination with reperfusion in patients with acute myocardial infarction].* Kardiologiia, 2005. **45**(5): p. 59.
136. Rawles, J.M. and A.C. Kenmure, *Controlled trial of oxygen in uncomplicated myocardial infarction.* Br Med J, 1976. **1**(6018): p. 1121-3.
137. Young, P., et al., *HyperOxic Therapy OR NormOxic Therapy after out-of-hospital cardiac arrest (HOT OR NOT): a randomised controlled feasibility trial.* Resuscitation, 2014. **85**(12): p. 1686-91.
138. Kuisma, M., et al., *Comparison of 30 and the 100% inspired oxygen concentrations during early post-resuscitation period: a randomised controlled pilot study.* Resuscitation, 2006. **69**(2): p. 199-206.
139. Roffe, C., et al., *Effect of Routine Low-Dose Oxygen Supplementation on Death and Disability in Adults With Acute Stroke: The Stroke Oxygen Study Randomized Clinical Trial.* JAMA, 2017. **318**(12): p. 1125-1135.
140. Mazdeh, M., et al., *Effects of Normobaric Hyperoxia in Severe Acute Stroke: a Randomized Controlled Clinical Trial Study.* Acta Med Iran, 2015. **53**(11): p. 676-80.

141. Roffe, C., et al., *The SOS pilot study: a RCT of routine oxygen supplementation early after acute stroke--effect on recovery of neurological function at one week.* PLoS One, 2011. **6**(5): p. e19113.
142. Padma, M.V., et al., *Normobaric oxygen therapy in acute ischemic stroke: A pilot study in Indian patients.* Ann Indian Acad Neurol, 2010. **13**(4): p. 284-8.
143. Gonzalez, R.G., et al., *Stability of large diffusion/perfusion mismatch in anterior circulation strokes for 4 or more hours.* BMC Neurol, 2010. **10**: p. 13.
144. Luo, D., et al., *Optimally estimating the sample mean from the sample size, median, mid-range, and/or mid-quartile range.* Stat Methods Med Res, 2018. **27**(6): p. 1785-1805.
145. Wan, X., et al., *Estimating the sample mean and standard deviation from the sample size, median, range and/or interquartile range.* BMC Med Res Methodol, 2014. **14**: p. 135.
146. Killip, T., 3rd and J.T. Kimball, *Treatment of myocardial infarction in a coronary care unit. A two year experience with 250 patients.* Am J Cardiol, 1967. **20**(4): p. 457-64.
147. Mijajlovic, M.D., et al., *Hyperbaric oxygen therapy in acute stroke: is it time for Justitia to open her eyes?* Neurol Sci, 2020. **41**(6): p. 1381-1390.
148. Cozene, B., et al., *An Extra Breath of Fresh Air: Hyperbaric Oxygenation as a Stroke Therapeutic.* Biomolecules, 2020. **10**(9).
149. Cheng, Z., et al., *Normobaric oxygen therapy attenuates hyperglycolysis in ischemic stroke.* Neural Regen Res, 2021. **16**(6): p. 1017-1023.
150. Akca, O., et al., *Association of Early Oxygenation Levels with Mortality in Acute Ischemic Stroke - A Retrospective Cohort Study.* J Stroke Cerebrovasc Dis, 2020. **29**(2): p. 104556.
151. Cheng, Z., et al., *Adjuvant High-Flow Normobaric Oxygen After Mechanical Thrombectomy for Anterior Circulation Stroke: a Randomized Clinical Trial.* Neurotherapeutics, 2021.
152. Beker, M.C., et al., *Effects of normobaric oxygen and melatonin on reperfusion injury: role of cerebral microcirculation.* Oncotarget, 2015. **6**(31): p. 30604-14.
153. Neuberger, U., et al., *Risk factors of intracranial hemorrhage after mechanical thrombectomy of anterior circulation ischemic stroke.* Neuroradiology, 2019. **61**(4): p. 461-469.
154. Chen, S., et al., *One-year outcomes of supersaturated oxygen therapy in acute anterior myocardial infarction: The IC-HOT study.* Catheter Cardiovasc Interv, 2020.
155. Andell, P., et al., *Oxygen therapy in suspected acute myocardial infarction and concurrent normoxemic chronic obstructive pulmonary disease: a prespecified subgroup analysis from the DETO2X-AMI trial.* Eur Heart J Acute Cardiovasc Care, 2020. **9**(8): p. 984-992.
156. Rangaraju, S., M. Frankel, and T.G. Jovin, *Prognostic Value of the 24-Hour Neurological Examination in Anterior Circulation Ischemic Stroke: A post hoc Analysis of Two Randomized Controlled Stroke Trials.* Interv Neurol, 2016. **4**(3-4): p. 120-9.
157. Ganesh, A., et al., *Infarct in a New Territory After Treatment Administration in the ESCAPE Randomized Controlled Trial (Endovascular Treatment for Small Core and Anterior Circulation Proximal Occlusion With Emphasis on Minimizing CT to Recanalization Times).* Stroke, 2016. **47**(12): p. 2993-2998.
158. Peter A. Ringleb, et al., *Guidelines for Management of Ischaemic Stroke and Transient Ischaemic Attack.* European Stroke Organization, 2008.
159. Turc, G., et al., *European Stroke Organisation (ESO) - European Society for Minimally Invasive Neurological Therapy (ESMINT) Guidelines on Mechanical Thrombectomy in Acute Ischemic Stroke.* J Neurointerv Surg, 2019.

160. Powers, W.J., et al., *Guidelines for the Early Management of Patients With Acute Ischemic Stroke: 2019 Update to the 2018 Guidelines for the Early Management of Acute Ischemic Stroke: A Guideline for Healthcare Professionals From the American Heart Association/American Stroke Association.* Stroke, 2019. **50**(12): p. e344-e418.
161. Taves, D.R., *Minimization: a new method of assigning patients to treatment and control groups.* Clin Pharmacol Ther, 1974. **15**(5): p. 443-53.
162. Atwood, C.W., et al., *Impact of heated humidified high flow air via nasal cannula on respiratory effort in patients with chronic obstructive pulmonary disease.* Chronic Obstr Pulm Dis, 2017. **4**(4): p. 279-286.
163. Moller, W., et al., *Nasal high flow clears anatomical dead space in upper airway models.* J Appl Physiol (1985), 2015. **118**(12): p. 1525-32.
164. Lyden, P., et al., *Underlying structure of the National Institutes of Health Stroke Scale: results of a factor analysis. NINDS tPA Stroke Trial Investigators.* Stroke, 1999. **30**(11): p. 2347-54.
165. Knauth, M., et al., *Potential of CT angiography in acute ischemic stroke.* AJNR Am J Neuroradiol, 1997. **18**(6): p. 1001-10.
166. d'Esterre, C.D., et al., *Regional Comparison of Multiphase Computed Tomographic Angiography and Computed Tomographic Perfusion for Prediction of Tissue Fate in Ischemic Stroke.* Stroke, 2017. **48**(4): p. 939-945.
167. Almekhlafi, M.A., et al., *Imaging Triage of Patients with Late-Window (6-24 Hours) Acute Ischemic Stroke: A Comparative Study Using Multiphase CT Angiography versus CT Perfusion.* AJNR Am J Neuroradiol, 2020. **41**(1): p. 129-133.
168. Lee, S.J., et al., *Optimal Multiphase Computed Tomographic Angiography-based Infarct Core Estimations for Acute Ischemic Stroke.* Sci Rep, 2019. **9**(1): p. 15243.
169. Rankin, J., *Cerebral vascular accidents in patients over the age of 60. II. Prognosis.* Scott Med J, 1957. **2**(5): p. 200-15.
170. van Swieten, J.C., et al., *Interobserver agreement for the assessment of handicap in stroke patients.* Stroke, 1988. **19**(5): p. 604-7.
171. Lopez-Cancio, E., et al., *Phone and Video-Based Modalities of Central Blinded Adjudication of Modified Rankin Scores in an Endovascular Stroke Trial.* Stroke, 2015. **46**(12): p. 3405-10.
172. Cooray, C., et al., *Mobile Phone-Based Questionnaire for Assessing 3 Months Modified Rankin Score After Acute Stroke: A Pilot Study.* Circ Cardiovasc Qual Outcomes, 2015. **8**(6 Suppl 3): p. S125-30.
173. Mahoney, F.I. and D.W. Barthel, *Functional Evaluation: The Barthel Index.* Md State Med J, 1965. **14**: p. 61-5.
174. Korner-Bitensky, N. and S. Wood-Dauphinee, *Barthel Index information elicited over the telephone. Is it reliable?* Am J Phys Med Rehabil, 1995. **74**(1): p. 9-18.
175. Della Pietra, G.L., et al., *Validity and reliability of the Barthel index administered by telephone.* Stroke, 2011. **42**(7): p. 2077-9.
176. Jorm, A.F., *The Informant Questionnaire on cognitive decline in the elderly (IQCODE): a review.* Int Psychogeriatr, 2004. **16**(3): p. 275-93.
177. Nasreddine, Z.S., et al., *The Montreal Cognitive Assessment, MoCA: a brief screening tool for mild cognitive impairment.* J Am Geriatr Soc, 2005. **53**(4): p. 695-9.
178. Lees, R., et al., *Test accuracy of cognitive screening tests for diagnosis of dementia and multidomain cognitive impairment in stroke.* Stroke, 2014. **45**(10): p. 3008-18.
179. Wong, A., et al., *Montreal Cognitive Assessment 5-minute protocol is a brief, valid, reliable, and feasible cognitive screen for telephone administration.* Stroke, 2015. **46**(4): p. 1059-64.

180. Duncan, P.W., et al., *Stroke Impact Scale-16: A brief assessment of physical function.* Neurology, 2003. **60**(2): p. 291-6.
181. Kwon, S., et al., *Measuring stroke impact with SIS: construct validity of SIS telephone administration.* Qual Life Res, 2006. **15**(3): p. 367-76.
182. Golicki, D., et al., *Validity of the Eq-5d-5l in Stroke Patients.* Value Health, 2014. **17**(7): p. A570.
183. Montgomery, S.A. and M. Asberg, *A new depression scale designed to be sensitive to change.* Br J Psychiatry, 1979. **134**: p. 382-9.
184. Chollet, F., et al., *Fluoxetine for motor recovery after acute ischaemic stroke (FLAME): a randomised placebo-controlled trial.* Lancet Neurol, 2011. **10**(2): p. 123-30.
185. Hermens, M.L., et al., *Administering the MADRS by telephone or face-to-face: a validity study.* Ann Gen Psychiatry, 2006. **5**: p. 3.
186. Kobak, K.A., et al., *Face-to-face versus remote administration of the Montgomery-Asberg Depression Rating Scale using videoconference and telephone.* Depress Anxiety, 2008. **25**(11): p. 913-9.
187. Albers, G.W., et al., *Ischemic core and hypoperfusion volumes predict infarct size in SWIFT PRIME.* Ann Neurol, 2016. **79**(1): p. 76-89.
188. Bauer, P. and K. Kohne, *Evaluation of experiments with adaptive interim analyses.* Biometrics, 1994. **50**(4): p. 1029-41.
189. Chaisinanunkul, N., et al., *Adopting a Patient-Centered Approach to Primary Outcome Analysis of Acute Stroke Trials Using a Utility-Weighted Modified Rankin Scale.* Stroke, 2015. **46**(8): p. 2238-43.
190. Higashida, R.T., et al., *Trial design and reporting standards for intra-arterial cerebral thrombolysis for acute ischemic stroke.* Stroke, 2003. **34**(8): p. e109-37.
191. Santos, E.M.M., et al., *Added value of multiphase CTA imaging for thrombus perviousness assessment.* Neuroradiology, 2018. **60**(1): p. 71-79.
192. Chen, Z., et al., *Thrombus Permeability on Dynamic CTA Predicts Good Outcome after Reperfusion Therapy.* AJNR Am J Neuroradiol, 2018. **39**(10): p. 1854-1859.
193. Tan, I.Y., et al., *CT angiography clot burden score and collateral score: correlation with clinical and radiologic outcomes in acute middle cerebral artery infarct.* AJNR Am J Neuroradiol, 2009. **30**(3): p. 525-31.

# 20 APPENDICES

Appendix 1 Results of own meta-analysis of the randomized trials relevant for the potential PROOF study population .......... II

Appendix 2 Statement of the German society for neuro-intensive care and emergency medicine (DGNI) concerning the suitability of mortality as a measure of quality in neuro-intensive treatment, including stroke care .......... XXVII

Appendix 3 Summary of Singhal's Phase IIb clinical trial of NBHO in acute ischemic stroke [ClinicalTrials.gov identifier: NCT00414726], unpublished data .......... XXIX

Appendix 4 National Institutes of Health Stroke Scale (NIHSS) .......... XXXVI

Appendix 5 modified Rankin Scale (mRS) .......... XLIV

Appendix 6 Barthel Index .......... XLV

Appendix 7 Short Form of the Informant Questionnaire on Cognitive Decline in the Elderly (Short IQCODE) .......... XLVI

Appendix 8 Montreal Cognitive Assessment (MOCA) .......... XLIX

Appendix 9 MoCA 5-min protocol .......... L

Appendix 10 Stroke Impact Scale (SIS-16) .......... LI

Appendix 11 EQ-5D-5L .......... LII

Appendix 12 EQ-5D-5L Telephone interview .......... LIV

Appendix 13 Montgomery-Asberg Depression Scale (MADRS) .......... LVIII

Appendix 14 Heidelberg bleeding classification .......... LX

## Appendix 1 Results of own meta-analysis of the randomized trials relevant for the potential PROOF study population

| Any oxygen therapy | All available RCT considered | | | | | | Without NCT00414726 | | | | | |
|---|---|---|---|---|---|---|---|---|---|---|---|---|
| | Studies, n | N | Liberal OT, n | Conservative OT, n | Risk ratio [95% CI] Random | Risk ratio [95% CI] Fixed | Studies, n | N | Liberal OT, n | Conservative OT, n | Risk ratio [95% CI] Random | Risk ratio [95% CI] Fixed |
| **Mortality in-hospital** | | | | | | | | | | | | |
| I: as shown by Chu et al. | 13 | 14101 | 7070 | 7031 | 1.18 [0.96, 1.46] | 1.19 [0.97, 1.47] | 12 | 14016 | 7027 | 6989 | 1.13 [0.91, 1.40] | 1.13 [0.91, 1.40] |
| II: same as I but numbers as reported in original publications, BC/WC | 13 | 16718 | 9694 | 7024 | 1.11 [0.88, 1.42] / 1.15 [0.92, 1.45] | 1.11 [0.91, 1.36] / 1.14 [0.93, 1.39] | 12 | 16633 | 9651 | 6982 | 1.05 [0.86, 1.30] / 1.09 [0.88, 1.34] | 1.06 [0.86, 1.30] / 1.08 [0.88, 1.33] |
| III: all RCT, BC/WC | 18 | 17066 | 9866 | 7200 | 1.08 [0.87, 1.34] / 1.14 [0.94, 1.39] | 1.08 [0.89, 1.31] / 1.14 [0.94, 1.38] | 17 | 16981 | 9823 | 7158 | 1.03 [0.85, 1.26] / 1.09 [0.89, 1.33] | 1.03 [0.84, 1.25] / 1.08 [0.89, 1.32] |
| IV: same as III but stroke only | 9 | 9069 | 5873 | 3196 | 1.14 [0.82, 1.57] | 1.10 [0.85, 1.41] | 8 | 8984 | 5830 | 3154 | 1.02 [0.78, 1.33] | 1.01 [0.77, 1.31] |
| **Mortality at 30 days** | | | | | | | | | | | | |
| I: as shown by Chu et al. | 8 | 13689 | 6862 | 6827 | 1.08 [0.92, 1.27] | 1.08 [0.93, 1.27] | 7 | 13604 | 6819 | 6785 | 1.05 [0.90, 1.24] | 1.05 [0.89, 1.23] |
| II: same as I but numbers as reported in original publications, BC/WC | 8 | 16124 | 9394 | 6730 | 1.05 [0.81, 1.35] / 1.07 [0.85, 1.35] | 0.99 [0.86, 1.15] / 1.00 [0.87, 1.16] | 7 | 16039 | 9351 | 6688 | 0.97 [0.83, 1.12] / 0.98 [0.84, 1.14] | 0.96 [0.83, 1.12] / 0.97 [0.84, 1.13] |
| III: all RCT, BC/WC | 11 | 16375 | 9525 | 6850 | 1.04 [0.83, 1.30] / 1.05 [0.86, 1.28] | 0.99 [0.86, 1.15] / 1.00 [0.87, 1.16] | 10 | 16290 | 9482 | 6808 | 0.97 [0.83, 1.12] / 0.98 [0.84, 1.13] | 0.96 [0.83, 1.11] / 0.97 [0.84, 1.13] |
| IV: same as III but stroke only | 7 | 8997 | 5839 | 3158 | 1.15 [0.81, 1.63] | 0.99 [0.84, 1.18] | 6 | 8912 | 5796 | 3116 | 0.96 [0.81, 1.14] | 0.95 [0.80, 1.13] |
| **Mortality at 90 days** | | | | | | | | | | | | |
| III: all RCT, BC/WC | 10 | 15938 | 9280 | 6658 | 1.04 [0.92, 1.18] | 1.03 [0.92, 1.16] | 9 | 15853 | 9237 | 6616 | 1.02 [0.91, 1.14] | 1.01 [0.90, 1.14] |
| IV: same as III but stroke only | 7 | 8708 | 5666 | 3042 | 1.14 [0.92, 1.42] | 1.05 [0.93, 1.20] | 6 | 8623 | 5623 | 3000 | 1.04 [0.91, 1.18] | 1.03 [0.91, 1.17] |
| **Mortality at longest follow-up** | | | | | | | | | | | | |
| I: as shown by Chu et al. | 16 | 14340 | 7188 | 7152 | 1.06 [0.96, 1.18] | 1.06 [0.95, 1.18] | 15 | 14255 | 7145 | 7110 | 1.05 [0.94, 1.17] | 1.04 [0.94, 1.16] |
| II: same as I but numbers as reported in original publications, BC/WC | 16 | 16457 | 9527 | 6930 | 1.03 [0.93, 1.14] / 1.05 [0.94, 1.16] | 1.03 [0.93, 1.14] / 1.04 [0.94, 1.15] | 15 | 16372 | 9484 | 6888 | 1.02 [0.92, 1.13] / 1.03 [0.93, 1.14] | 1.01 [0.91, 1.12] / 1.02 [0.92, 1.14] |
| III: all RCT, BC/WC | 18 | 16566 | 9581 | 6985 | 1.03 [0.93, 1.14] / 1.04 [0.94, 1.16] | 1.02 [0.92, 1.13] / 1.04 [0.94, 1.15] | 17 | 16481 | 9538 | 6943 | 1.01 [0.92, 1.12] / 1.03 [0.93, 1.14] | 1.01 [0.91, 1.12] / 1.02 [0.92, 1.14] |
| IV: same as III but stroke only | 9 | 8752 | 5682 | 3070 | 1.06 [0.93, 1.22] | 1.05 [0.93, 1.18] | 8 | 8667 | 5639 | 3028 | 1.03 [0.91, 1.17] | 1.03 [0.91, 1.16] |
| **Disability (modified Rankin Scale)** | | | | | Odds ratio [95% CI] | | | | | | Odds ratio [95% CI] | |
| I: excellent outcome (mRS 0-1), as shown by Chu et al. | 5 | 5523 | 2771 | 2752 | 0.98 [0.73, 1.32] | 1.01 [0.91, 1.13] | 4 | 5439 | 2728 | 2711 | 0.93 [0.65, 1.31] | 1.01 [0.90, 1.12] |
| II: same as I but numbers as reported in original publications | 5 | 8084 | 5332 | 2752 | 0.98 [0.73, 1.32] | 1.01 [0.92, 1.11] | 4 | 8000 | 5289 | 2711 | 0.93 [0.66, 1.31] | 1.01 [0.92, 1.11] |
| III: good outcome or better (mRS 0-2), as shown by Chu et al. | 5 | 5523 | 2771 | 2752 | 1.04 [0.93, 1.15] | 1.04 [0.93, 1.15] | 4 | 5439 | 2728 | 2711 | 1.03 [0.92, 1.14] | 1.03 [0.92, 1.14] |
| IV: same as III but numbers as reported in original publications | 5 | 8084 | 5332 | 2752 | 1.04 [0.95, 1.14] | 1.04 [0.95, 1.14] | 4 | 8000 | 5289 | 2711 | 1.03 [0.94, 1.14] | 1.03 [0.94, 1.13] |
| V: moderate disability or better (mRS 0-3), as shown by Chu et al. | 5 | 5523 | 2771 | 2752 | 1.07 [0.66, 1.74] | 0.98 [0.88, 1.10] | 4 | 5439 | 2728 | 2711 | 0.94 [0.67, 1.32] | 0.96 [0.86, 1.08] |
| VI: same as V but numbers as reported in original publications | 5 | 8084 | 5332 | 2752 | 1.09 [0.68, 1.75] | 1.01 [0.92, 1.12] | 4 | 8000 | 5289 | 2711 | 0.96 [0.69, 1.35] | 1.00 [0.90, 1.10] |
| VII: unable to walk unassisted (mRS 0-4), as shown by Chu et al. | 5 | 5523 | 2771 | 2752 | 1.00 [0.87, 1.16] | 1.00 [0.87, 1.16] | 4 | 5439 | 2728 | 2711 | 0.98 [0.85, 1.14] | 0.98 [0.85, 1.14] |
| VIII: same as VII but numbers as reported in original publications | 5 | 8084 | 5332 | 2752 | 1.00 [0.88, 1.14] | 1.00 [0.89, 1.14] | 4 | 8000 | 5289 | 2711 | 0.99 [0.87, 1.12] | 0.99 [0.87, 1.12] |
| **Tertiary outcomes** | | | | | Risk ratio [95% CI] | | | | | | Risk ratio [95% CI] | |
| I: hospital-acquired infection, as shown by Chu et al. | 4 | 6334 | 3171 | 3163 | 1.02 [0.90, 1.15] | 1.02 [0.90, 1.15] | 3 | 6249 | 3128 | 3121 | 1.01 [0.89, 1.14] | 1.01 [0.89, 1.14] |
| II: same as I but numbers as reported in original publications | 4 | 8731 | 5676 | 3055 | 1.00 [0.90, 1.12] | 1.00 [0.90, 1.11] | 3 | 8646 | 5633 | 3013 | 1.00 [0.89, 1.11] | 1.00 [0.89, 1.11] |
| III: hospital-acquired pneumonia, as shown by Chu et al. | 1 | 624 | 312 | 312 | 2.00 [0.18, 21.94] | 2.00 [0.18, 21.94] | 1 | 624 | 312 | 312 | 2.00 [0.18, 21.94] | 2.00 [0.18, 21.94] |
| IV: same as III but numbers as reported in original publications | 1 | NR | NR | NR | Not estimable | Not estimable | 0 | NR | NR | NR | Not estimable | Not estimable |
| V: respiratory serious adverse events | 1 | 85 | 43 | 42 | 1.47 [0.26, 8.33] | 1.47 [0.26, 8.33] | 0 | 0 | 0 | 0 | Not estimable | Not estimable |
| | | | | | Mean difference [95% CI] | | | | | | Mean difference [95% CI] | |
| VI: length of hospital stay, as shown by Chu et al. | 5 | 1250 | 642 | 608 | 0.10 [-0.32, 0.51] | 0.03 [-0.18, 0.24] | 4 | 1165 | 599 | 566 | 0.06 [-0.39, 0.52] | 0.01 [-0.20, 0.23] |
| VII: same as VI but numbers as reported in original publications | 5 | 1293 | 667 | 626 | 0.40 [0.19, 0.61] | 0.40 [0.19, 0.61] | 4 | 1208 | 624 | 584 | 0.39 [0.18, 0.60] | 0.39 [0.18, 0.60] |
| VIII: length of hospital stay, all RCT | 6 | 7922 | 3978 | 3944 | 0.33 [0.12, 0.54] | 0.34 [0.14, 0.53] | 5 | 7837 | 3935 | 3902 | 0.27 [-0.02, 0.57] | 0.33 [0.13, 0.53] |

| FiO2 > 0.5 | All available RCT considered | | | | | | Without NCT00414726 | | | | | |
|---|---|---|---|---|---|---|---|---|---|---|---|---|
| | Studies, n | N | Liberal OT, n | Conservative OT, n | Risk ratio [95% CI] Random | Risk ratio [95% CI] Fixed | Studies, n | N | Liberal OT, n | Conservative OT, n | Risk ratio [95% CI] Random | Risk ratio [95% CI] Fixed |
| **Mortality in-hospital** | | | | | | | | | | | | |
| I: as shown by Chu et al. | 7 | 948 | 481 | 467 | 1.05 [0.55, 2.02] | 1.09 [0.69, 1.74] | 6 | 863 | 438 | 425 | 0.79 [0.46, 1.37] | 0.70 [0.40, 1.23] |
| II: same as I but numbers as reported in original publications, BC/WC | 7 | 952 | 483 | 469 | 0.97 [0.49, 1.89] / 1.14 [0.59, 2.18] | 1.02 [0.65, 1.61] / 1.17 [0.74, 1.84] | 6 | 867 | 440 | 427 | 0.72 [0.42, 1.23] / 0.89 [0.52, 1.51] | 0.65 [0.38, 1.12] / 0.79 [0.46, 1.35] |
| III: all RCT, BC/WC | 8 | 992 | 503 | 489 | 0.90 [0.47, 1.74] / 1.06 [0.55, 2.01] | 0.96 [0.61, 1.50] / 1.09 [0.70, 1.70] | 7 | 907 | 460 | 447 | 0.69 [0.41, 1.17] / 0.85 [0.50, 1.43] | 0.61 [0.36, 1.04] / 0.73 [0.43, 1.24] |
| IV: same as III but stroke only | 4 | 159 | 81 | 78 | 0.85 [0.10, 7.03] | 1.80 [0.81, 3.99] | 3 | 74 | 38 | 36 | 0.23 [0.03, 1.94] | 0.23 [0.03, 1.93] |
| **Mortality at 30 days** | | | | | | | | | | | | |
| I: as shown by Chu et al. | 3 | 725 | 364 | 361 | 1.46 [0.50, 4.27] | 1.39 [0.79, 2.44] | 2 | 640 | 321 | 319 | 0.85 [0.41, 1.76] | 0.85 [0.41, 1.76] |
| II: same as I but numbers as reported in original publications | 3 | 542 | 270 | 272 | 1.28 [0.35, 4.72] | 1.26 [0.70, 2.28] | 2 | 457 | 227 | 230 | 0.64 [0.28, 1.45] | 0.64 [0.28, 1.45] |
| III: all RCT | 5 | 742 | 375 | 367 | 1.03 [0.41, 2.60] | 1.10 [0.65, 1.88] | 4 | 657 | 332 | 325 | 0.64 [0.32, 1.30] | 0.62 [0.31, 1.25] |
| IV: same as III but stroke only | 3 | 141 | 72 | 69 | 1.27 [0.24, 6.80] | 1.98 [0.88, 4.46] | 2 | 56 | 29 | 27 | 0.43 [0.06, 3.06] | 0.38 [0.06, 2.49] |
| **Mortality at 90 days** | | | | | | | | | | | | |
| III: all RCT | 5 | 742 | 375 | 367 | 1.08 [0.50, 2.36] | 1.12 [0.69, 1.82] | 4 | 657 | 332 | 325 | 0.69 [0.35, 1.38] | 0.67 [0.34, 1.32] |
| IV: same as III but stroke only | 3 | 141 | 72 | 69 | 1.63 [0.56, 4.81] | 1.78 [0.91, 3.47] | 2 | 56 | 29 | 27 | 0.71 [0.10, 5.23] | 0.62 [0.13, 3.03] |
| **Mortality at longest follow-up** | | | | | | | | | | | | |
| I: as shown by Chu et al. | 8 | 988 | 501 | 487 | 1.11 [0.74, 1.67] | 1.08 [0.74, 1.58] | 7 | 903 | 458 | 445 | 0.86 [0.55, 1.35] | 0.81 [0.51, 1.29] |
| II: same as I but numbers as reported in original publications, BC/WC | 8 | 809 | 409 | 400 | 1.04 [0.63, 1.71] / 1.20 [0.76, 1.91] | 1.05 [0.70, 1.57] / 1.16 [0.78, 1.74] | 7 | 724 | 366 | 358 | 0.79 [0.48, 1.28] / 0.94 [0.57, 1.53] | 0.73 [0.44, 1.20] / 0.86 [0.52, 1.40] |
| III: same as II but stroke only | 4 | 159 | 81 | 78 | 1.63 [0.56, 4.81] | 1.78 [0.91, 3.47] | 3 | 74 | 38 | 36 | 0.71 [0.10, 5.23] | 0.62 [0.13, 3.03] |
| **Disability (modified Rankin Scale)** | | | | | Odds ratio [95% CI] | | | | | | Odds ratio [95% CI] | |
| I: excellent outcome (mRS 0-1), as shown by Chu et al. | 2 | 100 | 52 | 48 | 0.80 [0.12, 5.36] | 1.12 [0.45, 2.80] | 1 | 16 | 9 | 7 | 0.21 [0.02, 2.52] | 0.21 [0.02, 2.52] |
| II: good outcome or better (mRS 0-2), as shown by Chu et al. | 2 | 100 | 52 | 48 | 0.85 [0.11, 6.34] | 1.33 [0.60, 2.97] | 1 | 16 | 9 | 7 | 0.21 [0.02, 2.52] | 0.21 [0.02, 2.52] |
| III: moderate disability or better (mRS 0-3), as shown by Chu et al. | 2 | 100 | 52 | 48 | 1.03 [0.08, 13.85] | 2.04 [0.92, 4.55] | 1 | 16 | 9 | 7 | 0.21 [0.02, 2.52] | 0.21 [0.02, 2.52] |
| IV: unable to walk unassisted (mRS 0-4), as shown by Chu et al. | 2 | 100 | 52 | 48 | 1.75 [0.62, 4.89] | 1.85 [0.79, 4.35] | 1 | 16 | 9 | 7 | 0.67 [0.09, 5.13] | 0.67 [0.09, 5.13] |
| **Tertiary outcomes** | | | | | Risk ratio [95% CI] | | | | | | Risk ratio [95% CI] | |
| I: hospital-acquired infection, as shown by Chu et al. | 2 | 709 | 355 | 354 | 1.33 [0.80, 2.20] | 1.36 [0.82, 2.28] | 1 | 624 | 312 | 312 | 1.75 [0.52, 5.92] | 1.75 [0.52, 5.92] |
| II: same as I but numbers as reported in original publications | 2 | 526 | 261 | 265 | 1.21 [0.70, 2.09] | 1.17 [0.68, 2.02] | 1 | 441 | 218 | 223 | 0.34 [0.01, 8.32] | 0.34 [0.01, 8.32] |
| III: hospital-acquired pneumonia, as shown by Chu et al. | 1 | 624 | 312 | 312 | 2.00 [0.18, 21.94] | 2.00 [0.18, 21.94] | 1 | 624 | 312 | 312 | 2.00 [0.18, 21.94] | 2.00 [0.18, 21.94] |
| IV: same as III but numbers as reported in original publications | 1 | NR | NR | NR | Not estimable | Not estimable | 1 | NR | NR | NR | Not estimable | Not estimable |
| V: respiratory serious adverse events | 1 | 85 | 43 | 42 | 1.47 [0.26, 8.33] | 1.47 [0.26, 8.33] | 0 | 0 | 0 | 0 | Not estimable | Not estimable |
| | | | | | Mean difference [95% CI] | | | | | | Mean difference [95% CI] | |
| VI: length of hospital stay, as shown by Chu et al. | 3 | 543 | 270 | 273 | 0.07 [-0.52, 0.66] | 0.01 [-0.21, 0.23] | 2 | 458 | 227 | 231 | 0.41 [-3.34, 2.52] | 0.00 [-0.22, 0.22] |
| VII: same as VI but numbers as reported in original publications | 3 | 543 | 270 | 273 | 0.41 [0.19, 0.62] | 0.41 [0.19, 0.62] | 2 | 458 | 227 | 231 | 0.40 [0.18, 0.62] | 0.40 [0.18, 0.62] |

BC, best case; CI, confidence interval; NR, not reported; OT, oxygen therapy; RCT, randomized controlled trial; SAE, serious adverse events; WC, worst case

# Mortality in-hospital, any oxygen therapy

## I: as shown by Chu et al. Lancet. 2018 Apr 28;391(10131):1693-1705; N = 14,101

| Study or Subgroup | Liberal Events | Liberal Total | Conservative Events | Conservative Total | Weight | Risk Ratio M-H, Random, 95% CI |
|---|---|---|---|---|---|---|
| Ali et al (2014) | 5 | 155 | 4 | 146 | 2.6% | 1.18 [0.32, 4.30] |
| Hofmann et al (2017), DETO2X-AMI | 53 | 3311 | 44 | 3318 | 27.9% | 1.21 [0.81, 1.80] |
| Khoshnood et al (2016), SOCCER | 3 | 85 | 3 | 75 | 1.8% | 0.88 [0.18, 4.24] |
| Kuisma et al (2006) | 4 | 14 | 4 | 14 | 3.2% | 1.00 [0.31, 3.23] |
| NCT00414726 | 14 | 43 | 4 | 42 | 4.2% | 3.42 [1.22, 9.54] |
| Rawles et al (1976) | 9 | 105 | 3 | 95 | 2.7% | 2.71 [0.76, 9.73] |
| Roffe et al (2017), SO2S | 50 | 2668 | 45 | 2668 | 27.5% | 1.11 [0.75, 1.66] |
| Rønning et al (1999) | 36 | 292 | 27 | 258 | 19.9% | 1.18 [0.74, 1.89] |
| Shi et al (2017) | 0 | 9 | 0 | 9 | | Not estimable |
| Singhal et al (2005) | 0 | 9 | 1 | 7 | 0.5% | 0.27 [0.01, 5.70] |
| Stub et al (2015) | 5 | 312 | 11 | 312 | 4.0% | 0.45 [0.16, 1.29] |
| Ukholkina et al (2005) | 1 | 58 | 0 | 79 | 0.4% | 4.07 [0.17, 98.10] |
| Young et al (2014), HOT OR NOT | 5 | 9 | 4 | 8 | 5.3% | 1.11 [0.45, 2.75] |
| **Total (95% CI)** | | **7070** | | **7031** | **100.0%** | **1.18 [0.96, 1.46]** |
| Total events | 185 | | 150 | | | |

Heterogeneity: Tau² = 0.00; Chi² = 10.77, df = 11 (P = 0.46); I² = 0%
Test for overall effect: Z = 1.57 (P = 0.12)

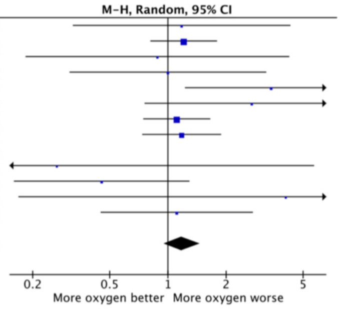


## II: same as I but numbers as reported in original publications; N = 16,718

| Study or Subgroup | Liberal Events | Liberal Total | Conservative Events | Conservative Total | Weight | Risk Ratio M-H, Random, 95% CI |
|---|---|---|---|---|---|---|
| Ali et al (2014) | 5 | 148 | 4 | 141 | 3.3% | 1.19 [0.33, 4.35] |
| Hofmann et al (2017), DETO2X-AMI | 53 | 3311 | 44 | 3318 | 23.5% | 1.21 [0.81, 1.80] |
| Khoshnood et al (2016), SOCCER | 3 | 85 | 3 | 75 | 2.3% | 0.88 [0.18, 4.24] |
| Kuisma et al (2006) | 4 | 16 | 6 | 16 | 4.8% | 0.67 [0.23, 1.92] |
| Kuisma et al (2006) | 6 | 16 | 4 | 16 | 0.0% | 1.50 [0.52, 4.32] |
| NCT00414726 | 14 | 43 | 4 | 42 | 5.1% | 3.42 [1.22, 9.54] |
| Rawles et al (1976) | 9 | 105 | 3 | 95 | 3.4% | 2.71 [0.76, 9.73] |
| Roffe et al (2017), SO2S | 85 | 5297 | 45 | 2664 | 26.6% | 0.95 [0.66, 1.36] |
| Rønning et al (1999) | 36 | 292 | 27 | 258 | 18.7% | 1.18 [0.74, 1.89] |
| Shi et al (2017) | 0 | 9 | 0 | 9 | | Not estimable |
| Singhal et al (2005) | 0 | 9 | 1 | 7 | 0.6% | 0.27 [0.01, 5.70] |
| Stub et al (2015) | 5 | 312 | 11 | 312 | 4.9% | 0.45 [0.16, 1.29] |
| Ukholkina et al (2005) | 1 | 58 | 0 | 79 | 0.6% | 4.07 [0.17, 98.10] |
| Young et al (2014), HOT OR NOT | 5 | 9 | 4 | 8 | 6.4% | 1.11 [0.45, 2.75] |
| **Total (95% CI)** | | **9694** | | **7024** | **100.0%** | **1.11 [0.88, 1.42]** |
| Total events | 220 | | 152 | | | |

Heterogeneity: Tau² = 0.02; Chi² = 12.72, df = 11 (P = 0.31); I² = 14%
Test for overall effect: Z = 0.88 (P = 0.38)

| | | | | | | |
|---|---|---|---|---|---|---|
| **Total (95% CI)** | | **9694** | | **7024** | **100.0%** | **1.15 [0.92, 1.45]** |
| Total events | 222 | | 150 | | | |

Heterogeneity: Tau² = 0.01; Chi² = 12.08, df = 11 (P = 0.36); I² = 9%
Test for overall effect: Z = 1.22 (P = 0.22)

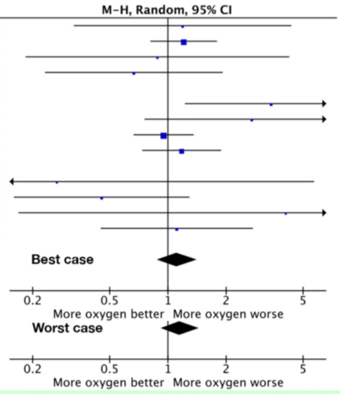


## III: all RCT; N = 17,066

| Study or Subgroup | Liberal Events | Liberal Total | Conservative Events | Conservative Total | Weight | Risk Ratio M-H, Random, 95% CI |
|---|---|---|---|---|---|---|
| Ali et al (2014) | 5 | 148 | 4 | 141 | 2.6% | 1.19 [0.33, 4.35] |
| Chiu et al (2006) | 1 | 17 | 7 | 29 | 0.0% | 0.24 [0.03, 1.82] |
| Hofmann et al (2017), DETO2X-AMI | 53 | 3311 | 44 | 3318 | 23.4% | 1.21 [0.81, 1.80] |
| Khoshnood et al (2016), SOCCER | 3 | 85 | 3 | 75 | 1.8% | 0.88 [0.18, 4.24] |
| Kuisma et al (2006) | 4 | 16 | 6 | 16 | 3.9% | 0.67 [0.23, 1.92] |
| Kuisma et al (2006) | 6 | 16 | 4 | 16 | 0.0% | 1.50 [0.52, 4.32] |
| Mazdeh et al (2015) | 5 | 26 | 3 | 25 | 2.5% | 1.60 [0.43, 6.01] |
| NCT00414726 | 14 | 43 | 4 | 42 | 4.2% | 3.42 [1.22, 9.54] |
| Padma et al (2010) | 0 | 20 | 2 | 20 | 0.5% | 0.20 [0.01, 3.92] |
| Ranchord et al (2012), OPTIMISE | 1 | 72 | 4 | 76 | 1.0% | 0.26 [0.03, 2.31] |
| Ranchord et al (2012), OPTIMISE | 3 | 72 | 2 | 76 | 0.0% | 1.58 [0.27, 9.20] |
| Rawles et al (1976) | 9 | 105 | 3 | 95 | 2.7% | 2.71 [0.76, 9.73] |
| Roffe et al (2010) | 2 | 29 | 3 | 30 | 1.5% | 0.69 [0.12, 3.83] |
| Roffe et al (2017), SO2S | 85 | 5297 | 45 | 2664 | 27.5% | 0.95 [0.66, 1.36] |
| Rønning et al (1999) | 36 | 292 | 27 | 258 | 17.6% | 1.18 [0.74, 1.89] |
| Shi et al (2017) | 0 | 9 | 0 | 9 | | Not estimable |
| Singhal et al (2005) | 0 | 9 | 1 | 7 | 0.5% | 0.27 [0.01, 5.70] |
| Stub et al (2015) | 5 | 312 | 11 | 312 | 4.0% | 0.45 [0.16, 1.29] |
| Ukholkina et al (2005) | 1 | 58 | 0 | 79 | 0.4% | 4.07 [0.17, 98.10] |
| Wilson et al (1997) | 0 | 25 | 1 | 25 | 0.5% | 0.33 [0.01, 7.81] |
| Wilson et al (1997) | 1 | 25 | 0 | 25 | 0.0% | 3.00 [0.13, 70.30] |
| Young et al (2014), HOT OR NOT | 5 | 9 | 4 | 8 | 5.3% | 1.11 [0.45, 2.75] |
| **Total (95% CI)** | | **9866** | | **7200** | **100.0%** | **1.08 [0.87, 1.34]** |
| Total events | 228 | | 165 | | | |

Heterogeneity: Tau² = 0.01; Chi² = 16.75, df = 16 (P = 0.40); I² = 4%
Test for overall effect: Z = 0.72 (P = 0.47)

| | | | | | | |
|---|---|---|---|---|---|---|
| **Total (95% CI)** | | **9866** | | **7200** | **100.0%** | **1.14 [0.94, 1.39]** |
| Total events | 233 | | 160 | | | |

Heterogeneity: Tau² = 0.00; Chi² = 14.47, df = 16 (P = 0.56); I² = 0%
Test for overall effect: Z = 1.29 (P = 0.20)

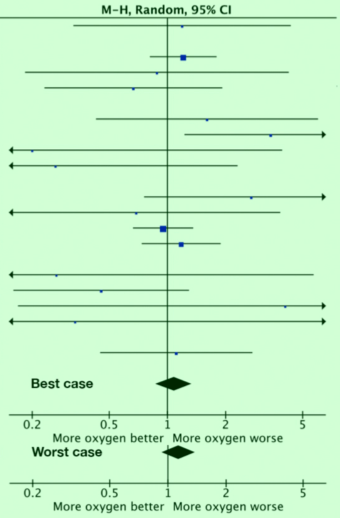


## IV: same as III but stroke only; N = 9,069

| Study or Subgroup | Liberal Events | Liberal Total | Conservative Events | Conservative Total | Weight | Risk Ratio M-H, Random, 95% CI |
|---|---|---|---|---|---|---|
| Ali et al (2014) | 5 | 148 | 4 | 141 | 5.9% | 1.19 [0.33, 4.35] |
| Chiu et al (2006) | 1 | 17 | 7 | 29 | 0.0% | 0.24 [0.03, 1.82] |
| Mazdeh et al (2015) | 5 | 26 | 3 | 25 | 5.6% | 1.60 [0.43, 6.01] |
| NCT00414726 | 14 | 43 | 4 | 42 | 9.0% | 3.42 [1.22, 9.54] |
| Padma et al (2010) | 0 | 20 | 2 | 20 | 1.2% | 0.20 [0.01, 3.92] |
| Roffe et al (2010) | 2 | 29 | 3 | 30 | 3.4% | 0.69 [0.12, 3.83] |
| Roffe et al (2017), SO2S | 85 | 5297 | 45 | 2664 | 42.8% | 0.95 [0.66, 1.36] |
| Rønning et al (1999) | 36 | 292 | 27 | 258 | 31.1% | 1.18 [0.74, 1.89] |
| Shi et al (2017) | 0 | 9 | 0 | 9 | | Not estimable |
| Singhal et al (2005) | 0 | 9 | 1 | 7 | 1.1% | 0.27 [0.01, 5.70] |
| **Total (95% CI)** | | **5873** | | **3196** | **100.0%** | **1.14 [0.82, 1.57]** |
| Total events | 147 | | 89 | | | |

Heterogeneity: Tau² = 0.03; Chi² = 8.11, df = 7 (P = 0.32); I² = 14%
Test for overall effect: Z = 0.78 (P = 0.43)

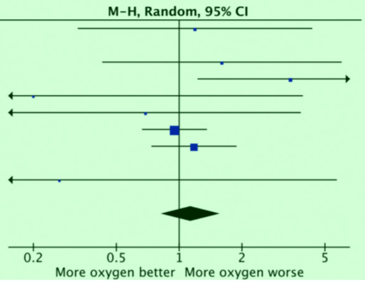

# Mortality in-hospital, any OT, w/o NCT00414726

## I: as shown by Chu et al. Lancet. 2018 Apr 28;391(10131):1693-1705; N = 14,016

| Study or Subgroup | Liberal Events | Liberal Total | Conservative Events | Conservative Total | Weight | Risk Ratio M-H, Random, 95% CI |
|---|---|---|---|---|---|---|
| Ali et al (2014) | 5 | 155 | 4 | 146 | 2.7% | 1.18 [0.32, 4.30] |
| Hofmann et al (2017), DETO2X-AMI | 53 | 3311 | 44 | 3318 | 29.1% | 1.21 [0.81, 1.80] |
| Khoshnood et al (2016), SOCCER | 3 | 85 | 3 | 75 | 1.9% | 0.88 [0.18, 4.24] |
| Kuisma et al (2006) | 4 | 14 | 4 | 14 | 3.3% | 1.00 [0.31, 3.23] |
| NCT00414726 | 14 | 43 | 4 | 42 | 0.0% | 3.42 [1.22, 9.54] |
| Rawles et al (1976) | 9 | 105 | 3 | 95 | 2.8% | 2.71 [0.76, 9.73] |
| Roffe et al (2017), SO2S | 50 | 2668 | 45 | 2668 | 28.7% | 1.11 [0.75, 1.66] |
| Rønning et al (1999) | 36 | 292 | 27 | 258 | 20.7% | 1.18 [0.74, 1.89] |
| Shi et al (2017) | 0 | 9 | 0 | 9 | | Not estimable |
| Singhal et al (2005) | 0 | 9 | 1 | 7 | 0.5% | 0.27 [0.01, 5.70] |
| Stub et al (2015) | 5 | 312 | 11 | 312 | 4.2% | 0.45 [0.16, 1.29] |
| Ukholkina et al (2005) | 1 | 58 | 0 | 79 | 0.5% | 4.07 [0.17, 98.10] |
| Young et al (2014), HOT OR NOT | 5 | 9 | 4 | 8 | 5.6% | 1.11 [0.45, 2.75] |
| **Total (95% CI)** | | **7027** | | **6989** | **100.0%** | **1.13 [0.91, 1.40]** |
| Total events | 171 | | 146 | | | |

Heterogeneity: $Tau^2 = 0.00; Chi^2 = 6.49, df = 10 (P = 0.77); I^2 = 0\%$
Test for overall effect: Z = 1.12 (P = 0.26)

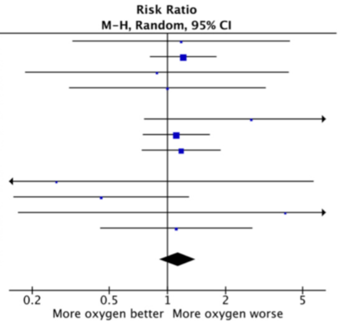


## II: same as I but numbers as reported in original publications; N = 16,633

| Study or Subgroup | Liberal Events | Liberal Total | Conservative Events | Conservative Total | Weight | Risk Ratio M-H, Random, 95% CI |
|---|---|---|---|---|---|---|
| Ali et al (2014) | 5 | 148 | 4 | 141 | 2.5% | 1.19 [0.33, 4.35] |
| Hofmann et al (2017), DETO2X-AMI | 53 | 3311 | 44 | 3318 | 27.0% | 1.21 [0.81, 1.80] |
| Khoshnood et al (2016), SOCCER | 3 | 85 | 3 | 75 | 1.7% | 0.88 [0.18, 4.24] |
| Kuisma et al (2006) | 6 | 16 | 4 | 16 | 0.0% | 1.50 [0.52, 4.32] |
| Kuisma et al (2006) | 4 | 16 | 6 | 16 | 3.8% | 0.67 [0.23, 1.92] |
| NCT00414726 | 14 | 43 | 4 | 42 | 0.0% | 3.42 [1.22, 9.54] |
| Rawles et al (1976) | 9 | 105 | 3 | 95 | 2.6% | 2.71 [0.76, 9.73] |
| Roffe et al (2017), SO2S | 85 | 5297 | 45 | 2664 | 33.1% | 0.95 [0.66, 1.36] |
| Rønning et al (1999) | 36 | 292 | 27 | 258 | 19.3% | 1.18 [0.74, 1.89] |
| Shi et al (2017) | 0 | 9 | 0 | 9 | | Not estimable |
| Singhal et al (2005) | 0 | 9 | 1 | 7 | 0.5% | 0.27 [0.01, 5.70] |
| Stub et al (2015) | 5 | 312 | 11 | 312 | 3.9% | 0.45 [0.16, 1.29] |
| Ukholkina et al (2005) | 1 | 58 | 0 | 79 | 0.4% | 4.07 [0.17, 98.10] |
| Young et al (2014), HOT OR NOT | 5 | 9 | 4 | 8 | 5.2% | 1.11 [0.45, 2.75] |
| **Total (95% CI)** | | **9651** | | **6982** | **100.0%** | **1.05 [0.86, 1.30]** |
| Total events | 206 | | 148 | | | |
| **Total (95% CI)** | | **9651** | | **6982** | **100.0%** | **1.09 [0.88, 1.34]** |
| Total events | 208 | | 146 | | | |

Best case: Heterogeneity: $Tau^2 = 0.00; Chi^2 = 7.86, df = 10 (P = 0.64); I^2 = 0\%$
Test for overall effect: Z = 0.50 (P = 0.61)

Worst case: Heterogeneity: $Tau^2 = 0.00; Chi^2 = 7.48, df = 10 (P = 0.68); I^2 = 0\%$
Test for overall effect: Z = 0.80 (P = 0.43)

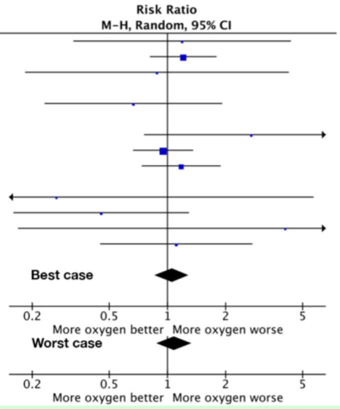


## III: all RCT; N = 16,981

| Study or Subgroup | Liberal Events | Liberal Total | Conservative Events | Conservative Total | Weight | Risk Ratio M-H, Random, 95% CI |
|---|---|---|---|---|---|---|
| Ali et al (2014) | 5 | 148 | 4 | 141 | 2.4% | 1.19 [0.33, 4.35] |
| Chiu et al (2006) | 1 | 17 | 7 | 29 | 0.0% | 0.24 [0.03, 1.82] |
| Hofmann et al (2017), DETO2X-AMI | 53 | 3311 | 44 | 3318 | 25.6% | 1.21 [0.81, 1.80] |
| Khoshnood et al (2016), SOCCER | 3 | 85 | 3 | 75 | 1.6% | 0.88 [0.18, 4.24] |
| Kuisma et al (2006) | 6 | 16 | 4 | 16 | 0.0% | 1.50 [0.52, 4.32] |
| Kuisma et al (2006) | 4 | 16 | 6 | 16 | 3.6% | 0.67 [0.23, 1.92] |
| Mazdeh et al (2015) | 5 | 26 | 3 | 25 | 2.3% | 1.60 [0.43, 6.01] |
| NCT00414726 | 14 | 43 | 4 | 42 | 0.0% | 3.42 [1.22, 9.54] |
| Padma et al (2010) | 0 | 20 | 2 | 20 | 0.5% | 0.20 [0.01, 3.92] |
| Ranchord et al (2012), OPTIMISE | 1 | 72 | 4 | 76 | 0.9% | 0.26 [0.03, 2.31] |
| Ranchord et al (2012), OPTIMISE | 3 | 72 | 2 | 76 | 0.0% | 1.58 [0.27, 9.20] |
| Rawles et al (1976) | 9 | 105 | 3 | 95 | 2.5% | 2.71 [0.76, 9.73] |
| Roffe et al (2010) | 2 | 29 | 3 | 30 | 1.4% | 0.69 [0.12, 3.83] |
| Roffe et al (2017), SO2S | 85 | 5297 | 45 | 2664 | 31.3% | 0.95 [0.66, 1.36] |
| Rønning et al (1999) | 36 | 292 | 27 | 258 | 18.2% | 1.18 [0.74, 1.89] |
| Shi et al (2017) | 0 | 9 | 0 | 9 | | Not estimable |
| Singhal et al (2005) | 0 | 9 | 1 | 7 | 0.4% | 0.27 [0.01, 5.70] |
| Stub et al (2015) | 5 | 312 | 11 | 312 | 3.7% | 0.45 [0.16, 1.29] |
| Ukholkina et al (2005) | 1 | 58 | 0 | 79 | 0.4% | 4.07 [0.17, 98.10] |
| Wilson et al (1997) | 1 | 25 | 0 | 25 | 0.0% | 3.00 [0.13, 70.30] |
| Wilson et al (1997) | 0 | 25 | 1 | 25 | 0.4% | 0.33 [0.01, 7.81] |
| Young et al (2014), HOT OR NOT | 5 | 9 | 4 | 8 | 4.9% | 1.11 [0.45, 2.75] |
| **Total (95% CI)** | | **9823** | | **7158** | **100.0%** | **1.03 [0.85, 1.26]** |
| Total events | 214 | | 161 | | | |
| **Total (95% CI)** | | **9823** | | **7158** | **100.0%** | **1.09 [0.89, 1.33]** |
| Total events | 219 | | 156 | | | |

Best case: Heterogeneity: $Tau^2 = 0.00; Chi^2 = 11.73, df = 15 (P = 0.70); I^2 = 0\%$
Test for overall effect: Z = 0.32 (P = 0.75)

Worst case: Heterogeneity: $Tau^2 = 0.00; Chi^2 = 9.90, df = 15 (P = 0.83); I^2 = 0\%$
Test for overall effect: Z = 0.86 (P = 0.39)

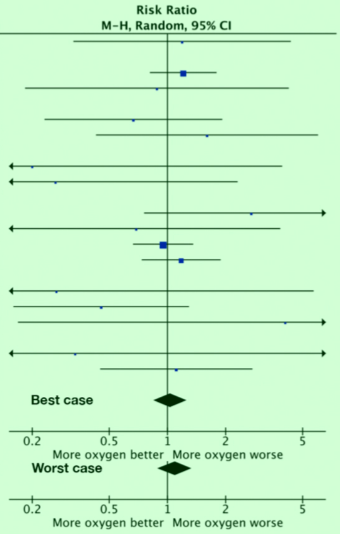


## IV: same as III but stroke only; N = 8,984

| Study or Subgroup | Liberal Events | Liberal Total | Conservative Events | Conservative Total | Weight | Risk Ratio M-H, Random, 95% CI |
|---|---|---|---|---|---|---|
| Ali et al (2014) | 5 | 148 | 4 | 141 | 4.3% | 1.19 [0.33, 4.35] |
| Chiu et al (2006) | 1 | 17 | 7 | 29 | 0.0% | 0.24 [0.03, 1.82] |
| Mazdeh et al (2015) | 5 | 26 | 3 | 25 | 4.1% | 1.60 [0.43, 6.01] |
| NCT00414726 | 14 | 43 | 4 | 42 | 0.0% | 3.42 [1.22, 9.54] |
| Padma et al (2010) | 0 | 20 | 2 | 20 | 0.8% | 0.20 [0.01, 3.92] |
| Roffe et al (2010) | 2 | 29 | 3 | 30 | 2.4% | 0.69 [0.12, 3.83] |
| Roffe et al (2017), SO2S | 85 | 5297 | 45 | 2664 | 55.5% | 0.95 [0.66, 1.36] |
| Rønning et al (1999) | 36 | 292 | 27 | 258 | 32.2% | 1.18 [0.74, 1.89] |
| Shi et al (2017) | 0 | 9 | 0 | 9 | | Not estimable |
| Singhal et al (2005) | 0 | 9 | 1 | 7 | 0.8% | 0.27 [0.01, 5.70] |
| **Total (95% CI)** | | **5830** | | **3154** | **100.0%** | **1.02 [0.78, 1.33]** |
| Total events | 133 | | 85 | | | |

Heterogeneity: $Tau^2 = 0.00; Chi^2 = 3.11, df = 6 (P = 0.79); I^2 = 0\%$
Test for overall effect: Z = 0.14 (P = 0.89)

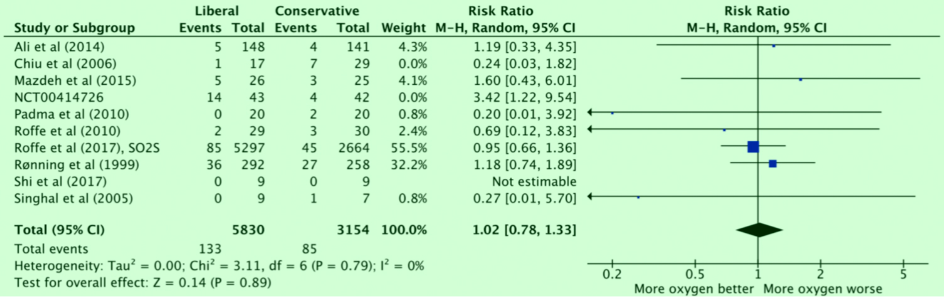

# Mortality in-hospital, FiO2 > 0.5

### I: as shown by Chu et al. Lancet. 2018 Apr 28;391(10131):1693-1705; N = 948

| Study or Subgroup | Liberal Events | Liberal Total | Conservative Events | Conservative Total | Weight | Risk Ratio M-H, Random, 95% CI |
|---|---|---|---|---|---|---|
| Khoshnood et al (2016), SOCCER | 3 | 85 | 3 | 75 | 12.4% | 0.88 [0.18, 4.24] |
| Kuisma et al (2006) | 4 | 14 | 4 | 14 | 18.2% | 1.00 [0.31, 3.23] |
| NCT00414726 | 14 | 43 | 4 | 42 | 21.0% | 3.42 [1.22, 9.54] |
| Shi et al (2017) | 0 | 9 | 0 | 9 | | Not estimable |
| Singhal et al (2005) | 0 | 9 | 1 | 7 | 4.1% | 0.27 [0.01, 5.70] |
| Stub et al (2015) | 5 | 312 | 11 | 312 | 20.6% | 0.45 [0.16, 1.29] |
| Young et al (2014), HOT OR NOT | 5 | 9 | 4 | 8 | 23.7% | 1.11 [0.45, 2.75] |
| **Total (95% CI)** | | **481** | | **467** | **100.0%** | **1.05 [0.55, 2.02]** |
| Total events | 31 | | 27 | | | |

Heterogeneity: $Tau^2 = 0.26$; $Chi^2 = 8.36$, df = 5 (P = 0.14); $I^2 = 40\%$
Test for overall effect: Z = 0.15 (P = 0.88)

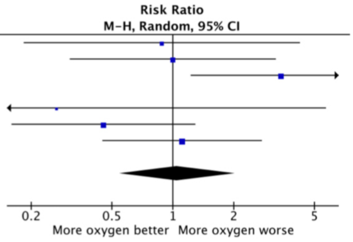


### II: same as I but numbers as reported in original publications; N = 952

| Study or Subgroup | Liberal Events | Liberal Total | Conservative Events | Conservative Total | Weight | Risk Ratio M-H, Random, 95% CI |
|---|---|---|---|---|---|---|
| Khoshnood et al (2016), SOCCER | 3 | 85 | 3 | 75 | 12.5% | 0.88 [0.18, 4.24] |
| Kuisma et al (2006) | 4 | 16 | 6 | 16 | 19.9% | 0.67 [0.23, 1.92] |
| Kuisma et al (2006) | 6 | 16 | 4 | 16 | 0.0% | 1.50 [0.52, 4.32] |
| NCT00414726 | 14 | 43 | 4 | 42 | 20.5% | 3.42 [1.22, 9.54] |
| Shi et al (2017) | 0 | 9 | 0 | 9 | | Not estimable |
| Singhal et al (2005) | 0 | 9 | 1 | 7 | 4.3% | 0.27 [0.01, 5.70] |
| Stub et al (2015) | 5 | 312 | 11 | 312 | 20.1% | 0.45 [0.16, 1.29] |
| Young et al (2014), HOT OR NOT | 5 | 9 | 4 | 8 | 22.9% | 1.11 [0.45, 2.75] |
| **Total (95% CI)** | | **483** | | **469** | **100.0%** | **0.97 [0.49, 1.89]** |
| Total events | 31 | | 29 | | | |

Heterogeneity: $Tau^2 = 0.30$; $Chi^2 = 9.05$, df = 5 (P = 0.11); $I^2 = 45\%$
Test for overall effect: Z = 0.10 (P = 0.92)

| | | | | | | |
|---|---|---|---|---|---|---|
| **Total (95% CI)** | | **483** | | **469** | **100.0%** | **1.14 [0.59, 2.18]** |
| Total events | 33 | | 27 | | | |

Heterogeneity: $Tau^2 = 0.26$; $Chi^2 = 8.58$, df = 5 (P = 0.13); $I^2 = 42\%$
Test for overall effect: Z = 0.39 (P = 0.70)

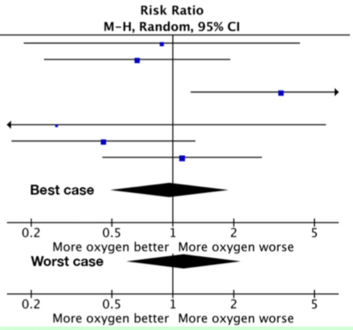


### III: all RCT; N = 992

| Study or Subgroup | Liberal Events | Liberal Total | Conservative Events | Conservative Total | Weight | Risk Ratio M-H, Random, 95% CI |
|---|---|---|---|---|---|---|
| Khoshnood et al (2016), SOCCER | 3 | 85 | 3 | 75 | 11.9% | 0.88 [0.18, 4.24] |
| Kuisma et al (2006) | 6 | 16 | 4 | 16 | 0.0% | 1.50 [0.52, 4.32] |
| Kuisma et al (2006) | 4 | 16 | 6 | 16 | 19.0% | 0.67 [0.23, 1.92] |
| NCT00414726 | 14 | 43 | 4 | 42 | 19.6% | 3.42 [1.22, 9.54] |
| Padma et al (2010) | 0 | 20 | 2 | 20 | 4.3% | 0.20 [0.01, 3.92] |
| Shi et al (2017) | 0 | 9 | 0 | 9 | | Not estimable |
| Singhal et al (2005) | 0 | 9 | 1 | 7 | 4.1% | 0.27 [0.01, 5.70] |
| Stub et al (2015) | 5 | 312 | 11 | 312 | 19.2% | 0.45 [0.16, 1.29] |
| Young et al (2014), HOT OR NOT | 5 | 9 | 4 | 8 | 21.9% | 1.11 [0.45, 2.75] |
| **Total (95% CI)** | | **503** | | **489** | **100.0%** | **0.90 [0.47, 1.74]** |
| Total events | 31 | | 31 | | | |

Heterogeneity: $Tau^2 = 0.29$; $Chi^2 = 10.15$, df = 6 (P = 0.12); $I^2 = 41\%$
Test for overall effect: Z = 0.31 (P = 0.76)

| | | | | | | |
|---|---|---|---|---|---|---|
| **Total (95% CI)** | | **503** | | **489** | **100.0%** | **1.06 [0.55, 2.01]** |
| Total events | 33 | | 29 | | | |

Heterogeneity: $Tau^2 = 0.28$; $Chi^2 = 9.94$, df = 6 (P = 0.13); $I^2 = 40\%$
Test for overall effect: Z = 0.17 (P = 0.87)

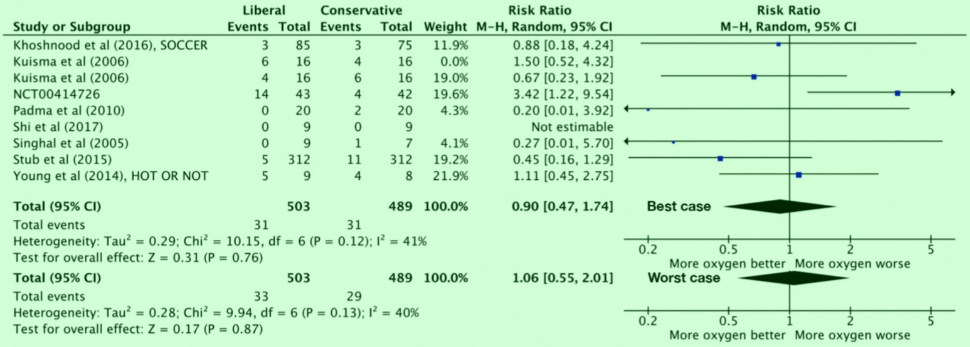


### IV: same as III but stroke only; N = 159

| Study or Subgroup | Liberal Events | Liberal Total | Conservative Events | Conservative Total | Weight | Risk Ratio M-H, Random, 95% CI |
|---|---|---|---|---|---|---|
| NCT00414726 | 14 | 43 | 4 | 42 | 48.3% | 3.42 [1.22, 9.54] |
| Padma et al (2010) | 0 | 20 | 2 | 20 | 26.2% | 0.20 [0.01, 3.92] |
| Shi et al (2017) | 0 | 9 | 0 | 9 | | Not estimable |
| Singhal et al (2005) | 0 | 9 | 1 | 7 | 25.4% | 0.27 [0.01, 5.70] |
| **Total (95% CI)** | | **81** | | **78** | **100.0%** | **0.85 [0.10, 7.03]** |
| Total events | 14 | | 7 | | | |

Heterogeneity: $Tau^2 = 2.13$; $Chi^2 = 5.09$, df = 2 (P = 0.08); $I^2 = 61\%$
Test for overall effect: Z = 0.15 (P = 0.88)

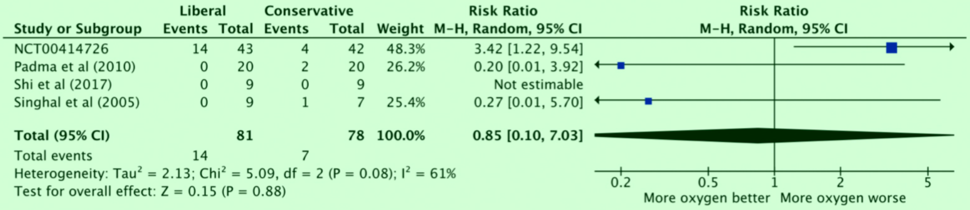

# Mortality in-hospital, FiO2 > 0.5, w/o NCT00414726

## I: as shown by Chu et al. Lancet. 2018 Apr 28;391(10131):1693-1705; N = 863

| Study or Subgroup | Liberal Events | Liberal Total | Conservative Events | Conservative Total | Weight | Risk Ratio M-H, Random, 95% CI |
|---|---|---|---|---|---|---|
| Khoshnood et al (2016), SOCCER | 3 | 85 | 3 | 75 | 12.0% | 0.88 [0.18, 4.24] |
| Kuisma et al (2006) | 4 | 14 | 4 | 14 | 21.6% | 1.00 [0.31, 3.23] |
| NCT00414726 | 14 | 43 | 4 | 42 | 0.0% | 3.42 [1.22, 9.54] |
| Shi et al (2017) | 0 | 9 | 0 | 9 | | Not estimable |
| Singhal et al (2005) | 0 | 9 | 1 | 7 | 3.2% | 0.27 [0.01, 5.70] |
| Stub et al (2015) | 5 | 312 | 11 | 312 | 27.1% | 0.45 [0.16, 1.29] |
| Young et al (2014), HOT OR NOT | 5 | 9 | 4 | 8 | 36.1% | 1.11 [0.45, 2.75] |
| **Total (95% CI)** | | **438** | | **425** | **100.0%** | **0.79 [0.46, 1.37]** |
| Total events | 17 | | 23 | | | |

Heterogeneity: Tau² = 0.00; Chi² = 2.46, df = 4 (P = 0.65); I² = 0%
Test for overall effect: Z = 0.84 (P = 0.40)

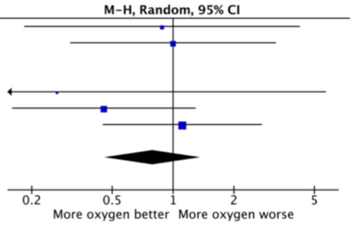


## II: same as I but numbers as reported in original publications; N = 867

| Study or Subgroup | Liberal Events | Liberal Total | Conservative Events | Conservative Total | Weight | Risk Ratio M-H, Random, 95% CI |
|---|---|---|---|---|---|---|
| Khoshnood et al (2016), SOCCER | 3 | 85 | 3 | 75 | 11.5% | 0.88 [0.18, 4.24] |
| Kuisma et al (2006) | 4 | 16 | 6 | 16 | 25.2% | 0.67 [0.23, 1.92] |
| Kuisma et al (2006) | 6 | 16 | 4 | 16 | 0.0% | 1.50 [0.52, 4.32] |
| NCT00414726 | 14 | 43 | 4 | 42 | 0.0% | 3.42 [1.22, 9.54] |
| Shi et al (2017) | 0 | 9 | 0 | 9 | | Not estimable |
| Singhal et al (2005) | 0 | 9 | 1 | 7 | 3.0% | 0.27 [0.01, 5.70] |
| Stub et al (2015) | 5 | 312 | 11 | 312 | 25.9% | 0.45 [0.16, 1.29] |
| Young et al (2014), HOT OR NOT | 5 | 9 | 4 | 8 | 34.4% | 1.11 [0.45, 2.75] |
| **Total (95% CI)** | | **440** | | **427** | **100.0%** | **0.72 [0.42, 1.23]** |
| Total events | 17 | | 25 | | | |

Heterogeneity: Tau² = 0.00; Chi² = 2.27, df = 4 (P = 0.69); I² = 0%
Test for overall effect: Z = 1.20 (P = 0.23)

| | | | | | | |
|---|---|---|---|---|---|---|
| **Total (95% CI)** | | **440** | | **427** | **100.0%** | **0.89 [0.52, 1.51]** |
| Total events | 19 | | 23 | | | |

Heterogeneity: Tau² = 0.00; Chi² = 3.54, df = 4 (P = 0.47); I² = 0%
Test for overall effect: Z = 0.44 (P = 0.66)

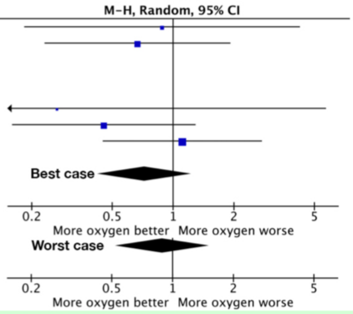


## III: all RCT; N = 907

| Study or Subgroup | Liberal Events | Liberal Total | Conservative Events | Conservative Total | Weight | Risk Ratio M-H, Random, 95% CI |
|---|---|---|---|---|---|---|
| Khoshnood et al (2016), SOCCER | 3 | 85 | 3 | 75 | 11.1% | 0.88 [0.18, 4.24] |
| Kuisma et al (2006) | 6 | 16 | 4 | 16 | 0.0% | 1.50 [0.52, 4.32] |
| Kuisma et al (2006) | 4 | 16 | 6 | 16 | 24.5% | 0.67 [0.23, 1.92] |
| NCT00414726 | 14 | 43 | 4 | 42 | 0.0% | 3.42 [1.22, 9.54] |
| Padma et al (2010) | 0 | 20 | 2 | 20 | 3.1% | 0.20 [0.01, 3.92] |
| Shi et al (2017) | 0 | 9 | 0 | 9 | | Not estimable |
| Singhal et al (2005) | 0 | 9 | 1 | 7 | 2.9% | 0.27 [0.01, 5.70] |
| Stub et al (2015) | 5 | 312 | 11 | 312 | 25.1% | 0.45 [0.16, 1.29] |
| Young et al (2014), HOT OR NOT | 5 | 9 | 4 | 8 | 33.3% | 1.11 [0.45, 2.75] |
| **Total (95% CI)** | | **460** | | **447** | **100.0%** | **0.69 [0.41, 1.17]** |
| Total events | 17 | | 27 | | | |

Heterogeneity: Tau² = 0.00; Chi² = 3.04, df = 5 (P = 0.69); I² = 0%
Test for overall effect: Z = 1.36 (P = 0.17)

| | | | | | | |
|---|---|---|---|---|---|---|
| **Total (95% CI)** | | **460** | | **447** | **100.0%** | **0.85 [0.50, 1.43]** |
| Total events | 19 | | 25 | | | |

Heterogeneity: Tau² = 0.00; Chi² = 4.58, df = 5 (P = 0.47); I² = 0%
Test for overall effect: Z = 0.62 (P = 0.53)

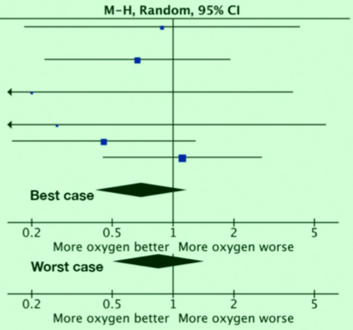


## IV: same as III but stroke only; N = 74

| Study or Subgroup | Liberal Events | Liberal Total | Conservative Events | Conservative Total | Weight | Risk Ratio M-H, Random, 95% CI |
|---|---|---|---|---|---|---|
| NCT00414726 | 14 | 43 | 4 | 42 | 0.0% | 3.42 [1.22, 9.54] |
| Padma et al (2010) | 0 | 20 | 2 | 20 | 51.4% | 0.20 [0.01, 3.92] |
| Shi et al (2017) | 0 | 9 | 0 | 9 | | Not estimable |
| Singhal et al (2005) | 0 | 9 | 1 | 7 | 48.6% | 0.27 [0.01, 5.70] |
| **Total (95% CI)** | | **38** | | **36** | **100.0%** | **0.23 [0.03, 1.94]** |
| Total events | 0 | | 3 | | | |

Heterogeneity: Tau² = 0.00; Chi² = 0.02, df = 1 (P = 0.89); I² = 0%
Test for overall effect: Z = 1.35 (P = 0.18)

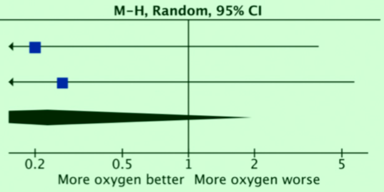

# Mortality at 30 days, any oxygen therapy

## I: as shown by Chu et al. Lancet. 2018 Apr 28;391(10131):1693-1705; N = 13,689

| Study or Subgroup | Liberal Events | Liberal Total | Conservative Events | Conservative Total | Weight | Risk Ratio M-H, Random, 95% CI |
|---|---|---|---|---|---|---|
| Ali et al (2014) | 13 | 155 | 11 | 146 | 4.2% | 1.11 [0.52, 2.41] |
| Hofmann et al (2017), DETO2X-AMI | 73 | 3311 | 67 | 3318 | 23.2% | 1.09 [0.79, 1.52] |
| NCT00414726 | 14 | 43 | 4 | 42 | 2.4% | 3.42 [1.22, 9.54] |
| Ranchord et al (2012), OPTIMISE | 1 | 72 | 2 | 76 | 0.4% | 0.53 [0.05, 5.70] |
| Roffe et al (2017), SO2S | 138 | 2668 | 140 | 2668 | 47.6% | 0.99 [0.78, 1.24] |
| Rønning et al (1999) | 55 | 292 | 38 | 258 | 17.5% | 1.28 [0.88, 1.87] |
| Singhal et al (2005) | 1 | 9 | 1 | 7 | 0.4% | 0.78 [0.06, 10.37] |
| Stub et al (2015) | 12 | 312 | 14 | 312 | 4.4% | 0.86 [0.40, 1.82] |
| **Total (95% CI)** | | **6862** | | **6827** | **100.0%** | **1.08 [0.92, 1.27]** |
| Total events | 307 | | 277 | | | |

Heterogeneity: Tau² = 0.00; Chi² = 7.00, df = 7 (P = 0.43); I² = 0%
Test for overall effect: Z = 0.99 (P = 0.32)

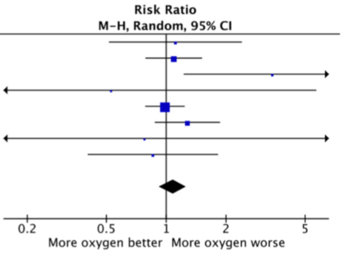


## II: same as I but numbers as reported in original publications; N = 16,124

| Study or Subgroup | Liberal Events | Liberal Total | Conservative Events | Conservative Total | Weight | Risk Ratio M-H, Random, 95% CI |
|---|---|---|---|---|---|---|
| Ali et al (2014) | 12 | 148 | 11 | 141 | 8.2% | 1.04 [0.47, 2.28] |
| Hofmann et al (2017), DETO2X-AMI | 73 | 3311 | 67 | 3318 | 24.0% | 1.09 [0.79, 1.52] |
| NCT00414726 | 14 | 43 | 4 | 42 | 5.3% | 3.42 [1.22, 9.54] |
| Ranchord et al (2012), OPTIMISE | 1 | 72 | 4 | 76 | 1.3% | 0.26 [0.03, 2.31] |
| Ranchord et al (2012), OPTIMISE | 3 | 72 | 2 | 76 | 0.0% | 1.58 [0.27, 9.20] |
| Roffe et al (2017), SO2S | 245 | 5301 | 140 | 2665 | 32.0% | 0.88 [0.72, 1.08] |
| Rønning et al (1999) | 55 | 292 | 38 | 258 | 21.2% | 1.28 [0.88, 1.87] |
| Singhal et al (2005) | 1 | 9 | 1 | 7 | 0.9% | 0.78 [0.06, 10.37] |
| Stub et al (2015) | 8 | 218 | 13 | 223 | 7.1% | 0.63 [0.27, 1.49] |
| **Total (95% CI)** | | **9394** | | **6730** | **100.0%** | **1.05 [0.81, 1.35]** Best case |
| Total events | 409 | | 278 | | | |

Heterogeneity: Tau² = 0.04; Chi² = 11.55, df = 7 (P = 0.12); I² = 39%
Test for overall effect: Z = 0.37 (P = 0.71)

| | | | | | | |
|---|---|---|---|---|---|---|
| **Total (95% CI)** | | **9394** | | **6730** | **100.0%** | **1.07 [0.85, 1.35]** Worst case |
| Total events | 411 | | 276 | | | |

Heterogeneity: Tau² = 0.03; Chi² = 10.37, df = 7 (P = 0.17); I² = 32%
Test for overall effect: Z = 0.55 (P = 0.59)

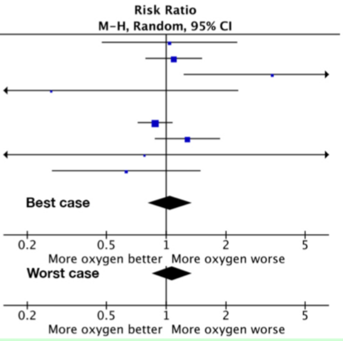


## III: all RCT; N = 16,375

| Study or Subgroup | Liberal Events | Liberal Total | Conservative Events | Conservative Total | Weight | Risk Ratio M-H, Random, 95% CI |
|---|---|---|---|---|---|---|
| Ali et al (2014) | 12 | 148 | 11 | 141 | 6.8% | 1.04 [0.47, 2.28] |
| Hofmann et al (2017), DETO2X-AMI | 73 | 3311 | 67 | 3318 | 23.0% | 1.09 [0.79, 1.52] |
| Khoshnood et al (2016), SOCCER | 3 | 85 | 3 | 75 | 1.9% | 0.88 [0.18, 4.24] |
| Mazdeh et al (2015) | 5 | 26 | 3 | 25 | 2.7% | 1.60 [0.43, 6.01] |
| NCT00414726 | 14 | 43 | 4 | 42 | 4.2% | 3.42 [1.22, 9.54] |
| Padma et al (2010) | 0 | 20 | 2 | 20 | 0.5% | 0.20 [0.01, 3.92] |
| Ranchord et al (2012), OPTIMISE | 1 | 72 | 4 | 76 | 1.0% | 0.26 [0.03, 2.31] |
| Ranchord et al (2012), OPTIMISE | 3 | 72 | 2 | 76 | 0.0% | 1.58 [0.27, 9.20] |
| Roffe et al (2017), SO2S | 245 | 5301 | 140 | 2665 | 33.5% | 0.88 [0.72, 1.08] |
| Rønning et al (1999) | 55 | 292 | 38 | 258 | 19.8% | 1.28 [0.88, 1.87] |
| Singhal et al (2005) | 1 | 9 | 1 | 7 | 0.7% | 0.78 [0.06, 10.37] |
| Stub et al (2015) | 8 | 218 | 13 | 223 | 5.8% | 0.63 [0.27, 1.49] |
| **Total (95% CI)** | | **9525** | | **6850** | **100.0%** | **1.04 [0.83, 1.30]** Best case |
| Total events | 417 | | 286 | | | |

Heterogeneity: Tau² = 0.03; Chi² = 13.19, df = 10 (P = 0.21); I² = 24%
Test for overall effect: Z = 0.35 (P = 0.73)

| | | | | | | |
|---|---|---|---|---|---|---|
| **Total (95% CI)** | | **9525** | | **6850** | **100.0%** | **1.05 [0.86, 1.28]** Worst case |
| Total events | 419 | | 284 | | | |

Heterogeneity: Tau² = 0.02; Chi² = 12.00, df = 10 (P = 0.28); I² = 17%
Test for overall effect: Z = 0.47 (P = 0.64)

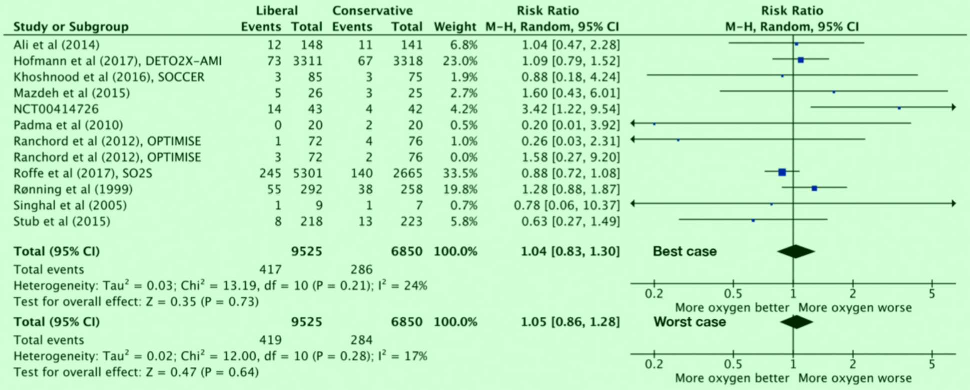


## IV: same as III but stroke only; N = 8,997

| Study or Subgroup | Liberal Events | Liberal Total | Conservative Events | Conservative Total | Weight | Risk Ratio M-H, Random, 95% CI |
|---|---|---|---|---|---|---|
| Ali et al (2014) | 12 | 148 | 11 | 141 | 13.6% | 1.04 [0.47, 2.28] |
| Mazdeh et al (2015) | 5 | 26 | 3 | 25 | 6.0% | 1.60 [0.43, 6.01] |
| NCT00414726 | 14 | 43 | 4 | 42 | 9.1% | 3.42 [1.22, 9.54] |
| Padma et al (2010) | 0 | 20 | 2 | 20 | 1.3% | 0.20 [0.01, 3.92] |
| Roffe et al (2017), SO2S | 245 | 5301 | 140 | 2665 | 39.0% | 0.88 [0.72, 1.08] |
| Rønning et al (1999) | 55 | 292 | 38 | 258 | 29.3% | 1.28 [0.88, 1.87] |
| Singhal et al (2005) | 1 | 9 | 1 | 7 | 1.7% | 0.78 [0.06, 10.37] |
| **Total (95% CI)** | | **5839** | | **3158** | **100.0%** | **1.15 [0.81, 1.63]** |
| Total events | 332 | | 199 | | | |

Heterogeneity: Tau² = 0.07; Chi² = 10.33, df = 6 (P = 0.11); I² = 42%
Test for overall effect: Z = 0.80 (P = 0.42)

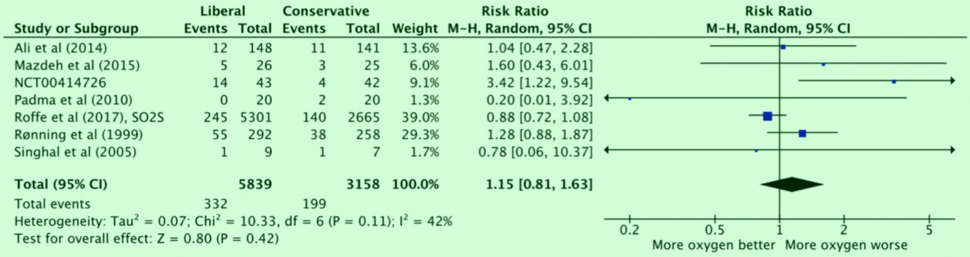

# Mortality at 30 days, any OT, w/o NCT00414726

### I: as shown by Chu et al. Lancet. 2018 Apr 28;391(10131):1693-1705; N = 13,604

| Study or Subgroup | Liberal Events | Liberal Total | Conservative Events | Conservative Total | Weight | Risk Ratio M-H, Random, 95% CI |
|---|---|---|---|---|---|---|
| Ali et al (2014) | 13 | 155 | 11 | 146 | 4.3% | 1.11 [0.52, 2.41] |
| Hofmann et al (2017), DETO2X-AMI | 73 | 3311 | 67 | 3318 | 23.7% | 1.09 [0.79, 1.52] |
| NCT00414726 | 14 | 43 | 4 | 42 | 0.0% | 3.42 [1.22, 9.54] |
| Ranchord et al (2012), OPTIMISE | 1 | 72 | 2 | 76 | 0.5% | 0.53 [0.05, 5.70] |
| Roffe et al (2017), SO2S | 138 | 2668 | 140 | 2668 | 48.8% | 0.99 [0.78, 1.24] |
| Rønning et al (1999) | 55 | 292 | 38 | 258 | 17.9% | 1.28 [0.88, 1.87] |
| Singhal et al (2005) | 1 | 9 | 1 | 7 | 0.4% | 0.78 [0.06, 10.37] |
| Stub et al (2015) | 12 | 312 | 14 | 312 | 4.5% | 0.86 [0.40, 1.82] |
| **Total (95% CI)** | | **6819** | | **6785** | **100.0%** | **1.05 [0.90, 1.24]** |
| Total events | 293 | | 273 | | | |

Heterogeneity: $Tau^2 = 0.00$; $Chi^2 = 2.07$, df = 6 (P = 0.91); $I^2 = 0\%$
Test for overall effect: Z = 0.63 (P = 0.53)

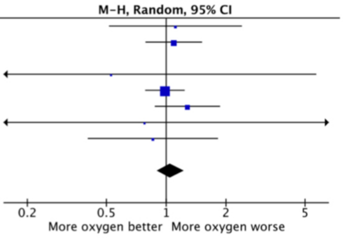


### II: same as I but numbers as reported in original publications; N = 16,039

| Study or Subgroup | Liberal Events | Liberal Total | Conservative Events | Conservative Total | Weight | Risk Ratio M-H, Random, 95% CI |
|---|---|---|---|---|---|---|
| Ali et al (2014) | 12 | 148 | 11 | 141 | 3.7% | 1.04 [0.47, 2.28] |
| Hofmann et al (2017), DETO2X-AMI | 73 | 3311 | 67 | 3318 | 21.1% | 1.09 [0.79, 1.52] |
| NCT00414726 | 14 | 43 | 4 | 42 | 0.0% | 3.42 [1.22, 9.54] |
| Ranchord et al (2012), OPTIMISE | 1 | 72 | 4 | 76 | 0.5% | 0.26 [0.03, 2.31] |
| Ranchord et al (2012), OPTIMISE | 3 | 72 | 2 | 76 | 0.0% | 1.58 [0.27, 9.20] |
| Roffe et al (2017), SO2S | 245 | 5301 | 140 | 2665 | 55.4% | 0.88 [0.72, 1.08] |
| Rønning et al (1999) | 55 | 292 | 38 | 258 | 15.9% | 1.28 [0.88, 1.87] |
| Singhal et al (2005) | 1 | 9 | 1 | 7 | 0.3% | 0.78 [0.06, 10.37] |
| Stub et al (2015) | 8 | 218 | 13 | 223 | 3.1% | 0.63 [0.27, 1.49] |
| **Total (95% CI)** | | **9351** | | **6688** | **100.0%** | **0.97 [0.83, 1.12]** Best case |
| Total events | 395 | | 274 | | | |

Heterogeneity: $Tau^2 = 0.00$; $Chi^2 = 5.86$, df = 6 (P = 0.44); $I^2 = 0\%$
Test for overall effect: Z = 0.44 (P = 0.66)

| | | | | | | |
|---|---|---|---|---|---|---|
| **Total (95% CI)** | | **9351** | | **6688** | **100.0%** | **0.98 [0.84, 1.14]** Worst case |
| Total events | 397 | | 272 | | | |

Heterogeneity: $Tau^2 = 0.00$; $Chi^2 = 4.77$, df = 6 (P = 0.57); $I^2 = 0\%$
Test for overall effect: Z = 0.31 (P = 0.76)

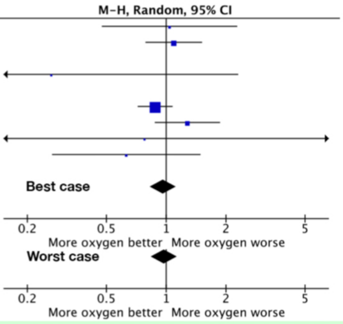


### III: all RCT; N = 16,290

| Study or Subgroup | Liberal Events | Liberal Total | Conservative Events | Conservative Total | Weight | Risk Ratio M-H, Random, 95% CI |
|---|---|---|---|---|---|---|
| Ali et al (2014) | 12 | 148 | 11 | 141 | 3.6% | 1.04 [0.47, 2.28] |
| Hofmann et al (2017), DETO2X-AMI | 73 | 3311 | 67 | 3318 | 20.6% | 1.09 [0.79, 1.52] |
| Khoshnood et al (2016), SOCCER | 3 | 85 | 3 | 75 | 0.9% | 0.88 [0.18, 4.24] |
| Mazdeh et al (2015) | 5 | 26 | 3 | 25 | 1.3% | 1.60 [0.43, 6.01] |
| NCT00414726 | 14 | 43 | 4 | 42 | 0.0% | 3.42 [1.22, 9.54] |
| Padma et al (2010) | 0 | 20 | 2 | 20 | 0.3% | 0.20 [0.01, 3.92] |
| Ranchord et al (2012), OPTIMISE | 1 | 72 | 4 | 76 | 0.5% | 0.26 [0.03, 2.31] |
| Ranchord et al (2012), OPTIMISE | 3 | 72 | 2 | 76 | 0.0% | 1.58 [0.27, 9.20] |
| Roffe et al (2017), SO2S | 245 | 5301 | 140 | 2665 | 54.1% | 0.88 [0.72, 1.08] |
| Rønning et al (1999) | 55 | 292 | 38 | 258 | 15.5% | 1.28 [0.88, 1.87] |
| Singhal et al (2005) | 1 | 9 | 1 | 7 | 0.3% | 0.78 [0.06, 10.37] |
| Stub et al (2015) | 8 | 218 | 13 | 223 | 3.0% | 0.63 [0.27, 1.49] |
| **Total (95% CI)** | | **9482** | | **6808** | **100.0%** | **0.97 [0.83, 1.12]** Best case |
| Total events | 403 | | 282 | | | |

Heterogeneity: $Tau^2 = 0.00$; $Chi^2 = 7.52$, df = 9 (P = 0.58); $I^2 = 0\%$
Test for overall effect: Z = 0.42 (P = 0.68)

| | | | | | | |
|---|---|---|---|---|---|---|
| **Total (95% CI)** | | **9482** | | **6808** | **100.0%** | **0.98 [0.84, 1.13]** Worst case |
| Total events | 405 | | 280 | | | |

Heterogeneity: $Tau^2 = 0.00$; $Chi^2 = 6.42$, df = 9 (P = 0.70); $I^2 = 0\%$
Test for overall effect: Z = 0.29 (P = 0.77)

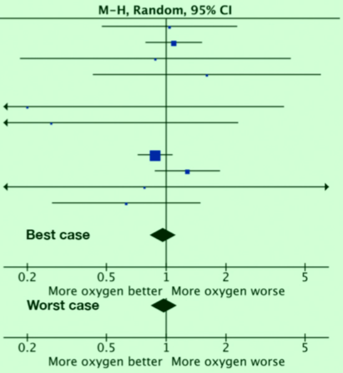


### IV: same as III but stroke only; N = 8,912

| Study or Subgroup | Liberal Events | Liberal Total | Conservative Events | Conservative Total | Weight | Risk Ratio M-H, Random, 95% CI |
|---|---|---|---|---|---|---|
| Ali et al (2014) | 12 | 148 | 11 | 141 | 4.8% | 1.04 [0.47, 2.28] |
| Mazdeh et al (2015) | 5 | 26 | 3 | 25 | 1.7% | 1.60 [0.43, 6.01] |
| NCT00414726 | 14 | 43 | 4 | 42 | 0.0% | 3.42 [1.22, 9.54] |
| Padma et al (2010) | 0 | 20 | 2 | 20 | 0.3% | 0.20 [0.01, 3.92] |
| Roffe et al (2017), SO2S | 245 | 5301 | 140 | 2665 | 72.1% | 0.88 [0.72, 1.08] |
| Rønning et al (1999) | 55 | 292 | 38 | 258 | 20.7% | 1.28 [0.88, 1.87] |
| Singhal et al (2005) | 1 | 9 | 1 | 7 | 0.4% | 0.78 [0.06, 10.37] |
| **Total (95% CI)** | | **5796** | | **3116** | **100.0%** | **0.96 [0.81, 1.14]** |
| Total events | 318 | | 195 | | | |

Heterogeneity: $Tau^2 = 0.00$; $Chi^2 = 4.64$, df = 5 (P = 0.46); $I^2 = 0\%$
Test for overall effect: Z = 0.44 (P = 0.66)

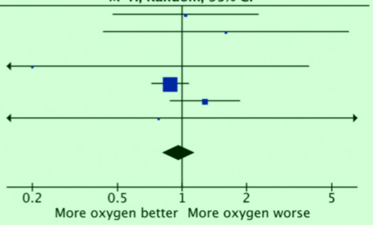

# Mortality at 30 days, FiO2 > 0.5

### I: as shown by Chu et al. Lancet. 2018 Apr 28;391(10131):1693-1705; N = 725

| Study or Subgroup | Liberal Events | Liberal Total | Conservative Events | Conservative Total | Weight | Risk Ratio M-H, Random, 95% CI |
| --- | --- | --- | --- | --- | --- | --- |
| NCT00414726 | 14 | 43 | 4 | 42 | 39.4% | 3.42 [1.22, 9.54] |
| Singhal et al (2005) | 1 | 9 | 1 | 7 | 13.4% | 0.78 [0.06, 10.37] |
| Stub et al (2015) | 12 | 312 | 14 | 312 | 47.2% | 0.86 [0.40, 1.82] |
| **Total (95% CI)** | | **364** | | **361** | **100.0%** | **1.46 [0.50, 4.27]** |
| Total events | 27 | | 19 | | | |

Heterogeneity: $Tau^2 = 0.49$; $Chi^2 = 4.72$, df = 2 (P = 0.09); $I^2 = 58\%$
Test for overall effect: Z = 0.69 (P = 0.49)

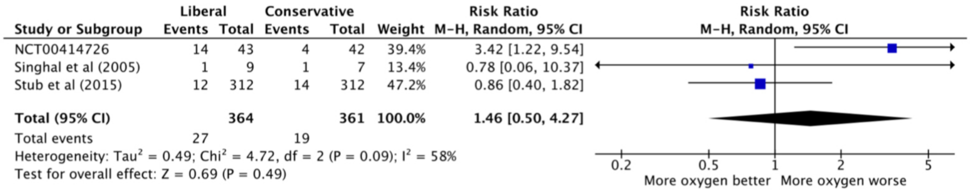


### II: same as I but numbers as reported in original publications; N = 542

| Study or Subgroup | Liberal Events | Liberal Total | Conservative Events | Conservative Total | Weight | Risk Ratio M-H, Random, 95% CI |
| --- | --- | --- | --- | --- | --- | --- |
| NCT00414726 | 14 | 43 | 4 | 42 | 39.9% | 3.42 [1.22, 9.54] |
| Singhal et al (2005) | 1 | 9 | 1 | 7 | 17.1% | 0.78 [0.06, 10.37] |
| Stub et al (2015) | 8 | 218 | 13 | 223 | 43.0% | 0.63 [0.27, 1.49] |
| **Total (95% CI)** | | **270** | | **272** | **100.0%** | **1.28 [0.35, 4.72]** |
| Total events | 23 | | 18 | | | |

Heterogeneity: $Tau^2 = 0.84$; $Chi^2 = 6.26$, df = 2 (P = 0.04); $I^2 = 68\%$
Test for overall effect: Z = 0.37 (P = 0.71)

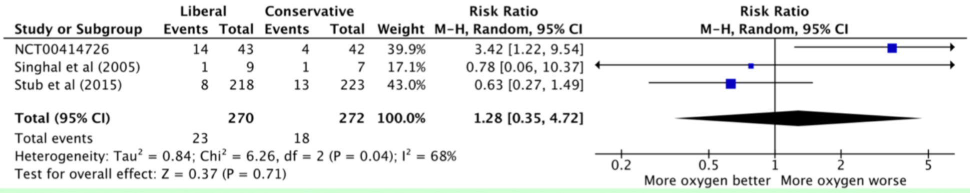


### III: all RCT; N = 742

| Study or Subgroup | Liberal Events | Liberal Total | Conservative Events | Conservative Total | Weight | Risk Ratio M-H, Random, 95% CI |
| --- | --- | --- | --- | --- | --- | --- |
| Khoshnood et al (2016), SOCCER | 3 | 85 | 3 | 75 | 19.8% | 0.88 [0.18, 4.24] |
| NCT00414726 | 14 | 43 | 4 | 42 | 29.3% | 3.42 [1.22, 9.54] |
| Padma et al (2010) | 0 | 20 | 2 | 20 | 8.0% | 0.20 [0.01, 3.92] |
| Singhal et al (2005) | 1 | 9 | 1 | 7 | 10.0% | 0.78 [0.06, 10.37] |
| Stub et al (2015) | 8 | 218 | 13 | 223 | 32.9% | 0.63 [0.27, 1.49] |
| **Total (95% CI)** | | **375** | | **367** | **100.0%** | **1.03 [0.41, 2.60]** |
| Total events | 26 | | 23 | | | |

Heterogeneity: $Tau^2 = 0.49$; $Chi^2 = 7.71$, df = 4 (P = 0.10); $I^2 = 48\%$
Test for overall effect: Z = 0.06 (P = 0.95)

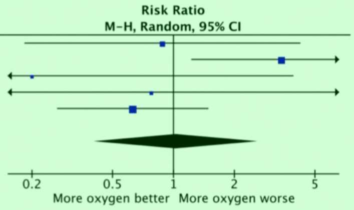


### IV: same as III but stroke only; N = 141

| Study or Subgroup | Liberal Events | Liberal Total | Conservative Events | Conservative Total | Weight | Risk Ratio M-H, Random, 95% CI |
| --- | --- | --- | --- | --- | --- | --- |
| NCT00414726 | 14 | 43 | 4 | 42 | 52.9% | 3.42 [1.22, 9.54] |
| Padma et al (2010) | 0 | 20 | 2 | 20 | 21.4% | 0.20 [0.01, 3.92] |
| Singhal et al (2005) | 1 | 9 | 1 | 7 | 25.6% | 0.78 [0.06, 10.37] |
| **Total (95% CI)** | | **72** | | **69** | **100.0%** | **1.27 [0.24, 6.80]** |
| Total events | 15 | | 7 | | | |

Heterogeneity: $Tau^2 = 1.11$; $Chi^2 = 3.87$, df = 2 (P = 0.14); $I^2 = 48\%$
Test for overall effect: Z = 0.28 (P = 0.78)

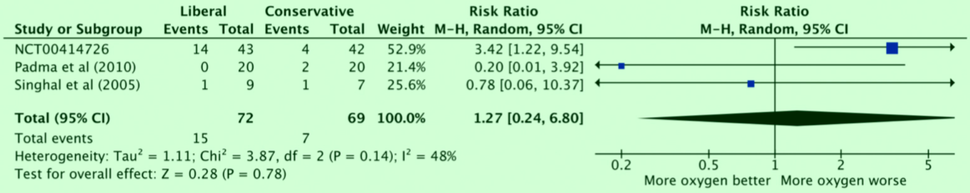

# Mortality at 30 days, FiO2 > 0.5, w/o NCT00414726

### I: as shown by Chu et al. Lancet. 2018 Apr 28;391(10131):1693-1705; N = 640

| Study or Subgroup | Liberal Events | Liberal Total | Conservative Events | Conservative Total | Weight | Risk Ratio M-H, Random, 95% CI |
|---|---|---|---|---|---|---|
| NCT00414726 | 14 | 43 | 4 | 42 | 0.0% | 3.42 [1.22, 9.54] |
| Singhal et al (2005) | 1 | 9 | 1 | 7 | 7.8% | 0.78 [0.06, 10.37] |
| Stub et al (2015) | 12 | 312 | 14 | 312 | 92.2% | 0.86 [0.40, 1.82] |
| **Total (95% CI)** | | **321** | | **319** | **100.0%** | **0.85 [0.41, 1.76]** |
| Total events | 13 | | 15 | | | |

Heterogeneity: Tau² = 0.00; Chi² = 0.00, df = 1 (P = 0.94); I² = 0%
Test for overall effect: Z = 0.44 (P = 0.66)

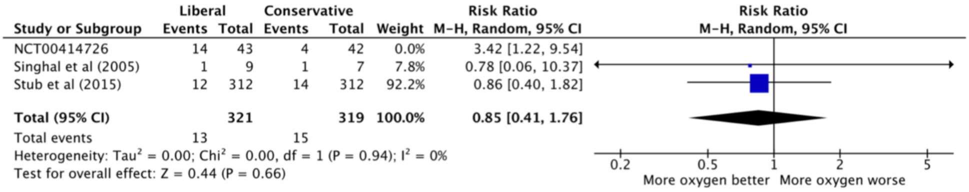


### II: same as I but numbers as reported in original publications; N = 457

| Study or Subgroup | Liberal Events | Liberal Total | Conservative Events | Conservative Total | Weight | Risk Ratio M-H, Random, 95% CI |
|---|---|---|---|---|---|---|
| NCT00414726 | 14 | 43 | 4 | 42 | 0.0% | 3.42 [1.22, 9.54] |
| Singhal et al (2005) | 1 | 9 | 1 | 7 | 9.9% | 0.78 [0.06, 10.37] |
| Stub et al (2015) | 8 | 218 | 13 | 223 | 90.1% | 0.63 [0.27, 1.49] |
| **Total (95% CI)** | | **227** | | **230** | **100.0%** | **0.64 [0.28, 1.45]** |
| Total events | 9 | | 14 | | | |

Heterogeneity: Tau² = 0.00; Chi² = 0.02, df = 1 (P = 0.88); I² = 0%
Test for overall effect: Z = 1.06 (P = 0.29)

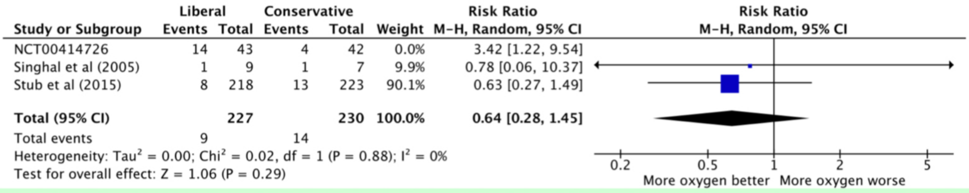


### III: all RCT; N = 657

| Study or Subgroup | Liberal Events | Liberal Total | Conservative Events | Conservative Total | Weight | Risk Ratio M-H, Random, 95% CI |
|---|---|---|---|---|---|---|
| Khoshnood et al (2016), SOCCER | 3 | 85 | 3 | 75 | 20.1% | 0.88 [0.18, 4.24] |
| NCT00414726 | 14 | 43 | 4 | 42 | 0.0% | 3.42 [1.22, 9.54] |
| Padma et al (2010) | 0 | 20 | 2 | 20 | 5.6% | 0.20 [0.01, 3.92] |
| Singhal et al (2005) | 1 | 9 | 1 | 7 | 7.4% | 0.78 [0.06, 10.37] |
| Stub et al (2015) | 8 | 218 | 13 | 223 | 66.9% | 0.63 [0.27, 1.49] |
| **Total (95% CI)** | | **332** | | **325** | **100.0%** | **0.64 [0.32, 1.30]** |
| Total events | 12 | | 19 | | | |

Heterogeneity: Tau² = 0.00; Chi² = 0.78, df = 3 (P = 0.86); I² = 0%
Test for overall effect: Z = 1.23 (P = 0.22)

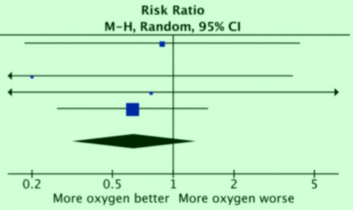


### IV: same as III but stroke only; N = 56

| Study or Subgroup | Liberal Events | Liberal Total | Conservative Events | Conservative Total | Weight | Risk Ratio M-H, Random, 95% CI |
|---|---|---|---|---|---|---|
| NCT00414726 | 14 | 43 | 4 | 42 | 0.0% | 3.42 [1.22, 9.54] |
| Padma et al (2010) | 0 | 20 | 2 | 20 | 43.1% | 0.20 [0.01, 3.92] |
| Singhal et al (2005) | 1 | 9 | 1 | 7 | 56.9% | 0.78 [0.06, 10.37] |
| **Total (95% CI)** | | **29** | | **27** | **100.0%** | **0.43 [0.06, 3.06]** |
| Total events | 1 | | 3 | | | |

Heterogeneity: Tau² = 0.00; Chi² = 0.47, df = 1 (P = 0.49); I² = 0%
Test for overall effect: Z = 0.84 (P = 0.40)

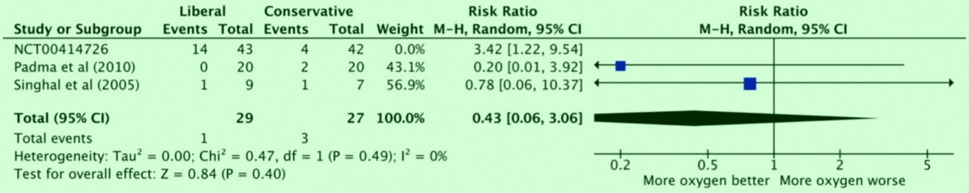

# Mortality at 90 days, any oxygen therapy

## I: all RCT; N = 15,938

| Study or Subgroup | Liberal Events | Liberal Total | Conservative Events | Conservative Total | Weight | Risk Ratio M-H, Random, 95% CI |
|---|---|---|---|---|---|---|
| Ali et al (2014) | 18 | 148 | 17 | 141 | 4.0% | 1.01 [0.54, 1.88] |
| Hofmann et al (2017), DETO2X-AMI | 93 | 3311 | 94 | 3318 | 18.3% | 0.99 [0.75, 1.32] |
| Khoshnood et al (2016), SOCCER | 3 | 85 | 3 | 75 | 0.6% | 0.88 [0.18, 4.24] |
| Mazdeh et al (2015) | 5 | 26 | 3 | 25 | 0.9% | 1.60 [0.43, 6.01] |
| NCT00414726 | 17 | 43 | 7 | 42 | 2.6% | 2.37 [1.10, 5.13] |
| Padma et al (2010) | 0 | 20 | 2 | 20 | 0.2% | 0.20 [0.01, 3.92] |
| Roffe et al (2017), SO2S | 493 | 5128 | 246 | 2549 | 57.8% | 1.00 [0.86, 1.15] |
| Rønning et al (1999) | 66 | 292 | 46 | 258 | 13.1% | 1.27 [0.90, 1.78] |
| Singhal et al (2005) | 2 | 9 | 1 | 7 | 0.3% | 1.56 [0.17, 13.87] |
| Stub et al (2015) | 8 | 218 | 13 | 223 | 2.1% | 0.63 [0.27, 1.49] |
| **Total (95% CI)** | | **9280** | | **6658** | **100.0%** | **1.04 [0.92, 1.18]** |
| Total events | 705 | | 432 | | | |

Heterogeneity: Tau² = 0.00; Chi² = 9.26, df = 9 (P = 0.41); I² = 3%
Test for overall effect: Z = 0.67 (P = 0.51)

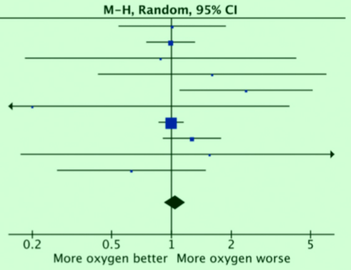


## II: same as I but stroke only; N = 8,708

| Study or Subgroup | Liberal Events | Liberal Total | Conservative Events | Conservative Total | Weight | Risk Ratio M-H, Random, 95% CI |
|---|---|---|---|---|---|---|
| Ali et al (2014) | 18 | 148 | 17 | 141 | 10.6% | 1.01 [0.54, 1.88] |
| Mazdeh et al (2015) | 5 | 26 | 3 | 25 | 2.7% | 1.60 [0.43, 6.01] |
| NCT00414726 | 17 | 43 | 7 | 42 | 7.3% | 2.37 [1.10, 5.13] |
| Padma et al (2010) | 0 | 20 | 2 | 20 | 0.5% | 0.20 [0.01, 3.92] |
| Roffe et al (2017), SO2S | 493 | 5128 | 246 | 2549 | 51.7% | 1.00 [0.86, 1.15] |
| Rønning et al (1999) | 66 | 292 | 46 | 258 | 26.1% | 1.27 [0.90, 1.78] |
| Singhal et al (2005) | 2 | 9 | 1 | 7 | 1.0% | 1.56 [0.17, 13.87] |
| **Total (95% CI)** | | **5666** | | **3042** | **100.0%** | **1.14 [0.92, 1.42]** |
| Total events | 601 | | 322 | | | |

Heterogeneity: Tau² = 0.02; Chi² = 7.71, df = 6 (P = 0.26); I² = 22%
Test for overall effect: Z = 1.17 (P = 0.24)

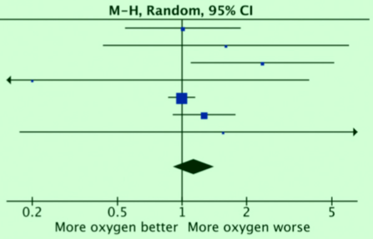

# Mortality at 90 days, any OT, w/o NCT00414726

## I: all RCT; N = 15,853

| Study or Subgroup | Liberal Events | Liberal Total | Conservative Events | Conservative Total | Weight | Risk Ratio M-H, Random, 95% CI |
|---|---|---|---|---|---|---|
| Ali et al (2014) | 18 | 148 | 17 | 141 | 3.5% | 1.01 [0.54, 1.88] |
| Hofmann et al (2017), DETO2X-AMI | 93 | 3311 | 94 | 3318 | 17.0% | 0.99 [0.75, 1.32] |
| Khoshnood et al (2016), SOCCER | 3 | 85 | 3 | 75 | 0.5% | 0.88 [0.18, 4.24] |
| Mazdeh et al (2015) | 5 | 26 | 3 | 25 | 0.8% | 1.60 [0.43, 6.01] |
| NCT00414726 | 17 | 43 | 7 | 42 | 0.0% | 2.37 [1.10, 5.13] |
| Padma et al (2010) | 0 | 20 | 2 | 20 | 0.2% | 0.20 [0.01, 3.92] |
| Roffe et al (2017), SO2S | 493 | 5128 | 246 | 2549 | 64.0% | 1.00 [0.86, 1.15] |
| Rønning et al (1999) | 66 | 292 | 46 | 258 | 11.9% | 1.27 [0.90, 1.78] |
| Singhal et al (2005) | 2 | 9 | 1 | 7 | 0.3% | 1.56 [0.17, 13.87] |
| Stub et al (2015) | 8 | 218 | 13 | 223 | 1.8% | 0.63 [0.27, 1.49] |
| **Total (95% CI)** | | **9237** | | **6616** | **100.0%** | **1.02 [0.91, 1.14]** |
| Total events | 688 | | 425 | | | |

Heterogeneity: Tau² = 0.00; Chi² = 4.73, df = 8 (P = 0.79); I² = 0%
Test for overall effect: Z = 0.30 (P = 0.76)

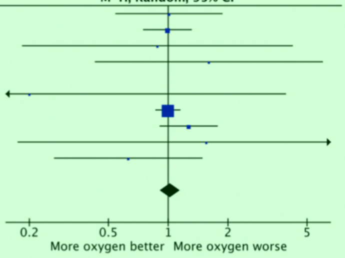


## II: same as I but stroke only; N = 8,623

| Study or Subgroup | Liberal Events | Liberal Total | Conservative Events | Conservative Total | Weight | Risk Ratio M-H, Random, 95% CI |
|---|---|---|---|---|---|---|
| Ali et al (2014) | 18 | 148 | 17 | 141 | 4.3% | 1.01 [0.54, 1.88] |
| Mazdeh et al (2015) | 5 | 26 | 3 | 25 | 1.0% | 1.60 [0.43, 6.01] |
| NCT00414726 | 17 | 43 | 7 | 42 | 0.0% | 2.37 [1.10, 5.13] |
| Padma et al (2010) | 0 | 20 | 2 | 20 | 0.2% | 0.20 [0.01, 3.92] |
| Roffe et al (2017), SO2S | 493 | 5128 | 246 | 2549 | 79.4% | 1.00 [0.86, 1.15] |
| Rønning et al (1999) | 66 | 292 | 46 | 258 | 14.8% | 1.27 [0.90, 1.78] |
| Singhal et al (2005) | 2 | 9 | 1 | 7 | 0.4% | 1.56 [0.17, 13.87] |
| **Total (95% CI)** | | **5623** | | **3000** | **100.0%** | **1.04 [0.91, 1.18]** |
| Total events | 584 | | 315 | | | |

Heterogeneity: Tau² = 0.00; Chi² = 3.39, df = 5 (P = 0.64); I² = 0%
Test for overall effect: Z = 0.54 (P = 0.59)

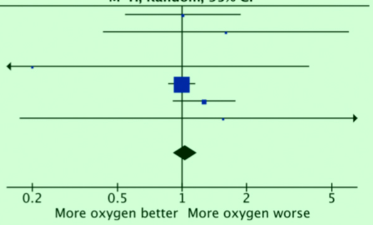

## Mortality at 90 days, FiO2 > 0.5

### I: all RCT; N = 742

| Study or Subgroup | Liberal Events | Liberal Total | Conservative Events | Conservative Total | Weight | Risk Ratio M-H, Random, 95% CI |
|---|---|---|---|---|---|---|
| Khoshnood et al (2016), SOCCER | 3 | 85 | 3 | 75 | 16.8% | 0.88 [0.18, 4.24] |
| NCT00414726 | 17 | 43 | 7 | 42 | 34.8% | 2.37 [1.10, 5.13] |
| Padma et al (2010) | 0 | 20 | 2 | 20 | 6.1% | 0.20 [0.01, 3.92] |
| Singhal et al (2005) | 2 | 9 | 1 | 7 | 10.2% | 1.56 [0.17, 13.87] |
| Stub et al (2015) | 8 | 218 | 13 | 223 | 32.1% | 0.63 [0.27, 1.49] |
| **Total (95% CI)** | | **375** | | **367** | **100.0%** | **1.08 [0.50, 2.36]** |
| Total events | 30 | | 26 | | | |

Heterogeneity: $Tau^2 = 0.30$; $Chi^2 = 6.83$, df = 4 (P = 0.15); $I^2 = 41\%$
Test for overall effect: Z = 0.20 (P = 0.84)

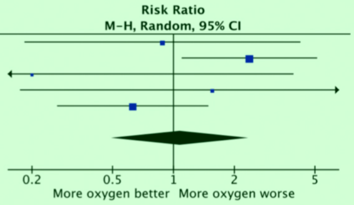


### II: same as I but stroke only; N = 141

| Study or Subgroup | Liberal Events | Liberal Total | Conservative Events | Conservative Total | Weight | Risk Ratio M-H, Random, 95% CI |
|---|---|---|---|---|---|---|
| NCT00414726 | 17 | 43 | 7 | 42 | 68.6% | 2.37 [1.10, 5.13] |
| Padma et al (2010) | 0 | 20 | 2 | 20 | 11.7% | 0.20 [0.01, 3.92] |
| Singhal et al (2005) | 2 | 9 | 1 | 7 | 19.8% | 1.56 [0.17, 13.87] |
| **Total (95% CI)** | | **72** | | **69** | **100.0%** | **1.63 [0.56, 4.81]** |
| Total events | 19 | | 10 | | | |

Heterogeneity: $Tau^2 = 0.29$; $Chi^2 = 2.62$, df = 2 (P = 0.27); $I^2 = 24\%$
Test for overall effect: Z = 0.89 (P = 0.37)

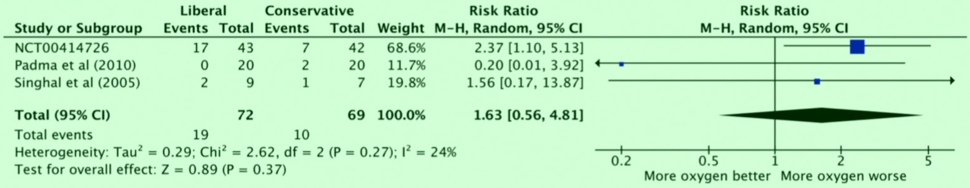

# Mortality at 90 days, FiO2 > 0.5, w/o NCT00414726

## I: all RCT; N = 657

| Study or Subgroup | Liberal Events | Liberal Total | Conservative Events | Conservative Total | Weight | Risk Ratio M-H, Random, 95% CI |
|---|---|---|---|---|---|---|
| Khoshnood et al (2016), SOCCER | 3 | 85 | 3 | 75 | 19.5% | 0.88 [0.18, 4.24] |
| NCT00414726 | 17 | 43 | 7 | 42 | 0.0% | 2.37 [1.10, 5.13] |
| Padma et al (2010) | 0 | 20 | 2 | 20 | 5.4% | 0.20 [0.01, 3.92] |
| Singhal et al (2005) | 2 | 9 | 1 | 7 | 10.1% | 1.56 [0.17, 13.87] |
| Stub et al (2015) | 8 | 218 | 13 | 223 | 65.0% | 0.63 [0.27, 1.49] |
| **Total (95% CI)** | | **332** | | **325** | **100.0%** | **0.69 [0.35, 1.38]** |
| Total events | 13 | | 19 | | | |

Heterogeneity: $Tau^2 = 0.00$; $Chi^2 = 1.34$, df = 3 (P = 0.72); $I^2 = 0\%$
Test for overall effect: Z = 1.04 (P = 0.30)

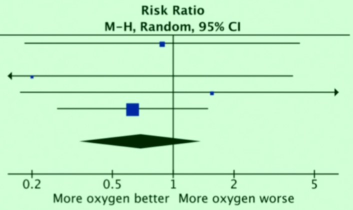


## II: same as I but stroke only; N = 56

| Study or Subgroup | Liberal Events | Liberal Total | Conservative Events | Conservative Total | Weight | Risk Ratio M-H, Random, 95% CI |
|---|---|---|---|---|---|---|
| NCT00414726 | 17 | 43 | 7 | 42 | 0.0% | 2.37 [1.10, 5.13] |
| Padma et al (2010) | 0 | 20 | 2 | 20 | 37.9% | 0.20 [0.01, 3.92] |
| Singhal et al (2005) | 2 | 9 | 1 | 7 | 62.1% | 1.56 [0.17, 13.87] |
| **Total (95% CI)** | | **29** | | **27** | **100.0%** | **0.71 [0.10, 5.23]** |
| Total events | 2 | | 3 | | | |

Heterogeneity: $Tau^2 = 0.42$; $Chi^2 = 1.23$, df = 1 (P = 0.27); $I^2 = 19\%$
Test for overall effect: Z = 0.33 (P = 0.74)

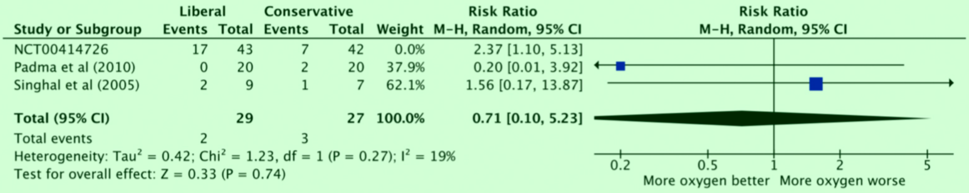

# Mortality at longest follow-up, any oxygen therapy

## I: as shown by Chu et al. Lancet. 2018 Apr 28;391(10131):1693-1705; N = 14,340

| | Liberal | | Conservative | | | Risk Ratio |
|---|---|---|---|---|---|---|
| **Study or Subgroup** | **Events** | **Total** | **Events** | **Total** | **Weight** | **M-H, Random, 95% CI** |
| Ali et al (2014) | 26 | 155 | 23 | 146 | 4.4% | 1.06 [0.64, 1.78] |
| Hofmann et al (2017), DETO2X-AMI | 166 | 3311 | 168 | 3318 | 26.3% | 0.99 [0.80, 1.22] |
| Khoshnood et al (2016), SOCCER | 3 | 85 | 3 | 75 | 0.5% | 0.88 [0.18, 4.24] |
| Kuisma et al (2006) | 4 | 14 | 4 | 14 | 0.8% | 1.00 [0.31, 3.23] |
| Mazdeh et al (2015) | 5 | 26 | 3 | 25 | 0.7% | 1.60 [0.43, 6.01] |
| NCT00414726 | 17 | 43 | 7 | 42 | 1.9% | 2.37 [1.10, 5.13] |
| Padma et al (2010) | 0 | 20 | 2 | 20 | 0.1% | 0.20 [0.01, 3.92] |
| Ranchord et al (2012), OPTIMISE | 1 | 72 | 2 | 76 | 0.2% | 0.53 [0.05, 5.70] |
| Rawles et al (1976) | 9 | 105 | 3 | 95 | 0.7% | 2.71 [0.76, 9.73] |
| Roffe et al (2017), SO2S | 268 | 2668 | 256 | 2668 | 43.4% | 1.05 [0.89, 1.23] |
| Rønning et al (1999) | 91 | 292 | 70 | 258 | 16.6% | 1.15 [0.88, 1.49] |
| Shi et al (2017) | 0 | 9 | 0 | 9 | | Not estimable |
| Singhal et al (2005) | 2 | 9 | 2 | 7 | 0.4% | 0.78 [0.14, 4.23] |
| Stub et al (2015) | 14 | 312 | 18 | 312 | 2.5% | 0.78 [0.39, 1.54] |
| Ukholkina et al (2005) | 1 | 58 | 0 | 79 | 0.1% | 4.07 [0.17, 98.10] |
| Young et al (2014), HOT OR NOT | 5 | 9 | 4 | 8 | 1.4% | 1.11 [0.45, 2.75] |
| **Total (95% CI)** | | **7188** | | **7152** | **100.0%** | **1.06 [0.96, 1.18]** |
| Total events | 612 | | 565 | | | |

Heterogeneity: $Tau^2 = 0.00$; $Chi^2 = 10.67$, df = 14 (P = 0.71); $I^2 = 0\%$
Test for overall effect: Z = 1.13 (P = 0.26)

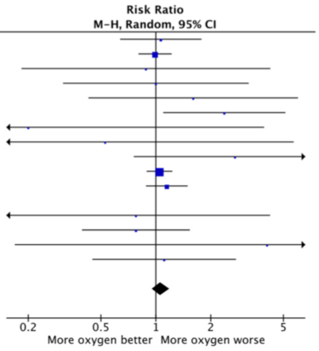


## II: same as I but numbers as reported in original publications; N = 16,457

| | Liberal | | Conservative | | | Risk Ratio |
|---|---|---|---|---|---|---|
| **Study or Subgroup** | **Events** | **Total** | **Events** | **Total** | **Weight** | **M-H, Random, 95% CI** |
| Ali et al (2014) | 22 | 126 | 21 | 130 | 3.5% | 1.08 [0.63, 1.86] |
| Hofmann et al (2017), DETO2X-AMI | 166 | 3311 | 168 | 3318 | 24.0% | 0.99 [0.80, 1.22] |
| Khoshnood et al (2016), SOCCER | 3 | 85 | 3 | 75 | 0.4% | 0.88 [0.18, 4.24] |
| Kuisma et al (2006) | 4 | 16 | 6 | 16 | 0.9% | 0.67 [0.23, 1.92] |
| Kuisma et al (2006) | 6 | 16 | 4 | 16 | 0.0% | 1.50 [0.52, 4.32] |
| Mazdeh et al (2015) | 5 | 26 | 3 | 25 | 0.6% | 1.60 [0.43, 6.01] |
| NCT00414726 | 17 | 43 | 7 | 42 | 1.8% | 2.37 [1.10, 5.13] |
| Padma et al (2010) | 0 | 20 | 2 | 20 | 0.1% | 0.20 [0.01, 3.92] |
| Ranchord et al (2012), OPTIMISE | 1 | 72 | 4 | 76 | 0.2% | 0.26 [0.03, 2.31] |
| Ranchord et al (2012), OPTIMISE | 3 | 72 | 2 | 76 | 0.0% | 1.58 [0.27, 9.20] |
| Rawles et al (1976) | 9 | 105 | 3 | 95 | 0.6% | 2.71 [0.76, 9.73] |
| Roffe et al (2017), SO2S | 493 | 5128 | 246 | 2549 | 49.6% | 1.00 [0.86, 1.15] |
| Rønning et al (1999) | 91 | 292 | 70 | 258 | 15.2% | 1.15 [0.88, 1.49] |
| Shi et al (2017) | 0 | 9 | 0 | 9 | | Not estimable |
| Singhal et al (2005) | 2 | 9 | 1 | 7 | 0.2% | 1.56 [0.17, 13.87] |
| Stub et al (2015) | 8 | 218 | 13 | 223 | 1.4% | 0.63 [0.27, 1.49] |
| Ukholkina et al (2005) | 1 | 58 | 0 | 79 | 0.1% | 4.07 [0.17, 98.10] |
| Young et al (2014), HOT OR NOT | 5 | 9 | 4 | 8 | 1.3% | 1.11 [0.45, 2.75] |
| **Total (95% CI)** | | **9527** | | **6930** | **100.0%** | **1.03 [0.93, 1.14]** |
| Total events | 827 | | 551 | | | |
| Heterogeneity: $Tau^2 = 0.00$; $Chi^2 = 13.69$, df = 14 (P = 0.47); $I^2 = 0\%$ | | | | | | |
| Test for overall effect: Z = 0.61 (P = 0.54) | | | | | | |
| **Total (95% CI)** | | **9527** | | **6930** | **100.0%** | **1.05 [0.94, 1.16]** |
| Total events | 831 | | 547 | | | |
| Heterogeneity: $Tau^2 = 0.00$; $Chi^2 = 12.16$, df = 14 (P = 0.59); $I^2 = 0\%$ | | | | | | |
| Test for overall effect: Z = 0.84 (P = 0.40) | | | | | | |

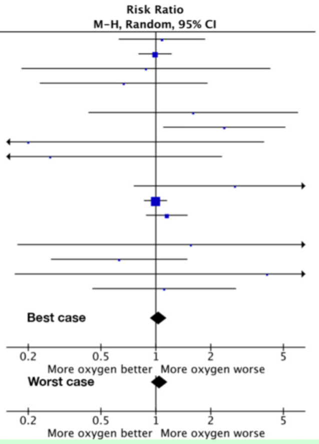


## III: all RCT; N = 16,566

| | Liberal | | Conservative | | | Risk Ratio |
|---|---|---|---|---|---|---|
| **Study or Subgroup** | **Events** | **Total** | **Events** | **Total** | **Weight** | **M-H, Random, 95% CI** |
| Ali et al (2014) | 22 | 126 | 21 | 130 | 3.5% | 1.08 [0.63, 1.86] |
| Chiu et al (2006) | 1 | 17 | 7 | 29 | 0.0% | 0.24 [0.03, 1.82] |
| Hofmann et al (2017), DETO2X-AMI | 166 | 3311 | 168 | 3318 | 23.9% | 0.99 [0.80, 1.22] |
| Khoshnood et al (2016), SOCCER | 3 | 85 | 3 | 75 | 0.4% | 0.88 [0.18, 4.24] |
| Kuisma et al (2006) | 4 | 16 | 6 | 16 | 0.9% | 0.67 [0.23, 1.92] |
| Kuisma et al (2006) | 6 | 16 | 4 | 16 | 0.0% | 1.50 [0.52, 4.32] |
| Mazdeh et al (2015) | 5 | 26 | 3 | 25 | 0.6% | 1.60 [0.43, 6.01] |
| NCT00414726 | 17 | 43 | 7 | 42 | 1.8% | 2.37 [1.10, 5.13] |
| Padma et al (2010) | 0 | 20 | 2 | 20 | 0.1% | 0.20 [0.01, 3.92] |
| Ranchord et al (2012), OPTIMISE | 1 | 72 | 4 | 76 | 0.2% | 0.26 [0.03, 2.31] |
| Ranchord et al (2012), OPTIMISE | 3 | 72 | 2 | 76 | 0.0% | 1.58 [0.27, 9.20] |
| Rawles et al (1976) | 9 | 105 | 3 | 95 | 0.6% | 2.71 [0.76, 9.73] |
| Roffe et al (2010) | 2 | 29 | 3 | 30 | 0.4% | 0.69 [0.12, 3.83] |
| Roffe et al (2017), SO2S | 493 | 5128 | 246 | 2549 | 49.3% | 1.00 [0.86, 1.15] |
| Rønning et al (1999) | 91 | 292 | 70 | 258 | 15.1% | 1.15 [0.88, 1.49] |
| Shi et al (2017) | 0 | 9 | 0 | 9 | | Not estimable |
| Singhal et al (2005) | 2 | 9 | 1 | 7 | 0.2% | 1.56 [0.17, 13.87] |
| Stub et al (2015) | 8 | 218 | 13 | 223 | 1.4% | 0.63 [0.27, 1.49] |
| Ukholkina et al (2005) | 1 | 58 | 0 | 79 | 0.1% | 4.07 [0.17, 98.10] |
| Wilson et al (1997) | 0 | 25 | 1 | 25 | 0.1% | 0.33 [0.01, 7.81] |
| Wilson et al (1997) | 1 | 25 | 0 | 25 | 0.0% | 3.00 [0.13, 70.30] |
| Young et al (2014), HOT OR NOT | 5 | 9 | 4 | 8 | 1.3% | 1.11 [0.45, 2.75] |
| **Total (95% CI)** | | **9581** | | **6985** | **100.0%** | **1.03 [0.93, 1.14]** |
| Total events | 829 | | 555 | | | |
| Heterogeneity: $Tau^2 = 0.00$; $Chi^2 = 14.39$, df = 16 (P = 0.57); $I^2 = 0\%$ | | | | | | |
| Test for overall effect: Z = 0.56 (P = 0.57) | | | | | | |
| **Total (95% CI)** | | **9581** | | **6985** | **100.0%** | **1.04 [0.94, 1.16]** |
| Total events | 834 | | 550 | | | |
| Heterogeneity: $Tau^2 = 0.00$; $Chi^2 = 12.81$, df = 16 (P = 0.69); $I^2 = 0\%$ | | | | | | |
| Test for overall effect: Z = 0.84 (P = 0.40) | | | | | | |

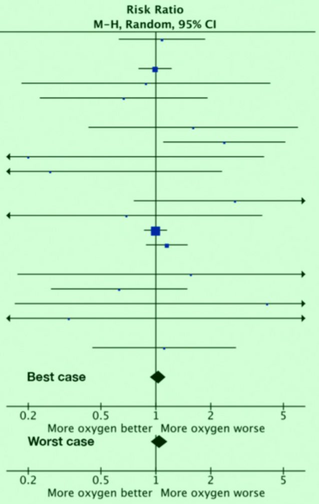


## IV: same as III but stroke only; N = 8,752

| | Liberal | | Conservative | | | Risk Ratio |
|---|---|---|---|---|---|---|
| **Study or Subgroup** | **Events** | **Total** | **Events** | **Total** | **Weight** | **M-H, Random, 95% CI** |
| Ali et al (2014) | 22 | 126 | 21 | 130 | 5.9% | 1.08 [0.63, 1.86] |
| Chiu et al (2006) | 1 | 17 | 7 | 29 | 0.0% | 0.24 [0.03, 1.82] |
| Mazdeh et al (2015) | 5 | 26 | 3 | 25 | 1.0% | 1.60 [0.43, 6.01] |
| NCT00414726 | 17 | 43 | 7 | 42 | 3.0% | 2.37 [1.10, 5.13] |
| Padma et al (2010) | 0 | 20 | 2 | 20 | 0.2% | 0.20 [0.01, 3.92] |
| Roffe et al (2010) | 2 | 29 | 3 | 30 | 0.6% | 0.69 [0.12, 3.83] |
| Roffe et al (2017), SO2S | 493 | 5128 | 246 | 2549 | 65.1% | 1.00 [0.86, 1.15] |
| Rønning et al (1999) | 91 | 292 | 70 | 258 | 23.8% | 1.15 [0.88, 1.49] |
| Shi et al (2017) | 0 | 9 | 0 | 9 | | Not estimable |
| Singhal et al (2005) | 2 | 9 | 1 | 7 | 0.4% | 1.56 [0.17, 13.87] |
| **Total (95% CI)** | | **5682** | | **3070** | **100.0%** | **1.06 [0.93, 1.22]** |
| Total events | 632 | | 353 | | | |

Heterogeneity: $Tau^2 = 0.00$; $Chi^2 = 7.21$, df = 7 (P = 0.41); $I^2 = 3\%$
Test for overall effect: Z = 0.91 (P = 0.37)

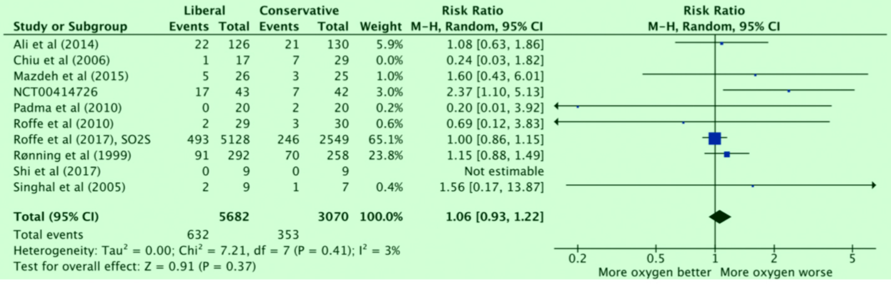

# Mortality at longest follow-up, any OT, w/o NCT00414726

## I: as shown by Chu et al. Lancet. 2018 Apr 28;391(10131):1693-1705; N = 14,255

| Study or Subgroup | Liberal Events | Liberal Total | Conservative Events | Conservative Total | Weight | Risk Ratio M-H, Random, 95% CI |
|---|---|---|---|---|---|---|
| Ali et al (2014) | 26 | 155 | 23 | 146 | 4.4% | 1.06 [0.64, 1.78] |
| Hofmann et al (2017), DETO2X-AMI | 166 | 3311 | 168 | 3318 | 26.8% | 0.99 [0.80, 1.22] |
| Khoshnood et al (2016), SOCCER | 3 | 85 | 3 | 75 | 0.5% | 0.88 [0.18, 4.24] |
| Kuisma et al (2006) | 4 | 14 | 4 | 14 | 0.9% | 1.00 [0.31, 3.23] |
| Mazdeh et al (2015) | 5 | 26 | 3 | 25 | 0.7% | 1.60 [0.43, 6.01] |
| NCT00414726 | 17 | 43 | 7 | 42 | 0.0% | 2.37 [1.10, 5.13] |
| Padma et al (2010) | 0 | 20 | 2 | 20 | 0.1% | 0.20 [0.01, 3.92] |
| Ranchord et al (2012), OPTIMISE | 1 | 72 | 2 | 76 | 0.2% | 0.53 [0.05, 5.70] |
| Rawles et al (1976) | 9 | 105 | 3 | 95 | 0.7% | 2.71 [0.76, 9.73] |
| Roffe et al (2017), SO2S | 268 | 2668 | 256 | 2668 | 44.3% | 1.05 [0.89, 1.23] |
| Rønning et al (1999) | 91 | 292 | 70 | 258 | 17.0% | 1.15 [0.88, 1.49] |
| Shi et al (2017) | 0 | 9 | 0 | 9 | | Not estimable |
| Singhal et al (2005) | 2 | 9 | 2 | 7 | 0.4% | 0.78 [0.14, 4.23] |
| Stub et al (2015) | 14 | 312 | 18 | 312 | 2.5% | 0.78 [0.39, 1.54] |
| Ukholkina et al (2005) | 1 | 58 | 0 | 79 | 0.1% | 4.07 [0.17, 98.10] |
| Young et al (2014), HOT OR NOT | 5 | 9 | 4 | 8 | 1.4% | 1.11 [0.45, 2.75] |
| **Total (95% CI)** | | **7145** | | **7110** | **100.0%** | **1.05 [0.94, 1.17]** |
| Total events | 595 | | 558 | | | |

Heterogeneity: $Tau^2 = 0.00$; $Chi^2 = 6.42$, df = 13 (P = 0.93); $I^2 = 0\%$
Test for overall effect: Z = 0.83 (P = 0.41)

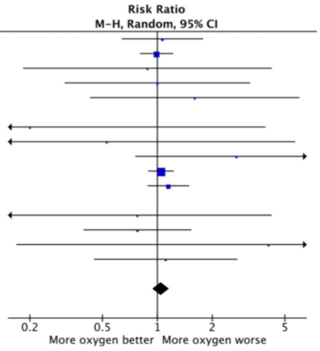


## II: same as I but numbers as reported in original publications; N = 16,372

| Study or Subgroup | Liberal Events | Liberal Total | Conservative Events | Conservative Total | Weight | Risk Ratio M-H, Random, 95% CI |
|---|---|---|---|---|---|---|
| Ali et al (2014) | 22 | 126 | 21 | 130 | 3.6% | 1.08 [0.63, 1.86] |
| Hofmann et al (2017), DETO2X-AMI | 166 | 3311 | 168 | 3318 | 24.4% | 0.99 [0.80, 1.22] |
| Khoshnood et al (2016), SOCCER | 3 | 85 | 3 | 75 | 0.4% | 0.88 [0.18, 4.24] |
| Kuisma et al (2006) | 6 | 16 | 4 | 16 | 0.0% | 1.50 [0.52, 4.32] |
| Kuisma et al (2006) | 4 | 16 | 6 | 16 | 1.0% | 0.67 [0.23, 1.92] |
| Mazdeh et al (2015) | 5 | 26 | 3 | 25 | 0.6% | 1.60 [0.43, 6.01] |
| NCT00414726 | 17 | 43 | 7 | 42 | 0.0% | 2.37 [1.10, 5.13] |
| Padma et al (2010) | 0 | 20 | 2 | 20 | 0.1% | 0.20 [0.01, 3.92] |
| Ranchord et al (2012), OPTIMISE | 3 | 72 | 2 | 76 | 0.0% | 1.58 [0.27, 9.20] |
| Ranchord et al (2012), OPTIMISE | 1 | 72 | 4 | 76 | 0.2% | 0.26 [0.03, 2.31] |
| Rawles et al (1976) | 9 | 105 | 3 | 95 | 0.7% | 2.71 [0.76, 9.73] |
| Roffe et al (2017), SO2S | 493 | 5128 | 246 | 2549 | 50.5% | 1.00 [0.86, 1.15] |
| Rønning et al (1999) | 91 | 292 | 70 | 258 | 15.5% | 1.15 [0.88, 1.49] |
| Shi et al (2017) | 0 | 9 | 0 | 9 | | Not estimable |
| Singhal et al (2005) | 2 | 9 | 1 | 7 | 0.2% | 1.56 [0.17, 13.87] |
| Stub et al (2015) | 8 | 218 | 13 | 223 | 1.4% | 0.63 [0.27, 1.49] |
| Ukholkina et al (2005) | 1 | 58 | 0 | 79 | 0.1% | 4.07 [0.17, 98.10] |
| Young et al (2014), HOT OR NOT | 5 | 9 | 4 | 8 | 1.3% | 1.11 [0.45, 2.75] |
| **Total (95% CI)** | | **9484** | | **6888** | **100.0%** | **1.02 [0.92, 1.13]** |
| Total events | 810 | | 544 | | | |

Heterogeneity: $Tau^2 = 0.00$; $Chi^2 = 9.13$, df = 13 (P = 0.76); $I^2 = 0\%$
Test for overall effect: Z = 0.32 (P = 0.75)

| | | | | | | |
|---|---|---|---|---|---|---|
| **Total (95% CI)** | | **9484** | | **6888** | **100.0%** | **1.03 [0.93, 1.14]** |
| Total events | 814 | | 540 | | | |

Heterogeneity: $Tau^2 = 0.00$; $Chi^2 = 7.73$, df = 13 (P = 0.86); $I^2 = 0\%$
Test for overall effect: Z = 0.56 (P = 0.58)

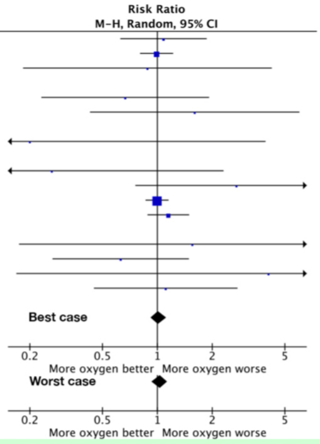


## III: all RCT; N = 16,481

| Study or Subgroup | Liberal Events | Liberal Total | Conservative Events | Conservative Total | Weight | Risk Ratio M-H, Random, 95% CI |
|---|---|---|---|---|---|---|
| Ali et al (2014) | 22 | 126 | 21 | 130 | 3.6% | 1.08 [0.63, 1.86] |
| Chiu et al (2006) | 1 | 17 | 7 | 29 | 0.0% | 0.24 [0.03, 1.82] |
| Hofmann et al (2017), DETO2X-AMI | 166 | 3311 | 168 | 3318 | 24.3% | 0.99 [0.80, 1.22] |
| Khoshnood et al (2016), SOCCER | 3 | 85 | 3 | 75 | 0.4% | 0.88 [0.18, 4.24] |
| Kuisma et al (2006) | 6 | 16 | 4 | 16 | 0.0% | 1.50 [0.52, 4.32] |
| Kuisma et al (2006) | 4 | 16 | 6 | 16 | 0.9% | 0.67 [0.23, 1.92] |
| Mazdeh et al (2015) | 5 | 26 | 3 | 25 | 0.6% | 1.60 [0.43, 6.01] |
| NCT00414726 | 17 | 43 | 7 | 42 | 0.0% | 2.37 [1.10, 5.13] |
| Padma et al (2010) | 0 | 20 | 2 | 20 | 0.1% | 0.20 [0.01, 3.92] |
| Ranchord et al (2012), OPTIMISE | 1 | 72 | 4 | 76 | 0.2% | 0.26 [0.03, 2.31] |
| Ranchord et al (2012), OPTIMISE | 3 | 72 | 2 | 76 | 0.0% | 1.58 [0.27, 9.20] |
| Rawles et al (1976) | 9 | 105 | 3 | 95 | 0.7% | 2.71 [0.76, 9.73] |
| Roffe et al (2010) | 2 | 29 | 3 | 30 | 0.4% | 0.69 [0.12, 3.83] |
| Roffe et al (2017), SO2S | 493 | 5128 | 246 | 2549 | 50.2% | 1.00 [0.86, 1.15] |
| Rønning et al (1999) | 91 | 292 | 70 | 258 | 15.4% | 1.15 [0.88, 1.49] |
| Shi et al (2017) | 0 | 9 | 0 | 9 | | Not estimable |
| Singhal et al (2005) | 2 | 9 | 1 | 7 | 0.2% | 1.56 [0.17, 13.87] |
| Stub et al (2015) | 8 | 218 | 13 | 223 | 1.4% | 0.63 [0.27, 1.49] |
| Ukholkina et al (2005) | 1 | 58 | 0 | 79 | 0.1% | 4.07 [0.17, 98.10] |
| Wilson et al (1997) | 1 | 25 | 0 | 25 | 0.0% | 3.00 [0.13, 70.30] |
| Wilson et al (1997) | 0 | 25 | 1 | 25 | 0.1% | 0.33 [0.01, 7.81] |
| Young et al (2014), HOT OR NOT | 5 | 9 | 4 | 8 | 1.3% | 1.11 [0.45, 2.75] |
| **Total (95% CI)** | | **9538** | | **6943** | **100.0%** | **1.01 [0.92, 1.12]** |
| Total events | 812 | | 548 | | | |

Heterogeneity: $Tau^2 = 0.00$; $Chi^2 = 9.81$, df = 15 (P = 0.83); $I^2 = 0\%$
Test for overall effect: Z = 0.28 (P = 0.78)

| | | | | | | |
|---|---|---|---|---|---|---|
| **Total (95% CI)** | | **9538** | | **6943** | **100.0%** | **1.03 [0.93, 1.14]** |
| Total events | 817 | | 543 | | | |

Heterogeneity: $Tau^2 = 0.00$; $Chi^2 = 8.38$, df = 15 (P = 0.91); $I^2 = 0\%$
Test for overall effect: Z = 0.55 (P = 0.58)

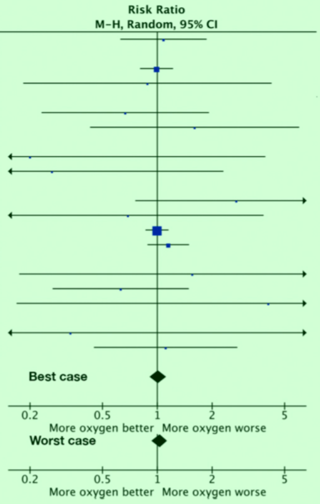


## IV: same as III but stroke only; N = 8,667

| Study or Subgroup | Liberal Events | Liberal Total | Conservative Events | Conservative Total | Weight | Risk Ratio M-H, Random, 95% CI |
|---|---|---|---|---|---|---|
| Ali et al (2014) | 22 | 126 | 21 | 130 | 5.1% | 1.08 [0.63, 1.86] |
| Chiu et al (2006) | 1 | 17 | 7 | 29 | 0.0% | 0.24 [0.03, 1.82] |
| Mazdeh et al (2015) | 5 | 26 | 3 | 25 | 0.9% | 1.60 [0.43, 6.01] |
| NCT00414726 | 17 | 43 | 7 | 42 | 0.0% | 2.37 [1.10, 5.13] |
| Padma et al (2010) | 0 | 20 | 2 | 20 | 0.2% | 0.20 [0.01, 3.92] |
| Roffe et al (2010) | 2 | 29 | 3 | 30 | 0.5% | 0.69 [0.12, 3.83] |
| Roffe et al (2017), SO2S | 493 | 5128 | 246 | 2549 | 71.2% | 1.00 [0.86, 1.15] |
| Rønning et al (1999) | 91 | 292 | 70 | 258 | 21.8% | 1.15 [0.88, 1.49] |
| Shi et al (2017) | 0 | 9 | 0 | 9 | | Not estimable |
| Singhal et al (2005) | 2 | 9 | 1 | 7 | 0.3% | 1.56 [0.17, 13.87] |
| **Total (95% CI)** | | **5639** | | **3028** | **100.0%** | **1.03 [0.91, 1.17]** |
| Total events | 615 | | 346 | | | |

Heterogeneity: $Tau^2 = 0.00$; $Chi^2 = 2.85$, df = 6 (P = 0.83); $I^2 = 0\%$
Test for overall effect: Z = 0.52 (P = 0.61)

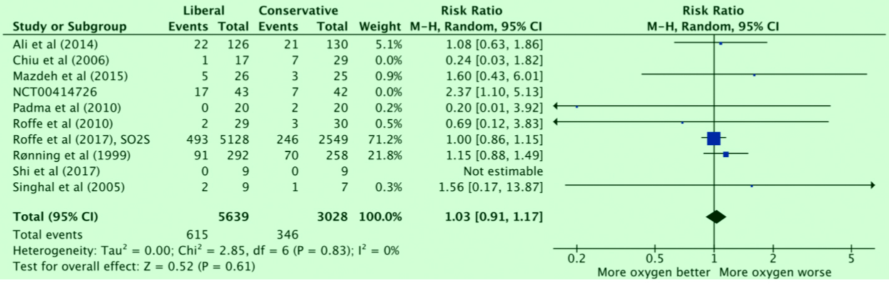

# Mortality at longest follow-up, FiO2 > 0.5

## I: as shown by Chu et al. Lancet. 2018 Apr 28;391(10131):1693-1705; N = 988

| Study or Subgroup | Liberal Events | Liberal Total | Conservative Events | Conservative Total | Weight | Risk Ratio M-H, Random, 95% CI |
|---|---|---|---|---|---|---|
| Khoshnood et al (2016), SOCCER | 3 | 85 | 3 | 75 | 6.5% | 0.88 [0.18, 4.24] |
| Kuisma et al (2006) | 4 | 14 | 4 | 14 | 11.5% | 1.00 [0.31, 3.23] |
| NCT00414726 | 17 | 43 | 7 | 42 | 24.9% | 2.37 [1.10, 5.13] |
| Padma et al (2010) | 0 | 20 | 2 | 20 | 1.9% | 0.20 [0.01, 3.92] |
| Shi et al (2017) | 0 | 9 | 0 | 9 | | Not estimable |
| Singhal et al (2005) | 2 | 9 | 2 | 7 | 5.6% | 0.78 [0.14, 4.23] |
| Stub et al (2015) | 14 | 312 | 18 | 312 | 31.0% | 0.78 [0.39, 1.54] |
| Young et al (2014), HOT OR NOT | 5 | 9 | 4 | 8 | 18.6% | 1.11 [0.45, 2.75] |
| **Total (95% CI)** | | **501** | | **487** | **100.0%** | **1.11 [0.74, 1.67]** |
| Total events | 45 | | 40 | | | |

Heterogeneity: Tau² = 0.02; Chi² = 6.36, df = 6 (P = 0.38); I² = 6%
Test for overall effect: Z = 0.50 (P = 0.61)

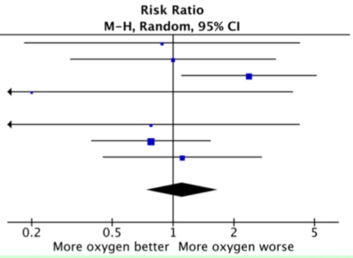


## II: same as I but numbers as reported in original publications; N = 809

| Study or Subgroup | Liberal Events | Liberal Total | Conservative Events | Conservative Total | Weight | Risk Ratio M-H, Random, 95% CI |
|---|---|---|---|---|---|---|
| Khoshnood et al (2016), SOCCER | 3 | 85 | 3 | 75 | 8.6% | 0.88 [0.18, 4.24] |
| Kuisma et al (2006) | 6 | 16 | 4 | 16 | 0.0% | 1.50 [0.52, 4.32] |
| Kuisma et al (2006) | 4 | 16 | 6 | 16 | 16.4% | 0.67 [0.23, 1.92] |
| NCT00414726 | 17 | 43 | 7 | 42 | 25.3% | 2.37 [1.10, 5.13] |
| Padma et al (2010) | 0 | 20 | 2 | 20 | 2.7% | 0.20 [0.01, 3.92] |
| Shi et al (2017) | 0 | 9 | 0 | 9 | | Not estimable |
| Singhal et al (2005) | 2 | 9 | 1 | 7 | 4.7% | 1.56 [0.17, 13.87] |
| Stub et al (2015) | 8 | 218 | 13 | 223 | 21.9% | 0.63 [0.27, 1.49] |
| Young et al (2014), HOT OR NOT | 5 | 9 | 4 | 8 | 20.4% | 1.11 [0.45, 2.75] |
| **Total (95% CI)** | | **409** | | **400** | **100.0%** | **1.04 [0.63, 1.71]** |
| Total events | 39 | | 36 | | | |

**Best case**

Heterogeneity: Tau² = 0.10; Chi² = 7.75, df = 6 (P = 0.26); I² = 23%
Test for overall effect: Z = 0.16 (P = 0.88)

| Study or Subgroup | Liberal Events | Liberal Total | Conservative Events | Conservative Total | Weight | Risk Ratio M-H, Random, 95% CI |
|---|---|---|---|---|---|---|
| **Total (95% CI)** | | **409** | | **400** | **100.0%** | **1.20 [0.76, 1.91]** |
| Total events | 41 | | 34 | | | |

**Worst case**

Heterogeneity: Tau² = 0.06; Chi² = 7.00, df = 6 (P = 0.32); I² = 14%
Test for overall effect: Z = 0.77 (P = 0.44)

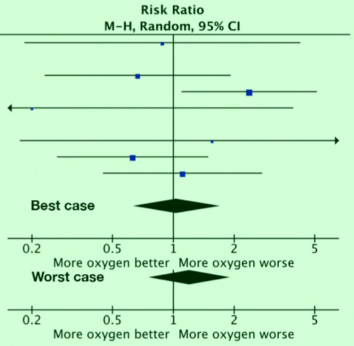


## III: same as II but stroke only; N = 159

| Study or Subgroup | Liberal Events | Liberal Total | Conservative Events | Conservative Total | Weight | Risk Ratio M-H, Random, 95% CI |
|---|---|---|---|---|---|---|
| NCT00414726 | 17 | 43 | 7 | 42 | 68.6% | 2.37 [1.10, 5.13] |
| Padma et al (2010) | 0 | 20 | 2 | 20 | 11.7% | 0.20 [0.01, 3.92] |
| Shi et al (2017) | 0 | 9 | 0 | 9 | | Not estimable |
| Singhal et al (2005) | 2 | 9 | 1 | 7 | 19.8% | 1.56 [0.17, 13.87] |
| **Total (95% CI)** | | **81** | | **78** | **100.0%** | **1.63 [0.56, 4.81]** |
| Total events | 19 | | 10 | | | |

Heterogeneity: Tau² = 0.29; Chi² = 2.62, df = 2 (P = 0.27); I² = 24%
Test for overall effect: Z = 0.89 (P = 0.37)

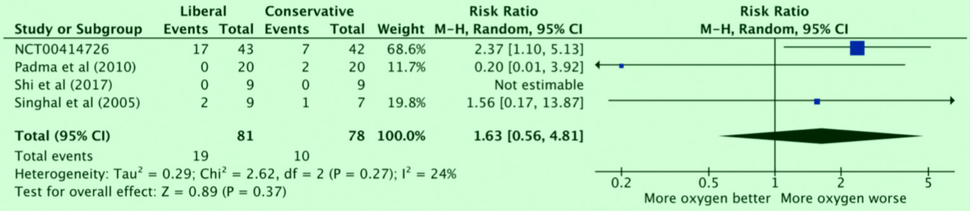

# Mortality at longest follow-up, FiO2 > 0.5, w/o NCT00414726

## I: as shown by Chu et al. Lancet. 2018 Apr 28;391(10131):1693-1705; N = 903

| Study or Subgroup | Liberal Events | Liberal Total | Conservative Events | Conservative Total | Weight | Risk Ratio M-H, Random, 95% CI |
|---|---|---|---|---|---|---|
| Khoshnood et al (2016), SOCCER | 3 | 85 | 3 | 75 | 8.2% | 0.88 [0.18, 4.24] |
| Kuisma et al (2006) | 4 | 14 | 4 | 14 | 14.7% | 1.00 [0.31, 3.23] |
| NCT00414726 | 17 | 43 | 7 | 42 | 0.0% | 2.37 [1.10, 5.13] |
| Padma et al (2010) | 0 | 20 | 2 | 20 | 2.3% | 0.20 [0.01, 3.92] |
| Shi et al (2017) | 0 | 9 | 0 | 9 | | Not estimable |
| Singhal et al (2005) | 2 | 9 | 2 | 7 | 7.0% | 0.78 [0.14, 4.23] |
| Stub et al (2015) | 14 | 312 | 18 | 312 | 43.4% | 0.78 [0.39, 1.54] |
| Young et al (2014), HOT OR NOT | 5 | 9 | 4 | 8 | 24.5% | 1.11 [0.45, 2.75] |
| **Total (95% CI)** | | **458** | | **445** | **100.0%** | **0.86 [0.55, 1.35]** |
| Total events | 28 | | 33 | | | |

Heterogeneity: $Tau^2 = 0.00$; $Chi^2 = 1.46$, df = 5 (P = 0.92); $I^2 = 0\%$
Test for overall effect: Z = 0.65 (P = 0.52)

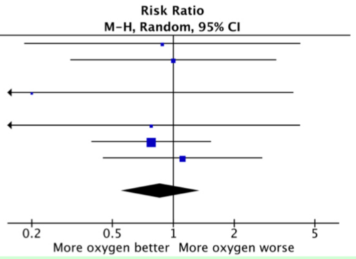


## II: same as I but numbers as reported in original publications; N = 724

| Study or Subgroup | Liberal Events | Liberal Total | Conservative Events | Conservative Total | Weight | Risk Ratio M-H, Random, 95% CI |
|---|---|---|---|---|---|---|
| Khoshnood et al (2016), SOCCER | 3 | 85 | 3 | 75 | 9.7% | 0.88 [0.18, 4.24] |
| Kuisma et al (2006) | 6 | 16 | 4 | 16 | 0.0% | 1.50 [0.52, 4.32] |
| Kuisma et al (2006) | 4 | 16 | 6 | 16 | 21.3% | 0.67 [0.23, 1.92] |
| NCT00414726 | 17 | 43 | 7 | 42 | 0.0% | 2.37 [1.10, 5.13] |
| Padma et al (2010) | 0 | 20 | 2 | 20 | 2.7% | 0.20 [0.01, 3.92] |
| Shi et al (2017) | 0 | 9 | 0 | 9 | | Not estimable |
| Singhal et al (2005) | 2 | 9 | 1 | 7 | 5.0% | 1.56 [0.17, 13.87] |
| Stub et al (2015) | 8 | 218 | 13 | 223 | 32.2% | 0.63 [0.27, 1.49] |
| Young et al (2014), HOT OR NOT | 5 | 9 | 4 | 8 | 29.1% | 1.11 [0.45, 2.75] |
| **Total (95% CI)** | | **366** | | **358** | **100.0%** | **0.79 [0.48, 1.28]** |
| Total events | 22 | | 29 | | | |

Heterogeneity: $Tau^2 = 0.00$; $Chi^2 = 2.21$, df = 5 (P = 0.82); $I^2 = 0\%$
Test for overall effect: Z = 0.96 (P = 0.34)

| | | | | | | |
|---|---|---|---|---|---|---|
| **Total (95% CI)** | | **366** | | **358** | **100.0%** | **0.94 [0.57, 1.53]** |
| Total events | 24 | | 27 | | | |

Heterogeneity: $Tau^2 = 0.00$; $Chi^2 = 3.09$, df = 5 (P = 0.69); $I^2 = 0\%$
Test for overall effect: Z = 0.26 (P = 0.79)

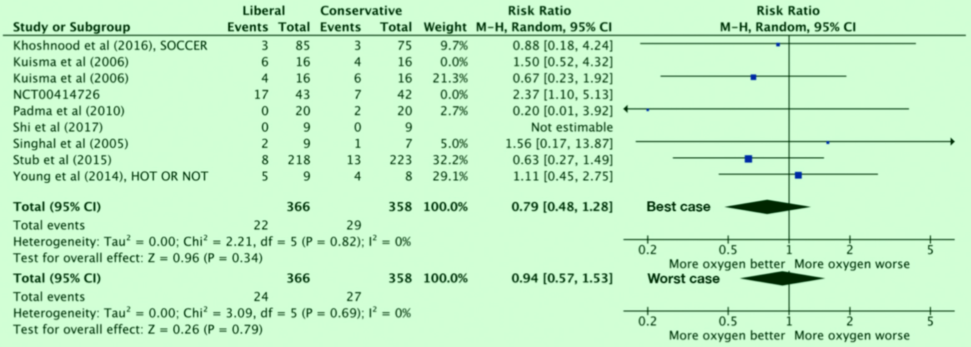


## III: same as II but stroke only; N = 74

| Study or Subgroup | Liberal Events | Liberal Total | Conservative Events | Conservative Total | Weight | Risk Ratio M-H, Random, 95% CI |
|---|---|---|---|---|---|---|
| NCT00414726 | 17 | 43 | 7 | 42 | 0.0% | 2.37 [1.10, 5.13] |
| Padma et al (2010) | 0 | 20 | 2 | 20 | 37.9% | 0.20 [0.01, 3.92] |
| Shi et al (2017) | 0 | 9 | 0 | 9 | | Not estimable |
| Singhal et al (2005) | 2 | 9 | 1 | 7 | 62.1% | 1.56 [0.17, 13.87] |
| **Total (95% CI)** | | **38** | | **36** | **100.0%** | **0.71 [0.10, 5.23]** |
| Total events | 2 | | 3 | | | |

Heterogeneity: $Tau^2 = 0.42$; $Chi^2 = 1.23$, df = 1 (P = 0.27); $I^2 = 19\%$
Test for overall effect: Z = 0.33 (P = 0.74)

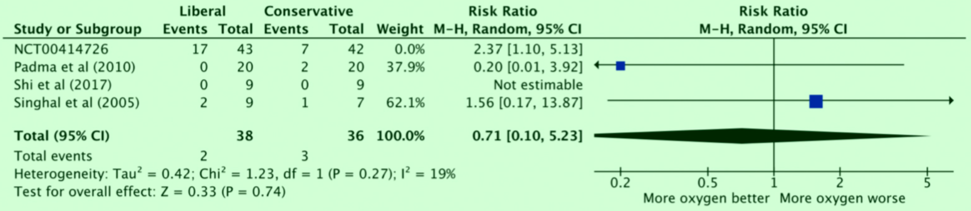

## Disability (modified Rankin Scale), any oxygen therapy

**I: excellent outcome (mRS 0-1), as shown by Chu et al. Lancet. 2018 Apr 28;391(10131):1693-1705; N = 5,523**

| Study or Subgroup | Liberal Events | Liberal Total | Conservative Events | Conservative Total | Weight | Odds Ratio (Non-event) M-H, Random, 95% CI |
|---|---|---|---|---|---|---|
| Ali et al (2014) | 39 | 126 | 42 | 130 | 21.8% | 1.06 [0.63, 1.80] |
| Mazdeh et al (2015) | 11 | 26 | 5 | 25 | 5.2% | 0.34 [0.10, 1.19] |
| NCT00414726 | 8 | 43 | 11 | 41 | 7.4% | 1.60 [0.57, 4.51] |
| Roffe et al (2017), SO2S | 1003 | 2567 | 1007 | 2549 | 64.2% | 1.02 [0.91, 1.14] |
| Singhal et al (2005) | 4 | 9 | 1 | 7 | 1.4% | 0.21 [0.02, 2.52] |
| **Total (95% CI)** | | **2771** | | **2752** | **100.0%** | **0.98 [0.73, 1.32]** |
| Total events | 1065 | | 1066 | | | |

Heterogeneity: Tau² = 0.03; Chi² = 5.26, df = 4 (P = 0.26); I² = 24%
Test for overall effect: Z = 0.12 (P = 0.91)

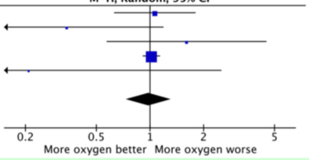


**II: same as I but numbers as reported in original publications; N = 8,084**

| Study or Subgroup | Liberal Events | Liberal Total | Conservative Events | Conservative Total | Weight | Odds Ratio (Non-event) M-H, Random, 95% CI |
|---|---|---|---|---|---|---|
| Ali et al (2014) | 39 | 126 | 42 | 130 | 21.4% | 1.06 [0.63, 1.80] |
| Mazdeh et al (2015) | 11 | 26 | 5 | 25 | 5.1% | 0.34 [0.10, 1.19] |
| NCT00414726 | 8 | 43 | 11 | 41 | 7.2% | 1.60 [0.57, 4.51] |
| Roffe et al (2017), SO2S | 2004 | 5128 | 1007 | 2549 | 64.9% | 1.02 [0.92, 1.12] |
| Singhal et al (2005) | 4 | 9 | 1 | 7 | 1.4% | 0.21 [0.02, 2.52] |
| **Total (95% CI)** | | **5332** | | **2752** | **100.0%** | **0.98 [0.73, 1.32]** |
| Total events | 2066 | | 1066 | | | |

Heterogeneity: Tau² = 0.03; Chi² = 5.26, df = 4 (P = 0.26); I² = 24%
Test for overall effect: Z = 0.11 (P = 0.91)

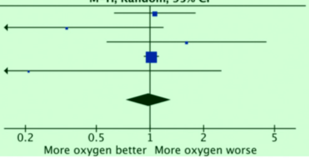


**III: good outcome or better (mRS 0-2), as shown by Chu et al.; N = 5,523**

| Study or Subgroup | Liberal Events | Liberal Total | Conservative Events | Conservative Total | Weight | Odds Ratio (Non-event) M-H, Random, 95% CI |
|---|---|---|---|---|---|---|
| Ali et al (2014) | 55 | 126 | 58 | 130 | 4.6% | 1.04 [0.63, 1.70] |
| Mazdeh et al (2015) | 12 | 26 | 10 | 25 | 0.9% | 0.78 [0.26, 2.36] |
| NCT00414726 | 14 | 43 | 19 | 41 | 1.4% | 1.79 [0.74, 4.34] |
| Roffe et al (2017), SO2S | 1325 | 2567 | 1337 | 2549 | 92.9% | 1.03 [0.93, 1.15] |
| Singhal et al (2005) | 4 | 9 | 1 | 7 | 0.2% | 0.21 [0.02, 2.52] |
| **Total (95% CI)** | | **2771** | | **2752** | **100.0%** | **1.04 [0.93, 1.15]** |
| Total events | 1410 | | 1425 | | | |

Heterogeneity: Tau² = 0.00; Chi² = 3.31, df = 4 (P = 0.51); I² = 0%
Test for overall effect: Z = 0.67 (P = 0.50)

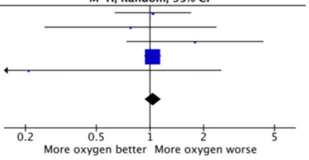


**IV: same as III but numbers as reported in original publications; N = 8,084**

| Study or Subgroup | Liberal Events | Liberal Total | Conservative Events | Conservative Total | Weight | Odds Ratio (Non-event) M-H, Random, 95% CI |
|---|---|---|---|---|---|---|
| Ali et al (2014) | 55 | 126 | 58 | 130 | 3.5% | 1.04 [0.63, 1.70] |
| Mazdeh et al (2015) | 12 | 26 | 10 | 25 | 0.7% | 0.78 [0.26, 2.36] |
| NCT00414726 | 14 | 43 | 19 | 41 | 1.1% | 1.79 [0.74, 4.34] |
| Roffe et al (2017), SO2S | 2641 | 5128 | 1337 | 2549 | 94.6% | 1.04 [0.94, 1.14] |
| Singhal et al (2005) | 4 | 9 | 1 | 7 | 0.1% | 0.21 [0.02, 2.52] |
| **Total (95% CI)** | | **5332** | | **2752** | **100.0%** | **1.04 [0.95, 1.14]** |
| Total events | 2726 | | 1425 | | | |

Heterogeneity: Tau² = 0.00; Chi² = 3.30, df = 4 (P = 0.51); I² = 0%
Test for overall effect: Z = 0.84 (P = 0.40)

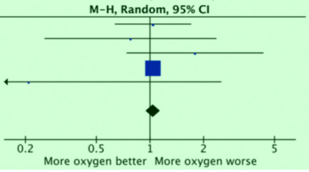


**V: moderate disability or better (mRS 0-3), as shown by Chu et al.; N = 5,523**

| Study or Subgroup | Liberal Events | Liberal Total | Conservative Events | Conservative Total | Weight | Odds Ratio (Non-event) M-H, Random, 95% CI |
|---|---|---|---|---|---|---|
| Ali et al (2014) | 76 | 126 | 85 | 130 | 27.9% | 1.24 [0.75, 2.07] |
| Mazdeh et al (2015) | 16 | 26 | 10 | 25 | 12.7% | 0.42 [0.14, 1.28] |
| NCT00414726 | 19 | 43 | 29 | 41 | 16.8% | 3.05 [1.24, 7.53] |
| Roffe et al (2017), SO2S | 1786 | 2567 | 1752 | 2549 | 39.1% | 0.96 [0.85, 1.08] |
| Singhal et al (2005) | 4 | 9 | 1 | 7 | 3.5% | 0.21 [0.02, 2.52] |
| **Total (95% CI)** | | **2771** | | **2752** | **100.0%** | **1.07 [0.66, 1.74]** |
| Total events | 1901 | | 1877 | | | |

Heterogeneity: Tau² = 0.15; Chi² = 10.73, df = 4 (P = 0.03); I² = 63%
Test for overall effect: Z = 0.27 (P = 0.79)

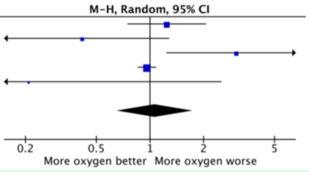


**VI: same as V but numbers as reported in original publications; N = 8,084**

| Study or Subgroup | Liberal Events | Liberal Total | Conservative Events | Conservative Total | Weight | Odds Ratio (Non-event) M-H, Random, 95% CI |
|---|---|---|---|---|---|---|
| Ali et al (2014) | 76 | 126 | 85 | 130 | 27.8% | 1.24 [0.75, 2.07] |
| Mazdeh et al (2015) | 16 | 26 | 10 | 25 | 12.4% | 0.42 [0.14, 1.28] |
| NCT00414726 | 19 | 43 | 29 | 41 | 16.4% | 3.05 [1.24, 7.53] |
| Roffe et al (2017), SO2S | 3524 | 5128 | 1752 | 2549 | 40.1% | 1.00 [0.90, 1.11] |
| Singhal et al (2005) | 4 | 9 | 1 | 7 | 3.3% | 0.21 [0.02, 2.52] |
| **Total (95% CI)** | | **5332** | | **2752** | **100.0%** | **1.09 [0.68, 1.75]** |
| Total events | 3639 | | 1877 | | | |

Heterogeneity: Tau² = 0.14; Chi² = 10.36, df = 4 (P = 0.03); I² = 61%
Test for overall effect: Z = 0.35 (P = 0.73)

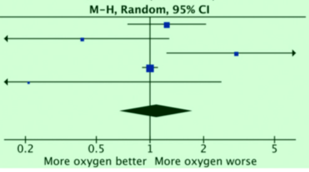


**VII: unable to walk unassisted or better (mRS 0-4), as shown by Chu et al.; N = 5,523**

| Study or Subgroup | Liberal Events | Liberal Total | Conservative Events | Conservative Total | Weight | Odds Ratio (Non-event) M-H, Random, 95% CI |
|---|---|---|---|---|---|---|
| Ali et al (2014) | 96 | 126 | 95 | 130 | 6.4% | 0.85 [0.48, 1.49] |
| Mazdeh et al (2015) | 19 | 26 | 17 | 25 | 1.4% | 0.78 [0.23, 2.62] |
| NCT00414726 | 26 | 43 | 32 | 41 | 2.2% | 2.32 [0.89, 6.07] |
| Roffe et al (2017), SO2S | 2162 | 2567 | 2147 | 2549 | 89.6% | 1.00 [0.86, 1.16] |
| Singhal et al (2005) | 6 | 9 | 4 | 7 | 0.5% | 0.67 [0.09, 5.13] |
| **Total (95% CI)** | | **2771** | | **2752** | **100.0%** | **1.00 [0.87, 1.16]** |
| Total events | 2309 | | 2295 | | | |

Heterogeneity: Tau² = 0.00; Chi² = 3.60, df = 4 (P = 0.46); I² = 0%
Test for overall effect: Z = 0.04 (P = 0.97)

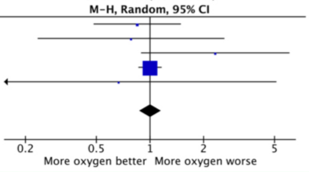


**VIII: same as VII but numbers as reported in original publications; N = 8,084**

| Study or Subgroup | Liberal Events | Liberal Total | Conservative Events | Conservative Total | Weight | Odds Ratio (Non-event) M-H, Random, 95% CI |
|---|---|---|---|---|---|---|
| Ali et al (2014) | 96 | 126 | 95 | 130 | 4.9% | 0.85 [0.48, 1.49] |
| Mazdeh et al (2015) | 19 | 26 | 17 | 25 | 1.1% | 0.78 [0.23, 2.62] |
| NCT00414726 | 26 | 43 | 32 | 41 | 1.7% | 2.32 [0.89, 6.07] |
| Roffe et al (2017), SO2S | 4319 | 5128 | 2147 | 2549 | 92.0% | 1.00 [0.88, 1.14] |
| Singhal et al (2005) | 6 | 9 | 4 | 7 | 0.4% | 0.67 [0.09, 5.13] |
| **Total (95% CI)** | | **5332** | | **2752** | **100.0%** | **1.00 [0.88, 1.14]** |
| Total events | 4466 | | 2295 | | | |

Heterogeneity: Tau² = 0.00; Chi² = 3.61, df = 4 (P = 0.46); I² = 0%
Test for overall effect: Z = 0.04 (P = 0.97)

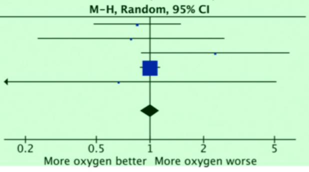

# Disability (mRS), any OT, w/o NCT00414726

## I: excellent outcome (mRS 0-1), as shown by Chu et al. Lancet. 2018 Apr 28;391(10131):1693-1705; N = 5,439

| Study or Subgroup | Liberal Events | Liberal Total | Conservative Events | Conservative Total | Weight | Odds Ratio (Non-event) M-H, Random, 95% CI |
|---|---|---|---|---|---|---|
| Ali et al (2014) | 39 | 126 | 42 | 130 | 26.8% | 1.06 [0.63, 1.80] |
| Mazdeh et al (2015) | 11 | 26 | 5 | 25 | 7.0% | 0.34 [0.10, 1.19] |
| NCT00414726 | 8 | 43 | 11 | 41 | 0.0% | 1.60 [0.57, 4.51] |
| Roffe et al (2017), SO2S | 1003 | 2567 | 1007 | 2549 | 64.2% | 1.02 [0.91, 1.14] |
| Singhal et al (2005) | 4 | 9 | 1 | 7 | 1.9% | 0.21 [0.02, 2.52] |
| **Total (95% CI)** | | **2728** | | **2711** | **100.0%** | **0.93 [0.65, 1.31]** |
| Total events | 1057 | | 1055 | | | |

Heterogeneity: Tau² = 0.05; Chi² = 4.49, df = 3 (P = 0.21); I² = 33%
Test for overall effect: Z = 0.43 (P = 0.67)

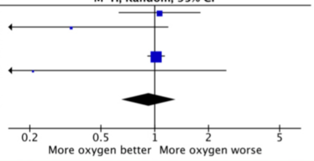


## II: same as I but numbers as reported in original publications; N = 8,000

| Study or Subgroup | Liberal Events | Liberal Total | Conservative Events | Conservative Total | Weight | Odds Ratio (Non-event) M-H, Random, 95% CI |
|---|---|---|---|---|---|---|
| Ali et al (2014) | 39 | 126 | 42 | 130 | 26.5% | 1.06 [0.63, 1.80] |
| Mazdeh et al (2015) | 11 | 26 | 5 | 25 | 6.9% | 0.34 [0.10, 1.19] |
| NCT00414726 | 8 | 43 | 11 | 41 | 0.0% | 1.60 [0.57, 4.51] |
| Roffe et al (2017), SO2S | 2004 | 5128 | 1007 | 2549 | 64.7% | 1.02 [0.92, 1.12] |
| Singhal et al (2005) | 4 | 9 | 1 | 7 | 1.9% | 0.21 [0.02, 2.52] |
| **Total (95% CI)** | | **5289** | | **2711** | **100.0%** | **0.93 [0.66, 1.31]** |
| Total events | 2058 | | 1055 | | | |

Heterogeneity: Tau² = 0.05; Chi² = 4.50, df = 3 (P = 0.21); I² = 33%
Test for overall effect: Z = 0.43 (P = 0.67)

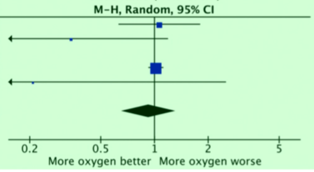


## III: good outcome or better (mRS 0-2), as shown by Chu et al.; N = 5,439

| Study or Subgroup | Liberal Events | Liberal Total | Conservative Events | Conservative Total | Weight | Odds Ratio (Non-event) M-H, Random, 95% CI |
|---|---|---|---|---|---|---|
| Ali et al (2014) | 55 | 126 | 58 | 130 | 4.7% | 1.04 [0.63, 1.70] |
| Mazdeh et al (2015) | 12 | 26 | 10 | 25 | 0.9% | 0.78 [0.26, 2.36] |
| NCT00414726 | 14 | 43 | 19 | 41 | 0.0% | 1.79 [0.74, 4.34] |
| Roffe et al (2017), SO2S | 1325 | 2567 | 1337 | 2549 | 94.2% | 1.03 [0.93, 1.15] |
| Singhal et al (2005) | 4 | 9 | 1 | 7 | 0.2% | 0.21 [0.02, 2.52] |
| **Total (95% CI)** | | **2728** | | **2711** | **100.0%** | **1.03 [0.92, 1.14]** |
| Total events | 1396 | | 1406 | | | |

Heterogeneity: Tau² = 0.00; Chi² = 1.83, df = 3 (P = 0.61); I² = 0%
Test for overall effect: Z = 0.52 (P = 0.60)

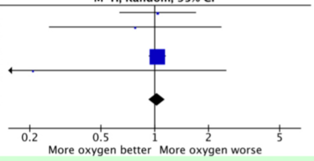


## IV: same as III but numbers as reported in original publications; N = 8,000

| Study or Subgroup | Liberal Events | Liberal Total | Conservative Events | Conservative Total | Weight | Odds Ratio (Non-event) M-H, Random, 95% CI |
|---|---|---|---|---|---|---|
| Ali et al (2014) | 55 | 126 | 58 | 130 | 3.5% | 1.04 [0.63, 1.70] |
| Mazdeh et al (2015) | 12 | 26 | 10 | 25 | 0.7% | 0.78 [0.26, 2.36] |
| NCT00414726 | 14 | 43 | 19 | 41 | 0.0% | 1.79 [0.74, 4.34] |
| Roffe et al (2017), SO2S | 2641 | 5128 | 1337 | 2549 | 95.6% | 1.04 [0.94, 1.14] |
| Singhal et al (2005) | 4 | 9 | 1 | 7 | 0.1% | 0.21 [0.02, 2.52] |
| **Total (95% CI)** | | **5289** | | **2711** | **100.0%** | **1.03 [0.94, 1.14]** |
| Total events | 2712 | | 1406 | | | |

Heterogeneity: Tau² = 0.00; Chi² = 1.85, df = 3 (P = 0.60); I² = 0%
Test for overall effect: Z = 0.71 (P = 0.48)

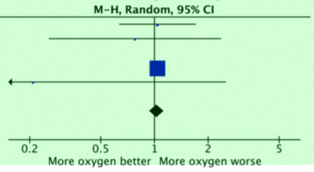


## V: moderate disability or better (mRS 0-3), as shown by Chu et al.; N = 5,439

| Study or Subgroup | Liberal Events | Liberal Total | Conservative Events | Conservative Total | Weight | Odds Ratio (Non-event) M-H, Random, 95% CI |
|---|---|---|---|---|---|---|
| Ali et al (2014) | 76 | 126 | 85 | 130 | 27.1% | 1.24 [0.75, 2.07] |
| Mazdeh et al (2015) | 16 | 26 | 10 | 25 | 8.1% | 0.42 [0.14, 1.28] |
| NCT00414726 | 19 | 43 | 29 | 41 | 0.0% | 3.05 [1.24, 7.53] |
| Roffe et al (2017), SO2S | 1786 | 2567 | 1752 | 2549 | 63.0% | 0.96 [0.85, 1.08] |
| Singhal et al (2005) | 4 | 9 | 1 | 7 | 1.8% | 0.21 [0.02, 2.52] |
| **Total (95% CI)** | | **2728** | | **2711** | **100.0%** | **0.94 [0.67, 1.32]** |
| Total events | 1882 | | 1848 | | | |

Heterogeneity: Tau² = 0.04; Chi² = 4.55, df = 3 (P = 0.21); I² = 34%
Test for overall effect: Z = 0.38 (P = 0.71)

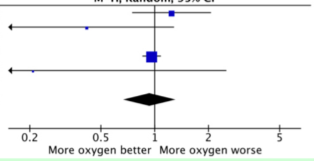


## VI: same as V but numbers as reported in original publications; N = 8,000

| Study or Subgroup | Liberal Events | Liberal Total | Conservative Events | Conservative Total | Weight | Odds Ratio (Non-event) M-H, Random, 95% CI |
|---|---|---|---|---|---|---|
| Ali et al (2014) | 76 | 126 | 85 | 130 | 26.6% | 1.24 [0.75, 2.07] |
| Mazdeh et al (2015) | 16 | 26 | 10 | 25 | 7.9% | 0.42 [0.14, 1.28] |
| NCT00414726 | 19 | 43 | 29 | 41 | 0.0% | 3.05 [1.24, 7.53] |
| Roffe et al (2017), SO2S | 3524 | 5128 | 1752 | 2549 | 63.7% | 1.00 [0.90, 1.11] |
| Singhal et al (2005) | 4 | 9 | 1 | 7 | 1.8% | 0.21 [0.02, 2.52] |
| **Total (95% CI)** | | **5289** | | **2711** | **100.0%** | **0.96 [0.69, 1.35]** |
| Total events | 3620 | | 1848 | | | |

Heterogeneity: Tau² = 0.04; Chi² = 4.55, df = 3 (P = 0.21); I² = 34%
Test for overall effect: Z = 0.23 (P = 0.82)

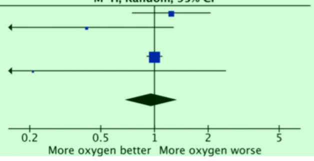


## VII: unable to walk unassisted or better (mRS 0-4), as shown by Chu et al.; N = 5,439

| Study or Subgroup | Liberal Events | Liberal Total | Conservative Events | Conservative Total | Weight | Odds Ratio (Non-event) M-H, Random, 95% CI |
|---|---|---|---|---|---|---|
| Ali et al (2014) | 96 | 126 | 95 | 130 | 6.5% | 0.85 [0.48, 1.49] |
| Mazdeh et al (2015) | 19 | 26 | 17 | 25 | 1.4% | 0.78 [0.23, 2.62] |
| NCT00414726 | 26 | 43 | 32 | 41 | 0.0% | 2.32 [0.89, 6.07] |
| Roffe et al (2017), SO2S | 2162 | 2567 | 2147 | 2549 | 91.6% | 1.00 [0.86, 1.16] |
| Singhal et al (2005) | 6 | 9 | 4 | 7 | 0.5% | 0.67 [0.09, 5.13] |
| **Total (95% CI)** | | **2728** | | **2711** | **100.0%** | **0.98 [0.85, 1.14]** |
| Total events | 2283 | | 2263 | | | |

Heterogeneity: Tau² = 0.00; Chi² = 0.59, df = 3 (P = 0.90); I² = 0%
Test for overall effect: Z = 0.21 (P = 0.83)

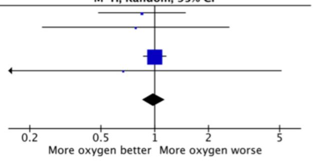


## VIII: same as VII but numbers as reported in original publications; N = 8,000

| Study or Subgroup | Liberal Events | Liberal Total | Conservative Events | Conservative Total | Weight | Odds Ratio (Non-event) M-H, Random, 95% CI |
|---|---|---|---|---|---|---|
| Ali et al (2014) | 96 | 126 | 95 | 130 | 5.0% | 0.85 [0.48, 1.49] |
| Mazdeh et al (2015) | 19 | 26 | 17 | 25 | 1.1% | 0.78 [0.23, 2.62] |
| NCT00414726 | 26 | 43 | 32 | 41 | 0.0% | 2.32 [0.89, 6.07] |
| Roffe et al (2017), SO2S | 4319 | 5128 | 2147 | 2549 | 93.5% | 1.00 [0.88, 1.14] |
| Singhal et al (2005) | 6 | 9 | 4 | 7 | 0.4% | 0.67 [0.09, 5.13] |
| **Total (95% CI)** | | **5289** | | **2711** | **100.0%** | **0.99 [0.87, 1.12]** |
| Total events | 4440 | | 2263 | | | |

Heterogeneity: Tau² = 0.00; Chi² = 0.60, df = 3 (P = 0.90); I² = 0%
Test for overall effect: Z = 0.19 (P = 0.85)

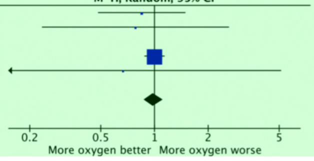

## Disability (modified Rankin Scale), FiO2 > 0.5

### I: excellent outcome (mRS 0-1), as shown by Chu et al. Lancet. 2018 Apr 28;391(10131):1693-1705; N = 100

| Study or Subgroup | Liberal Events | Liberal Total | Conservative Events | Conservative Total | Weight | Odds Ratio (Non-event) M-H, Random, 95% CI |
|---|---|---|---|---|---|---|
| NCT00414726 | 8 | 43 | 11 | 41 | 66.0% | 1.60 [0.57, 4.51] |
| Singhal et al (2005) | 4 | 9 | 1 | 7 | 34.0% | 0.21 [0.02, 2.52] |
| **Total (95% CI)** | | **52** | | **48** | **100.0%** | **0.80 [0.12, 5.36]** |
| Total events | 12 | | 12 | | | |

Heterogeneity: Tau² = 1.15; Chi² = 2.21, df = 1 (P = 0.14); I² = 55%
Test for overall effect: Z = 0.23 (P = 0.82)

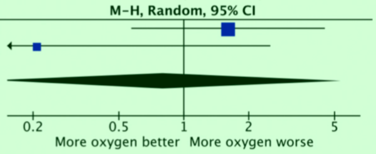


### II: good outcome or better (mRS 0-2), as shown by Chu et al.; N = 100

| Study or Subgroup | Liberal Events | Liberal Total | Conservative Events | Conservative Total | Weight | Odds Ratio (Non-event) M-H, Random, 95% CI |
|---|---|---|---|---|---|---|
| NCT00414726 | 14 | 43 | 19 | 41 | 65.2% | 1.79 [0.74, 4.34] |
| Singhal et al (2005) | 4 | 9 | 1 | 7 | 34.8% | 0.21 [0.02, 2.52] |
| **Total (95% CI)** | | **52** | | **48** | **100.0%** | **0.85 [0.11, 6.34]** |
| Total events | 18 | | 20 | | | |

Heterogeneity: Tau² = 1.42; Chi² = 2.56, df = 1 (P = 0.11); I² = 61%
Test for overall effect: Z = 0.16 (P = 0.87)

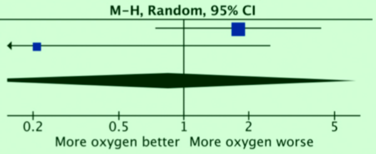


### III: moderate disability or better (mRS 0-3), as shown by Chu et al.; N = 100

| Study or Subgroup | Liberal Events | Liberal Total | Conservative Events | Conservative Total | Weight | Odds Ratio (Non-event) M-H, Random, 95% CI |
|---|---|---|---|---|---|---|
| NCT00414726 | 19 | 43 | 29 | 41 | 59.6% | 3.05 [1.24, 7.53] |
| Singhal et al (2005) | 4 | 9 | 1 | 7 | 40.4% | 0.21 [0.02, 2.52] |
| **Total (95% CI)** | | **52** | | **48** | **100.0%** | **1.03 [0.08, 13.85]** |
| Total events | 23 | | 30 | | | |

Heterogeneity: Tau² = 2.73; Chi² = 3.99, df = 1 (P = 0.05); I² = 75%
Test for overall effect: Z = 0.02 (P = 0.98)

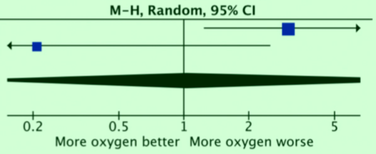


### IV: unable to walk unassisted or better (mRS 0-4), as shown by Chu et al.; N = 100

| Study or Subgroup | Liberal Events | Liberal Total | Conservative Events | Conservative Total | Weight | Odds Ratio (Non-event) M-H, Random, 95% CI |
|---|---|---|---|---|---|---|
| NCT00414726 | 26 | 43 | 32 | 41 | 77.0% | 2.32 [0.89, 6.07] |
| Singhal et al (2005) | 6 | 9 | 4 | 7 | 23.0% | 0.67 [0.09, 5.13] |
| **Total (95% CI)** | | **52** | | **48** | **100.0%** | **1.75 [0.62, 4.89]** |
| Total events | 32 | | 36 | | | |

Heterogeneity: Tau² = 0.12; Chi² = 1.18, df = 1 (P = 0.28); I² = 15%
Test for overall effect: Z = 1.06 (P = 0.29)

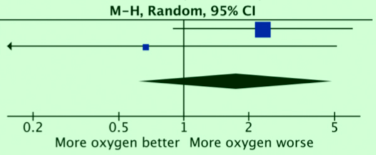

# Disability (mRS), FiO2 > 0.5, w/o NCT00414726

## I: excellent outcome (mRS 0-1), as shown by Chu et al. Lancet. 2018 Apr 28;391(10131):1693-1705; N = 16

| | Liberal | | Conservative | | | Odds Ratio (Non-event) |
|---|---|---|---|---|---|---|
| **Study or Subgroup** | **Events** | **Total** | **Events** | **Total** | **Weight** | **M-H, Random, 95% CI** |
| NCT00414726 | 8 | 43 | 11 | 41 | 0.0% | 1.60 [0.57, 4.51] |
| Singhal et al (2005) | 4 | 9 | 1 | 7 | 100.0% | 0.21 [0.02, 2.52] |
| **Total (95% CI)** | | **9** | | **7** | **100.0%** | **0.21 [0.02, 2.52]** |
| Total events | 4 | | 1 | | | |
| Heterogeneity: Not applicable | | | | | | |
| Test for overall effect: Z = 1.23 (P = 0.22) | | | | | | |

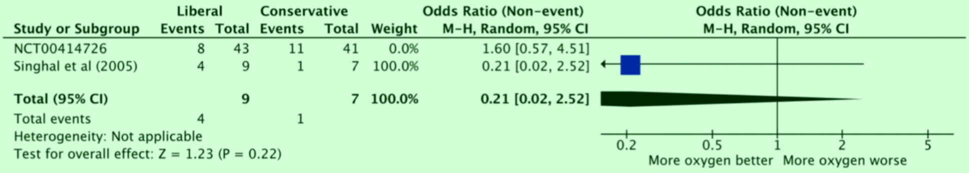


## II: good outcome or better (mRS 0-2), as shown by Chu et al.; N = 16

| | Liberal | | Conservative | | | Odds Ratio (Non-event) |
|---|---|---|---|---|---|---|
| **Study or Subgroup** | **Events** | **Total** | **Events** | **Total** | **Weight** | **M-H, Random, 95% CI** |
| NCT00414726 | 14 | 43 | 19 | 41 | 0.0% | 1.79 [0.74, 4.34] |
| Singhal et al (2005) | 4 | 9 | 1 | 7 | 100.0% | 0.21 [0.02, 2.52] |
| **Total (95% CI)** | | **9** | | **7** | **100.0%** | **0.21 [0.02, 2.52]** |
| Total events | 4 | | 1 | | | |
| Heterogeneity: Not applicable | | | | | | |
| Test for overall effect: Z = 1.23 (P = 0.22) | | | | | | |

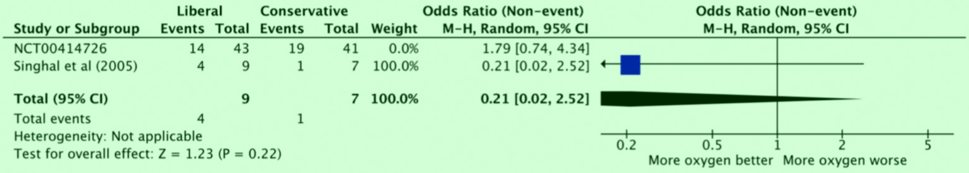


## III: moderate disability or better (mRS 0-3), as shown by Chu et al.; N = 16

| | Liberal | | Conservative | | | Odds Ratio (Non-event) |
|---|---|---|---|---|---|---|
| **Study or Subgroup** | **Events** | **Total** | **Events** | **Total** | **Weight** | **M-H, Random, 95% CI** |
| NCT00414726 | 19 | 43 | 29 | 41 | 0.0% | 3.05 [1.24, 7.53] |
| Singhal et al (2005) | 4 | 9 | 1 | 7 | 100.0% | 0.21 [0.02, 2.52] |
| **Total (95% CI)** | | **9** | | **7** | **100.0%** | **0.21 [0.02, 2.52]** |
| Total events | 4 | | 1 | | | |
| Heterogeneity: Not applicable | | | | | | |
| Test for overall effect: Z = 1.23 (P = 0.22) | | | | | | |

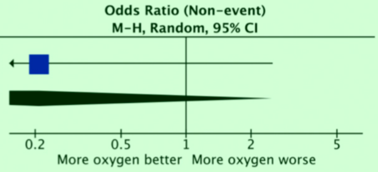


## IV: unable to walk unassisted or better (mRS 0-4), as shown by Chu et al.; N = 16

| | Liberal | | Conservative | | | Odds Ratio (Non-event) |
|---|---|---|---|---|---|---|
| **Study or Subgroup** | **Events** | **Total** | **Events** | **Total** | **Weight** | **M-H, Random, 95% CI** |
| NCT00414726 | 26 | 43 | 32 | 41 | 0.0% | 2.32 [0.89, 6.07] |
| Singhal et al (2005) | 6 | 9 | 4 | 7 | 100.0% | 0.67 [0.09, 5.13] |
| **Total (95% CI)** | | **9** | | **7** | **100.0%** | **0.67 [0.09, 5.13]** |
| Total events | 6 | | 4 | | | |
| Heterogeneity: Not applicable | | | | | | |
| Test for overall effect: Z = 0.39 (P = 0.70) | | | | | | |

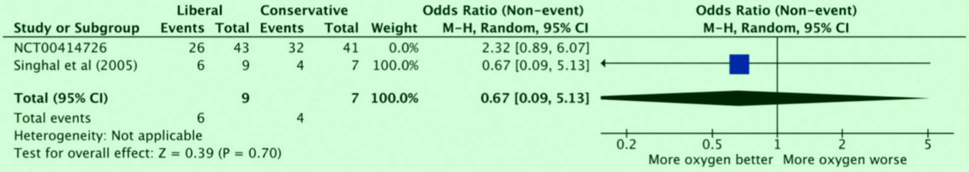

# Tertiary outcomes, any oxygen therapy

## I: hospital-acquired infection, as shown by Chu et al. Lancet. 2018 Apr 28;391(10131):1693-1705; N = 6,334

| Study or Subgroup | Liberal Events | Liberal Total | Conservative Events | Conservative Total | Weight | Risk Ratio M-H, Random, 95% CI |
|---|---|---|---|---|---|---|
| Ali et al (2014) | 27 | 148 | 22 | 141 | 5.5% | 1.17 [0.70, 1.95] |
| NCT00414726 | 18 | 43 | 14 | 42 | 4.7% | 1.26 [0.72, 2.19] |
| Roffe et al (2017), SO2S | 400 | 2668 | 403 | 2668 | 88.8% | 0.99 [0.87, 1.13] |
| Stub et al (2015) | 7 | 312 | 4 | 312 | 1.0% | 1.75 [0.52, 5.92] |
| **Total (95% CI)** | | **3171** | | **3163** | **100.0%** | **1.02 [0.90, 1.15]** |
| Total events | 452 | | 443 | | | |

Heterogeneity: $Tau^2 = 0.00$; $Chi^2 = 1.74$, df = 3 (P = 0.63); $I^2 = 0\%$
Test for overall effect: Z = 0.29 (P = 0.77)

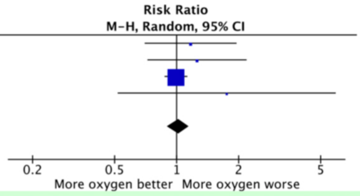


## II: same as I but numbers as reported in original publications; N = 8,731

| Study or Subgroup | Liberal Events | Liberal Total | Conservative Events | Conservative Total | Weight | Risk Ratio M-H, Random, 95% CI |
|---|---|---|---|---|---|---|
| Ali et al (2014) | 27 | 148 | 22 | 141 | 4.3% | 1.17 [0.70, 1.95] |
| Chiu et al (2006) | 4 | 17 | 15 | 29 | 0.0% | 0.45 [0.18, 1.15] |
| NCT00414726 | 18 | 43 | 14 | 42 | 3.7% | 1.26 [0.72, 2.19] |
| Roffe et al (2017), SO2S | 793 | 5267 | 403 | 2649 | 92.0% | 0.99 [0.89, 1.11] |
| Stub et al (2015) | 0 | 218 | 1 | 223 | 0.1% | 0.34 [0.01, 8.32] |
| **Total (95% CI)** | | **5676** | | **3055** | **100.0%** | **1.00 [0.90, 1.12]** |
| Total events | 838 | | 440 | | | |

Heterogeneity: $Tau^2 = 0.00$; $Chi^2 = 1.47$, df = 3 (P = 0.69); $I^2 = 0\%$
Test for overall effect: Z = 0.08 (P = 0.94)

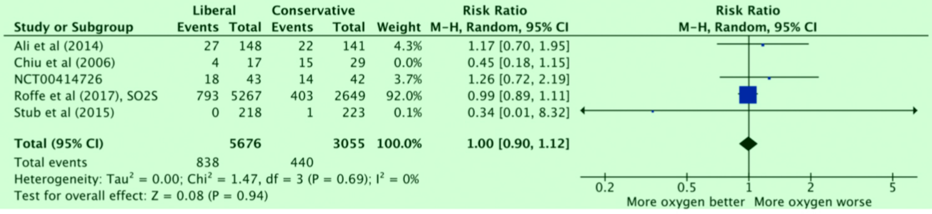


## III: hospital-acquired pneumonia, as shown by Chu et al.; N = 624

| Study or Subgroup | Liberal Events | Liberal Total | Conservative Events | Conservative Total | Weight | Risk Ratio M-H, Random, 95% CI |
|---|---|---|---|---|---|---|
| Stub et al (2015) | 2 | 312 | 1 | 312 | 100.0% | 2.00 [0.18, 21.94] |
| **Total (95% CI)** | | **312** | | **312** | **100.0%** | **2.00 [0.18, 21.94]** |
| Total events | 2 | | 1 | | | |

Heterogeneity: Not applicable
Test for overall effect: Z = 0.57 (P = 0.57)

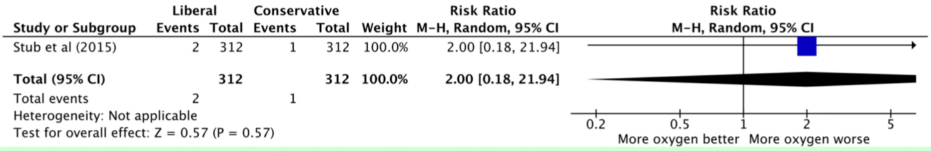


## IV: same as III but numbers as reported in original publications; N = 0

| Study or Subgroup | Liberal Events | Liberal Total | Conservative Events | Conservative Total | Weight | Risk Ratio M-H, Random, 95% CI |
|---|---|---|---|---|---|---|
| Chiu et al (2006) | 1 | 17 | 6 | 29 | 0.0% | 0.28 [0.04, 2.17] |
| Stub et al (2015) | not reported | | not reported | | | Not estimable |
| **Total (95% CI)** | | **0** | | **0** | | **Not estimable** |
| Total events | 0 | | 0 | | | |

Heterogeneity: Not applicable
Test for overall effect: Not applicable

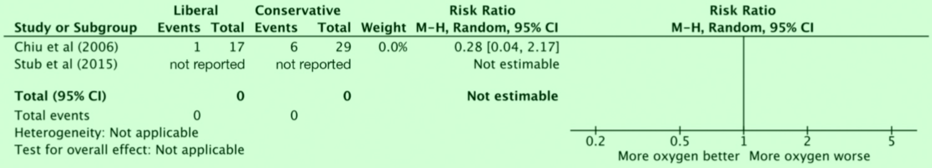


## V: respiratory serious adverse events; N = 85

| Study or Subgroup | Liberal Events | Liberal Total | Conservative Events | Conservative Total | Weight | Risk Ratio M-H, Random, 95% CI |
|---|---|---|---|---|---|---|
| Chiu et al (2006) | 3 | 17 | 8 | 29 | 0.0% | 0.64 [0.20, 2.09] |
| NCT00414726 | 3 | 43 | 2 | 42 | 100.0% | 1.47 [0.26, 8.33] |
| **Total (95% CI)** | | **43** | | **42** | **100.0%** | **1.47 [0.26, 8.33]** |
| Total events | 3 | | 2 | | | |

Heterogeneity: Not applicable
Test for overall effect: Z = 0.43 (P = 0.67)

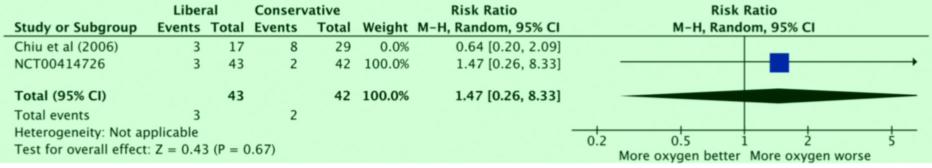


## VI: length of hospital stay, as shown by Chu et al.; N = 1,250

| Study or Subgroup | Liberal Mean | Liberal SD | Liberal Total | Conservative Mean | Conservative SD | Conservative Total | Weight | Mean Difference IV, Random, 95% CI |
|---|---|---|---|---|---|---|---|---|
| NCT00414726 | 8 | 4 | 43 | 7 | 5 | 42 | 4.4% | 1.00 [−0.93, 2.93] |
| Rawles et al (1976) | 16.2 | 5.4 | 80 | 14.9 | 5.3 | 77 | 5.7% | 1.30 [−0.37, 2.97] |
| Rønning et al (1999) | 8.6 | 6 | 292 | 8.7 | 7 | 258 | 12.4% | −0.10 [−1.20, 1.00] |
| Stub et al (2015) | 4 | 0.7 | 218 | 4 | 1.5 | 223 | 77.4% | 0.00 [−0.22, 0.22] |
| Young et al (2014), HOT OR NOT | 6.5 | 5.8 | 9 | 12.4 | 14.5 | 8 | 0.1% | −5.90 [−16.64, 4.84] |
| **Total (95% CI)** | | | **642** | | | **608** | **100.0%** | **0.10 [−0.32, 0.51]** |

Heterogeneity: $Tau^2 = 0.04$; $Chi^2 = 4.48$, df = 4 (P = 0.34); $I^2 = 11\%$
Test for overall effect: Z = 0.46 (P = 0.64)

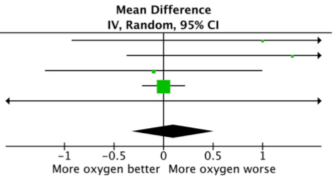


## VII: same as VI but numbers as reported in original publications; N = 1,293

| Study or Subgroup | Liberal Mean | Liberal SD | Liberal Total | Conservative Mean | Conservative SD | Conservative Total | Weight | Mean Difference IV, Random, 95% CI |
|---|---|---|---|---|---|---|---|---|
| NCT00414726 | 8 | 4 | 43 | 7 | 5 | 42 | 1.2% | 1.00 [−0.93, 2.93] |
| Rawles et al (1976) | 15 | 5.7 | 105 | 14 | 5.6 | 95 | 1.8% | 1.00 [−0.57, 2.57] |
| Rønning et al (1999) | 8.6 | 6 | 292 | 8.7 | 7 | 258 | 3.7% | −0.10 [−1.20, 1.00] |
| Stub et al (2015) | 4.4 | 0.7 | 218 | 4 | 1.5 | 223 | 93.3% | 0.40 [0.18, 0.62] |
| Young et al (2014), HOT OR NOT | 5.1 | 5 | 9 | 11.7 | 23.5 | 8 | 0.0% | −6.60 [−23.21, 10.01] |
| **Total (95% CI)** | | | **667** | | | **626** | **100.0%** | **0.40 [0.19, 0.61]** |

Heterogeneity: $Tau^2 = 0.00$; $Chi^2 = 2.42$, df = 4 (P = 0.66); $I^2 = 0\%$
Test for overall effect: Z = 3.71 (P = 0.0002)

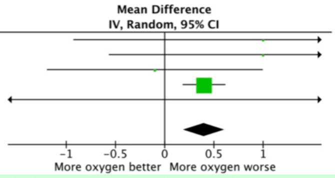


## VIII: length of hospital stay, all RCT; N = 7,922

| Study or Subgroup | Liberal Mean | Liberal SD | Liberal Total | Conservative Mean | Conservative SD | Conservative Total | Weight | Mean Difference IV, Random, 95% CI |
|---|---|---|---|---|---|---|---|---|
| Chiu et al (2006) | 17.2 | 15.3 | 16 | 21.1 | 16.2 | 22 | 0.0% | −3.90 [−14.00, 6.20] |
| Hofmann et al (2017), DETO2X-AMI | 3.3 | 9.6 | 3311 | 3.4 | 13.4 | 3318 | 13.8% | −0.10 [−0.66, 0.46] |
| NCT00414726 | 8 | 4 | 43 | 7 | 5 | 42 | 1.2% | 1.00 [−0.93, 2.93] |
| Rawles et al (1976) | 15 | 5.7 | 105 | 14 | 5.6 | 95 | 1.8% | 1.00 [−0.57, 2.57] |
| Rønning et al (1999) | 8.6 | 6 | 292 | 8.7 | 7 | 258 | 3.7% | −0.10 [−1.20, 1.00] |
| Stub et al (2015) | 4.4 | 0.7 | 218 | 4 | 1.5 | 223 | 79.4% | 0.40 [0.18, 0.62] |
| Young et al (2014), HOT OR NOT | 5.1 | 5 | 9 | 11.7 | 23.5 | 8 | 0.0% | −6.60 [−23.21, 10.01] |
| **Total (95% CI)** | | | **3978** | | | **3944** | **100.0%** | **0.33 [0.12, 0.54]** |

Heterogeneity: $Tau^2 = 0.00$; $Chi^2 = 5.07$, df = 5 (P = 0.41); $I^2 = 1\%$
Test for overall effect: Z = 3.05 (P = 0.002)

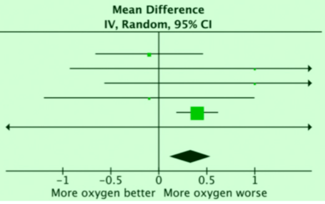

# Tertiary outcomes, any OT, w/o NCT00414726

## I: hospital-acquired infection, as shown by Chu et al. Lancet. 2018 Apr 28;391(10131):1693-1705; N = 6,249

| Study or Subgroup | Liberal Events | Liberal Total | Conservative Events | Conservative Total | Weight | Risk Ratio M-H, Random, 95% CI |
|---|---|---|---|---|---|---|
| Ali et al (2014) | 27 | 148 | 22 | 141 | 5.7% | 1.17 [0.70, 1.95] |
| NCT00414726 | 18 | 43 | 14 | 42 | 0.0% | 1.26 [0.72, 2.19] |
| Roffe et al (2017), SO2S | 400 | 2668 | 403 | 2668 | 93.2% | 0.99 [0.87, 1.13] |
| Stub et al (2015) | 7 | 312 | 4 | 312 | 1.0% | 1.75 [0.52, 5.92] |
| **Total (95% CI)** | | **3128** | | **3121** | **100.0%** | **1.01 [0.89, 1.14]** |
| Total events | 434 | | 429 | | | |

Heterogeneity: $Tau^2 = 0.00$; $Chi^2 = 1.16$, df = 2 (P = 0.56); $I^2 = 0\%$
Test for overall effect: Z = 0.12 (P = 0.90)

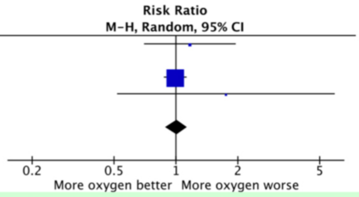


## II: same as I but numbers as reported in original publications; N = 8,646

| Study or Subgroup | Liberal Events | Liberal Total | Conservative Events | Conservative Total | Weight | Risk Ratio M-H, Random, 95% CI |
|---|---|---|---|---|---|---|
| Ali et al (2014) | 27 | 148 | 22 | 141 | 4.4% | 1.17 [0.70, 1.95] |
| Chiu et al (2006) | 4 | 17 | 15 | 29 | 0.0% | 0.45 [0.18, 1.15] |
| NCT00414726 | 18 | 43 | 14 | 42 | 0.0% | 1.26 [0.72, 2.19] |
| Roffe et al (2017), SO2S | 793 | 5267 | 403 | 2649 | 95.5% | 0.99 [0.89, 1.11] |
| Stub et al (2015) | 0 | 218 | 1 | 223 | 0.1% | 0.34 [0.01, 8.32] |
| **Total (95% CI)** | | **5633** | | **3013** | **100.0%** | **1.00 [0.89, 1.11]** |
| Total events | 820 | | 426 | | | |

Heterogeneity: $Tau^2 = 0.00$; $Chi^2 = 0.82$, df = 2 (P = 0.66); $I^2 = 0\%$
Test for overall effect: Z = 0.08 (P = 0.94)

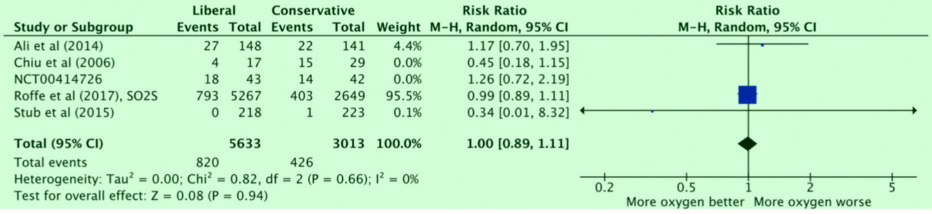


## III: hospital-acquired pneumonia, as shown by Chu et al.; N = 624

| Study or Subgroup | Liberal Events | Liberal Total | Conservative Events | Conservative Total | Weight | Risk Ratio M-H, Random, 95% CI |
|---|---|---|---|---|---|---|
| Stub et al (2015) | 2 | 312 | 1 | 312 | 100.0% | 2.00 [0.18, 21.94] |
| **Total (95% CI)** | | **312** | | **312** | **100.0%** | **2.00 [0.18, 21.94]** |
| Total events | 2 | | 1 | | | |

Heterogeneity: Not applicable
Test for overall effect: Z = 0.57 (P = 0.57)

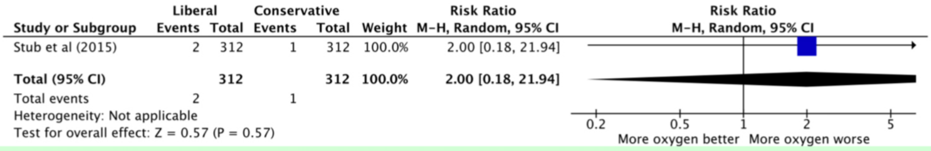


## IV: same as III but numbers as reported in original publications; N = 0

| Study or Subgroup | Liberal Events | Liberal Total | Conservative Events | Conservative Total | Weight | Risk Ratio M-H, Random, 95% CI |
|---|---|---|---|---|---|---|
| Chiu et al (2006) | 1 | 17 | 6 | 29 | 0.0% | 0.28 [0.04, 2.17] |
| Stub et al (2015) | not reported | | not reported | | | Not estimable |
| **Total (95% CI)** | | **0** | | **0** | | **Not estimable** |
| Total events | 0 | | 0 | | | |

Heterogeneity: Not applicable
Test for overall effect: Not applicable

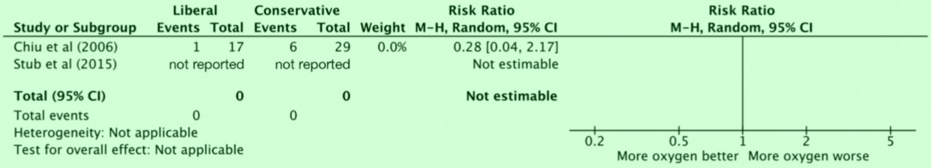


## V: respiratory serious adverse events; N = 0

| Study or Subgroup | Liberal Events | Liberal Total | Conservative Events | Conservative Total | Weight | Risk Ratio M-H, Random, 95% CI |
|---|---|---|---|---|---|---|
| Chiu et al (2006) | 3 | 17 | 8 | 29 | 0.0% | 0.64 [0.20, 2.09] |
| NCT00414726 | 3 | 43 | 2 | 42 | 0.0% | 1.47 [0.26, 8.33] |
| **Total (95% CI)** | | **0** | | **0** | | **Not estimable** |
| Total events | 0 | | 0 | | | |

Heterogeneity: Not applicable
Test for overall effect: Not applicable

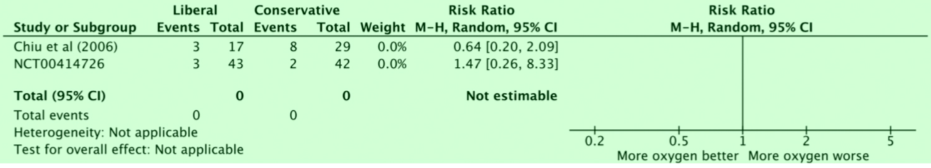


## VI: length of hospital stay, as shown by Chu et al.; N = 1,165

| Study or Subgroup | Liberal Mean | Liberal SD | Liberal Total | Conservative Mean | Conservative SD | Conservative Total | Weight | Mean Difference IV, Random, 95% CI |
|---|---|---|---|---|---|---|---|---|
| NCT00414726 | 8 | 4 | 43 | 7 | 5 | 42 | 0.0% | 1.00 [−0.93, 2.93] |
| Rawles et al (1976) | 16.2 | 5.4 | 80 | 14.9 | 5.3 | 77 | 6.8% | 1.30 [−0.37, 2.97] |
| Rønning et al (1999) | 8.6 | 6 | 292 | 8.7 | 7 | 258 | 14.5% | −0.10 [−1.20, 1.00] |
| Stub et al (2015) | 4 | 0.7 | 218 | 4 | 1.5 | 223 | 78.5% | 0.00 [−0.22, 0.22] |
| Young et al (2014), HOT OR NOT | 6.5 | 5.8 | 9 | 12.4 | 14.5 | 8 | 0.2% | −5.90 [−16.64, 4.84] |
| **Total (95% CI)** | | | **599** | | | **566** | **100.0%** | **0.06 [−0.39, 0.52]** |

Heterogeneity: $Tau^2 = 0.06$; $Chi^2 = 3.49$, df = 3 (P = 0.32); $I^2 = 14\%$
Test for overall effect: Z = 0.27 (P = 0.78)

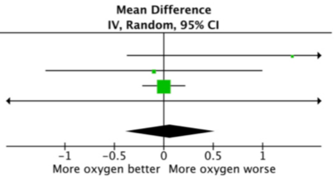


## VII: same as VI but numbers as reported in original publications; N = 1,208

| Study or Subgroup | Liberal Mean | Liberal SD | Liberal Total | Conservative Mean | Conservative SD | Conservative Total | Weight | Mean Difference IV, Random, 95% CI |
|---|---|---|---|---|---|---|---|---|
| NCT00414726 | 8 | 4 | 43 | 7 | 5 | 42 | 0.0% | 1.00 [−0.93, 2.93] |
| Rawles et al (1976) | 15 | 5.7 | 105 | 14 | 5.6 | 95 | 1.8% | 1.00 [−0.57, 2.57] |
| Rønning et al (1999) | 8.6 | 6 | 292 | 8.7 | 7 | 258 | 3.7% | −0.10 [−1.20, 1.00] |
| Stub et al (2015) | 4.4 | 0.7 | 218 | 4 | 1.5 | 223 | 94.4% | 0.40 [0.18, 0.62] |
| Young et al (2014), HOT OR NOT | 5.1 | 5 | 9 | 11.7 | 23.5 | 8 | 0.0% | −6.60 [−23.21, 10.01] |
| **Total (95% CI)** | | | **624** | | | **584** | **100.0%** | **0.39 [0.18, 0.60]** |

Heterogeneity: $Tau^2 = 0.00$; $Chi^2 = 2.04$, df = 3 (P = 0.56); $I^2 = 0\%$
Test for overall effect: Z = 3.62 (P = 0.0003)

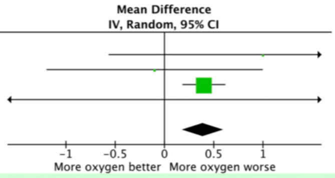


## VIII: length of hospital stay, all RCT; N = 7,837

| Study or Subgroup | Liberal Mean | Liberal SD | Liberal Total | Conservative Mean | Conservative SD | Conservative Total | Weight | Mean Difference IV, Random, 95% CI |
|---|---|---|---|---|---|---|---|---|
| Chiu et al (2006) | 17.2 | 15.3 | 16 | 21.1 | 16.2 | 22 | 0.0% | −3.90 [−14.00, 6.20] |
| Hofmann et al (2017), DETO2X-AMI | 3.3 | 9.6 | 3311 | 3.4 | 13.4 | 3318 | 21.9% | −0.10 [−0.66, 0.46] |
| NCT00414726 | 8 | 4 | 43 | 7 | 5 | 42 | 0.0% | 1.00 [−0.93, 2.93] |
| Rawles et al (1976) | 15 | 5.7 | 105 | 14 | 5.6 | 95 | 3.4% | 1.00 [−0.57, 2.57] |
| Rønning et al (1999) | 8.6 | 6 | 292 | 8.7 | 7 | 258 | 6.8% | −0.10 [−1.20, 1.00] |
| Stub et al (2015) | 4.4 | 0.7 | 218 | 4 | 1.5 | 223 | 67.9% | 0.40 [0.18, 0.62] |
| Young et al (2014), HOT OR NOT | 5.1 | 5 | 9 | 11.7 | 23.5 | 8 | 0.0% | −6.60 [−23.21, 10.01] |
| **Total (95% CI)** | | | **3935** | | | **3902** | **100.0%** | **0.27 [−0.02, 0.57]** |

Heterogeneity: $Tau^2 = 0.02$; $Chi^2 = 4.61$, df = 4 (P = 0.33); $I^2 = 13\%$
Test for overall effect: Z = 1.83 (P = 0.07)

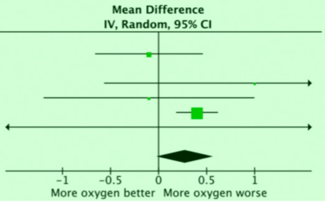

# Tertiary outcomes, FiO2 > 0.5

## I: hospital-acquired infection, as shown by Chu et al. Lancet. 2018 Apr 28;391(10131):1693-1705; N = 709

| Study or Subgroup | Liberal Events | Liberal Total | Conservative Events | Conservative Total | Weight | Risk Ratio M-H, Random, 95% CI |
|---|---|---|---|---|---|---|
| NCT00414726 | 18 | 43 | 14 | 42 | 82.9% | 1.26 [0.72, 2.19] |
| Stub et al (2015) | 7 | 312 | 4 | 312 | 17.1% | 1.75 [0.52, 5.92] |
| **Total (95% CI)** | | **355** | | **354** | **100.0%** | **1.33 [0.80, 2.20]** |
| Total events | 25 | | 18 | | | |

Heterogeneity: $Tau^2 = 0.00$; $Chi^2 = 0.25$, df = 1 (P = 0.62); $I^2 = 0\%$
Test for overall effect: Z = 1.11 (P = 0.27)

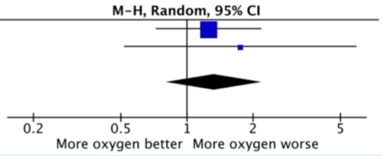


## II: same as I but numbers as reported in original publications; N = 526

| Study or Subgroup | Liberal Events | Liberal Total | Conservative Events | Conservative Total | Weight | Risk Ratio M-H, Random, 95% CI |
|---|---|---|---|---|---|---|
| NCT00414726 | 18 | 43 | 14 | 42 | 97.1% | 1.26 [0.72, 2.19] |
| Stub et al (2015) | 0 | 218 | 1 | 223 | 2.9% | 0.34 [0.01, 8.32] |
| **Total (95% CI)** | | **261** | | **265** | **100.0%** | **1.21 [0.70, 2.09]** |
| Total events | 18 | | 15 | | | |

Heterogeneity: $Tau^2 = 0.00$; $Chi^2 = 0.64$, df = 1 (P = 0.43); $I^2 = 0\%$
Test for overall effect: Z = 0.68 (P = 0.50)

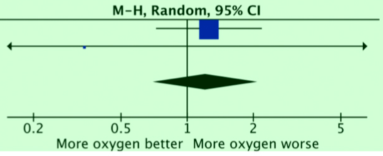


## III: hospital-acquired pneumonia, as shown by Chu et al.; N = 624

| Study or Subgroup | Liberal Events | Liberal Total | Conservative Events | Conservative Total | Weight | Risk Ratio M-H, Random, 95% CI |
|---|---|---|---|---|---|---|
| Stub et al (2015) | 2 | 312 | 1 | 312 | 100.0% | 2.00 [0.18, 21.94] |
| **Total (95% CI)** | | **312** | | **312** | **100.0%** | **2.00 [0.18, 21.94]** |
| Total events | 2 | | 1 | | | |

Heterogeneity: Not applicable
Test for overall effect: Z = 0.57 (P = 0.57)

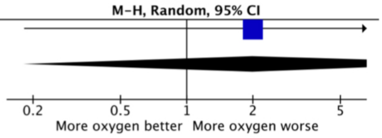


## IV: same as III but numbers as reported in original publications; N = 0

| Study or Subgroup | Liberal Events | Liberal Total | Conservative Events | Conservative Total | Weight | Risk Ratio M-H, Random, 95% CI |
|---|---|---|---|---|---|---|
| Stub et al (2015) | not reported | | not reported | | | Not estimable |
| **Total (95% CI)** | | **0** | | **0** | | **Not estimable** |
| Total events | 0 | | 0 | | | |

Heterogeneity: Not applicable
Test for overall effect: Not applicable

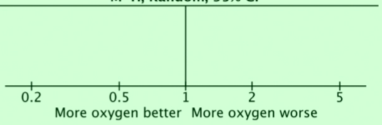


## V: respiratory serious adverse events; N = 85

| Study or Subgroup | Liberal Events | Liberal Total | Conservative Events | Conservative Total | Weight | Risk Ratio M-H, Random, 95% CI |
|---|---|---|---|---|---|---|
| NCT00414726 | 3 | 43 | 2 | 42 | 100.0% | 1.47 [0.26, 8.33] |
| **Total (95% CI)** | | **43** | | **42** | **100.0%** | **1.47 [0.26, 8.33]** |
| Total events | 3 | | 2 | | | |

Heterogeneity: Not applicable
Test for overall effect: Z = 0.43 (P = 0.67)

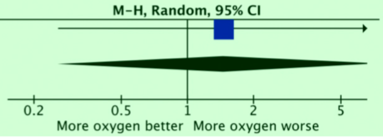


## VI: length of hospital stay, as shown by Chu et al.; N = 543

| Study or Subgroup | Liberal Mean | Liberal SD | Liberal Total | Conservative Mean | Conservative SD | Conservative Total | Weight | Mean Difference IV, Random, 95% CI |
|---|---|---|---|---|---|---|---|---|
| NCT00414726 | 8 | 4 | 43 | 7 | 5 | 42 | 8.6% | 1.00 [−0.93, 2.93] |
| Stub et al (2015) | 4 | 0.7 | 218 | 4 | 1.5 | 223 | 91.1% | 0.00 [−0.22, 0.22] |
| Young et al (2014), HOT OR NOT | 6.5 | 5.8 | 9 | 12.4 | 14.5 | 8 | 0.3% | −5.90 [−16.64, 4.84] |
| **Total (95% CI)** | | | **270** | | | **273** | **100.0%** | **0.07 [−0.52, 0.66]** |

Heterogeneity: $Tau^2 = 0.09$; $Chi^2 = 2.18$, df = 2 (P = 0.34); $I^2 = 8\%$
Test for overall effect: Z = 0.23 (P = 0.82)

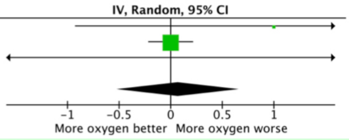


## VII: same as VI but numbers as reported in original publications; N = 543

| Study or Subgroup | Liberal Mean | Liberal SD | Liberal Total | Conservative Mean | Conservative SD | Conservative Total | Weight | Mean Difference IV, Random, 95% CI |
|---|---|---|---|---|---|---|---|---|
| NCT00414726 | 8 | 4 | 43 | 7 | 5 | 42 | 1.3% | 1.00 [−0.93, 2.93] |
| Stub et al (2015) | 4.4 | 0.7 | 218 | 4 | 1.5 | 223 | 98.7% | 0.40 [0.18, 0.62] |
| Young et al (2014), HOT OR NOT | 5.1 | 5 | 9 | 11.7 | 23.5 | 8 | 0.0% | −6.60 [−23.21, 10.01] |
| **Total (95% CI)** | | | **270** | | | **273** | **100.0%** | **0.41 [0.19, 0.62]** |

Heterogeneity: $Tau^2 = 0.00$; $Chi^2 = 1.05$, df = 2 (P = 0.59); $I^2 = 0\%$
Test for overall effect: Z = 3.68 (P = 0.0002)

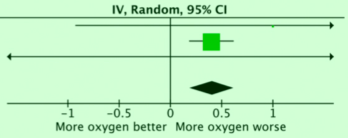

# Tertiary outcomes, FiO2 > 0.5, w/o NCT00414726

## I: hospital-acquired infection, as shown by Chu et al. Lancet. 2018 Apr 28;391(10131):1693-1705; N = 624

| Study or Subgroup | Liberal Events | Liberal Total | Conservative Events | Conservative Total | Weight | Risk Ratio M-H, Random, 95% CI |
|---|---|---|---|---|---|---|
| NCT00414726 | 18 | 43 | 14 | 42 | 0.0% | 1.26 [0.72, 2.19] |
| Stub et al (2015) | 7 | 312 | 4 | 312 | 100.0% | 1.75 [0.52, 5.92] |
| **Total (95% CI)** | | **312** | | **312** | **100.0%** | **1.75 [0.52, 5.92]** |
| Total events | 7 | | 4 | | | |

Heterogeneity: Not applicable
Test for overall effect: Z = 0.90 (P = 0.37)

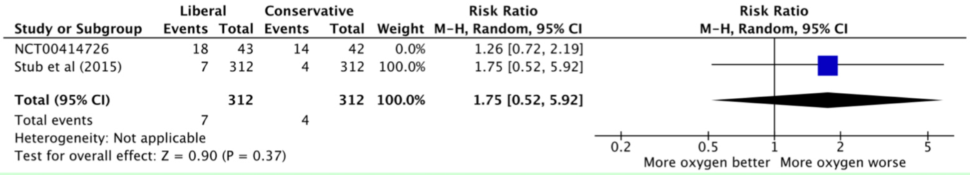


## II: same as I but numbers as reported in original publications; N = 441

| Study or Subgroup | Liberal Events | Liberal Total | Conservative Events | Conservative Total | Weight | Risk Ratio M-H, Random, 95% CI |
|---|---|---|---|---|---|---|
| NCT00414726 | 18 | 43 | 14 | 42 | 0.0% | 1.26 [0.72, 2.19] |
| Stub et al (2015) | 0 | 218 | 1 | 223 | 100.0% | 0.34 [0.01, 8.32] |
| **Total (95% CI)** | | **218** | | **223** | **100.0%** | **0.34 [0.01, 8.32]** |
| Total events | 0 | | 1 | | | |

Heterogeneity: Not applicable
Test for overall effect: Z = 0.66 (P = 0.51)

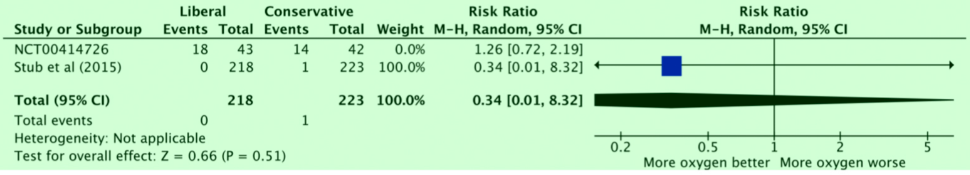


## III: hospital-acquired pneumonia, as shown by Chu et al.; N = 624

| Study or Subgroup | Liberal Events | Liberal Total | Conservative Events | Conservative Total | Weight | Risk Ratio M-H, Random, 95% CI |
|---|---|---|---|---|---|---|
| Stub et al (2015) | 2 | 312 | 1 | 312 | 100.0% | 2.00 [0.18, 21.94] |
| **Total (95% CI)** | | **312** | | **312** | **100.0%** | **2.00 [0.18, 21.94]** |
| Total events | 2 | | 1 | | | |

Heterogeneity: Not applicable
Test for overall effect: Z = 0.57 (P = 0.57)

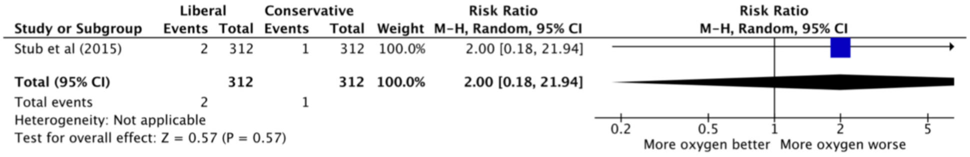


## IV: same as III but numbers as reported in original publications; N = 0

| Study or Subgroup | Liberal Events | Liberal Total | Conservative Events | Conservative Total | Weight | Risk Ratio M-H, Random, 95% CI |
|---|---|---|---|---|---|---|
| Stub et al (2015) | not reported | | not reported | | | Not estimable |
| **Total (95% CI)** | | **0** | | **0** | | **Not estimable** |
| Total events | 0 | | 0 | | | |

Heterogeneity: Not applicable
Test for overall effect: Not applicable

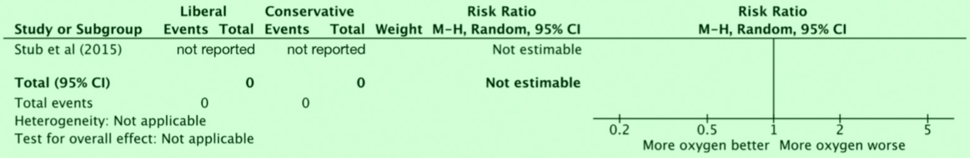


## V: respiratory serious adverse events; N = 0

| Study or Subgroup | Liberal Events | Liberal Total | Conservative Events | Conservative Total | Weight | Risk Ratio M-H, Random, 95% CI |
|---|---|---|---|---|---|---|
| NCT00414726 | 3 | 43 | 2 | 42 | 0.0% | 1.47 [0.26, 8.33] |
| **Total (95% CI)** | | **0** | | **0** | | **Not estimable** |
| Total events | 0 | | 0 | | | |

Heterogeneity: Not applicable
Test for overall effect: Not applicable

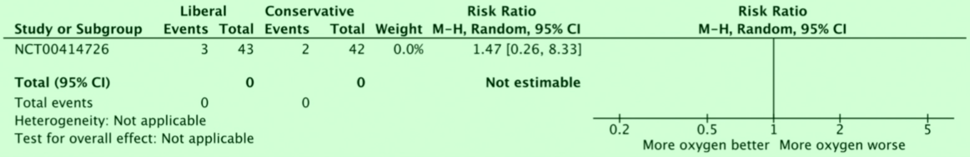


## VI: length of hospital stay, as shown by Chu et al.; N = 458

| Study or Subgroup | Liberal Mean | Liberal SD | Liberal Total | Conservative Mean | Conservative SD | Conservative Total | Weight | Mean Difference IV, Random, 95% CI |
|---|---|---|---|---|---|---|---|---|
| NCT00414726 | 8 | 4 | 43 | 7 | 5 | 42 | 0.0% | 1.00 [−0.93, 2.93] |
| Stub et al (2015) | 4 | 0.7 | 218 | 4 | 1.5 | 223 | 93.1% | 0.00 [−0.22, 0.22] |
| Young et al (2014), HOT OR NOT | 6.5 | 5.8 | 9 | 12.4 | 14.5 | 8 | 6.9% | −5.90 [−16.64, 4.84] |
| **Total (95% CI)** | | | **227** | | | **231** | **100.0%** | **−0.41 [−3.34, 2.52]** |

Heterogeneity: $Tau^2 = 2.39$; $Chi^2 = 1.16$, df = 1 (P = 0.28); $I^2 = 14\%$
Test for overall effect: Z = 0.27 (P = 0.79)

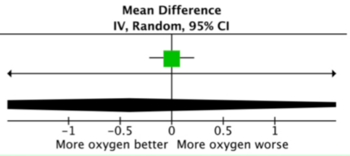


## VII: same as VI but numbers as reported in original publications; N = 458

| Study or Subgroup | Liberal Mean | Liberal SD | Liberal Total | Conservative Mean | Conservative SD | Conservative Total | Weight | Mean Difference IV, Random, 95% CI |
|---|---|---|---|---|---|---|---|---|
| NCT00414726 | 8 | 4 | 43 | 7 | 5 | 42 | 0.0% | 1.00 [−0.93, 2.93] |
| Stub et al (2015) | 4.4 | 0.7 | 218 | 4 | 1.5 | 223 | 100.0% | 0.40 [0.18, 0.62] |
| Young et al (2014), HOT OR NOT | 5.1 | 5 | 9 | 11.7 | 23.5 | 8 | 0.0% | −6.60 [−23.21, 10.01] |
| **Total (95% CI)** | | | **227** | | | **231** | **100.0%** | **0.40 [0.18, 0.62]** |

Heterogeneity: $Tau^2 = 0.00$; $Chi^2 = 0.68$, df = 1 (P = 0.41); $I^2 = 0\%$
Test for overall effect: Z = 3.59 (P = 0.0003)

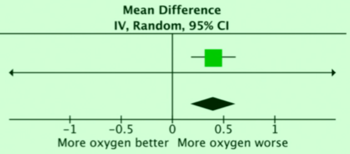

## Appendix 2 Statement of the German society for neuro-intensive care and emergency medicine (DGNI) concerning the suitability of mortality as a measure of quality in neuro-intensive treatment, including stroke care

Due to copyright restrictions the statement is not shown here. Please refer to:
https://www.dgni.de/670-sterblichkeitsrate-qualitaetsindikator-in-der-neurointensivmedizin.html

**Appendix 3 Summary of Singhal's Phase IIb clinical trial of NBHO in acute ischemic stroke [ClinicalTrials.gov identifier: NCT00414726], unpublished data**

Due to copyright restrictions the summary cannot be shown here. Please refer to the following publications which contain the relevant information:
https://n.neurology.org/content/80/7_Supplement/S02.001 and
https://n.neurology.org/content/80/7_Supplement/S42.001.

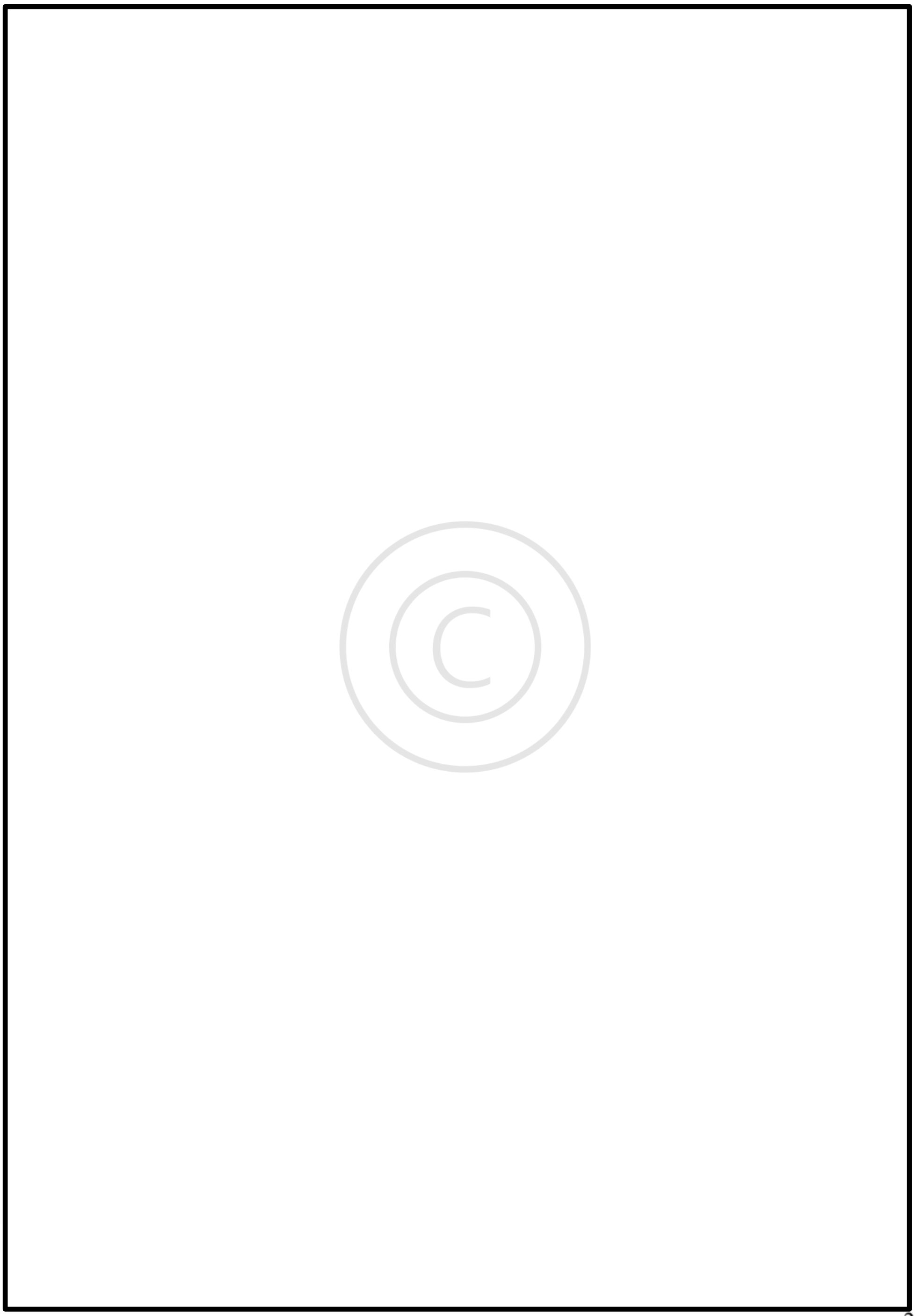

2

©

4

5

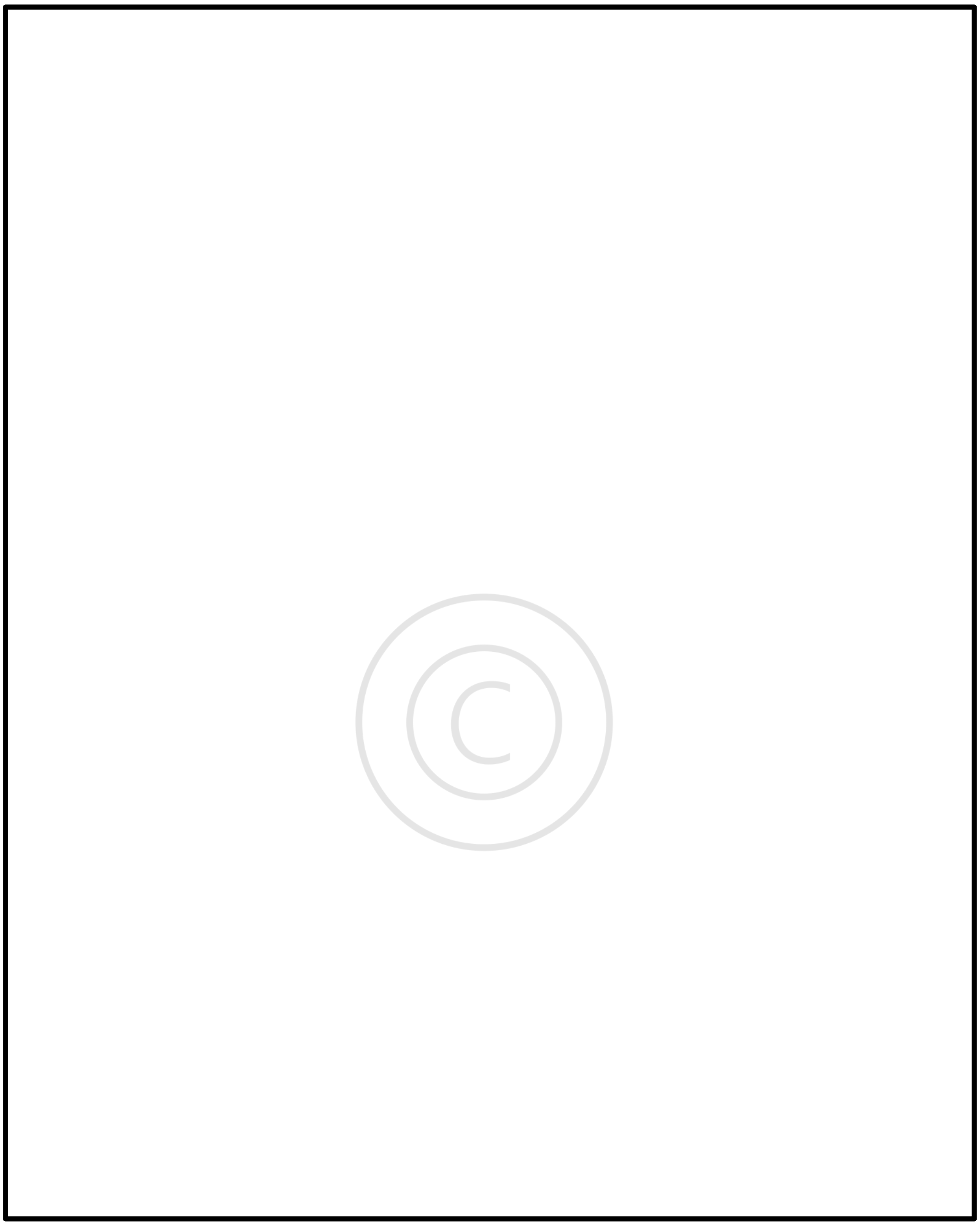

## Appendix 4 National Institutes of Health Stroke Scale (NIHSS)

Please refer to https://www.stroke.nih.gov/documents/NIH_Stroke_Scale_508C.pdf, due to copyright restrictions.

©

**Appendix 5 modified Rankin Scale (mRS)**

Please refer to table 1 in van Swieten, J.C., et al. 1988 [170], due to copyright restrictions.

**Appendix 6 Barthel Index**

Please refer to Mahoney, F.I. and D.W. Barthel 1965 [173].

## Appendix 7 Short Form of the Informant Questionnaire on Cognitive Decline in the Elderly (Short IQCODE)

Please refer to https://nceph.anu.edu.au/research/tools-resources/informant-questionnaire-cognitive-decline-elderly, due to copyright restrictions.

©

**Appendix 8 Montreal Cognitive Assessment (MOCA)**

Please refer to http://www.mocatest.org, due to copyright restrictions.

**Appendix 9 MoCA 5-min protocol**

Please refer to table 2 in Wong, A., et al. 2015 [179], due to copyright restrictions.

**Appendix 10 Stroke Impact Scale (SIS-16)**

Please refer to http://www.kumc.edu/school-of-medicine/preventive-medicine-and-public-health/research-and-community-engagement/stroke-impact-scale/sis-16.html, due to copyright restrictions.

**Appendix 11 EQ-5D-5L**

Please refer to https://euroqol.org/wp-content/uploads/2020/09/Sample_UK-English-EQ-5D-5L-Paper-Self-Complete-v1.2-ID-24700.pdf, due to copyright restrictions.

©

**Appendix 12 EQ-5D-5L Telephone interview**

Please refer to https://euroqol.org/eq-5d-instruments/eq-5d-5l-available-modes-of-administration/telephone-interview/, due to copyright restrictions.

©

## Appendix 133 Montgomery-Asberg Depression Scale (MADRS)

Due to copyright restrictions, please refer to Montgomery, S.A. and M. Asberg 1979 [183].

## Appendix 14 Heidelberg bleeding classification

Due to copyright restrictions, please refer to von Kummer, R., et al. 2015 [101].
